# Diversity, distribution and conservation of the terrestrial reptiles of Oman (Sauropsida, Squamata)

**DOI:** 10.1371/journal.pone.0190389

**Published:** 2018-02-07

**Authors:** Salvador Carranza, Meritxell Xipell, Pedro Tarroso, Andrew Gardner, Edwin Nicholas Arnold, Michael D. Robinson, Marc Simó-Riudalbas, Raquel Vasconcelos, Philip de Pous, Fèlix Amat, Jiří Šmíd, Roberto Sindaco, Margarita Metallinou, Johannes Els, Juan Manuel Pleguezuelos, Luis Machado, David Donaire, Gabriel Martínez, Joan Garcia-Porta, Tomáš Mazuch, Thomas Wilms, Jürgen Gebhart, Javier Aznar, Javier Gallego, Bernd-Michael Zwanzig, Daniel Fernández-Guiberteau, Theodore Papenfuss, Saleh Al Saadi, Ali Alghafri, Sultan Khalifa, Hamed Al Farqani, Salim Bait Bilal, Iman Sulaiman Alazri, Aziza Saud Al Adhoobi, Zeyana Salim Al Omairi, Mohammed Al Shariani, Ali Al Kiyumi, Thuraya Al Sariri, Ahmed Said Al Shukaili, Suleiman Nasser Al Akhzami

**Affiliations:** 1 Institute of Evolutionary Biology (CSIC-Universitat Pompeu Fabra), Passeig Marítim de la Barceloneta, Barcelona, Spain; 2 CIBIO, Centro de Investigação em Biodiversidade e Recursos Genéticos, Universidade do Porto, InBio Laboratório Associado, Vairão, Portugal; 3 School of Molecular Sciences, University of Western Australia, Crawley, Western Australia; 4 Department of Zoology, The Natural History Museum, London, United Kingdom; 5 Department of Biology, College of Science, Sultan Qaboos University, Al-Khod Muscat, Oman; 6 Àrea d’Herpetologia, Museu de Granollers–Ciències Naturals, Palaudàries, Granollers, Spain; 7 Department of Zoology, National Museum, Cirkusová, Prague, Czech Republic; 8 Museo Civico di Storia Naturale, Carmagnola (TO), Italy; 9 Breeding Centre for Endangered Arabian Wildlife, Environment and Protected Areas Authority, Sharjah, UAE; 10 Departamento de Zoología, Facultad de Ciencias, Universidad de Granada, Granada, Spain; 11 Departamento de Biologia, Faculdade de Ciencias da Universidade do Porto, Porto, Portugal; 12 Asociación Herpetológica Fretum Gaditanum, Mar Egeo, Jerez de la Fra, Spain; 13 Independent Researcher, Granada, Spain; 14 Independent Researcher, Dříteč, Czech Republic; 15 Allwetterzoo Münster, Münster, Germany; 16 Independent Researcher, Wiedergeltingen, Germany; 17 Independent Researcher, Madrid, Spain; 18 Independent Researcher, Almeria, Spain; 19 Independent Researcher, Storkow, Germany; 20 Escola de Natura de Parets, C/ Galende, Parets del Vallès, Spain; 21 Department of Integrative Biology, Museum of Vertebrate Zoology, University of California, Berkeley, CA, United States of America; 22 Ministry of Environment and Climate Affairs, Muscat, Oman; State Museum of Natural History, GERMANY

## Abstract

In the present work, we use an exceptional database including 5,359 records of 101 species of Oman’s terrestrial reptiles together with spatial tools to infer the spatial patterns of species richness and endemicity, to infer the habitat preference of each species and to better define conservation priorities, with especial focus on the effectiveness of the protected areas in preserving this unique arid fauna. Our results indicate that the sampling effort is not only remarkable from a taxonomic point of view, with multiple observations for most species, but also for the spatial coverage achieved. The observations are distributed almost continuously across the two-dimensional climatic space of Oman defined by the mean annual temperature and the total annual precipitation and across the Principal Component Analysis (PCA) of the multivariate climatic space and are well represented within 17 out of the 20 climatic clusters grouping 10% of the explained climatic variance defined by PC1 and PC2. Species richness is highest in the Hajar and Dhofar Mountains, two of the most biodiverse areas of the Arabian Peninsula, and endemic species richness is greatest in the Jebel Akhdar, the highest part of the Hajar Mountains. Oman’s 22 protected areas cover only 3.91% of the country, including within their limits 63.37% of terrestrial reptiles and 50% of all endemics. Our analyses show that large areas of the climatic space of Oman lie outside protected areas and that seven of the 20 climatic clusters are not protected at all. The results of the gap analysis indicate that most of the species are below the conservation target of 17% or even the less restrictive 12% of their total area within a protected area in order to be considered adequately protected. Therefore, an evaluation of the coverage of the current network of protected areas and the identification of priority protected areas for reptiles using reserve design algorithms are urgently needed. Our study also shows that more than half of the species are still pending of a definitive evaluation by the International Union for Conservation of Nature (IUCN).

## Introduction

Reptiles (a paraphyletic assemblage that includes all non-avian sauropsids) represent the world’s most diverse group of terrestrial vertebrates (10,544 species; [1]) and are a major component of the global biodiversity, remarkable from an ecological and evolutionary point of view [2]. Unlike other non-volant vertebrates, reptiles have dispersed widely including long-range trans-oceanic colonizations [3–5], offering ample possibilities for studying biogeography at many different levels [6, 7]. Being ectotherms, reptiles are greatly affected by the thermal landscapes of their habitat. Moreover, they are relatively easy to catch and sample for phylogenetic studies, they are widely represented in museum collections worldwide (which facilitates morphological studies) and, for many groups, there is abundant and detailed information on their taxonomy, distribution ranges and ecology [1, 7–10]. For these and other reasons they constitute excellent models for evolutionary, biogeographic, ecologic and conservation studies, and have been used as such for many decades [6, 11–13]. Despite their relevance, knowledge on the conservation status of reptiles lags behind that of birds, mammals and amphibians [14]. Only approximately 40% of the world’s reptile species have had their conservation status assessed by the International Union for Conservation of Nature (IUCN), and detailed analysis of extinction risks has been limited to a subset of 1,500 species [14, 15]. Even within reptiles, a study by Meiri and Chappel (2016) [15] using lizards and amphisbaenians (all reptiles excluding snakes, turtles, crocodiles and the tuatara) concluded that only 36% of the 6,338 described species have had their conservation status assessed. Whilst data deficient species (16%) played an important role, a major concern was the approximately 4,000 non-assessed species. According to Meiri and Chappel (2016) [15], there are important biases in the assessed species. These were more likely to be diurnal, to have larger body and clutch sizes, to have broader distributional and altitudinal ranges and to occur at more northerly latitudes. This highlights the necessity to improve the methods for species discovery and description, to increase our knowledge of reptile distributions, ecology and life history and to focus future work on under-assessed taxa inhabiting under-sampled regions such as the arid areas of North Africa and Arabia. Arid areas encompass a large portion of the Earth’s surface and are important for understanding global biodiversity patterns [13]. Generally, arid areas have relatively low species richness compared to other biomes such as tropical rainforests but they are often inhabited by many specialized deep lineages of highly adapted species and clades [16]. While much attention has been focused on arid areas from Australia and North America, centuries of fragile political stability and continuous clashes among its inhabitants have restrained research in North Africa and especially Arabia, leaving these massive arid biomes lesser known [17].

**Fig 1:**
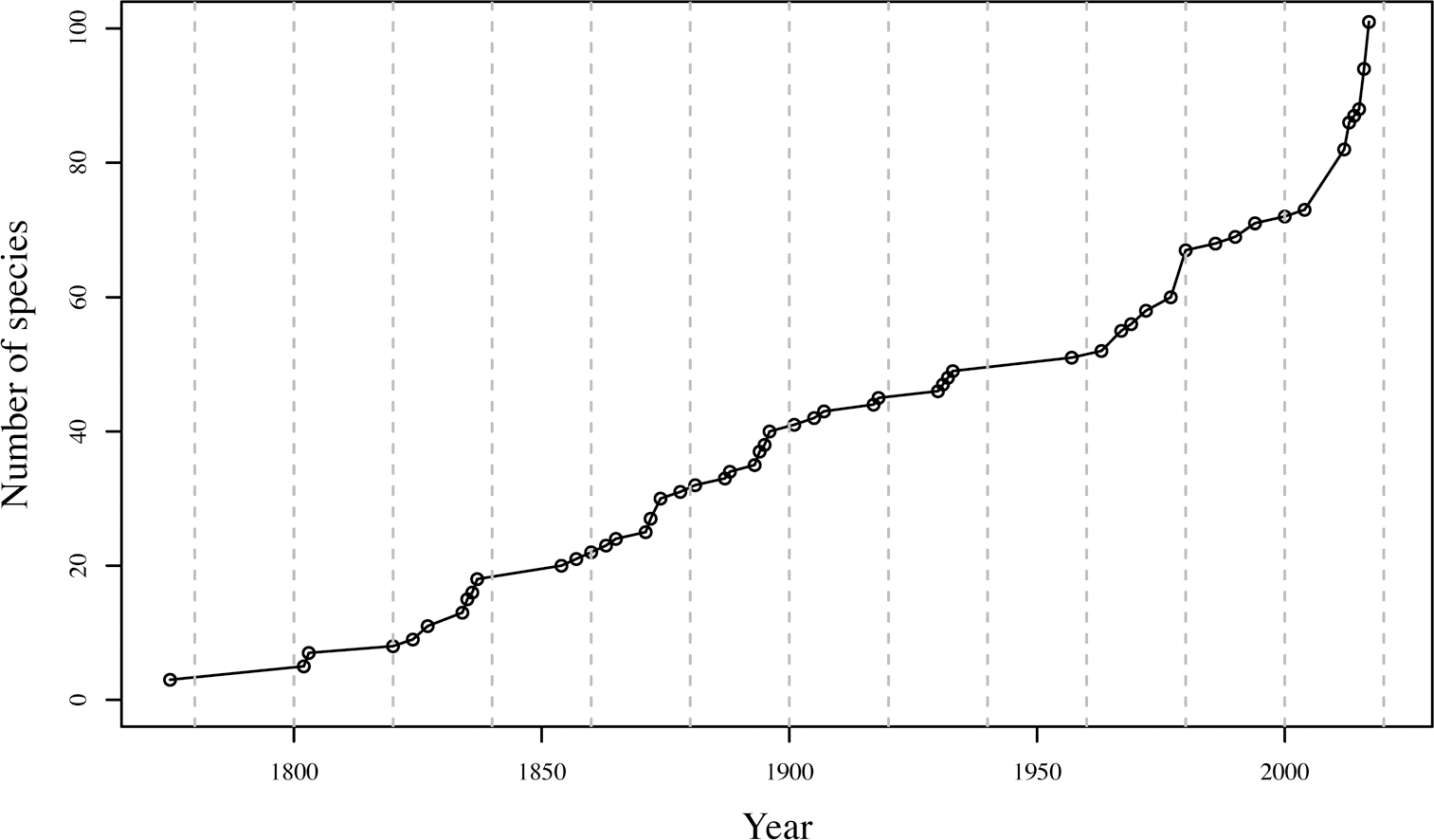
Number of species displayed in a cumulative way. Dots represent the years with species descriptions. Dashed lines divide the graph into intervals of 20 years. The last dot includes the species in the process of being described.

With 101 species (92 described and 9 in the process of being described), the Sultanate of Oman (Oman hereafter) harbors approximately 50% of the total number of reptile species in the Arabian Peninsula, ranking it as one of the countries with the highest reptile diversity [18, 19]. Due to an increased interest in the systematics of Oman’s reptiles, the pace of species descriptions and taxonomic knowledge has increased exponentially within the last 10 years and shows no sign of reaching a plateau (Fig 1) [18, 20–30]. Because of the past but especially the recent interest in Oman herpetology, the country’s current level of taxonomic knowledge ranks among the highest in Asia, with most of the groups having been investigated using integrative approaches including both morphological and molecular data analyzed with multivariate, phylogenetic and population genetic methods (see Carranza *et al*. (2016) [27] for a recent example). This approach has uncovered considerable levels of undescribed diversity [18, 20, 22, 27], including several remarkable examples of cryptic diversity [21, 28–30].

**Fig 2:**
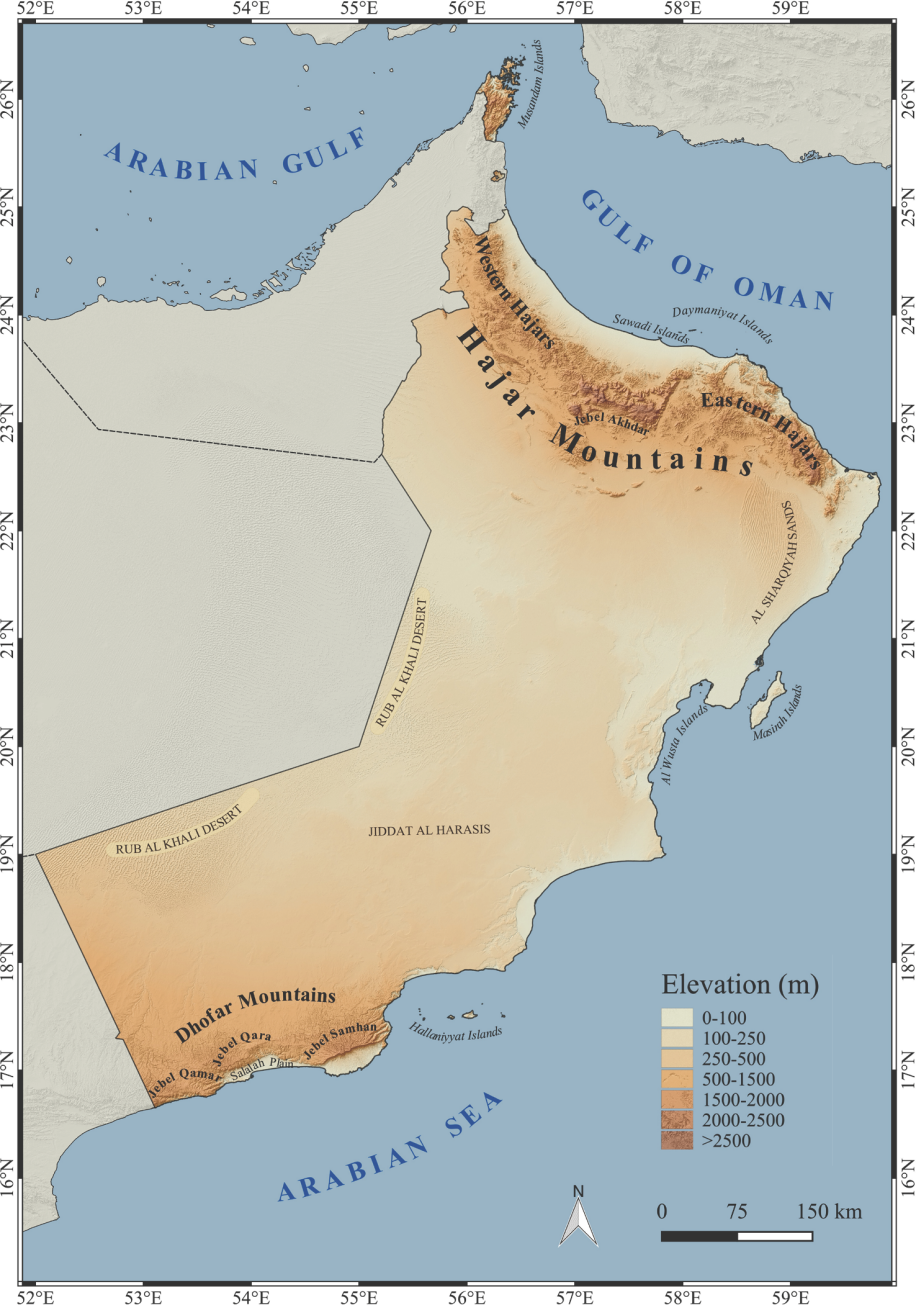
Physical map of Oman. Map of Oman showing topographical relief and names of the most relevant toponyms mentioned in this study.

Oman has two areas of great diversity, each with high levels of endemicity: the Hajar Mountains in the north and the Dhofar Mountains in the south. The Hajar Mountains are the highest in eastern Arabia, running from northwest to southeast for 650 km in an arc paralleling the Oman and United Arab Emirates (UAE) coastlines of the Gulf of Oman and bordered on the west by a very large desert ([31]; see Fig 2). The Hajar Mountains are the only area in eastern Arabia with habitats above 2000 m in elevation (Fig 2) and with an annual mean temperature of 13°C at the highest peaks (S1A Fig). Despite the altitude, annual rainfall is low (between 250–300 mm; S1B Fig) evapotranspiration is high and the almost treeless, barren nature of the terrain has made some authors to consider the Hajars a mountain desert (S1C Fig) [31, 32]. This situation contrasts sharply with the Dhofar Mountains of southern Oman, bounded to the north by the Rub al Khali (also known as the Empty Quarter), the largest desert in Arabia, to the south by the Arabian Sea and separated from the rest of Oman in the northeast by a desert steppe (Jiddat al Harasis) [33] (Fig 2). The summit of the mountain range is a relatively wide (10–25 km) flat plateau that runs for about 150 km mostly between 700 and 900 m in elevation, from the Jebel Qamar in the west, through the Jebel Qara in the central part, to the Jebel Samhan in the east (Fig 2). The highest point is over 2000 m in this latter massif, where temperatures reach the lowest values in southern Oman (Fig 2; S1A Fig) [33]. The Dhofar Mountains lie within the monsoon belt, and most rain falls in July and August, during the summer monsoon season resulting in the unique green vegetation on the south-facing (sea) side of the mountain range (S1C Fig), where the clouds form a variable belt along the coast from the Jebel Qamar to the Jebel Samhan that press against the mountain ridges causing frequent fog and light rain that does not surpass 200 mm per year ([34]; S1B Fig). Clouds only occasionally spill over the top of Jebel Qamar but on the much lower Jebel Qara they ride up to the summit [33]. However, the northern slopes across the whole mountain range are in a rain shadow. As a result, the north-facing (inland) side of the Dhofar Mountains is much drier and less vegetated than the lush south-facing side (S1B and S1C Fig). These climatic differences have played an important role in shaping the flora and fauna of this interesting biodiversity rich region (see [18] and references therein). Apart from these two mountain ranges, 60% of the approximately 330,000 km^2^ of Oman (area calculated from our GIS analysis) consist of a flat arid desert below 250 m in elevation (Fig 2; S1D Fig). These vast areas are mostly barren, vegetated by infrequent areas of widely spaced low perennial shrubs, interspersed between much broader areas of bare sand, gravel and rocks (S1C Fig). The mean annual temperatures are high (28°C; S1A Fig) and annual precipitation is very low (<150 mm; S1B Fig).

Oman borders with the UAE to the north and northwest, with Saudi Arabia to the west and with Yemen to the southwest (S2 Fig). The country is divided into 11 governorates, Musandam being the smallest, with just 1,805 km^2^, and Dhofar the largest, with 104,498 km^2^ and covering nearly 1/3 of the country (S3 Fig). The population is mainly concentrated in the capital, Muscat, and surrounding areas, making Muscat the most populated governorate, with 1,274,159 inhabitants. The country does not have a large network of tarmac roads and motorways but there is an extensive and excellent network of off-road trails that communicates villages, sometimes across desert areas, and connects oil refineries from the interior of the country with the main harbor in the Al Wusta governorate. Some of these roads have been used to survey the biodiversity of remote and previously inaccessible areas, sometimes resulting in unexpected discoveries [35].

**Fig 3:**
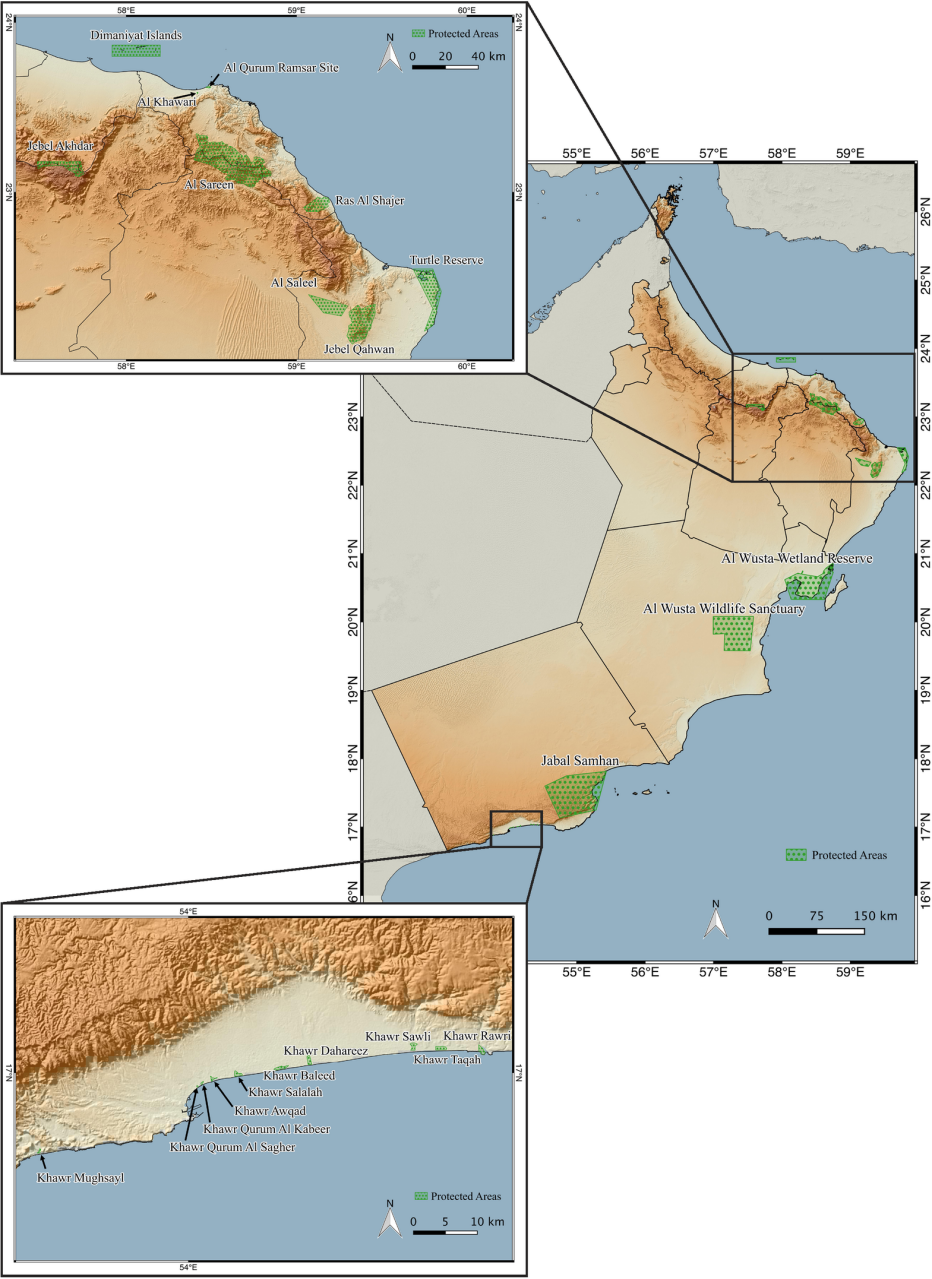
Map of protected areas of Oman. Topographical map of Oman showing the distribution, limits and names of the 22 protected areas. Information provided by the Ministry of Environment and Climate Affairs of Oman.

Oman has 22 protected areas (PAs) distributed across the country, of which Jebel Samhan Nature Reserve in the Dhofar Mountains is the largest (5,057.49 km^2^; 1.53% of the country), and the smallest being the Khawr Qurum Al Sagheer (0.04 km^2^; 0.00000012% of the country) (Fig 3; S1 Table). In total, the protected areas occupy an area of 12,916.52 km^2^, which corresponds to 3.91% of the total area of Oman.

In the present work, we use an unprecedented database including 5,359 records of 101 species of Oman’s terrestrial reptiles, together with spatial tools to assess the quality of our sampling, provide detailed species’ distribution maps and to infer the spatial patterns of species richness and endemicity. Using environmental datasets, we infer the habitat preference of each species. As a poorly assessed area for conservation priorities, additional data were provided to inform the conservation status and conservation threats of the reptiles of Oman, with a particular emphasis on the effectiveness of the protected areas to preserve this unique fauna.

## Materials and methods

### Ethics statement

No in vivo experiments were performed. The field study was carried out with the authorization of the governments of Oman (Ministry of Environment and Climate Affairs, MECA) with permits issued by the Nature Conservation Department of the Ministry of Environment and Climate Affairs, Oman (Refs: 08/2005; 16/2008; 38/2010; 12/2011; 13/2013; 21/2013; 37/2014; 31/2016). This research is not Institutional. As a result of the characteristics of this study and the total control and compliance with the laws, regulations and procedures of this kind of biodiversity studies in Oman, it did not need the approval by an Institutional Animal Care and Use Committee or ethics committee.

### Database

The database used in the present study included a total of 5,359 observations of terrestrial reptiles from Oman (Class Sauropsida, Order Squamata) obtained from different sources: 61.31% of the data (3,286 observations) results from fieldwork carried out by S. Carranza’s research team and collaborators between 2005 and 2015 as well as records of museum vouchers and bibliography data posterior to 2013 (bibliographic records up to 2013 had been gathered and included in Gardner (2013) [10]; see below). The remaining 38.72% of the data (2,073 observations) had been published by Gardner (2013) [10] and result from fieldwork carried out by A. Gardner himself during more than 25 years of living and working in the area, and from bibliographic data, museum vouchers and observations from many herpetologists that have collaborated with him. Gardner’s data ranges from the first scientific observations of Oman reptiles more than 40 years ago [36–40] up to 2013, the year of the publication of his book [10]. Prior to this study, both datasets were compared in order to eliminate any duplicated records.

### Taxonomy

This study gathered data for the terrestrial reptiles of Oman and therefore did not include observations from the marine snakes of the family Hydrophiidae or the marine turtles of the families Cheloniidae and Dermochelyidae, the only other non-avian sauropsids of Oman. In total, information is provided for 101 species (S2 Appendix), 18 species more than those reported by Gardner (2013) [10]. This increase of 17.8% in the number of species is the result of the intensive taxonomic work integrating information from both molecular and morphological data that has been carried out in Oman mainly by S. Carranza’s research team since 2005. This research has increased the taxonomic knowledge of Oman’s reptiles, which is already much higher than in any other country in Arabia and in most Asian countries [18, 20–30].

From the 101 species included in this study, 92 have been already described. However, in the database we have 9 species still in the process of being described that could be assigned to the generic level but, in the present work, we refer to them with the specific epithet “sp.”. In the two instances that more than one undescribed species exists for the same genus (*Pristurus* and *Mesalina*) we have used consecutive numbers: sp. 1, sp. 2, sp. 3, etc, for each putative new species. In a recent manuscript, the subspecies *Pristurus rupestris rupestris* endemic to the Hajar Mountains was shown to include high levels of undescribed diversity [28], with up to 14 candidate species recovered using species delimitation analyses with the software BP&P (Bayesian Phylogenetics and Phylogeography; [41]). These candidate species are grouped into five highly divergent lineages that diversified between 15.2 and 5.3 Ma. Although in some cases the limits between the 14 candidate species are not clear and will need to be investigated further, the five deep lineages are sympatric among them and do not share haplotypes in the nuclear genes c-*mos*, *ACM4*, *MC1R*, *RAG*-1 and *RAG*-2 analyzed [28]; a clear indication that they are different species. As a result of that, and pending a final taxonomic revision that clarifies the final number of species, in the present work we have considered each one of the five deep lineages of *P*. *r*. *rupestris* as different species: the nominal *P*. *rupestris*, *Pristurus* sp. 2, *Pristurus* sp. 3, *Pristurus* sp. 4 and *Pristurus* sp. 5, which correspond to lineages 4, 2, 3, 16 and 1 from Garcia-Porta *et al*. (2017) [28], respectively. Moreover, in the taxonomy used in the present study, there are two subspecies recognized within *Uromastyx aegyptia* (*U*. *a*. *microlepis* and *U*. *a*. *leptieni*); given their ecological differentiation in Oman (*U*. *a*. *leptieni* lives mainly in the Hajar Mountains and *U*. *a*. *microlepis* in the arid flat plains) but especially as a result of their relevance from a conservation point of view (*U*. *aegyptia* has been catalogued as Vulnerable by the IUCN) we have decided to analyze them independently as if they were independent species, although we maintain their subspecific status in the species’ accounts (S2 Appendix).

### IUCN Red List categories

The IUCN conservation categories for the taxa reported here have been obtained from the IUCN Red List of Threatened Species website (http://www.iucnredlist.org) and IUCN publications [19]. The conservation status of a species is not considered fully accepted until it is published in the IUCN Red List of Threatened Species. Therefore, we have differentiated between species with a classification already available from the IUCN from those evaluated by Cox *et al*. (2012) [19] but that are still pending of final approval and publication by the IUCN. There are 25 species in this situation, all of them classified as Low Concern (LC). These have been highlighted as LC* in the figures and tables to differentiate them from the 32 LC species already published in the IUCN website.

As a result of the taxonomy used in the present study, which includes taxonomic changes that occurred as recently as the 2^nd^ of August 2017 (description of *Ptyodactylus ruusaljibalicus*; [29]), and 9 species still in the process of being described (see above), many of the conservation categories for the Oman reptiles in the IUCN website or in Cox *et al*. (2012) [19] are incorrect and many taxa should be reevaluated. All 32 taxa affected by taxonomic changes have been classified as Not Evaluated (NE).

### Spatial data

The shapefile with the political borders of Oman and the 11 governorates as well as the shapefiles of the 22 protected areas of Oman were provided by the Ministry of Environment and Climate Affairs of the Government of Oman. Climate data were obtained from WorldClim (http://worldclim.org) and included 19 bioclimatic variables (BIO1-BIO19) with a spatial resolution of 30 arc-seconds (~1 km). These variables are derived from interpolated raw climate data (monthly minimum and maximum temperature plus precipitation) gathered from weather stations [42]. The elevation data from the Shuttle Radar Topography Mission at a spatial resolution of one arc-seconds (~30 m) was downloaded from the Reverb tool from NASA (http://reverb.echo.nasa.gov). Finally, a layer of land cover was downloaded from the International Steering Committee for Global Mapping (ISCGM) website (https://www.iscgm.org/gmd) at a resolution of 15 arc-seconds (~500 m) and, for the study area, nine types of habitats have been considered: Tree Open, Cropland, Cropland / Other vegetation mosaic, Shrub, Herbaceous, Sparse Vegetation, Bare Areas Gravel Rock, Bare Areas Sand, Urban. All data were stored and processed with geographic coordinates using WGS84 coordinate system, except when otherwise indicated.

### Distribution maps

Distribution maps for the 101 species were inferred using the same resolution as in Gardner (2013) [10]: a grid of 10 arc-minutes of latitude and longitude. This was done to facilitate the use of Gardner’s (2013) [10] data and because that size of grid (approximately 18x18 km; 324 km^2^) was appropriate for the sampling intensity (5,359 observations) and the total area of Oman (1,108 grid squares in a total area of approximately 330,000 km^2^). The vectorial grid was created for the extent of the study area in QGIS v. 2.6.1 Brighton [43] using the “vector grid” function. A QGIS add-on was written in Python programming language (JoinSplit; available at https://github.com/ptarroso/JoinSplit) to automate a spatial joint of the coordinates of species’ observations with the created grid, resulting in an individual vector grid for each species. The spatial data for the 101 species of terrestrial reptiles of Oman is available in S3 Appendix.

### Species ecological characterization

Species were characterized using the frequency of occurrence in elevation, climate and land cover variables, to reveal their ecological preferences. The elevation frequency distribution was obtained by crossing the elevation layer of 30 m resolution (see above) with the species occurrence data and it was represented by a histogram with bins of 100 m. The climate data for each species was extracted following the same procedure, but at a resolution of 1 km. The climate preference of each species was inferred within a two-dimensional climatic space of Oman defined by total annual precipitation (BIO12) and mean annual temperature (BIO1). Both variables provide a general information of the local climate and are ecologically relevant for many vertebrates, particularly for ectotherms. For each species, the land cover preference was inferred by calculating the distance of each observation to the nine different land cover types, and the distribution was shown as a boxplot. To provide metric distances, the land cover was projected using a World Equidistant Cylindrical projection.

The analyses and graphic output were produced with R programming environment using the functions “hist”, “plot” and “boxplot” [44, 45].

### Gap analysis

A gap analysis was used to calculate the proportion of the total distribution of each species that was included within a protected area [12]. The species’ distribution area was defined using two different approaches: 1) the area of occupancy (AOO) taking into account the distribution records at a resolution of 1 km^2^; and 2) the extent of occurrence (EOO), calculated by the sum of all 1 km^2^ pixels within the minimum convex polygon (MCP) defined by all the records and filtered by the species’ average altitude; a refined approach of the methodology used by the IUCN. The AOO used in the gap analysis was at a resolution of 1 km^2^; higher than the 4 km^2^ (2x2 km) suggested by the IUCN Standards and Petitions Subcommittee 2016 [46]. There are some very small protected areas that would have their areas overestimated with a grid of 4 km^2^, and consequently the area with some degree of protection would also be artificially increased. Moreover, most of the species of Oman reptiles are of very small body size and of low vagility, which justifies using a higher resolution. Nevertheless, a list of the AOO of each species at a resolution of 4 km^2^ is provided in S2 Table. Ten out of the 101 species did not have the minimum of three points necessary to infer a MCP and therefore their EOO was calculated using the distribution records at a resolution of 1 km^2^ (like in approach 1). In order to be considered “adequately protected” we have used the 17%, following Aichi Biodiversity Targets; [47, 48], and the less restrictive 12% [12, 49, 50] of the species’ total area within a protected area as conservation targets.

The analyses and the representation of the percentage of the total area that falls within a protected area were carried out using R programming language.

### Principal Component Analysis (PCA) of the climatic space of Oman and delimitation of the study area into climatic clusters

A Principal Component Analysis (PCA) was carried out including 12 of the 19 available climatic variables (BIO1, 4, 5–7, 10–13, 16, 18–19; http://www.worldclim.org/bioclim). Visual inspection of the climatic variables revealed atypical patterns in seven of them, likely due to the combination of low number of local weather stations and the interpolation method, and were therefore removed from the analysis. The PCA analysis, data escalation and the definition of the different climatic clusters in Oman was done in R using the function “prcomp”. The different climatic clusters were delimited so that they grouped 10% of the total explained variance in each component (PC1 and PC2; see S1 Appendix for the script in R). This clustering method divides the multidimensional space instead of focusing in features of the sampled points (e.g. density, distance or shape) to derive clusters. It assures that each cluster represents the same variance, independently of its geographical area or connectivity in the PCA space. As the method uses a fraction of the total variance to derive the cluster area, and as components are ordered by explained variance, the number of cluster divisions decreases from the first component to the last. The method is, however, dependent on the origin in the multidimensional space. Here we chose the 0 in each component as the origin for the clustering. In order to represent the climatic space of the 101 species and the protected areas, the climate of each species and that within the protected areas were extracted using QGIS and predicted for the above PCA space in R.

## Results

### Evaluation of the sampling effort

The evaluation of the sampling effort was carried out by analyzing the distribution of all observations using three different methods: 1) the spatial distribution of the samples in a grid of 10 arc-minutes of latitude and longitude covering the entire country; 2) by analyzing the distribution of all the samples in the climatic space of Oman defined by BIO12 and BIO1; and 3) by analyzing the distribution of all the samples in the PCA of the climatic space of Oman and within each one of the different climatic clusters.

**Fig 4:**
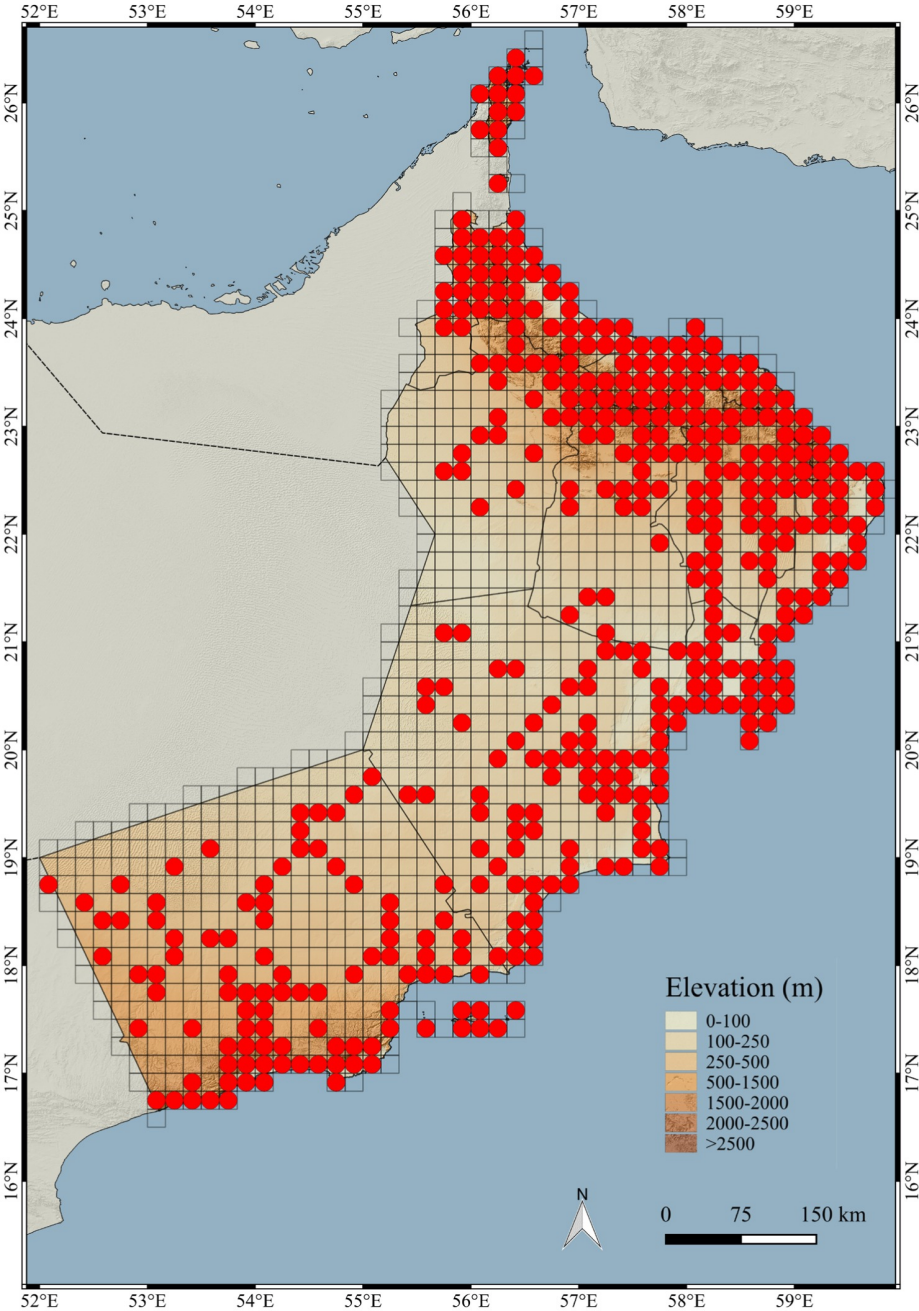
Map of Oman indicating the sampling effort. Grids of 10 arc-minutes (~18km) with observations (red dot). Empty grid cells are either due to no observation or no sampling.

The spatial analysis determined that 429 (38.72%) of the 1,108 grids were sampled for this study (Fig 4). As a result of the origin of the data (see Material and Methods), it was impossible to differentiate between grids that had been visited without success (no observations) and grids that had not been visited (unsampled grids). In any case, reptiles are a major component of the vertebrate fauna of Oman and in the records obtained by S. Carranza’s research team there was not a single grid that had been visited without success (no observations). This suggests that most of the grids of this study without presence data are the results of the lack of exploration. The sampled grids cover entirely the Hajar Mountains and adjoining areas (the coastal Batinah plain and the inland areas) and the Dhofar Mountains, including both the lush south-facing (sea) side and, to a lesser extent, the dry north-facing (land) side of the mountains (see Fig 2 and S1C Fig for geographical and land cover information). The eastern coastal area of the Arabian Sea is also well sampled. The less sampled areas are the Rub al Khali desert in the western part of the country, in the border area with Saudi Arabia, and the barren desert areas of Jiddat al Harasis, in the western inland side of Oman, between the Dhofar and the Hajar Mountains.

**Fig 5:**
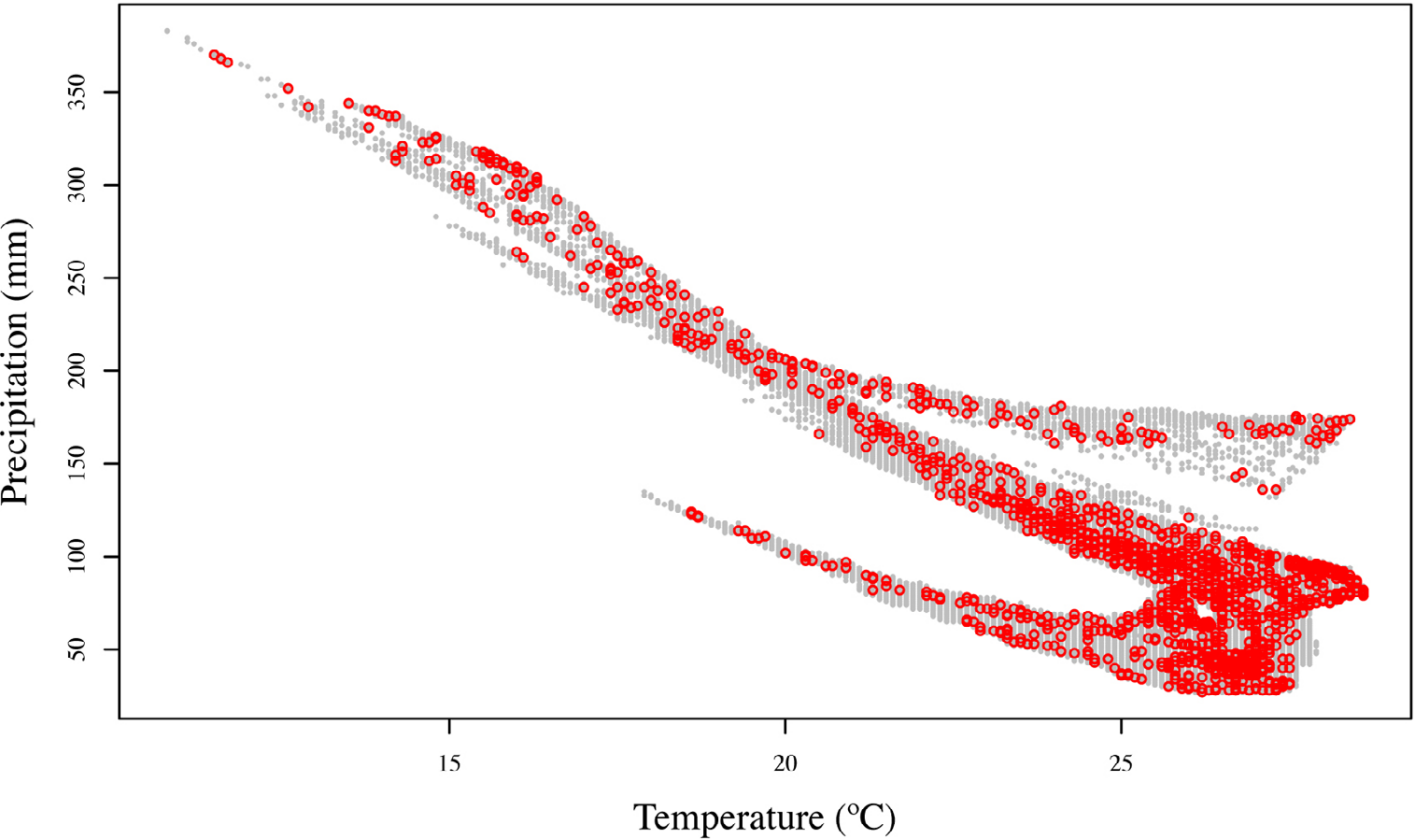
Two-dimensional climatic space of Oman (grey dots) defined by total annual precipitation (BIO12) and mean annual temperature (BIO1). Red dots represent the distribution of the 5,359 observations in this climatic space.

The distribution of all the observations in the two-dimensional climatic space of Oman (Fig 5) indicates that the samples are widespread across the whole climatic space defined by annual precipitation (BIO12) and mean annual temperature (BIO1), with no important gaps. The maximum number of observations accumulates in the area of the graph defined by high annual mean temperatures and low values of annual precipitation, which is also the most dominant climate in Oman, i.e. including most pixels. The area of Oman with lower mean annual temperatures (10–15°C) and relatively higher values of precipitation (around 350 mm/year), include fewer observations but also have less area available and are, thus, proportionally well sampled (Fig 5).

**Fig 6:**
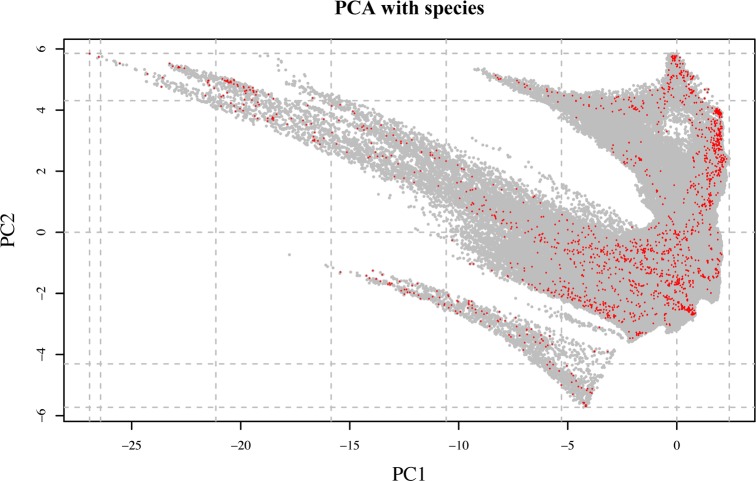
Principal Component Analysis (PCA) of the climatic space of Oman (grey dots) using 12 BIOCLIM variables. Dashed lines delimit the climatic clusters that group 10% of the explained variance by PC1 and PC2. Red dots represent the distribution of the 5,359 observations in the PCA of the climatic space.

**Fig 7:**
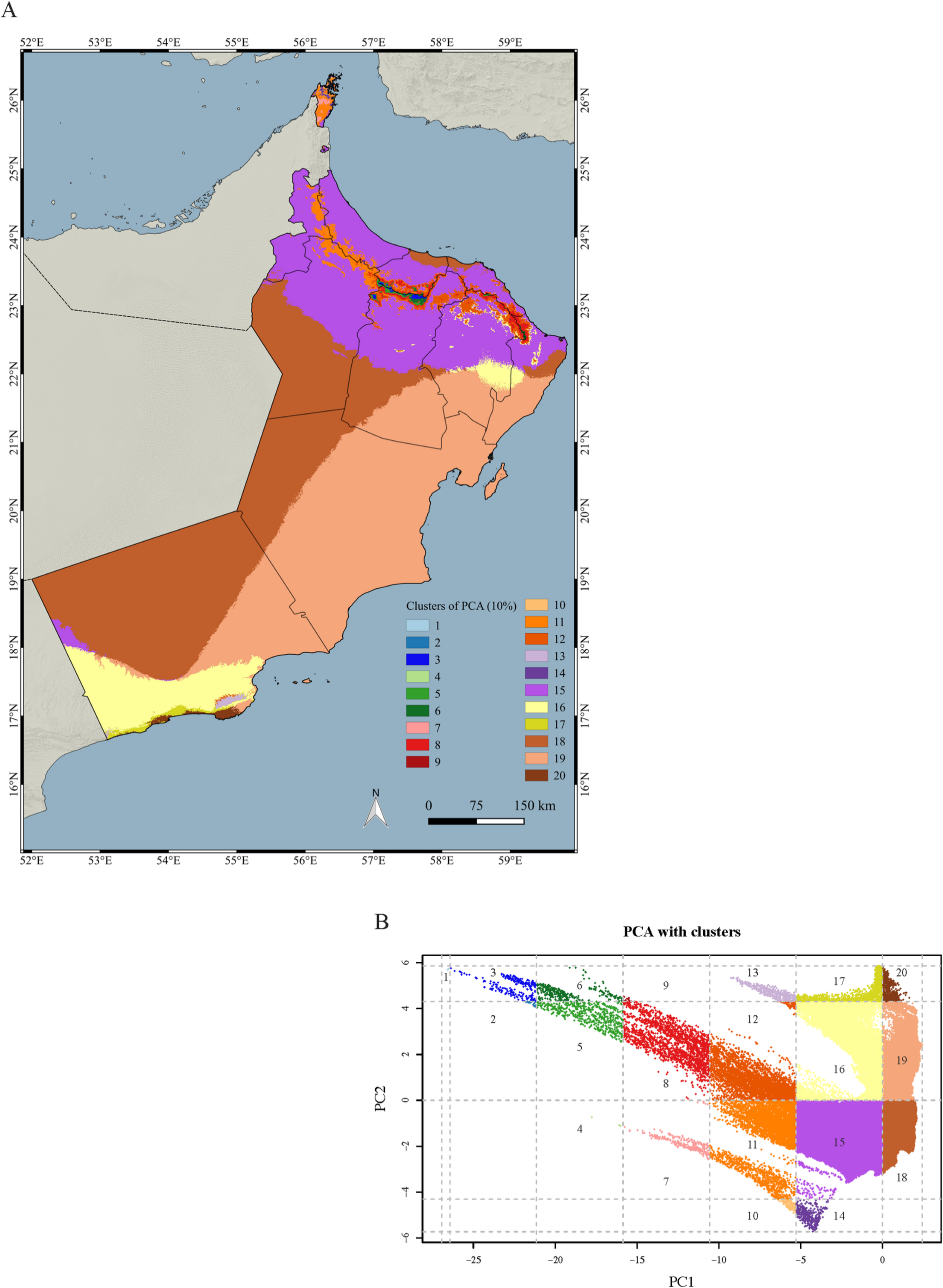
Climate variability of Oman. (A) Map showing the distribution and extension of the 20 climatic clusters of Oman identified in this study that group 10% of the explained variance by PC1 and PC2; (B) Principal Component Analysis (PCA) of the climatic space of Oman using 12 BIOCLIM variables and with the 20 climatic clusters that group 10% of the explained variance by PC1 and PC2 with the same colors as in the map. Clusters have been numbered from 1 to 20 with the following order: from left to right and from bottom to top.

As shown in Fig 6, the observations are also well distributed across the climatic space defined by the PCA (S3 Table). However, when the area was divided into clusters grouping 10% of the explained climatic variance by PC1 and PC2, not all 20 resulting clusters included observations and the distribution of the samples across the different clusters was also not proportional to cluster size (Figs 6 and 7; Table 1). Some of the clusters, such as clusters 18 and 19, cover very large areas across the whole country (Fig 7A), including the Rub al Khali Desert, Jiddat al Harasis and the Al Shariqiyah Sands. Other clusters are only present in northern or southern Oman (clusters 6 and 13, respectively) and some others, such as clusters 15 and 16, are present in both areas of Oman. The highest number of clusters is found in the Hajar Mountains with 15 clusters, including clusters 1 and 4, both with just 2.52 km^2^, the smallest of all 20 clusters. In the south, the highest number of clusters is found in the Dhofar Mountains and the Salalah Plain.

**Table 1:** List of all 20 climatic clusters that group 10% of the explained variance by PC1 and PC2 of the Principal Component Analysis (PCA) of the climatic space of Oman. The table shows the number of species present in each cluster, the number of localities of 30 arc-seconds of latitude and longitude sampled within each cluster, the percentage of each cluster sampled, and the area of each cluster.

Cluster	Number of species by cluster	Localities sampled	% sampled	Cluster area (Km^2^)
1	2	2	66.67%	2.52
2	0	0	0.00%	15.96
3	14	16	5.57%	241.02
4	0	0	0.00%	2.52
5	18	34	5.43%	525.71
6	19	38	10.33%	309.05
7	11	31	10.16%	256.14
8	20	47	2.06%	1915.58
9	0	0	0.00%	10.08
10	6	5	3.33%	125.97
11	22	115	1.80%	5356.24
12	25	57	1.09%	4407.27
13	13	16	2.45%	547.55
14	14	20	4.57%	367.83
15	62	538	0.71%	63522.47
16	53	110	0.38%	24019.96
17	27	70	4.20%	1399.95
18	59	210	0.16%	110749.46
19	58	380	0.29%	108287.17
20	32	61	4.84%	1058.99

The clusters can be divided into three categories: small (0–1,000 km^2^); medium, (1,000–20,000 km^2^); and large (>20,000 km^2^). The small category includes 11 clusters of which three have not been sampled (clusters 2, 4 and 9) and one (cluster 1) that is the best sampled of all clusters (Table 1). All five medium and four large clusters have been sampled and, although the effort was not equivalent to cluster size, it is clear from the data that the sampling effort is equivalent between all medium (2–5% of the sampled area) and large (0.2–0.7% of the sampled area) clusters.

Oman’s terrestrial reptiles are classified into seven main taxonomic groups. Geckos (Gekkonidae, Phyllodactylidae, Sphaerodactylidae) are the most diverse one with 44.55% of all the species and 80% of the endemic species, followed by the snakes (Boidae, Colubridae, Elapidae, Lamprophiidae, Leptotyphlopidae, Typhlopidae, Viperidae) with 20.79% of the species and no endemic species (S4A Fig; S2 Appendix). Three other groups, agamids (Agamidae, 12.87%), skinks (Scincidae, 6.94%) and lacertids (Lacertidae, 12.87%) represent 30% of the diversity and 20% of the endemic species (10%, 0%, and 10%, respectively). The least diverse groups are varanids (Varanidae) and amphisbaenids (Amphisbaenidae), representing only 2% of the diversity and without any endemic species. These percentages among taxonomic groups are not proportional to the number of observations in our database. The most discrepant groups are the geckos, with 64.11% of the observations, and snakes, with only 11.92% of the observations (S4B Fig). The sampling effort in the remaining groups seems proportional to their diversity, with agamids representing 9.72% of the observations, skinks 4.12%, lacertids 9.31% and varanids together with amphisbaenids 0.82% (S4 Fig; S2 Appendix).

### Distribution of species richness

**Fig 8:**
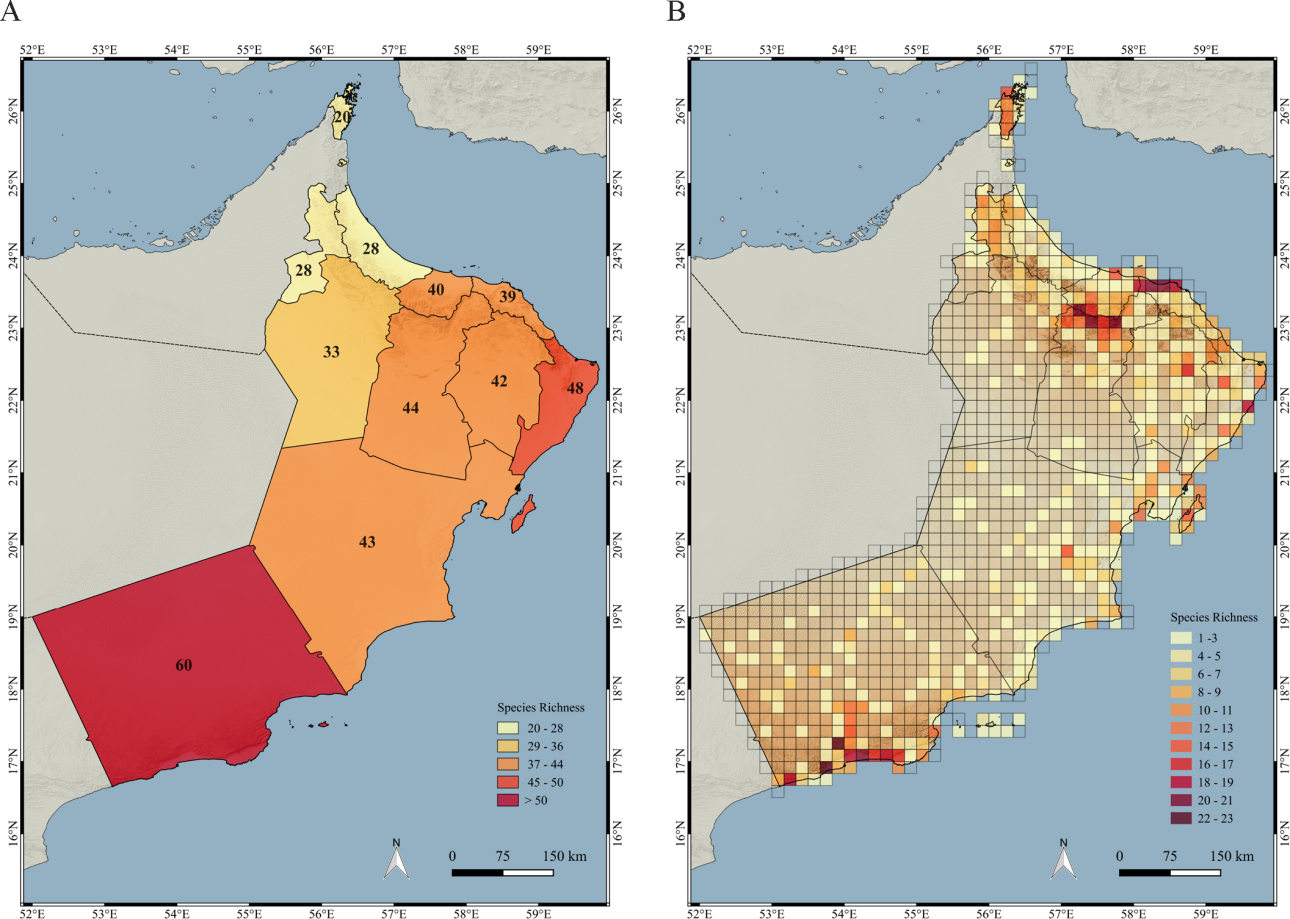
Maps of species richness. (A) Species richness by governorate; (B) species richness by grids of 10 arc-minutes of latitude and longitude.

The distribution of species richness was calculated for all 11 governorates and at a finer scale (10 arc-minutes grid). Dhofar has the highest diversity, with 60 species, and is followed by Ash Sharqiyyah South with 48. The remaining governorates have less than 45 species, being Musandam (20), Al Buraymi (28) and Al Batinah North (28) the governorates with the lowest number of species (Fig 8A; S2 Appendix). When the species richness was analyzed using the 10 arc-minutes grid, the highest number of species appeared in the high elevation parts of the Hajar Mountains (Jebel Akhdar), the coastal area and wadis around the capital (Muscat) and in the Dhofar Mountains and the Salalah Plain in the south (Fig 8B; see also Fig 2).

As a result of their relevance from a human health and conservation point of view, the species richness of the nine venomous snake species of Oman was also analyzed for each governorate and each sampled grid of 10 arc-minutes. According to S5A Fig, it is clear that Dhofar is the governorate with the highest number of venomous species (six species), followed by Al Wusta (four species). With just one species, *Echis omanensis*, al Buraymi is the governorate with the lowest number of venomous snakes. The analyses at a finer scale (10 arc-minutes grid) reveals that species richness is also highest in Dhofar; more specifically in some areas of the Dhofar Mountains (Jebel Qara) (S5B Fig). Of the nine venomous snakes, *Naja arabica*, *Atractaspis andersonii* and *Bitis arietans* are only found in Dhofar, *Echis coloratus* and *E*. *khosatzkii* are found in Dhofar and Al Wusta, *Cerastes gasperettii gasperettii* and *E*. *carinatus sochureki* have wider distributions, the first avoiding the north and the second the south of Oman. Finally, *E*. *omanensis* and *Pseudocerastes persicus* are restricted to the Hajar Mountains and immediate surrounding areas (S2 Appendix).

### Island diversity

Islands represent outstanding examples of biodiversity, endemicity and species extinctions. Island species are often unique, yet are highly vulnerable to novel disturbances, such as invasive species. As the risk of extinction is highest on islands it is important to know their diversity in order to monitor and conserve it. Of the 101 species of Oman reptiles, 29 (28.71%) are found on islands (S1 Table; S2 Appendix) but only one, *Hemidactylus masirahensis*, is an island endemic (Masirah Island, in the Arabian Sea, 14,3 km off the northeast coast of Oman; see Fig 2). Masirah is the largest Oman island (approx. 700 km^2^) and the one that present the highest number of species, 19, which represents 65.51% of all island species and 18.81% of all species of Oman’s terrestrial reptiles (S1 Table).

### Distribution and richness of endemic species

**Fig 9:**
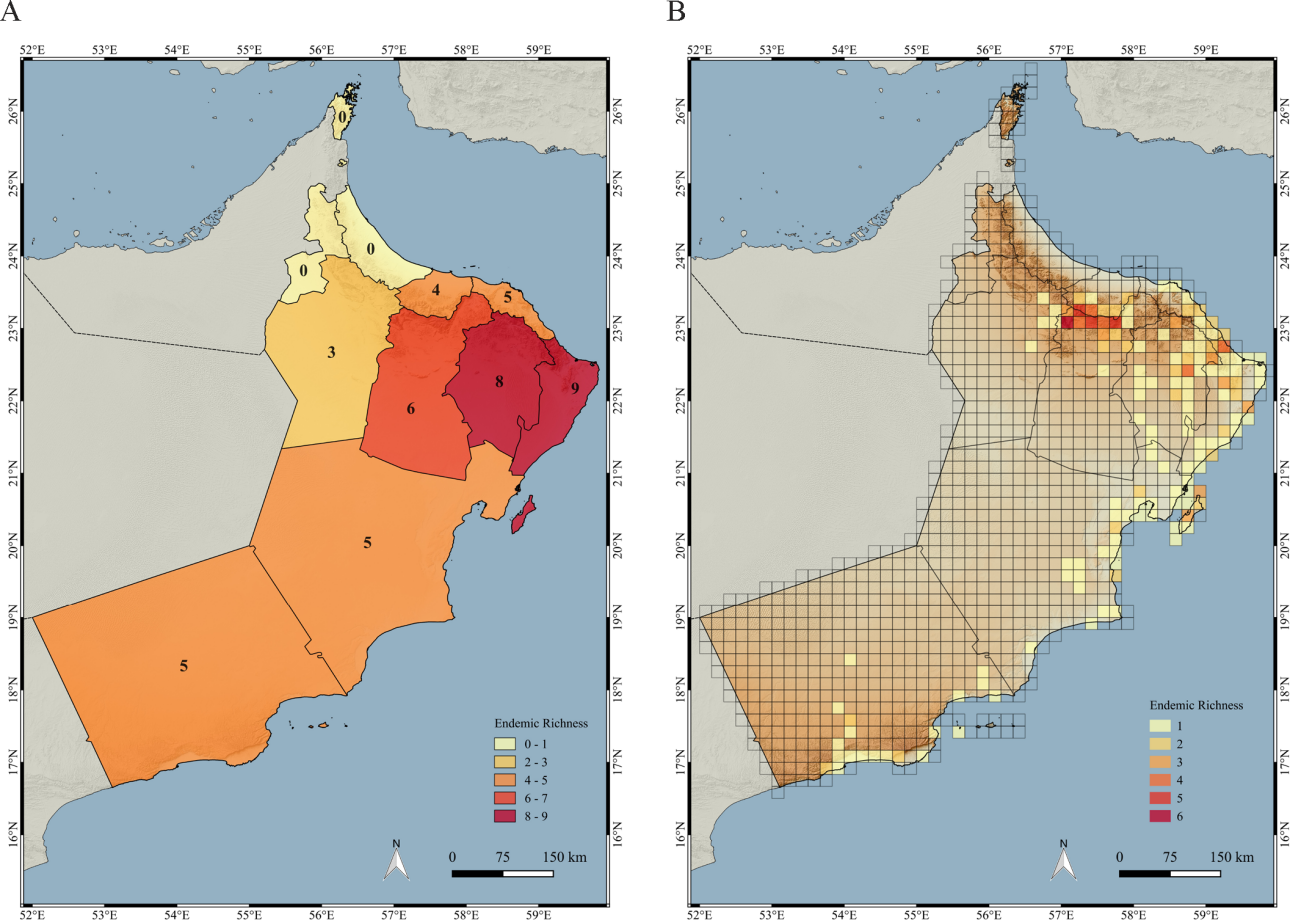
Maps of endemicity. (A) Endemic species richness by governorate; (B) Endemic species richness by grids of 10 arc-minutes of latitude and longitude.

In total, 20 (19.8%) species are endemic to Oman. The governorate with the highest endemicity is Ash Sharqiyyah South, in the extreme northeast part of the country, which has nine out of the 20 endemic species (45%; see S1 Table). It is followed very closely by Ash Sharqiyyah North, with eight endemic species (Fig 9A; see also S2 Fig). However, when the endemicity richness is analyzed at a finer scale (10 arc-minutes grid), the areas with the highest values are found in the Hajar Mountains in northern Oman and more specifically in the Jebel Akhdar, that contains as many as six endemic species in some grids. Other grids with relatively high levels of endemicity richness are found in the northeast of the country, including Masirah Island. Endemicity is not very high in Dhofar, with the highest values of grid endemicity being two grids situated in the south-facing (sea) side of the Dhofar Mountains and one grid on the north-facing (land) side of the mountains (Fig 9B).

### Analysis of the IUCN Red List conservation categories

In this study, the IUCN Red List categories of the reptiles of Oman included: NE (Not Evaluated), DD (Data Deficient), LC (Least Concern), NT (Near Threatened) and VU (Vulnerable). There are no EN (Endangered), CR (Critically Endangered), EW (Extinct in the Wild) or EX (Extinct) species. Therefore, in Oman, the only threatened taxa are five VU species (S2 Appendix). The remaining taxa include 5 DD, 58 LC, 1 NT (*Pristurus gallagheri*) and 32 NE species, of which nine are already described endemic species and five are endemic species in the process of being described. Because of the small distribution for most of these endemic NE species, some of them will be assessed in the future as threatened species, through the B criteria (geographic range) in the red listing process. Therefore, 14 out of the 20 endemic species in our dataset (70%) are classified as NE. Of the 58 LC species, 32 have had their classifications already published in the IUCN Red List of Threatened species, while 26 LC* were evaluated by Cox *et al*. (2012) [19] but are still pending of final approval from IUCN and publication (S6 Fig). The proportion of each one of the conservation categories by governorate is shown in S7 Fig. All governorates with the exception of Musandam and Al Batinah North have VU species, and Al Batinah South and Ad Dakhliyyah also have the single NT species. Only two governorates, Ash Sharqiyyah South and Al Wusta have DD species and all the governorates have NE species, with Ash Sharqiyyah South presenting the highest number of NE species (14) and Al Buraymi the lowest (4) (S1 Table).

### Analysis of protected areas

**Fig 10:**
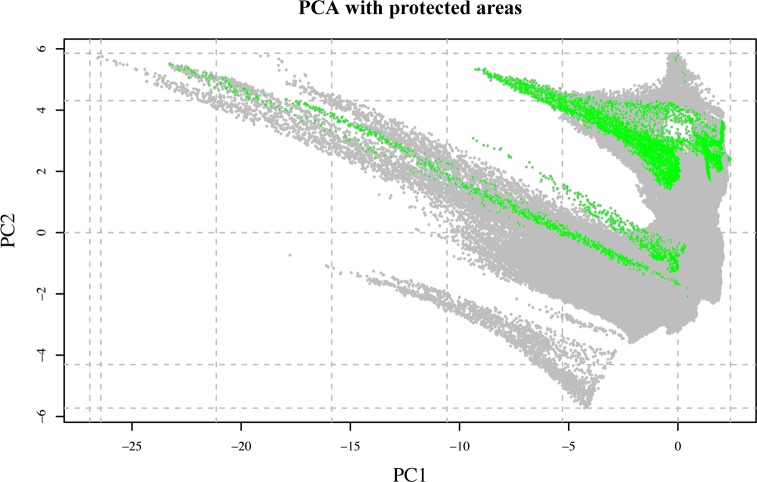
Principal Component Analysis (PCA) of the climatic space of Oman (grey dots) using 12 BIOCLIM variables. Dashed lines delimit the climatic clusters that group 10% of the explained variance by PC1 and PC2. Green dots represent the climatic space of protected areas.

A list providing relevant information on the 22 protected areas of Oman is shown on Table 2. Several analyses were carried out in order to evaluate the effectiveness of the 22 protected areas of Oman. The first analysis included the visualization of the distribution of the protected areas in the PCA of the climatic space of Oman and within each one of the 20 climatic clusters defined by 10% of climatic variance (see above). As shown in Fig 10, there are large parts of the climatic space of Oman outside protected areas and, as result of that, there are seven clusters (clusters 1, 2, 4, 7, 9, 10 and 14) not represented in the protected areas.

**Table 2:** Oman protected areas. List of all 22 protected areas of Oman including their management type, area information and date of establishment.

Area Name	Management Type	Area (Km^2^)	Perimeter (Km)	Date
Al Sareen	Especially Important Area	785.10	165.91	1976
Ras Al Shajer	Especially Important Area	102.07	47.93	1985
Khawr Salalah	Especially Important Area	0.67	3.93	1986
Al Wusta Wildlife Sanctuary	Wildlife Sanctuary	3013.24	239.44	1994
Dimaniyat Islands	Nature Reserve	233.64	77.84	1996
Turtle Reserve	Coastal Reserve	302.25	103.26	1996
Jabal Samhan	Nature Reserve	5057.49	287.35	1997
Khawr Mughsayl	Reserve	0.16	2.26	1997
Khawr Baleed	Reserve	0.77	5.52	1997
Khawr Sawli	Reserve	0.83	4.51	1997
Khawr Dahareez	Reserve	0.81	4.24	1997
Khawr Taqah	Reserve	0.97	4.58	1997
Khawr Rawri	Reserve	0.87	4.35	1997
Khawr Awqad	Reserve	0.27	3.50	1997
Khawr Qurum Al Sagher	Reserve	0.04	0.83	1997
Khawr Qurum Al Kabeer	Reserve	0.11	1.52	1997
Al Saleel	National Park	159.43	61.77	1997
Al Khawair	Especially Important Area	0.32	4.27	2006
Jebel Akhdar	Scenic Reserve	133.06	76.00	2011
Al Qurum Ramsar Site	Ramsar Site	1.91	8.34	2013
Al Wusta Wetland Reserve	Wetland Reserve	2809.10	253.13	2014
Jebel Qahwan	Nature Reserve	313.41	80.22	2014

**Fig 11:**
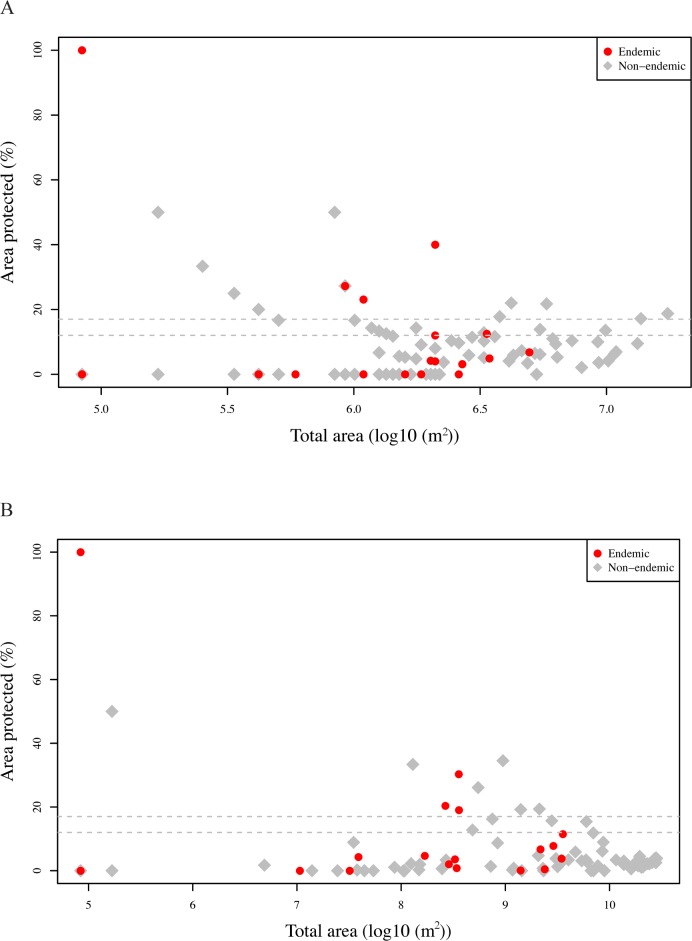
Percentage of the species’ distribution area included within a protected area. Dashed lines indicate the conservation target of 17% and 12% of the total species’ distribution area within a protected area. (A) The extent of species occurrence was defined using the presence-absence in every pixel of 1 km x 1 km. (B) The extent of species occurrence was defined using a minimum convex polygon (MCP) of the observations filtered by the species’ average altitude.

The results of the gap analyses using two different approaches to calculate the presence data (AOO and EOO, see above) are given in S4 and S5 Tables, respectively and are shown in Fig 11. These indicate that 15 (AOO approach) and 10 (EOO approach) species have more than 17% of their distribution within a protected area, and 28 (AOO approach) and 14 (EOO approach) reach the 12% conservation target. As for the endemics, the situation is very concerning, as only four (both approaches) reach the 17% target and only six (AOO approach) and four (EOO approach) the 12% target.

Combining the results of both approaches (S4 and S5 Tables), there is a total of 20 species (five endemics) that are above the 17% target and 32 (seven endemic) that are above the 12% target. As for the VU species (*Asaccus montanus–*endemic, *Acanthodactylus felicis*, *Uromastyx a*. *leptieni*, *U*. *a*. *microlepis*, and *U*. *thomasi–*endemic), there are only two species (*A*. *felicis* EOO approach and *U*. *thomasi* AOO approach) that are above the 17% target, the same species that are above the 12% target. More importantly, 34 species (nine endemic) (AOO approach) and 20 species (five endemic) (EOO approach) have 0% of their distribution within a protected area and therefore failed completely to reach conservation targets (see S4 and S5 Tables).

Despite the rather low proportion of the distribution ranges of all the species included within protected areas (only 32 and 20 species are above the 12% and 17% protection target, respectively; S4 and S5 Tables; Fig 11), and the fact that the protected areas only represent 3.91% of the total area of Oman, and are not represented in seven out of the 20 climatic clusters, these include within their limits 64 of the 101 species (63.37%), 10 of the 20 endemic species (50%), seven of the nine venomous species (77.78%) and three of the five threatened species (60%) (S1 Table).

### Ecological analysis of Oman reptiles

The ecological preferences of all the species of Oman reptiles were characterized using the frequency of occurrence in the elevation, climate, and land cover variables (see material and methods) and revealed some generalist and specialist species (S2 Appendix). The skink *Trachylepis tessellata* is distributed across the Hajar and Dhofar Mountains in the north and south of the country, respectively, and in Masirah Island. The species is found from sea level up to 1900 m in elevation, covers most of the bioclimatic area defined by the mean annual temperature and annual precipitation and is not far from most land cover types, with a slight preference for bare areas with gravel and rocks. It is the only lizard species with such generalist ecological preferences. Of the 21 species of snakes, three show a similar generalist pattern: *Psammophis schokari*, *Telescopus dhara dhara* and *Platyceps rhodorachis rhodorachis*, the latter having the greatest elevational range (from sea level up to 2600 m) and a distribution across the entire climatic space of Oman. The remaining species of reptiles are more restricted in elevation, climatic space and land cover preferences and linked to specific areas such as coastal deserts, inland deserts, arid mountains (high and low elevation), tropical mountains, islands and coastal plains, among others.

Within the agamids, the genus *Phrynocephalus* is restricted to the arid areas of Oman with high temperatures and low precipitation and has never been found above 400 m in elevation. The genus *Pseudotrapelus* has two morphologically very similar species with very different bioclimatic and elevation preferences. *Pseudotrapelus dhofarensis* is found in the Dhofar Mountains and some arid areas to the north, mainly at low elevation, while *P*. *jensvindumi* is mainly restricted to the Hajar Mountains of north Oman, with many records at high elevation. Nevertheless, both species seem to have similar preferences for the land cover type (bare areas with gravel and rocks). The two subspecies *Uromastyx a*. *leptieni* and *U*. *a*. *microlepis* also have completely different ecological preferences in Oman, the latter inhabiting the Hajar Mountains between sea level up to 1000 m in elevation with preference for bare areas with gravel and rocks, and the former inhabiting mainly lowland (up to 500 m in elevation) hot and dry desert areas of the interior with preference for bare areas with gravel and rocks or sand.

Geckos are the most diverse reptile group in Oman (45% of all the species) and include the genera *Hemidactylus* (15 species); *Pristurus* (10 species); *Asaccus* (six species); *Stenodactylus* (four species); *Ptyodactylus* (three species); *Trachydactylus* (two species) and *Tropiocolotes* (two species). At the generic level, *Hemidactylus*, *Pristurus* and *Trachydactylus* independently cover most of the climatic space of Oman but at the specific level many ecological specializations are found, such as *P*. *gallagheri* or *P*. *minimus*, restricted to high elevation areas of the Hajar Mountains and to the lowland hot and dry desert areas, respectively. The genus *Asaccus* is only found in the rocky and arid Hajar Mountains of north Oman and the genus *Tropiocolotes* is restricted to the tropical Dhofar Mountains and some arid areas of southern Oman. As a result, both groups show very different ecological preferences. The genus *Stenodactylus* is restricted to low elevation (usually below 500 m in elevation) in hot and dry desert areas, with preference for bare areas with gravel and rocks or sand. The three species of the genus *Ptyodactylus* inhabit mountainous areas in both north (Hajar Mountains) and south (Dhofar Mountains). The two species from the north (*P*. *orlovi* and *P*. *ruusaljibalicus*) and the southern species (*P*. *dhofarensis*) occupy a very similar habitat but a rather different climatic space defined by BIO1 and BIO12.

The 13 species of lacertids include three different genera: *Acanthodactylus* (seven species), *Mesalina* (four species) and *Omanosaura* (two species). *Acanthodactylus* and *Mesalina* are mainly restricted to elevations normally below 1000 m in hot and dry desert areas, while the two species of *Omanosaura* are restricted to the Hajar Mountains, with some populations reaching up to 2800 m in elevation in environments with relatively high precipitation and low temperature.

The skinks inhabit the entire ecological space analyzed in this study and, with the exception of the generalist *Trachylepis tessellata* (see above), the other species are restricted to particular areas with different ecological preferences.

*Varanus griseus* is the largest lizard of Oman (more than 1.2 m including the tail) and exploits a very particular habitat characterized by low elevation (up to 600 m), hot and dry bare and rocky areas with sparse vegetation. A similar habitat is exploited by *Diplometopon zarudnyi*, the only amphisbaenid of Oman.

The 21 species of snakes compose the most ecologically heterogeneous group. Three of them are generalists (see above); others such as *Cerastes gasperettii gasperettii* only dwell at elevations below 500 m, in hot and dry sandy areas, and others such as *Pseudocerastes persicus* are only found in the highest parts of the Hajar Mountains (between 500 and 2500 m in elevation), under relatively cold and humid conditions and associated with rocky environments.

Finally, of the 101 species there is evidence for the introduction of four species, three originally from India: *Calotes versicolor*, *Hemidactylus flaviviridis*, and *H*. *leschenaultii*, and a fourth one, the snake *Indotyphlops braminus*, of unknown origin. This species is the most widespread snake species in the world and is parthenogenetic.

## Discussion

### Reducing the linnean shortfall

In the present work, we have analyzed a database including 5,359 observations of all 101 Oman terrestrial reptiles (all reptiles excluding marine turtles and marine snakes) classified using a nearly complete taxonomy resulting from more than 10 years of exploration and systematic work using morphological and molecular data analyzed with multivariate, phylogenetic and population genetic methods, among others [18, 20–30]. These newer methodologies and analytical techniques have resulted in an increase of 17.8% in the species richness of the country compared to previous taxonomic and biogeographic research by Gardner (2013) [10] and the more than 20 years of thorough morphological revisions by E. N. Arnold and collaborators [37–39, 51–56]. The use of DNA has revolutionized systematic studies and has greatly contributed to the discovery of considerable levels of undescribed diversity [18, 20, 22, 27], including several remarkable examples of cryptic diversity [21, 28–30]. This and other molecular techniques are becoming standard methods for species recognition and the use of DNA Barcoding is, in some cases, the only useful identification method [21, 28, 57].

The importance of taxonomy and its impact on conservation efforts was identified long ago [58]. Since then, numerous papers took up the idea that ‘‘bad” taxonomy can hinder conservation [59–64]. Although some authors found no evidence of a consistent effect of taxonomic change in conservation [65], most of them agreed that splitting taxa may allow a better protection. Several examples of Oman taxa that have been split in recent years illustrate that point. One such example includes the geckos of the “*Hemidactylus homoeolepis*” species complex. Before 2012, *H*. *homoeolepis* was thought to be abundant and widespread across the southern Arabian Peninsula (southwest Saudi Arabia, Yemen, Oman), including the islands of Masirah and Hallaniyyat (Oman), and the Socotra Archipelago (Yemen). Consequently, the conservation status of *H*. *homoeolepis* in the IUCN Red List of Threatened Species is of Low Concern (LC). Following the taxonomic revisions by Carranza and Arnold (2012) [18] and Vasconcelos and Carranza (2014) [22], *H*. *homoeolepis* was split into seven different species, including two island endemics and several species with extremely restricted distribution ranges: *H*. *homoeolepis* (endemic to the Socotra Archipelago); *H*. *minutus* (distributed along the Arabian Sea coast, from northeastern Oman to extreme eastern Yemen); *H*. *masirahensis* (endemic to Masirah Island; Oman); *H*. *inexpectatus* (restricted to the coastal area of the Al Wusta governorate, Oman); *H*. *paucituberculatus* (restricted to the lush south-facing sea side of extreme eastern Yemen and the Dhofar Mountains of Oman and the Hallaniyyat Islands); *Hemidactylus* sp. 12 (only known from a single locality in Shaqra, Yemen. Voucher code: BMNH1953.1.6.99; The Natural History Museum, London, UK); and *Hemidactylus* sp. 13 (only known from a single locality in Khiyat, Saudi Arabia. Voucher code: BMNH1992.168; The Natural History Museum, London, UK).

More recently, the gecko species *Asaccus caudivolvulus*, known from the northern Hajar Mountain range of Oman and the UAE, was split into three microendemic species [27]: *A*. *gardneri*, *A*. *margaritae*, and *A*. *caudivolvulus* (the only endemic vertebrate of the UAE, restricted to a small coastal area and at risk from heavy development). Another example involves the two species of the gecko genus *Trachydactylus*: *T*. *hajarensis* and *T*. *spatalurus*, formerly recognized as the same species within the widespread Middle East genus *Bunopus* and now considered two different species of an endemic Arabian genus restricted to the southern Arabian Peninsula [25].

Despite the importance of all the taxonomic changes discussed above, none of them appear in the IUCN Red List of Threatened Species, as there are 32 species of Oman terrestrial reptiles that have not been evaluated (NE) (S6 Fig). Of these, 21 (nine endemic) correspond to described species and nine (five endemic) are species still to be described. Moreover, of the 101 species of Oman reptiles included in this study, 26 (25.75%) had been evaluated by Cox *et al*. (2012) [19] but are still pending of final approval from IUCN and publication on the web page. Of the 92 described species, 48 (52.2%) are still pending of definitive evaluation by the IUCN. This lack of knowledge on the conservation status of the Oman terrestrial reptiles is of similar proportion to the approximately 60%, 63% and 44.4% of non-assessed world’s reptiles, world’s lizards and amphisbaenians and Sahara-Sahel reptiles, respectively [14, 15, 48]. Even though reptiles are very important from an evolutionary, biogeographic, ecologic and conservation point of view, knowledge on their conservation status lags behind that of birds, mammals and amphibians [14, 15, 48].

As ectotherms with relatively small body size and of low vagility, reptiles are greatly affected by the thermal landscapes of their habitat; something that often results in high levels of endemicity [6]. In Oman, 20 (19.80%) reptile species are endemic, while there is not a single species of endemic amphibian, bird or mammal, although the number of birds (527) and mammals (62) is relatively high [10, 66, 67]. This highlights the possible role of reptiles as surrogates for conservation studies in Oman and other arid countries, especially for defining priority conservation areas and to evaluate the coverage of the current network of protected areas [12, 17, 48]

### Reducing the wallacean shortfall

The sampling effort of the present work is not only remarkable from a taxonomic point of view, with multiple observations for most species, but also on the spatial coverage achieved. As explained in the results, the 5,359 observations cover 38.72% of the total area of Oman (divided by grids of 10 arc-minutes; see Fig 4). They are distributed almost continuously across the two-dimensional climatic space of Oman (defined by BIO1 and BIO12; see Fig 5) and are well distributed across the climatic space defined by the PCA inferred using 12 bioclimatic variables (Fig 6). They are also well represented within 17 out of the 20 clusters grouping 10% of the explained climatic variance by PC1 and PC2 (Fig 6; Table 1). The three clusters that could not be sampled: clusters 4 (2.52 km^2^), 2 (15.96 km^2^) and 9 (10.08 km^2^), are situated in the Western Hajars, the Jebel Akhdar and the Eastern Hajars, respectively (Fig 7A). Despite being the best explored area in Oman (most of the 10 arc-minutes grids include observations; Fig 4), future expeditions should target the Hajar Mountains and, more specifically, these three unsampled clusters that represent different climatic areas. A strategy that worked well in previous expeditions, in which climatically diverse areas defined using a similar approach were explored with very positive results [35]. Moreover, exploration should also be directed to reduce the great dissimilarity in the percentage of the clusters’ sampled areas that now range between 0.16% and 66.67%. According to Fig 4, some areas of the interior of the country, on the edge of the Rub Al Khali Desert and in the Jiddat Al Harasis, should also be explored to reduce the proportion of sampled and unsampled grid squares.

There is a positive bias in the number of observations towards the geckos, which include 64.11% of the observations despite representing only 44.55% of the total reptile species and a negative bias in snakes, with just 11.92% of the observations despite representing 20.79% of the total reptile species (S4 Fig). This bias can be explained by the taxonomic interest in geckos as a result of their high levels of undescribed diversity, cryptic diversity and endemicity [18, 21–23, 27–30]. On the other hand, the snakes of Oman are much larger than geckos, more mobile and less prone to diversification processes that may result in patterns of endemicity or microendemicity [9, 10, 68–70]. This hypothesis is supported by the fact that there is not a single endemic snake species in Oman (S2 Appendix). Moreover, as predators of vertebrates, snakes are usually more secretive, less abundant and are therefore much more difficult to observe in the wild [68, 71].

### Explaining the patterns of species and endemicity richness

Most of the grid squares with the highest values of species richness are situated in the Hajar and the Dhofar Mountains (see Figs 8B and 9B). The Hajar Mountains of Oman and the UAE are one of the most biodiversity rich regions in Arabia [18, 25, 27, 29, 30] and, including both countries, have 19 described endemic species of reptiles and four endemic species in the process of being described. Of all the species endemic to the Hajar Mountains, seven described and three undescribed are endemic to Oman (are not found in the UAE) and are the ones used to calculate endemicity richness in the present work.

The highest levels of species richness within the Omani part of the Hajar Mountains concentrate in the Jebel Akhdar and some areas further east and in the Musandam Peninsula. Interestingly, some areas of high species richness are around the capital, Muscat, and most probably result from the combination of two factors: 1) it is a diverse and ecologically rich area, including well preserved beaches, plains, wadis, mountains and wetlands, and 2) as a result of its proximity to the capital, it has been surveyed very thoroughly. These results are also an indication that Oman reptiles have not been greatly affected by human presence, with several species finding suitable environments in and around human settlements and human modified ecosystems [10, 21].

In the south, the highest diversity is concentrated in the Dhofar Mountains, where the climatic differences between the lush south-facing (sea) side and the dry north-facing (land) side of the mountains have played an important role in shaping the flora and fauna of this interesting biodiversity rich region [38, 39, 72–79].

The pattern of endemic species richness in Oman changes dramatically and is mainly concentrated in the Jebel Akhdar, with some areas with less endemic species richness in the Eastern Hajars and Masirah Island. Interestingly, Dhofar has very low levels of endemicity. This pattern does not match with the map of species richness (much higher in Dhofar than in the north; Fig 8) and can be explained by the fact that some species in the Omani side of the Dhofar Mountains extend their range to the Yemeni side of the Dhofar Mountains and other species have disjoint distributions in Dhofar and the Western Mountains of Yemen and Saudi Arabia [8, 9] (similar to the situation in the Hajar Mountains, where many species also occur in the UAE and are therefore not considered endemic to Oman; see above). All these species found in the Dhofar Mountains of Oman but that also occur elsewhere in Yemen and Saudi Arabia (and are therefore not endemic) include: *Acanthocercus adramitanus*, *Hemidactylus festivus*, *H*. *lemurinus*, *H*. *alkiyumii*, *H*. *paucituberculatus*, *H*. *minutus*, *Ptyodactylus dhofarensis*, *Pseudotrapelus dhofarensis*, *Tropiocolotes scortecci*, *Trachydactylus spatalurus*, *Mesalina ayunensis*, *Uromastyx benti*, *Chamaeleo arabicus*, *Acanthodactylus felicis*, *Mesalina* sp.1, *Platyceps thomasi*, *Rhynchocalamus arabicus*, *Atractaspis andersonii*, *Bitis arietans*, *Echis coloratus*, *E*. *khosatzkii* and *Naja arabica*.

The Dhofar and Western Mountains of Yemen and Saudi Arabia are partially or totally affected by the moisture laden southwest monsoon winds that blow against the sea-facing cliffs between July and August and that are responsible for the unique green vegetation on the coastal side of these mountain ranges, creating a similar ecosystem of tropical forest with a patchy distribution across more than 2,000 km that has facilitated the dispersal of tropical taxa [10, 18, 21, 23–26, 33, 38].

### Evaluating the effectiveness of the protected areas

Despite the importance of Oman’s terrestrial reptiles in terms of diversity and endemicity (see above and S2 Appendix), the 22 protected areas of Oman have been established aimed mostly to conserve threatened large mammals, such as the leopard, several species of gazelles and the Arabian Oryx, endemic flora and mountain sceneries, marine habitats, khawrs and wetlands, and breeding marine turtles. Although the 22 protected areas are distributed across the country (Fig 3), there are large parts of the climatic space of Oman outside protected areas and seven clusters (different environmental states) are not represented by the protected areas at all (Fig 10). All seven clusters are in the Hajar Mountains (Fig 7A).

The coverage of the protected areas is relatively small, totaling 3.91% of the country. Nevertheless, they include within their limits 64 terrestrial reptiles (63.37%), 10 endemic species (50%) and three threatened species (60%). This level of protection of the reptile fauna could be improved by covering the areas of maximum species richness and maximum endemic species richness (Figs 8 and 9), situated in some areas of the Hajar Mountains in the north and the Dhofar Mountains in the south. There are eight protected areas in the Hajar Mountains (Fig 3; Table 2): Jebel Akhdar, Al Khawari, Al Qurum, Al Sareen, Ras Al Shajer, Al Saleel, Jebel Qahwan and the Turtle reserve, all situated in the central and eastern part of the Hajar Mountains but there is not a single protected area situated in the Western Hajars and especially in the Musandam governorate, a unique area from a physiogeographical and ecological point of view that includes some microendemic species such as *Asaccus gardneri*, *A*. *margaritae* and *Ptyodactylus ruusaljibalicus* ([26, 28]; S2 Appendix). Moreover, clusters 4 (2.58 km^2^) and 14 (367.83 km^2^) are only present in the Musandam Peninsula and represent different environmental states that may be worth protecting.

The largest protected area is located in Dhofar, covering the easternmost part of the Dhofar Mountains (Jebel Samhan; 1.53% of all protected areas; S1 Table and Figs 2 and 3). Nevertheless, this large protected area clearly fails to protect the areas with the highest species richness and endemic species richness in Dhofar, situated more to the west in the Jebel Qamar, Jebel Qara and the Salalah Plain (Figs 8B and 9B). Another relevant area in Oman outside protected areas is Masirah Island, an island that includes 19 species, two Oman endemics and one species endemic to the island (*Hemidactylus masirahensis*).

At an individual level, the percentage of the species’ distribution area included within a protected area indicates that most of the species (endemic and non-endemic) are below the conservation target of 17% or even the less restrictive 12% of their total area within a protected area in order to be considered “adequately protected”. In fact, being very conservative and taking into account a combination of both approaches used in the gap analysis (see material and methods), 69 (68.31%) of all the species, including 13 (65%) of the endemic species, have less than 12% of their distribution area protected. More importantly, even considering the most favorable of the two approaches used in the gap analysis (EOO approach in this case), there are still 20 species (19.8%) including five endemic (25%) with their full distribution outside the protected areas. This low level of protection contrasts with the situation in the arid archipelago of Cape Verde, where most of the reptile species were well above the targeted 12% [12].

Our results suggest that the current protected areas of Oman may not provide adequate refuge for reptiles. Therefore, an evaluation of the coverage of the current protected areas and the identification of priority conservation areas using better descriptions of distributions (e.g. ecological modelling) and reserve design algorithms are urgently needed.

## Supporting information

S1 Appendix Script for PCA based clustering. The clusters are generated per component, grouping the samples within the user defined percentage of variation. The final classification is given for the number of components chosen. The script has a function to generate clusters based on a PCA in R and an example of use.
(PDF)

S2 Appendix Atlas of the terrestrial reptiles of Oman. An appendix showing photographs, distribution and ecology of all the 101 species of terrestrial reptiles of Oman included in the present study; number of observations; species lists by governorate; species lists by protected area; all the species’ distribution maps defined using minimum convex polygon (MCP) of the observations filtered by the species’ average altitude, and a list of all the authors of the photographs (all of them authors of the manuscript).
(PDF)

S3 Appendix Spatial data of the terrestrial reptiles of Oman. Appendix including the spatial data in shapefile format with species listed in the attribute table. Species scientific names were abbreviated and the correspondence to full names is given in the accompanying text file.
(ZIP)

S1 Fig Topoclimatic characterization of Oman. (A) Map of the annual mean temperatures in °C (BIO1); (B) Map of the annual precipitation in mm (BIO12); (C) Map of land cover types of the year 2008; (D) Graph of the elevation frequency dividing Oman into intervals of 100 m. Details in the Material and Methods section.
(TIFF)

S2 Fig Political map of Oman. The map shows the limits of the 11 governorates. Inlet: Location of Oman (red) in the world map.
(TIFF)

S3 Fig Population of Oman by governorate. (A) Table indicating the area and population of each governorate; (B) The same information presented visually on a map.
(TIF)

S4 Fig Main taxonomic groups observed. Percentage of the total number of (A) species and (B) observations in the seven main taxonomic groups of Oman reptiles in the database (between parenthesis, the respective number of observation).
(TIFF)

S5 Fig Maps of venomous species richness. **(**A) Venomous species richness by governorate; (B) venomous species richness by grids of 10 arc-minutes of latitude and longitude.
(TIFF)

S6 Fig Percentage of the number of species of Oman reptiles by IUCN conservation category. LC* indicates species that have been evaluated by Cox *et al*. (2012) [19] but are still pending of final approval from IUCN and publication on the web of the IUCN Red List of Threatened species (http://www.iucnredlist.org/)).
(TIFF)

S7 Fig Percentage of number of species in each IUCN conservation category by governorate. “LC not available on the web” indicates species that have been evaluated by Cox et al. (2012) [19] but are still pending of final approval from IUCN and publication on the web of the IUCN Red List of Threatened species (http://www.iucnredlist.org/)).
(TIFF)

S1 Table Information on the 101 species of Oman terrestrial reptiles. Number and percentage of the total number of species, endemic species, venomous species, threatened species, species not evaluated (NE) by the IUCN Red List of Threatened species organized by governorates, protected areas and islands or island groups. The last two columns show the area of each governorate, protected area and island or island group and the percentage of this area with respect to the total area of Oman.
(DOCX)

S2 Table Area of Occupancy at 4 km^2^ of all 101 terrestrial reptiles of Oman. Number of occupied cells of 4 km^2^ and Area of Occupancy (AOO) calculated for each species according to the IUCN Red List Guidelines 2016.
(DOCX)

S3 Table Loadings, eigenvalues, and variance explained by the two first components retained from the Principal Component Analysis (PCA) performed on the 12 bioclimatic variables used in this study. The two variables with the highest loadings values in both PC1 and PC2 are highlighted in bold.
(DOCX)

S4 Table Information for the gap analysis with distribution records at a resolution of 1 km^2^. List of all 101 reptile species of Oman, showing if they are endemic or not, total area of extent of occurrence (defined by the presence-absence in every pixel of 1 km x 1 km), area of extent of occurrence inside protected areas and percentage of the area of extent of occurrence inside protected areas.
(DOCX)

S5 Table Information for the gap analysis with distribution records using the minimum convex polygon (MCP) filtered by the species’ average altitude. List of all 101 reptile species of Oman, showing if they are endemic or not, total area of extent occurrence (defined by the MCP), area of extent of occurrence inside protected areas and the percentage of the area of extent of occurrence inside protected areas.
(DOCX)
