# Supplementary material for: Diversity, distribution and conservation of the terrestrial reptiles of Oman (Sauropsida, Squamata)
Source: PLoS One. 2018 Feb 7;13(2):e0190389. doi: 10.1371/journal.pone.0190389 (PMC5802441; doi:10.1371/journal.pone.0190389)
Supplement: S1 Appendix — The clusters are generated per component, grouping the samples within the user defined percentage of variation. The final classification is given for the number of components chosen. The script has a function to generate clusters based on a PCA in R and an example of use. (PDF) [file pone.0190389.s001.pdf]

```r
# PCA clusters based on a percentage of the variance explained in each
# component. The algorithm calculates the variance explained in each component
# and then calculates the number of possible divisions that hold the amount of
# variance defined. Example: component when explains 70% of the total varaince,
# and the user defines 10% of explained variance for each cluster/component,
# then the component has 7 clusters.
#
# Authors: Pedro Tarroso, Meritxell Xipell
# Last revision: 14/07/2017
# citation: Carranza S, Xipell M, Tarroso P, et al (2017) Distribution,
#           diversity and conservation of the terrestrial reptiles of Oman
#           (Sauropsida, Squamata). Submitted.



cluster.pca <- function(pca, percent, comp = 1:2) {
    ### Function to compute pca clusters based on total variance
    ### pca - a pca object from 'princomp' or 'prcomp'
    ### percent - a percentage of explanation for each cluster
    ### comp - number of components to calculate clusters
    ###
    ### Outputs a list with clusters per sample and per component,
    ### a list of centroids (position in each component) for each
    ### cluster, and a list of breaks of each cluster in each component.

    var.expl <- pca$sdev**2 / sum(pca$sdev**2) * 100
    cluster.n <- var.expl / percent
    scores <- predict(pca)
    n <- length(comp)
    clusters <- matrix(NA, nrow(scores), n+1)
    centroids <- breaks <- list()

    for (i in 1:n) {
        size <- diff(range(scores[,comp[i]]))/cluster.n[comp[i]]
        brk <- c(min(scores[,comp[i]]),
                 rev(seq(0, min(scores[,comp[i]]), -size)[-1]),
                 seq(0, max(scores[,comp[i]]), size),
                 max(scores[,comp[i]]))
        breaks[[i]] <- brk
        clusters[,i] <- .bincode(scores[,comp[i]], brk, F, T)
        centroids[[i]] <- brk[1:(length(brk)-1)] + diff(brk)/2
    }

    combined <- as.integer(apply(clusters[,1:n], 1, paste, collapse=""))
    clusters[,n+1] <- as.integer(as.factor(combined))
    colnames(clusters) <- c(paste("comp", comp, "cluster", sep="_"), "combined")

    return(list(clusters=clusters, centroids=centroids, breaks=breaks))
}


#####################
 ## Example usage ##
#####################

pca <- princomp(iris[1:4])
cluster <- cluster.pca(pca, 100)
```

```
# plot the comp.1 and 2 from pca
plot(pca$scores[,1:2], asp=1)

# add to plot the cluster breaks of each component
abline(v=cluster$breaks[[1]], lty=2, col='red')
abline(h=cluster$breaks[[2]], lty=2, col='red')

# Get the centroids of each component cluster
x <- cluster$centroids[[1]][cluster$clusters[,1]]
y <- cluster$centroids[[2]][cluster$clusters[,2]]

# merge and get only as list of unique centroids/clusters
centroids <- data.frame(x, y, cluster$clusters[,3])
centroids <- unique(centroids)

## add the cluster unique ID to plot
text(centroids[,1], centroids[,2], centroids[,3], cex=2)
```
