# Supplementary material for: Diversity, distribution and conservation of the terrestrial reptiles of Oman (Sauropsida, Squamata)
Source: PLoS One. 2018 Feb 7;13(2):e0190389. doi: 10.1371/journal.pone.0190389 (PMC5802441; doi:10.1371/journal.pone.0190389)
Supplement: S2 Appendix — An appendix showing photographs, distribution and ecology of all the 101 species of terrestrial reptiles of Oman included in the present study; number of observations; species lists by governorate; species lists by protected area; all the species’ distribution maps defined using minimum convex polygon (MCP) of the observations filtered by the species’ average altitude, and a list of all the authors of the photographs (all of them authors of the manuscript). (PDF) [file pone.0190389.s002.pdf]

S2 Appendix. Atlas of the terrestrial reptiles of Oman

Appendix showing photographs, distribution and ecology of all the 101 species of terrestrial reptiles of Oman included in the present study [S1–S101]; number of observations [S102]; species lists by governorate [S103–S116]; species lists by protected area [S117–S121]; all the species' distribution maps defined using minimum convex polygon (MCP) of the observations filtered by the species' average altitude [S122–S134], and a list of all the authors of the photographs (all of them authors of the manuscript).

A HIGH-RESOLUTION VERSION OF THIS APPENDIX CAN BE DOWNLOADED FROM THE FOLLOWING WEB ADDRESS

http://purl.org/carranza/data

Index

S1–S101: SPECIES INFORMATION 8
LIZARDS 9
AGAMIDS 9
Agamidae 9
Acanthocercus adramitanus 9
Calotes versicolor 10
Phrynocephalus arabicus 11
Phrynocephalus maculatus 12
Phrynocephalus sakoi 13
Pseudotrapelus dhofarensis 14
Pseudotrapelus jensvindumi 15
Trapelus flavimaculatus 16
Uromastyx aegyptia leptieni 17
Uromastyx aegyptia microlepis 18
Uromastyx benti 19
Uromastyx thomasi 20
Chamaeleonidae 21
Chamaeleo arabicus 21
GECKOS 23
Gekkonidae 23
Bunopus tuberculatus 22
Cyrtopodion scabrum 23
Hemidactylus alkiyumii 24
Hemidactylus endophis 25
Hemidactylus festivus 26
Hemidactylus flaviviridis 27
Hemidactylus hajarensis 28
Hemidactylus inexpectatus 29
Hemidactylus lemurinus 30
Hemidactylus leschenaultii 31
Hemidactylus luqueorum 32
Hemidactylus masirahensis 33
Hemidactylus minutus 34
Hemidactylus paucituberculatus 35
Hemidactylus persicus 36
Hemidactylus robustus 37
Hemidactylus sp. 38
Pseudoceramodactylus khobarensis 39
Stenodactylus arabicus 40
Stenodactylus doriae 41
Stenodactylus leptocosymbotes 42
Stenodactylus sharqiyahensis 43
Trachydactylus hajarensis 44
Trachydactylus spatalurus 45
Tropiocolotes scortecci 46
Tropiocolotes sp. 47
Phyllodactylidae 48
Asaccus gallagheri 48
Asaccus gardneri 49
Asaccus margaritae 50
Asaccus montanus 51
Asaccus platyrhynchus 52
Asaccus arnoldi 53
Ptyodactylus dhofarensis 54
Ptyodactylus orlovi 55
Ptyodactylus ruusaljibalicus 56
Sphaerodactylidae 57
Pristurus carteri 57
Pristurus celerrimus 58
Pristurus gallagheri 59
Pristurus minimus 60
Pristurus rupestris rupestris 61
Pristurus sp. 1 62

Pristurus sp. 2 63
Pristurus sp. 3 64
Pristurus sp. 4 65
Pristurus sp. 5 66
LACERTIDS 67
Lacertidae 67
Acanthodactylus blanfordii 67
Acanthodactylus boskianus 68
Acanthodactylus felicis 69
Acanthodactylus haasi 70
Acanthodactylus masirae 71
Acanthodactylus opheodurus 72
Acanthodactylus schmidti 73
Mesalina adramitana 74
Mesalina ayunensis 75
Mesalina sp. 1 76
Mesalina sp. 2 77
Omanosaura cyanura 78
Omanosaura jayakari 79
SKINKS 81
Scincidae 81
Ablepharus pannonicus 80
Chalcides ocellatus ocellatus 81
Heremites septemtaeniatus 82
Scincus mitranus 83
Scincus scincus conirostris 84
Trachylepis brevicollis 85
Trachylepis tessellata 86
AMPHISBAENIDS 87
Trogonophidae 87
Diplometopon zarudnyi 87
VARANIDS 88
Varanidae 88
Varanus griseus 88
SNAKES 89
Boidae 89
Eryx jayakari 89
Colubridae 91
Lytorhynchus diadema diadema 90
Platyceps rhodorachis rhodorachis 91
Platyceps thomasi 92
Rhynchocalamus arabicus 93
Spalerosophis diadema cliffordii 94
Telescopus dhara dhara 95
Elapidae 96
Naja arabica 96
Lamprophiidae 97
Atractaspis andersonii 97
Psammophis schokari 98
Rhagerhis moilensis 99
Leptotyphlopidae 101
Myriopholis macrorhyncha 100
Myriopholis nursii 101
Typhlopidae 102
Indotyphlops braminus 102
Viperidae 103
Bitis arietans 103
Cerastes gasperettii gasperettii 104
Echis carinatus sochureki 105
Echis coloratus 106
Echis khosatzkii 107
Echis omanensis 108
Pseudocerastes persicus 109

S102: TOTAL NUMBER OF OBSERVATIONS . . . 112
S103-S116: SPECIES LISTS OF OMAN . . . 116
Oman . . . 118
Musandam Governorate . . . 120
Al Buraymi Governorate . . . 121
Al Batinah North Governorate . . . 122
Al Batinah South Governorate . . . 123
Adh Dhahirah Governorate . . . 124
Ad Dakhliyyah Governorate . . . 125
Muscat Governorate . . . 126
Ash Sharqiyyah North Governorate . . . 127
Ash Sharqiyyah South Governorate . . . 128
Al Wusta Governorate . . . 130
Dhofar Governorate . . . 131
S117-S121: SPECIES LISTS BY PROTECTED AREA . . . 134
Protected areas of Oman . . . 135
Dimaniyat Islands, Al Qurum Ramsar Site, Al Khawari, Jebel Akhdar, Al Sareen & Ras Al Shajer . . . 136
Al Saleel, Turtle Reserve & Jebel Qahwan . . . 137
Al Wusta Wetland Reserve, Al Wusta Wildlife Sanctuary & Jabal Samhan . . . 138
Khawr Rawri, Khawr Taqah, Khawr Sawli, Khawr Dahareez, Khawr Baleed, Khawr Salalah, Khawr Awqad, Khawr Qurum Al Kabeer, Khawr Qurum Al Sagher & Khawr Mughsayi . . . 139
S122-S134: MAPS OF SPECIES' DISTRIBUTION DEFINED USING MINIMUM CONVEX POLYGONS . . . 142
S135: AUTHORS OF THE PHOTOGRAPHS . . . 158

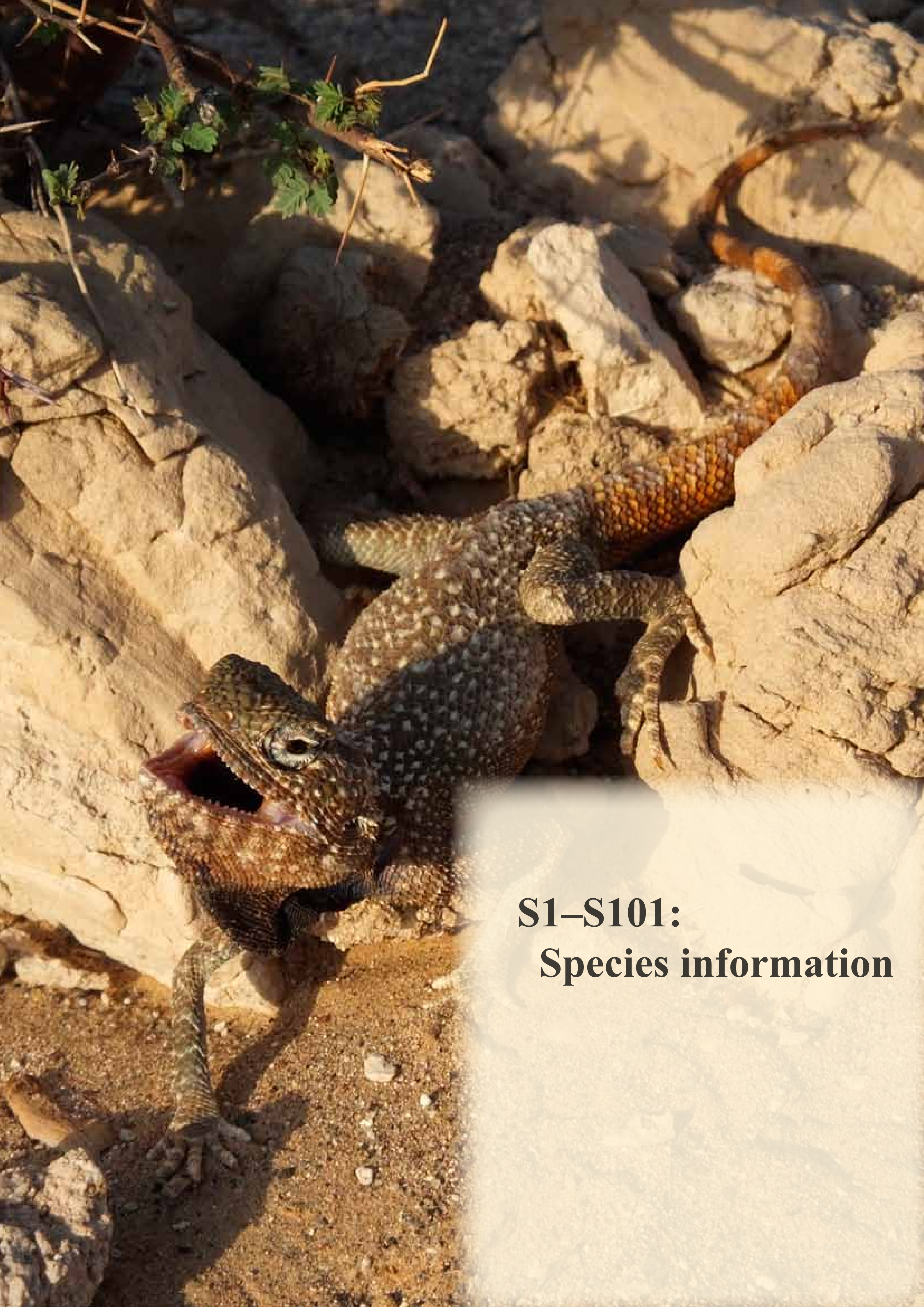

S1–S101: Species information

S1–S101: Species information

There is a separate plate for each one of the 101 species. Each plate contains the species name and authority (except undescribed species) and the following items:

A) picture of the species

B) distribution map with observations (red dots) at a scale of 10 arc-minutes (~18 km)

C) table with some useful information

D) graph of the elevation frequency at 100 m intervals

E) two-dimensional climatic space of Oman (grey dots) defined by total annual precipitation (BIO12) and mean annual temperature (BIO1) with red dots representing the species distribution

F) Boxplot of Land Cover distances (see Fig. 3C for the Land Cover map). TO (Tree Open); C (Cropland); COVM (Cropland / Other vegetation mosaic); S (Shrub); H (Herbaceous); SV (Sparse Vegetation); BAGR (Bare Areas Gravel Rock); BAS (Bare Areas Sand); U (Urban). The top of the rectangle indicates the third quartile, a horizontal line near the middle of the rectangle indicates the median, and the bottom of the rectangle indicates the first quartile. The vertical line extending from the top of the rectangle indicates the maximum value, and another vertical line extending from the bottom of the rectangle indicates the minimum value. Empty circles represent outliers.

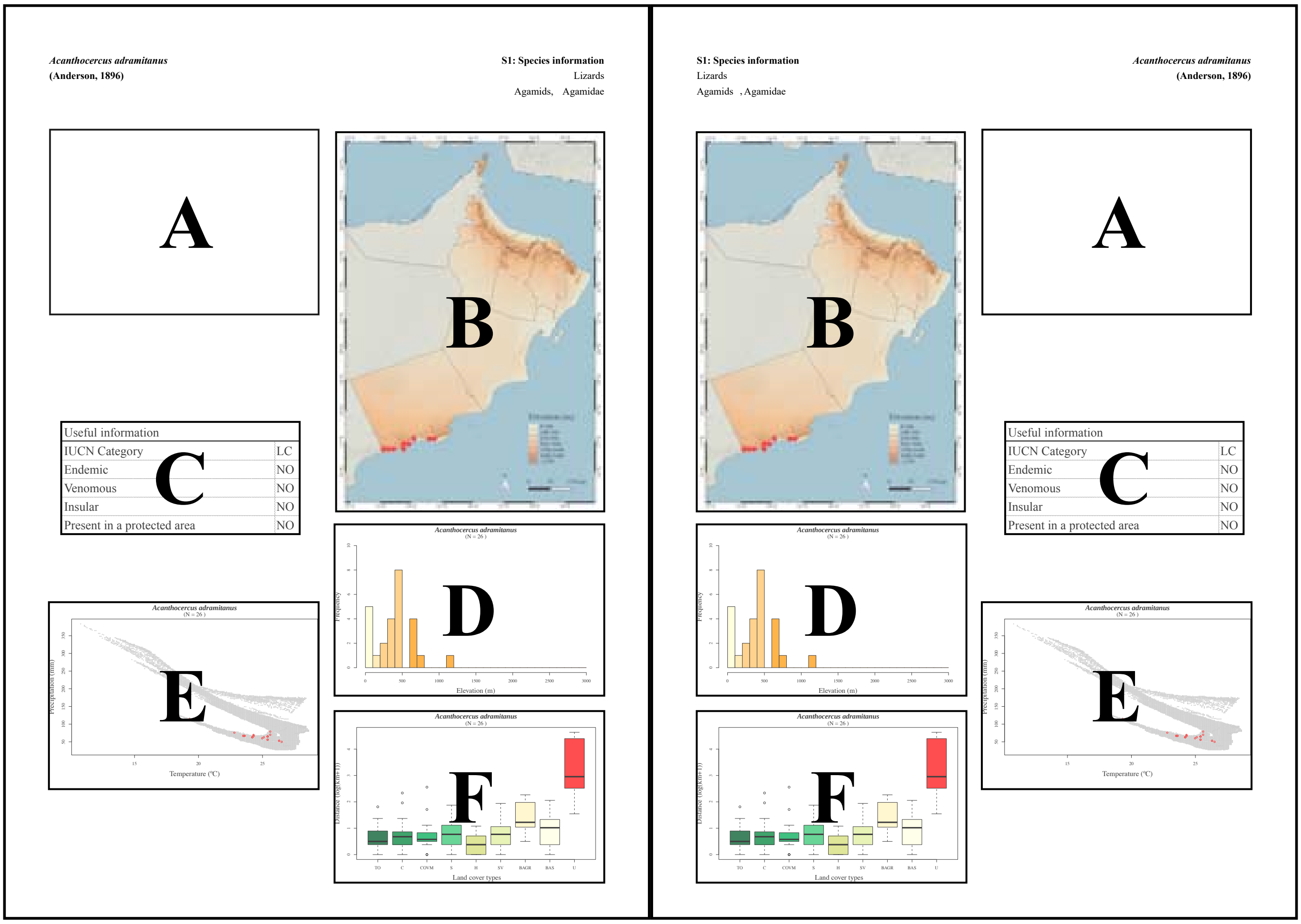

S1: Species information
Lizards
Agamids, Agamidae

Acanthocercus adramitanus
(Anderson, 1896)

A

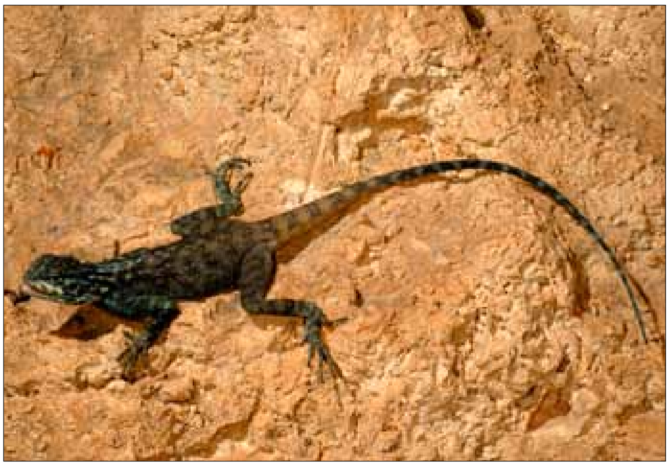

B

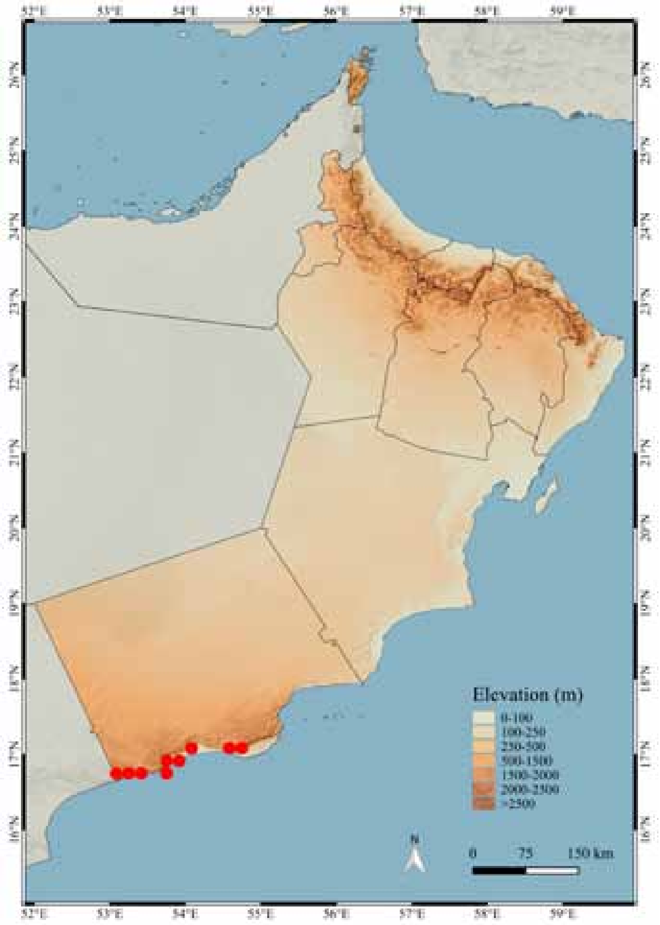

C

Useful information	
IUCN Category	LC
Endemic	NO
Venomous	NO
Insular	NO
Present in a protected area	NO

D

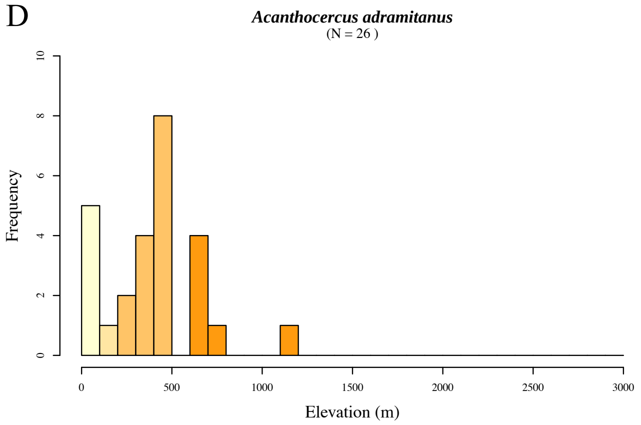

E

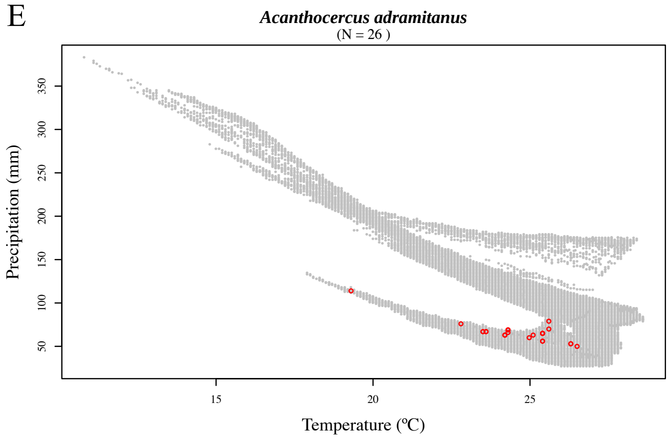

F

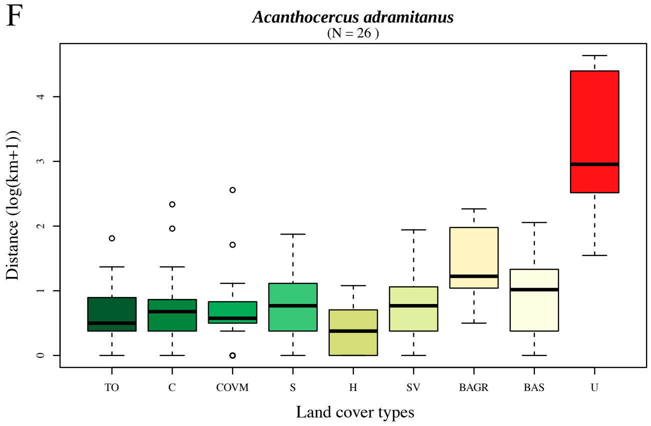

Calotes versicolor
(Daudin, 1802)

S2: Species information
Lizards
Agamids, Agamidae

A

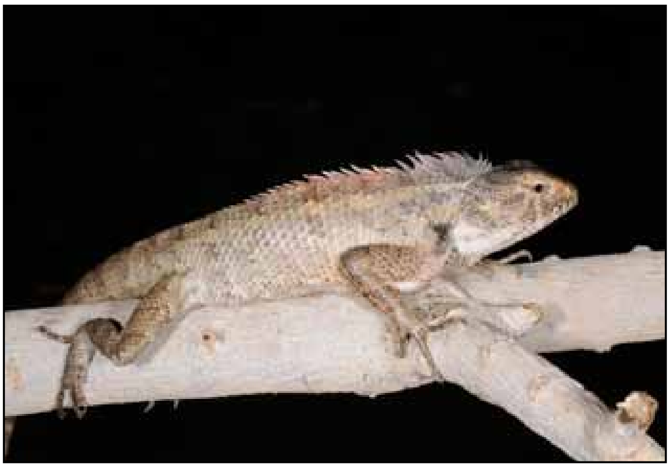

B

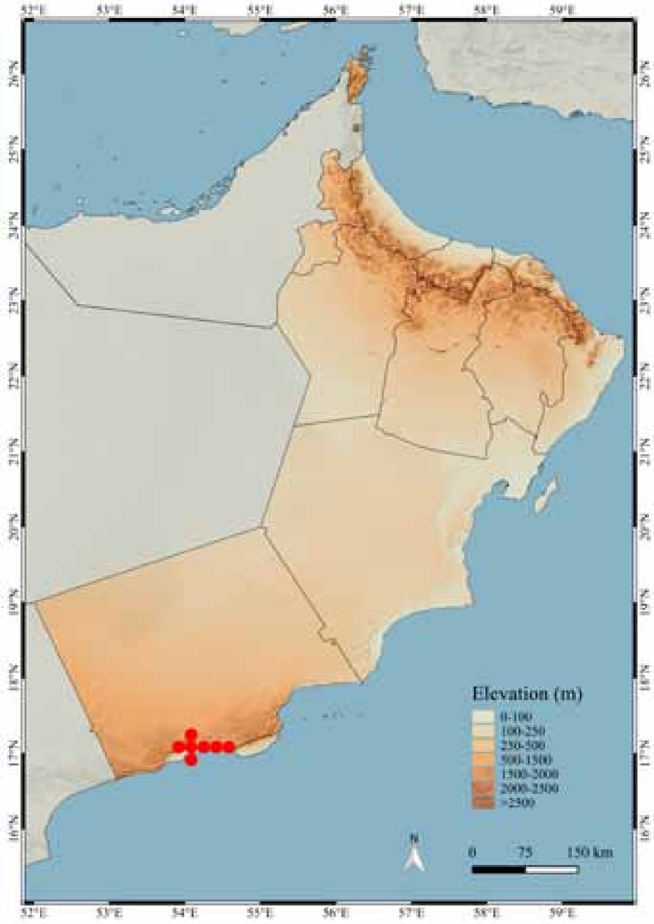

C

Useful information	
IUCN Category	LC*
Endemic	NO
Venomous	NO
Insular	NO
Present in a protected area	NO

*Not available on the web

D

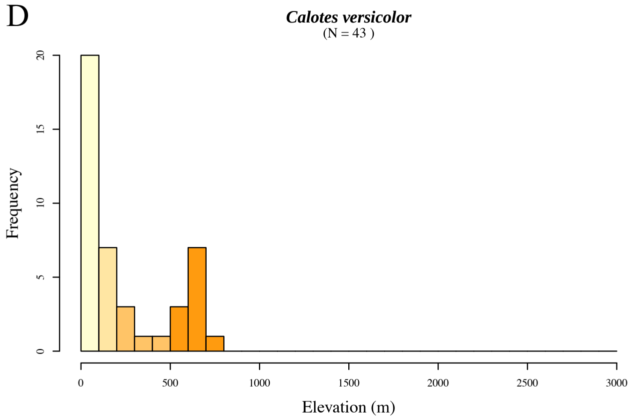

E

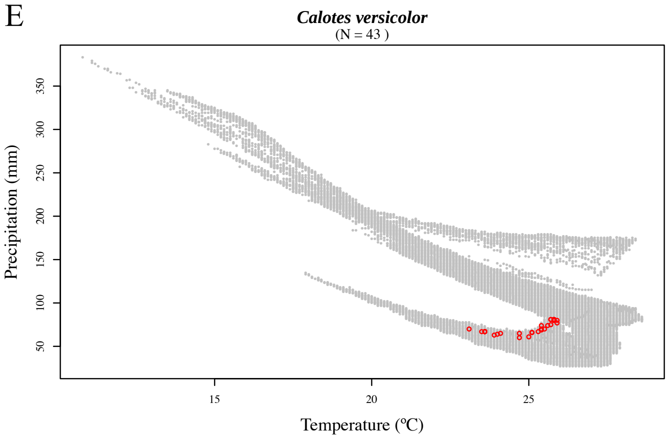

F

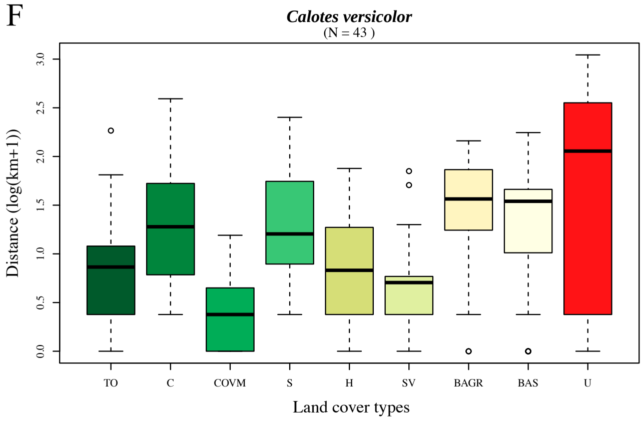

S3: Species information

Lizards

Agamids, Agamidae

Phrynocephalus arabicus
(Anderson, 1894)

B

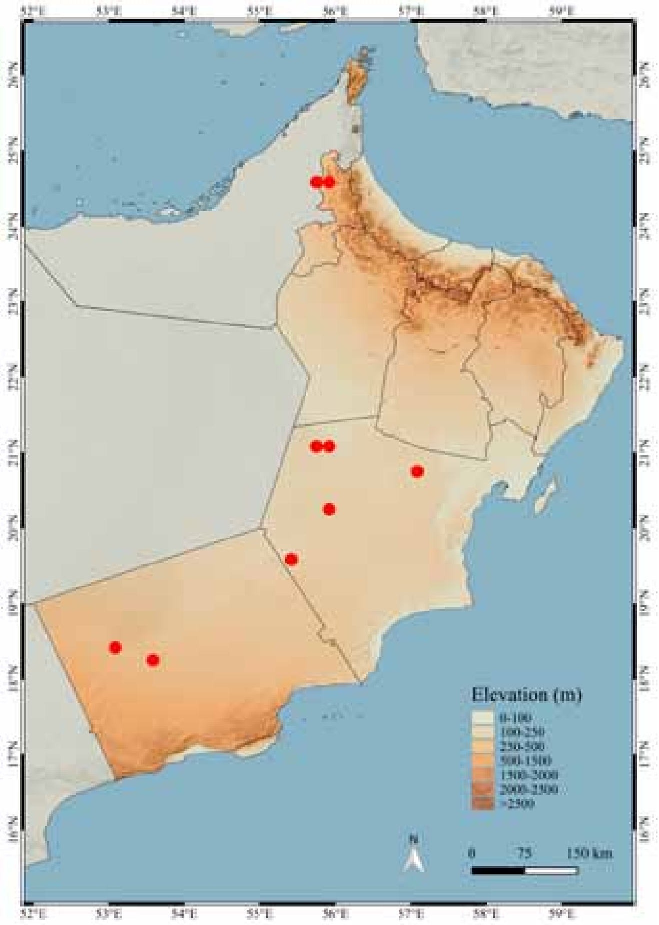

A

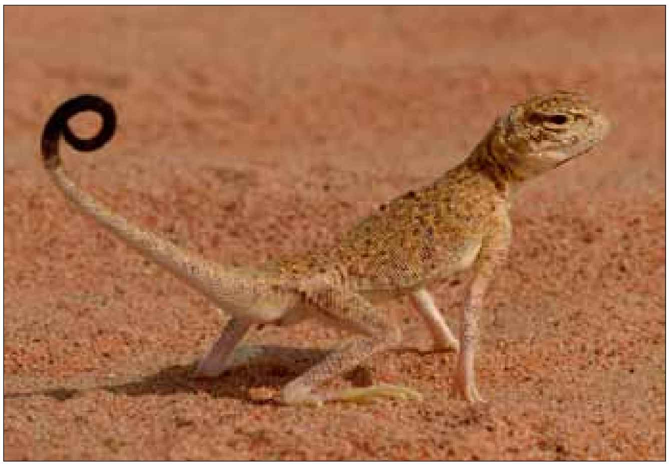

C

Useful information	
IUCN Category	LC
Endemic	NO
Venomous	NO
Insular	NO
Present in a protected area	NO

D

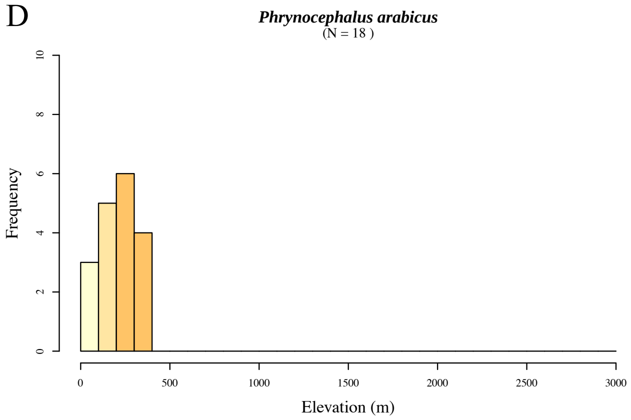

E

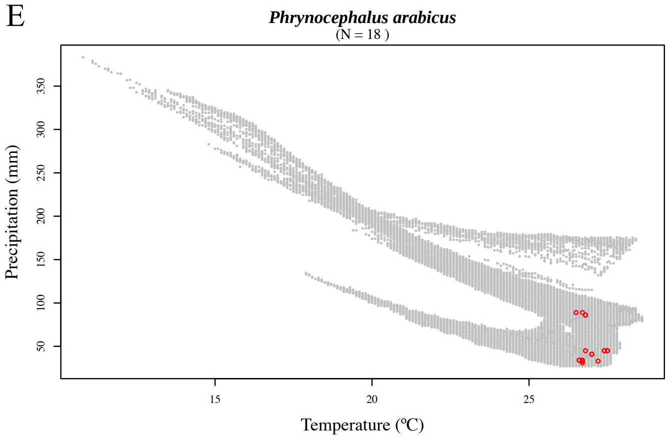

F

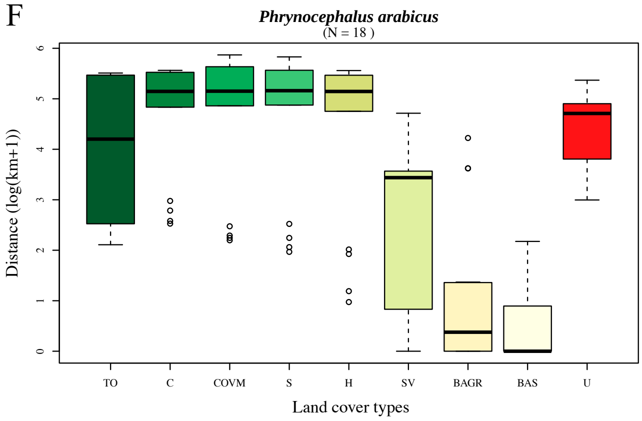

Phrynocephalus maculatus
Anderson, 1872

S4: Species information
Lizards
Agamids, Agamidae

A

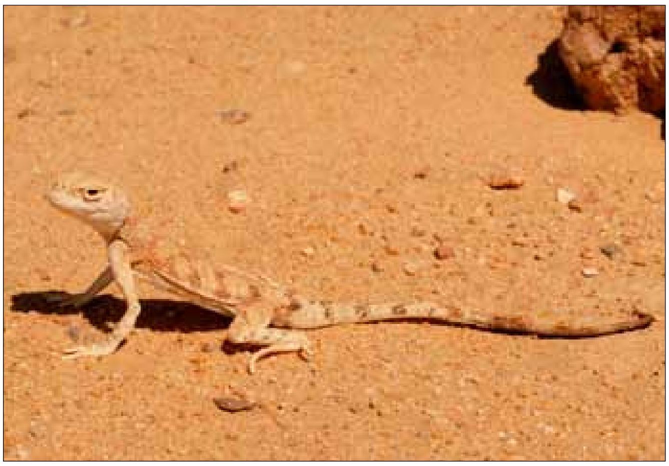

B

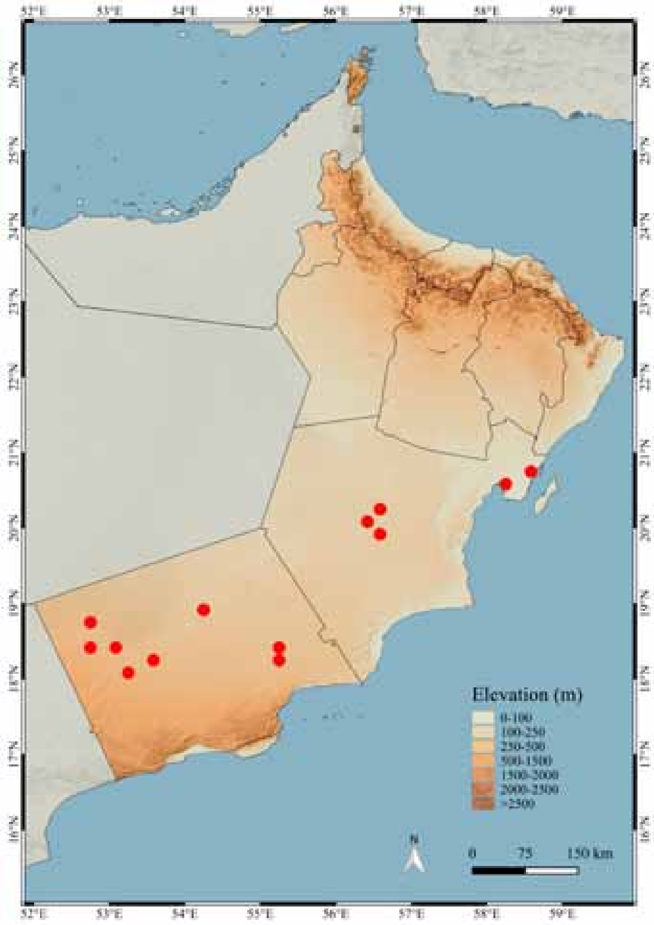

C

Useful information	
IUCN Category	LC*
Endemic	NO
Venomous	NO
Insular	NO
Present in a protected area	YES

*Not available on the web

D

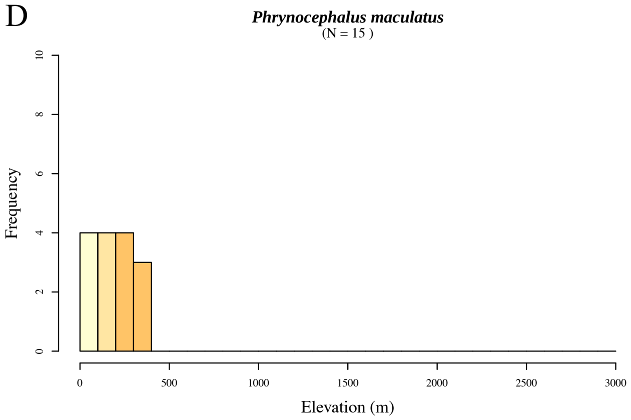

E

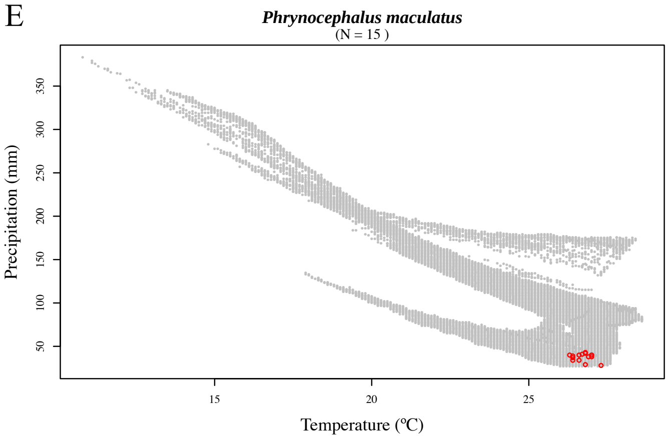

F

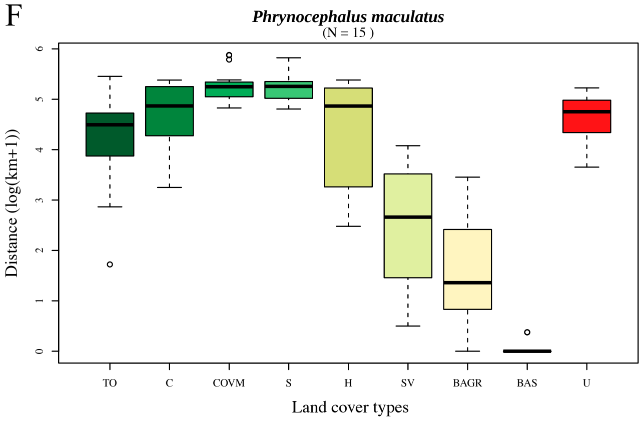

S5: Species information

Lizards

Agamids, Agamidae

Phrynocephalus sakoi

Melnikov, Melnikova, Nazarov, Al-Johany & Ananjeva, 2015

B

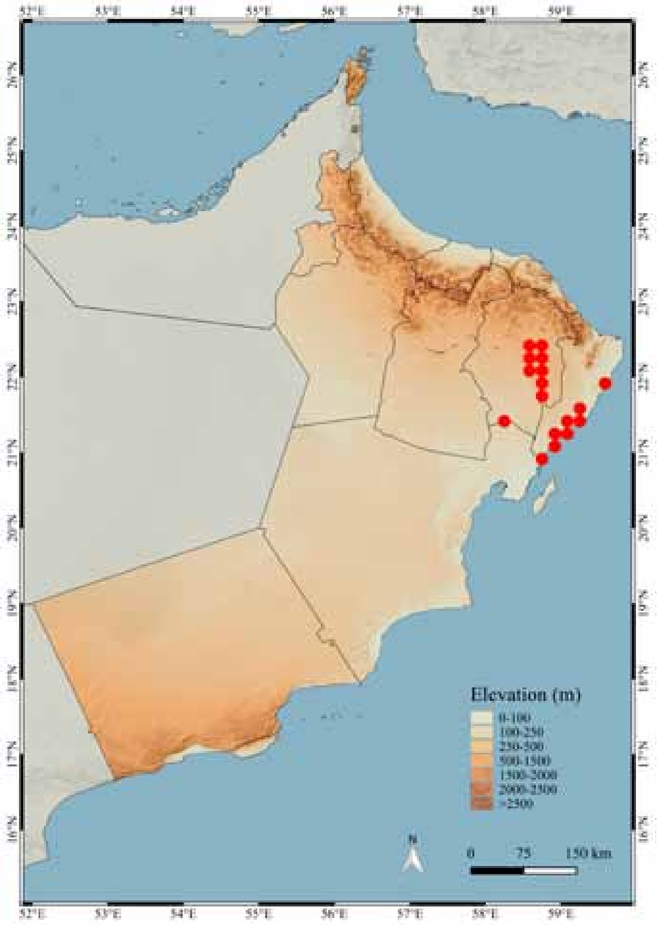

A

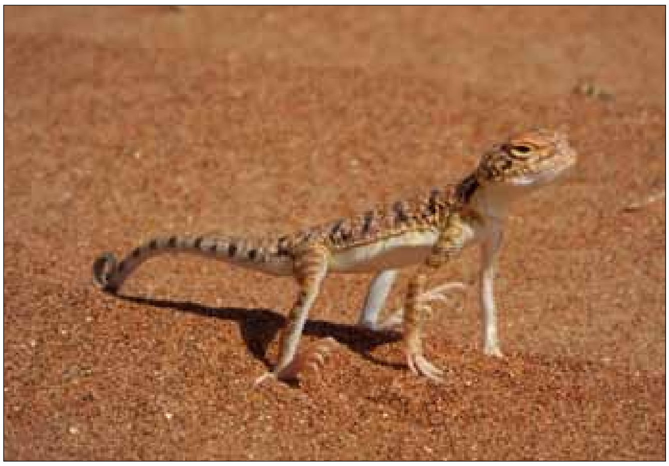

C

Useful information

IUCN Category	NE
Endemic	YES
Venomous	NO
Insular	NO
Present in a protected area	YES

D

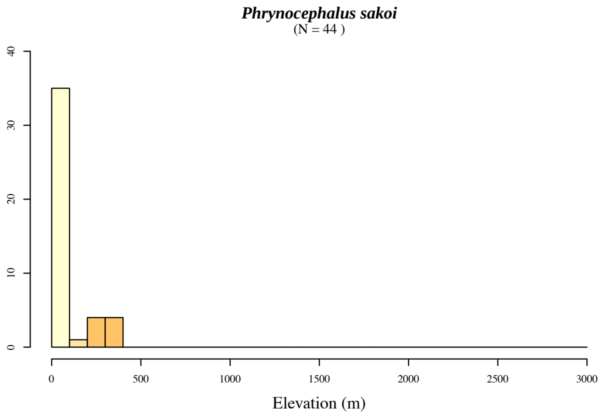

E

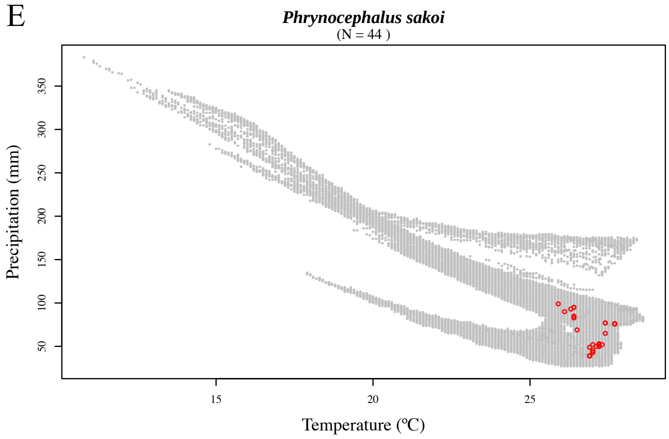

F

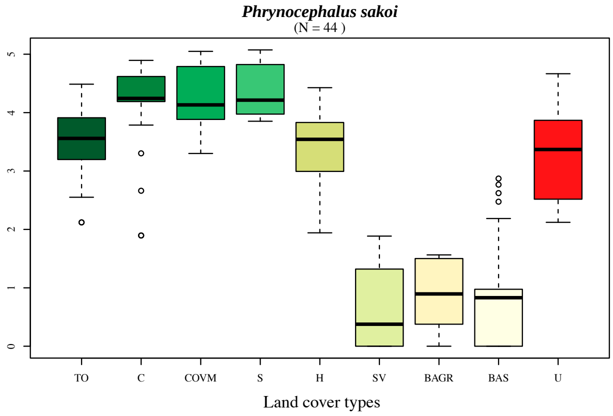

Pseudotrapelus dhofarensis
Melnikov & Pierson, 2012

S6: Species information
Lizards
Agamids, Agamidae

A

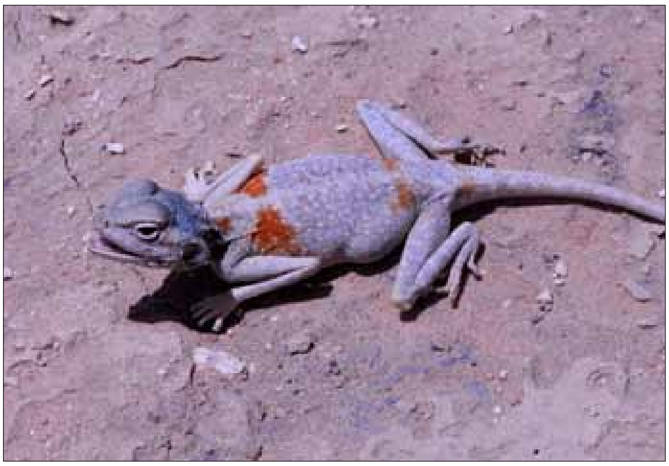

B

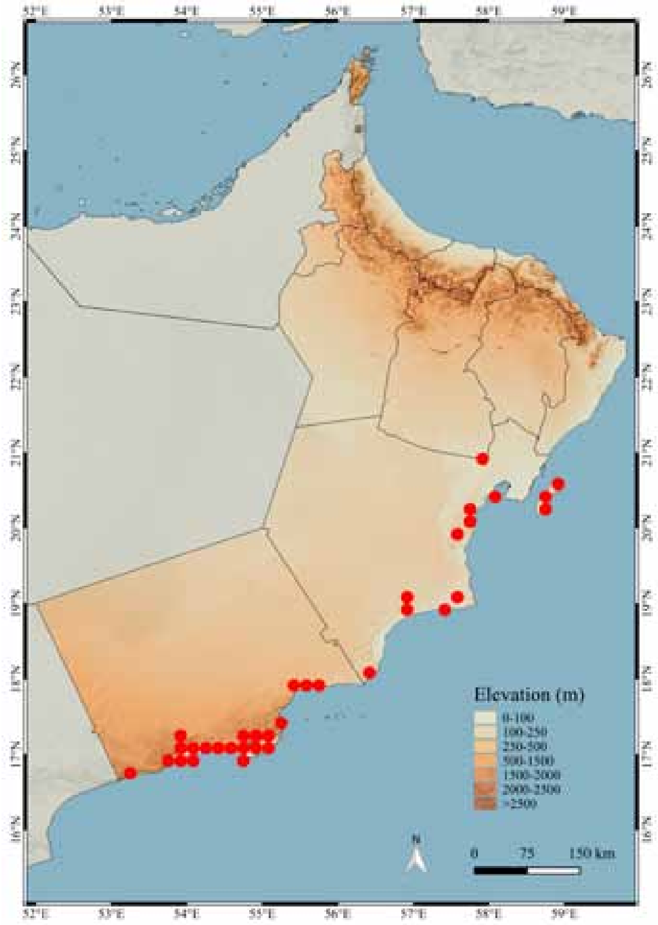

C

Useful information	
IUCN Category	NE
Endemic	NO
Venomous	NO
Insular	YES
Present in a protected area	YES

D

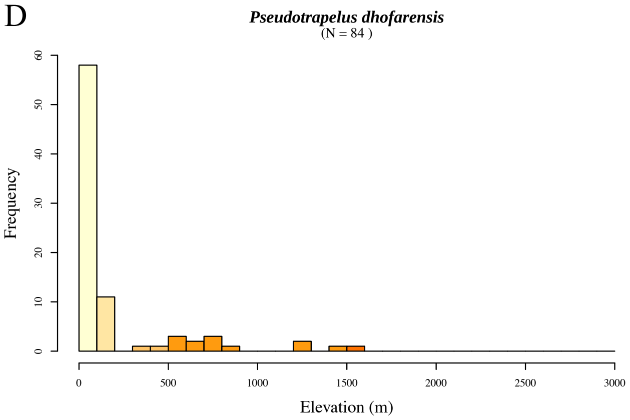

E

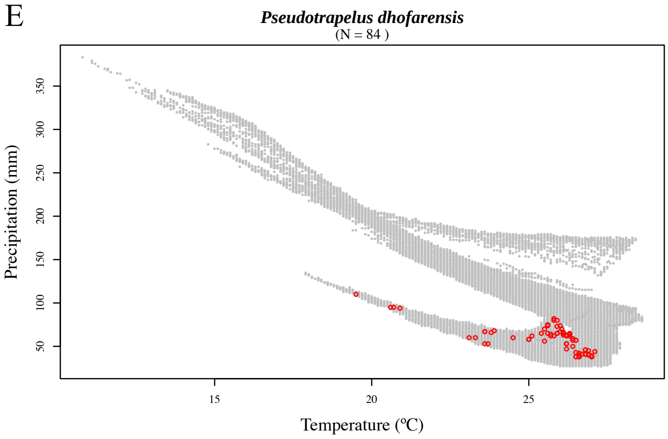

F

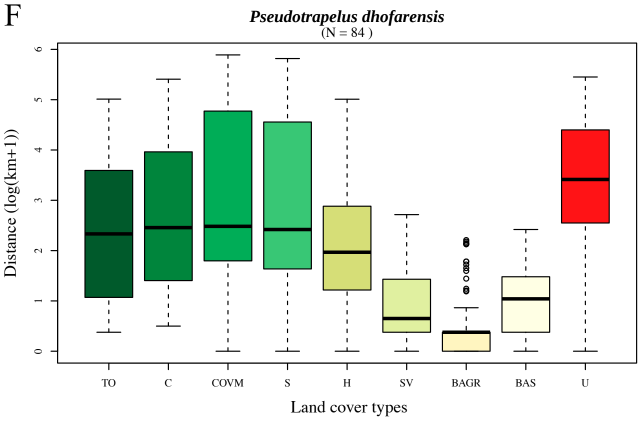

S7: Species information

Lizards

Agamids, Agamidae

Pseudotrapelus jensvindumi

Melnikov, Ananjeva & Papenfuss, 2013

A

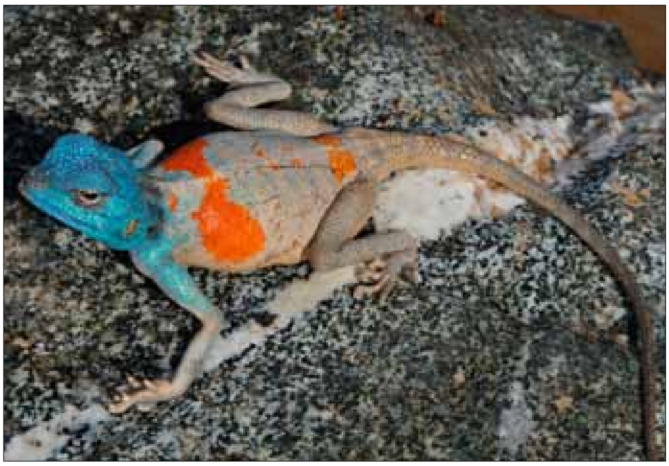

B

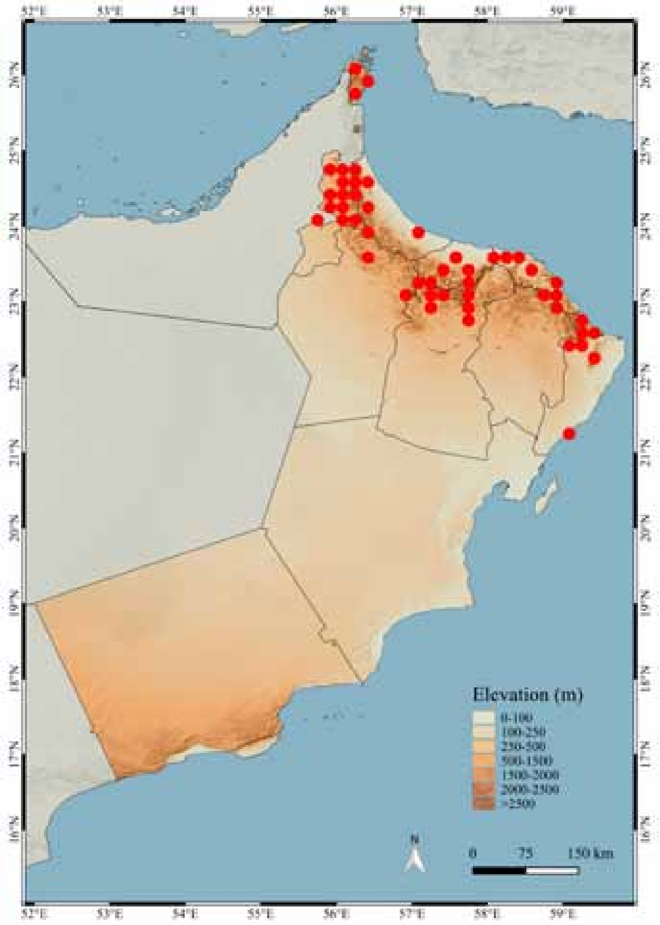

C

Useful information

IUCN Category	NE
Endemic	NO
Venomous	NO
Insular	NO
Present in a protected area	YES

D

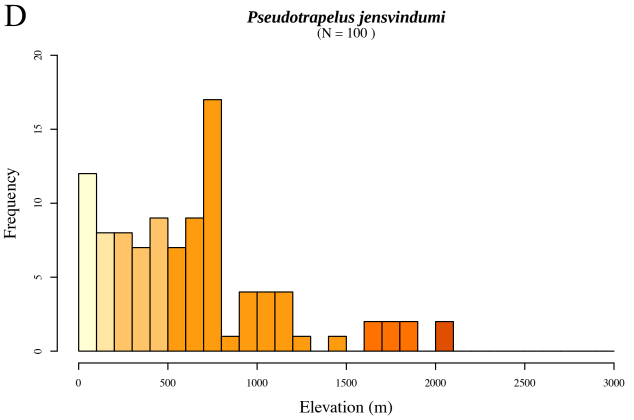

E

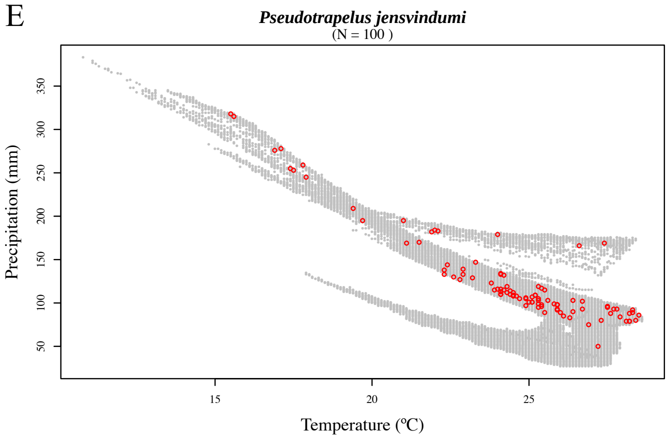

F

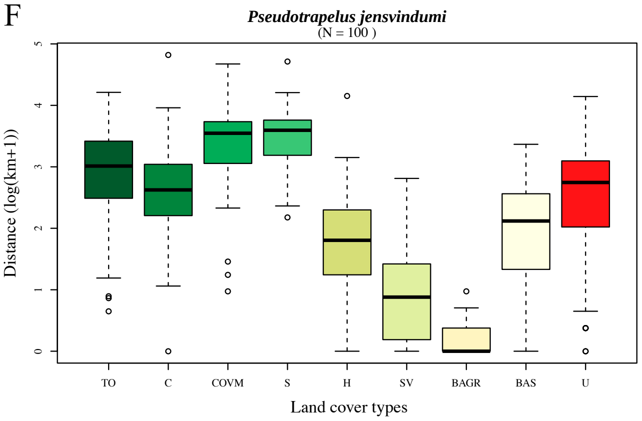

Trapelus flavimaculatus
Rüppell, 1835

S8: Species information
Lizards
Agamids, Agamidae

A

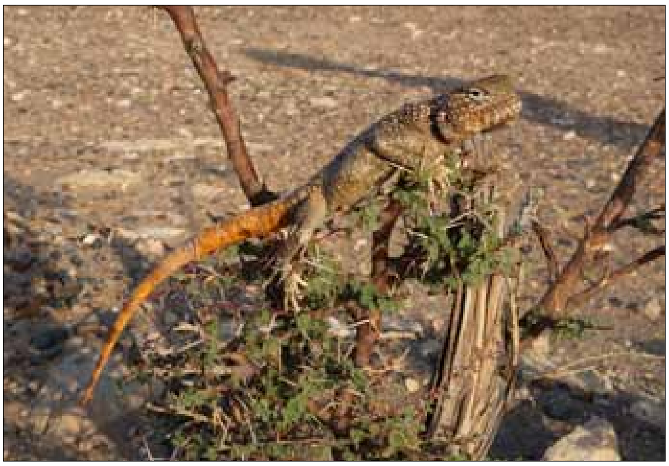

B

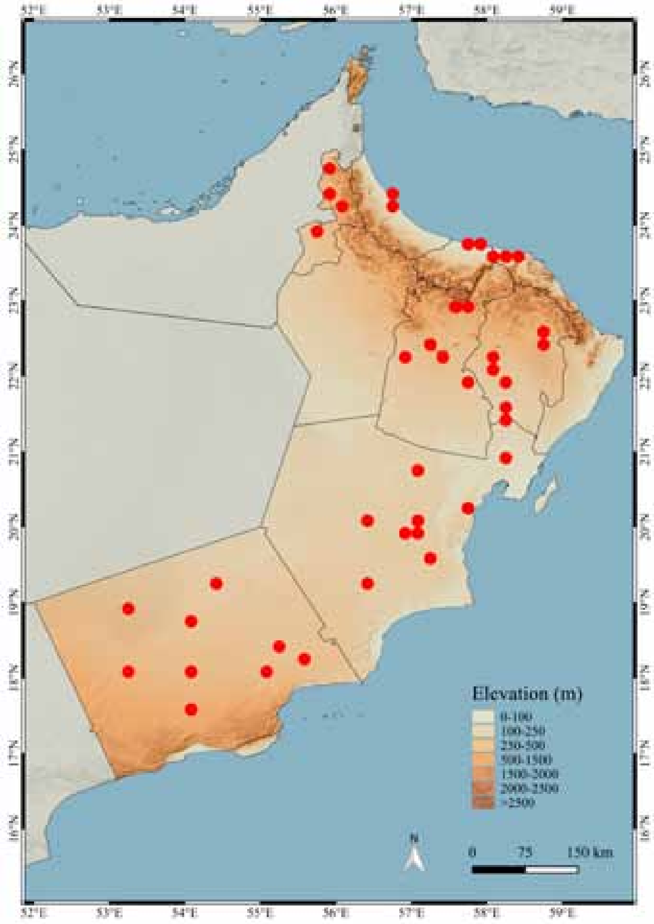

C

Useful information	
IUCN Category	LC
Endemic	NO
Venomous	NO
Insular	NO
Present in a protected area	YES

D

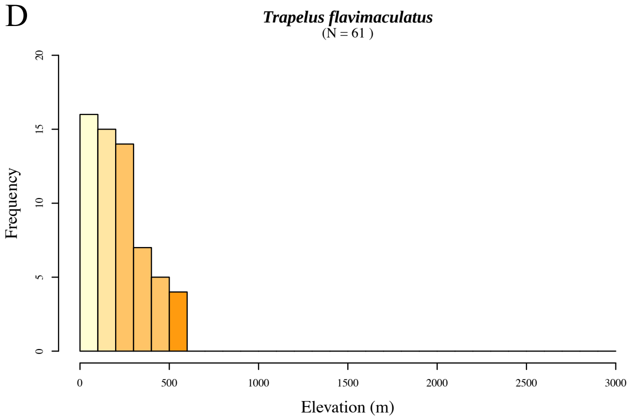

E

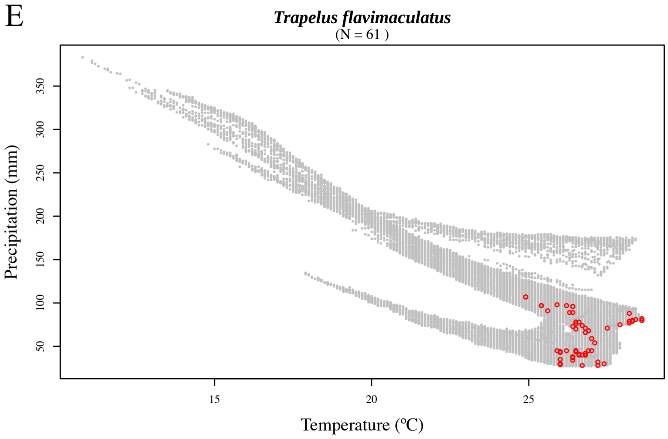

F

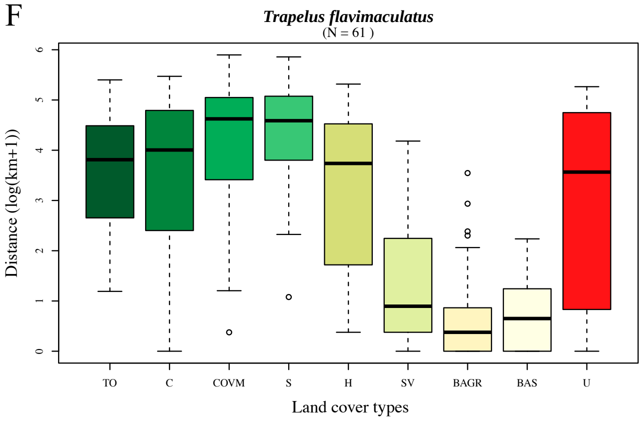

S9: Species information

Lizards

Agamids, Agamidae

Uromastyx aegyptia leptieni

Wilms & Böhme, 2000

A

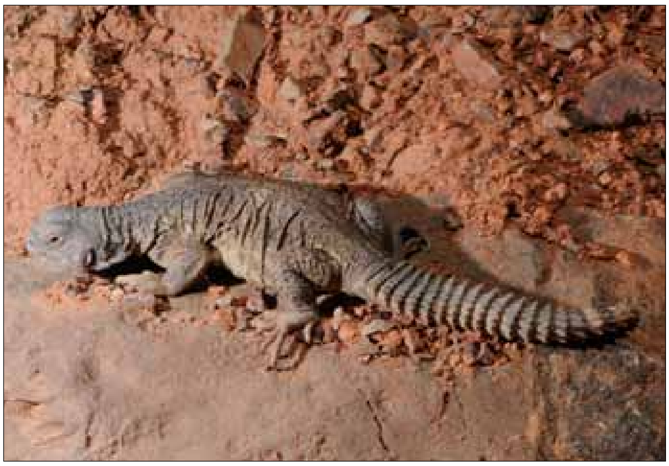

B

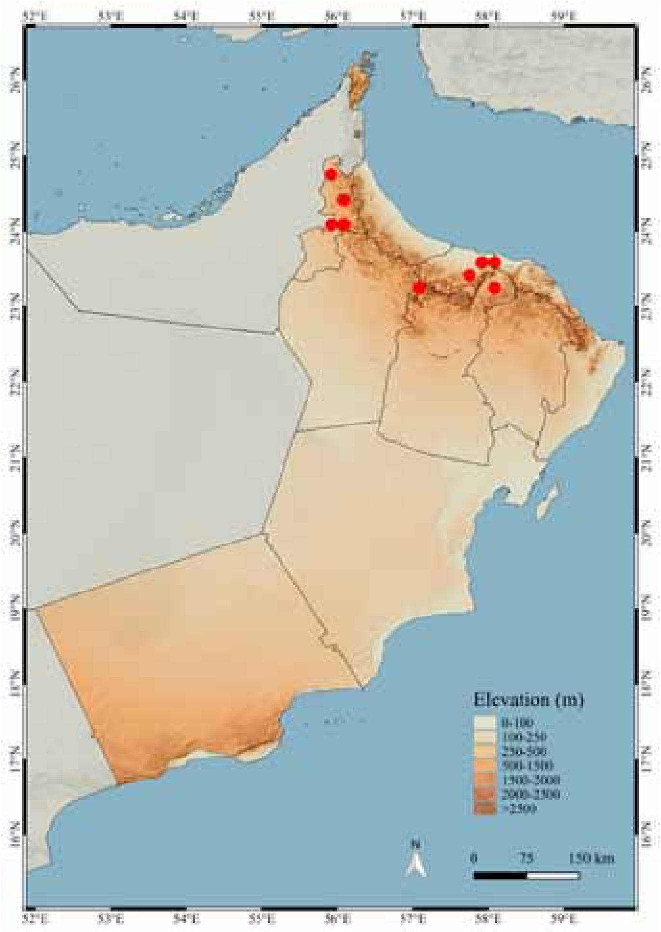

C

Useful information	
IUCN Category	VU
Endemic	NO
Venomous	NO
Insular	NO
Present in a protected area	NO

D

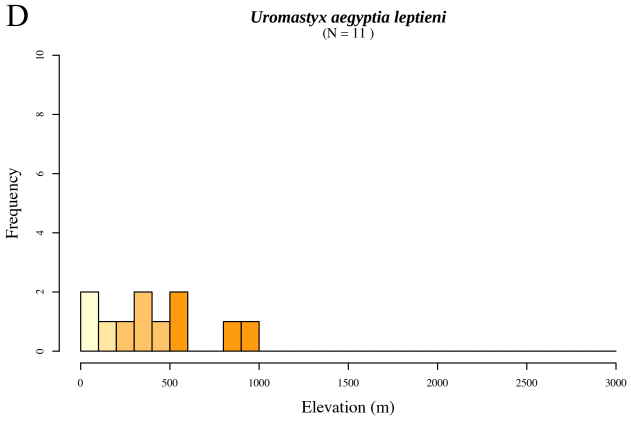

E

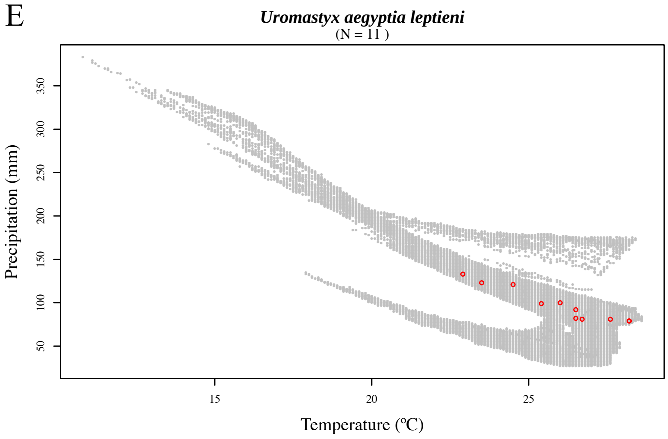

F

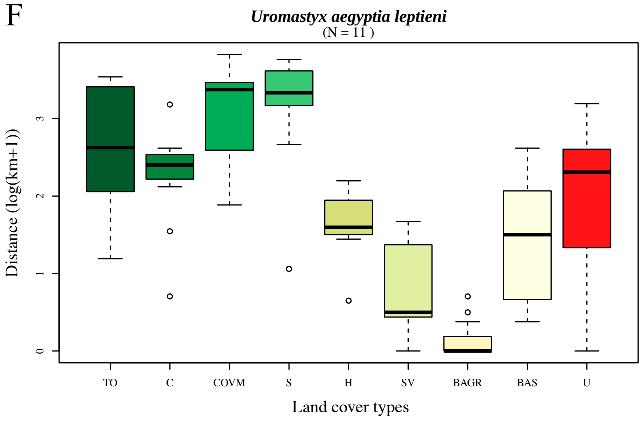

Uromastyx aegyptia microlepis
Arnold, 1980

S10: Species information
Lizards
Agamids, Agamidae

A

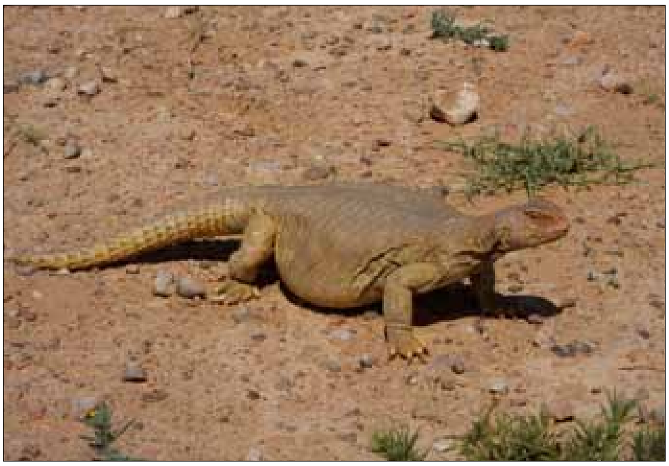

B

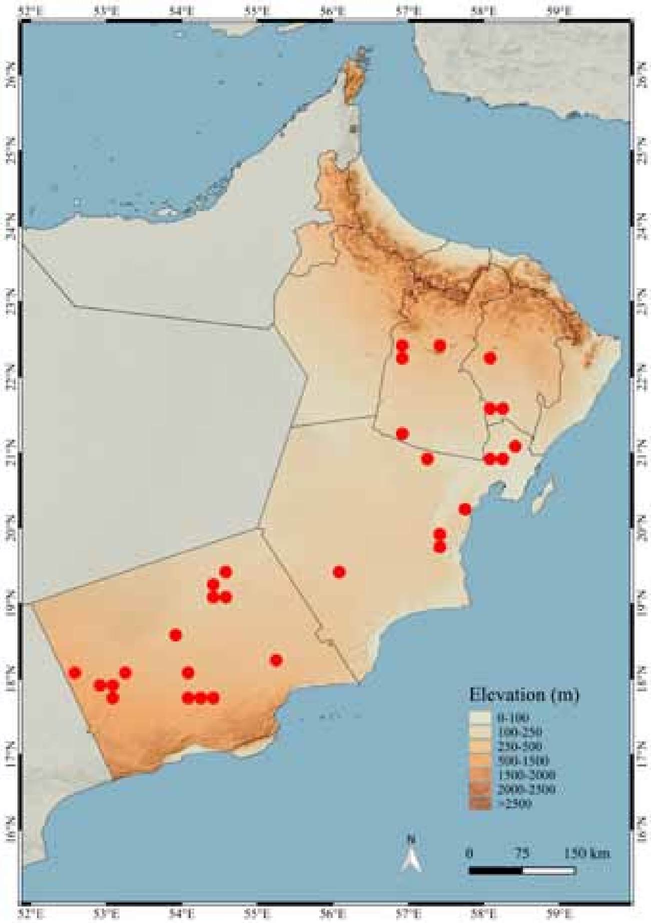

C

Useful information	
IUCN Category	VU
Endemic	NO
Venomous	NO
Insular	NO
Present in a protected area	YES

D

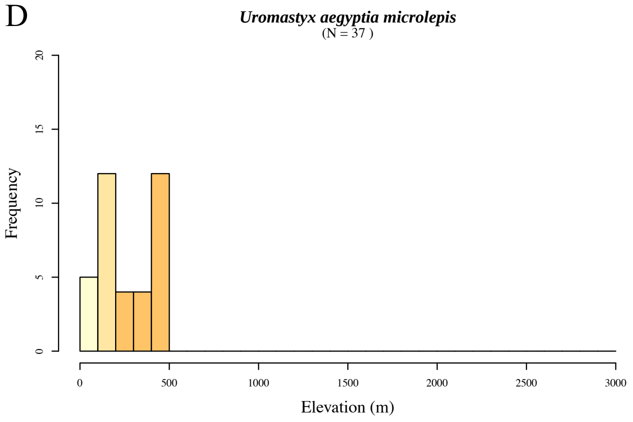

E

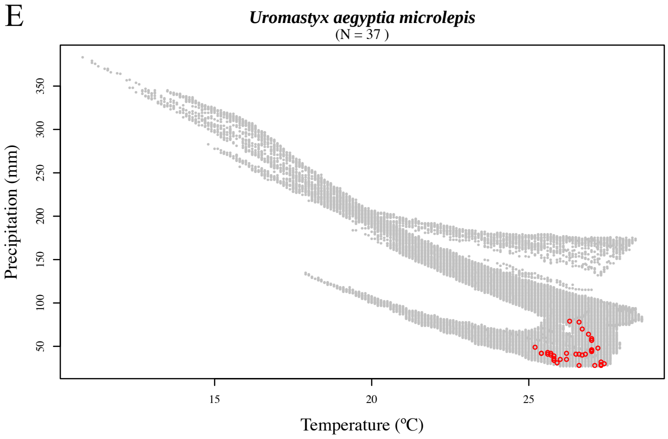

F

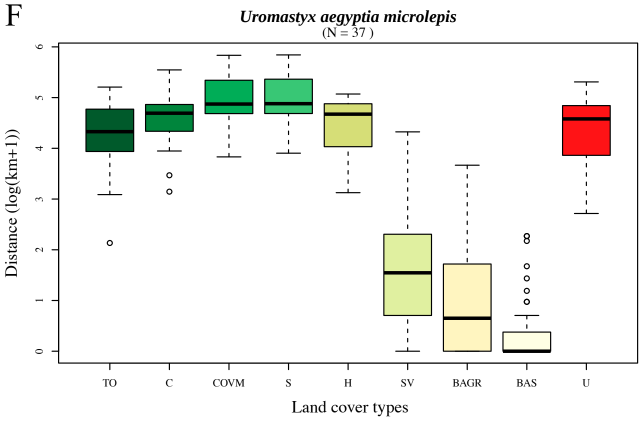

S11: Species information

Lizards

Agamids, Agamidae

Uromastyx benti
(Anderson, 1894)

A

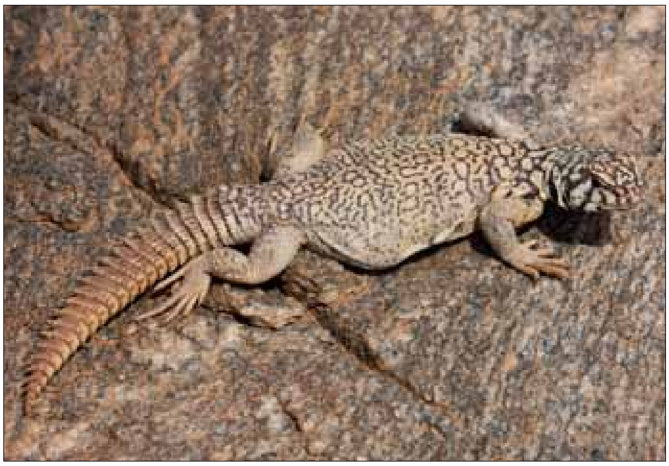

B

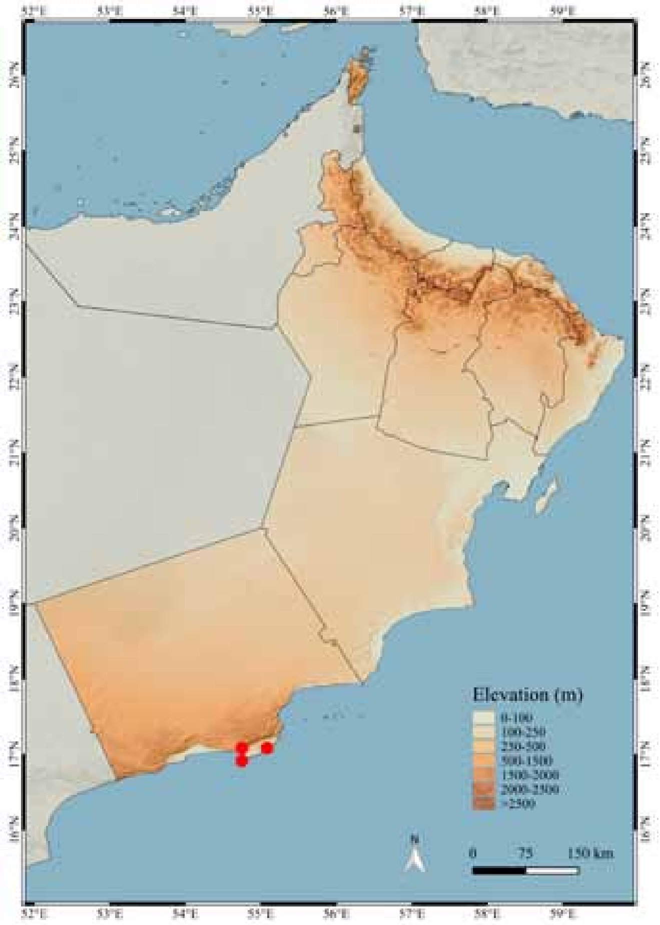

C

Useful information	
IUCN Category	LC
Endemic	NO
Venomous	NO
Insular	NO
Present in a protected area	NO

D

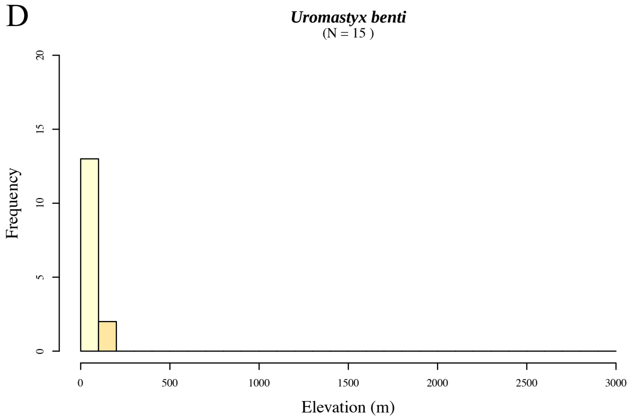

E

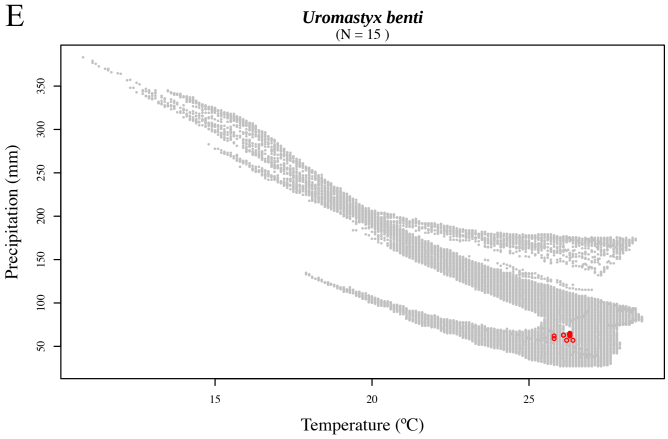

F

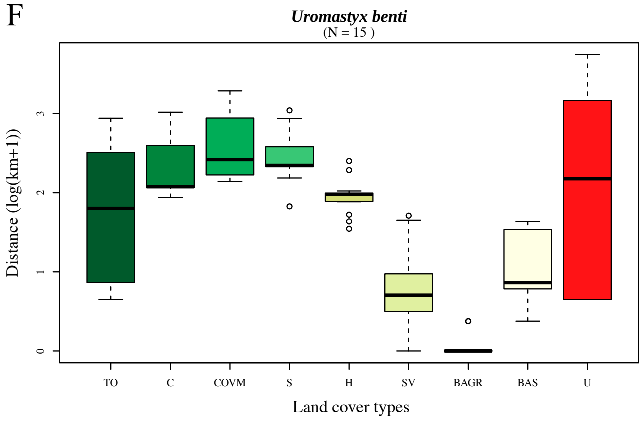

Uromastyx thomasi
Parker, 1930

S12: Species information
Lizards
Agamids, Agamidae

A

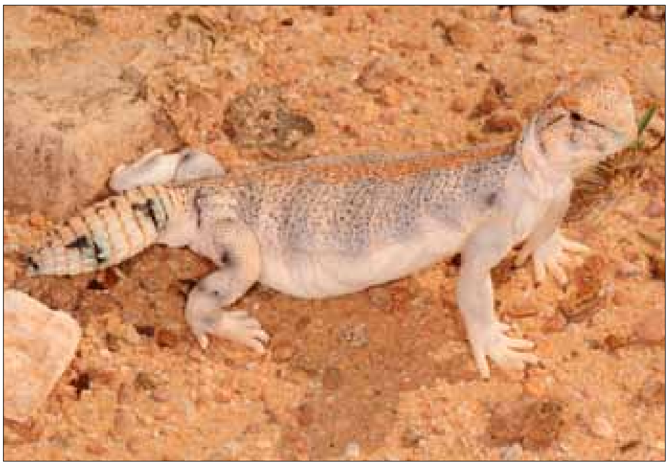

B

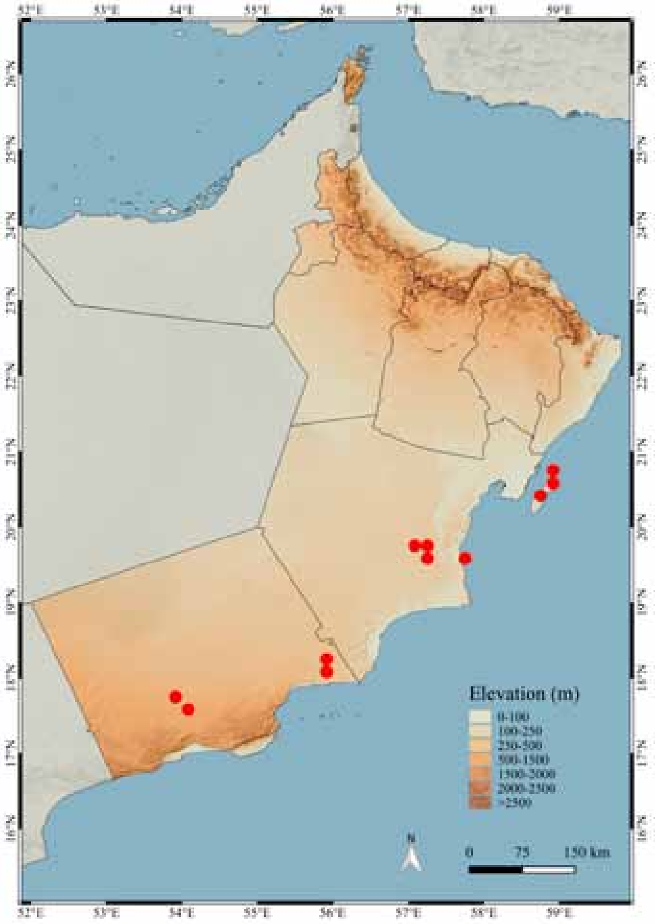

C

Useful information	
IUCN Category	VU
Endemic	YES
Venomous	NO
Insular	YES
Present in a protected area	YES

D

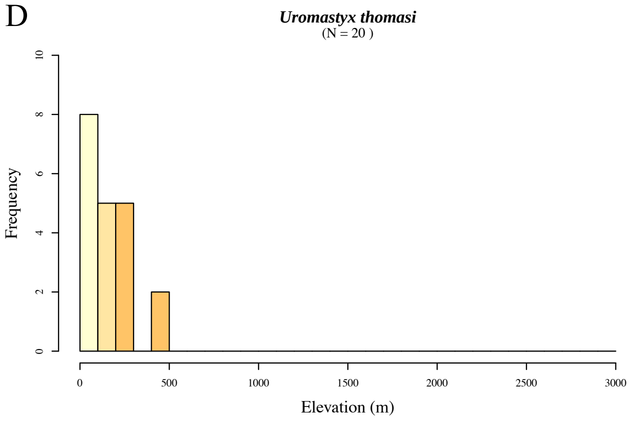

E

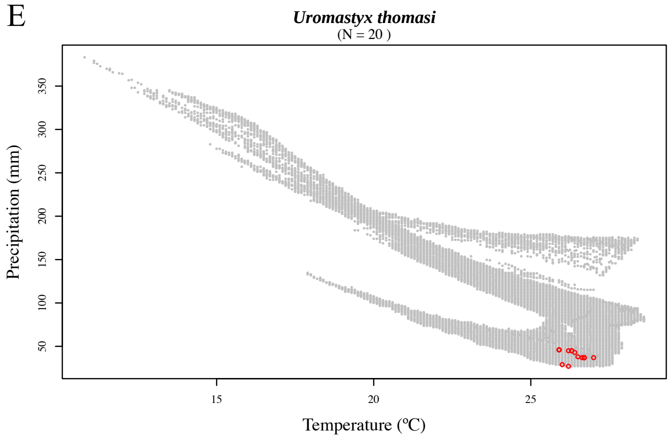

F

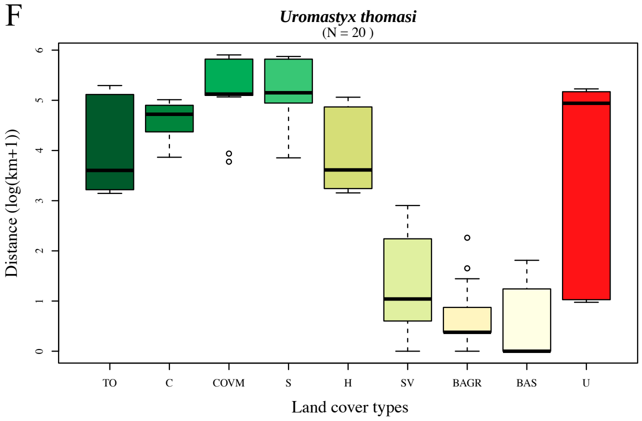

S13: Species information

Lizards

Agamids, Chamaeleonidae

Chamaeleo arabicus
Matschie, 1893

A

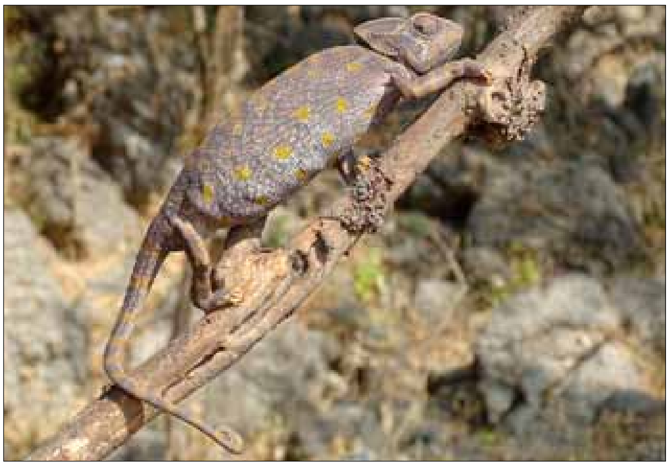

B

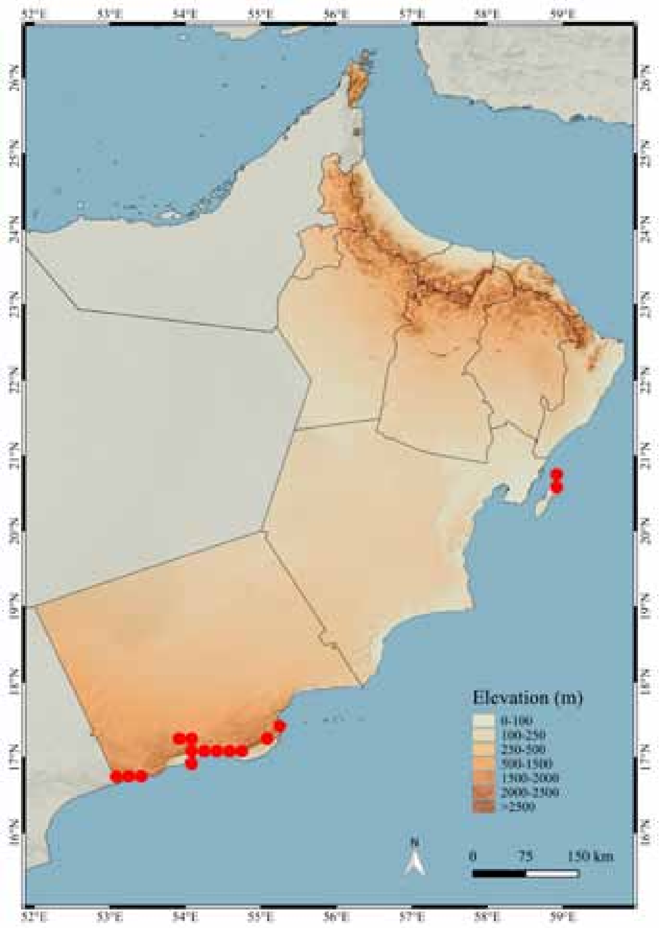

C

Useful information	
IUCN Category	LC
Endemic	NO
Venomous	NO
Insular	YES
Present in a protected area	YES

D

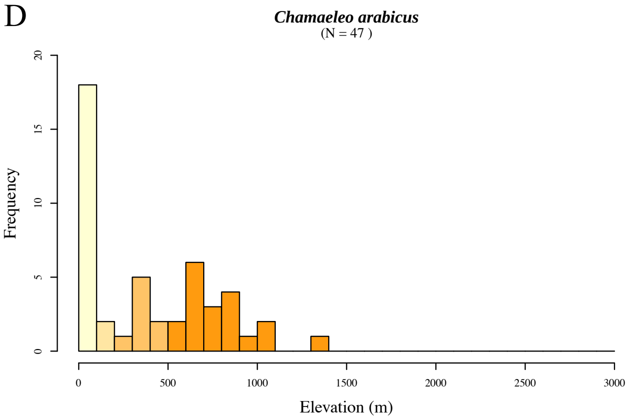

E

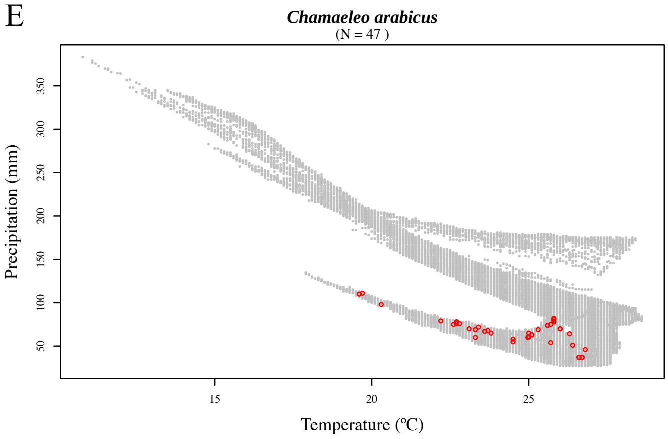

F

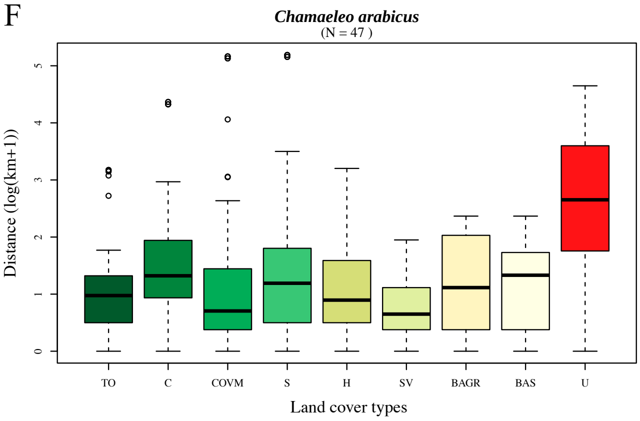

Bunopus tuberculatus
Blanford, 1874

S14: Species information
Lizards
Geckos, Gekkonidae

A

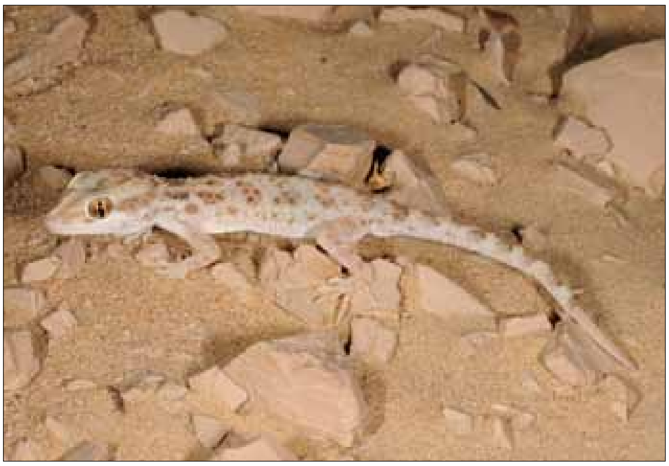

B

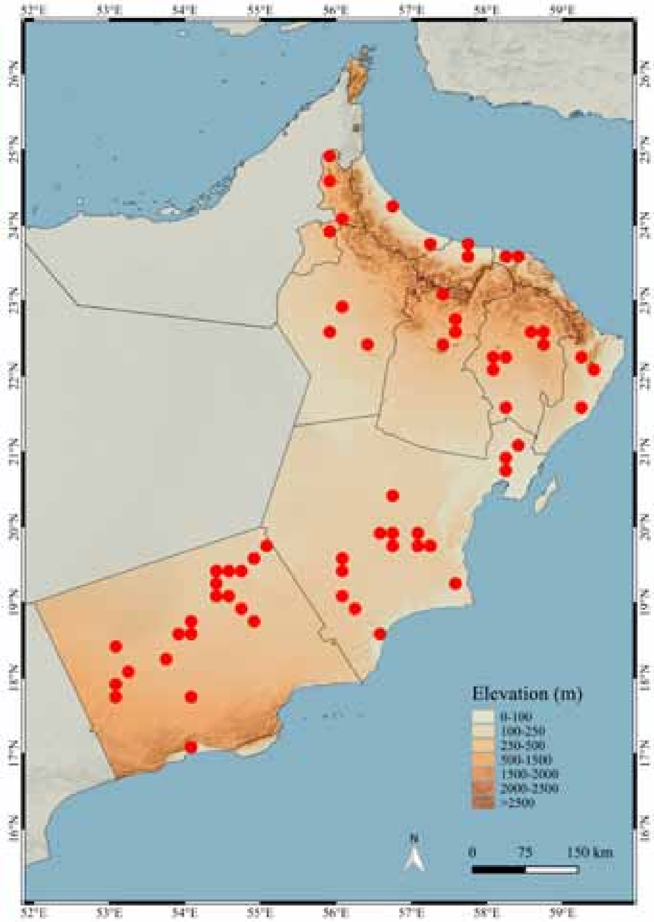

C

Useful information	
IUCN Category	LC
Endemic	NO
Venomous	NO
Insular	NO
Present in a protected area	YES

D

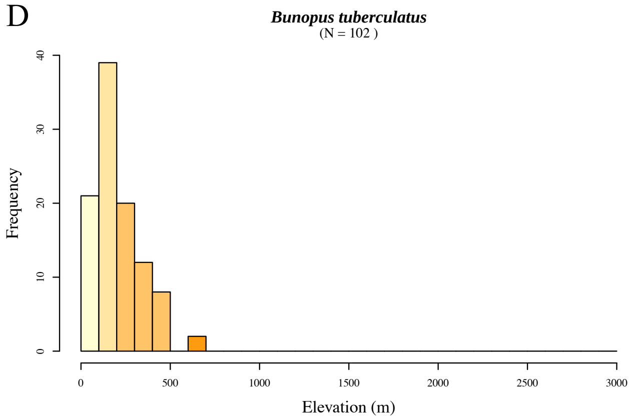

E

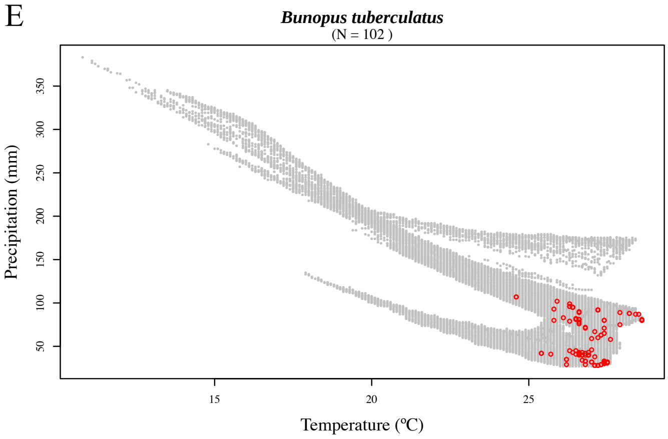

F

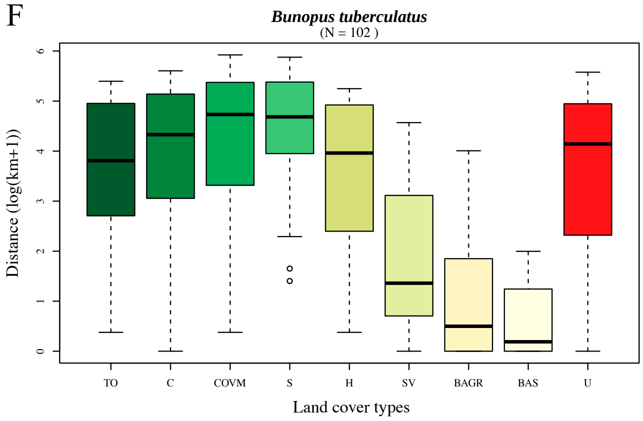

S15: Species information
Lizards
Geckos, Gekkonidae

Cyrtopodion scabrum
(Heyden, 1827)

B

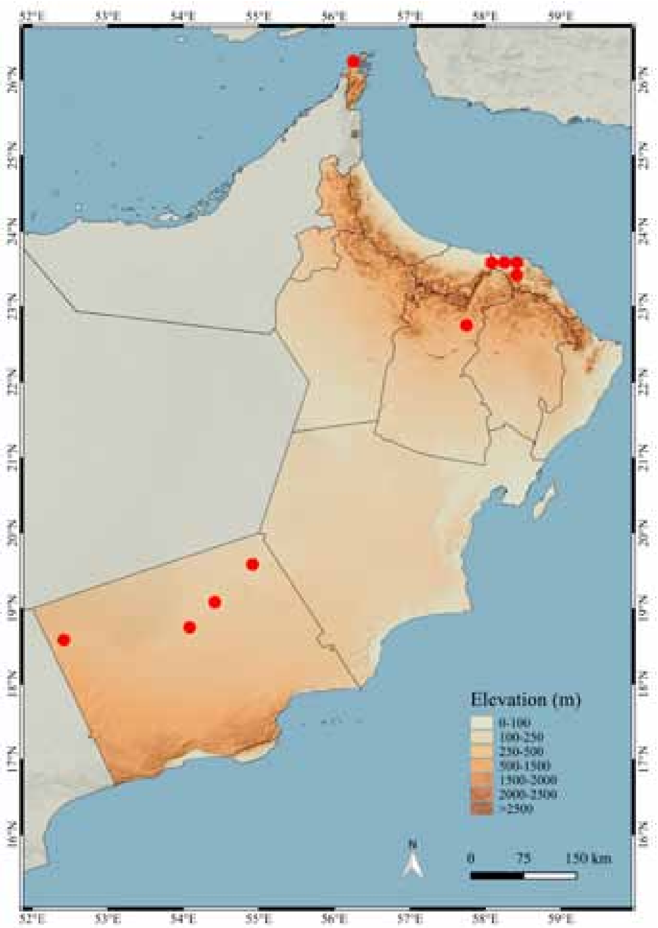

A

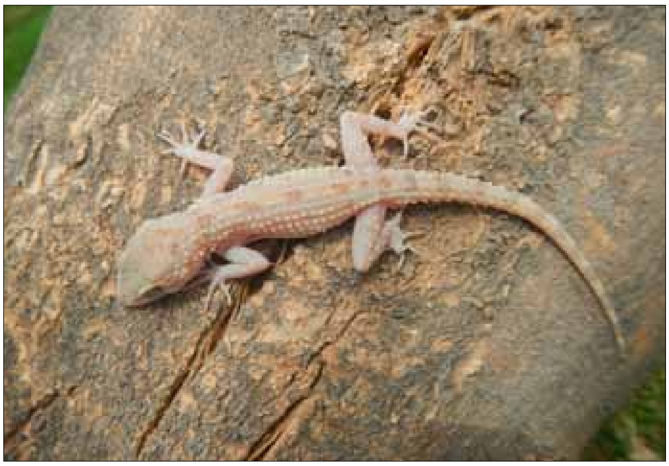

C

Useful information	
IUCN Category	LC
Endemic	NO
Venomous	NO
Insular	NO
Present in a protected area	NO

D

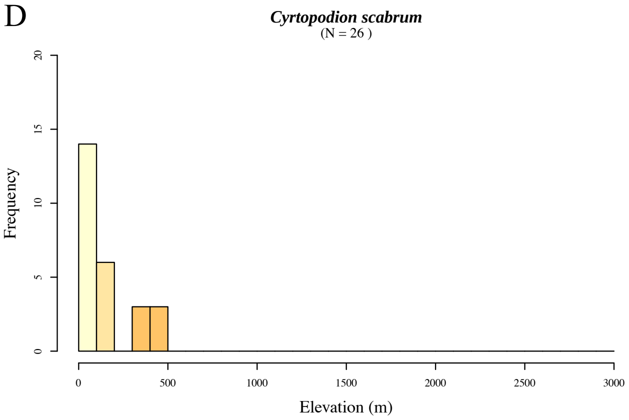

E

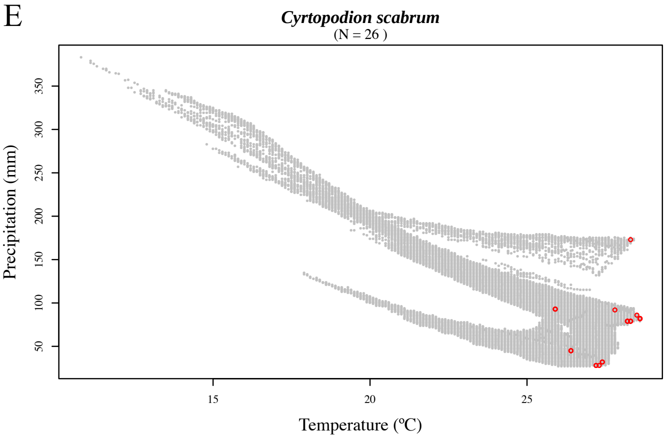

F

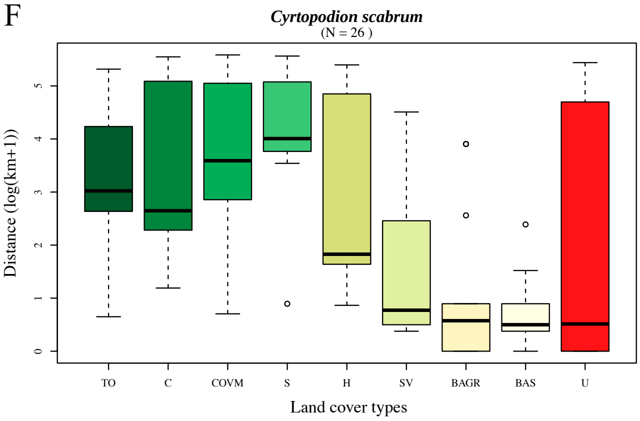

Hemidactylus alkiyumii
Carranza & Arnold, 2012

S16: Species information
Lizards
Geckos, Gekkonidae

A

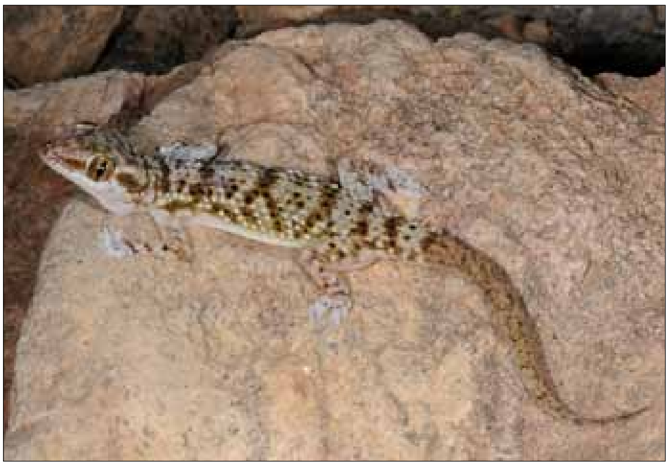

B

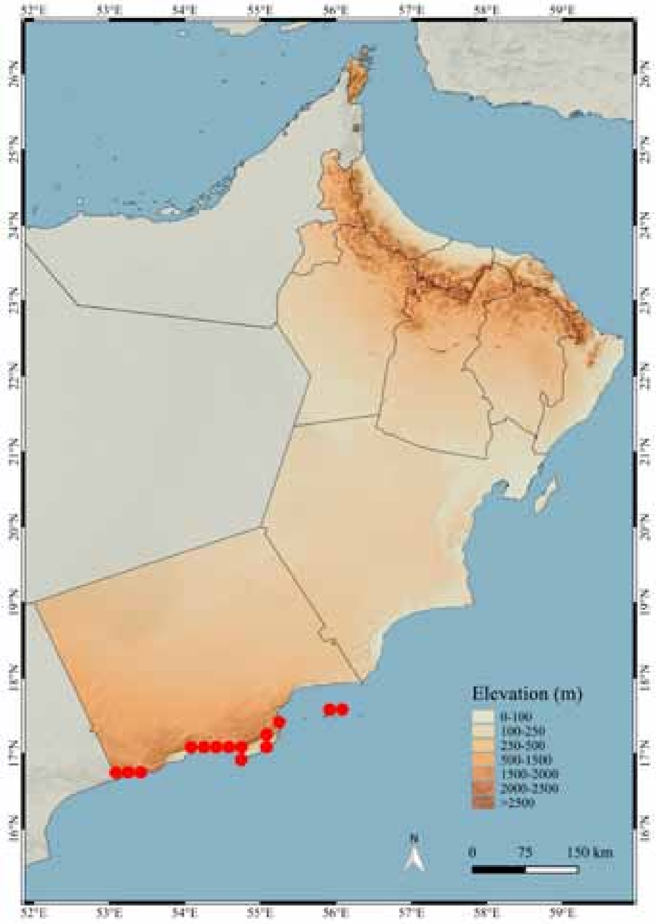

C

Useful information	
IUCN Category	NE
Endemic	NO
Venomous	NO
Insular	YES
Present in a protected area	YES

D

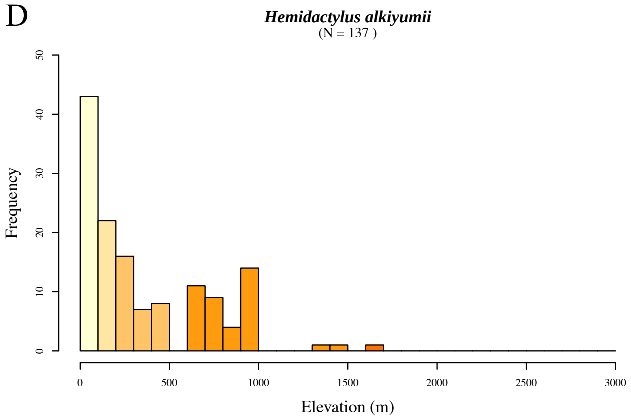

E

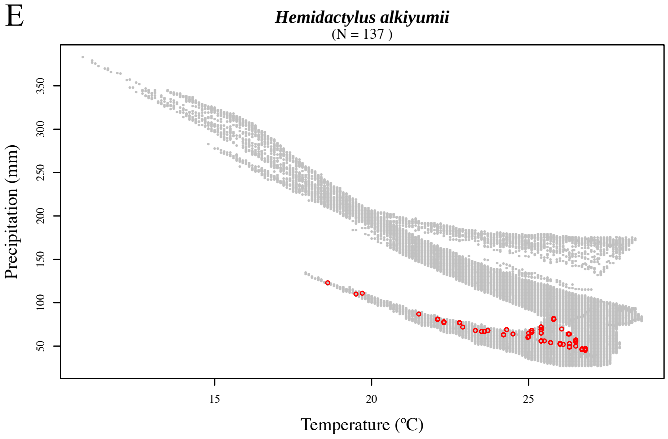

F

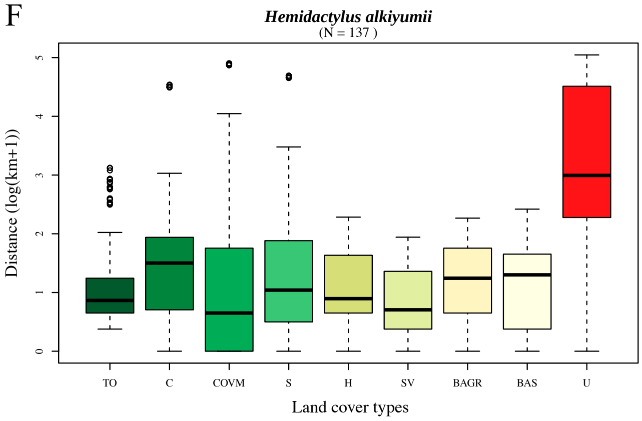

S17: Species information

Lizards

Geckos, Gekkonidae

Hemidactylus endophis

Carranza & Arnold, 2012

A

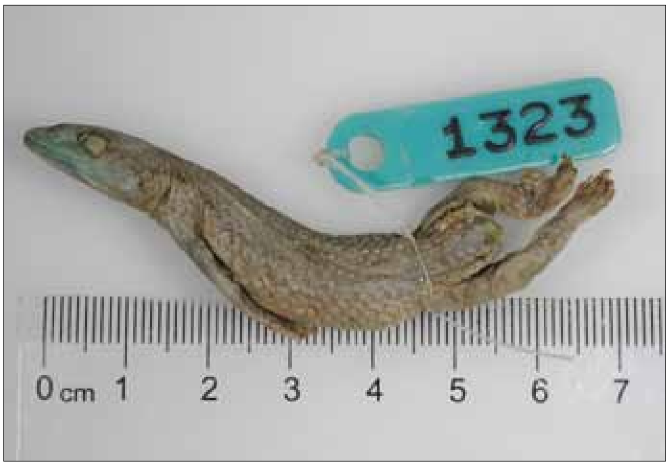

Specimen code: **BMNH1976.1323**

B

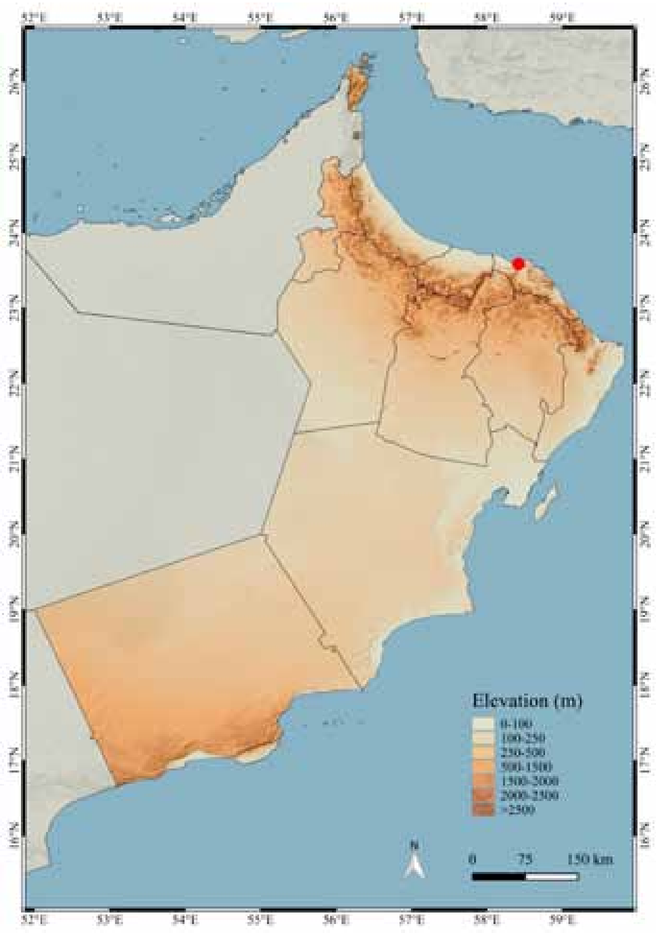

C

Useful information	
IUCN Category	NE
Endemic	YES
Venomous	NO
Insular	NO
Present in a protected area	NO

D

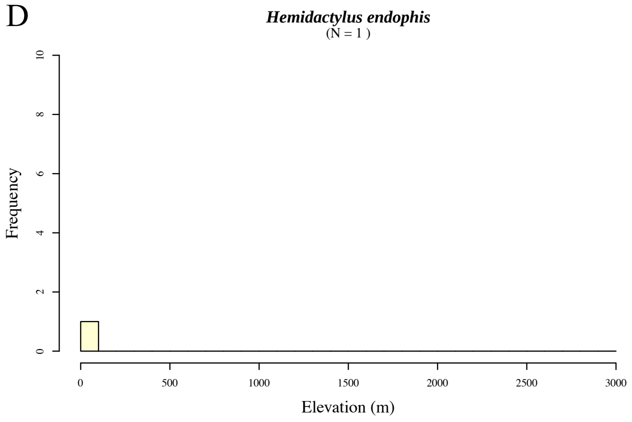

E

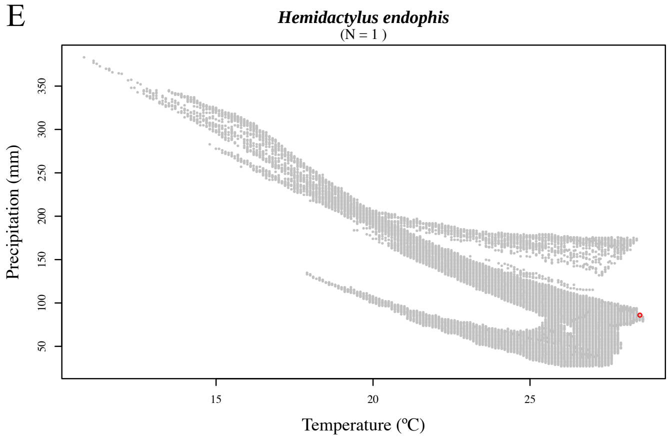

F

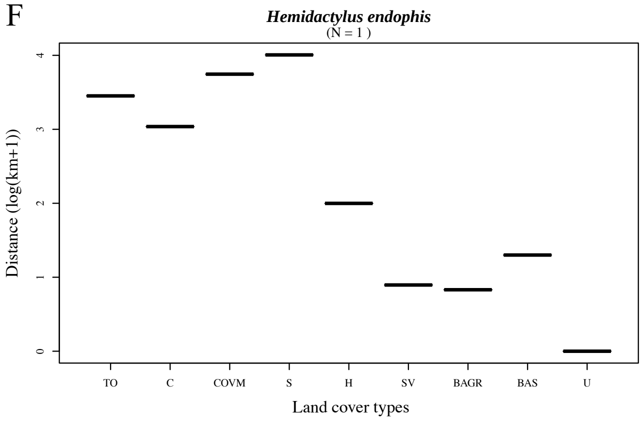

Hemidactylus festivus
Carranza & Arnold, 2012

S18: Species information
Lizards
Geckos, Gekkonidae

A

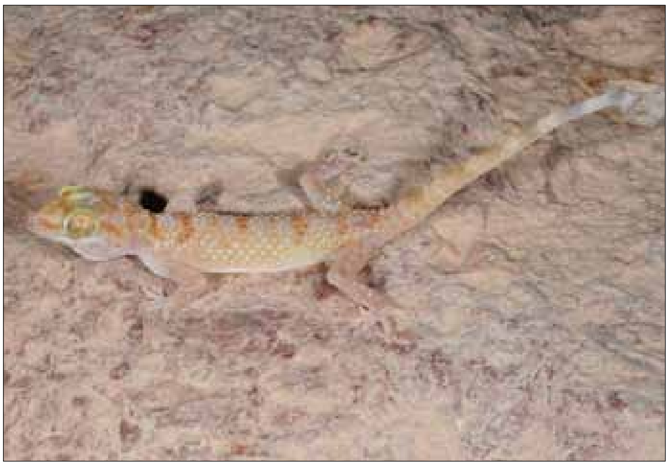

B

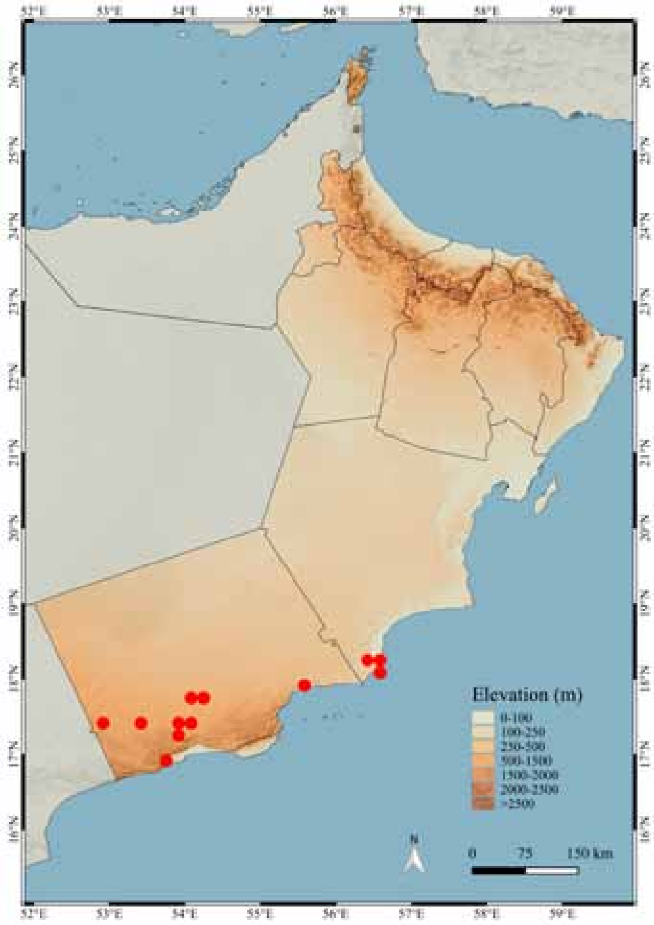

C

Useful information	
IUCN Category	NE
Endemic	NO
Venomous	NO
Insular	NO
Present in a protected area	NO

D

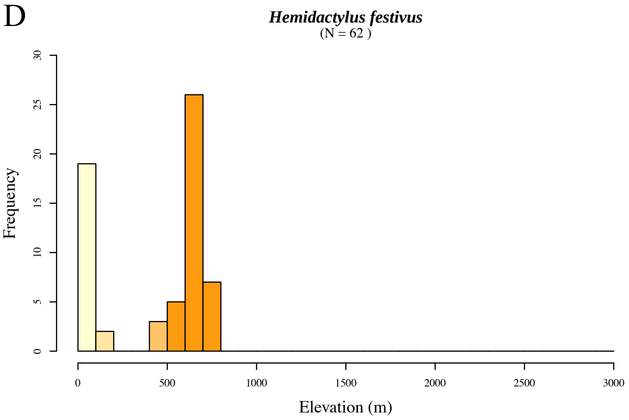

E

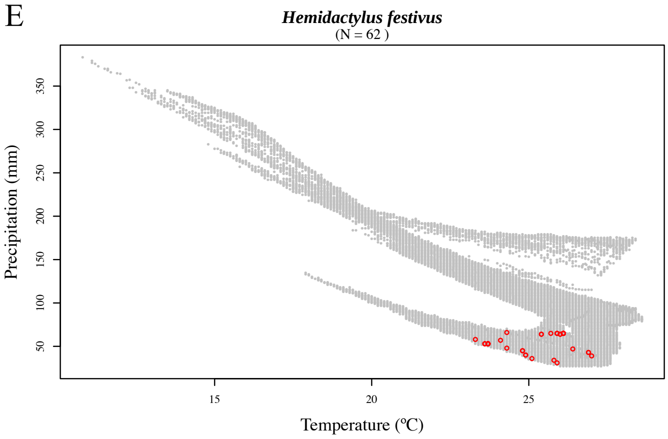

F

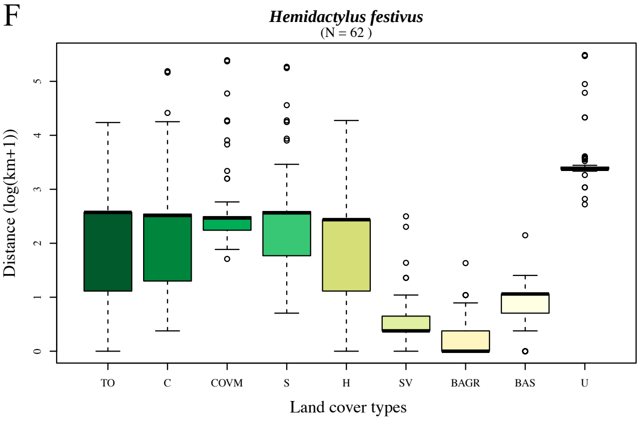

S19: Species information

Lizards

Geckos, Gekkonidae

Hemidactylus flaviviridis
Rüppell, 1835

A

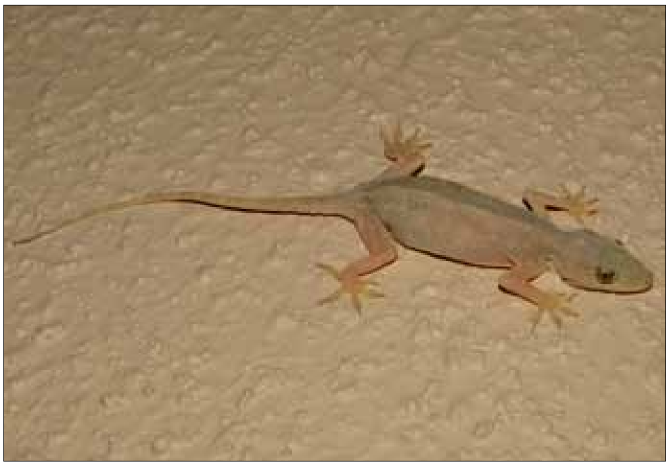

B

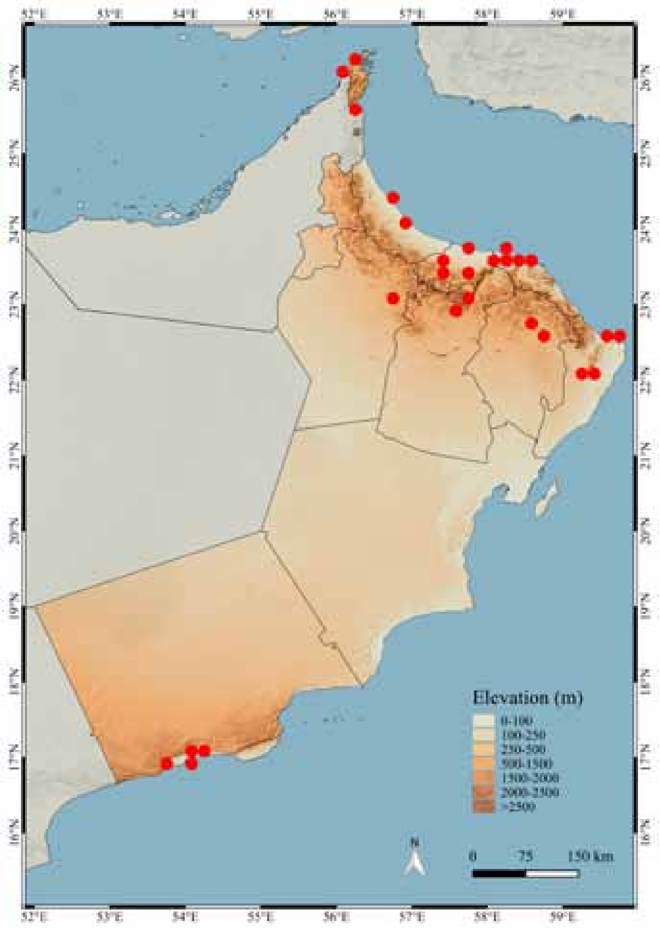

C

Useful information	
IUCN Category	LC*
Endemic	NO
Venomous	NO
Insular	NO
Present in a protected area	YES

*Not available on the web

D

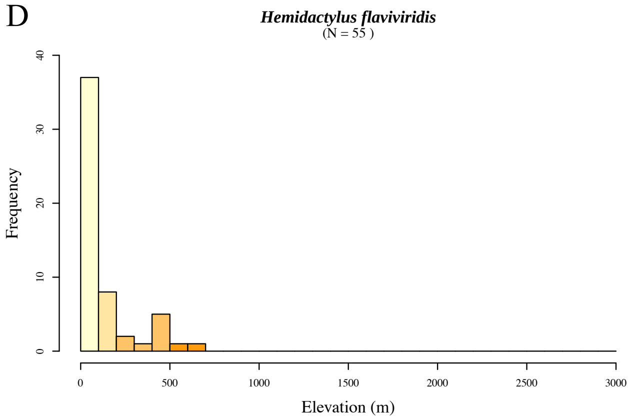

E

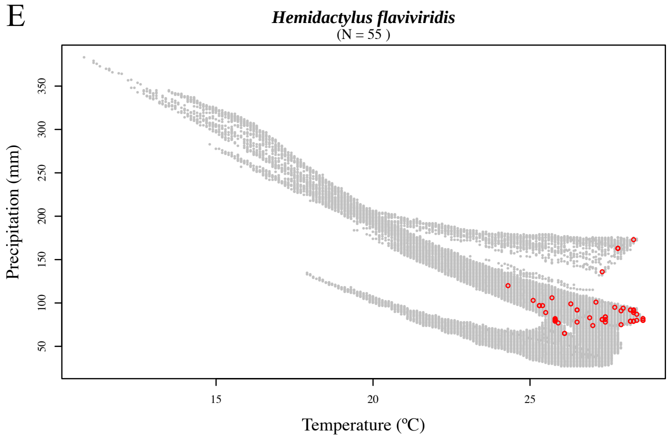

F

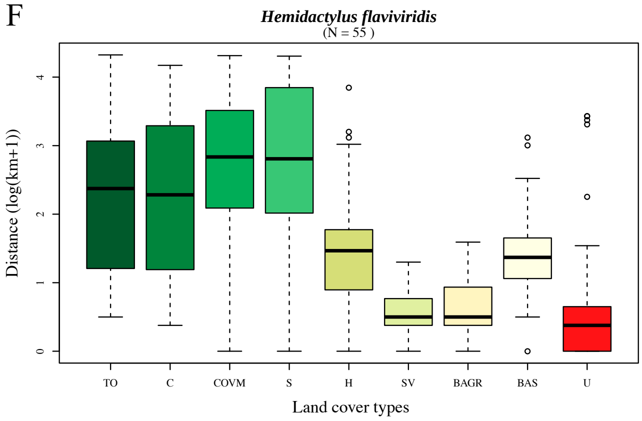

Hemidactylus hajarensis
Carranza & Arnold, 2012

S20: Species information
Lizards
Geckos, Gekkonidae

A

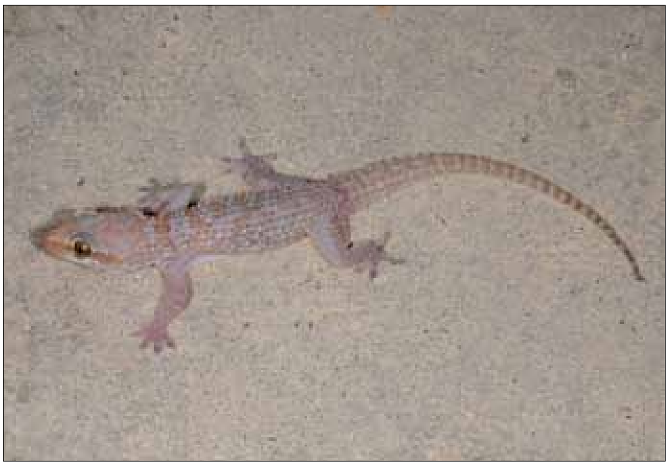

B

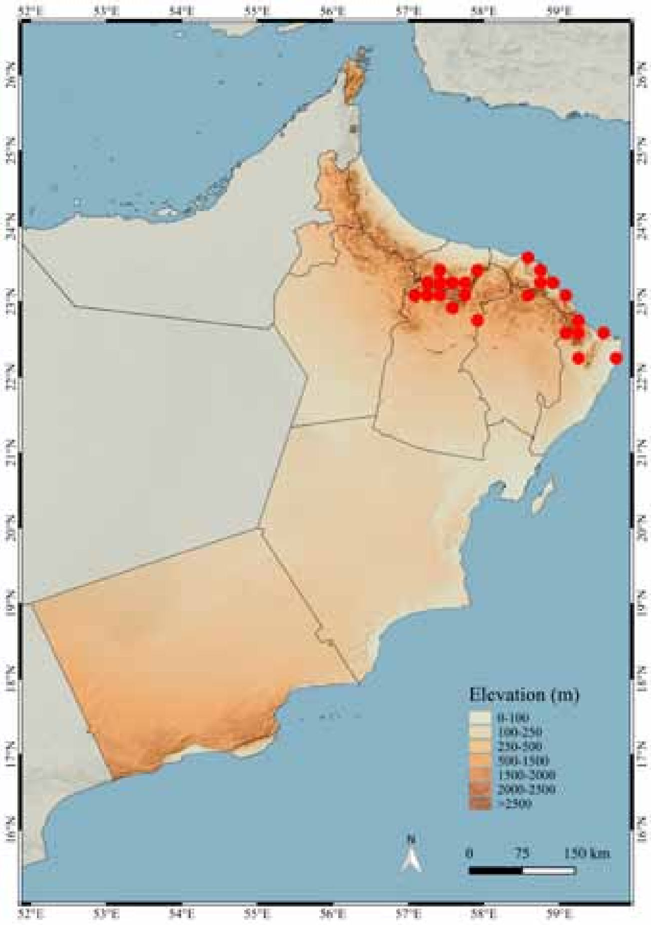

C

Useful information	
IUCN Category	NE
Endemic	YES
Venomous	NO
Insular	NO
Present in a protected area	YES

D

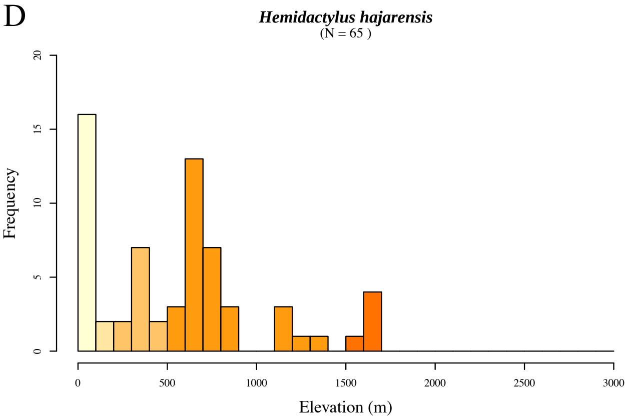

E

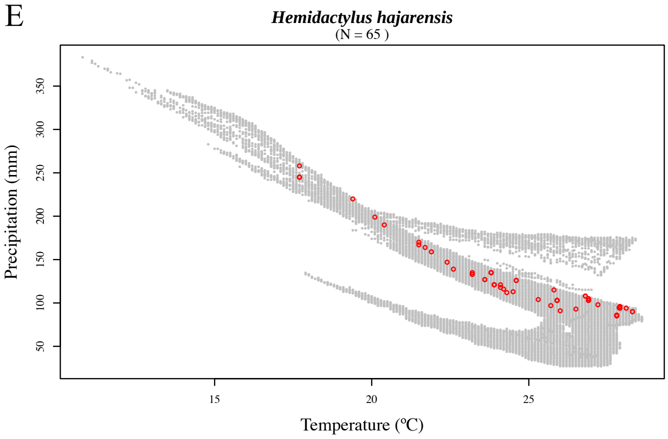

F

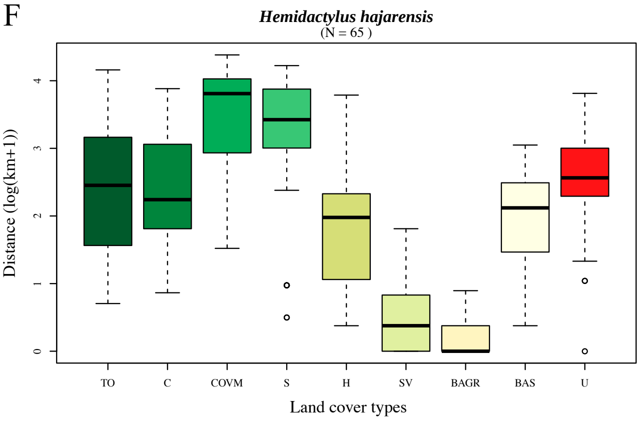

S21: Species information

Lizards

Geckos, Gekkonidae

Hemidactylus inexpectatus

Carranza & Arnold, 2012

B

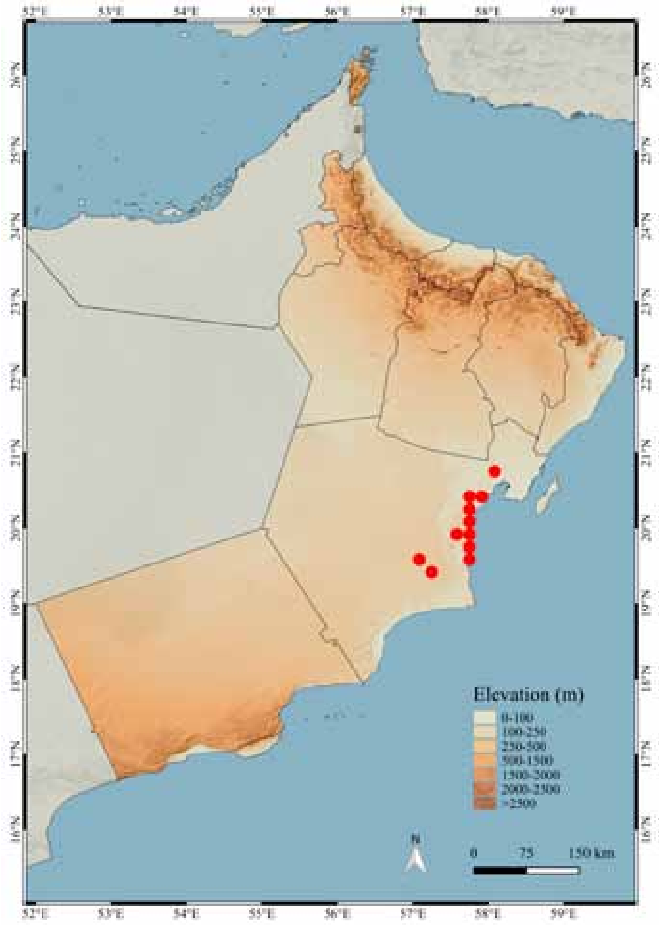

A

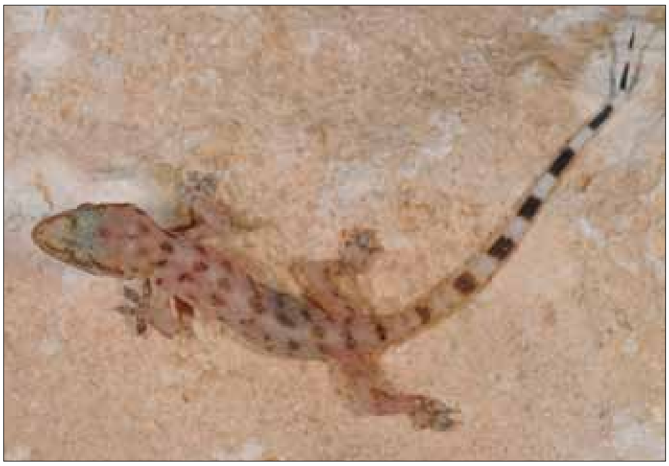

C

Useful information	
IUCN Category	NE
Endemic	YES
Venomous	NO
Insular	YES
Present in a protected area	NO

D

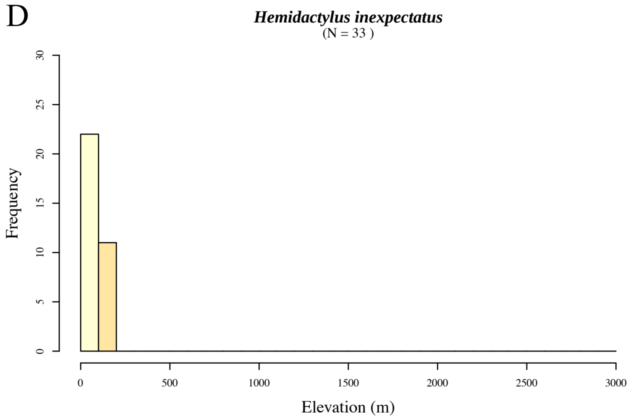

E

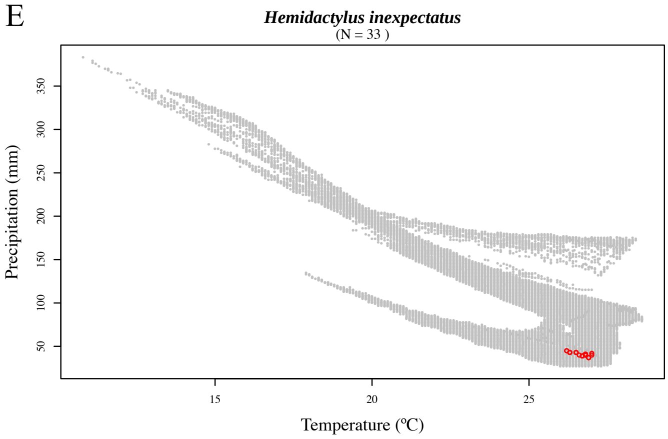

F

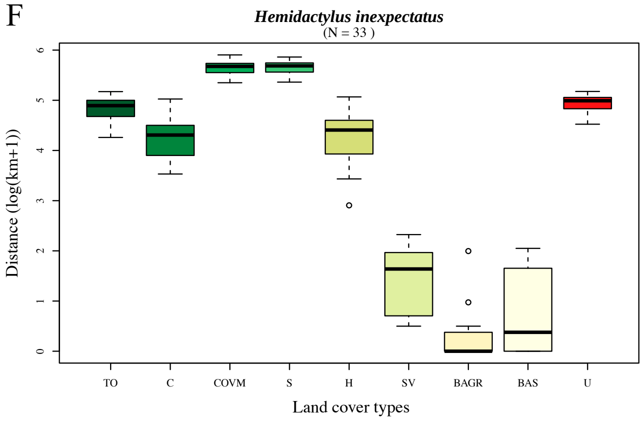

Hemidactylus lemurinus
Arnold, 1980

S22: Species information
Lizards
Geckos, Gekkonidae

A

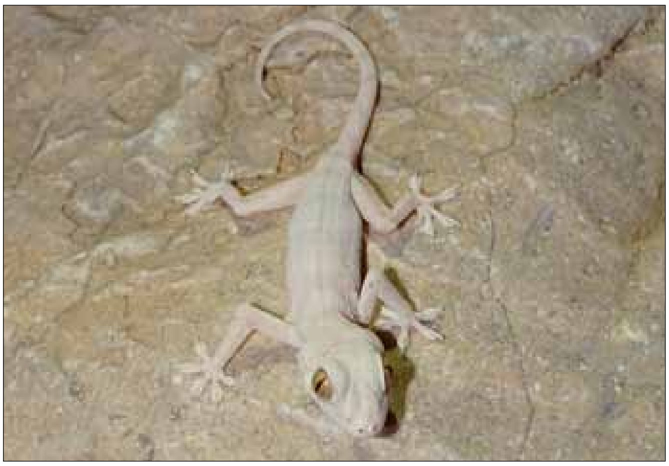

B

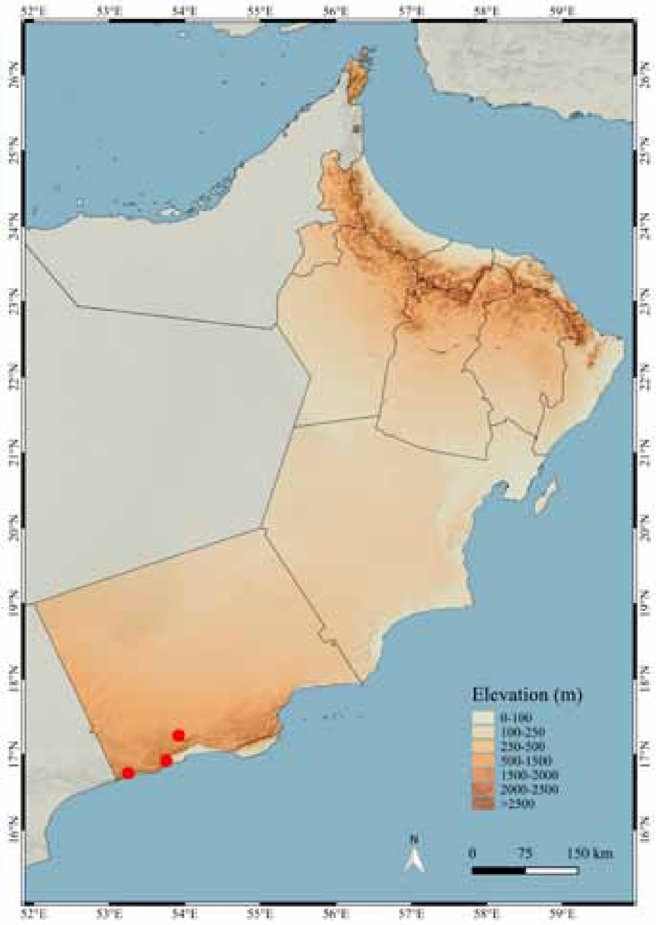

C

Useful information	
IUCN Category	DD
Endemic	NO
Venomous	NO
Insular	NO
Present in a protected area	NO

D

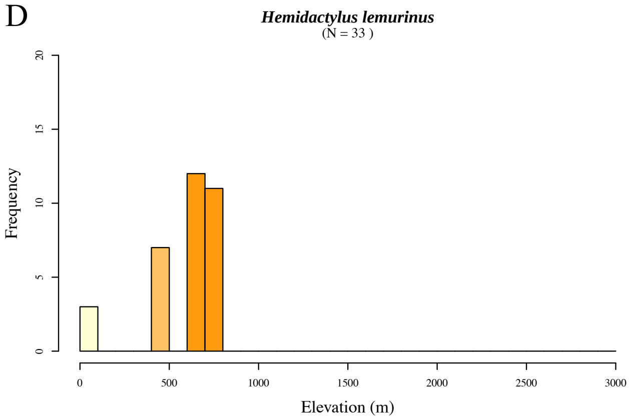

E

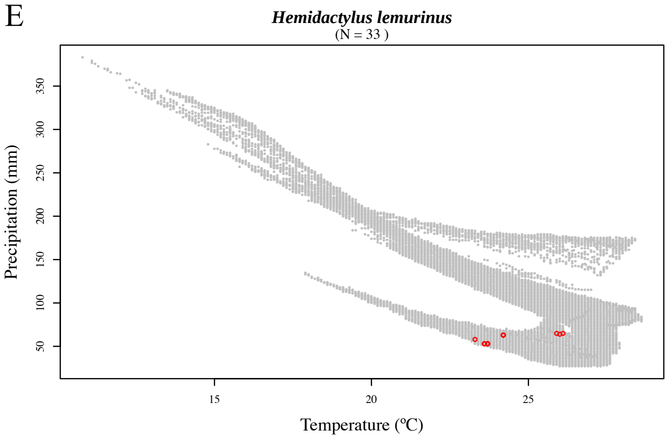

F

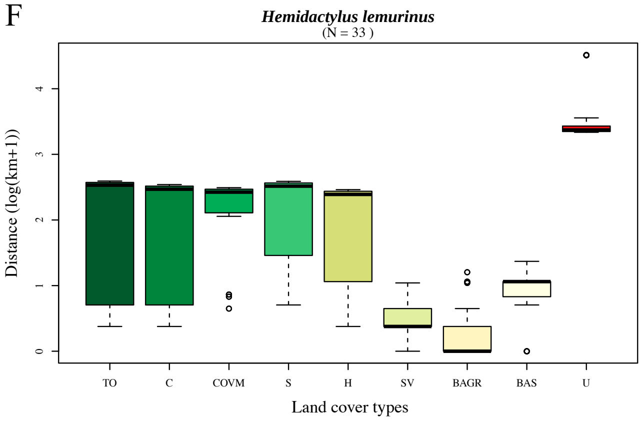

S23: Species information

Lizards

Geckos, Gekkonidae

Hemidactylus leschenaultii

Duméril & Bibron, 1836

A

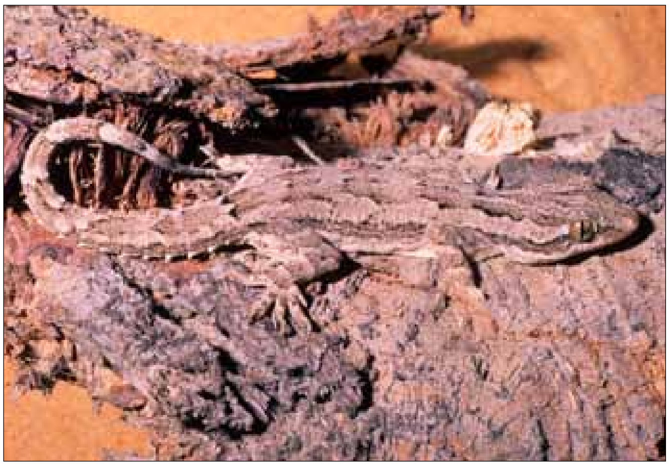

B

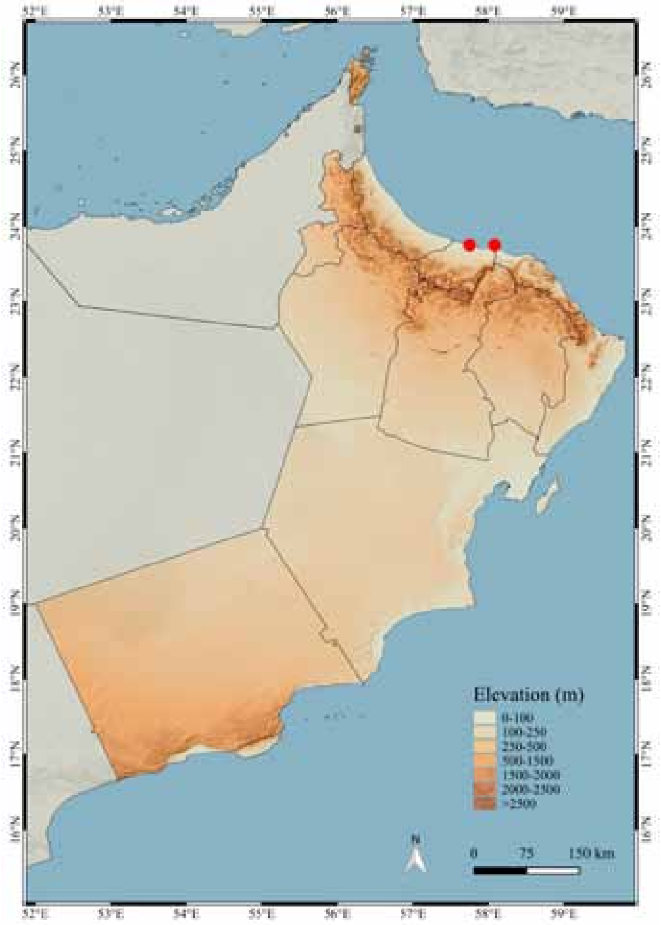

C

Useful information	
IUCN Category	LC*
Endemic	NO
Venomous	NO
Insular	NO
Present in a protected area	NO

*Not available on the web

D

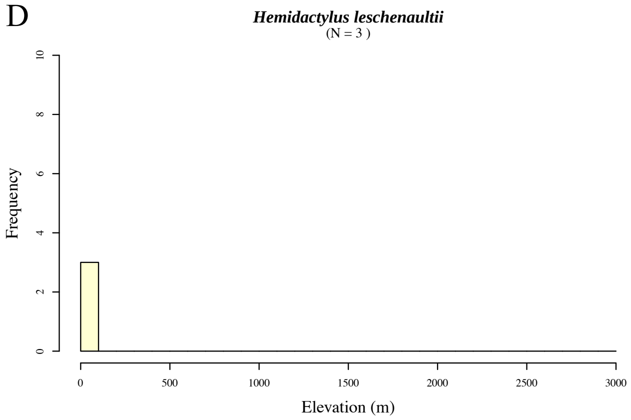

E

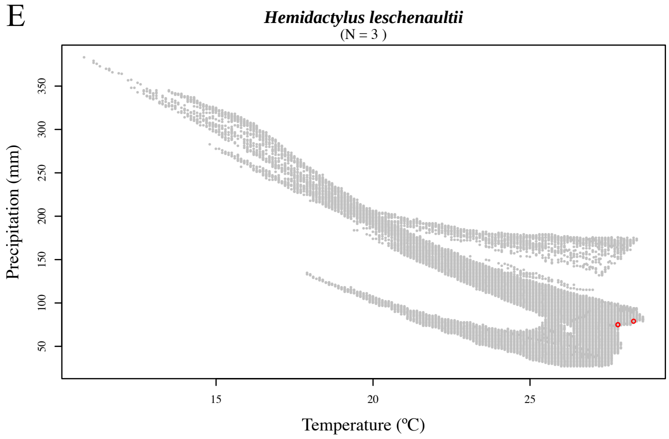

F

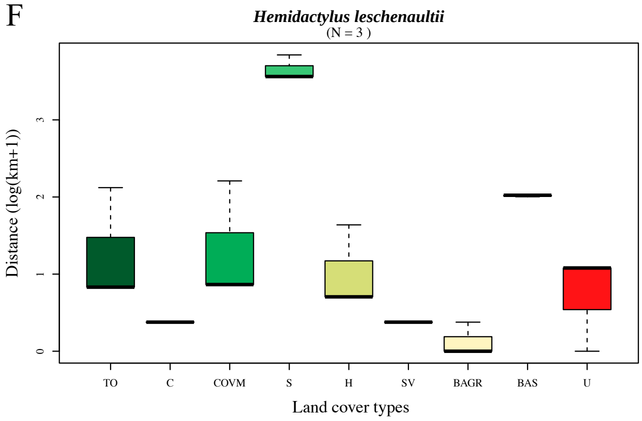

Hemidactylus luqueorum
Carranza & Arnold, 2012

S24: Species information
Lizards
Geckos, Gekkonidae

A

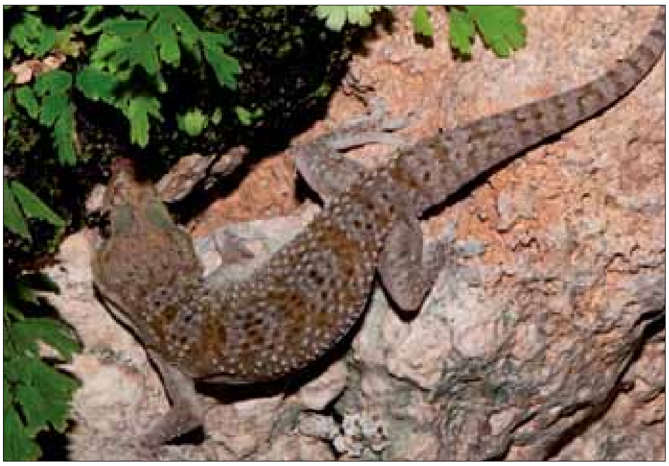

B

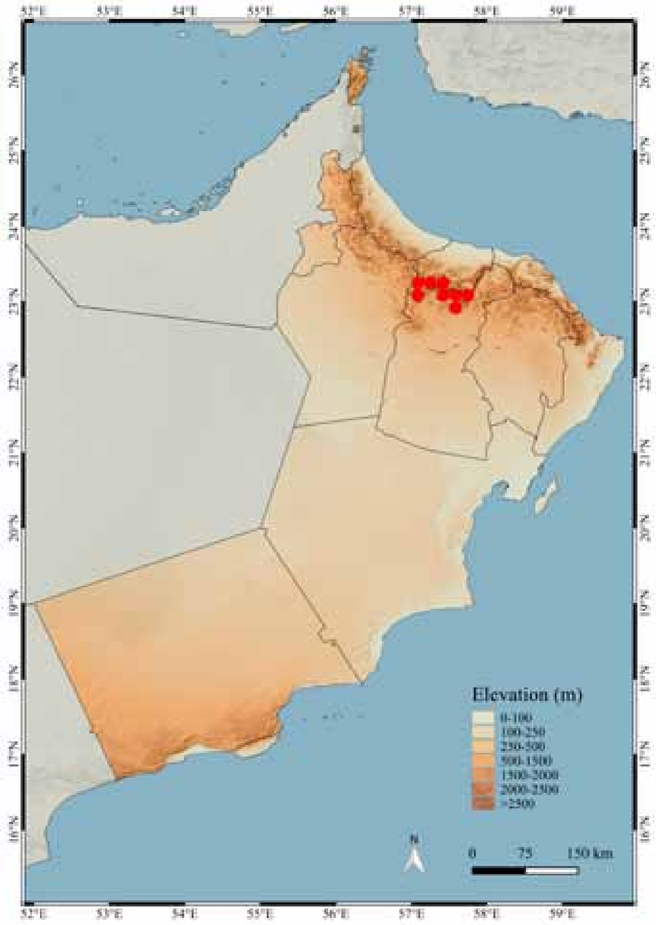

C

Useful information	
IUCN Category	NE
Endemic	YES
Venomous	NO
Insular	NO
Present in a protected area	NO

D

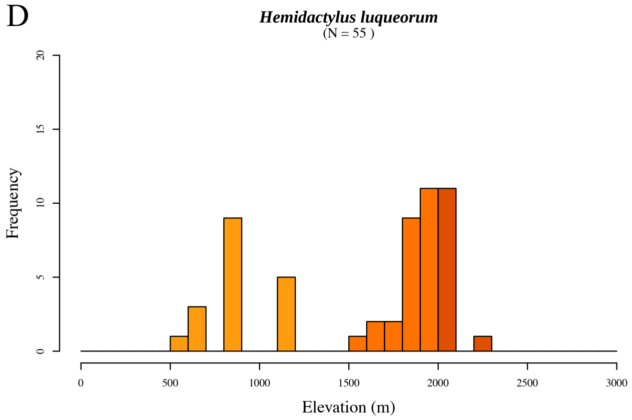

E

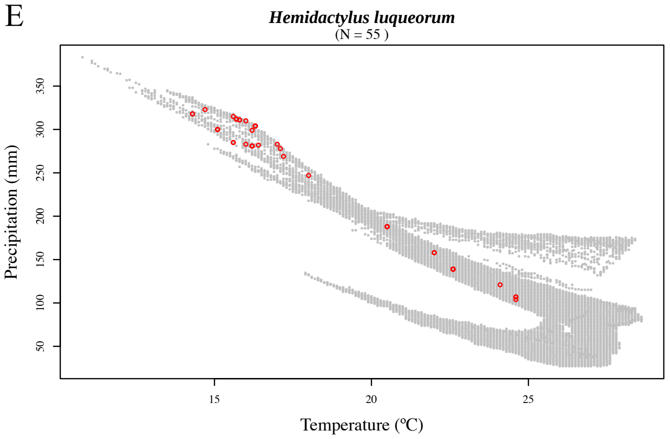

F

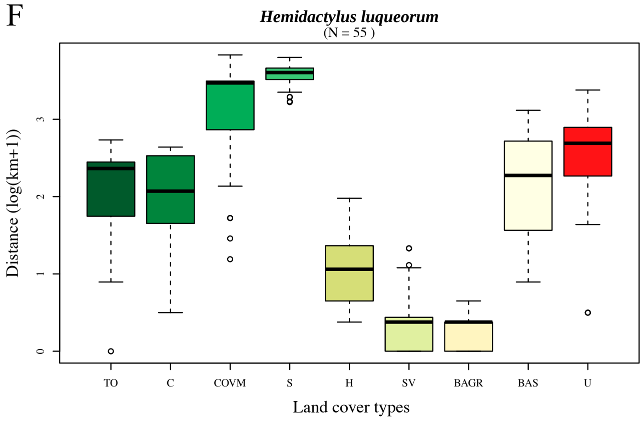

S25: Species information

Lizards

Geckos, Gekkonidae

Hemidactylus masirahensis

Carranza & Arnold, 2012

A

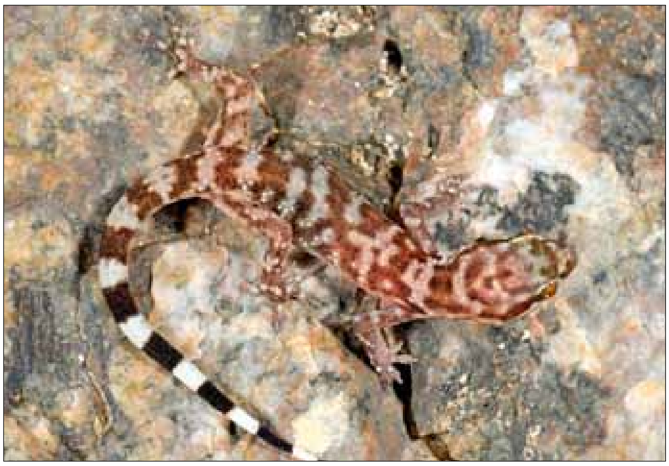

B

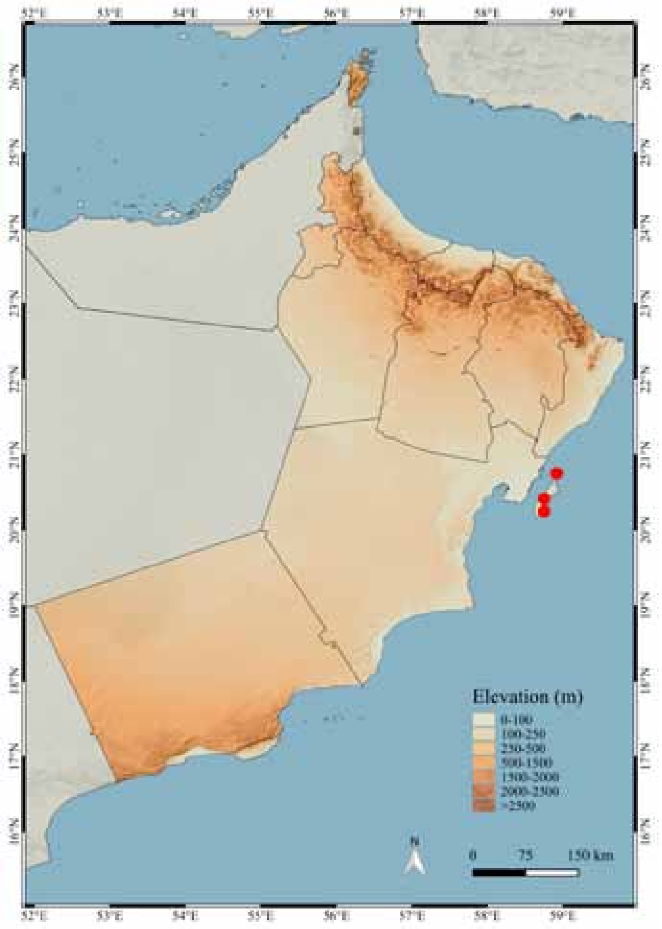

C

Useful information	
IUCN Category	NE
Endemic	YES
Venomous	NO
Insular	YES
Present in a protected area	NO

D

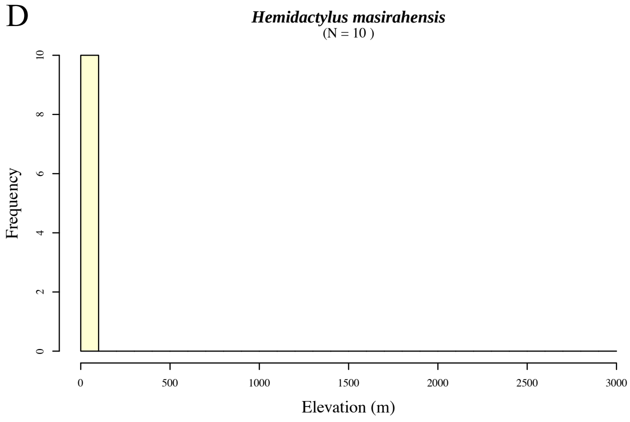

E

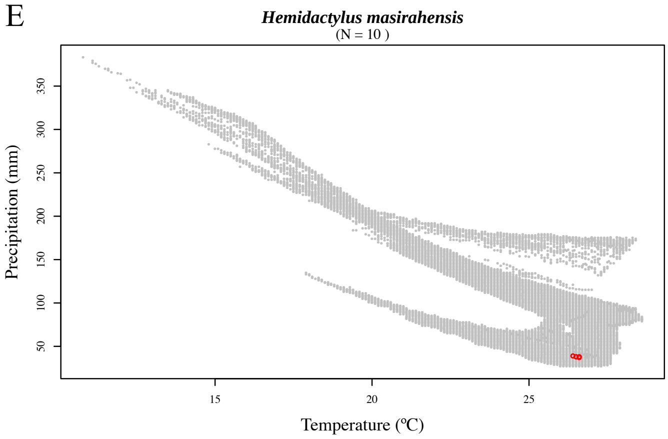

F

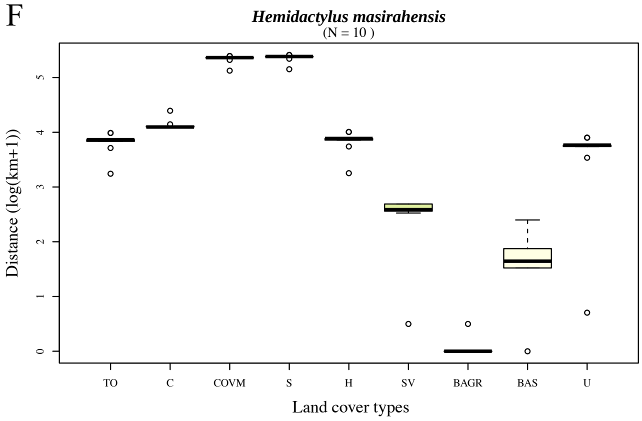

Hemidactylus minutus
Vasconcelos & Carranza, 2014

S26: Species information
Lizards
Geckos, Gekkonidae

A

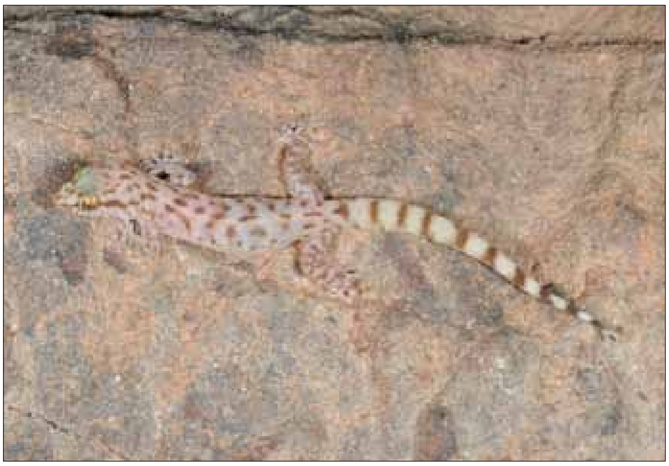

B

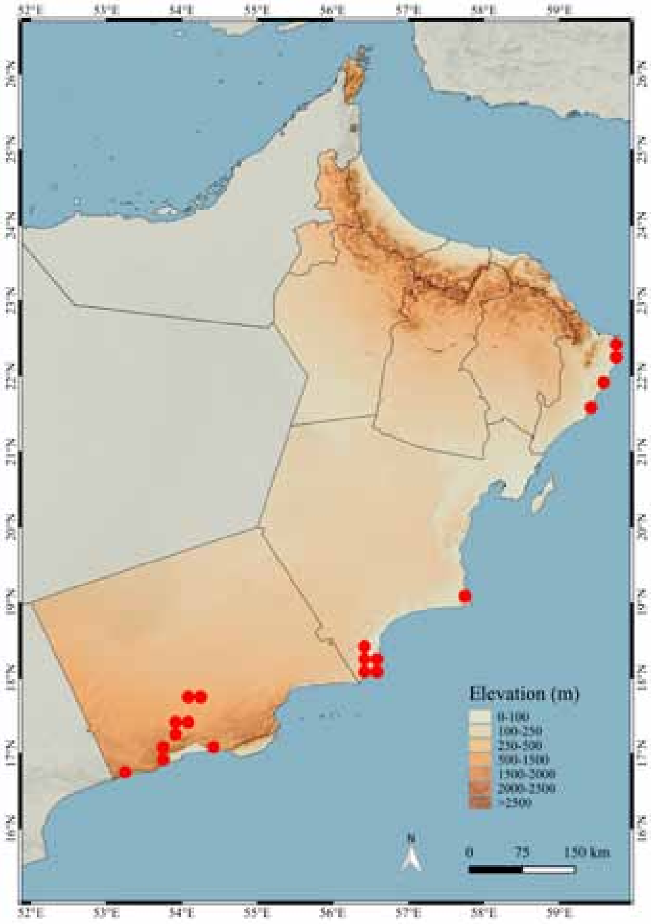

C

Useful information	
IUCN Category	NE
Endemic	NO
Venomous	NO
Insular	NO
Present in a protected area	YES

D

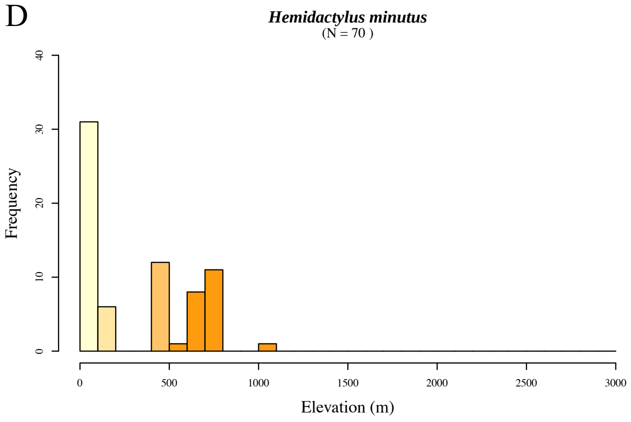

E

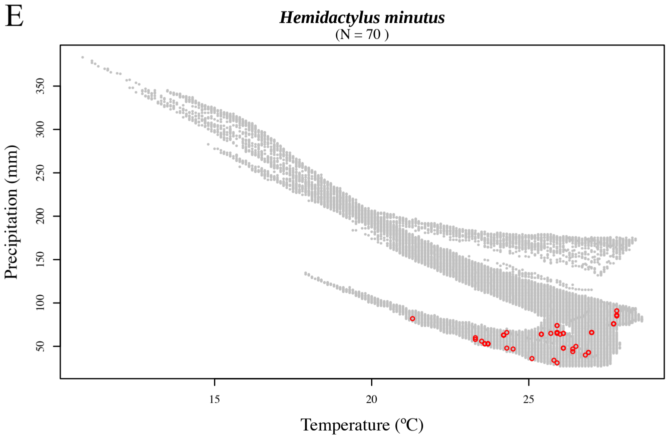

F

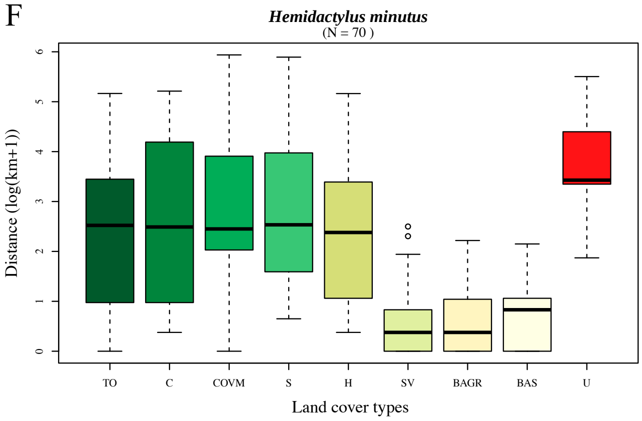

S27: Species information

Lizards

Geckos, Gekkonidae

Hemidactylus paucituberculatus

Carranza & Arnold, 2012

A

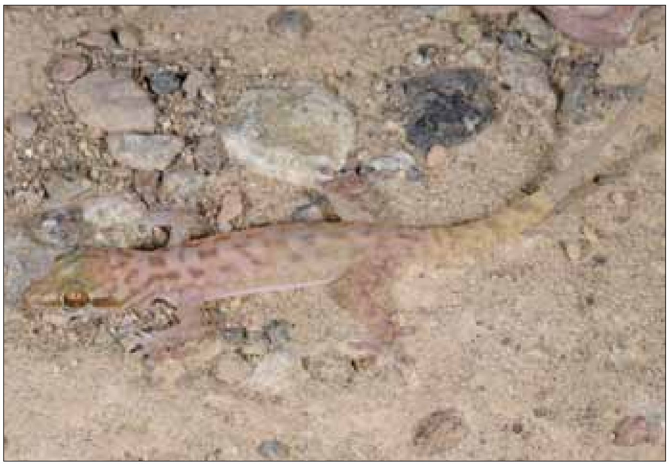

B

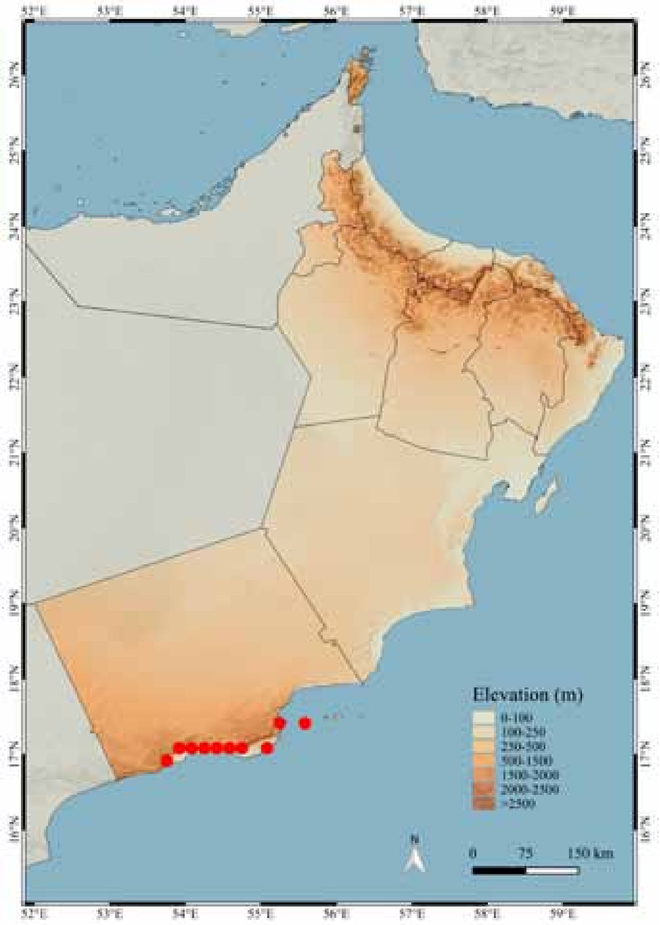

C

Useful information	
IUCN Category	NE
Endemic	YES
Venomous	NO
Insular	YES
Present in a protected area	YES

D

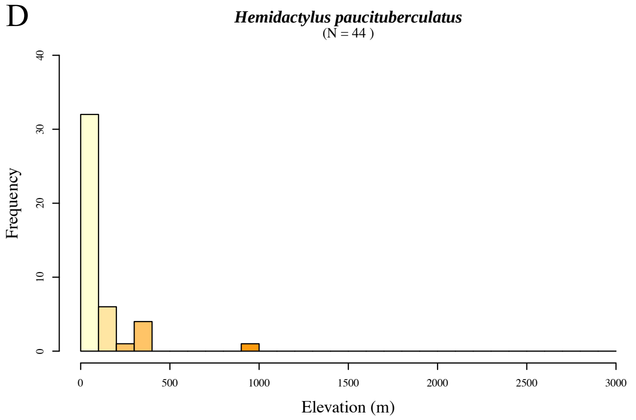

E

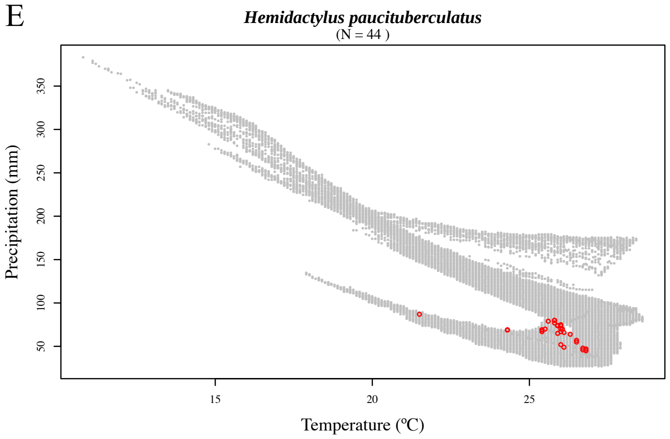

F

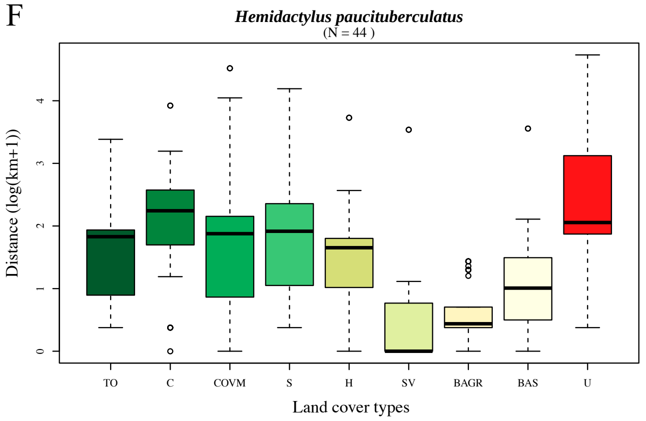

Lizards
Agamids
Geckos
Lacertids
Skinks
Amphisbaenids
Varanids
Snakes
Boidae
Colubridae
Elapidae
Lamprophiidae
Leptotyphlopidae
Typhlopidae
Viperidae

Hemidactylus persicus
Anderson, 1872

S28: Species information
Lizards
Geckos, Gekkonidae

A

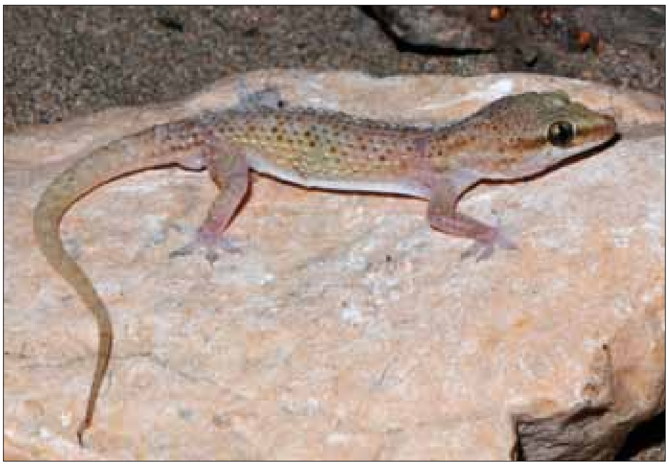

B

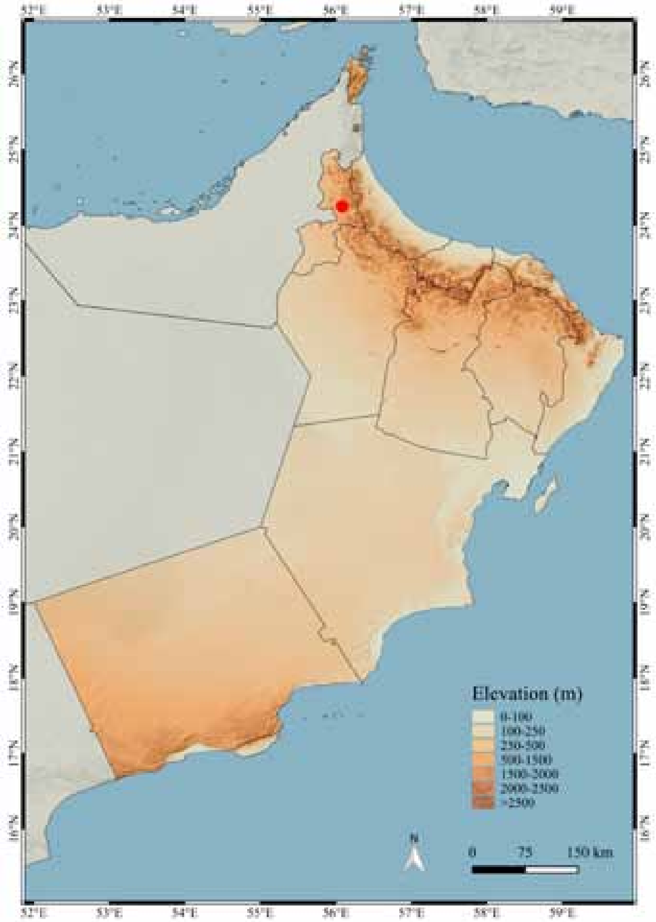

C

Useful information	
IUCN Category	LC*
Endemic	NO
Venomous	NO
Insular	NO
Present in a protected area	NO

*Not available on the web

D

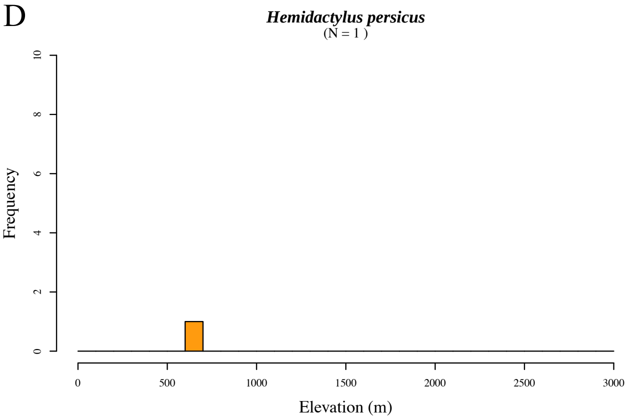

E

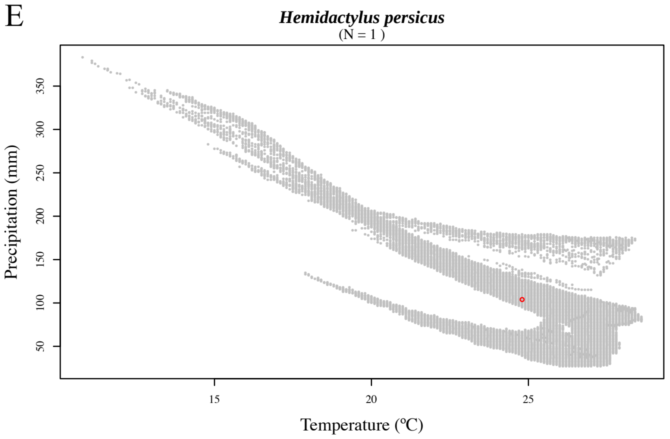

F

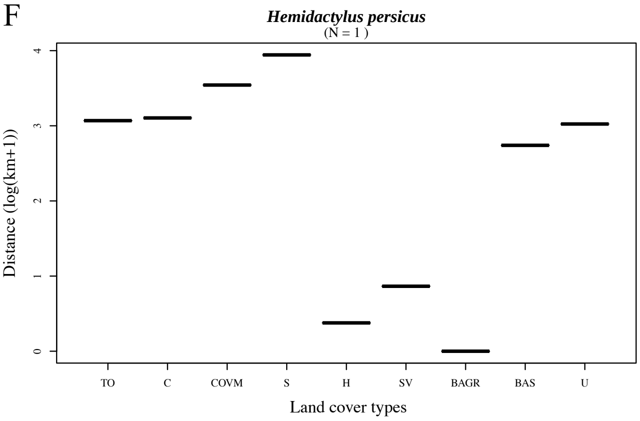

S29: Species information

Lizards

Geckos, Gekkonidae

Hemidactylus robustus
Heyden, 1827

B

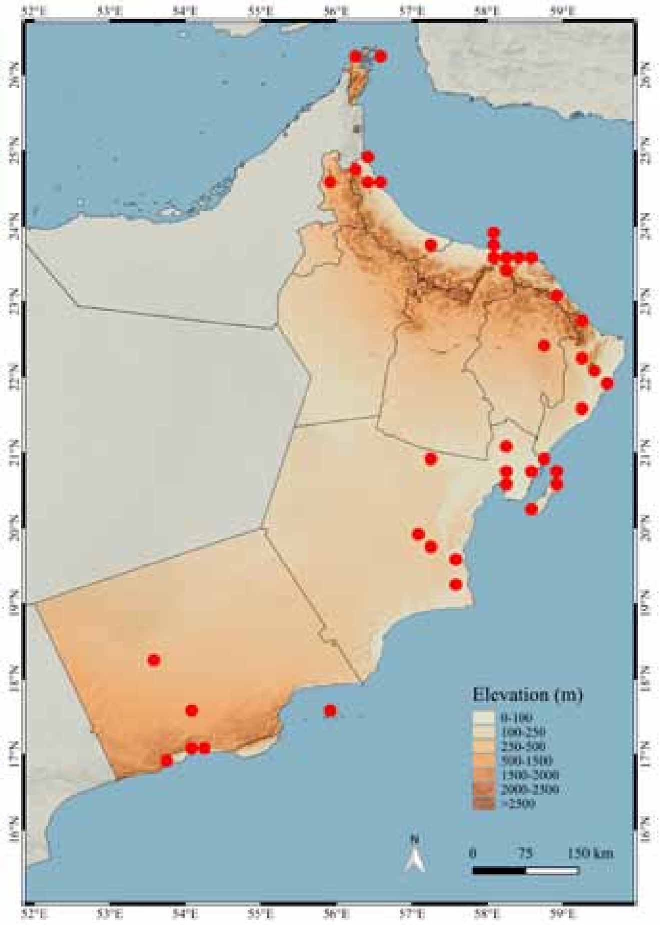

A

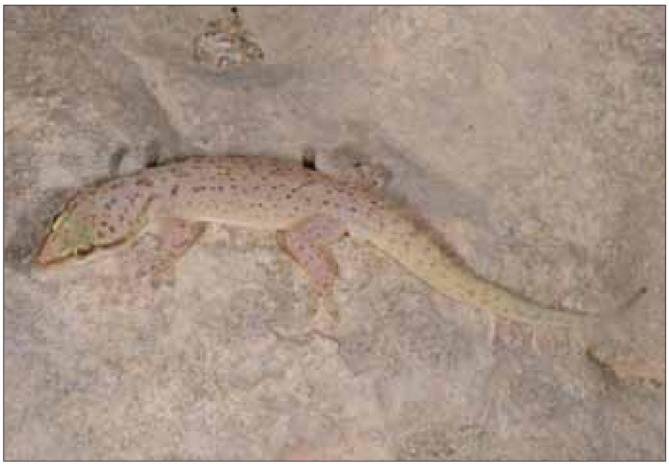

C

Useful information	
IUCN Category	LC*
Endemic	NO
Venomous	NO
Insular	YES
Present in a protected area	YES

*Not available on the web

D

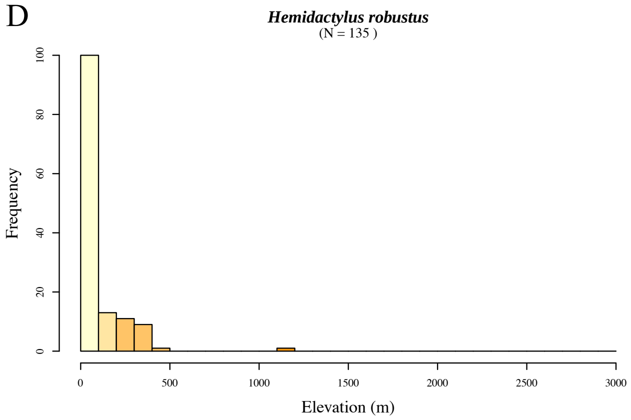

E

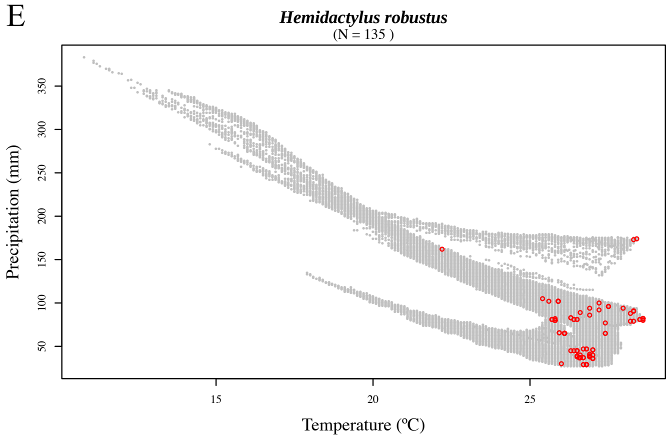

F

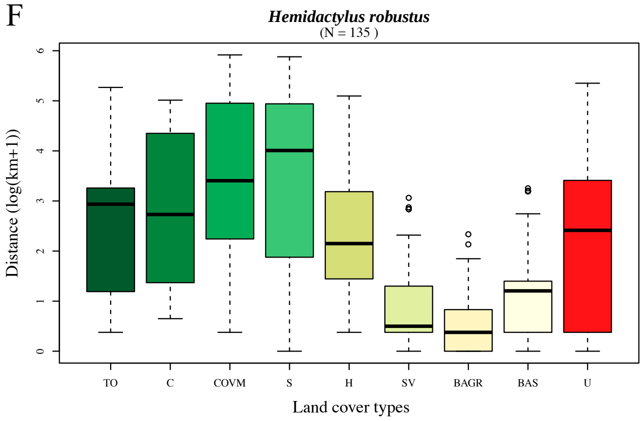

Hemidactylus sp.
Undescribed

S30: Species information
Lizards
Geckos, Gekkonidae

A

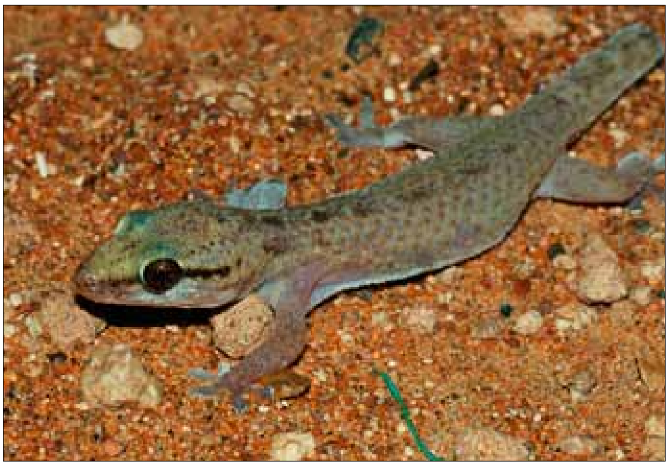

B

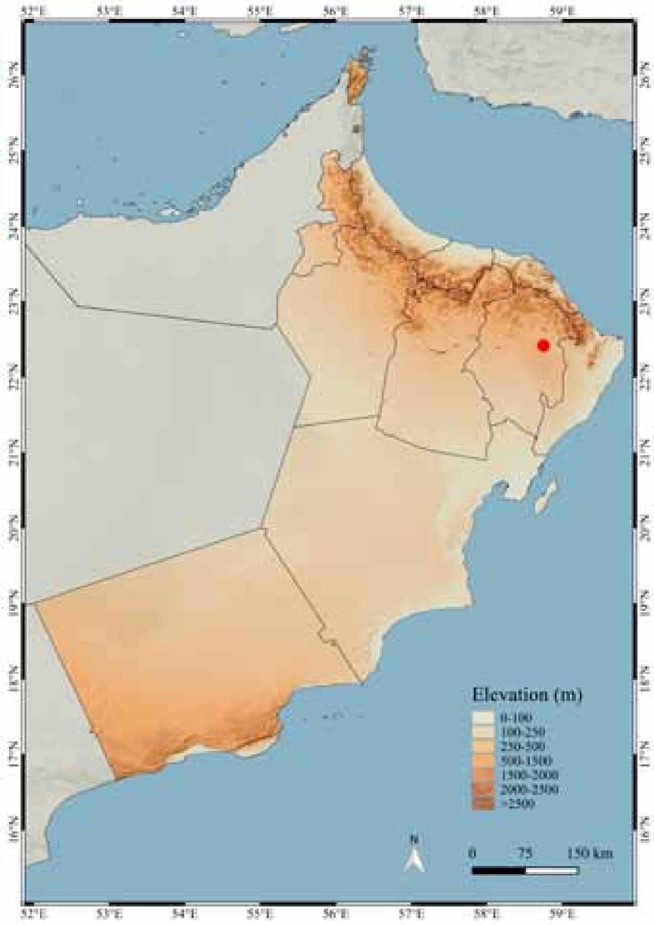

C

Useful information	
IUCN Category	NE
Endemic	YES
Venomous	NO
Insular	NO
Present in a protected area	NO

D

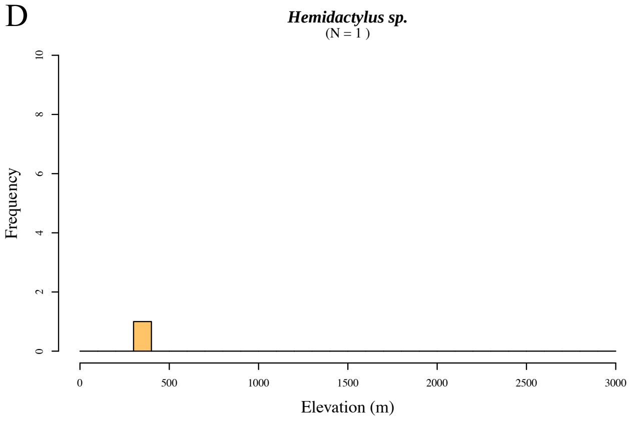

E

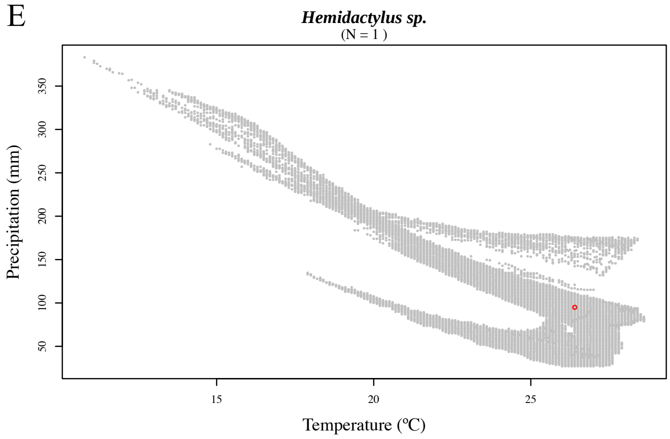

F

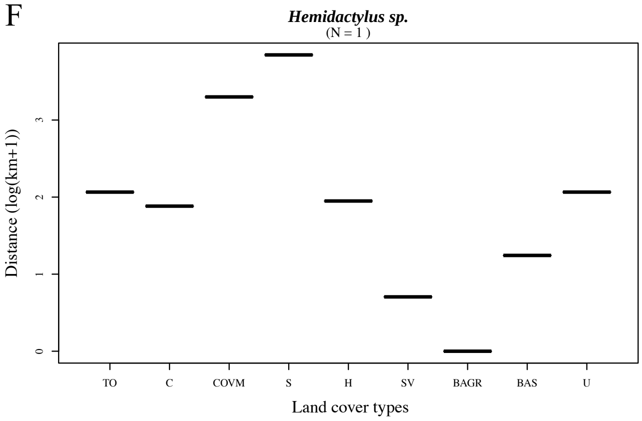

S31: Species information

Lizards

Geckos, Gekkonidae

Pseudoceramodactylus khobarensis

Haas, 1857

A

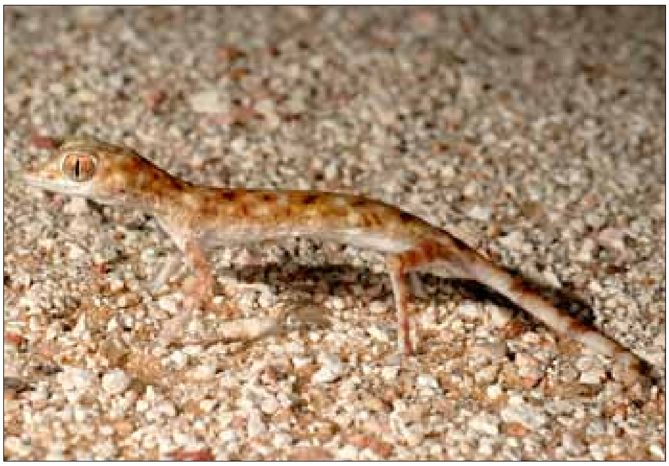

B

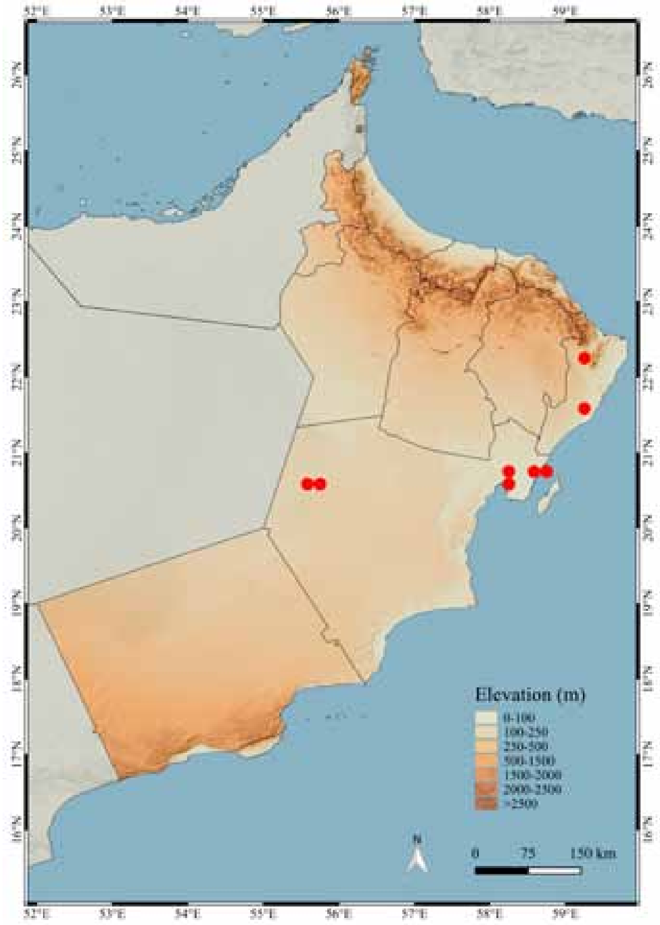

C

Useful information	
IUCN Category	LC
Endemic	NO
Venomous	NO
Insular	NO
Present in a protected area	YES

D

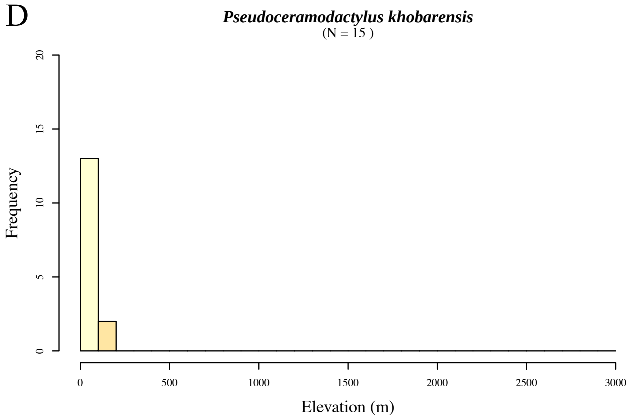

E

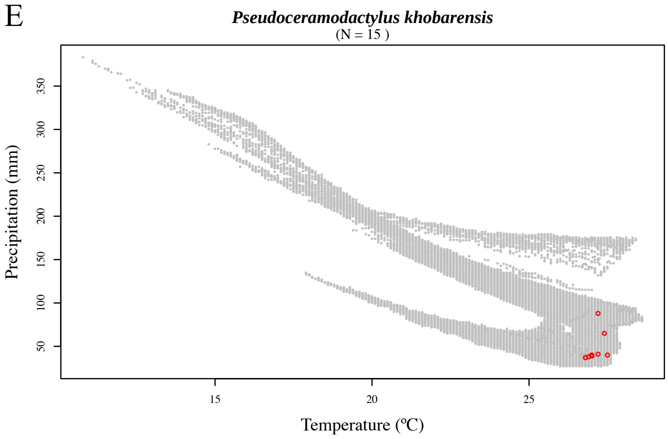

F

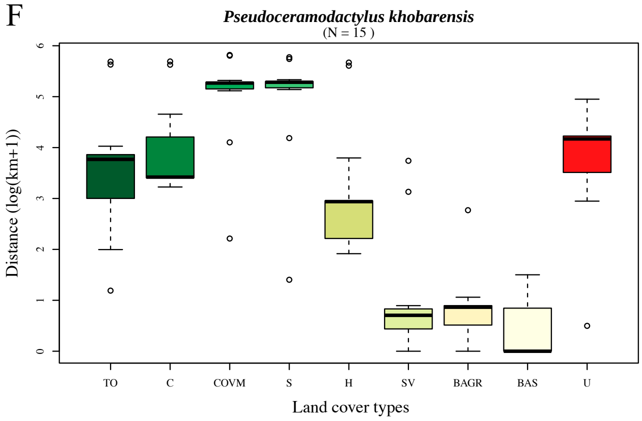

Stenodactylus arabicus
(Haas, 1957)

S32: Species information
Lizards
Geckos, Gekkonidae

A

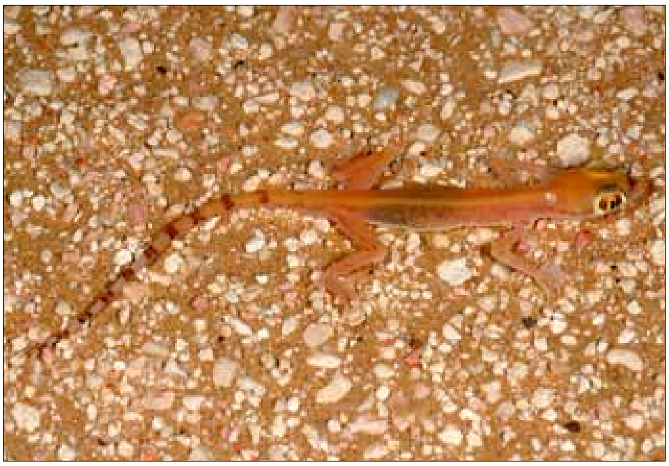

B

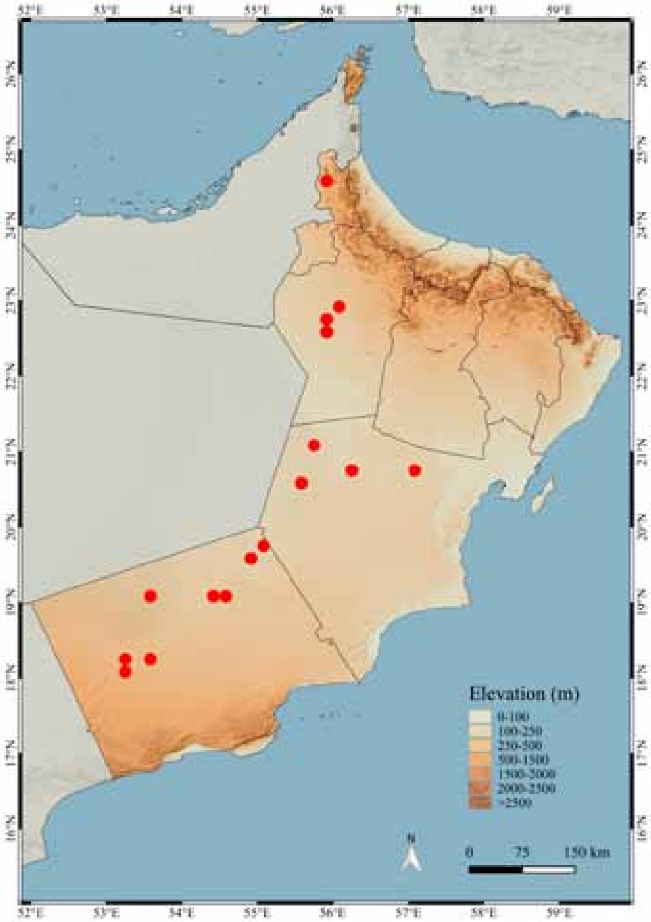

C

Useful information	
IUCN Category	LC
Endemic	NO
Venomous	NO
Insular	NO
Present in a protected area	NO

D

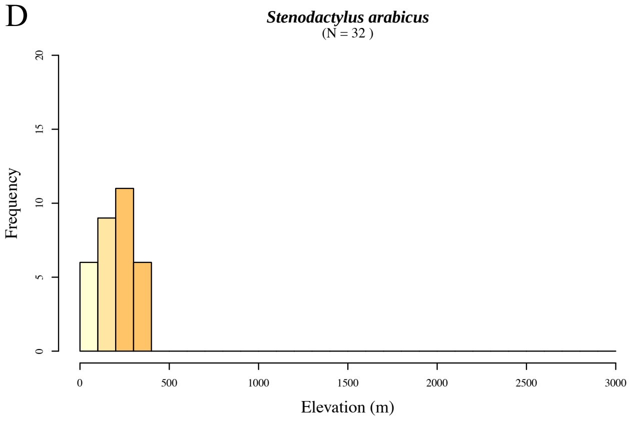

E

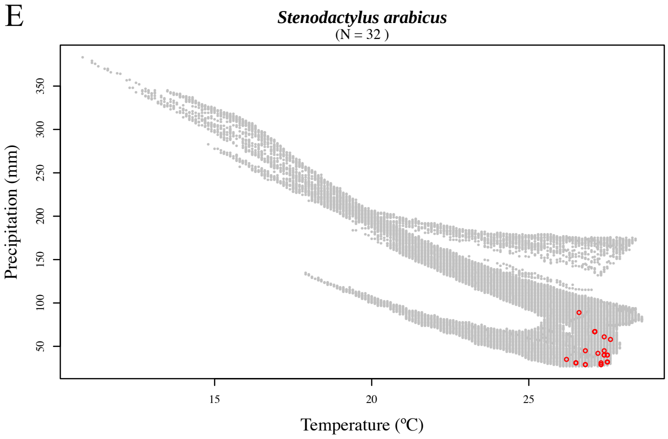

F

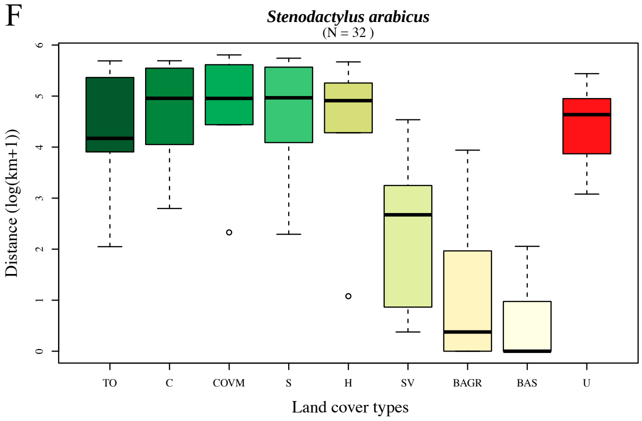

S33: Species information

Lizards

Geckos, Gekkonidae

Stenodactylus doriae
(Blanford, 1874)

A

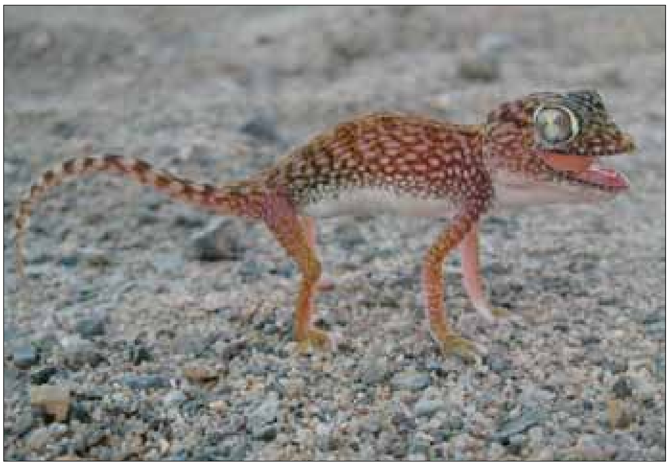

B

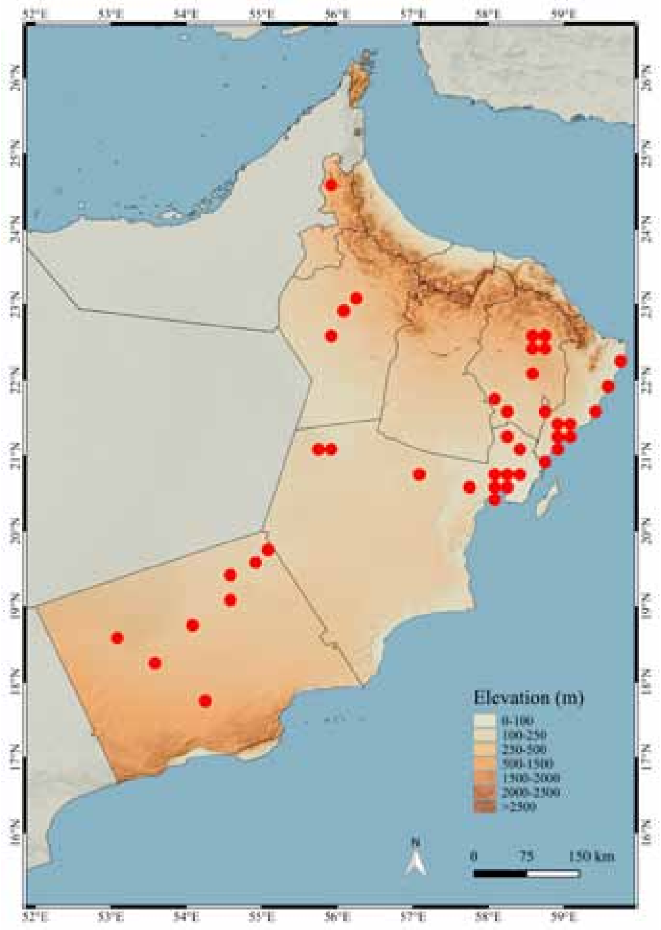

C

Useful information	
IUCN Category	LC
Endemic	NO
Venomous	NO
Insular	NO
Present in a protected area	YES

D

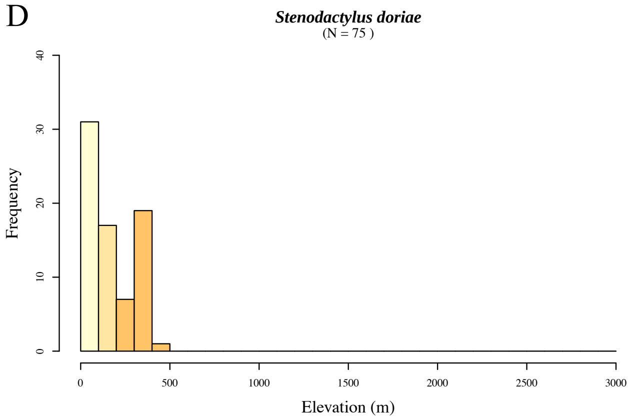

E

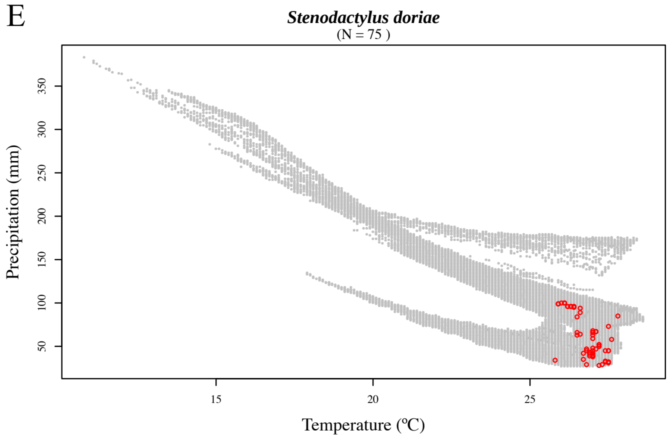

F

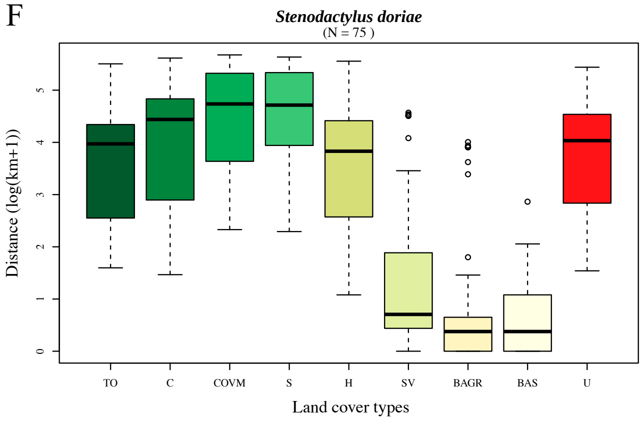

Lizards
Agamids
Geckos
Lacertids
Skinks
Amphisbaenids
Varanids
Snakes
Boidae
Colubridae
Elapidae
Lamprophiidae
Leptotyphlopidae
Typhlopidae
Viperidae

Stenodactylus leptocosymbotes
Leviton & Anderson, 1967

S34: Species information
Lizards
Geckos, Gekkonidae

A

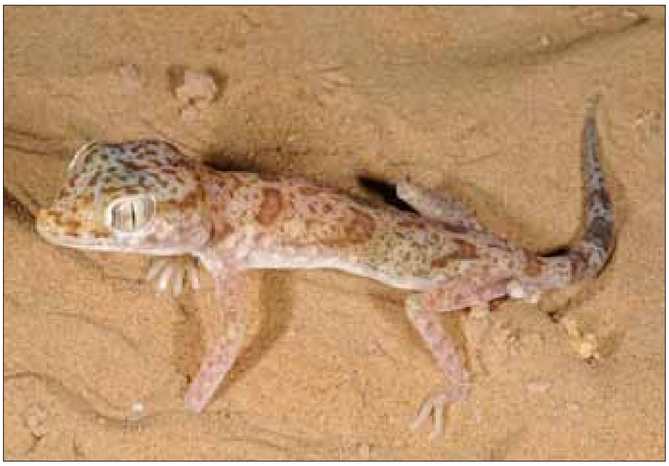

B

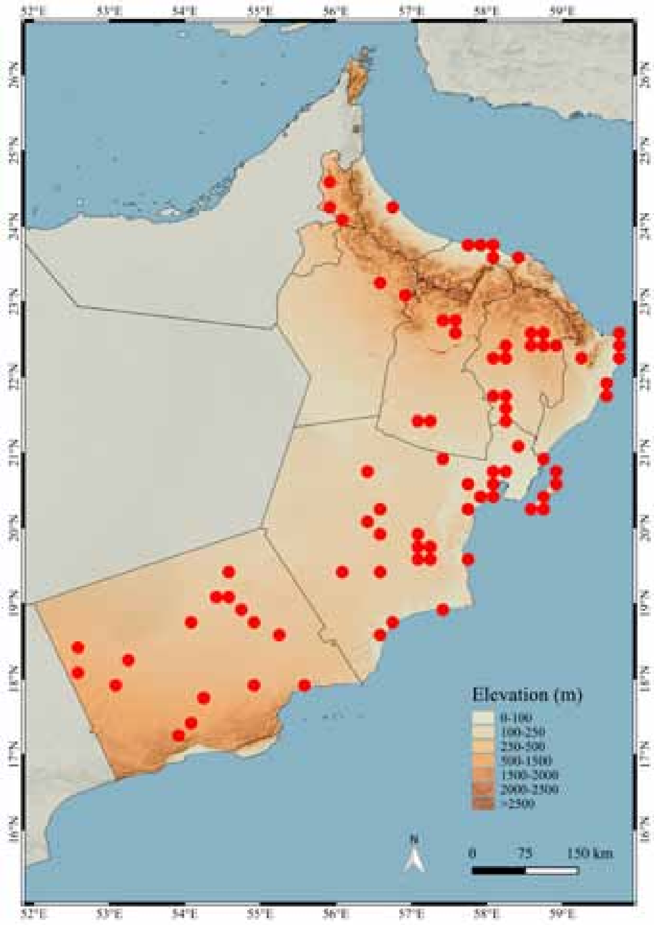

C

Useful information	
IUCN Category	LC*
Endemic	NO
Venomous	NO
Insular	YES
Present in a protected area	YES

*Not available on the web

D

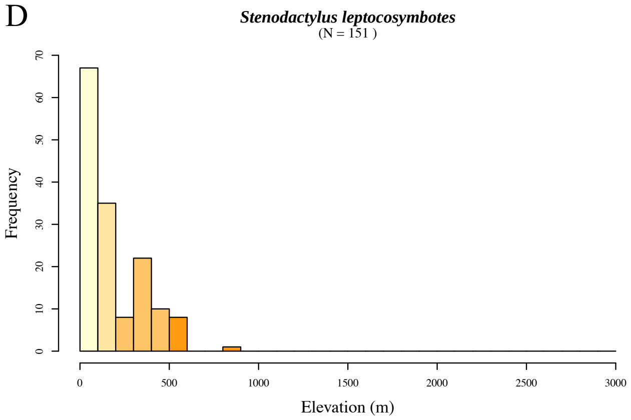

E

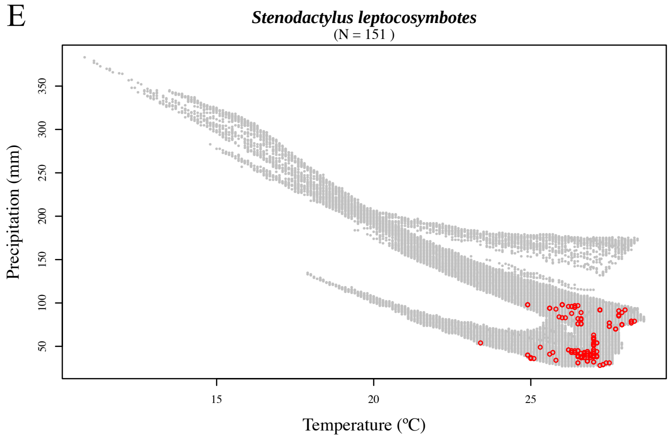

F

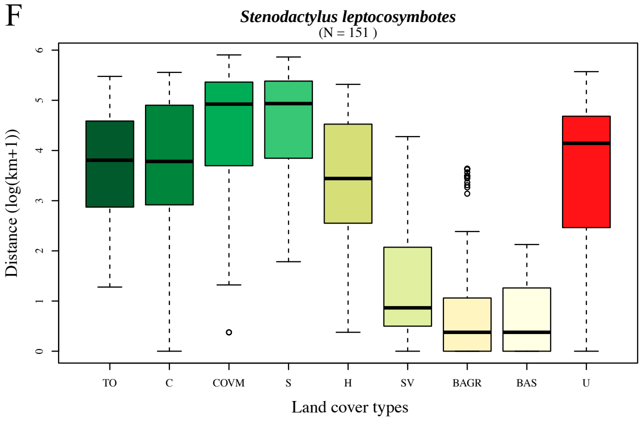

S35: Species information

Lizards

Geckos, Gekkonidae

Stenodactylus sharqiyahensis
Metallinou & Carranza, 2013

A

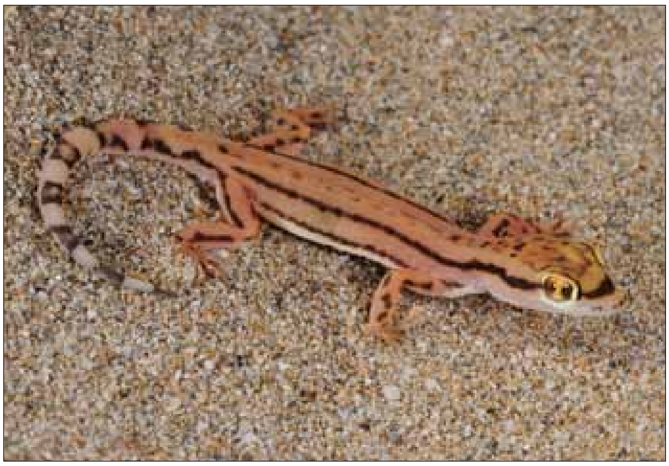

B

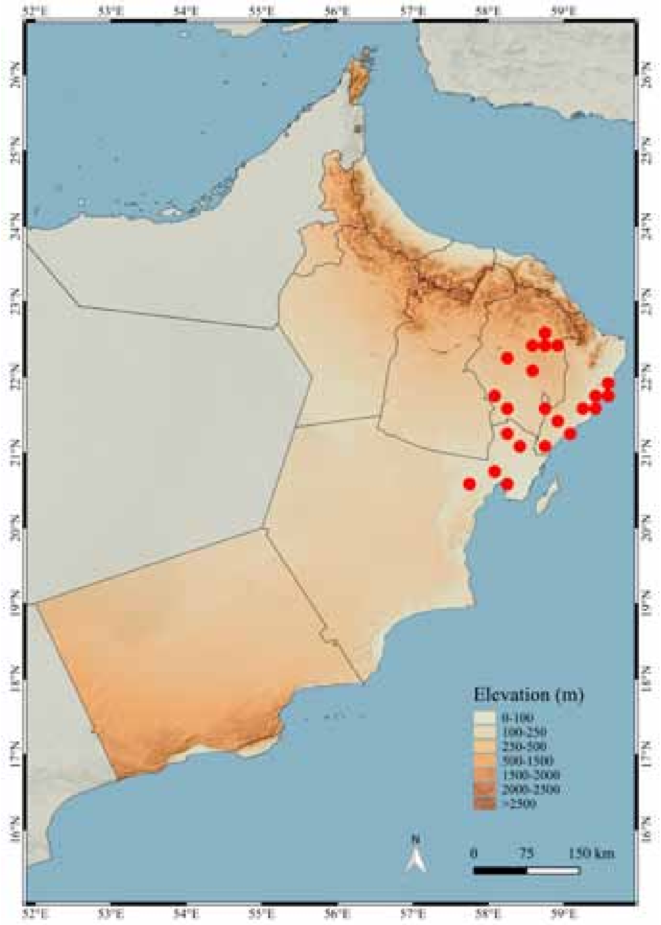

C

Useful information	
IUCN Category	NE
Endemic	YES
Venomous	NO
Insular	NO
Present in a protected area	YES

D

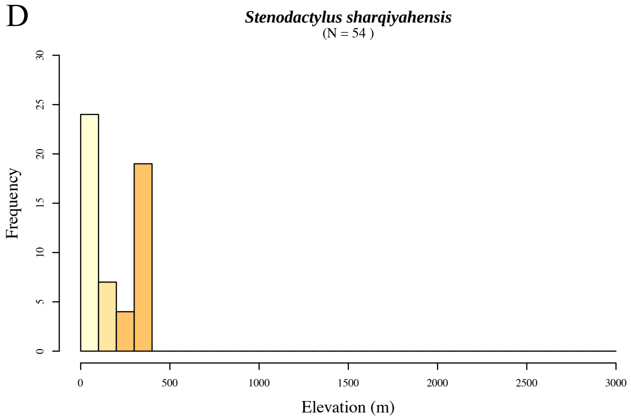

E

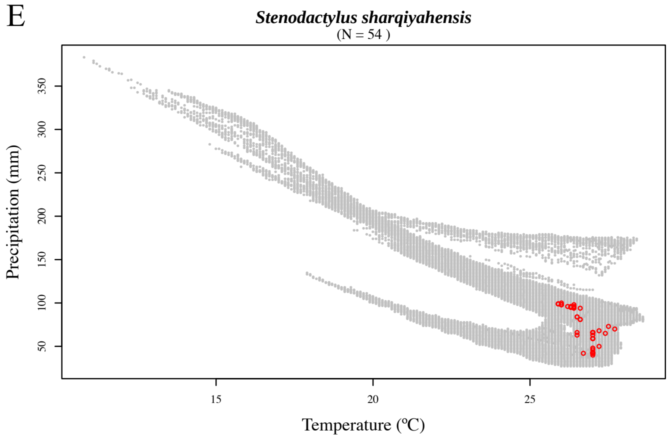

F

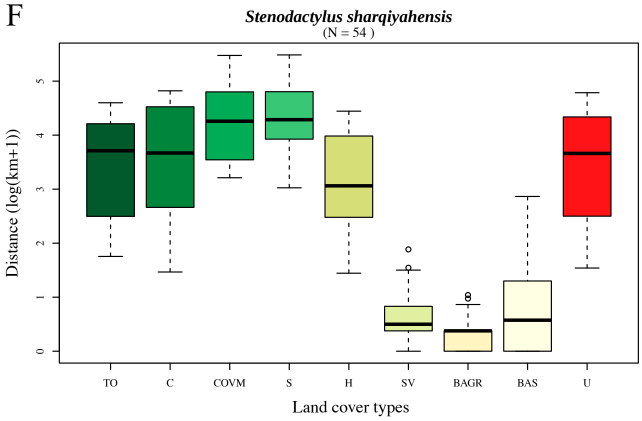

Trachydactylus hajarensis
(Arnold, 1980)

S36: Species information
Lizards
Geckos, Gekkonidae

A

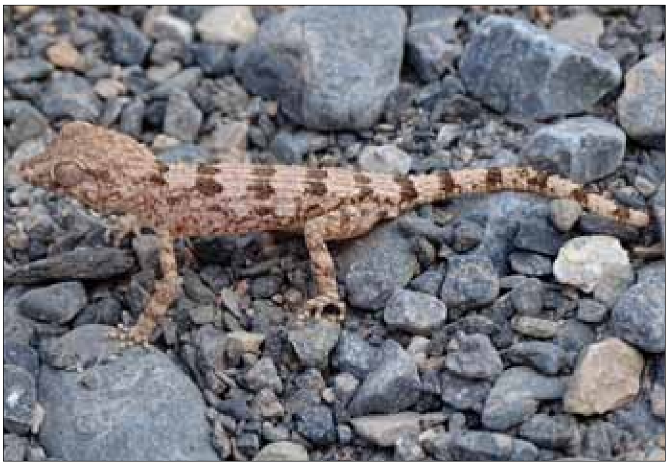

B

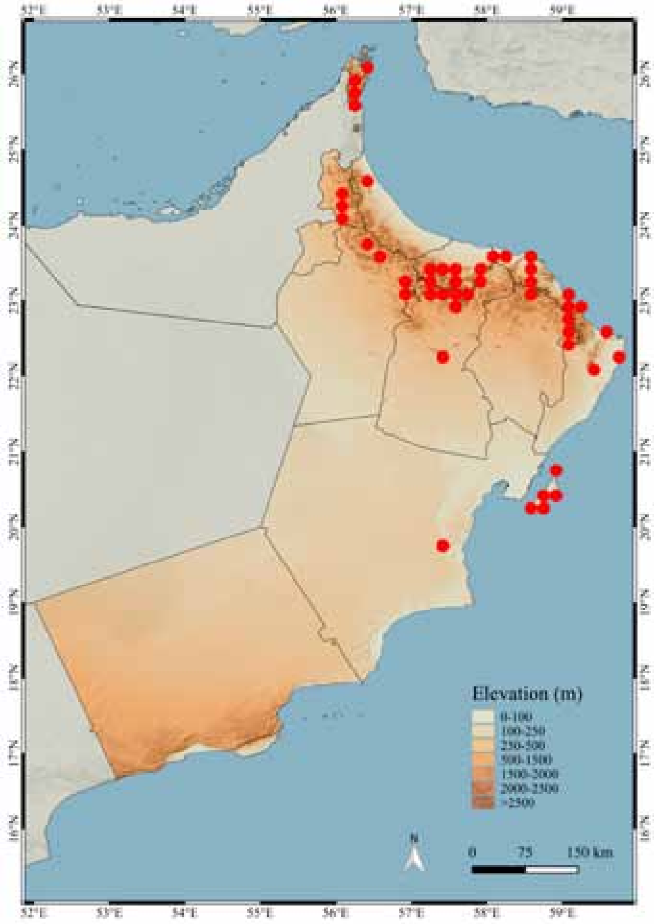

C

Useful information	
IUCN Category	NE
Endemic	NO
Venomous	NO
Insular	YES
Present in a protected area	YES

D

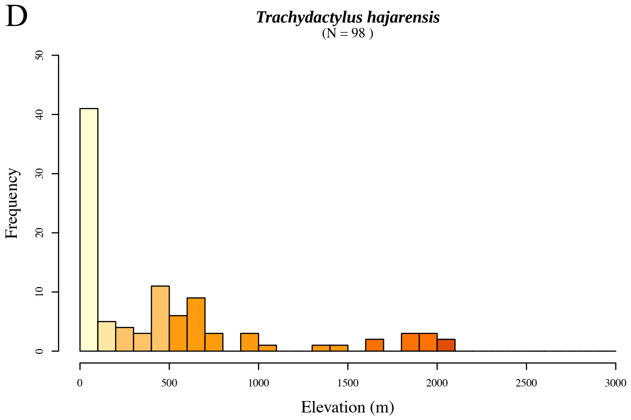

E

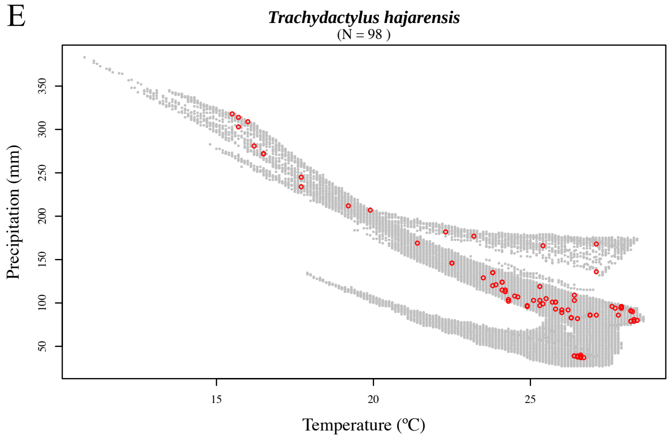

F

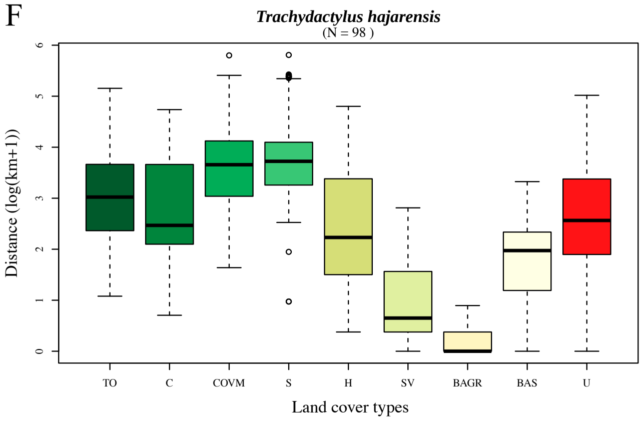

S37: Species information

Lizards

Geckos, Gekkonidae

Trachydactylus spatalurus
(Anderson, 1901)

A

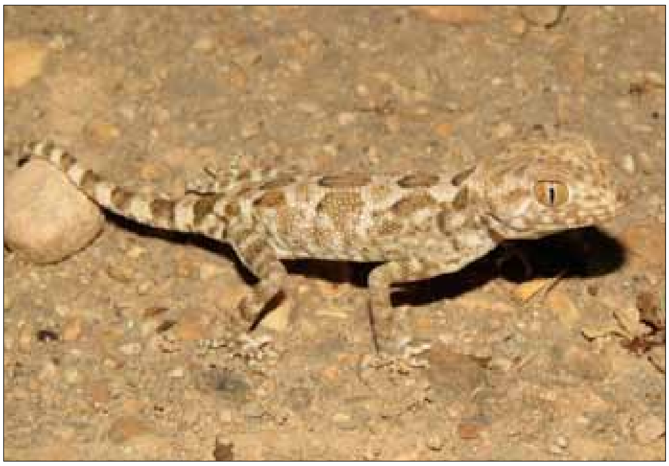

B

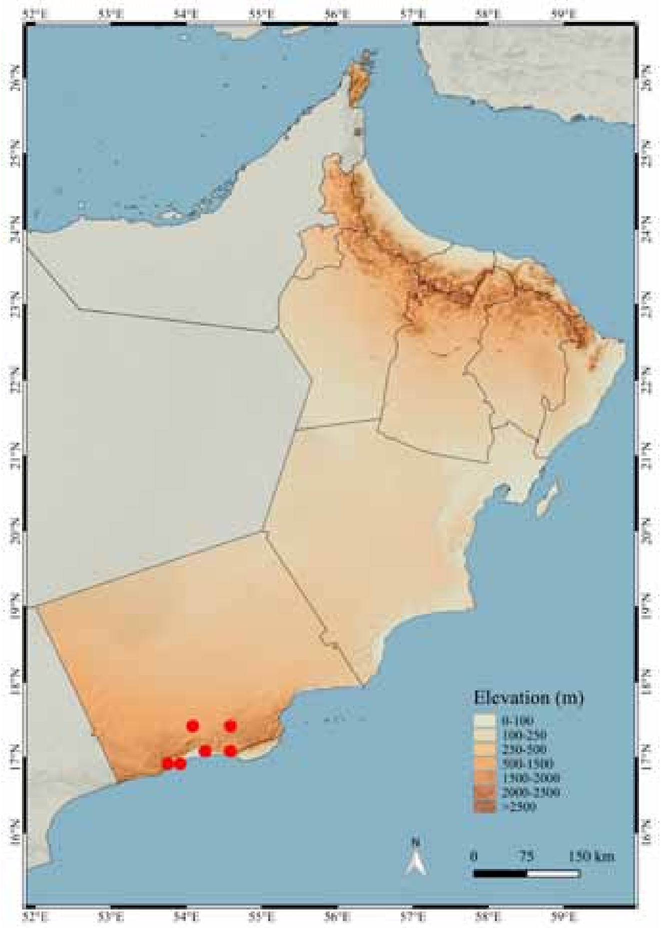

C

Useful information	
IUCN Category	NE
Endemic	NO
Venomous	NO
Insular	NO
Present in a protected area	YES

D

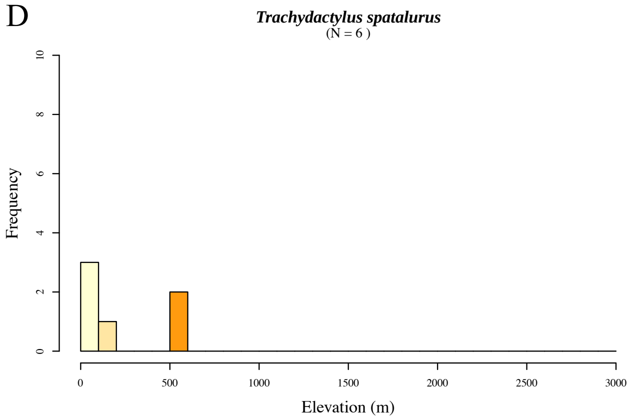

E

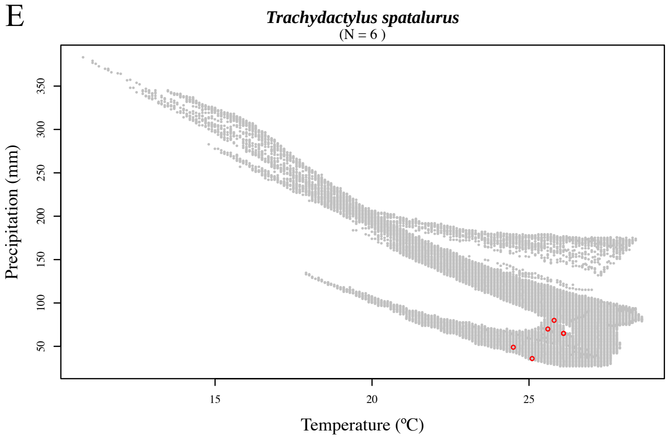

F

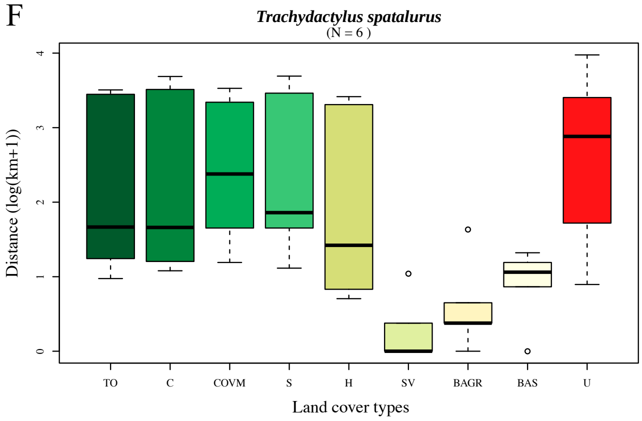

Tropiocolotes scortecci
Cherchi & Spano, 1963

S38: Species information
Lizards
Geckos, Gekkonidae

A

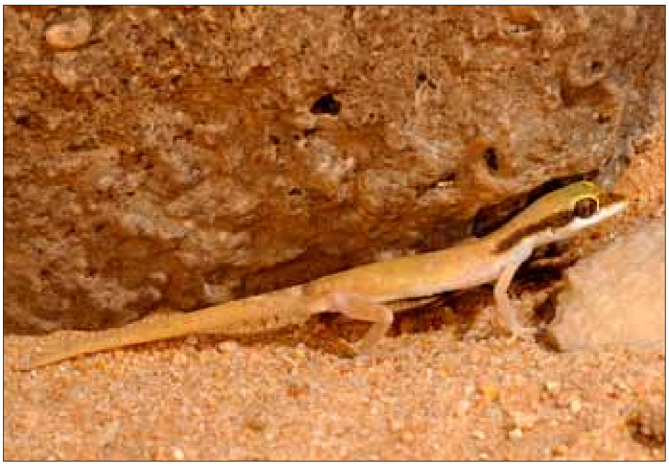

B

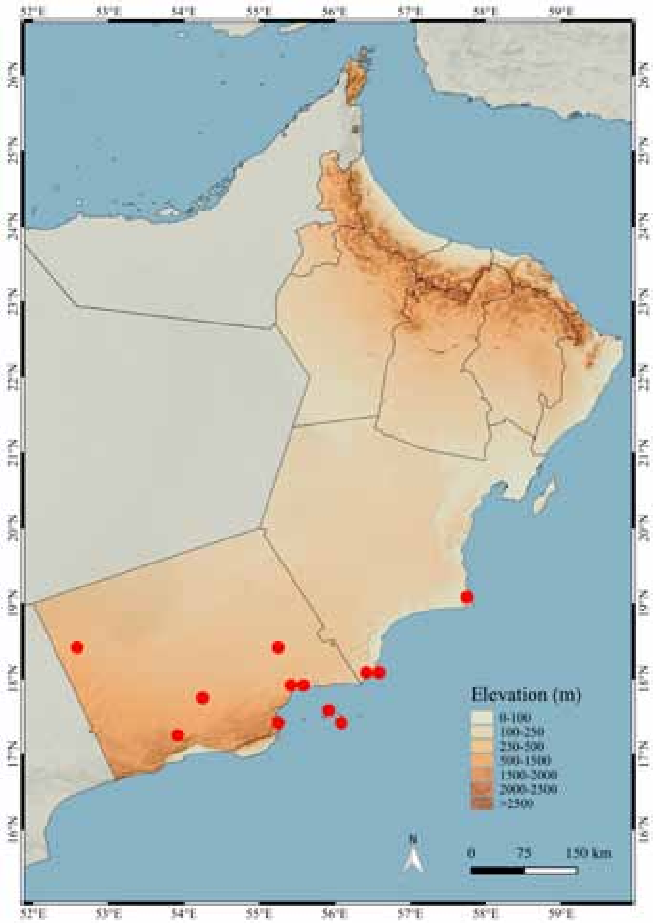

C

Useful information	
IUCN Category	LC
Endemic	NO
Venomous	NO
Insular	YES
Present in a protected area	YES

D

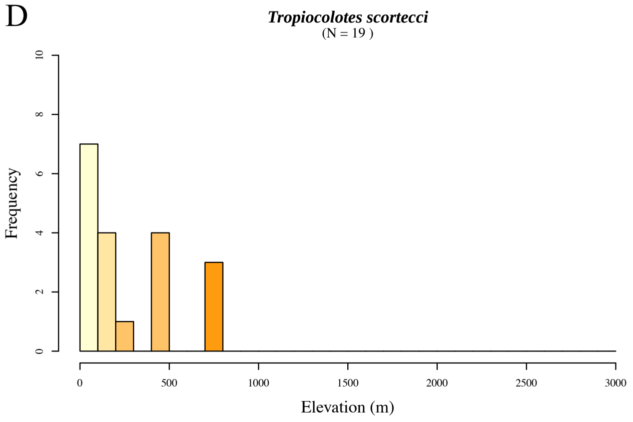

E

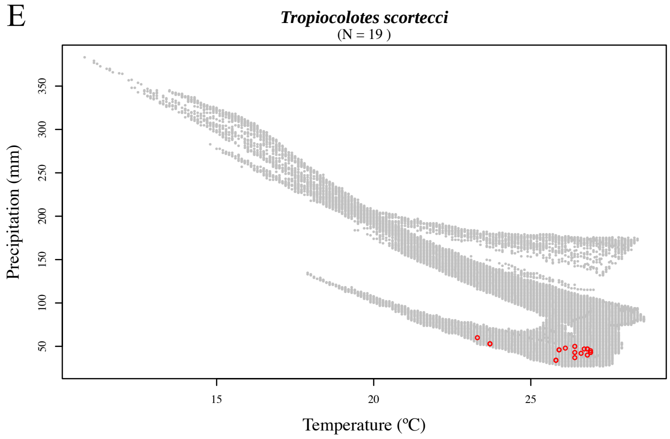

F

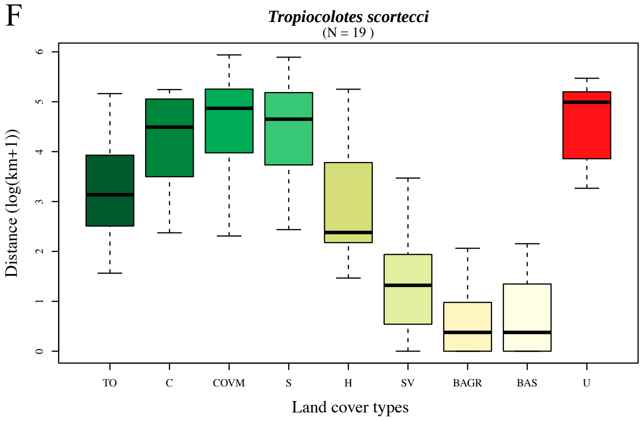

S39: Species information

Lizards

Geckos, Gekkonidae

***Tropiocolotes* sp.**
Undescribed

B

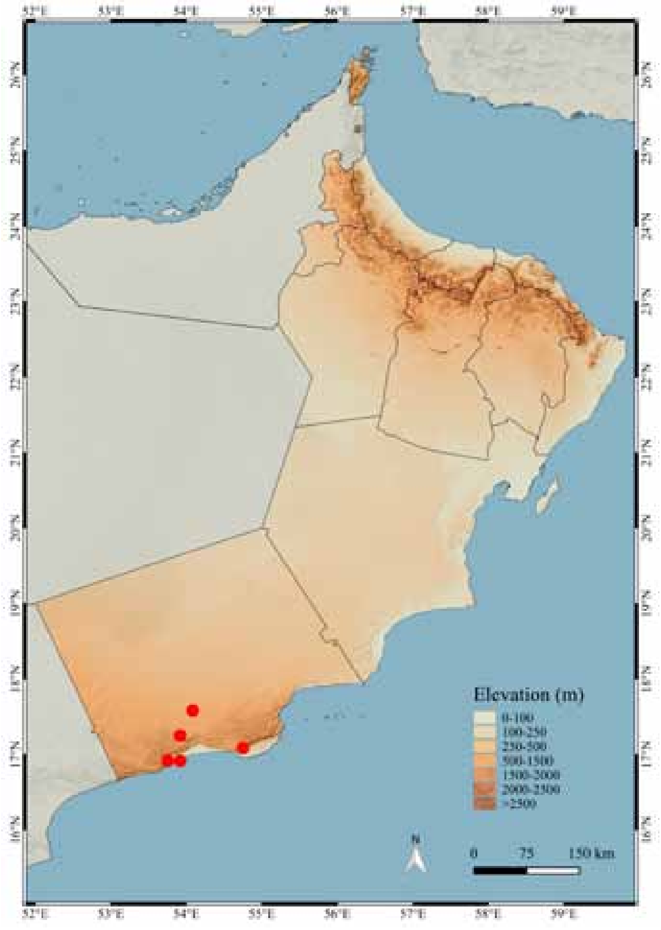

A

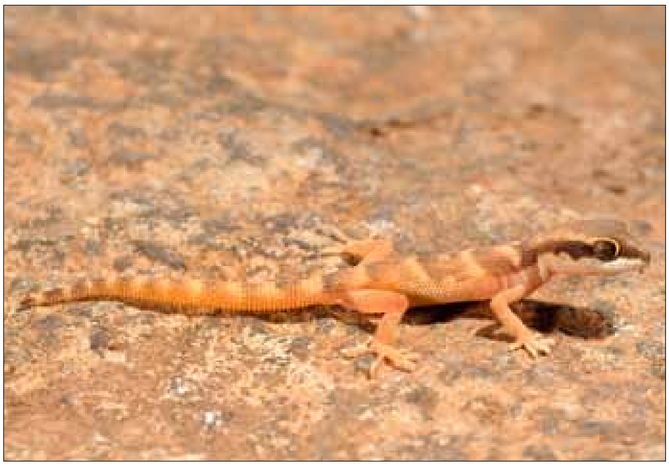

C

Useful information	
IUCN Category	NE
Endemic	YES
Venomous	NO
Insular	NO
Present in a protected area	NO

D

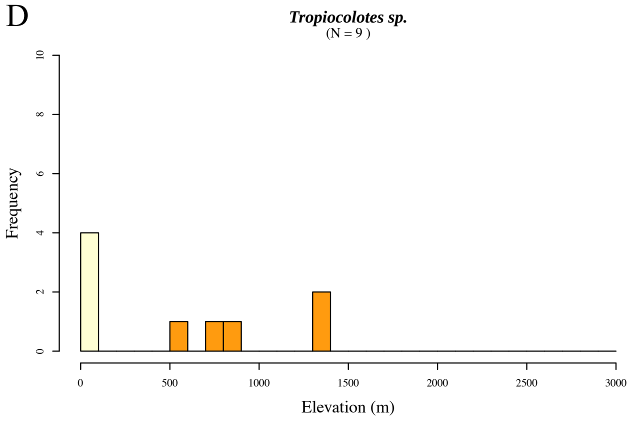

E

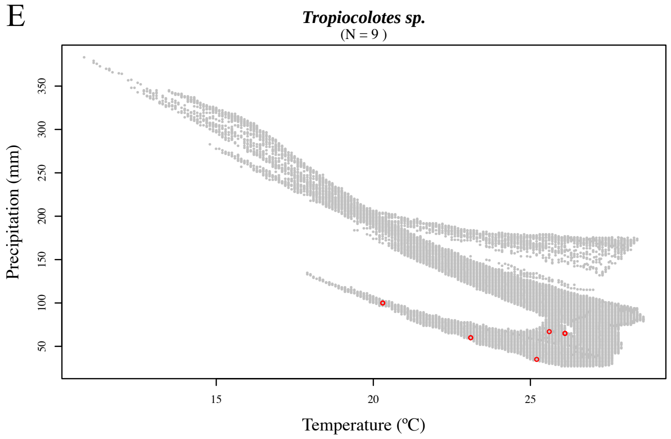

F

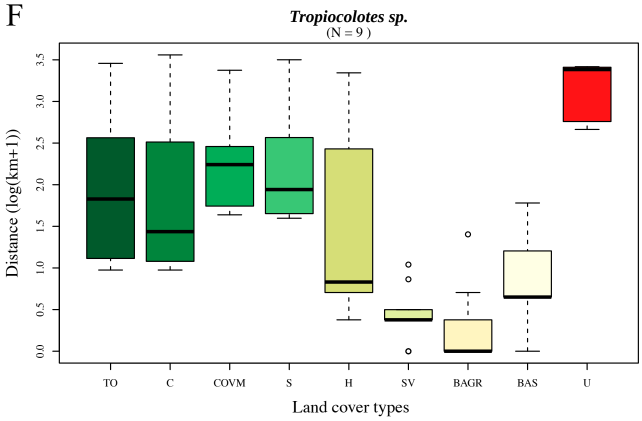

Asaccus gallagheri
(Arnold, 1972)

S40: Species information
Lizards
Geckos, Phyllodactylidae

A

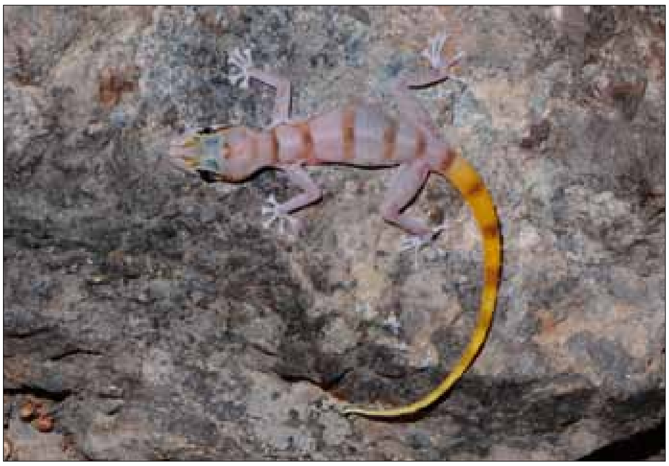

B

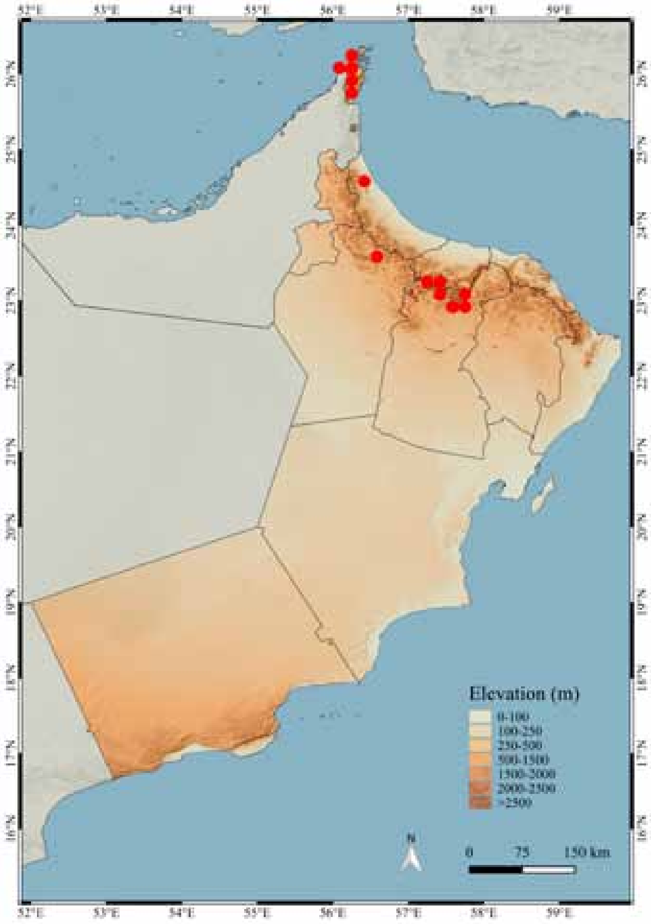

C

Useful information	
IUCN Category	NE
Endemic	NO
Venomous	NO
Insular	NO
Present in a protected area	NO

D

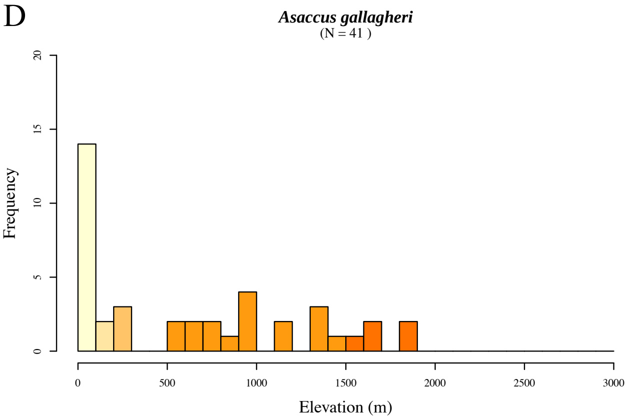

E

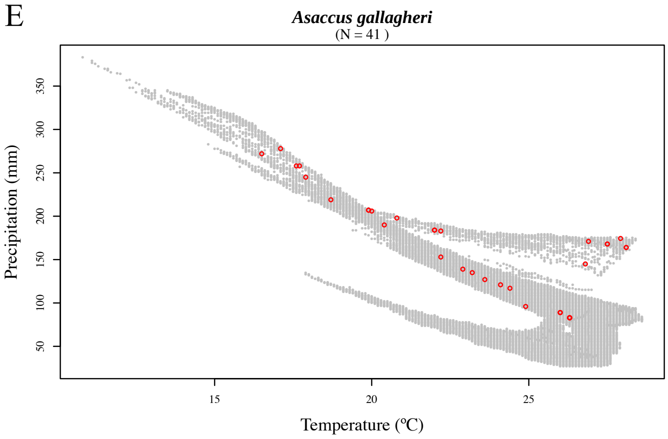

F

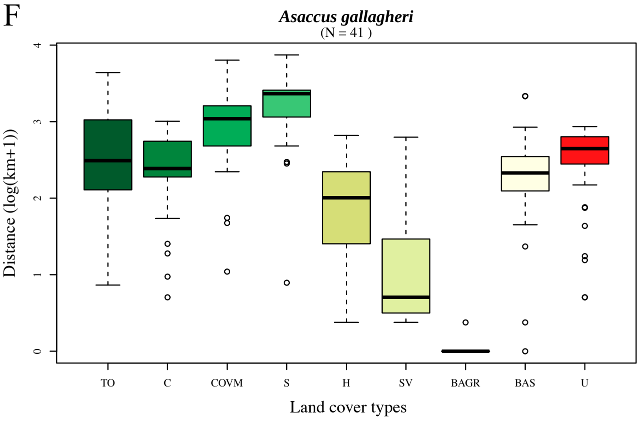

S41: Species information

Lizards

Geckos, Phyllodactylidae

Asaccus gardneri

Carranza, Simó-Riudalbas, Jayasinghe, Wilms & Els, 2016

A

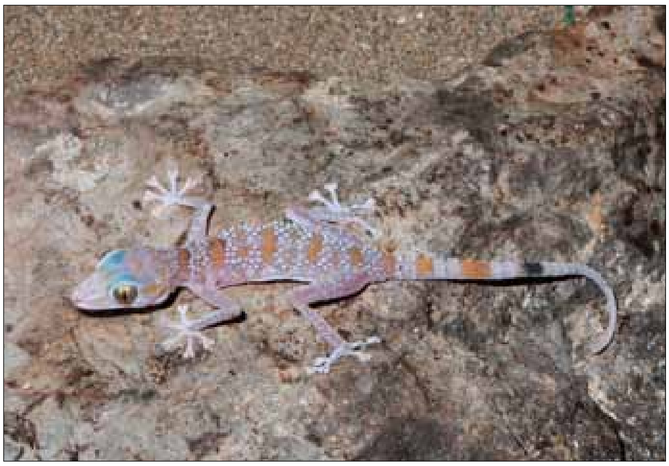

B

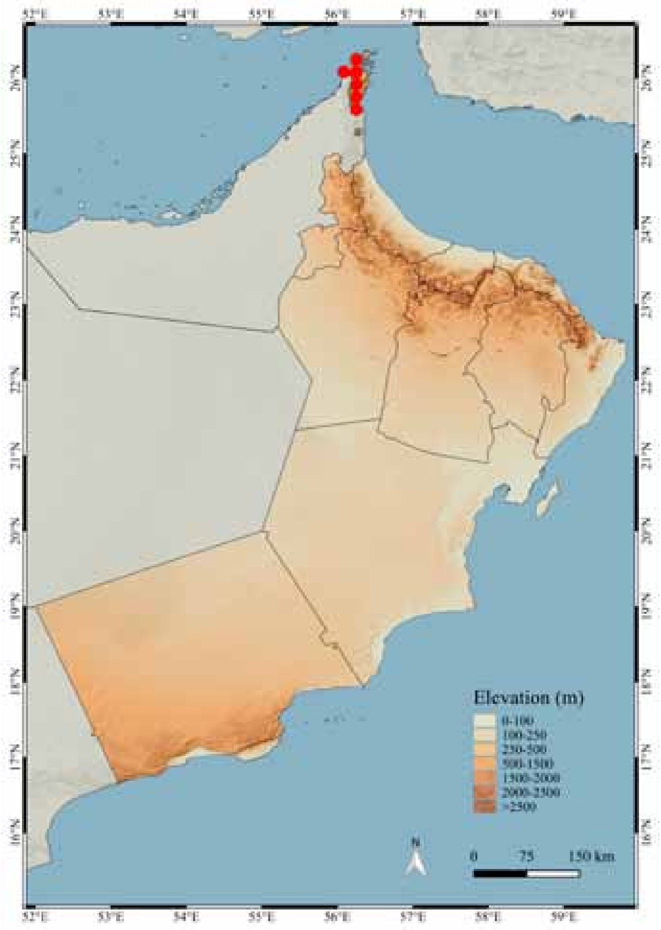

C

Useful information	
IUCN Category	NE
Endemic	NO
Venomous	NO
Insular	NO
Present in a protected area	NO

D

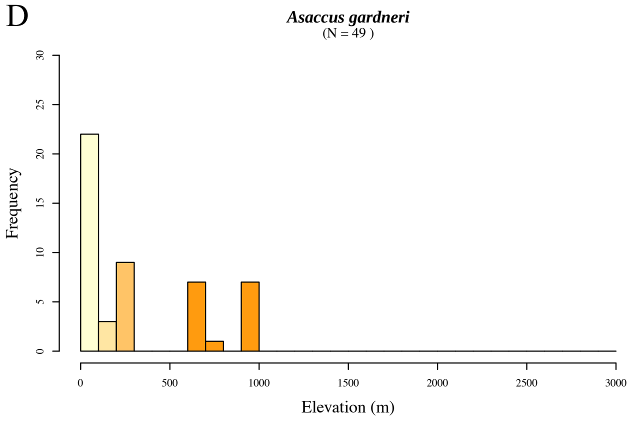

E

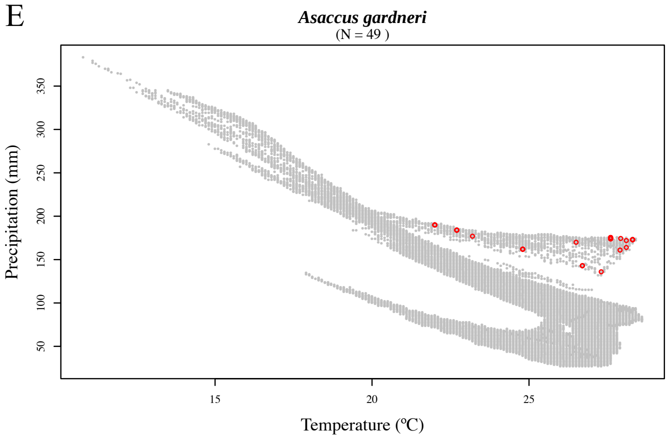

F

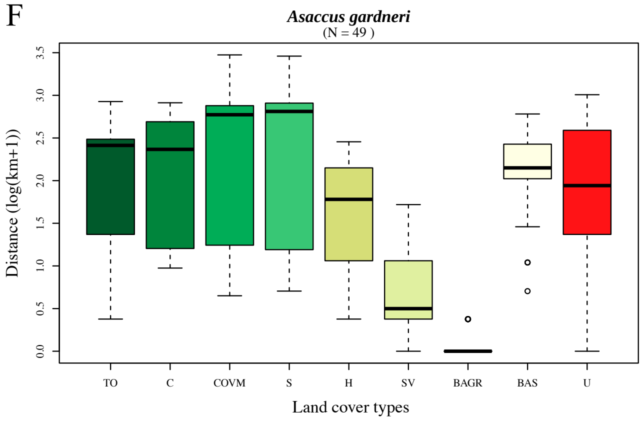

Lizards
Agamids
Geckos
Lacertids
Skinks
Amphisbaenids
Varanids
Snakes
Boidae
Colubridae
Elapidae
Lamprophiidae
Leptotyphlopidae
Typhlopidae
Viperidae

Asaccus margaritae
Carranza, Simó-Riudalbas, Jayasinghe, Wilms & Els, 2016

S42: Species information
Lizards
Geckos, Phyllodactylidae

A

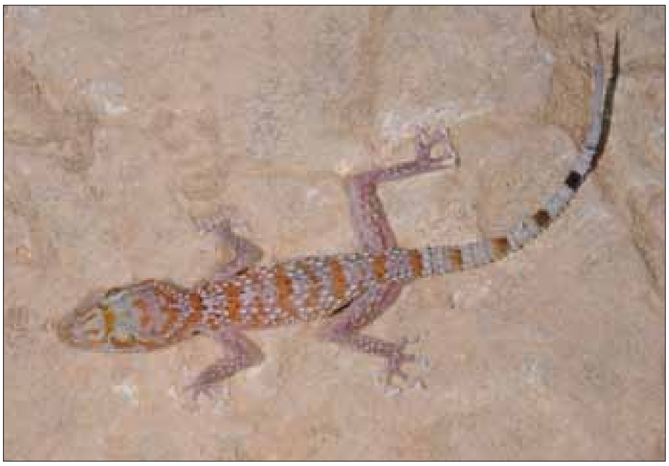

B

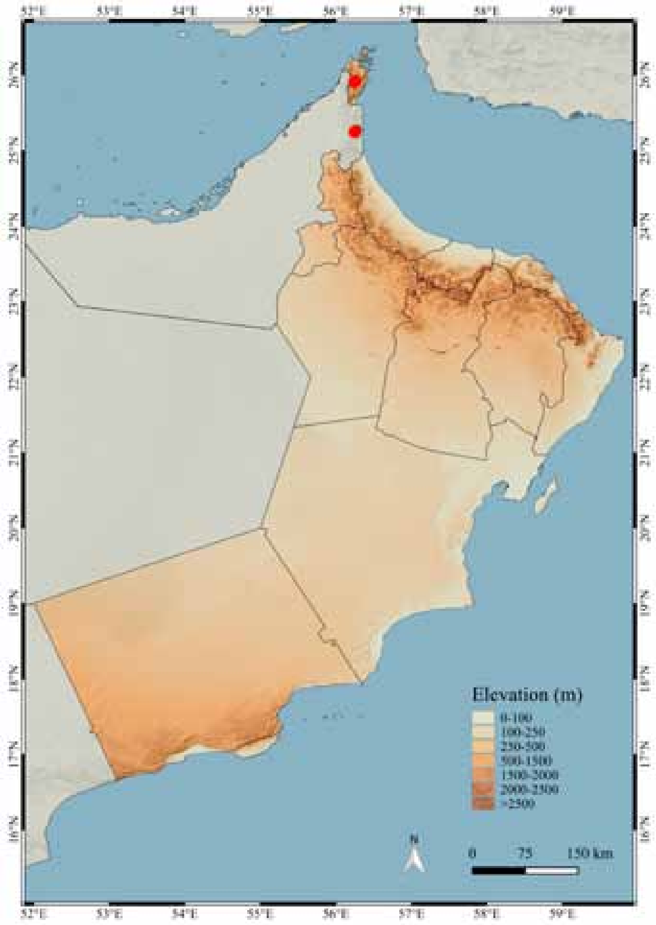

C

Useful information	
IUCN Category	NE
Endemic	NO
Venomous	NO
Insular	NO
Present in a protected area	NO

D

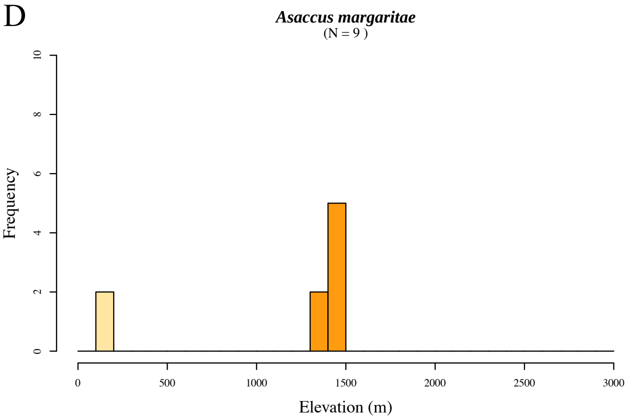

E

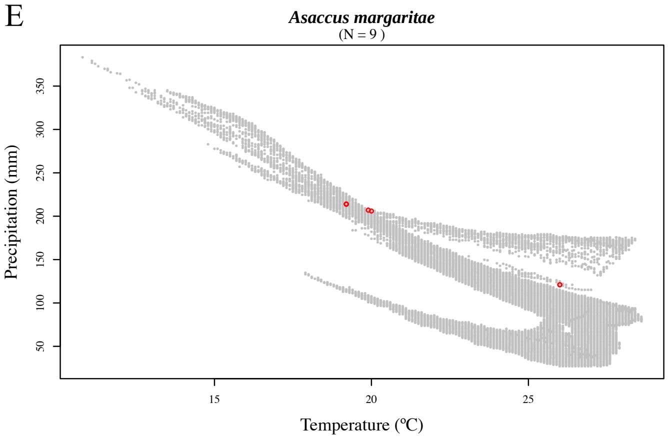

F

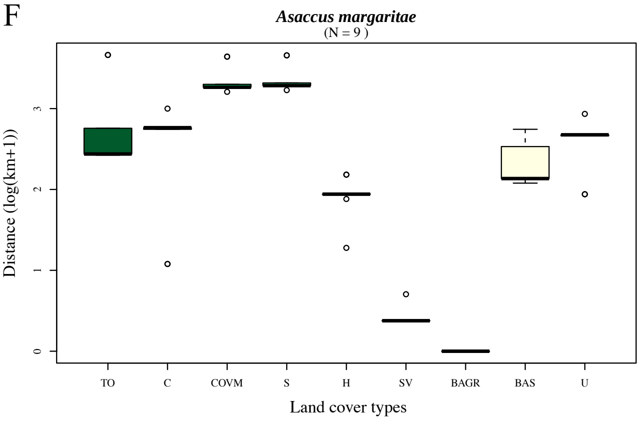

S43: Species information

Lizards

Geckos, Phyllodactylidae

Asaccus montanus
Gardner, 1994

A

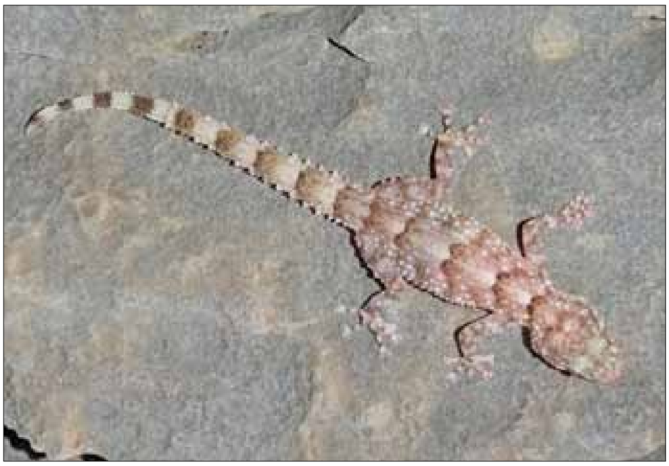

B

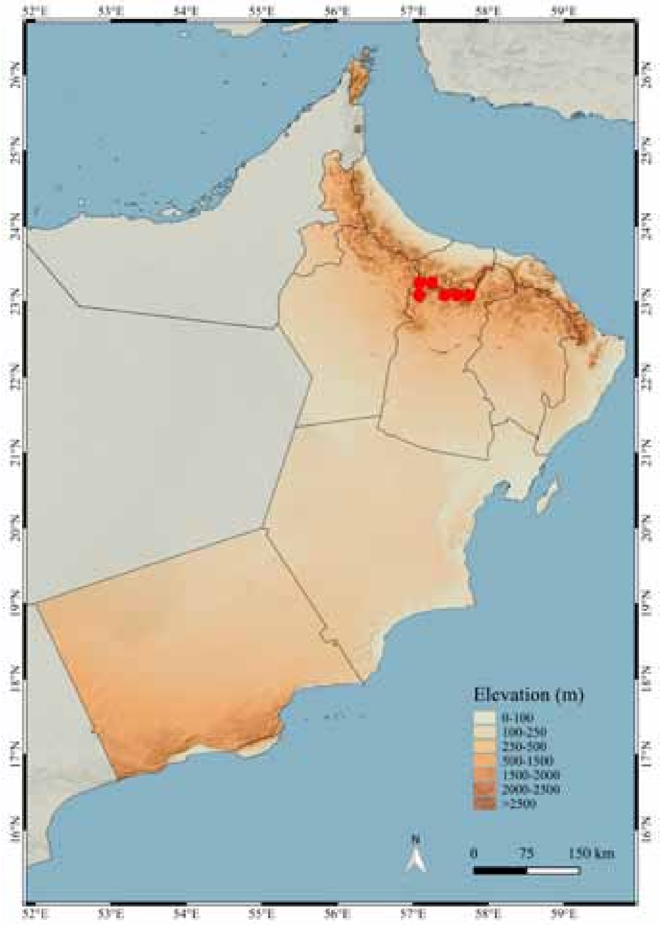

C

Useful information	
IUCN Category	VU
Endemic	YES
Venomous	NO
Insular	NO
Present in a protected area	NO

D

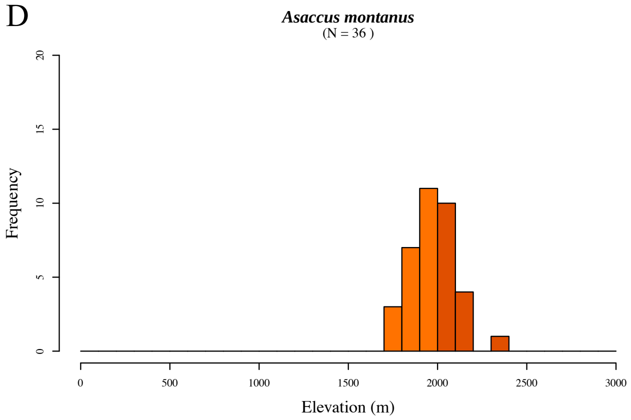

E

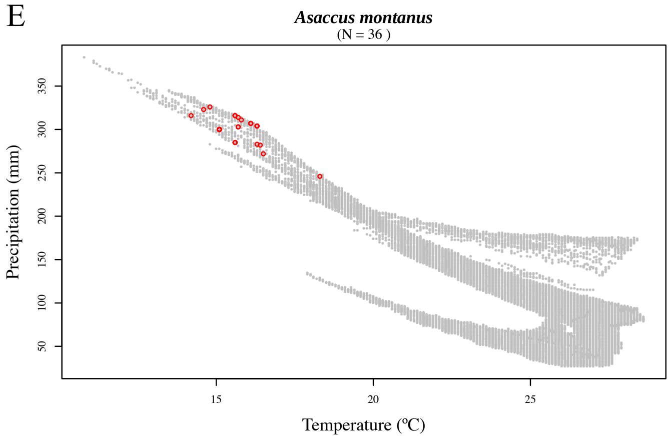

F

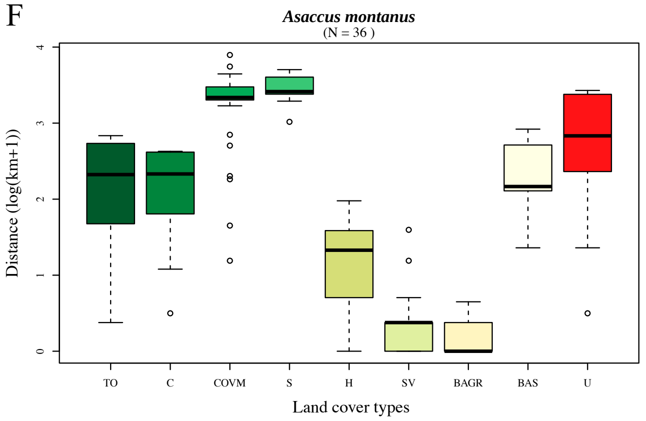

Asaccus platyrhynchus
Arnold & Gardner, 1994

S44: Species information
Lizards
Geckos, Phyllodactylidae

A

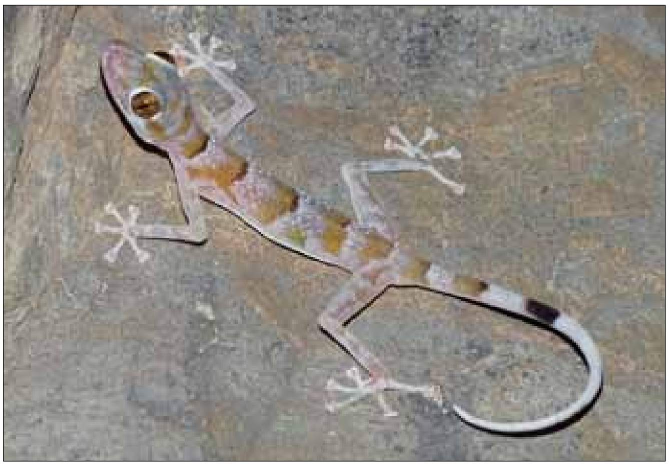

B

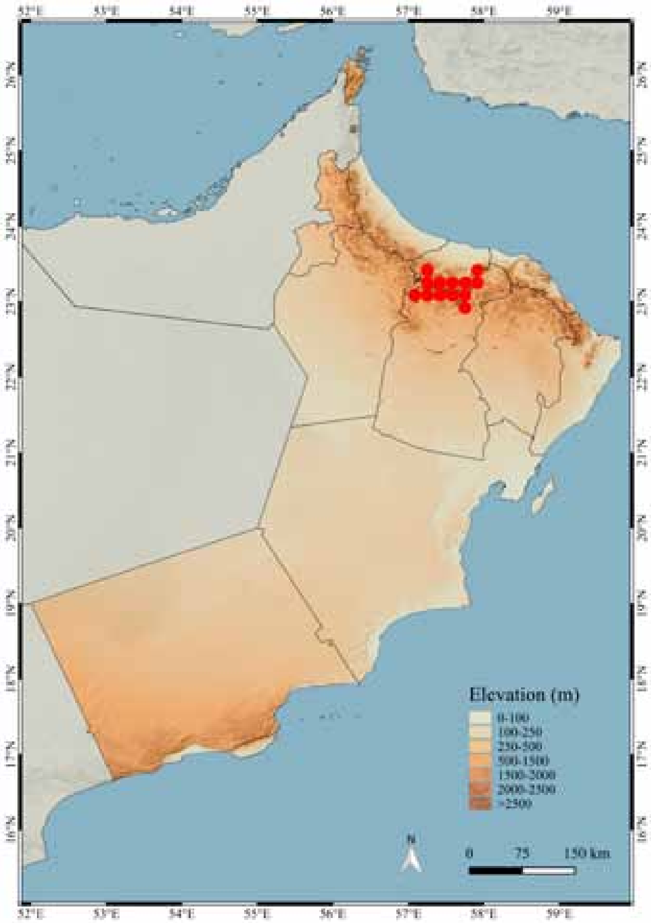

C

Useful information	
IUCN Category	LC
Endemic	YES
Venomous	NO
Insular	NO
Present in a protected area	NO

D

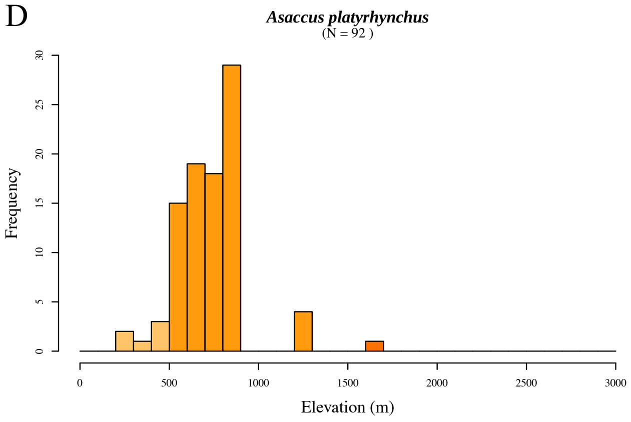

E

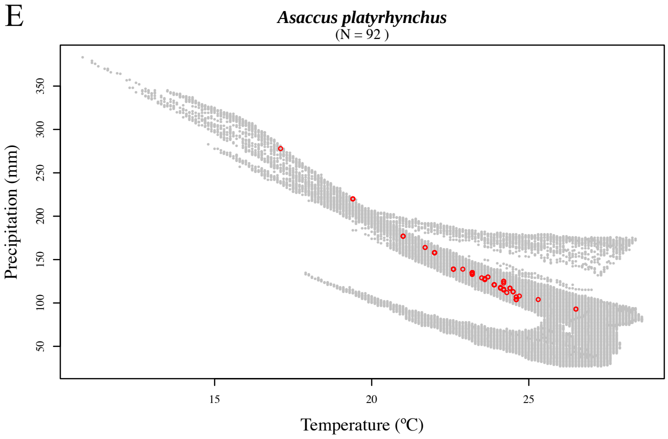

F

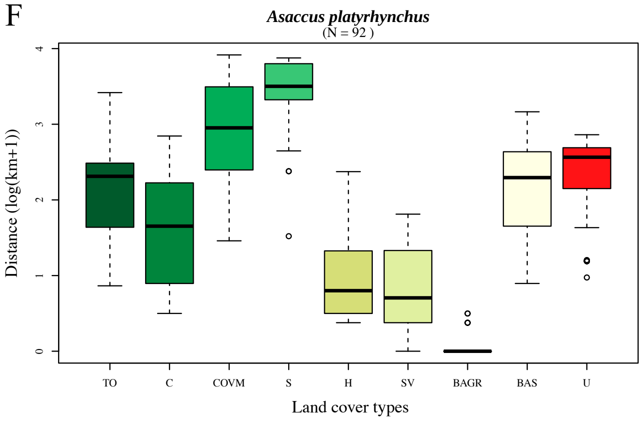

S45: Species information

Lizards

Geckos, Phyllodactylidae

Asaccus arnoldi

Simó-Riudalbas, Tarroso, Papenfuss, Al-Sariri & Carranza, 2017

A

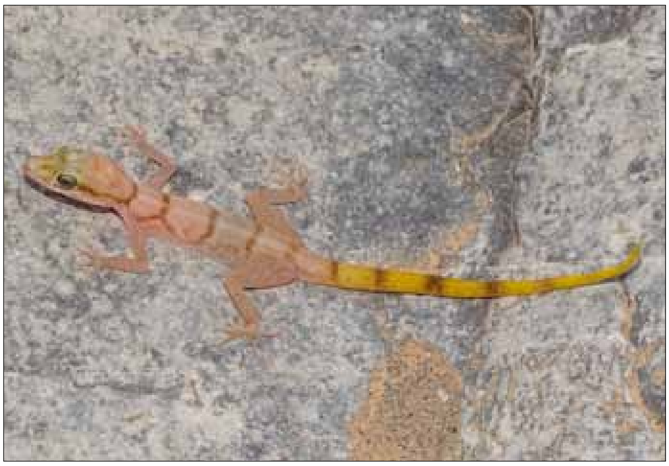

B

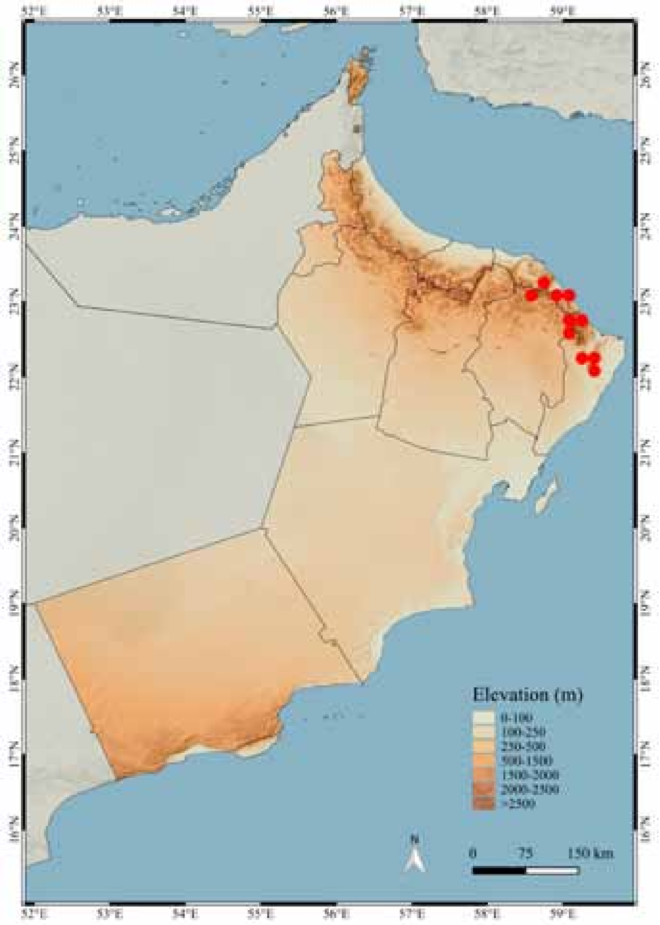

C

Useful information	
IUCN Category	NE
Endemic	YES
Venomous	NO
Insular	NO
Present in a protected area	YES

D

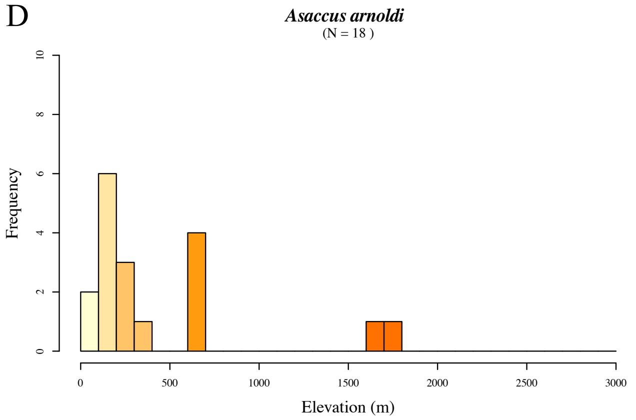

E

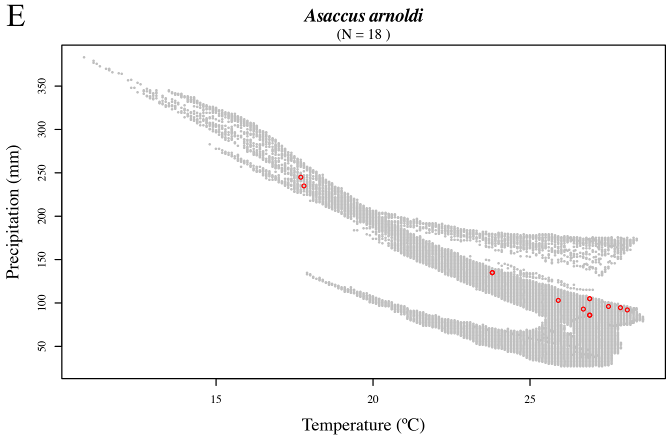

F

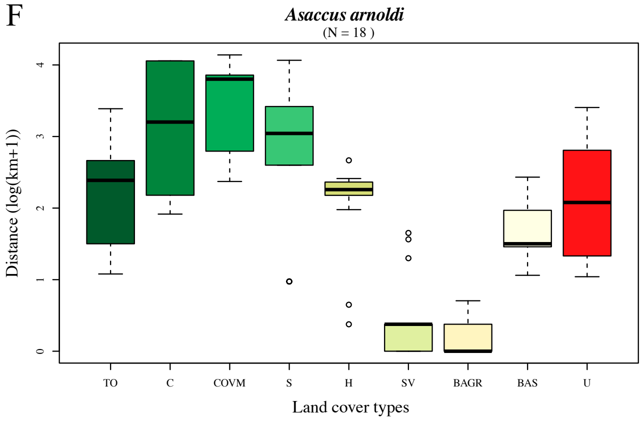

Ptyodactylus dhofarensis
Nazarov, Melnikov & Melnikova, 2013

S46: Species information
Lizards
Geckos, Phyllodactylidae

A

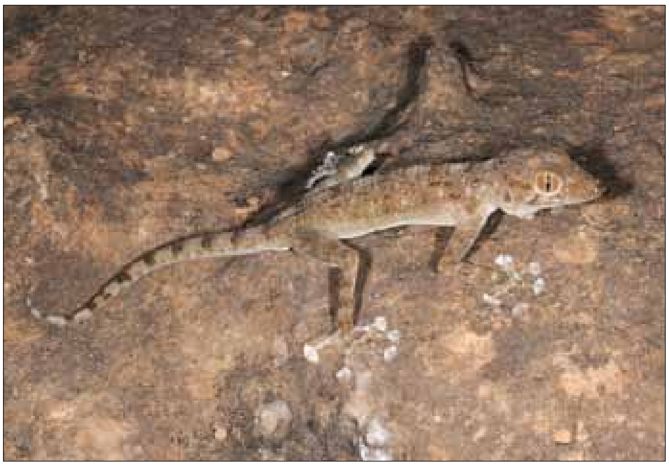

B

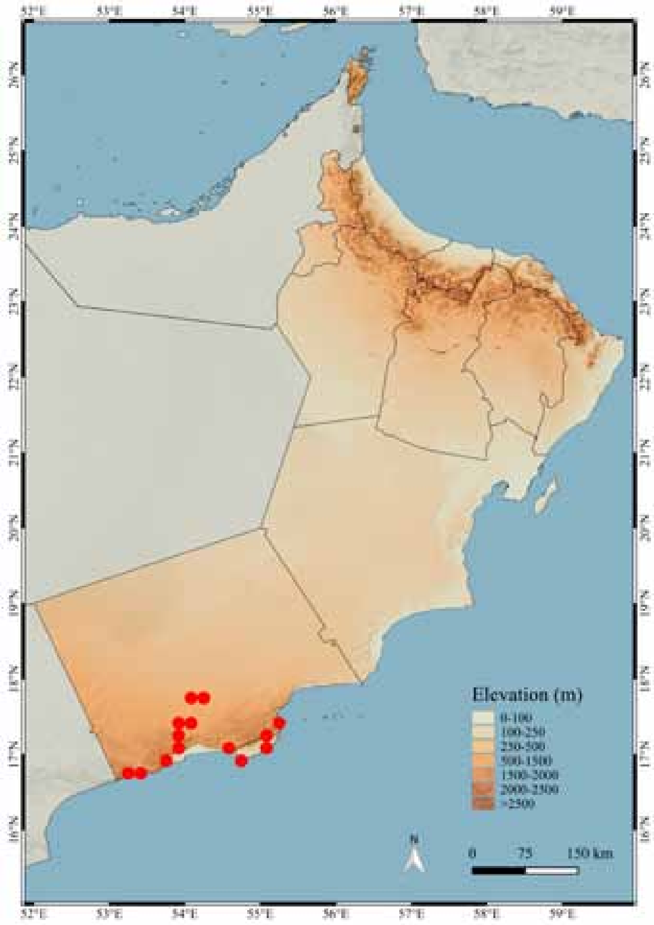

C

Useful information	
IUCN Category	NE
Endemic	NO
Venomous	NO
Insular	NO
Present in a protected area	YES

D

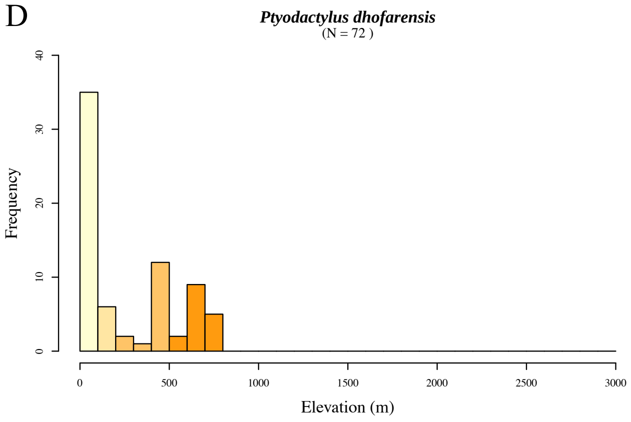

E

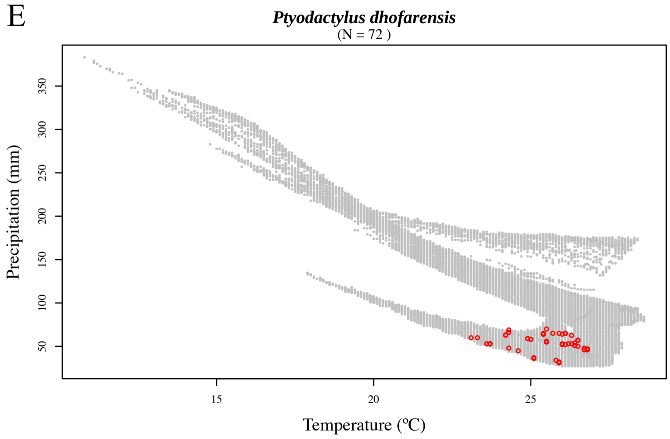

F

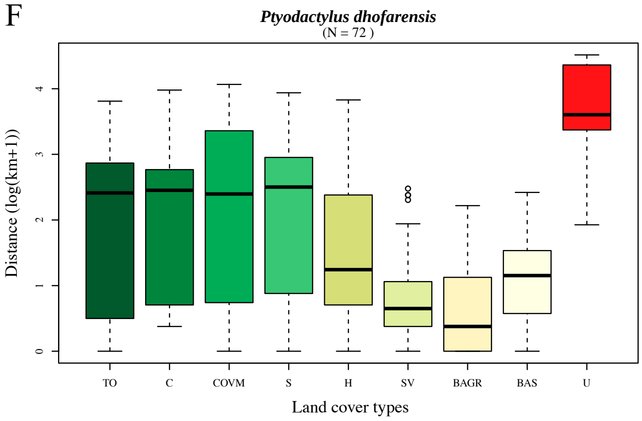

S47: Species information

Lizards

Geckos, Phyllodactylidae

Ptyodactylus orlovi

Nazarov, Melnikov & Melnikova, 2013

A

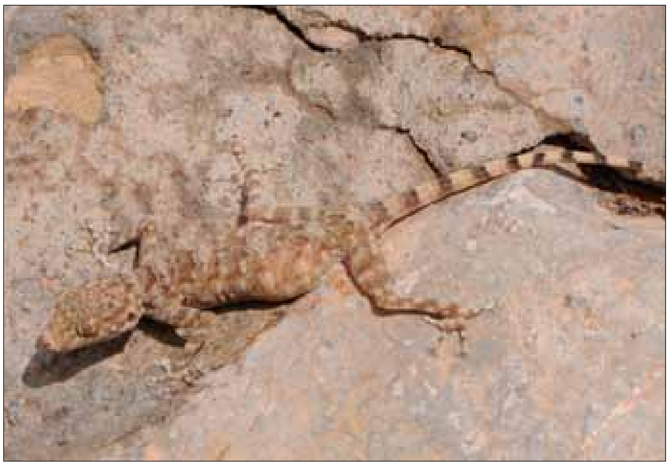

B

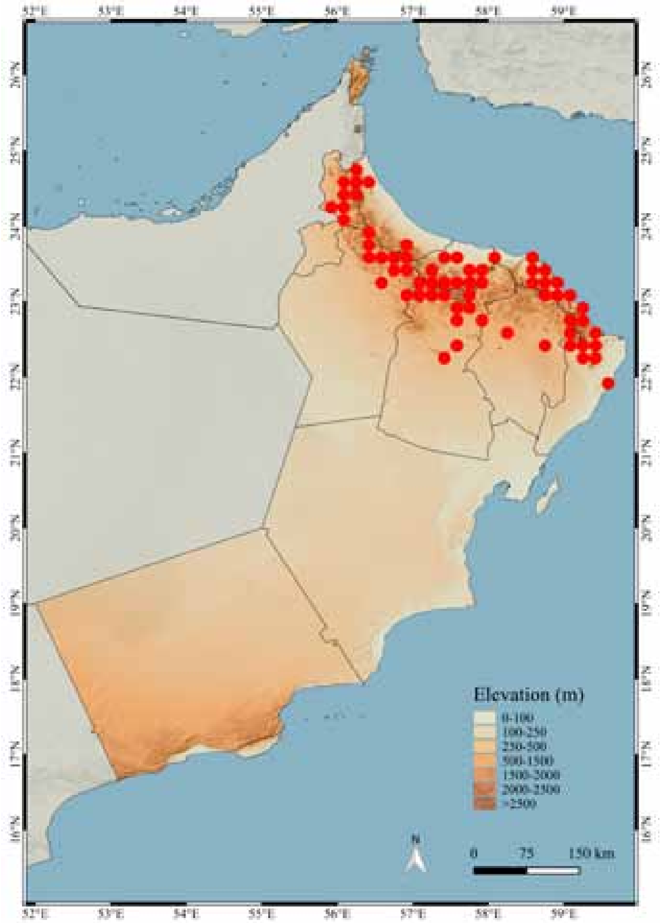

C

Useful information	
IUCN Category	NE
Endemic	NO
Venomous	NO
Insular	NO
Present in a protected area	YES

D

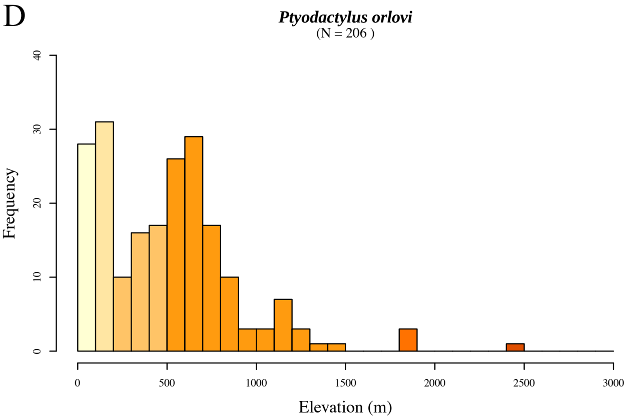

E

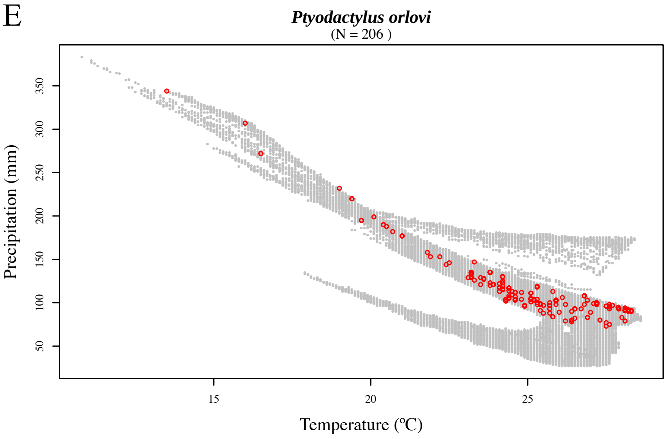

F

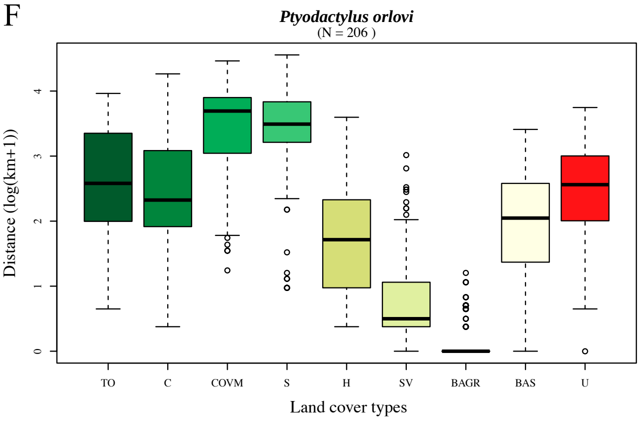

Ptyodactylus ruusaljibalicus
Undescribed

S48: Species information
Lizards
Geckos, Phyllodactylidae

A

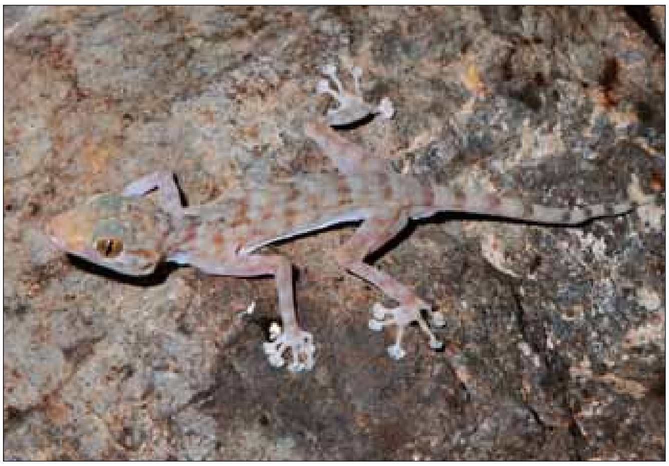

B

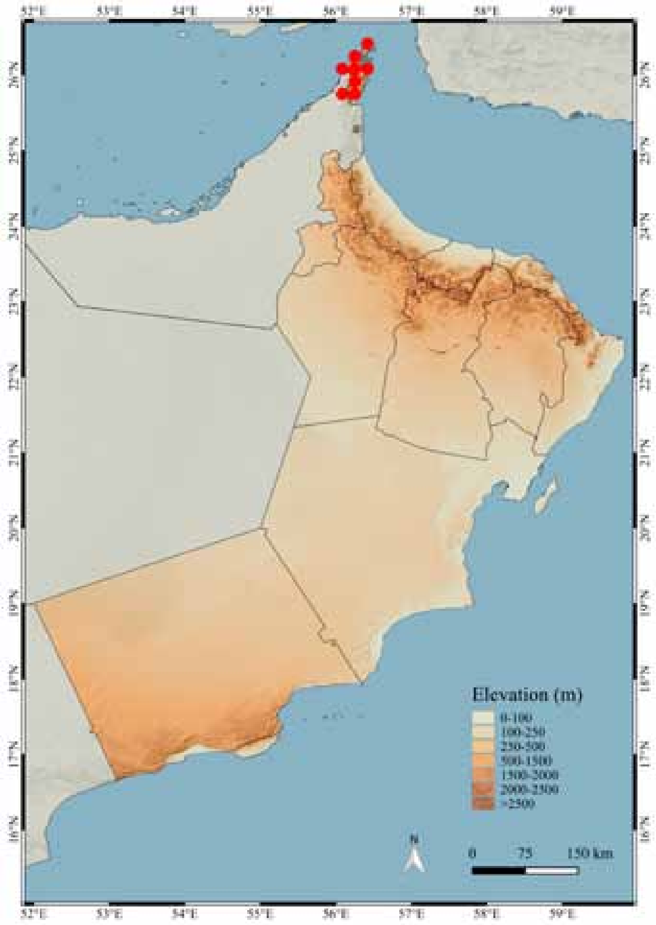

C

Useful information	
IUCN Category	NE
Endemic	NO
Venomous	NO
Insular	YES
Present in a protected area	NO

D

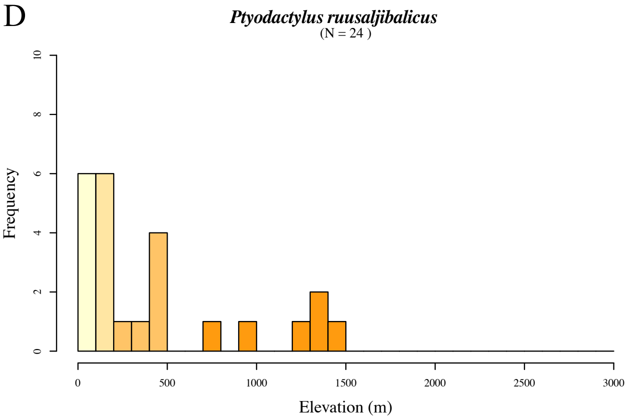

E

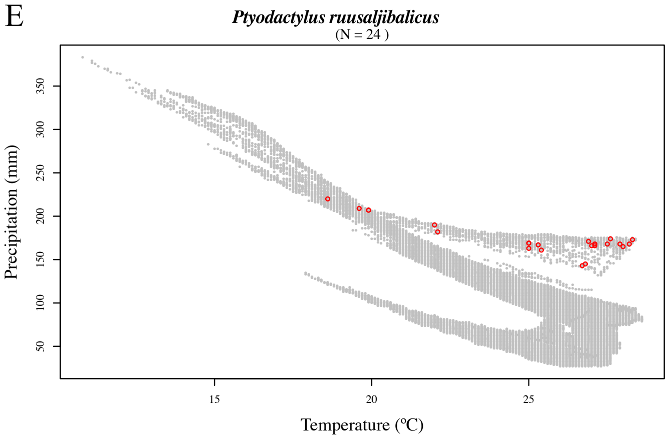

F

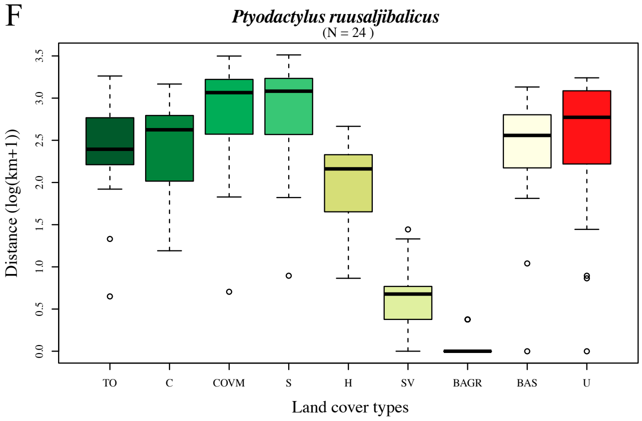

S49: Species information

Lizards

Geckos, Sphaerodactylidae

Pristurus carteri
(Gray, 1863)

A

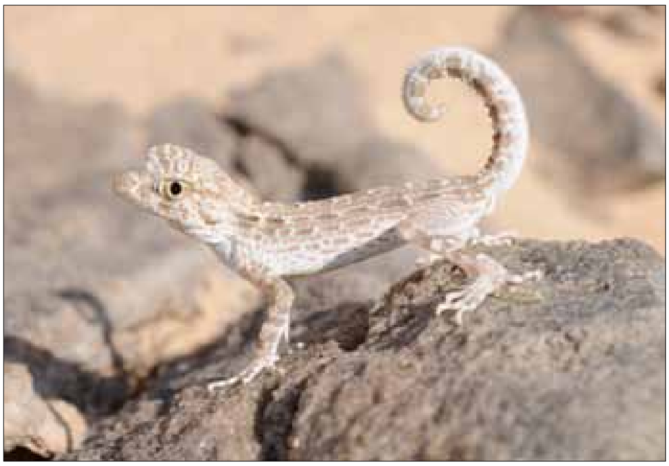

B

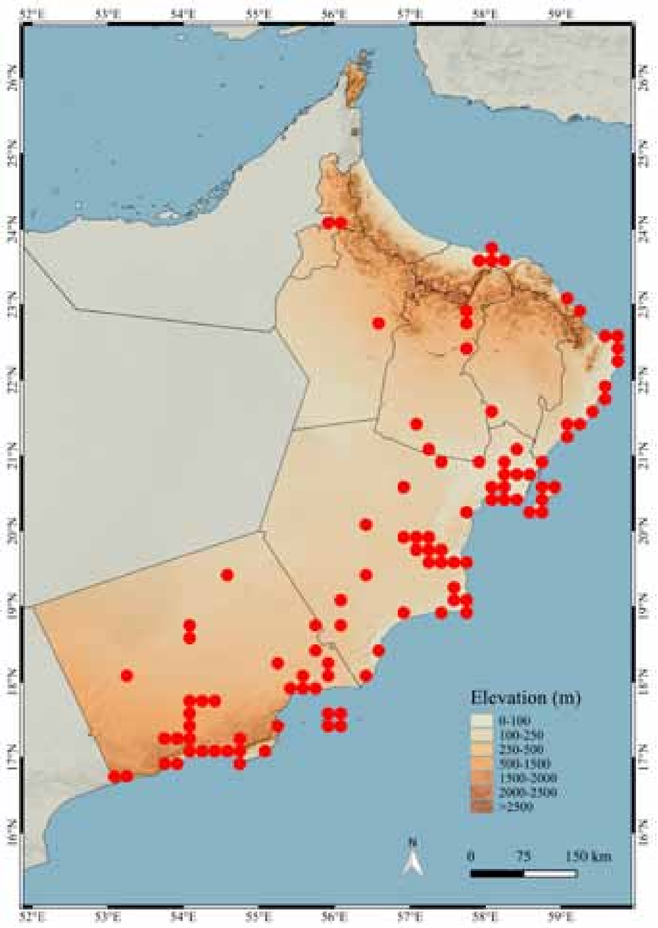

C

Useful information	
IUCN Category	LC
Endemic	NO
Venomous	NO
Insular	YES
Present in a protected area	YES

D

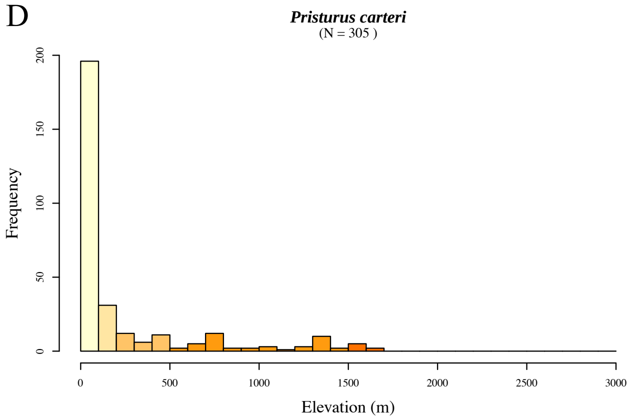

E

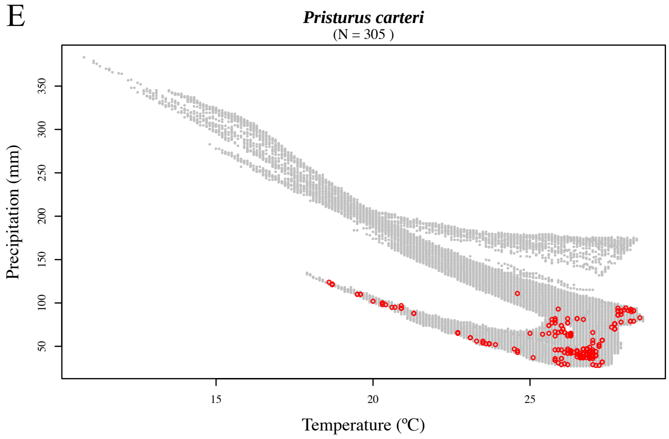

F

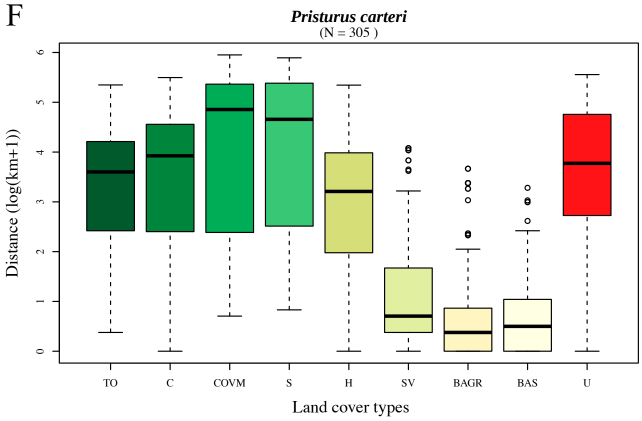

Pristurus celerrimus
Arnold, 1977

S50: Species information
Lizards
Geckos, Sphaerodactylidae

A

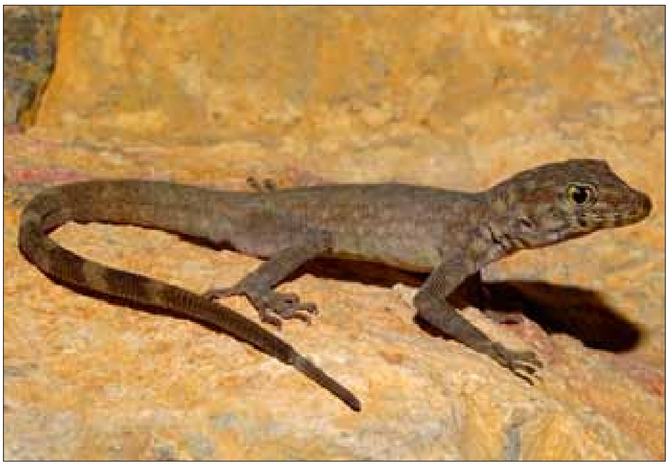

B

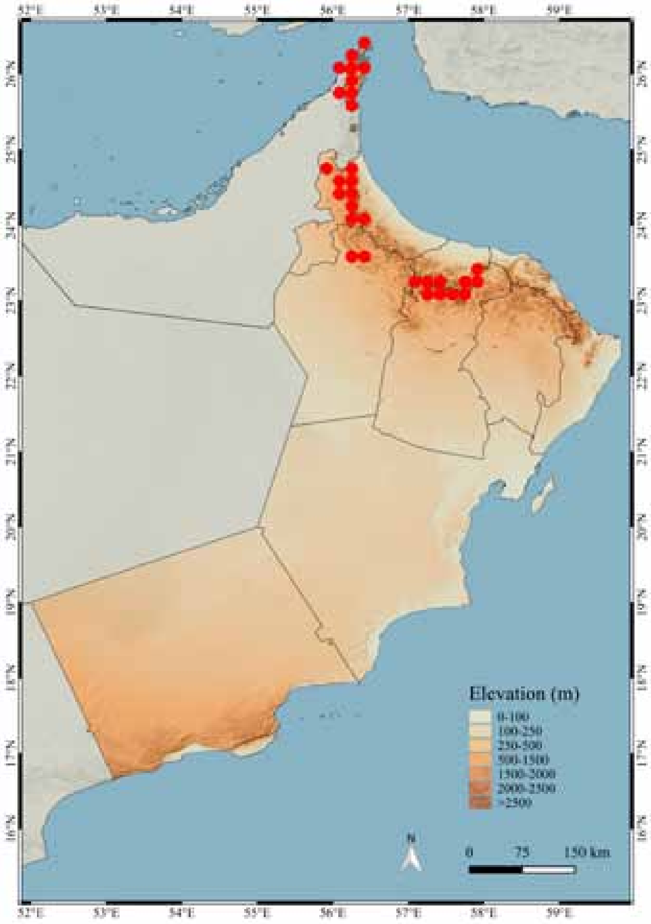

C

Useful information	
IUCN Category	LC
Endemic	NO
Venomous	NO
Insular	YES
Present in a protected area	YES

D

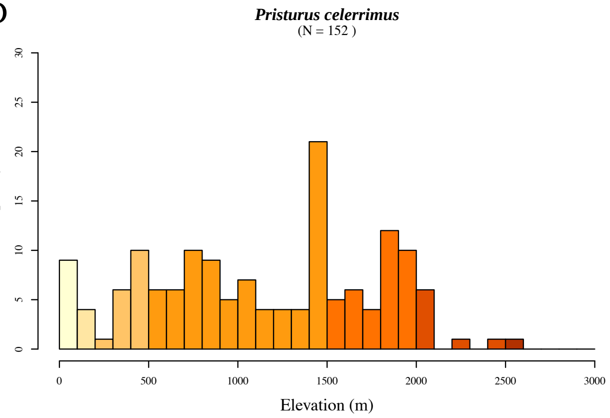

E

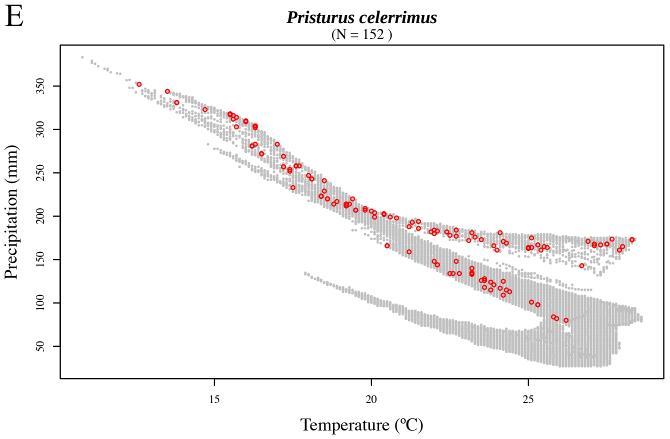

F

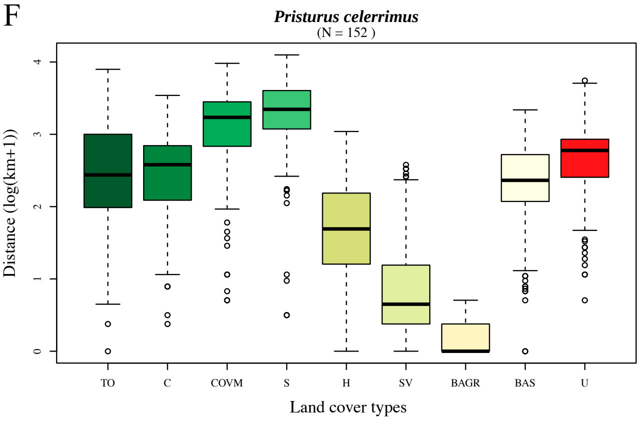

S51: Species information

Lizards

Geckos, Sphaerodactylidae

Pristurus gallagheri
Arnold, 1986

A

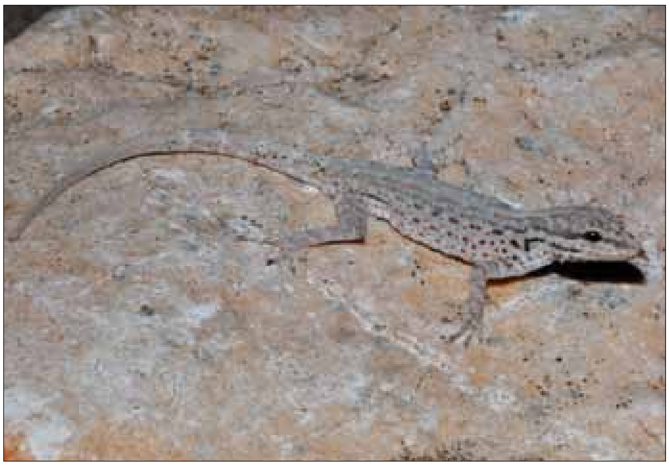

B

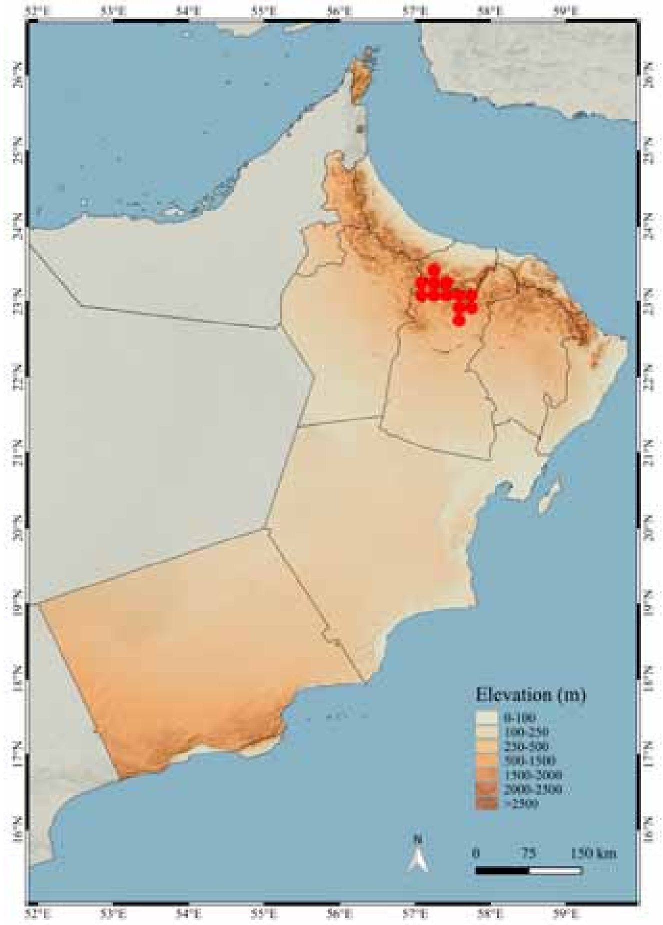

C

Useful information	
IUCN Category	NT
Endemic	YES
Venomous	NO
Insular	NO
Present in a protected area	YES

D

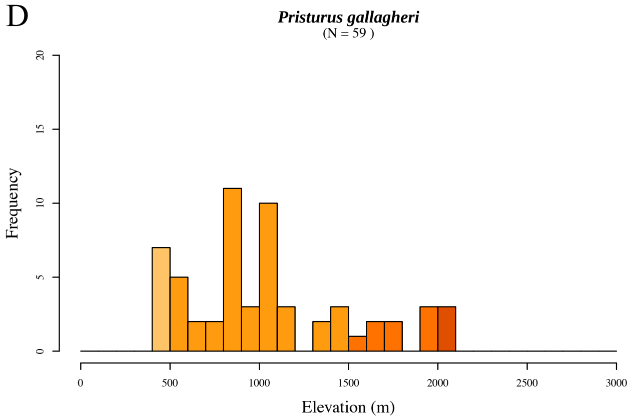

E

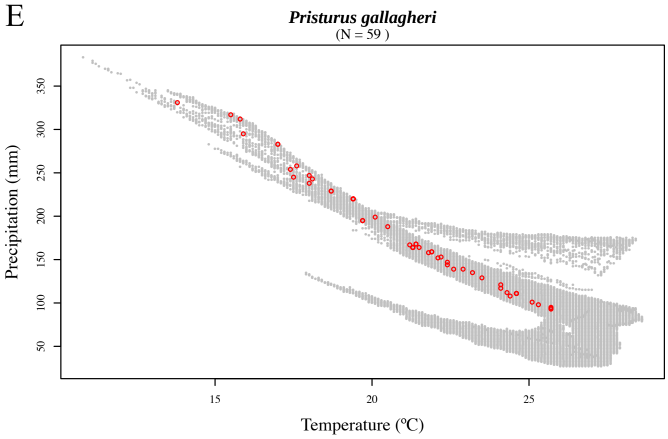

F

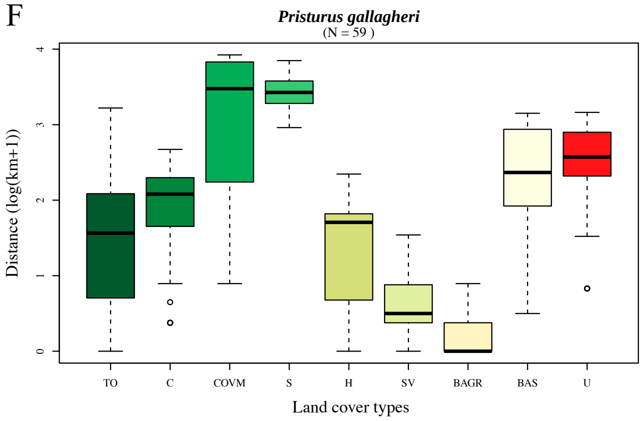

Pristurus minimus
Arnold, 1977

S52: Species information
Lizards
Geckos, Sphaerodactylidae

A

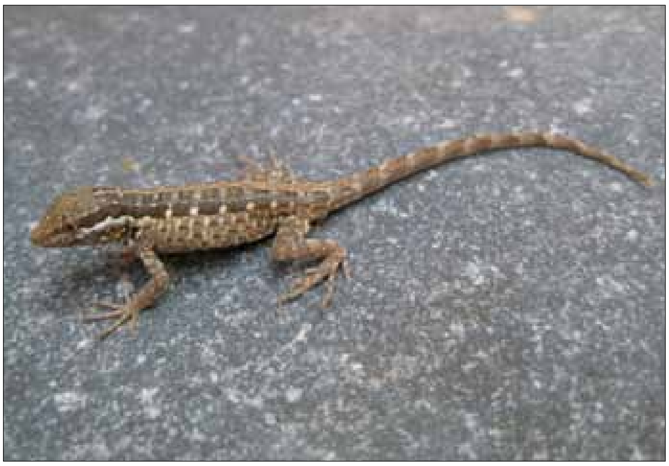

B

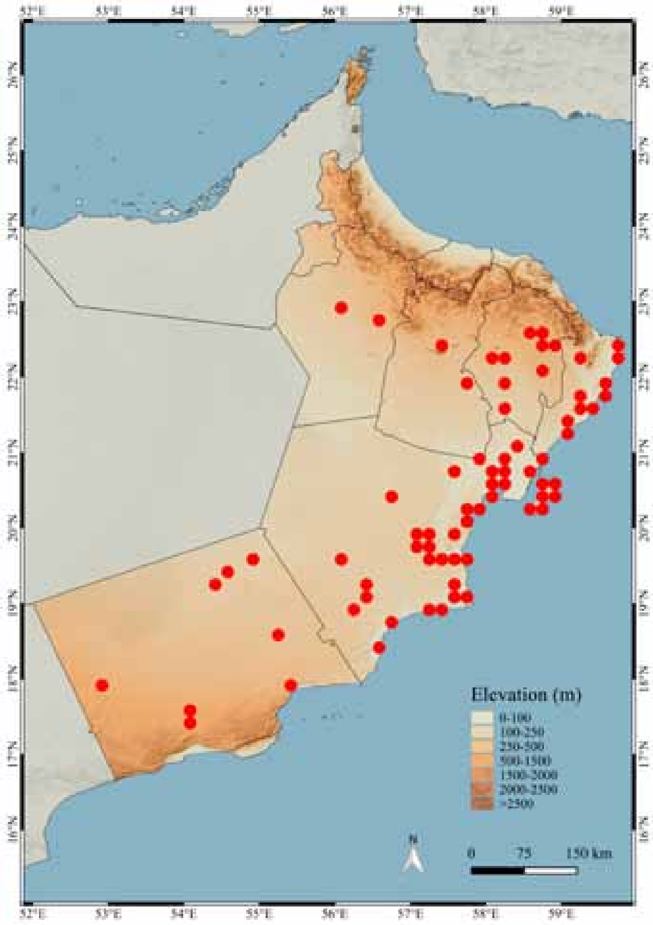

C

Useful information	
IUCN Category	LC
Endemic	NO
Venomous	NO
Insular	YES
Present in a protected area	YES

D

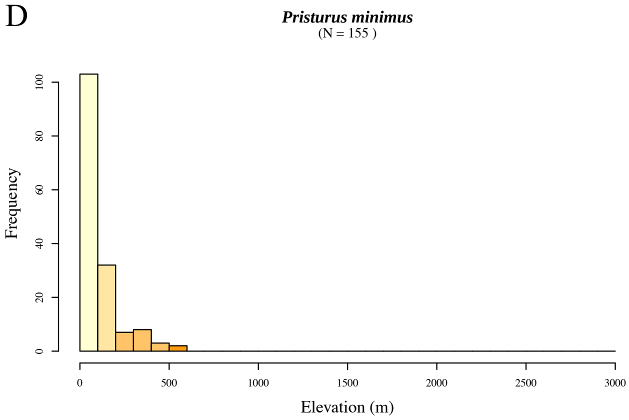

E

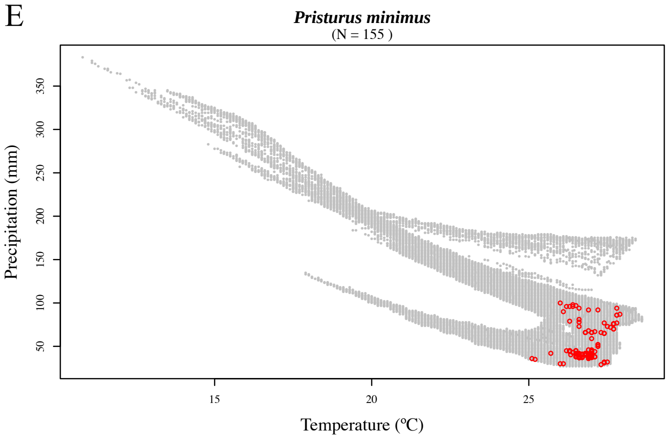

F

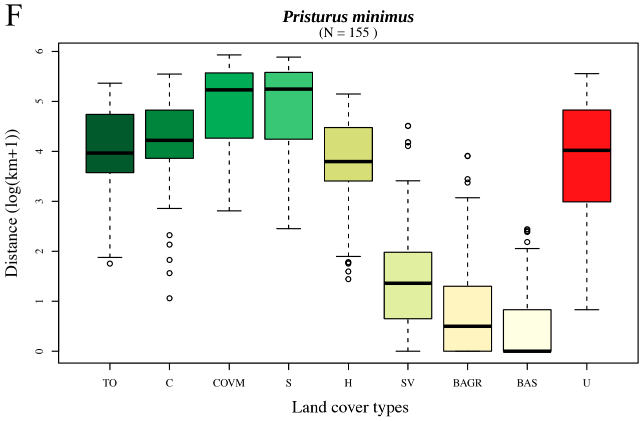

S53: Species information

Lizards

Geckos, Sphaerodactylidae

Pristurus rupestris rupestris

Blanford, 1874

A

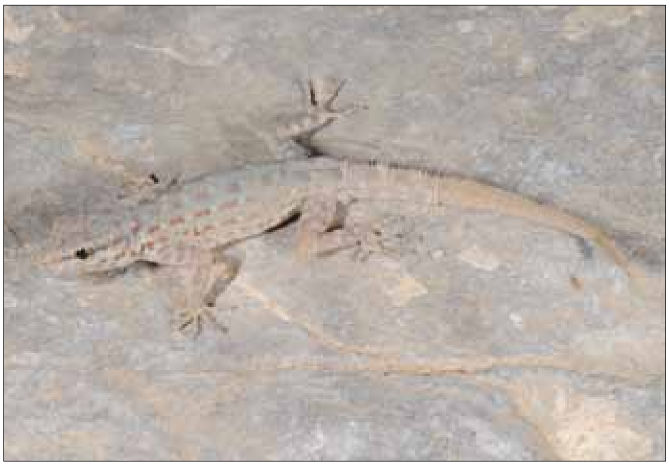

B

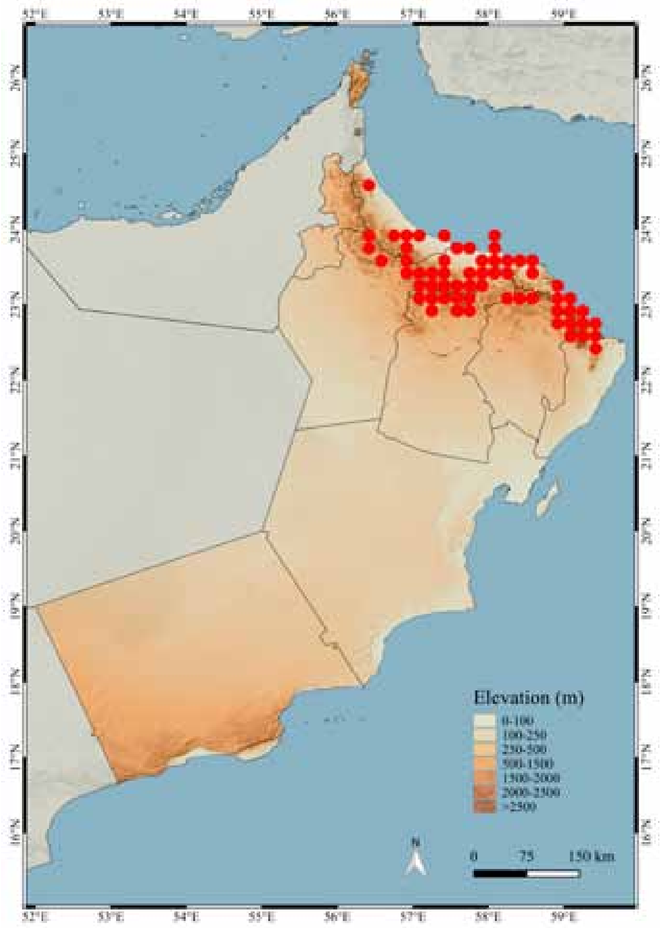

C

Useful information

IUCN Category	NE
Endemic	NO
Venomous	NO
Insular	YES
Present in a protected area	YES

D

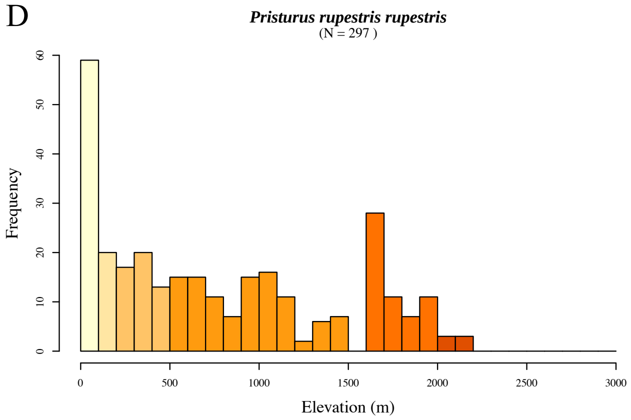

E

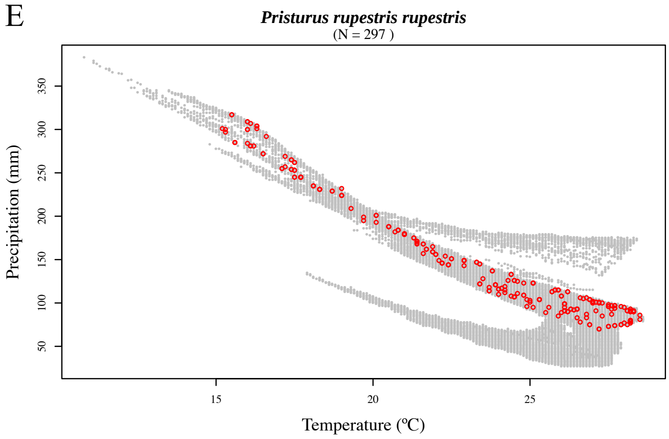

F

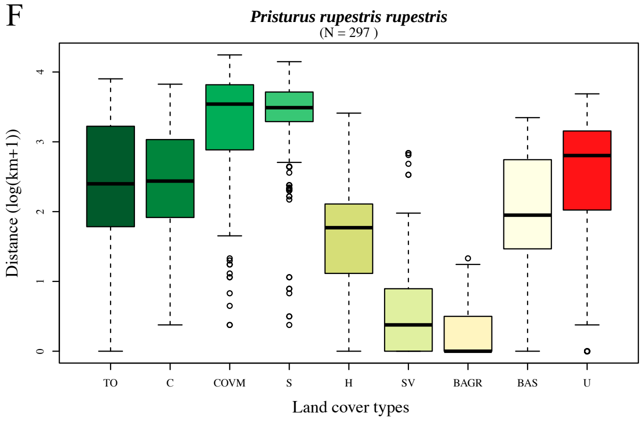

Pristurus sp. 1
Undescribed

S54: Species information
Lizards
Geckos, Sphaerodactylidae

A

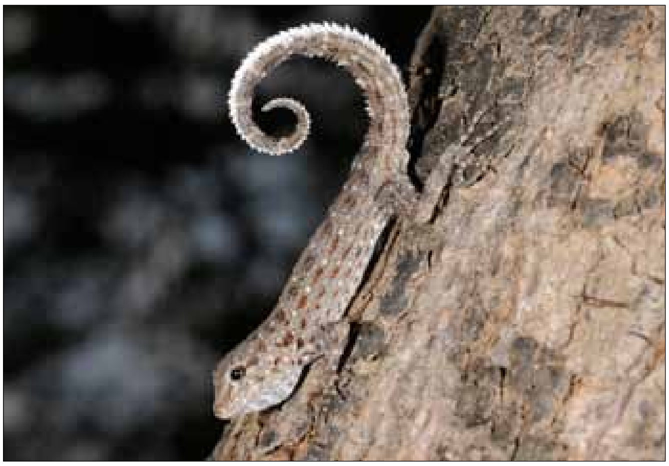

B

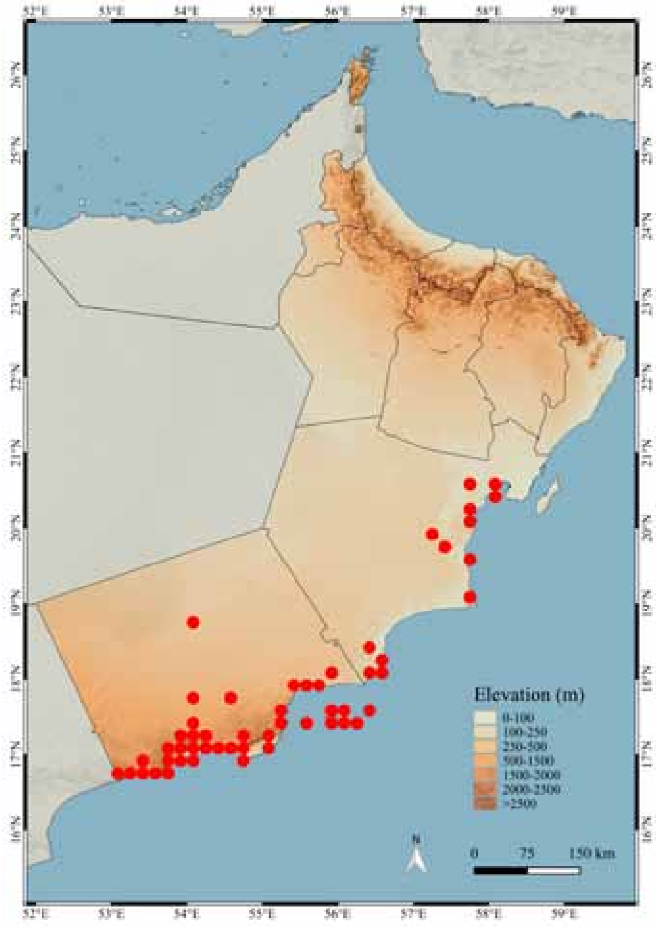

C

Useful information	
IUCN Category	NE
Endemic	NO
Venomous	NO
Insular	YES
Present in a protected area	YES

D

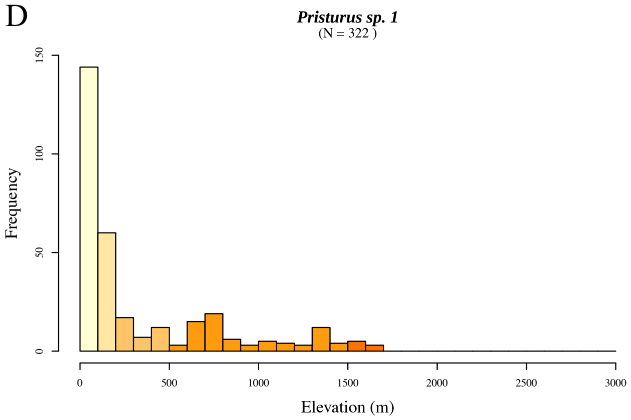

E

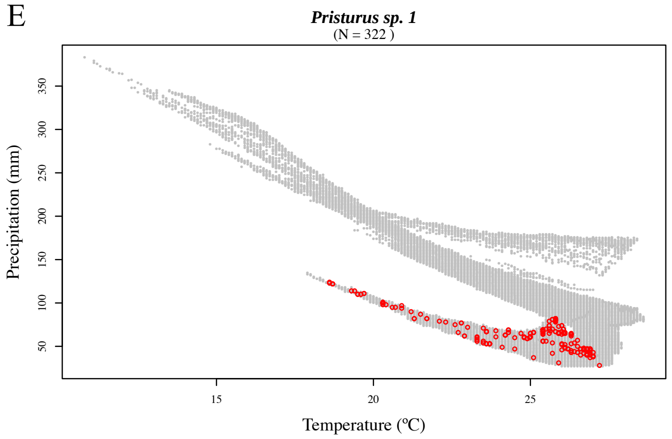

F

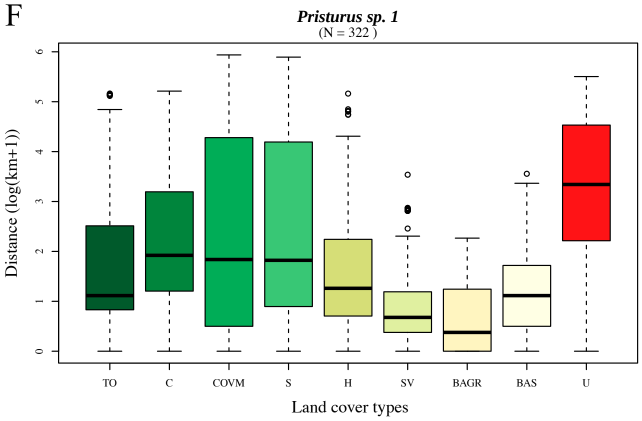

S55: Species information

Lizards

Geckos, Sphaerodactylidae

Pristurus sp. 2
Undescribed

A

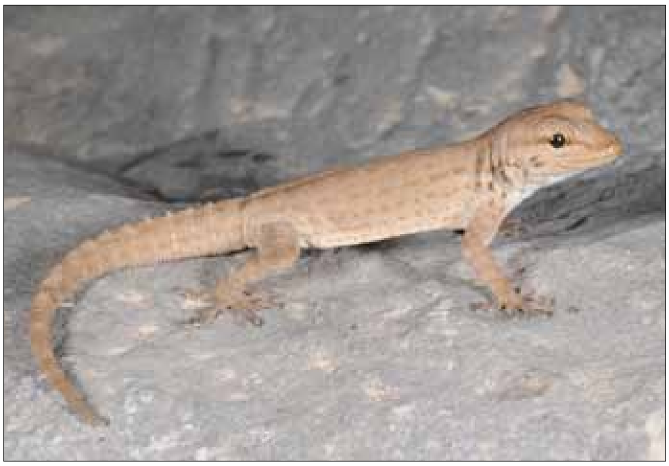

B

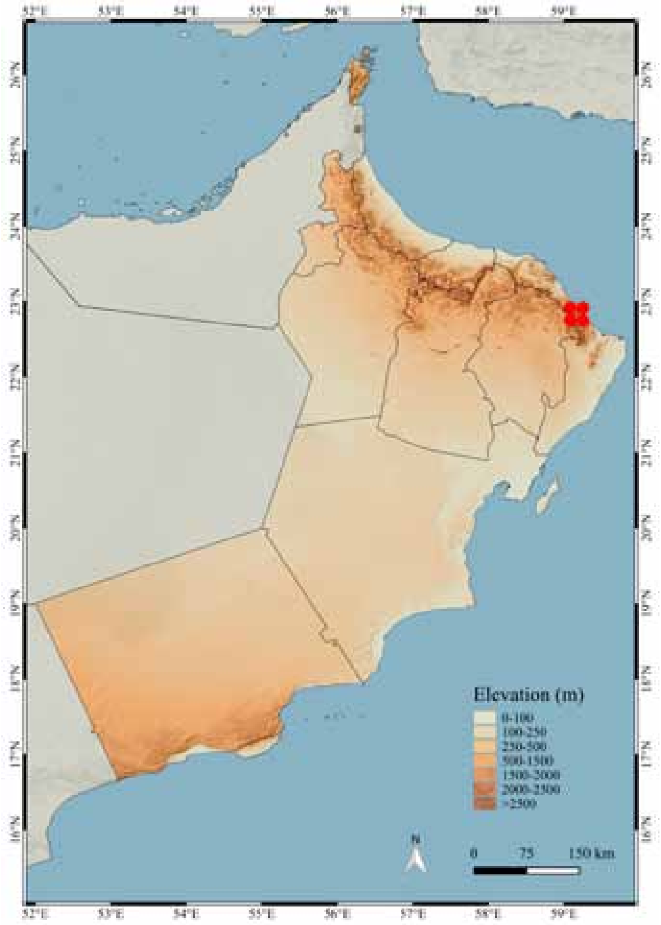

C

Useful information	
IUCN Category	NE
Endemic	YES
Venomous	NO
Insular	NO
Present in a protected area	NO

D

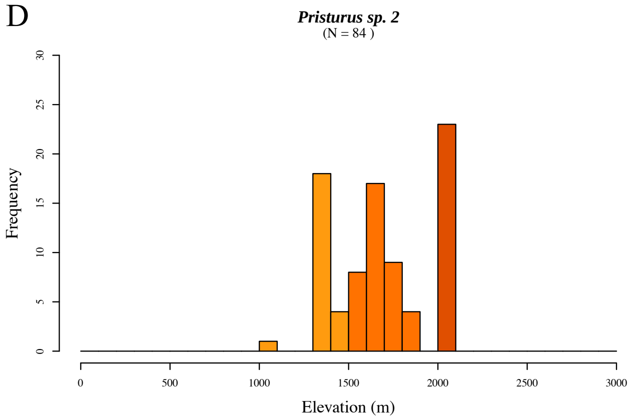

E

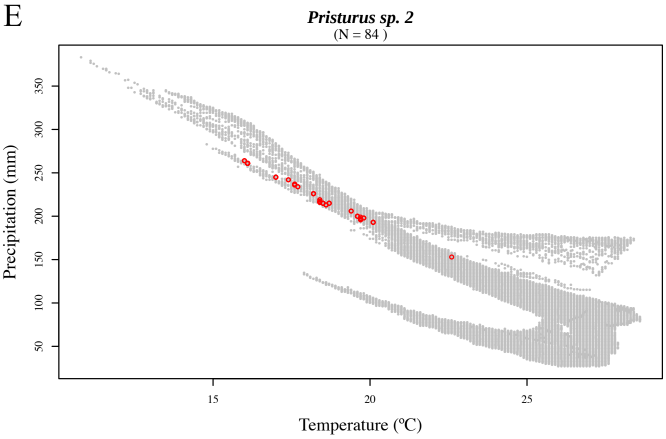

F

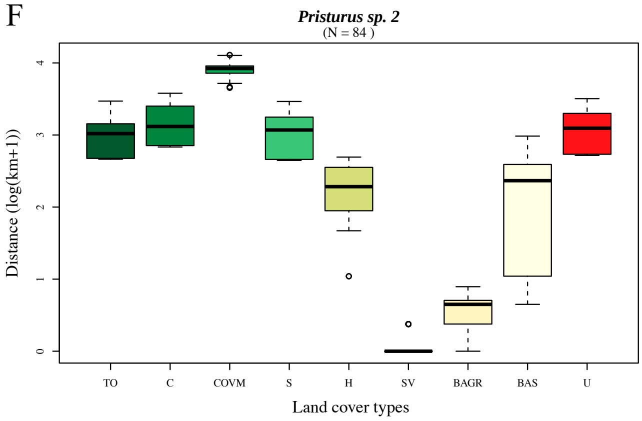

Pristurus sp. 3
Undescribed

S56: Species information
Lizards
Geckos, Sphaerodactylidae

A

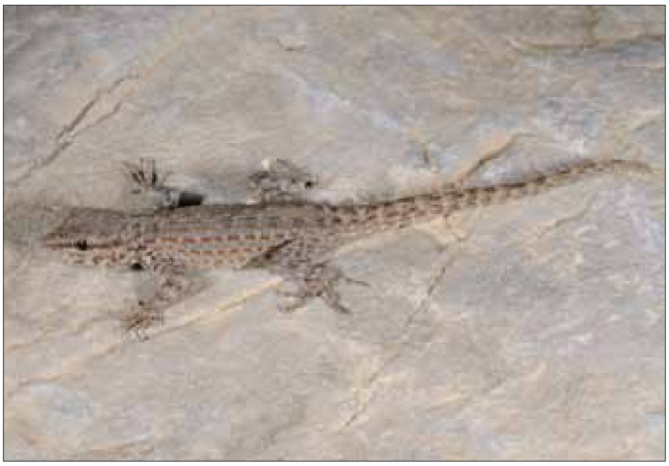

B

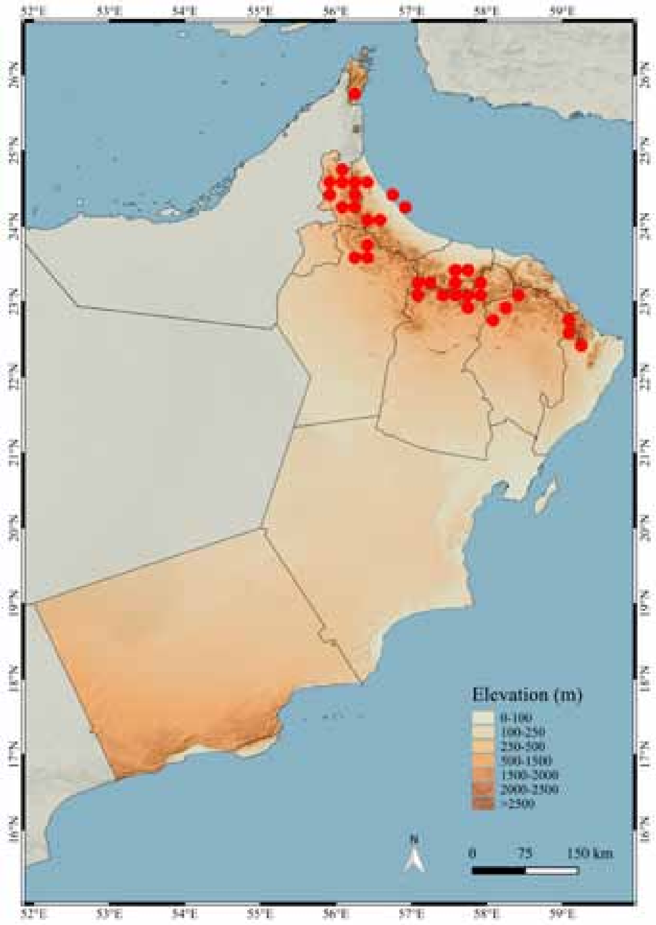

C

Useful information	
IUCN Category	NE
Endemic	NO
Venomous	NO
Insular	NO
Present in a protected area	NO

D

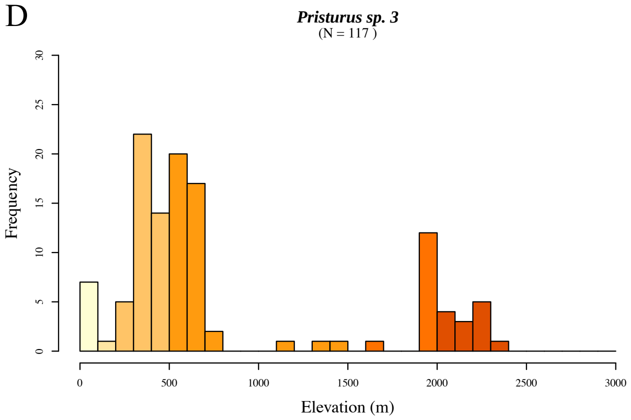

E

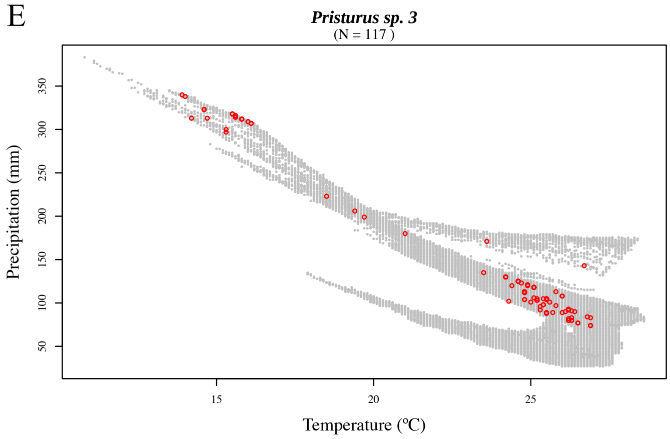

F

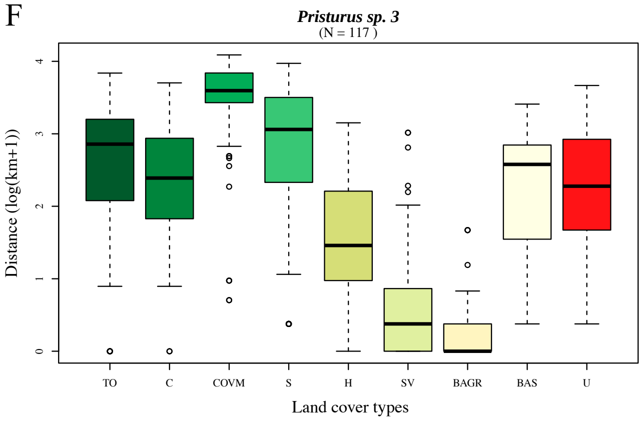

S57: Species information

Lizards

Geckos, Sphaerodactylidae

Pristurus sp. 4
Undescribed

A

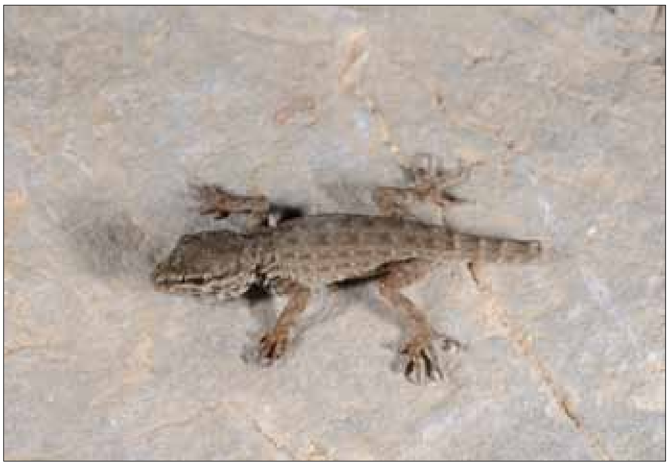

B

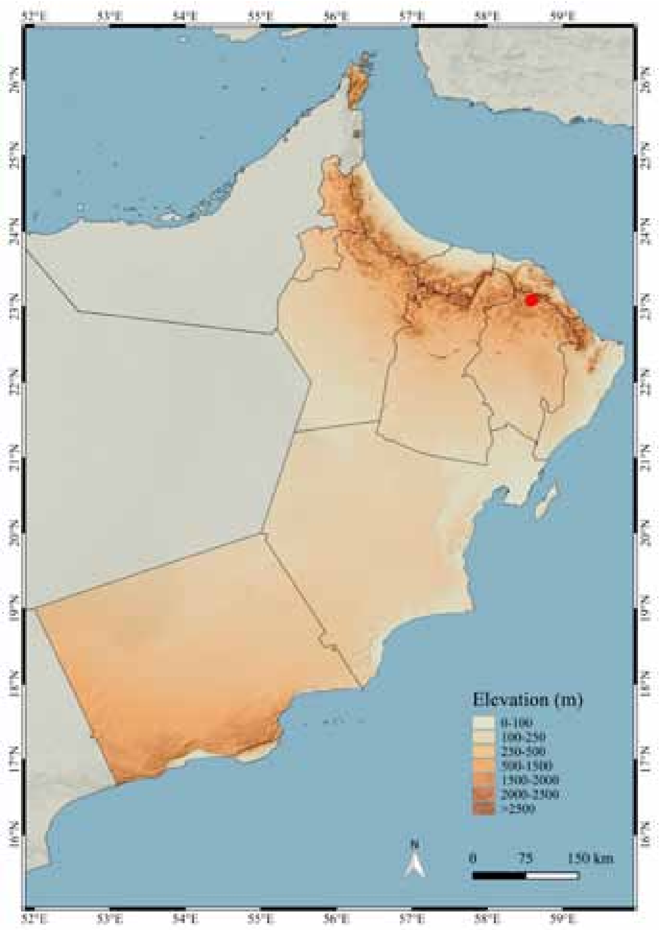

C

Useful information	
IUCN Category	NE
Endemic	YES
Venomous	NO
Insular	NO
Present in a protected area	YES

D

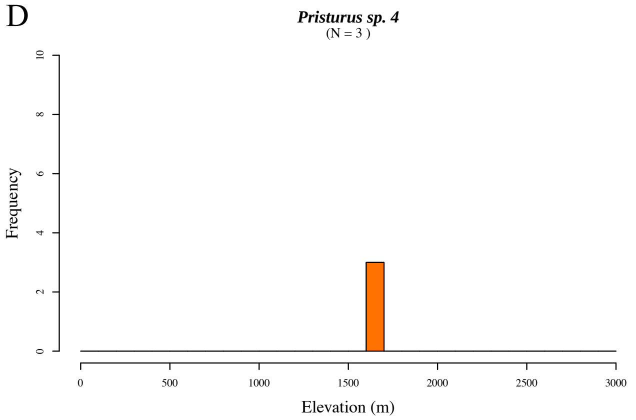

E

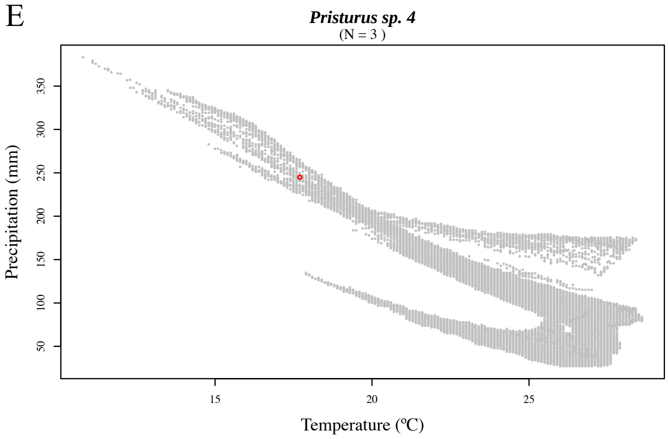

F

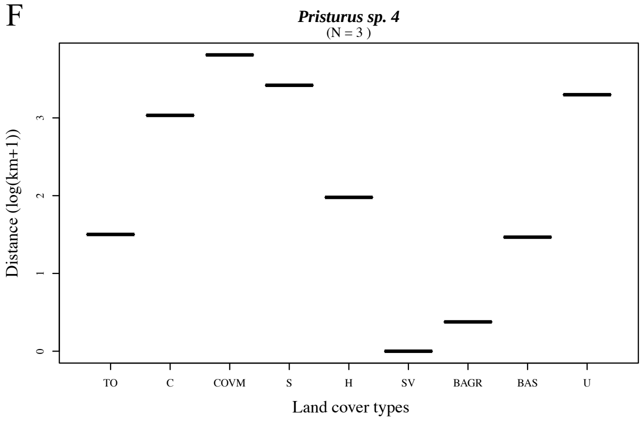

***Pristurus* sp. 5**
Undescribed

S58: Species information
Lizards
Geckos, Sphaerodactylidae

A

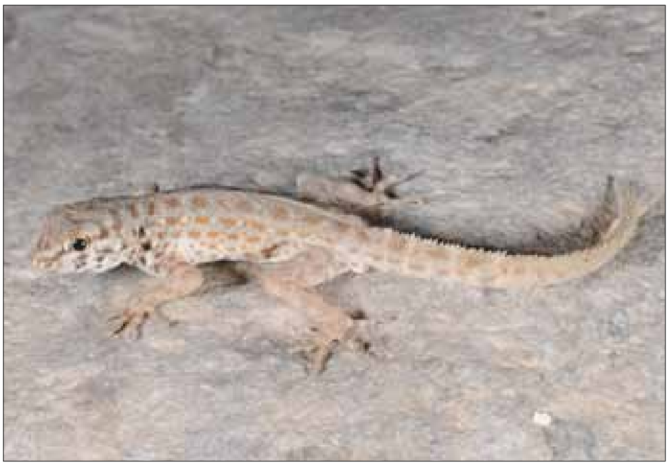

B

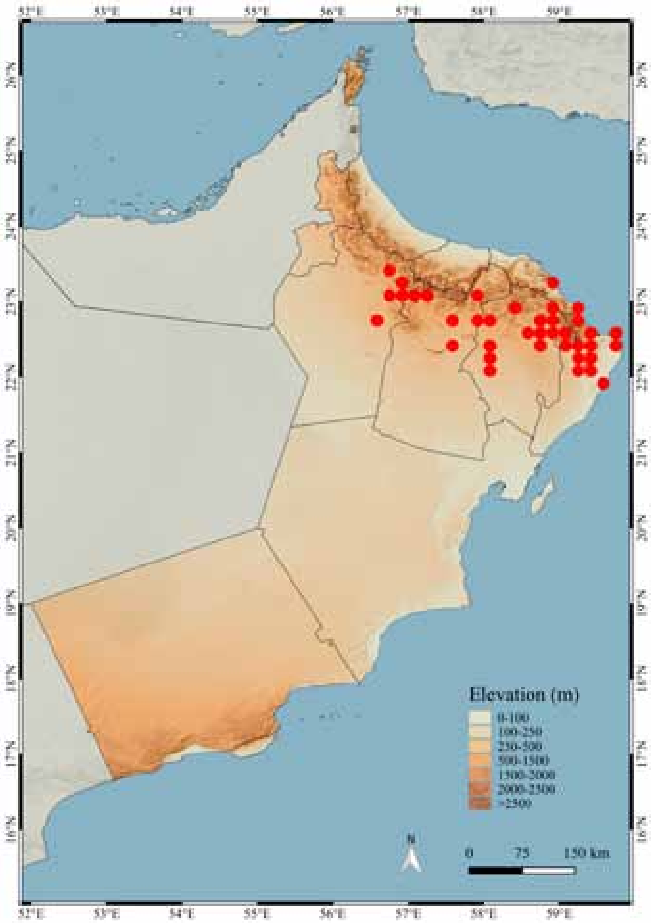

C

Useful information	
IUCN Category	NE
Endemic	YES
Venomous	NO
Insular	NO
Present in a protected area	YES

D

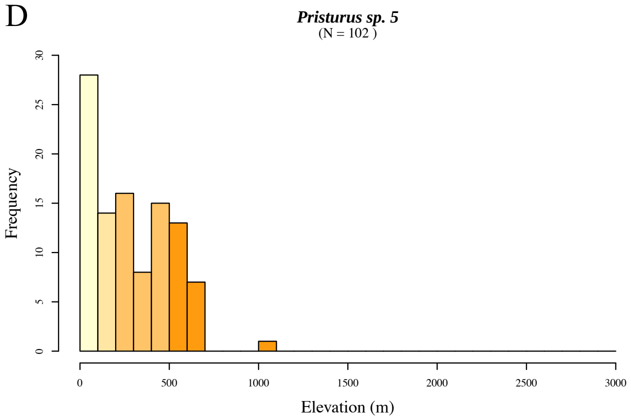

E

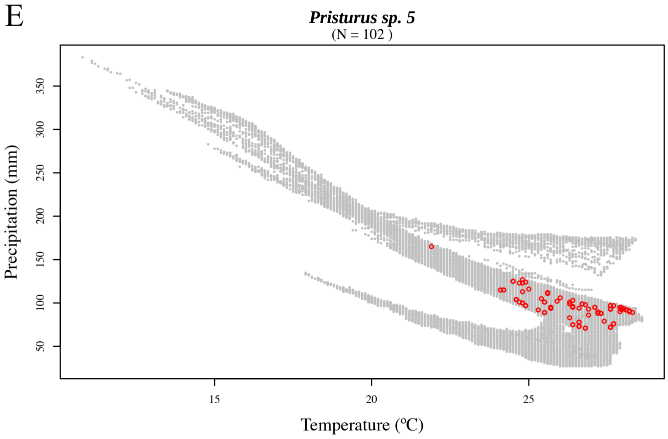

F

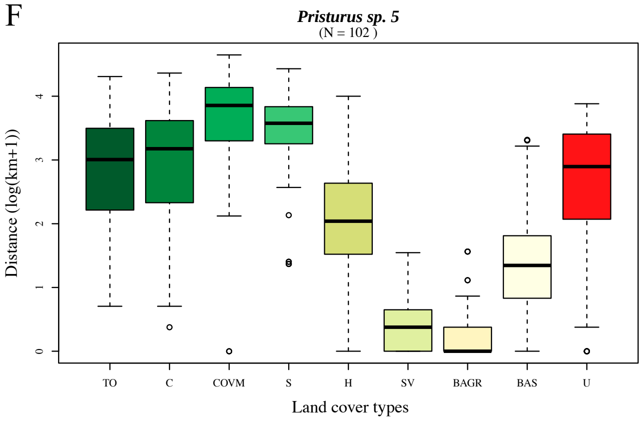

S59: Species information

Lizards

Lacertids, Lacertidae

Acanthodactylus blanfordii
Boulanger, 1918

B

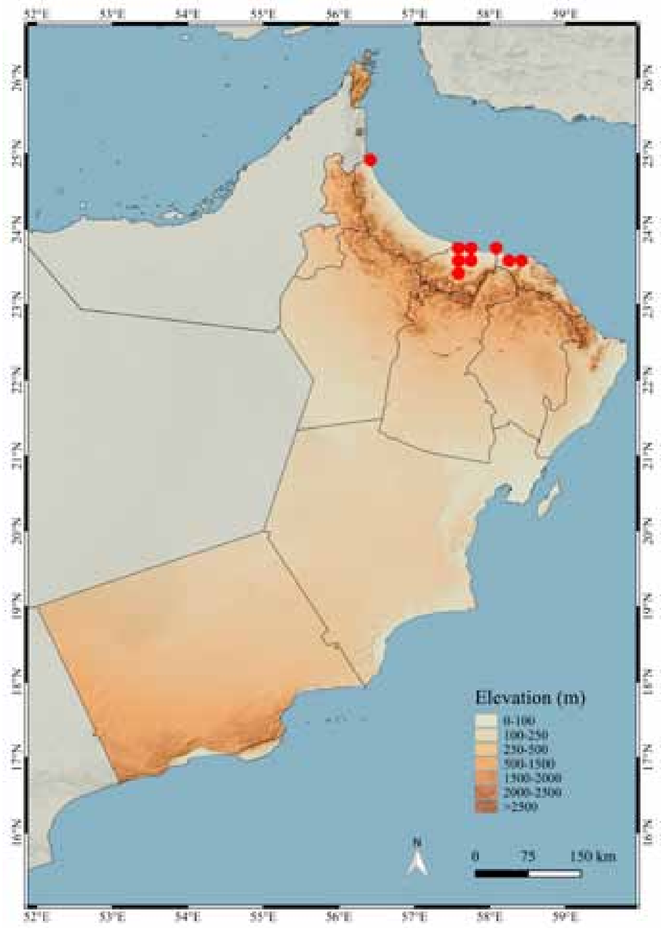

A

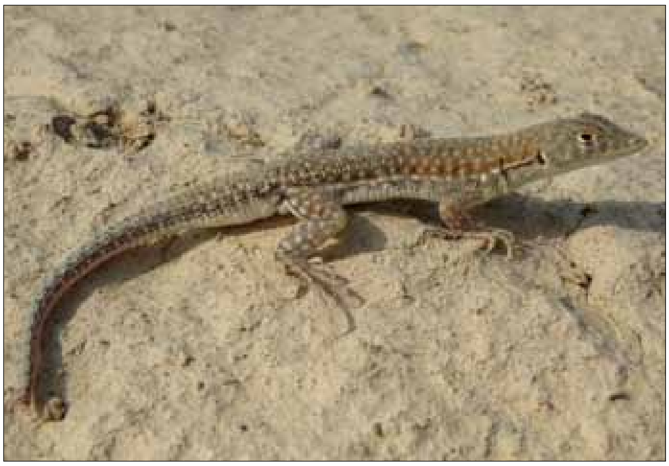

C

Useful information	
IUCN Category	LC*
Endemic	NO
Venomous	NO
Insular	NO
Present in a protected area	YES

*Not available on the web

D

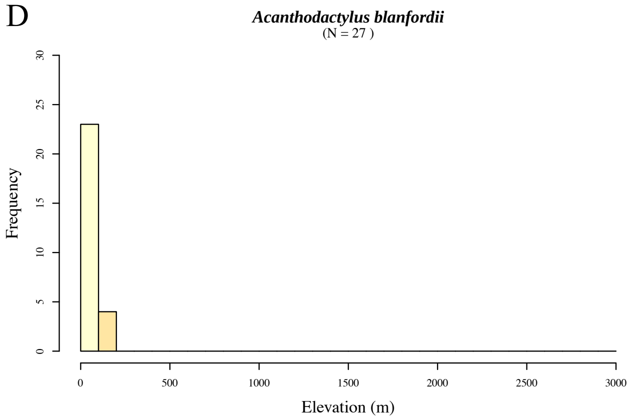

E

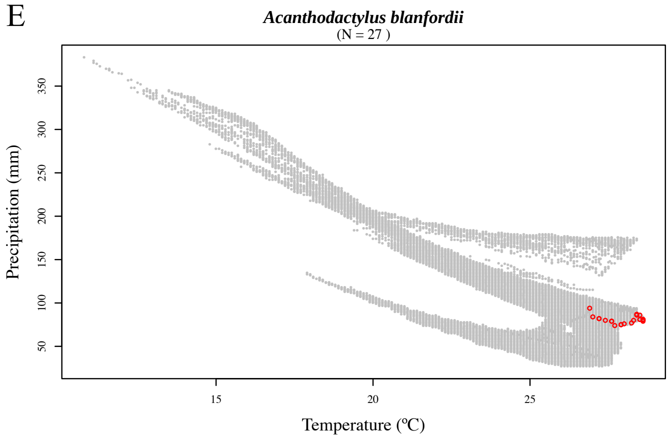

F

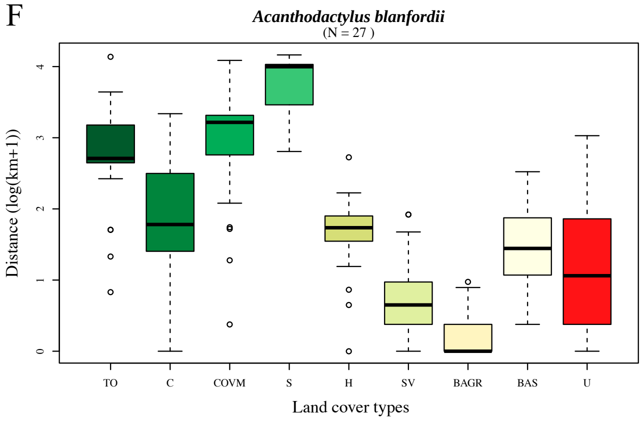

Acanthodactylus boskianus
(Daudin, 1802)

S60: Species information
Lizards
Lacertids, Lacertidae

A

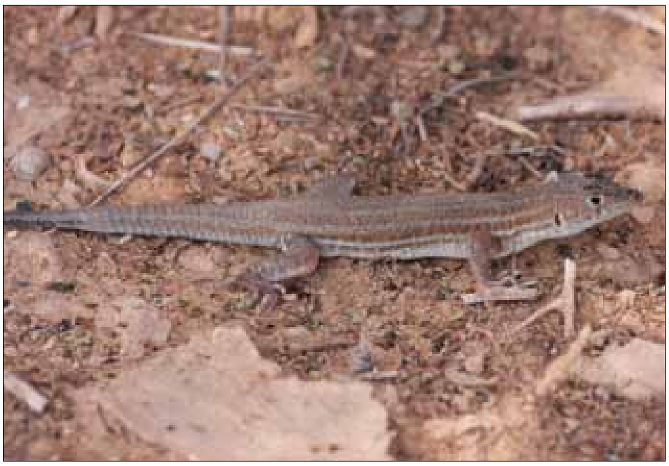

B

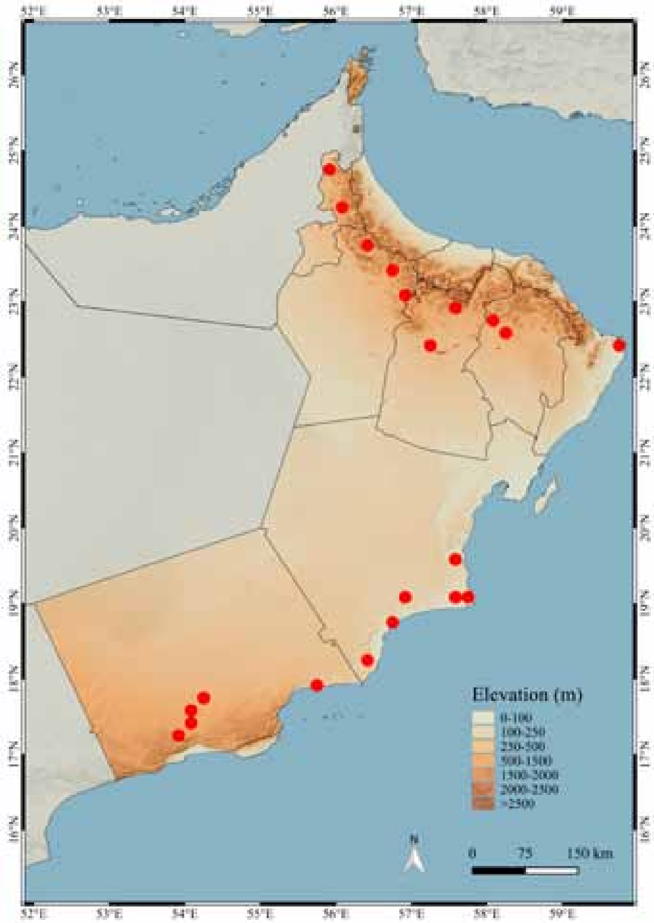

C

Useful information	
IUCN Category	LC*
Endemic	NO
Venomous	NO
Insular	NO
Present in a protected area	NO

*Not available on the web

D

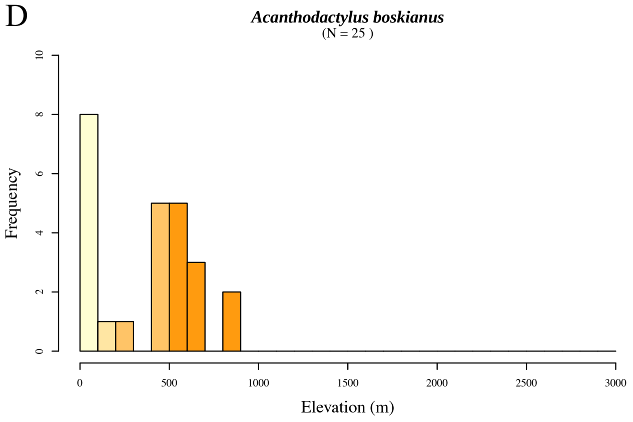

E

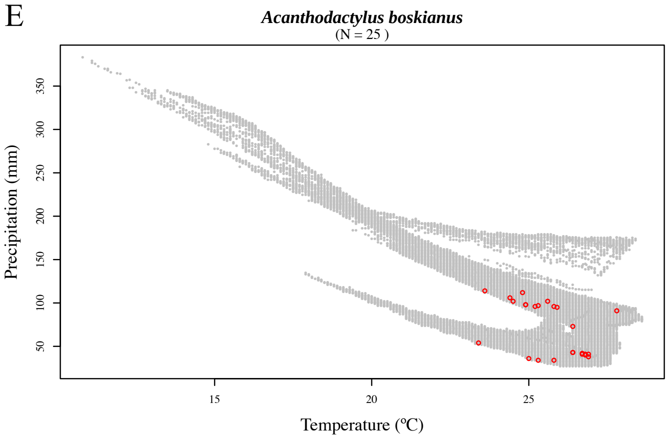

F

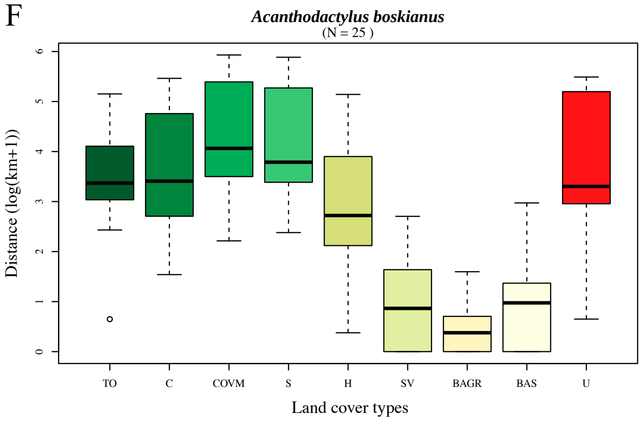

S61: Species information

Lizards

Lacertids, Lacertidae

Acanthodactylus felicis

Arnold, 1980

A

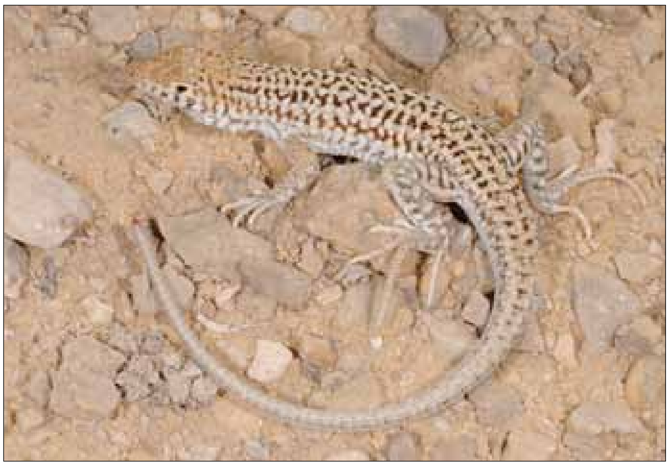

B

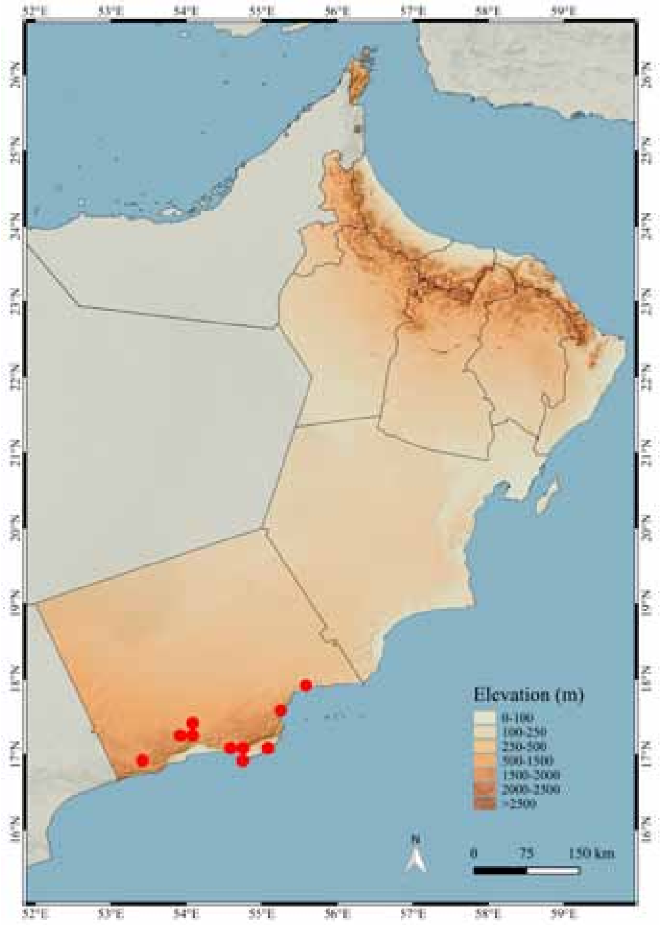

C

Useful information	
IUCN Category	VU
Endemic	NO
Venomous	NO
Insular	NO
Present in a protected area	YES

D

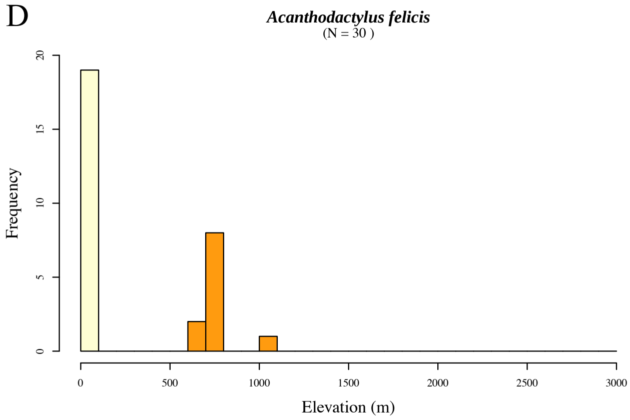

E

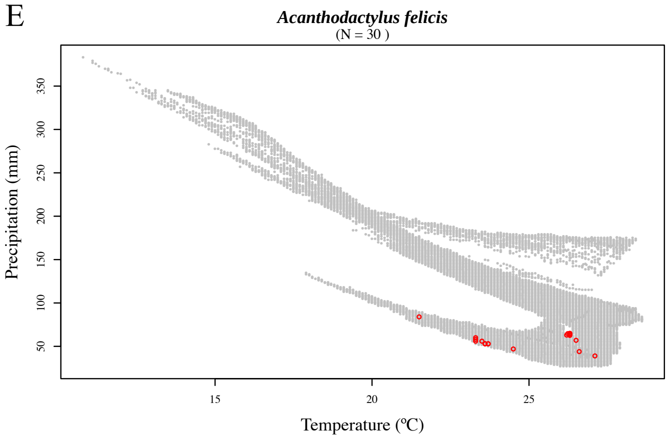

F

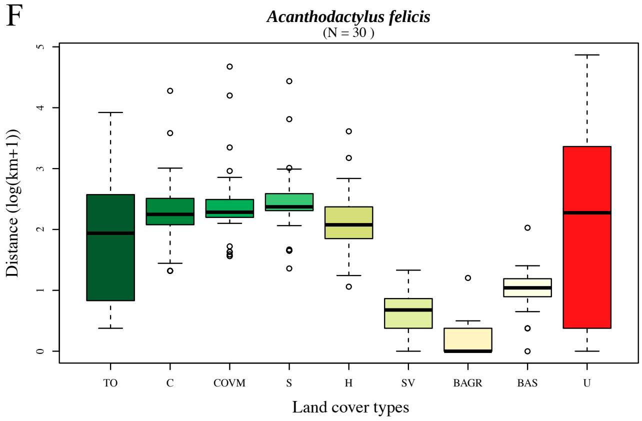

Acanthodactylus haasi
Leviton & Anderson, 1967

S62: Species information
Lizards
Lacertids, Lacertidae

A

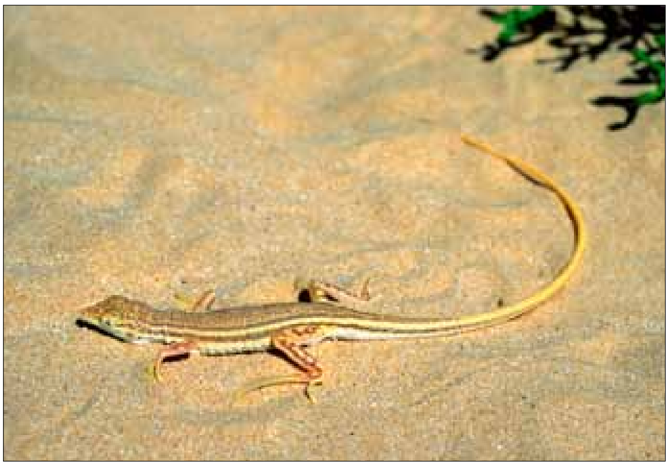

B

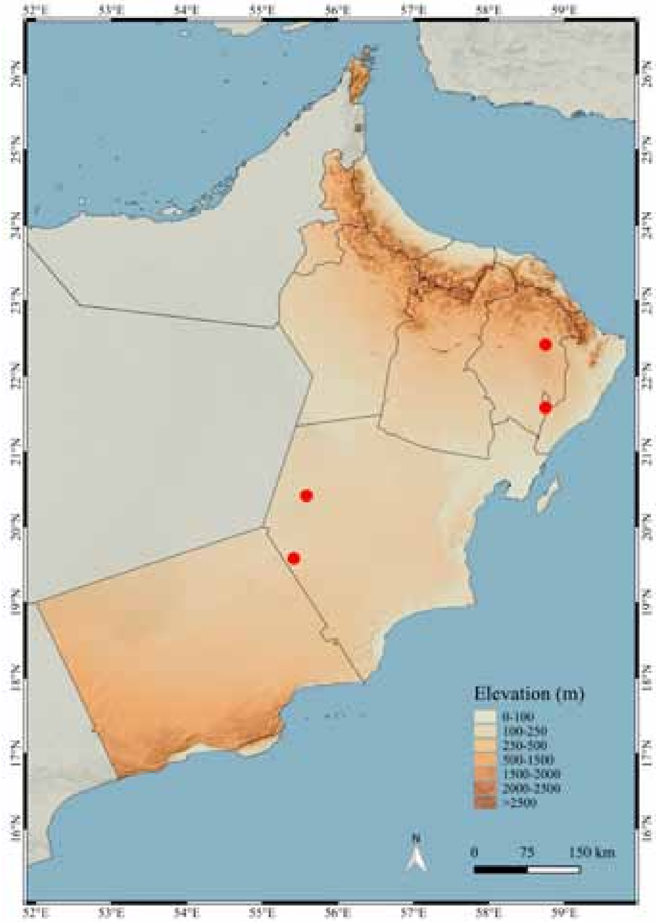

C

Useful information	
IUCN Category	LC
Endemic	NO
Venomous	NO
Insular	NO
Present in a protected area	NO

D

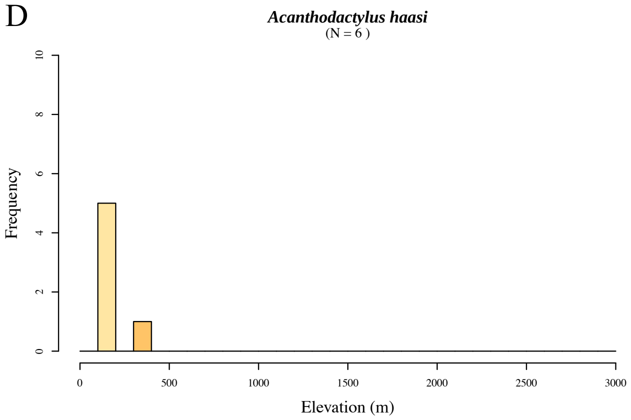

E

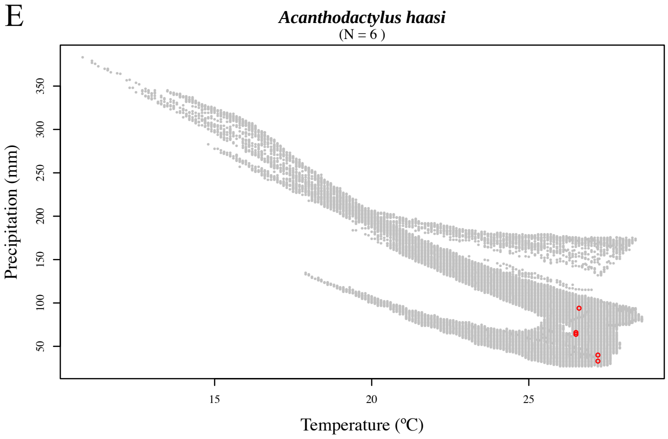

F

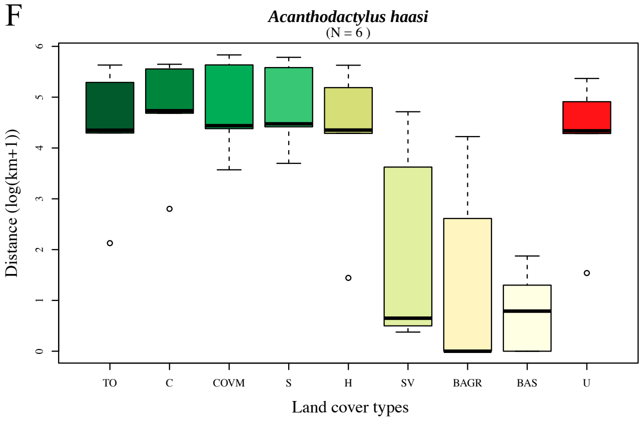

S63: Species information

Lizards

Lacertids, Lacertidae

Acanthodactylus masirae
Arnold, 1980

A

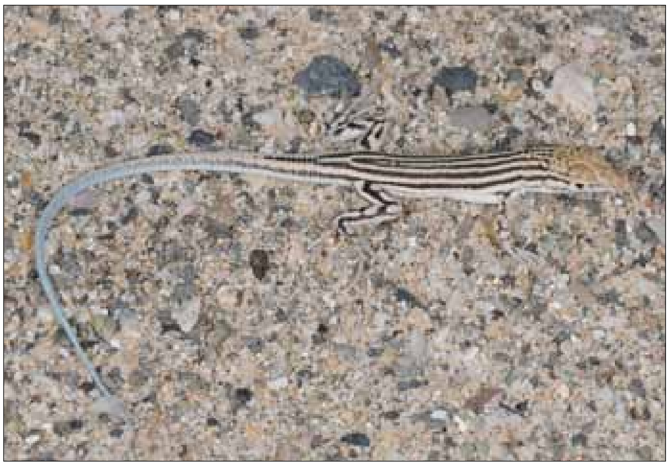

B

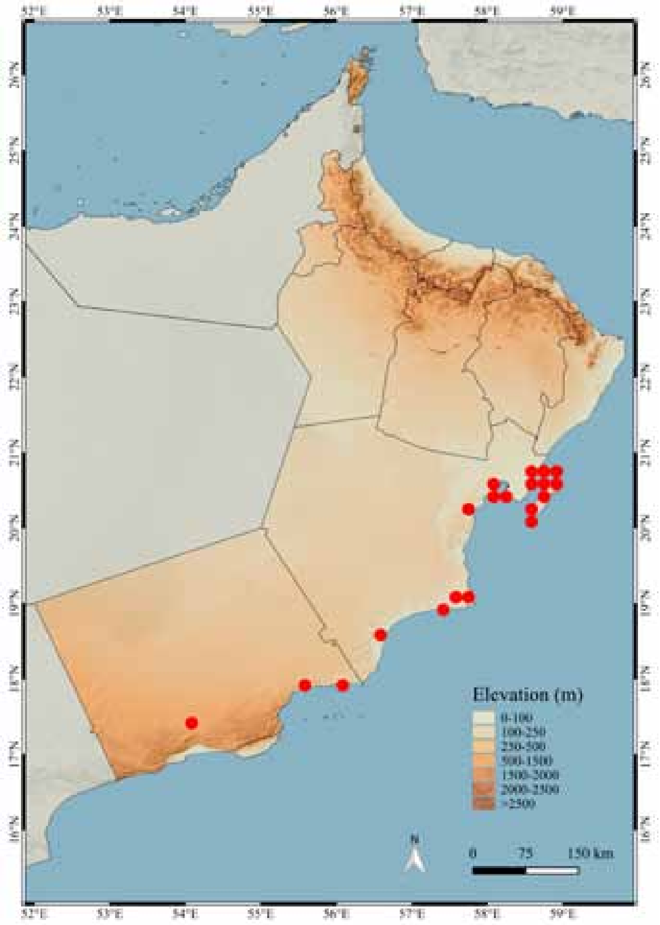

C

Useful information

IUCN Category	DD
Endemic	YES
Venomous	NO
Insular	YES
Present in a protected area	YES

D

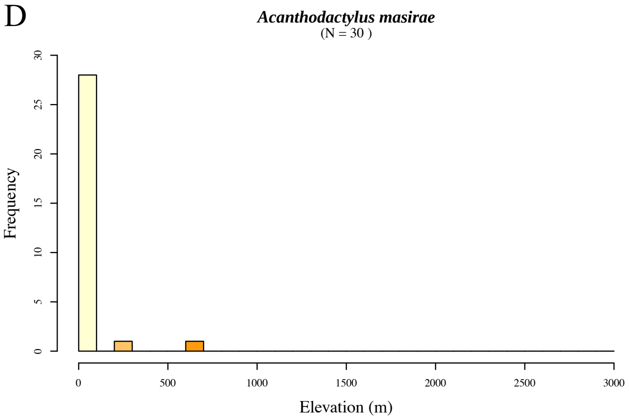

E

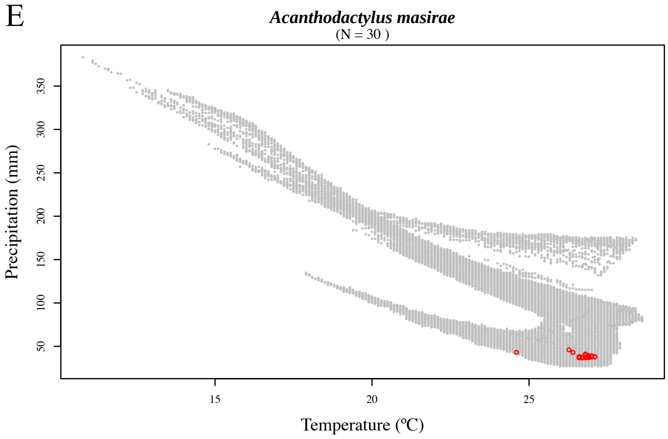

F

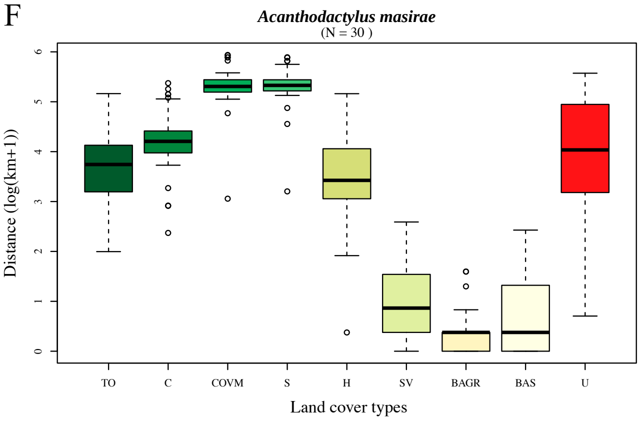

Acanthodactylus opheodurus
Arnold, 1980

S64: Species information
Lizards
Lacertids, Lacertidae

A

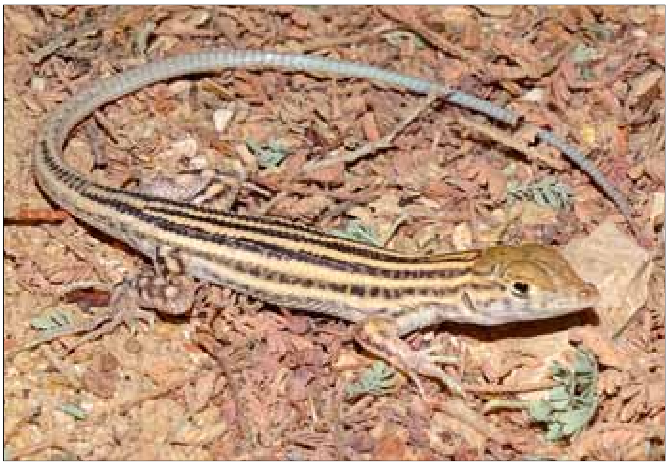

B

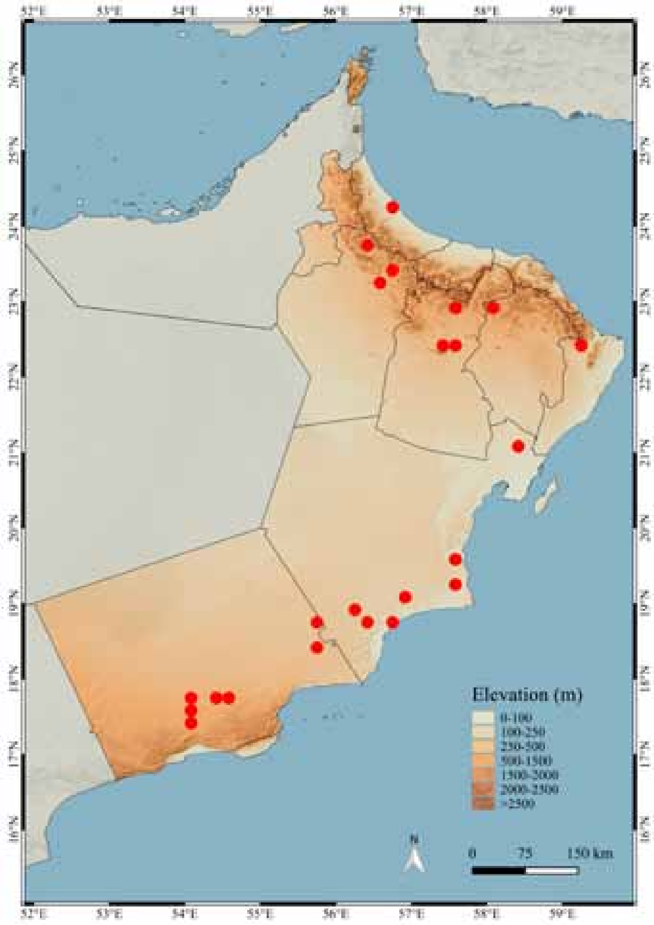

C

Useful information	
IUCN Category	LC
Endemic	NO
Venomous	NO
Insular	NO
Present in a protected area	NO

D

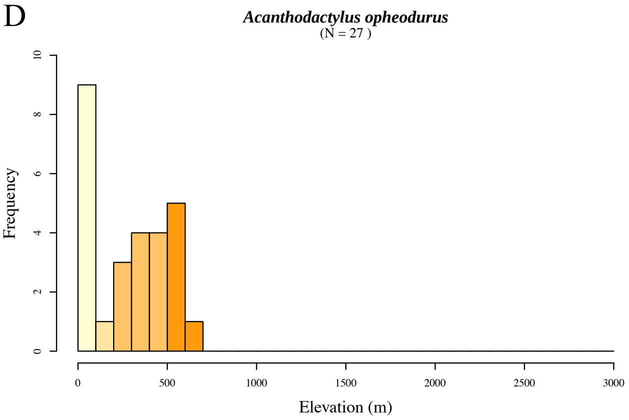

E

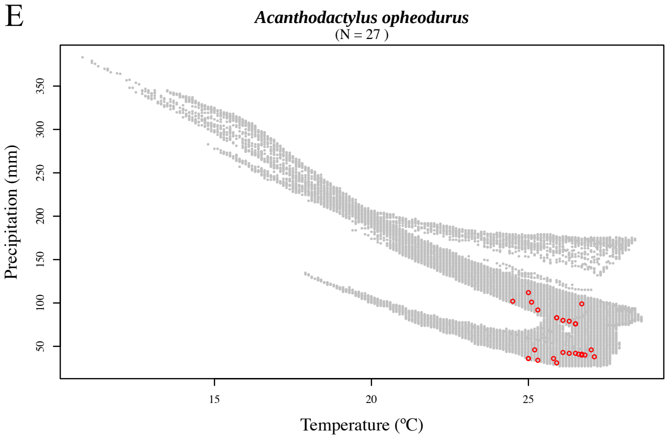

F

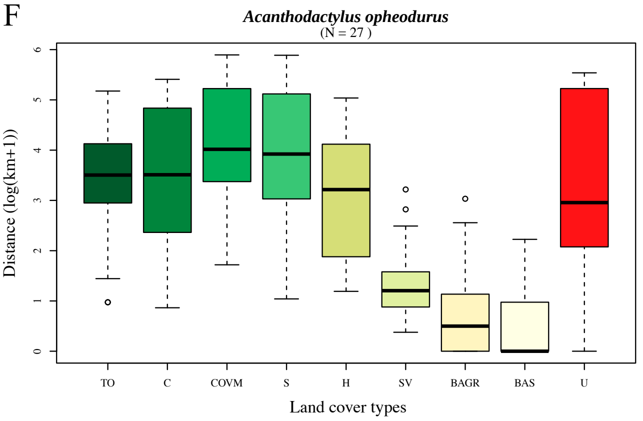

S65: Species information

Lizards

Lacertids, Lacertidae

Acanthodactylus schmidti

Haas, 1957

A

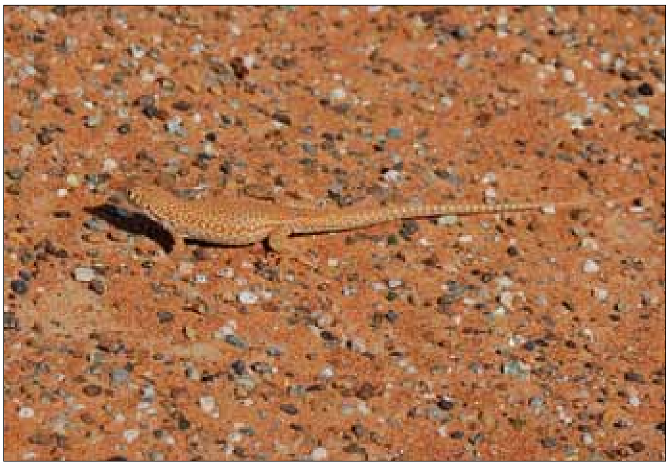

B

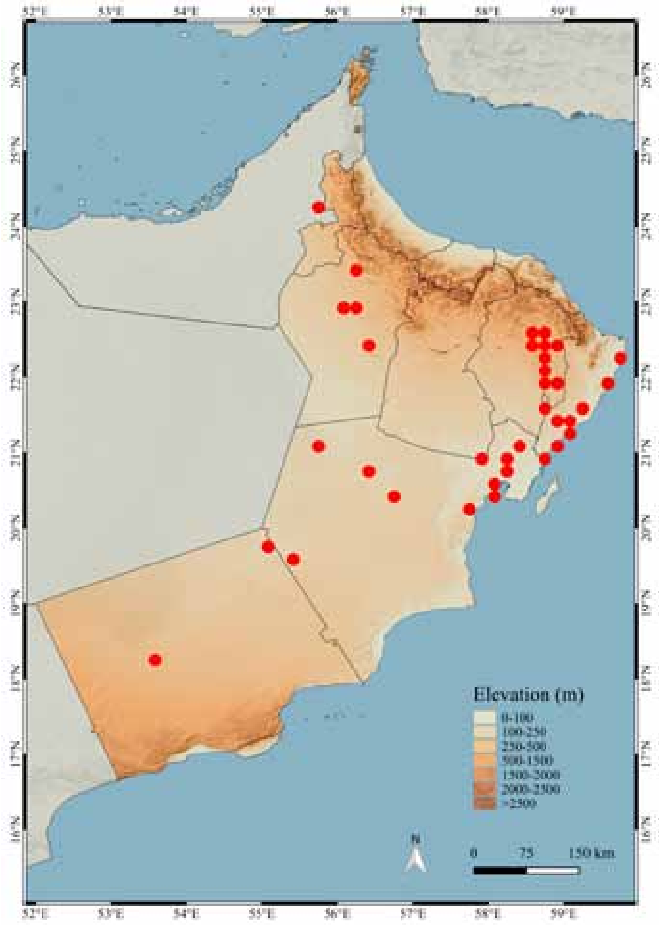

C

Useful information	
IUCN Category	LC
Endemic	NO
Venomous	NO
Insular	NO
Present in a protected area	YES

D

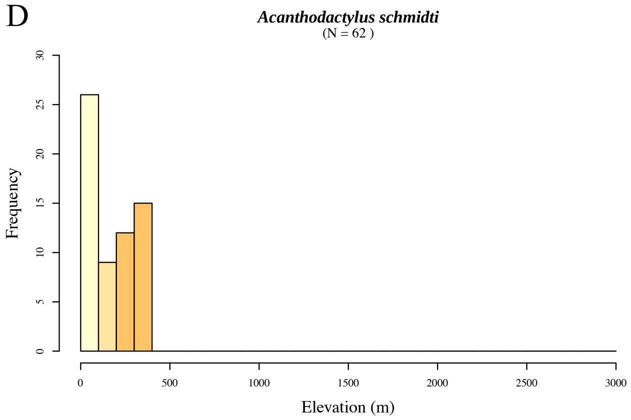

E

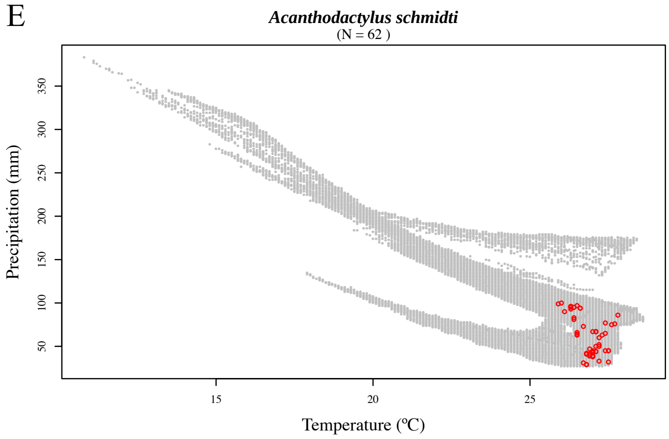

F

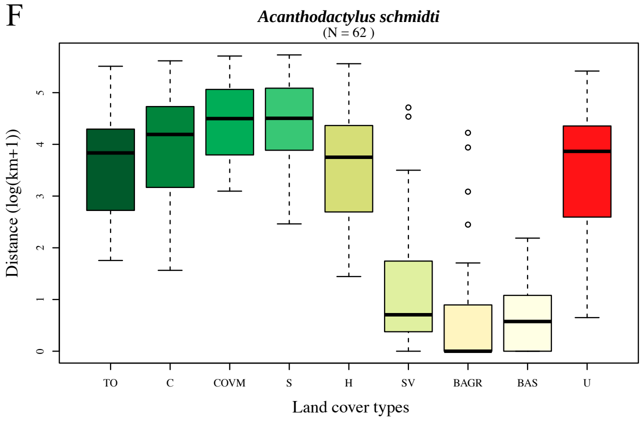

Mesalina adramitana
(Boulenger, 1917)

S66: Species information
Lizards
Lacertids, Lacertidae

A

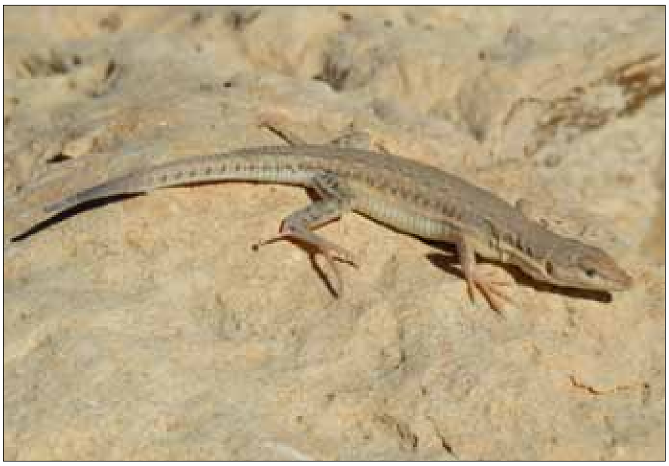

B

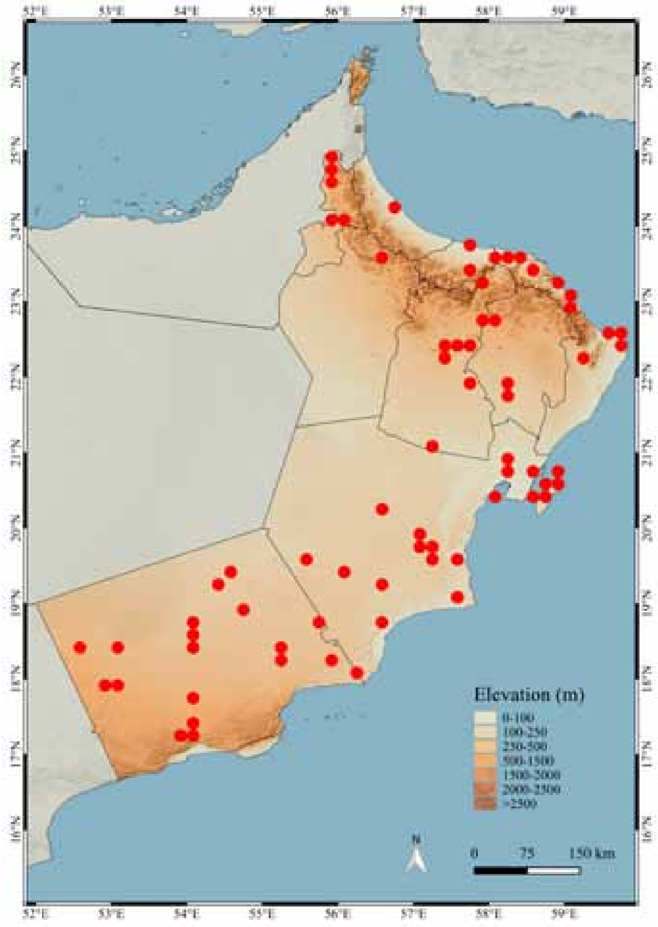

C

Useful information	
IUCN Category	LC
Endemic	NO
Venomous	NO
Insular	YES
Present in a protected area	YES

D

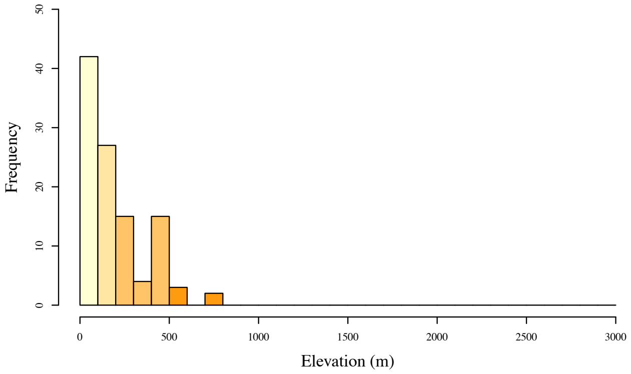

E

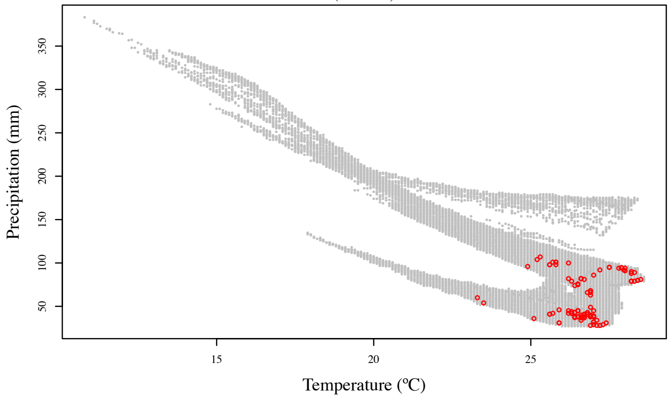

F

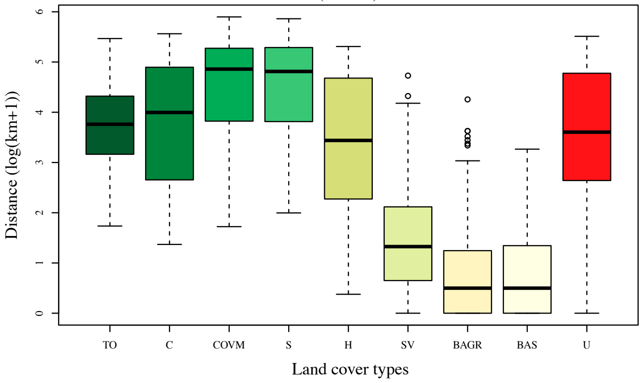

S67: Species information

Lizards

Lacertids, Lacertidae

Mesalina ayunensis

Arnold, 1980

A

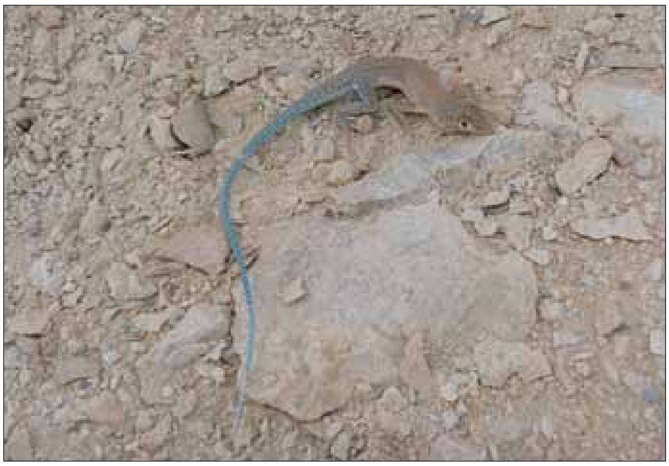

B

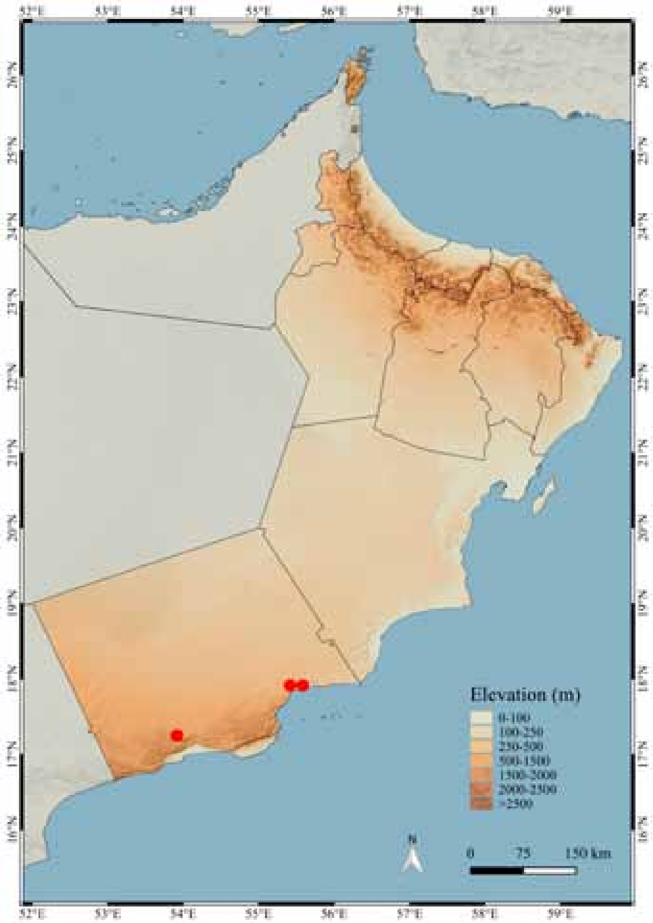

C

Useful information	
IUCN Category	DD
Endemic	NO
Venomous	NO
Insular	NO
Present in a protected area	NO

D

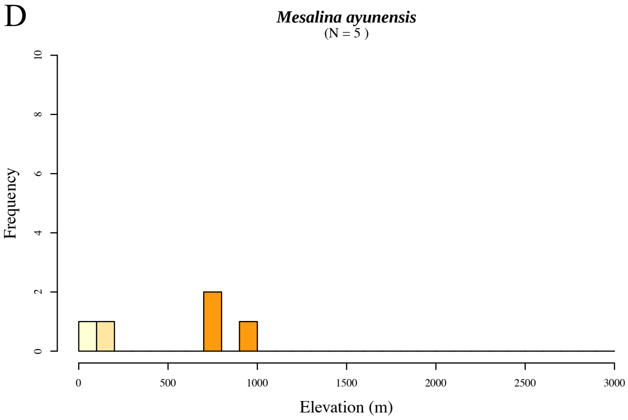

E

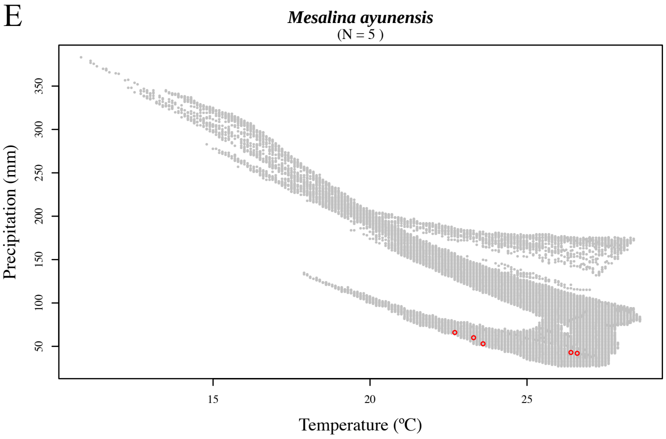

F

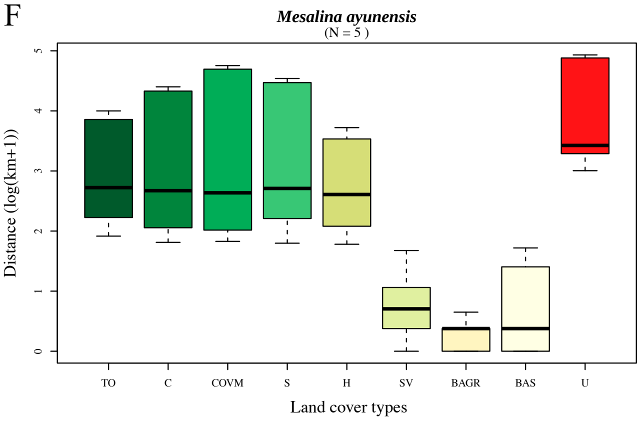

Lizards
Agamids
Geckos
Lacertids
Skinks
Amphisbaenids
Varanids
Snakes
Boidae
Colubridae
Elapidae
Lamprophiidae
Leptotyphlopidae
Typhlopidae
Viperidae

Mesalina sp. 1

Undescribed

S68: Species information

Lizards

Lacertids, Lacertidae

A

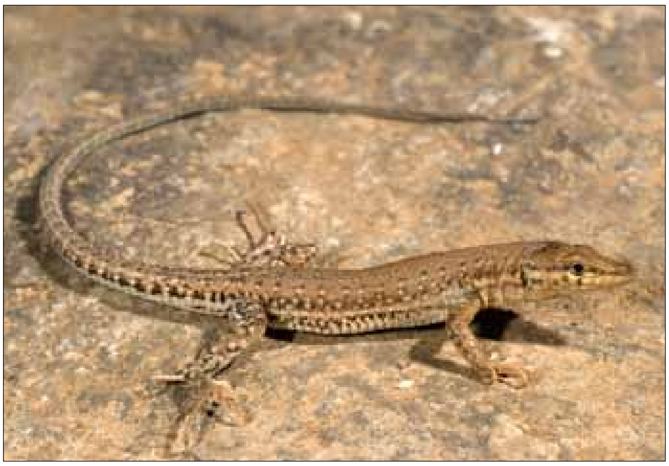

B

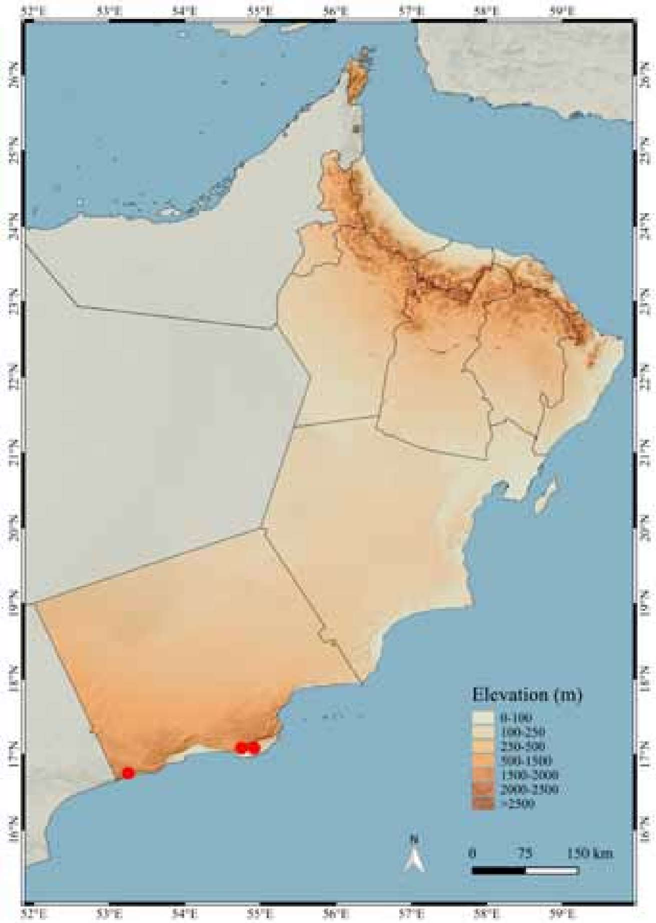

C

Useful information	
IUCN Category	NE
Endemic	NO
Venomous	NO
Insular	NO
Present in a protected area	YES

D

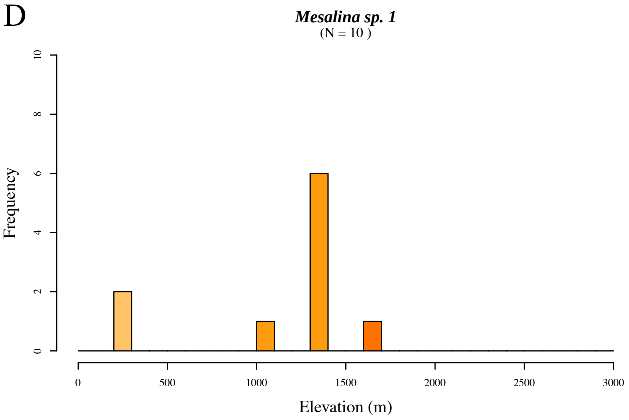

E

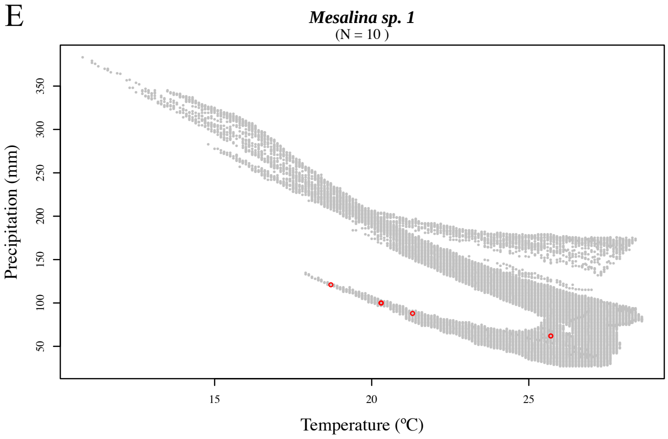

F

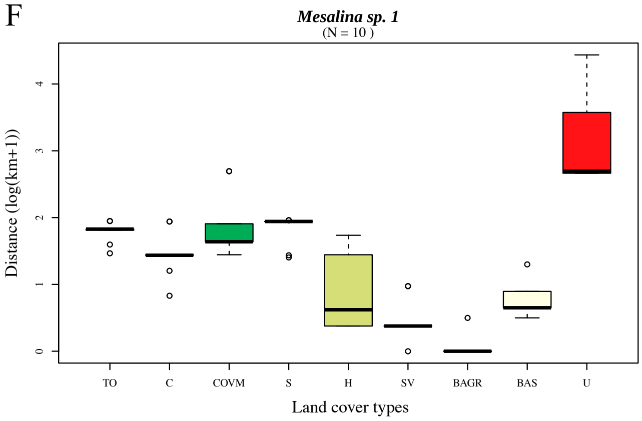

S69: Species information

Lizards

Lacertids, Lacertidae

Mesalina sp. 2

Undescribed

A

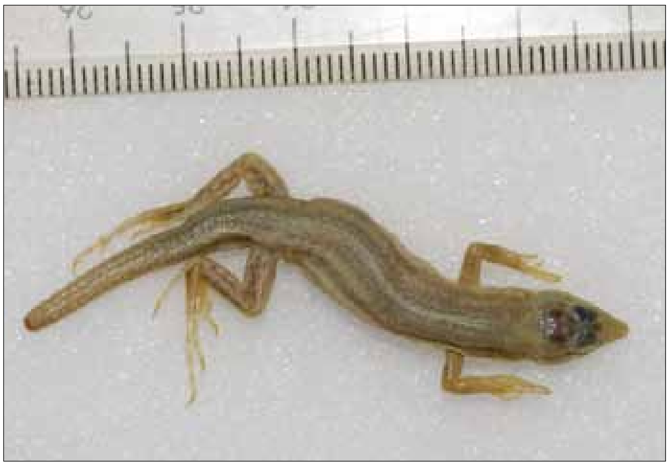

Specimen code: **BMNH1976.1470**

B

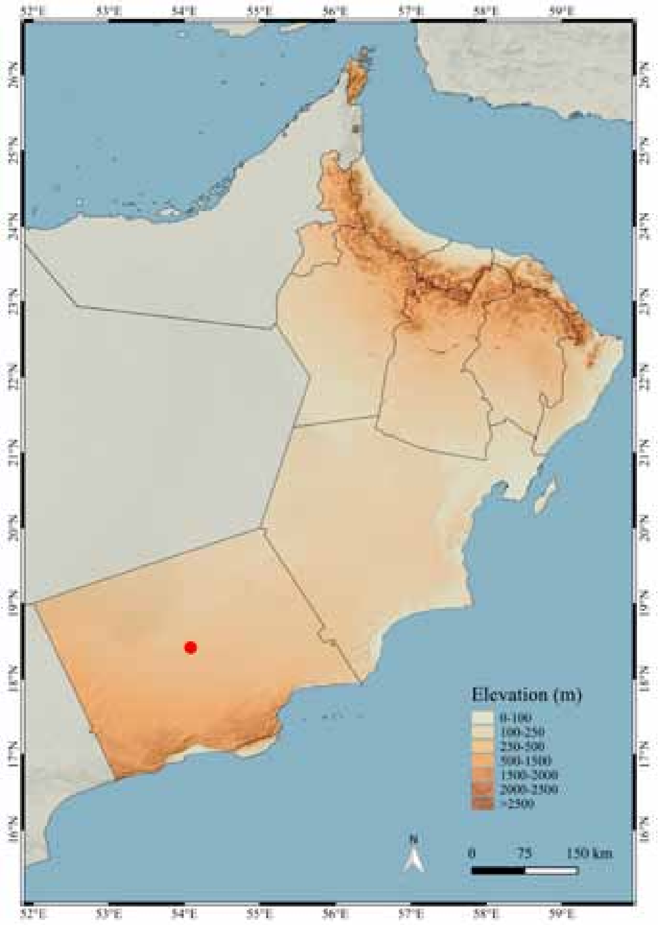

C

Useful information	
IUCN Category	NE
Endemic	YES
Venomous	NO
Insular	NO
Present in a protected area	NO

D

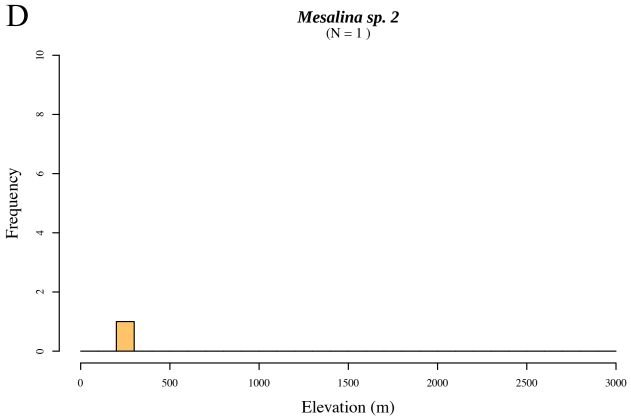

E

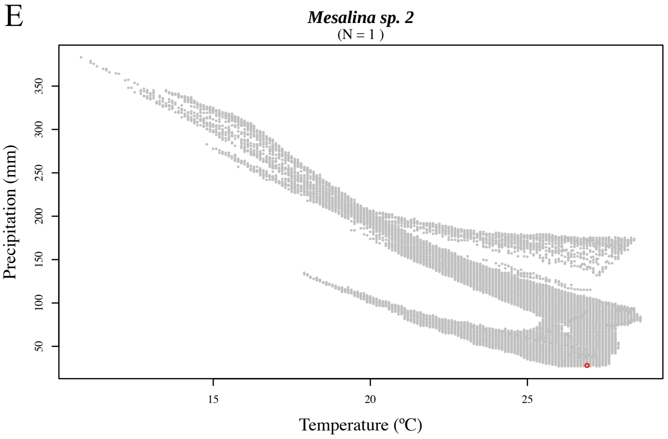

F

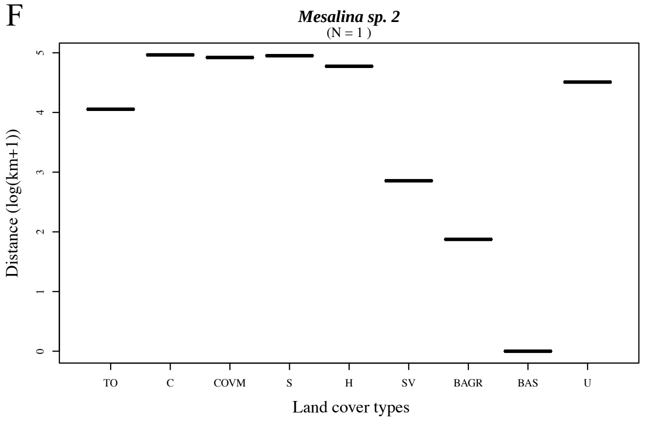

Omanosaura cyanura
(Arnold, 1972)

S70: Species information
Lizards
Lacertids, Lacertidae

A

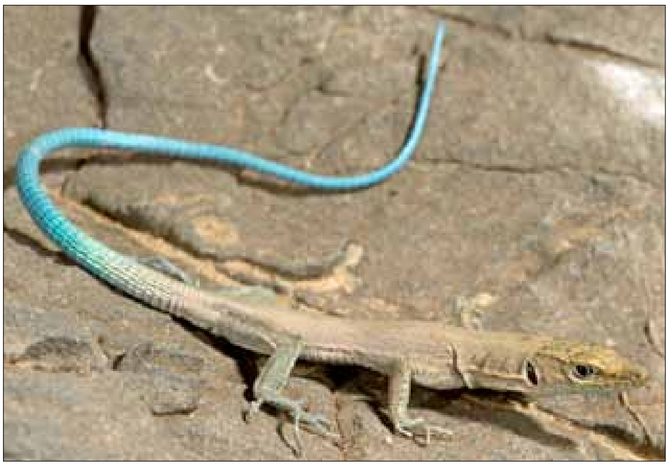

B

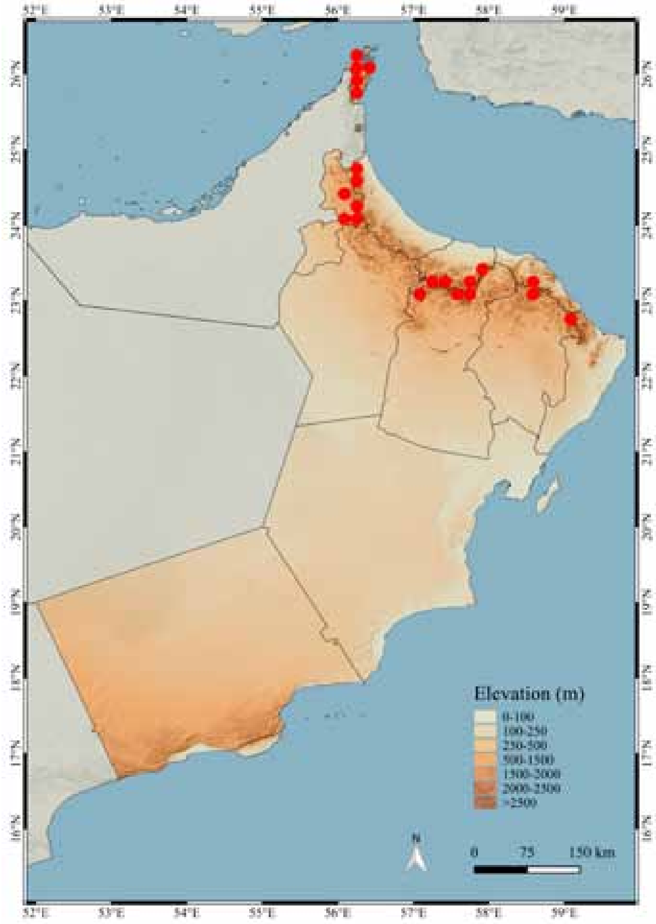

C

Useful information	
IUCN Category	LC
Endemic	NO
Venomous	NO
Insular	NO
Present in a protected area	YES

D

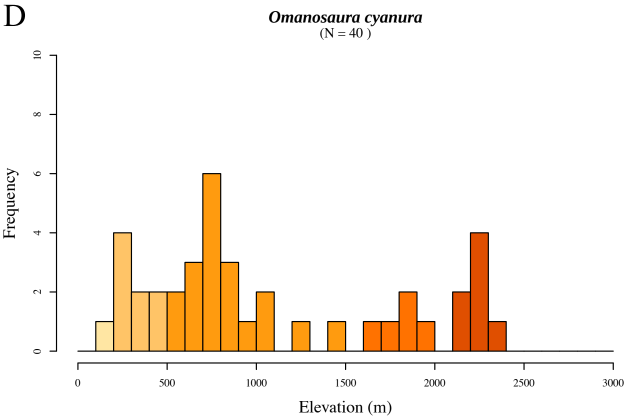

E

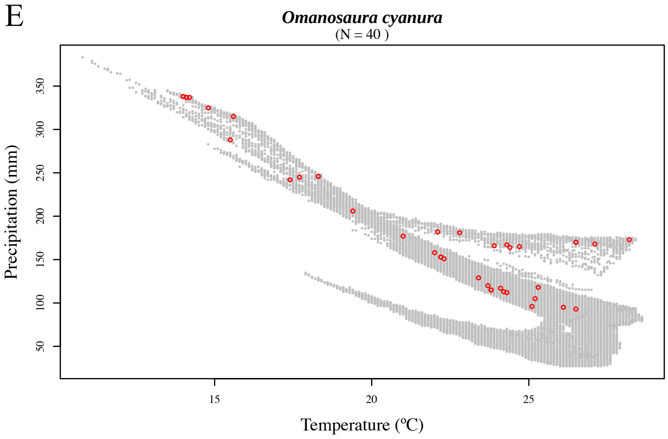

F

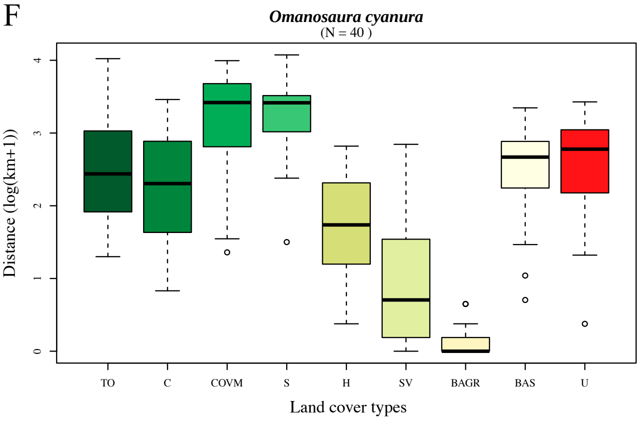

S71: Species information
Lizards
Lacertids, Lacertidae

Omanosaura jayakari
(Boulenger, 1887)

A

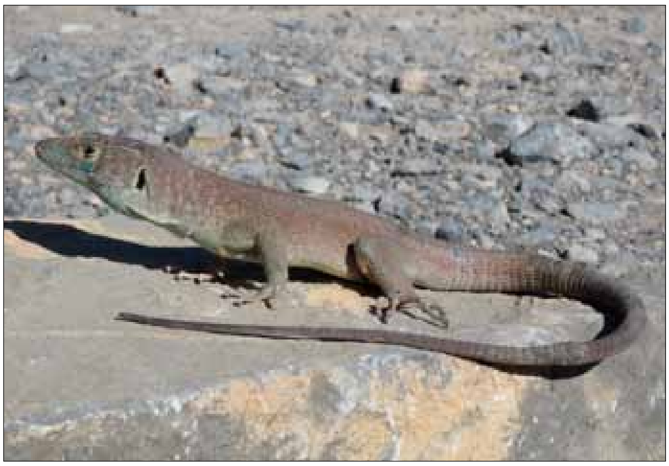

B

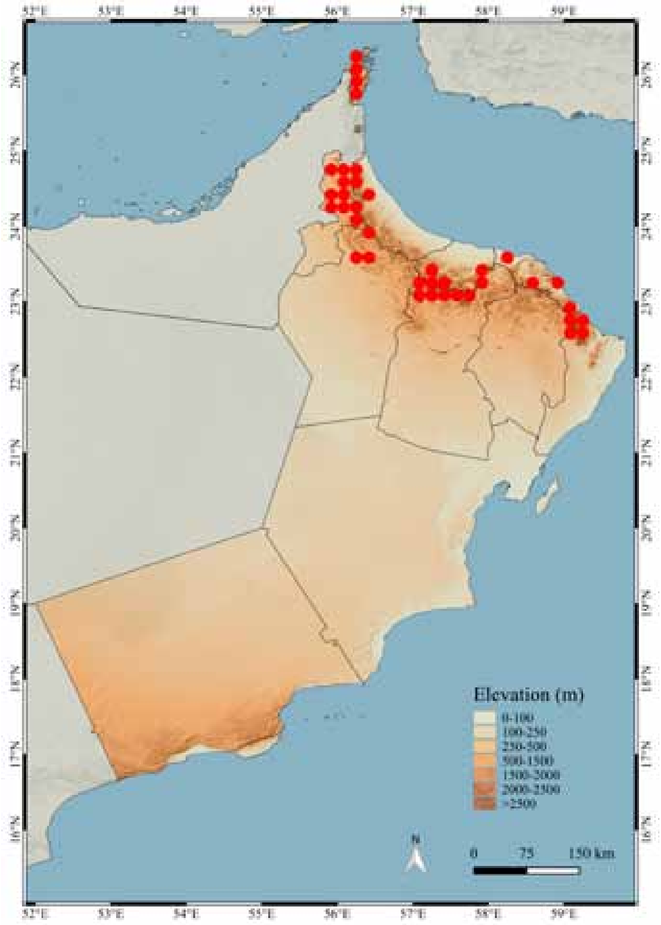

C

Useful information	
IUCN Category	LC
Endemic	NO
Venomous	NO
Insular	NO
Present in a protected area	YES

D

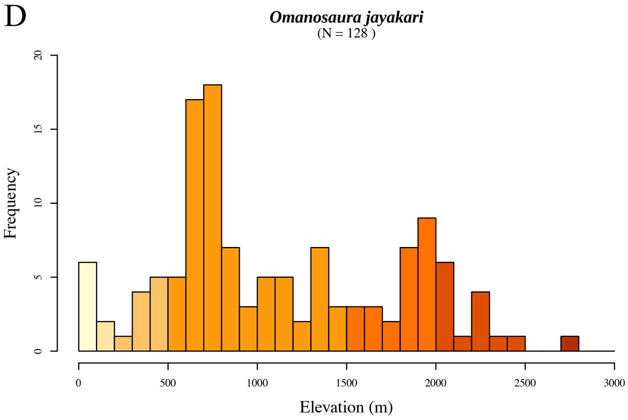

E

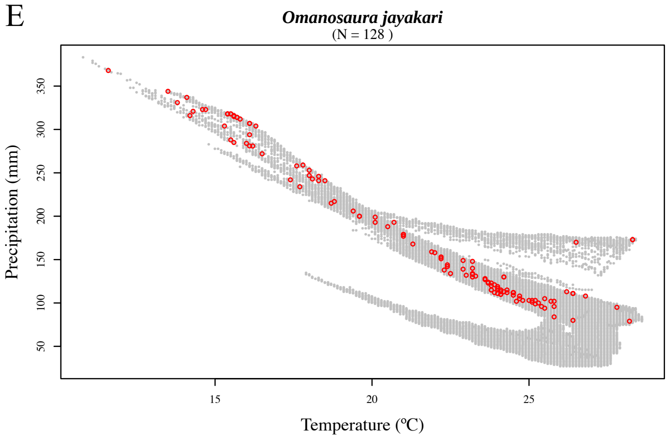

F

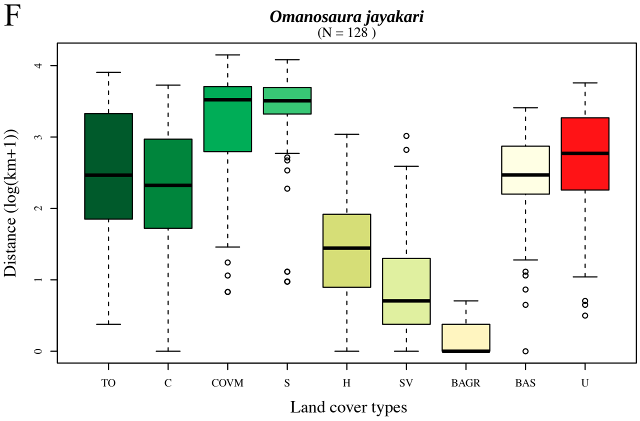

Ablepharus pannonicus
(Fitzinger, 1824)

S72: Species information
Lizards
Skinks, Scincidae

A

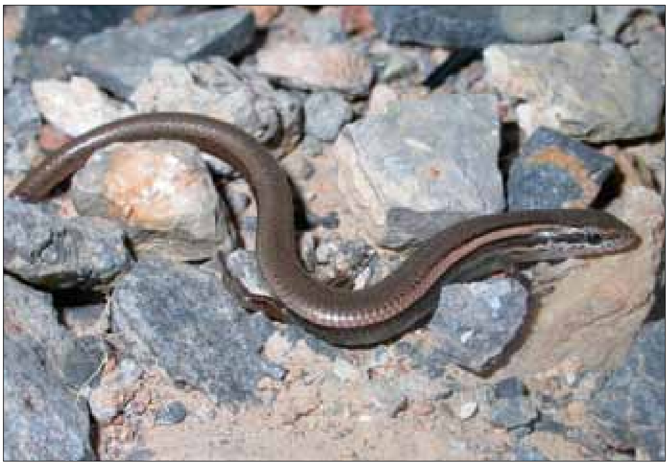

B

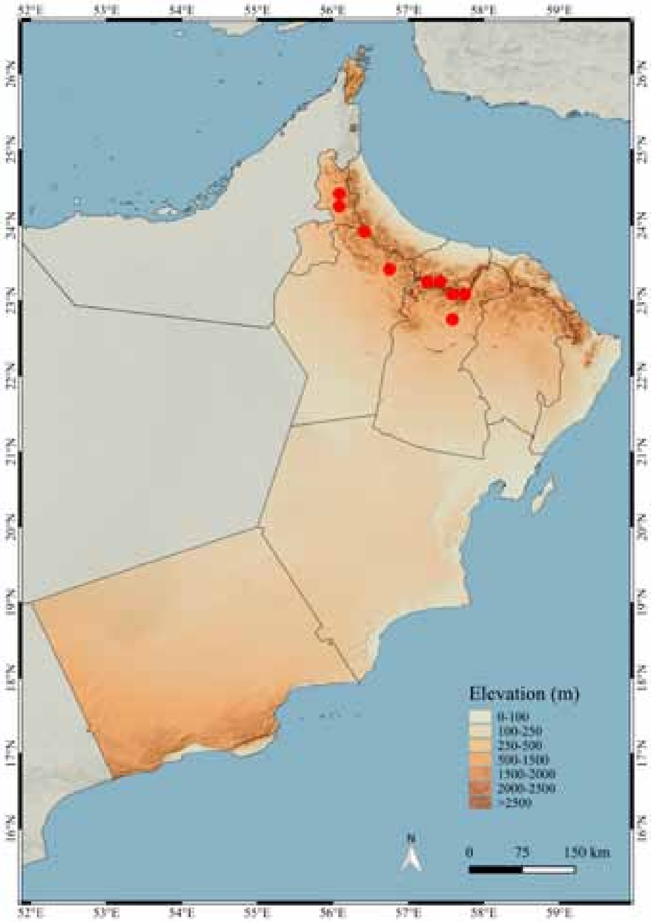

C

Useful information	
IUCN Category	LC*
Endemic	NO
Venomous	NO
Insular	NO
Present in a protected area	NO

*Not available on the web

D

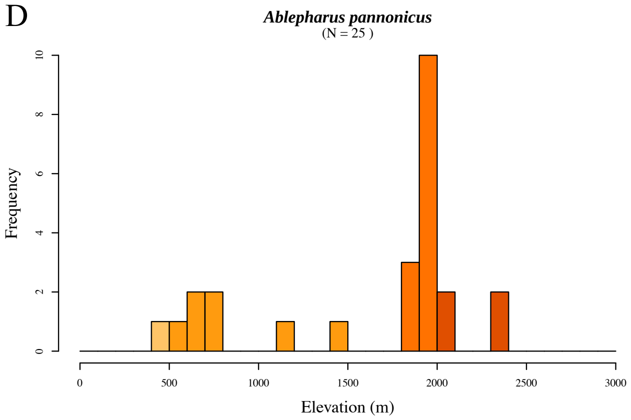

E

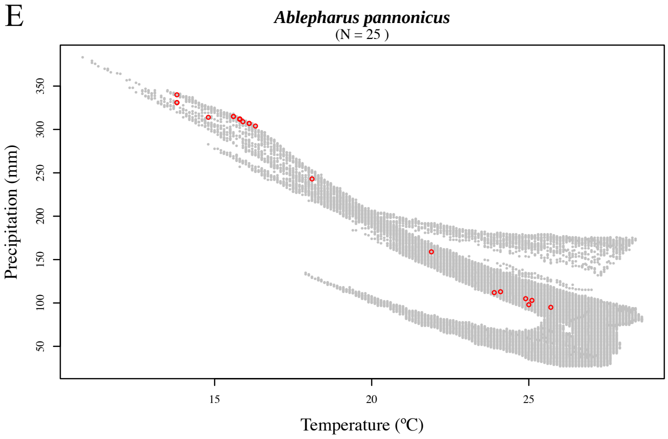

F

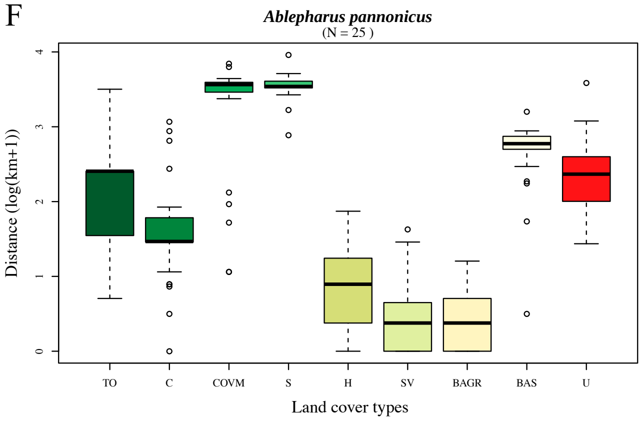

S73: Species information
Lizards
Skinks, Scincidae

Chalcides ocellatus ocellatus
(Forskal, 1775)

B

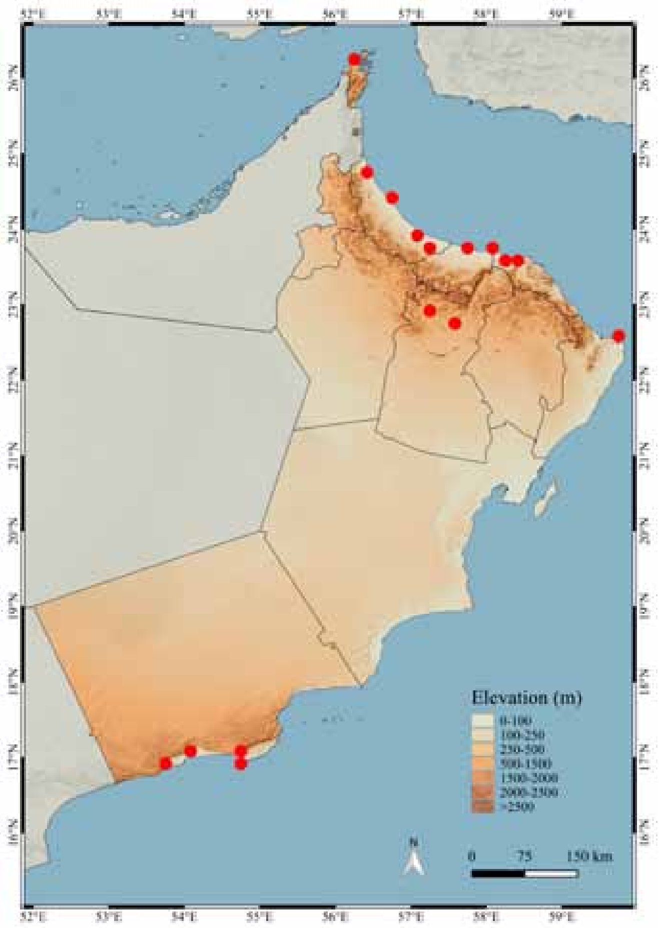

A

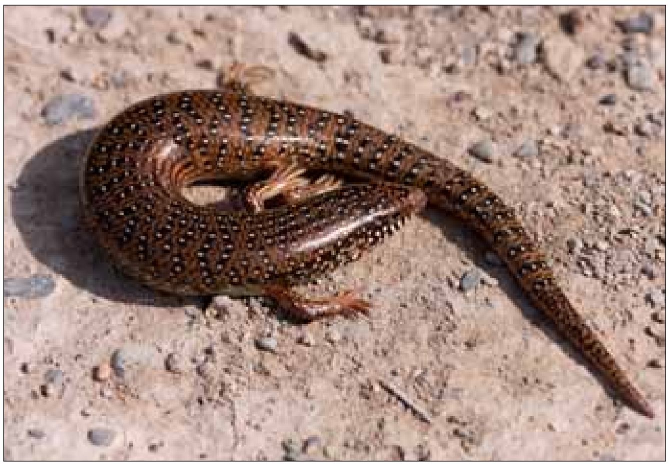

C

Useful information	
IUCN Category	LC*
Endemic	NO
Venomous	NO
Insular	YES
Present in a protected area	YES

*Not available on the web

D

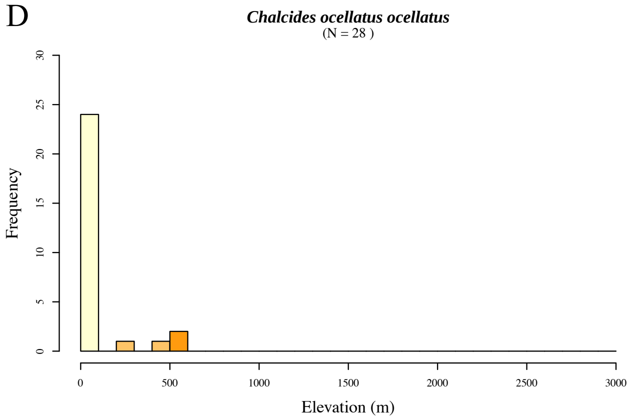

E

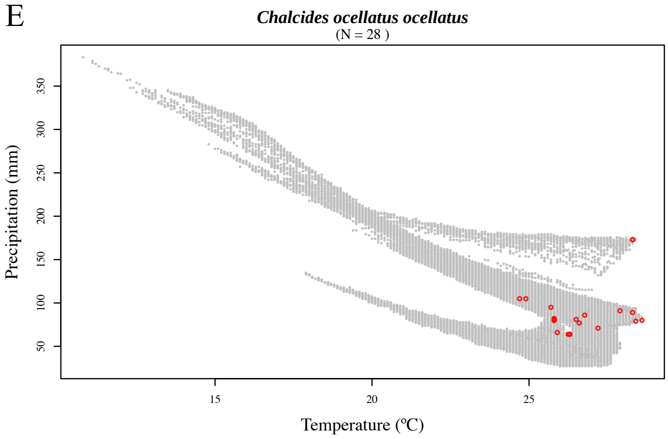

F

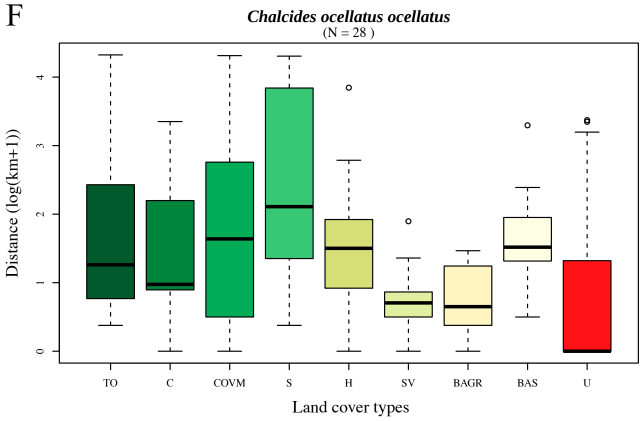

Heremites septemtaeniatus
(Reuss, 1834)

S74: Species information
Lizards
Skinks, Scincidae

A

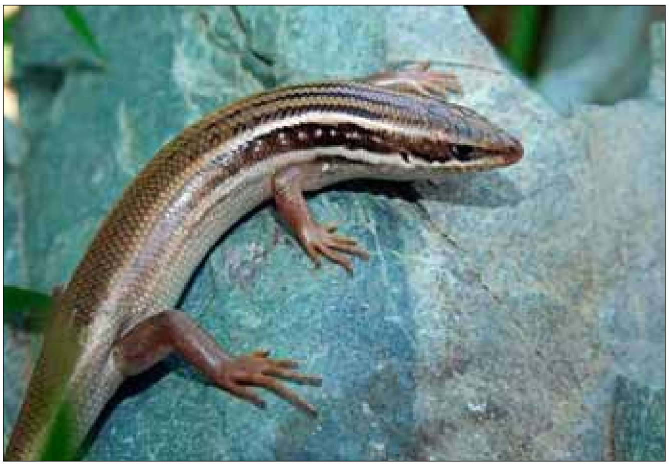

B

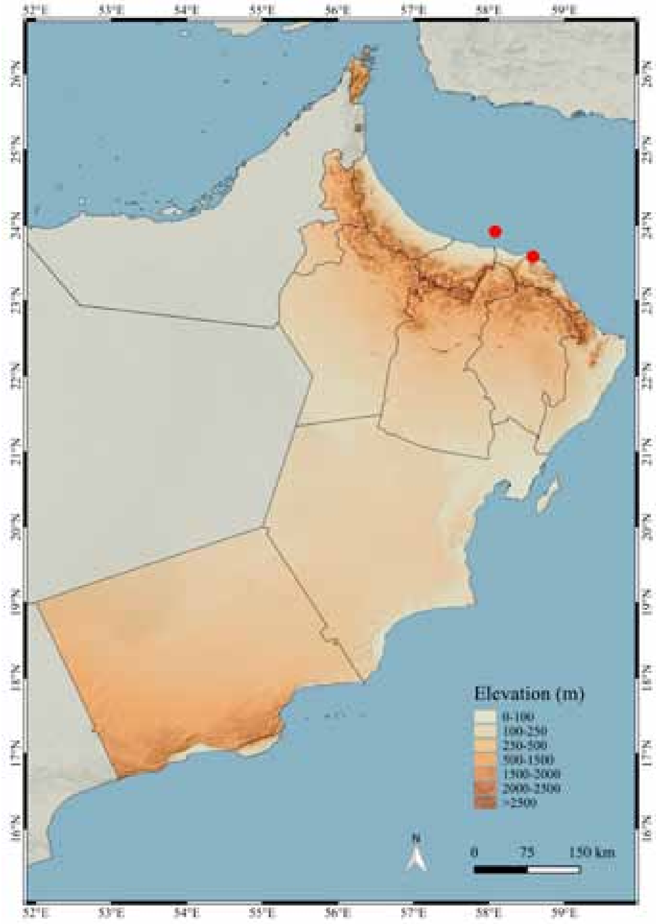

C

Useful information	
IUCN Category	LC*
Endemic	NO
Venomous	NO
Insular	YES
Present in a protected area	YES

*Not available on the web

D

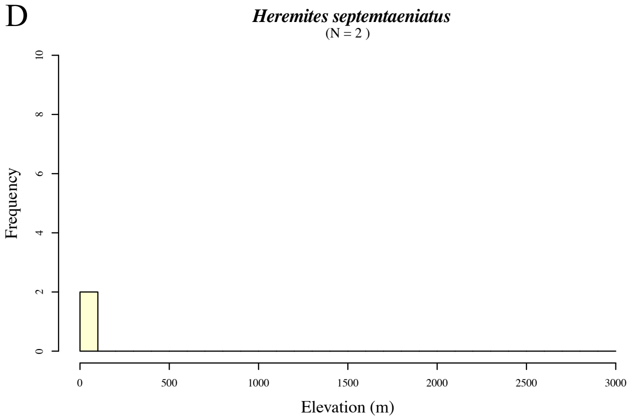

E

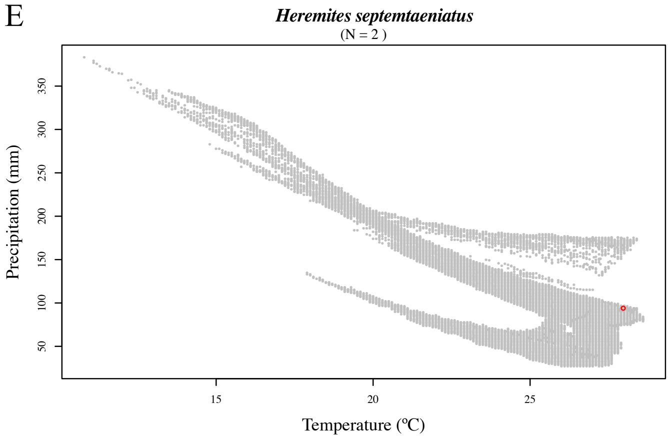

F

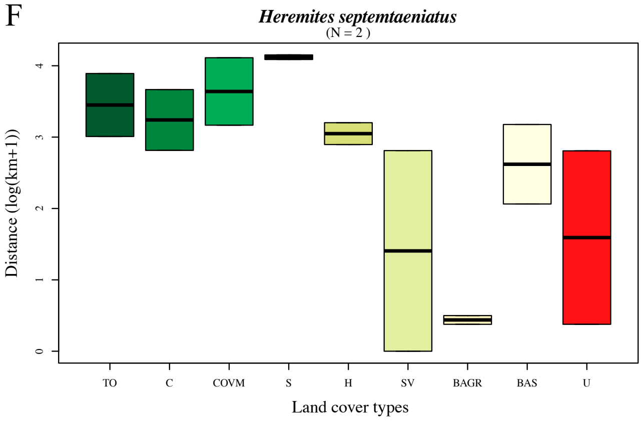

S75: Species information

Lizards

Skinks, Scincidae

Scincus mitranus
Anderson, 1871

A

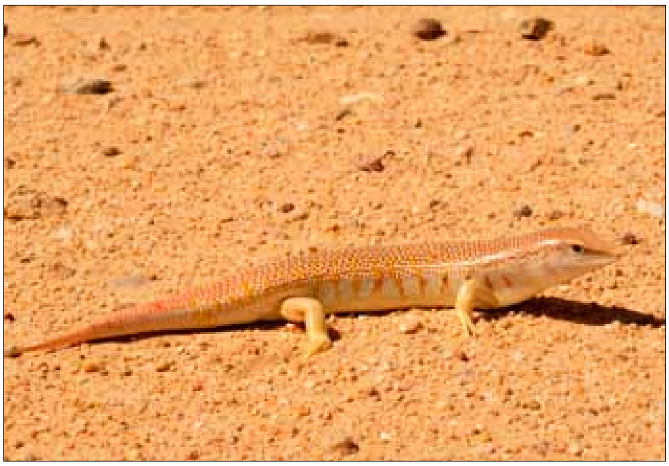

B

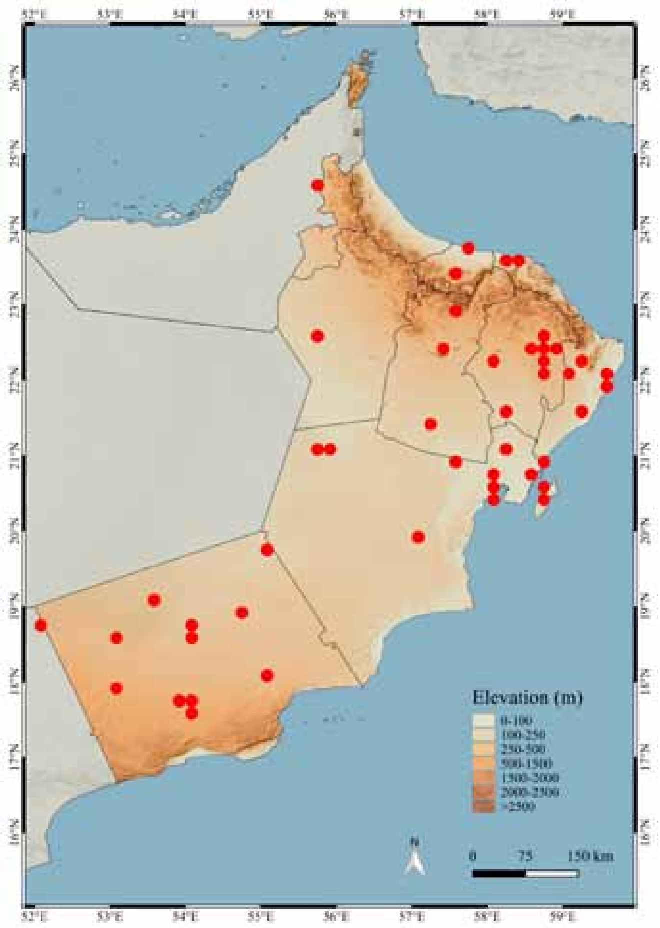

C

Useful information	
IUCN Category	LC
Endemic	NO
Venomous	NO
Insular	YES
Present in a protected area	YES

D

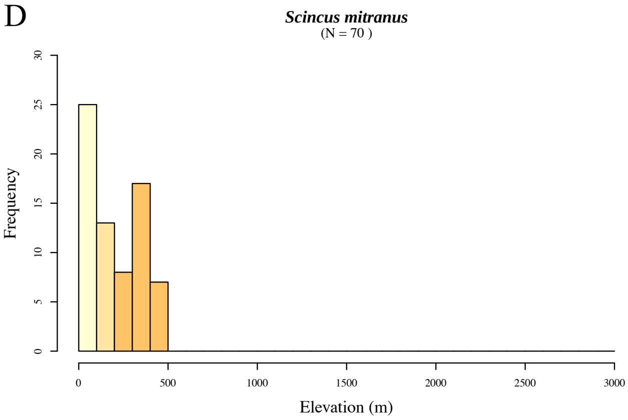

E

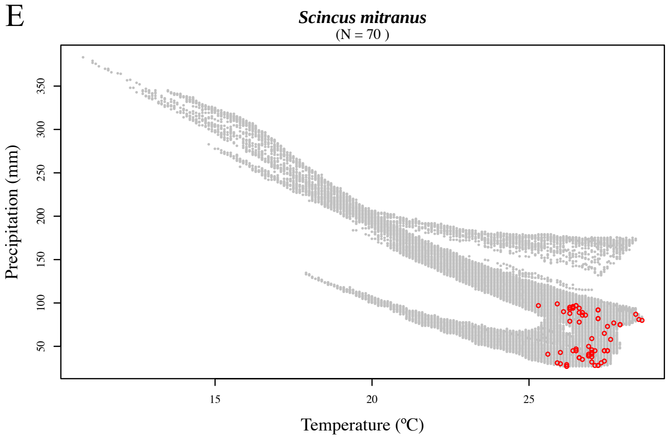

F

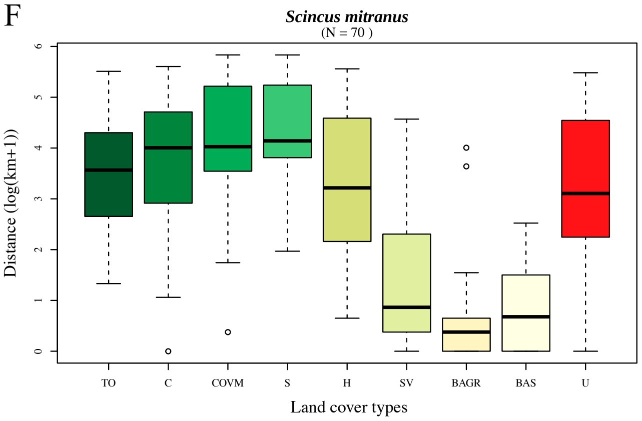

Scincus scincus conirostris
Blanford, 1881

S76: Species information
Lizards
Skinks, Scincidae

A

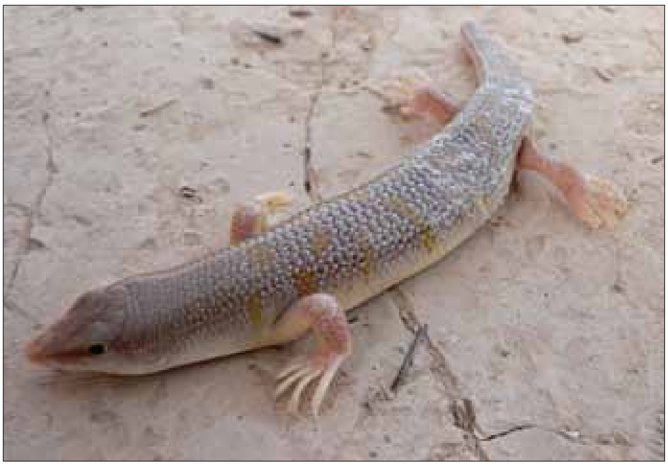

B

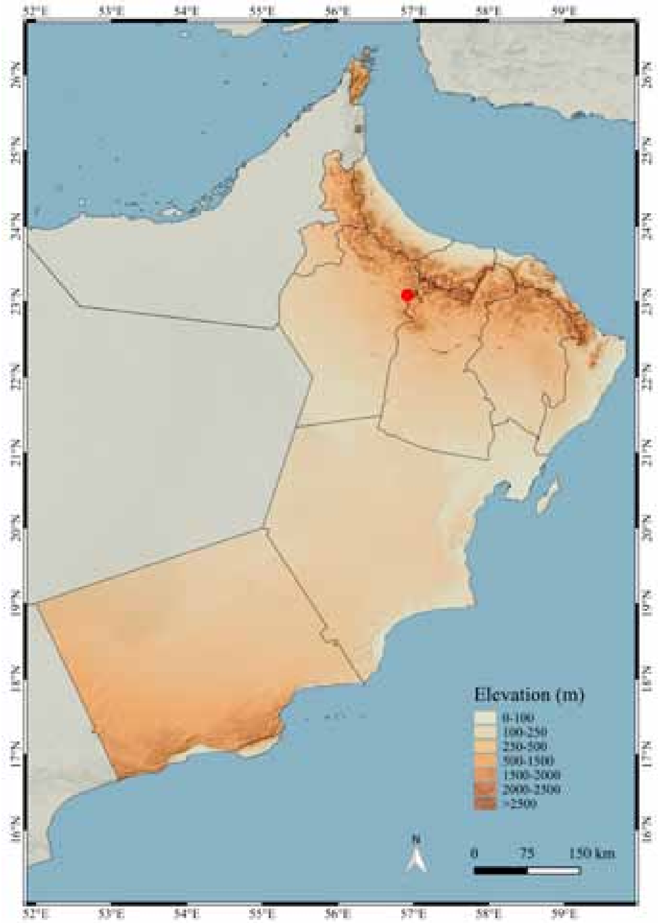

C

Useful information	
IUCN Category	LC*
Endemic	NO
Venomous	NO
Insular	NO
Present in a protected area	NO

*Not available on the web

D

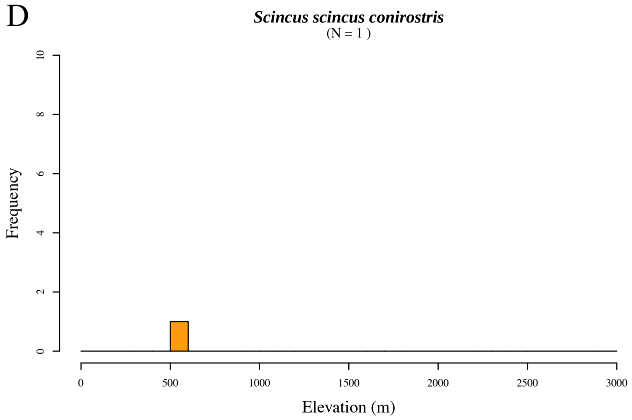

E

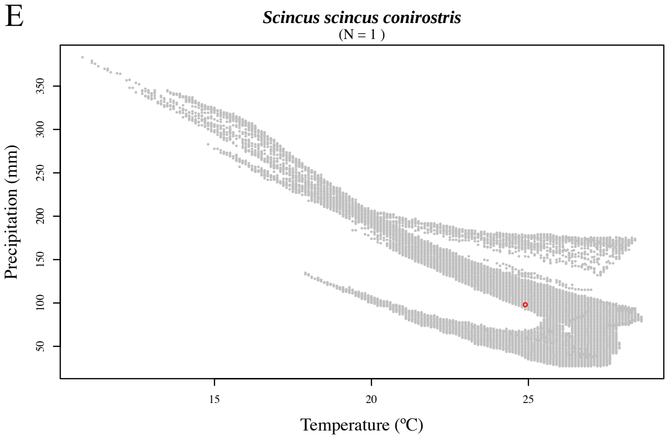

F

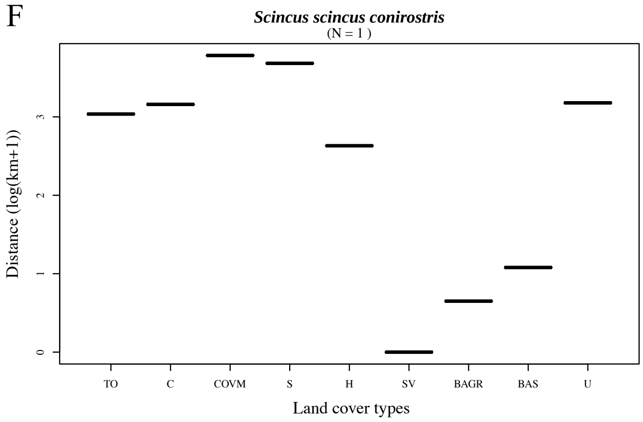

S77: Species information

Lizards

Skinks, Scincidae

Trachylepis brevicollis
(Wiegmann, 1837)

A

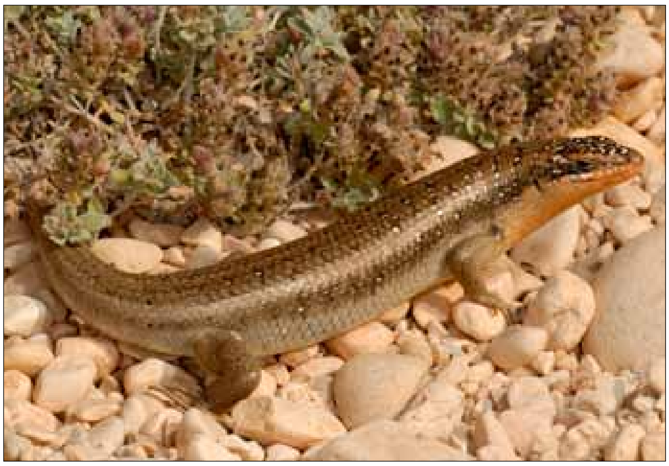

B

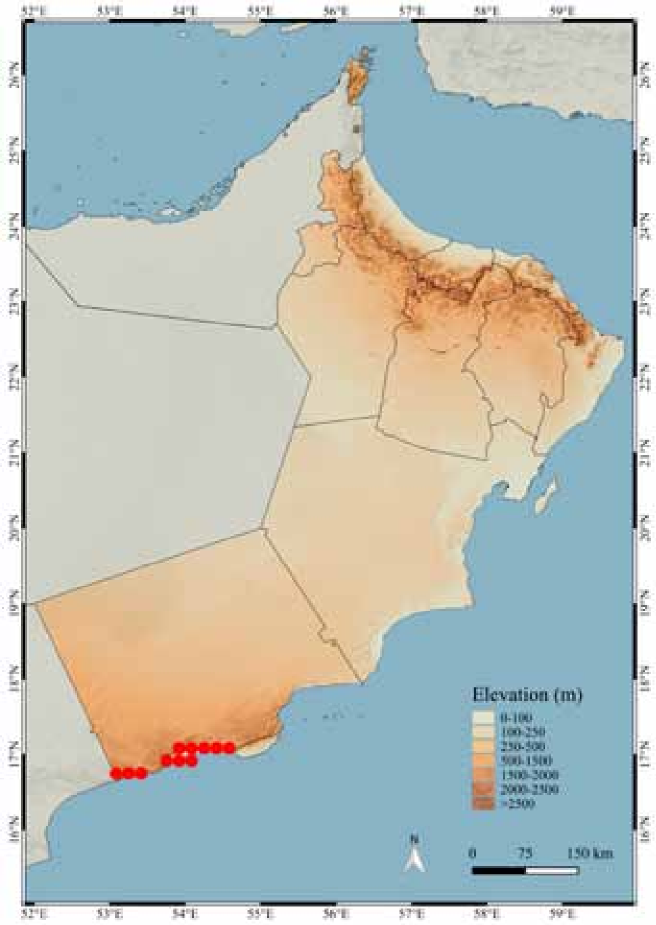

C

Useful information	
IUCN Category	LC*
Endemic	NO
Venomous	NO
Insular	NO
Present in a protected area	NO

*Not available on the web

D

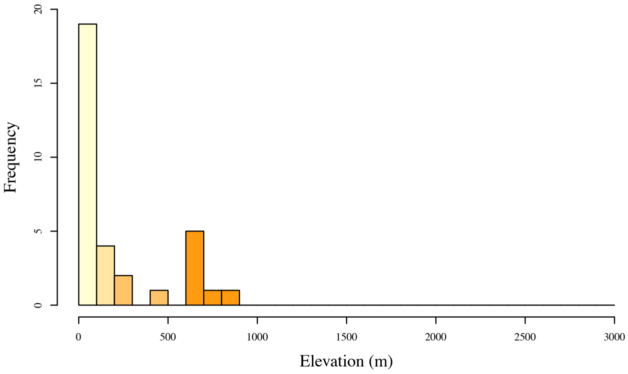

E

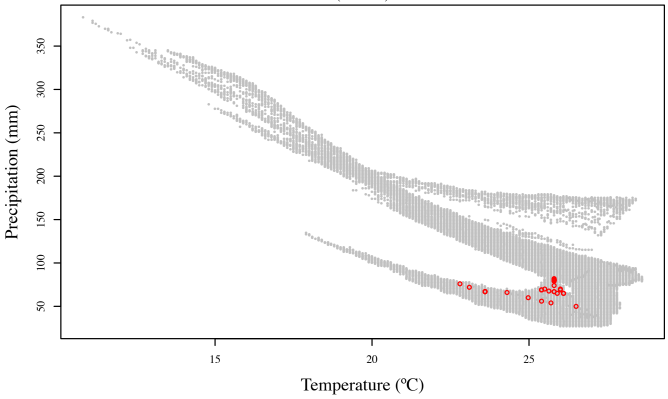

F

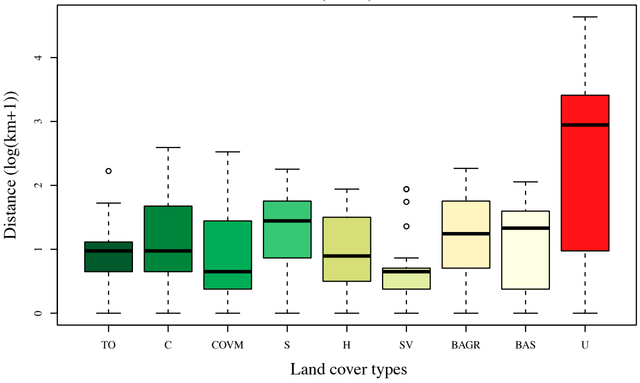

Trachylepis tessellata
(Anderson, 1895)

S78: Species information
Lizards
Skinks, Scincidae

A

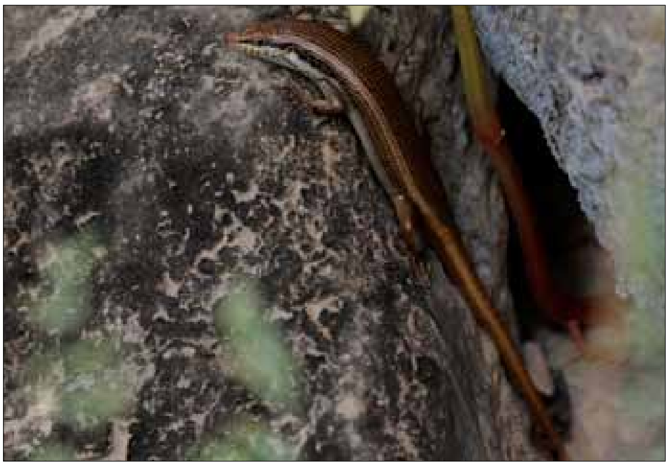

B

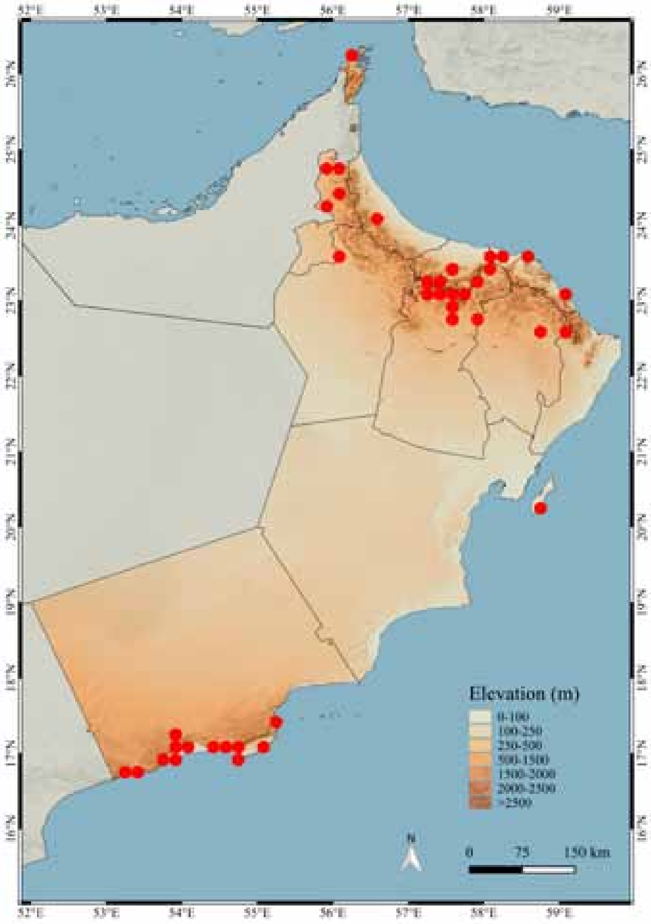

C

Useful information	
IUCN Category	LC
Endemic	NO
Venomous	NO
Insular	YES
Present in a protected area	YES

D

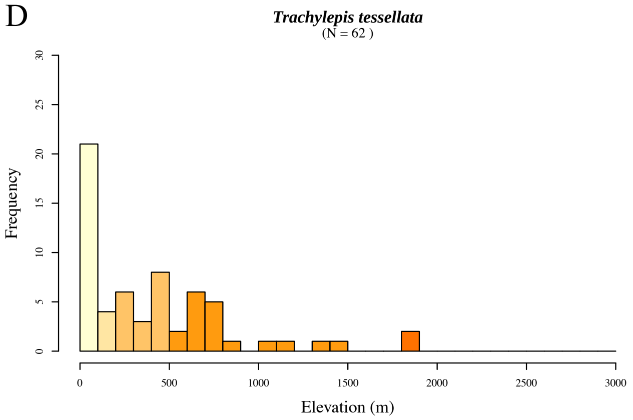

E

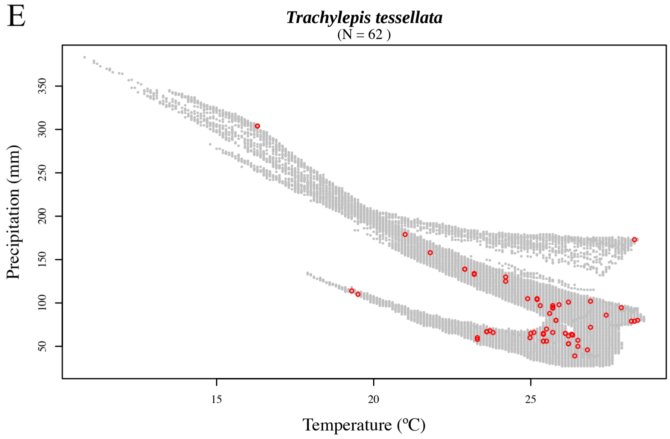

F

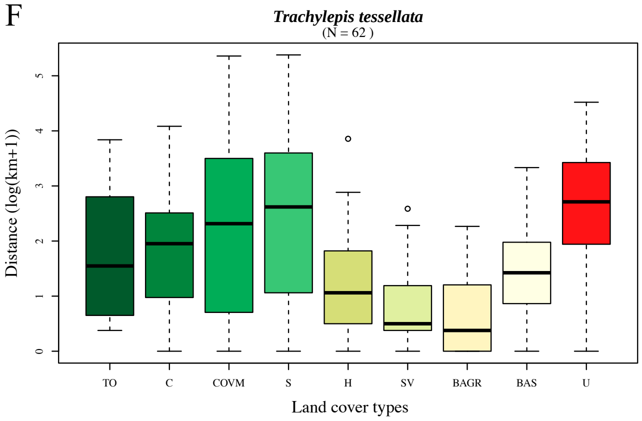

S79: Species information

Lizards

Amphisbaenids, Trogonophidae

Diplometopon zarudnyi **Nikolsky, 1907**

A

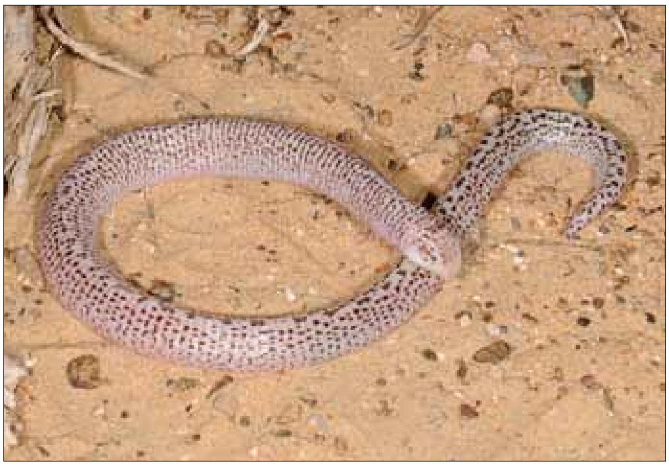

B

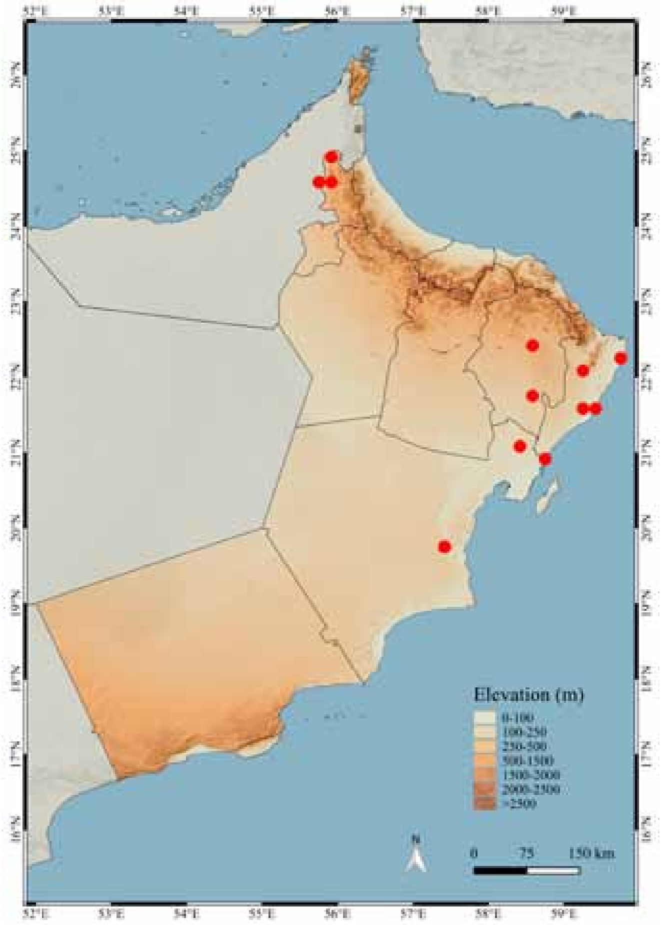

C

Useful information	
IUCN Category	LC
Endemic	NO
Venomous	NO
Insular	NO
Present in a protected area	YES

D

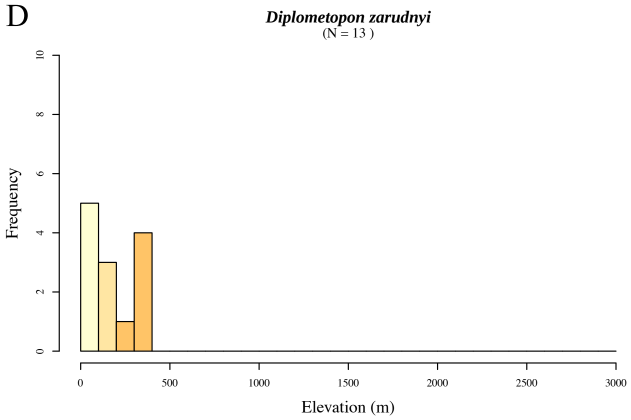

E

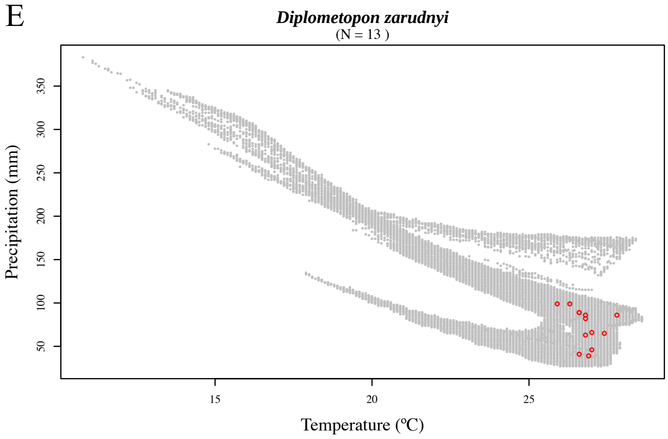

F

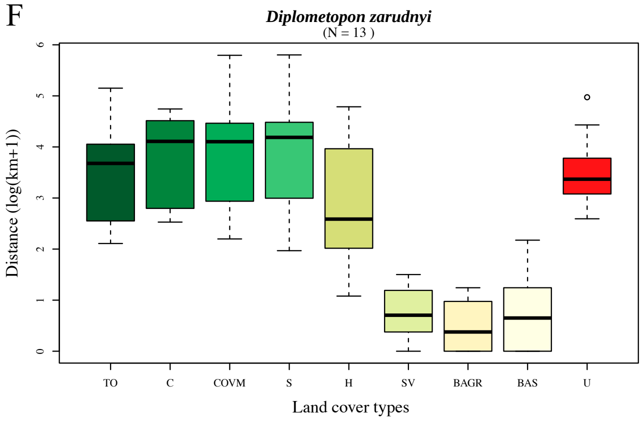

Varanus griseus
(Daudin, 1803)

S80: Species information
Lizards
Varanids, Varanidae

A

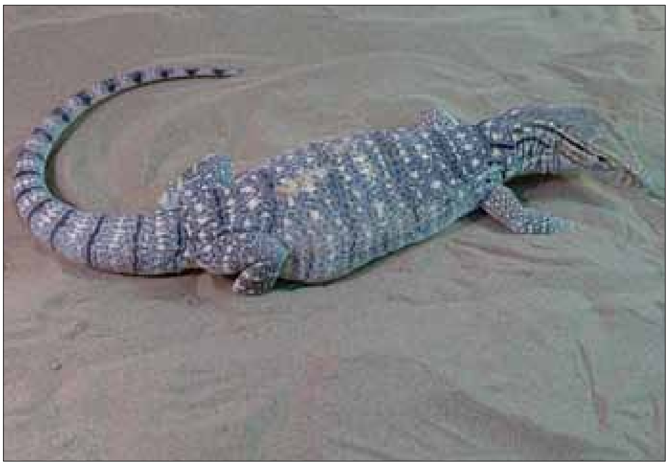

B

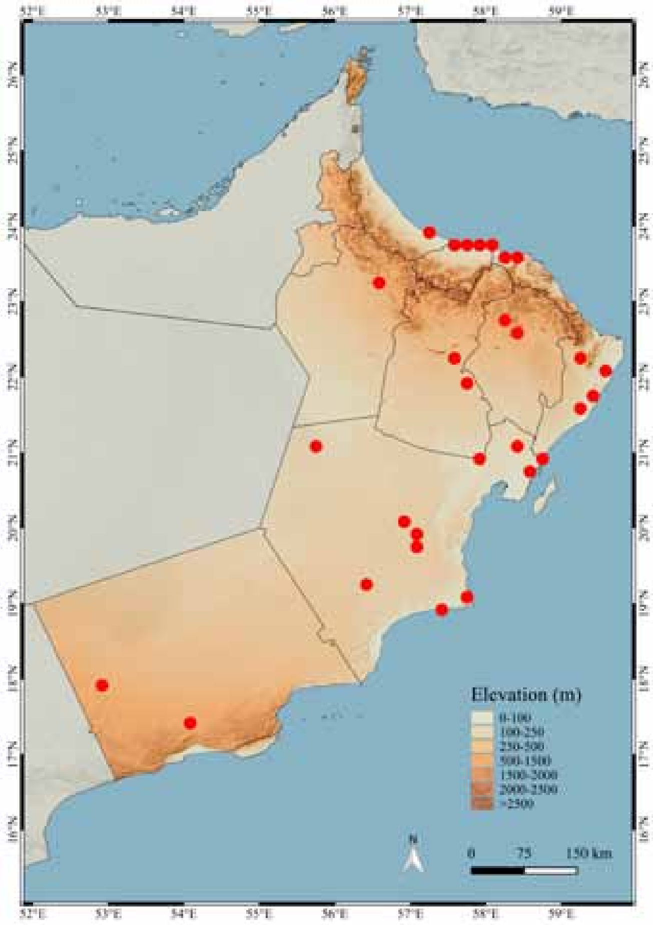

C

Useful information	
IUCN Category	LC*
Endemic	NO
Venomous	NO
Insular	NO
Present in a protected area	YES

*Not available on the web

D

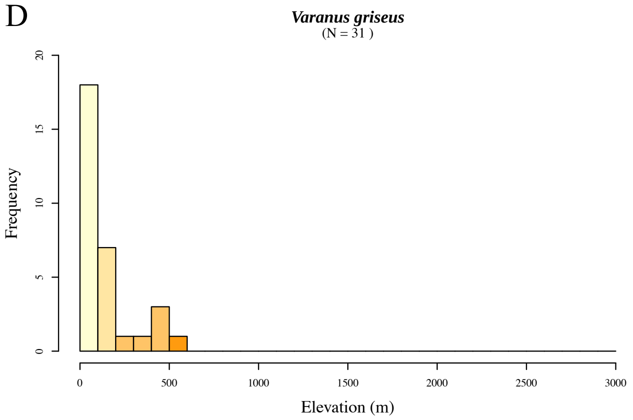

E

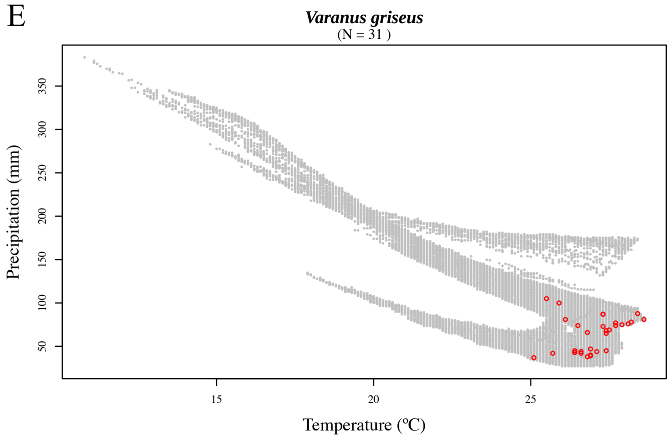

F

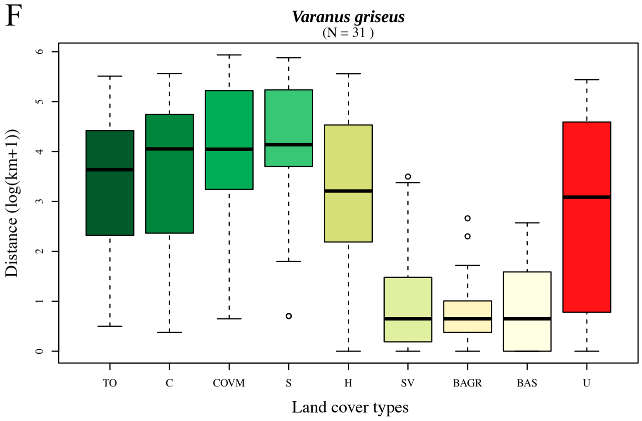

S81: Species information

Snakes

Boidae

Eryx jayakari
Boulenger, 1888

A

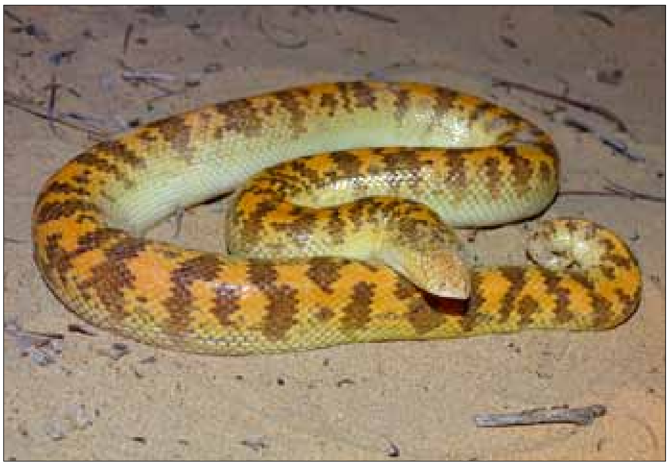

B

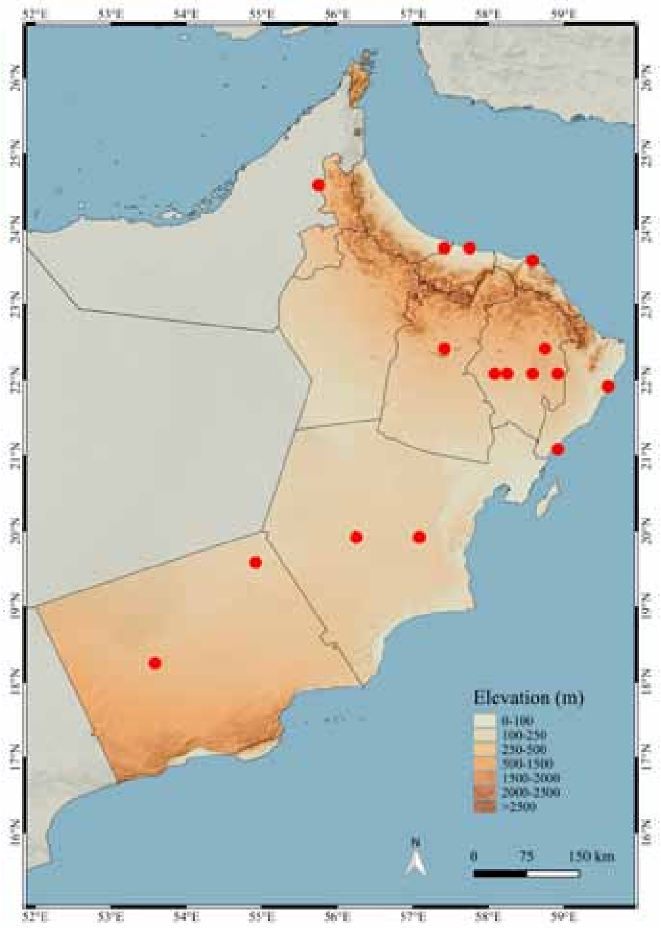

C

Useful information	
IUCN Category	LC
Endemic	NO
Venomous	NO
Insular	NO
Present in a protected area	YES

D

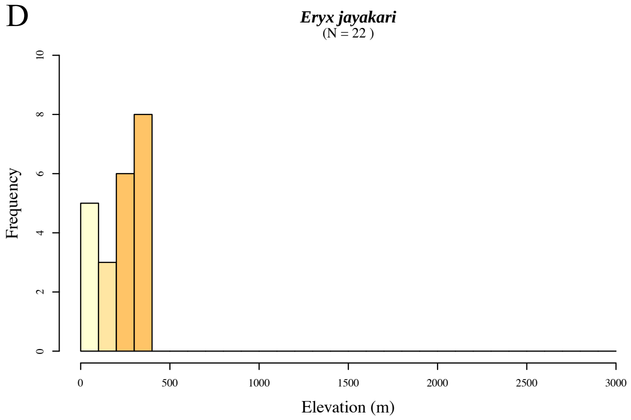

E

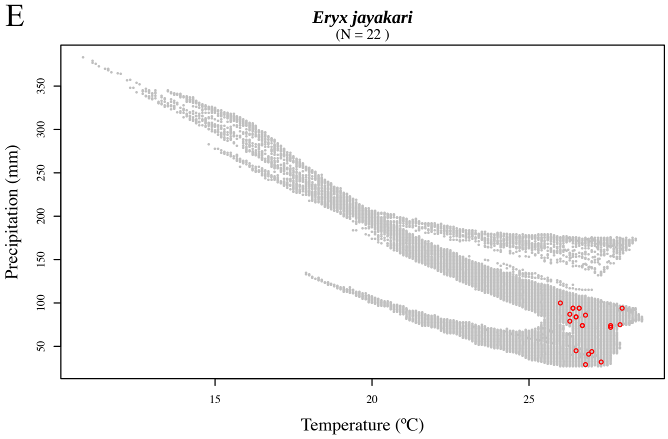

F

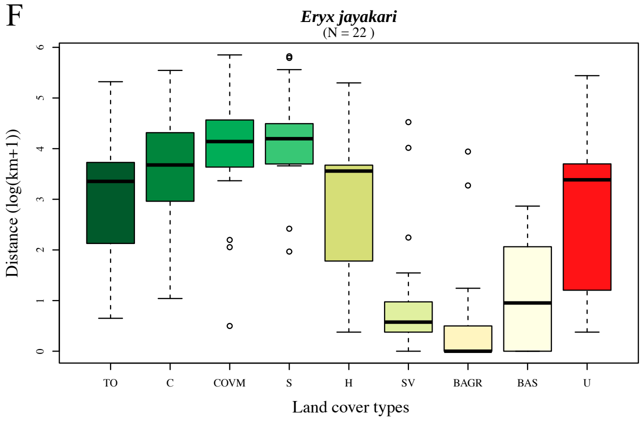

Lytorhynchus diadema diadema
(Duméril, Bibron & Duméril, 1854)

S82: Species information

Snakes

Colubridae

A

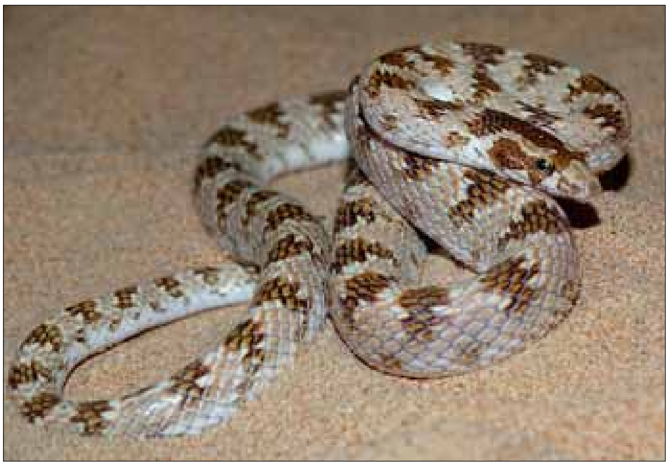

B

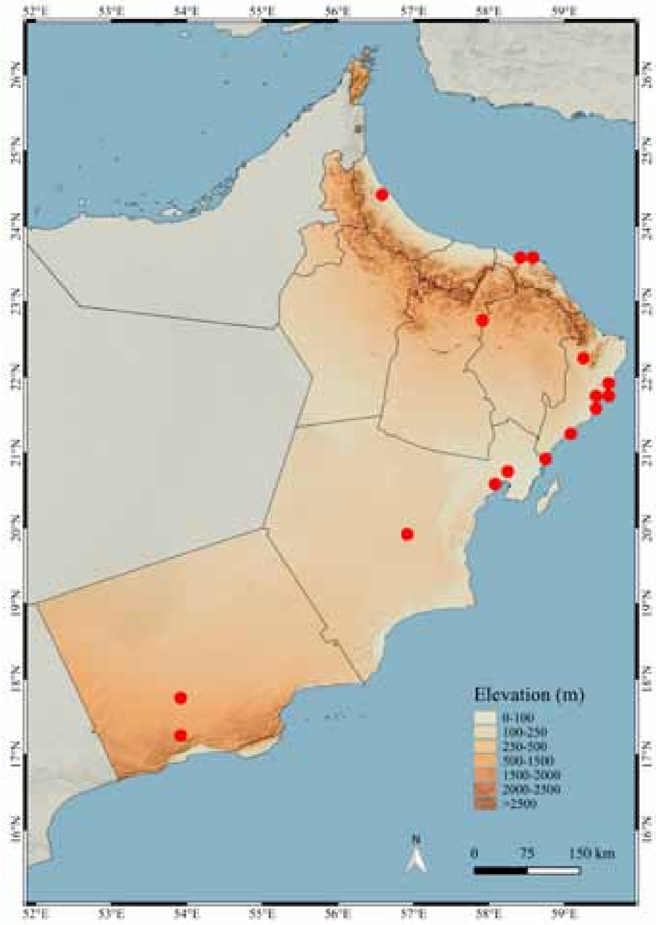

C

Useful information	
IUCN Category	LC
Endemic	NO
Venomous	NO
Insular	NO
Present in a protected area	YES

D

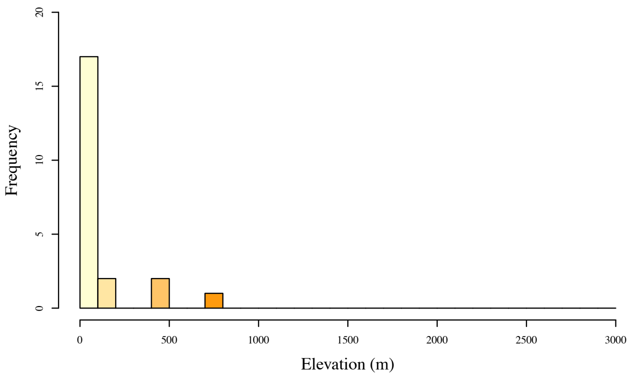

E

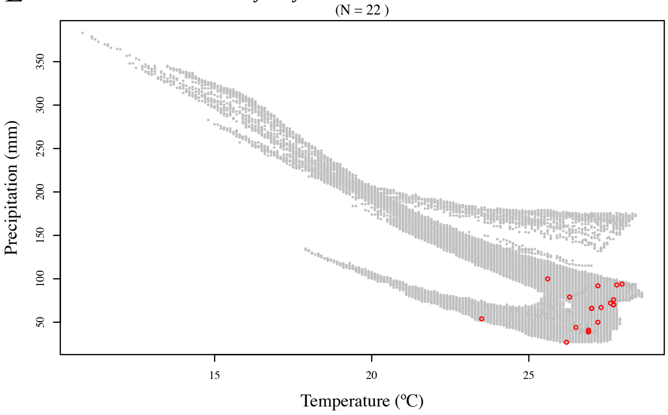

F

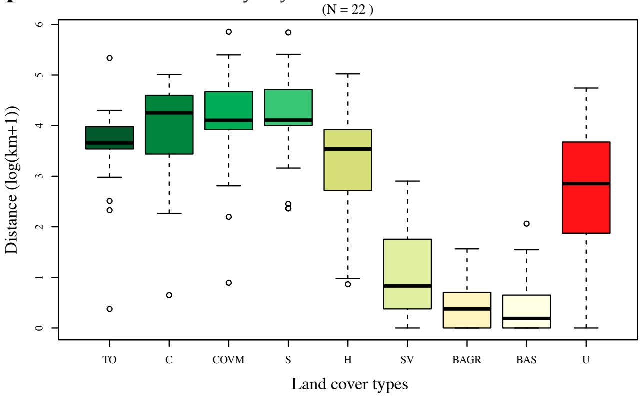

S83: Species information

Snakes

Colubridae

Platyceps rhodorachis rhodorachis

(Jan, 1865)

A

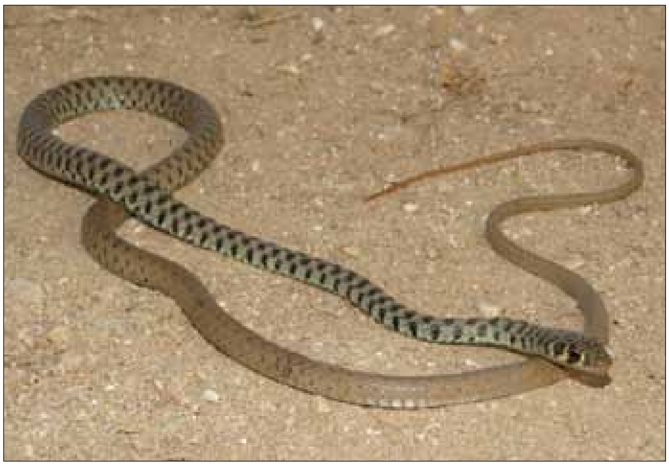

B

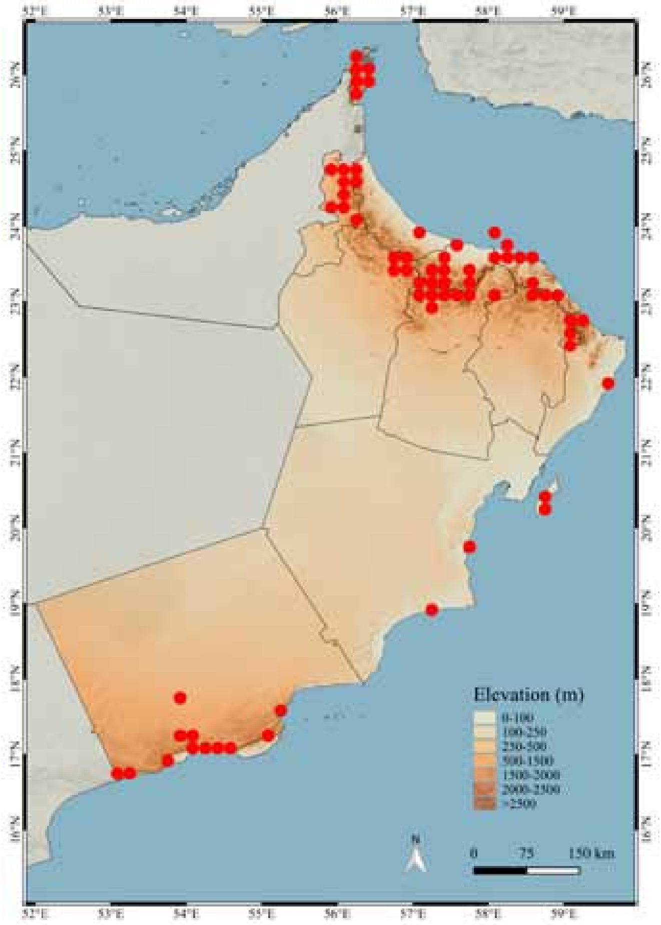

C

Useful information	
IUCN Category	LC*
Endemic	NO
Venomous	NO
Insular	YES
Present in a protected area	YES

*Not available on the web

D

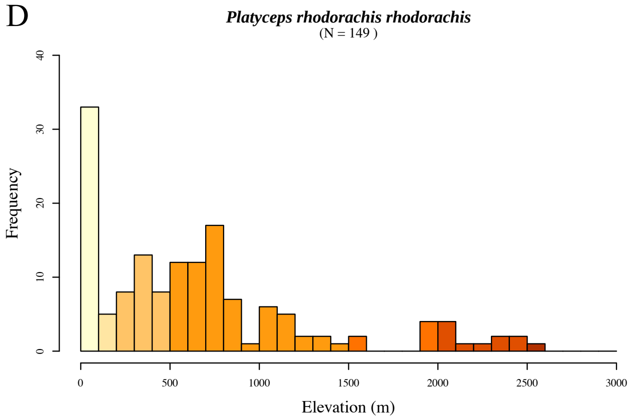

E

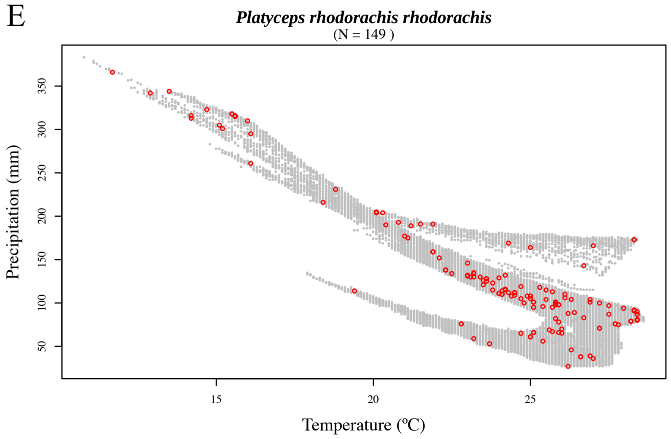

F

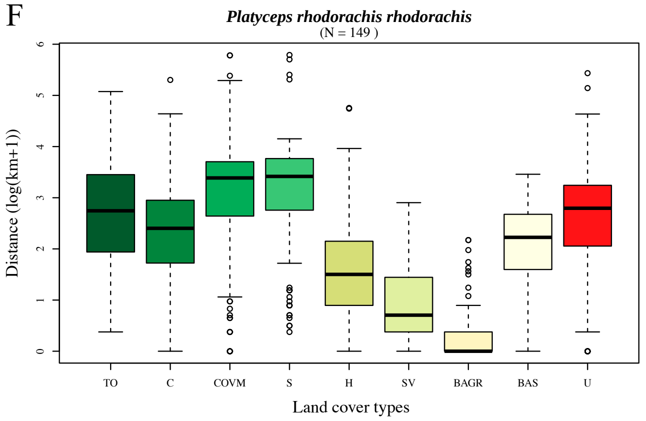

Platyceps thomasi
(Parker, 1931)

S84: Species information
Snakes
Colubridae

A

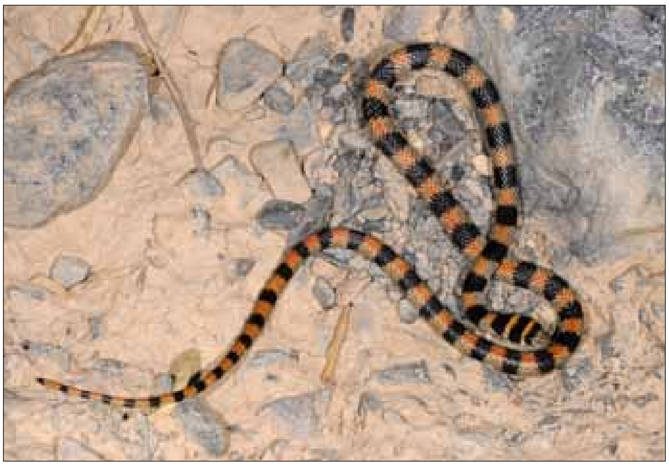

B

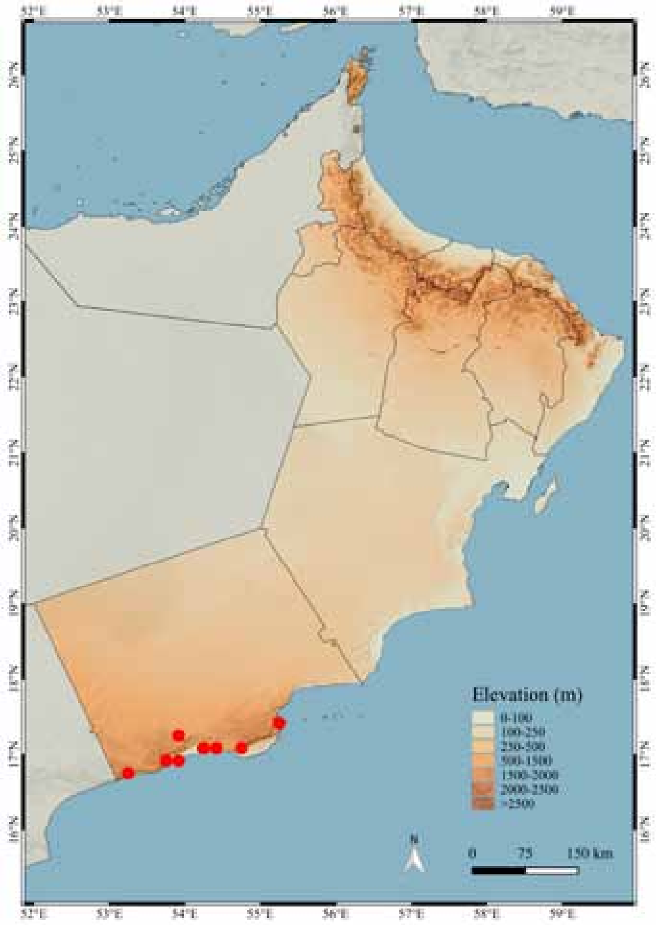

C

Useful information	
IUCN Category	DD
Endemic	NO
Venomous	NO
Insular	NO
Present in a protected area	YES

D

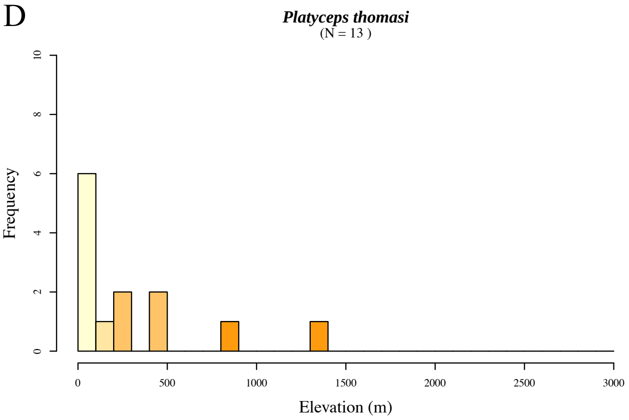

E

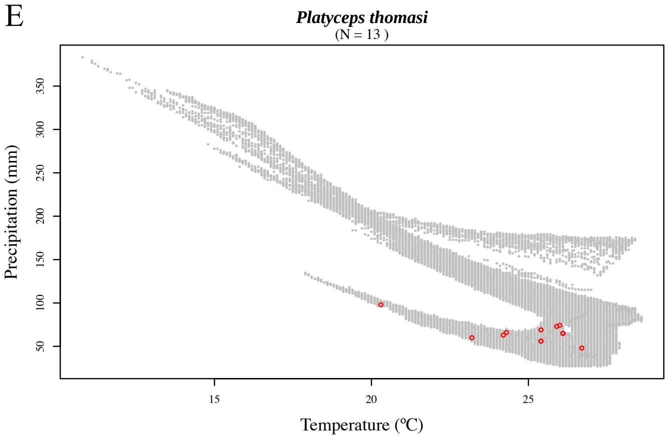

F

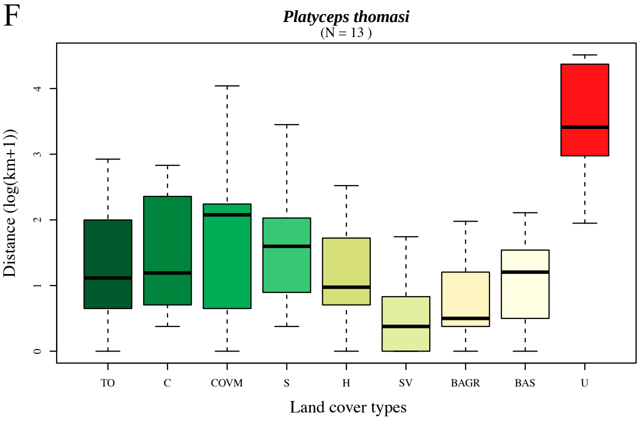

S85: Species information

Snakes

Colubridae

Rhynchocalamus arabicus

Schmidt, 1933

A

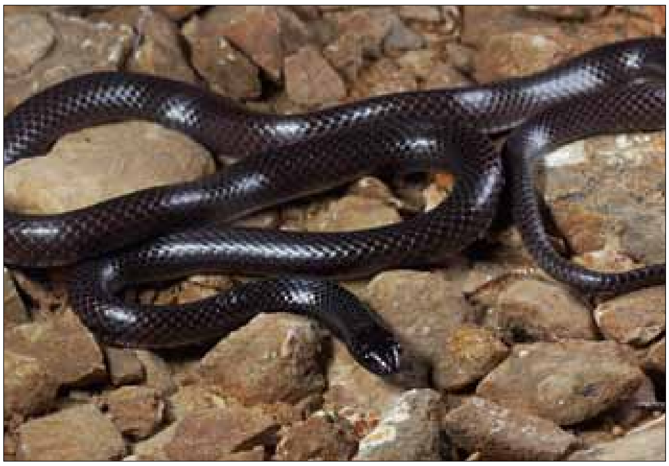

B

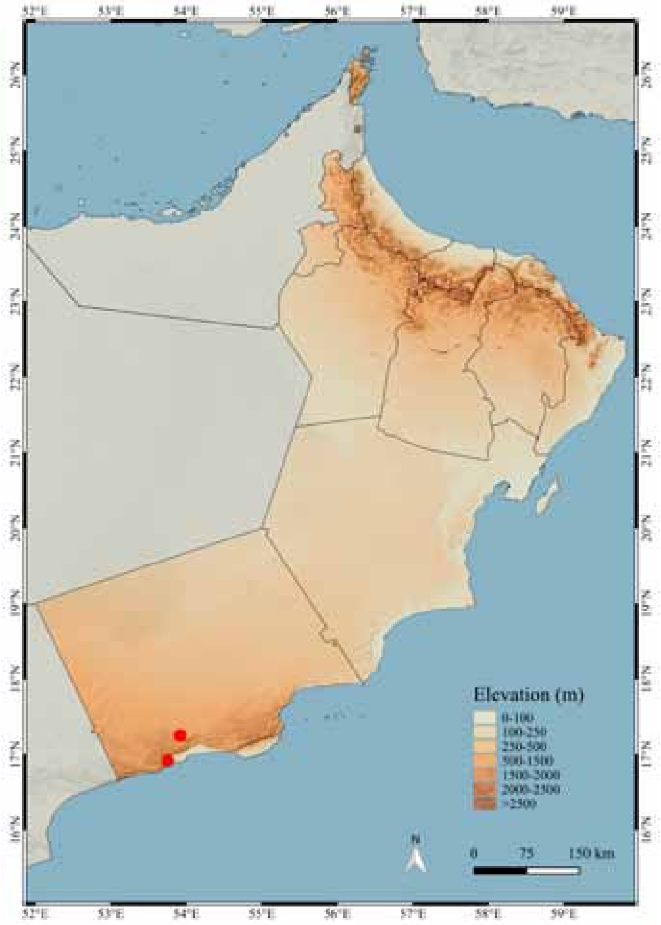

C

Useful information	
IUCN Category	DD
Endemic	NO
Venomous	NO
Insular	NO
Present in a protected area	NO

D

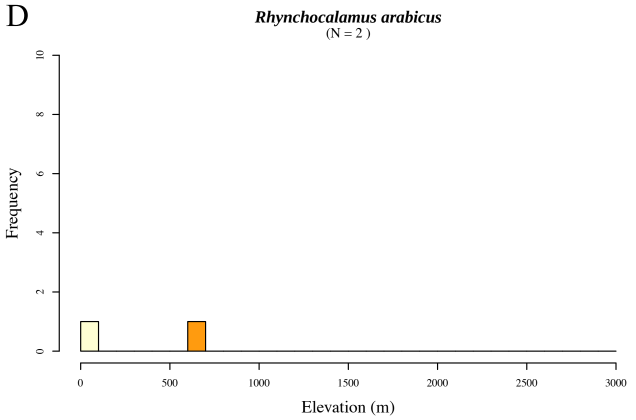

E

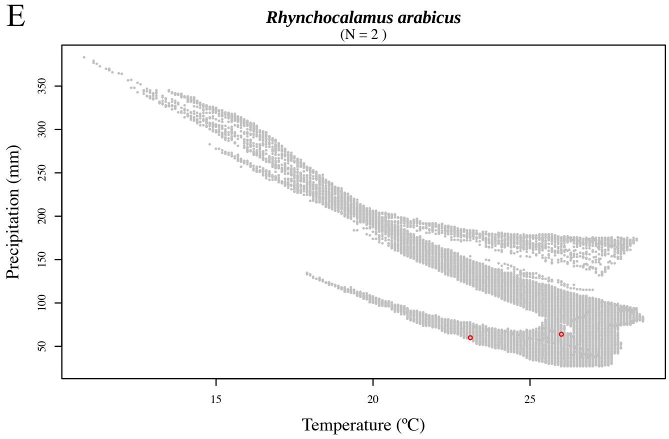

F

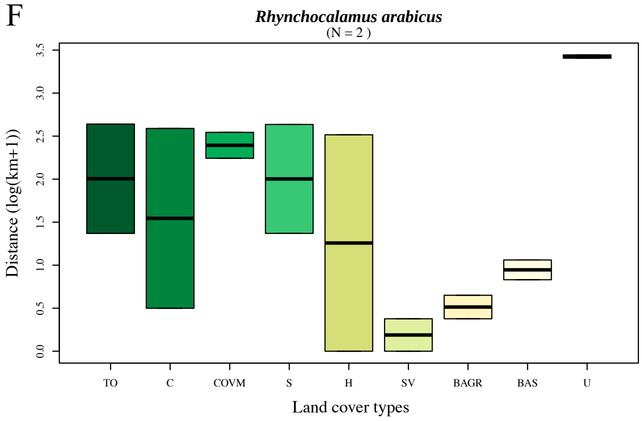

Spalerosophis diadema cliffordii
(Schlegel, 1837)

S86: Species information
Snakes
Colubridae

A

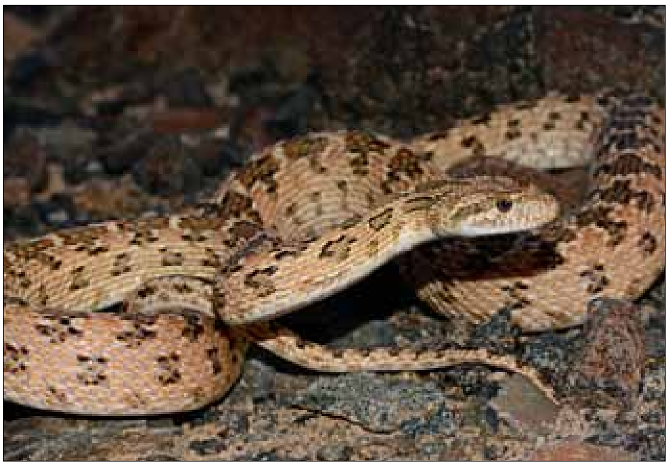

B

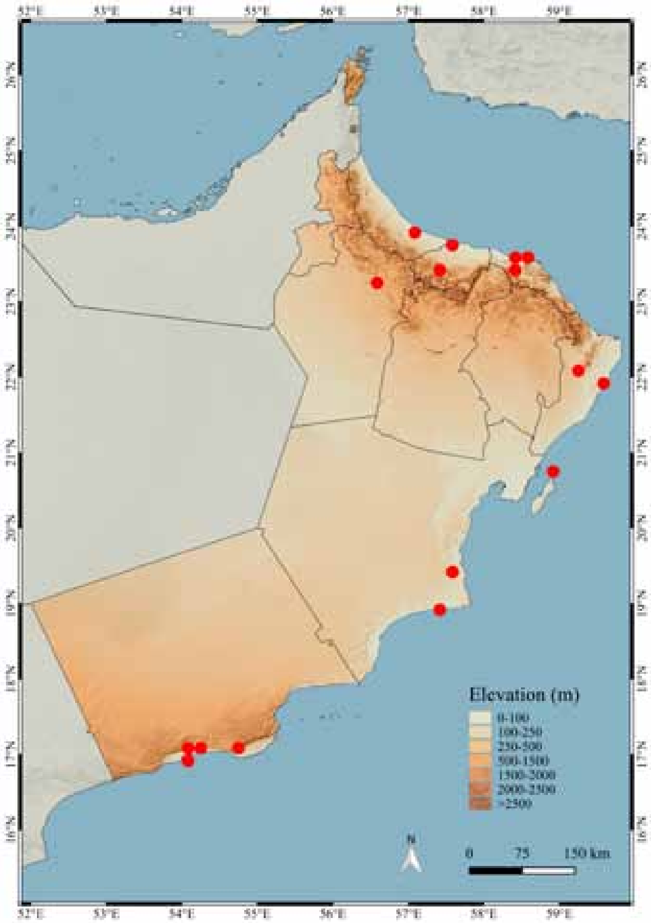

C

Useful information	
IUCN Category	LC*
Endemic	NO
Venomous	NO
Insular	YES
Present in a protected area	YES

*Not available on the web

D

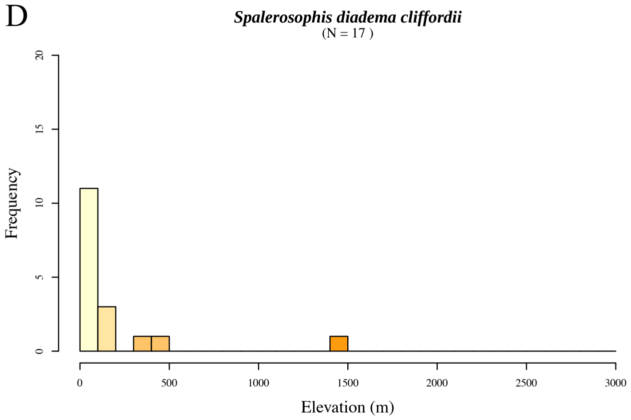

E

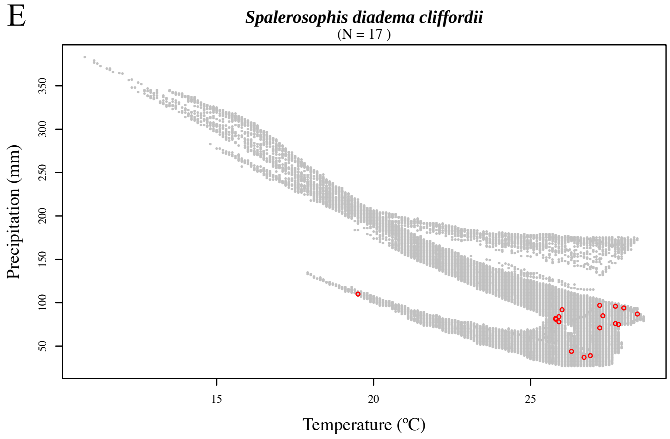

F

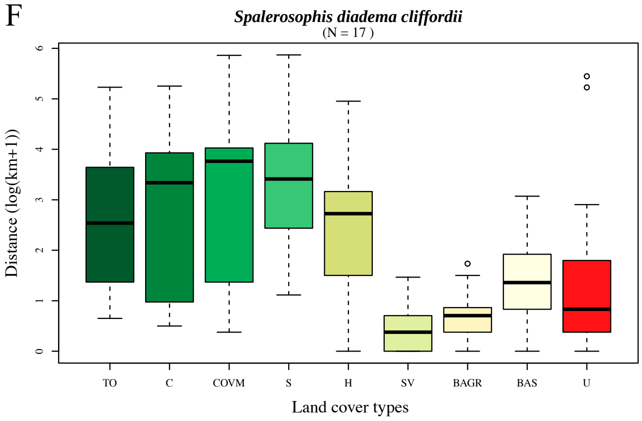

S87: Species information

Snakes

Colubridae

Telescopus dhara dhara
(Forskal, 1775)

A

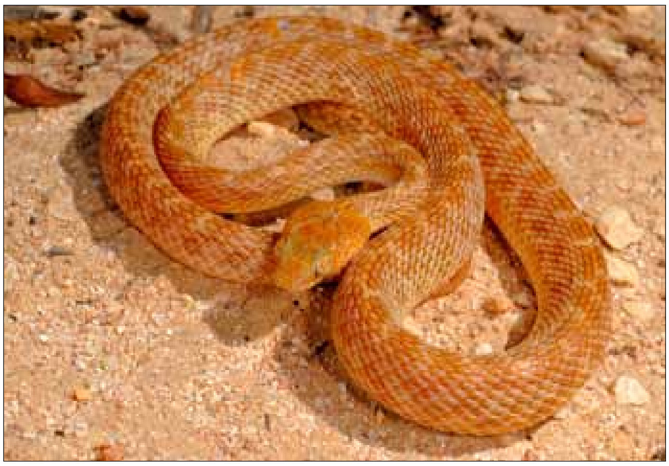

B

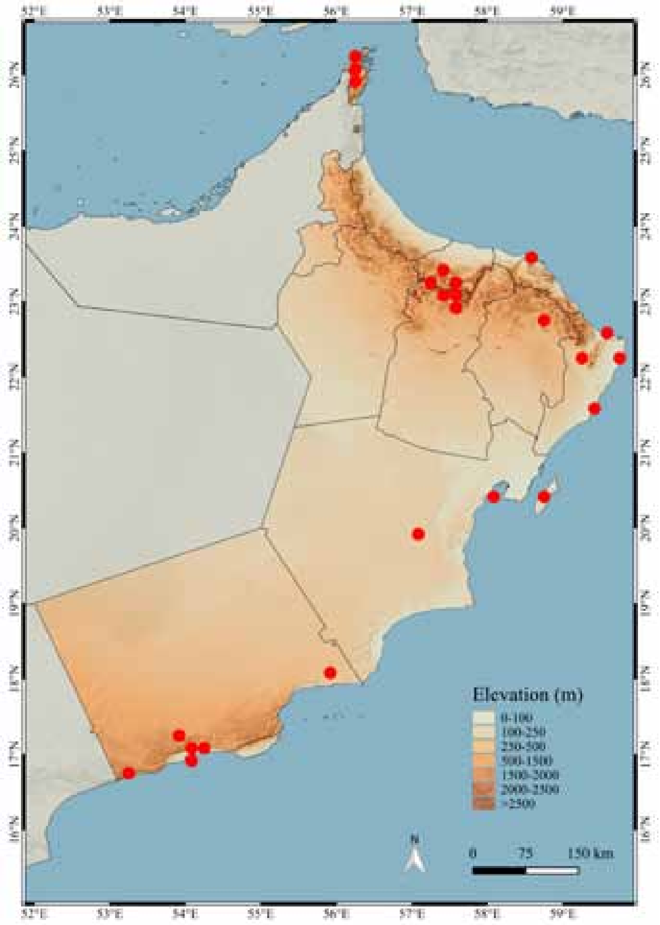

C

Useful information	
IUCN Category	LC*
Endemic	NO
Venomous	NO
Insular	YES
Present in a protected area	YES

*Not available on the web

D

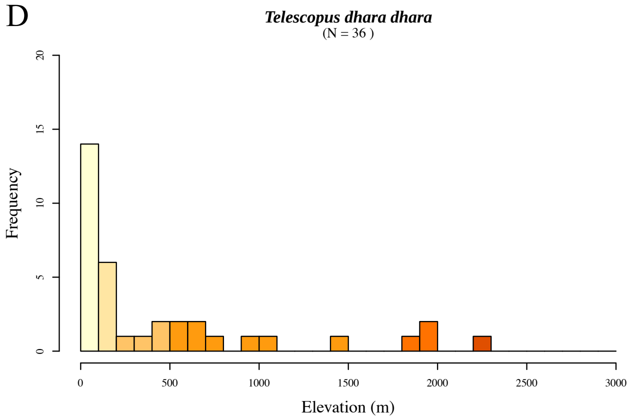

E

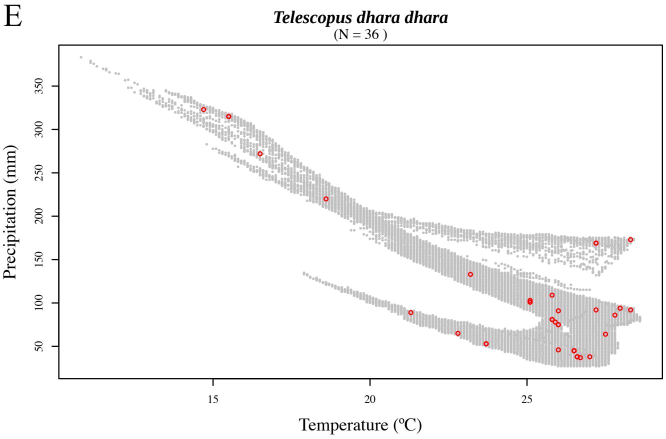

F

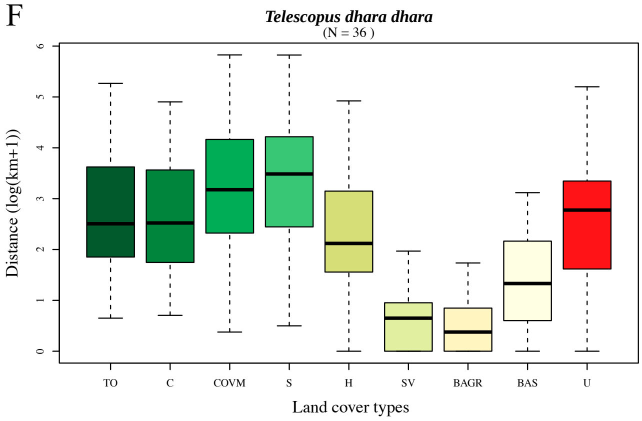

Lizards
Agamids
Geckos
Lacertids
Skinks
Amphisbaenids
Varanids
Snakes
Boidae
Colubridae
Elapidae
Lamprophiidae
Leptotyphlopidae
Typhlopidae
Viperidae

Naja arabica
Scortecci, 1932

S88: Species information
Snakes
Elapidae

A

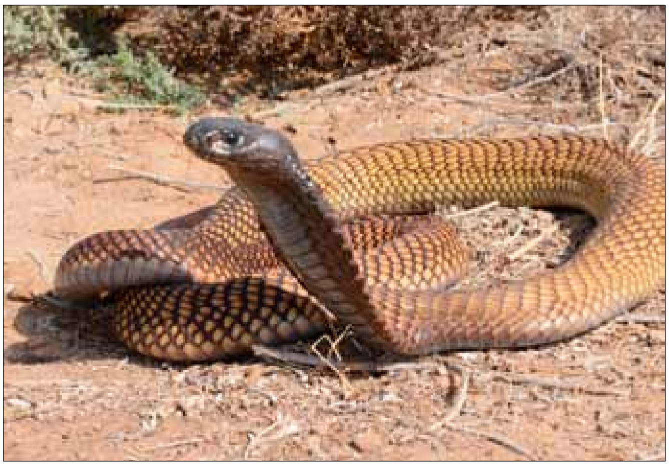

B

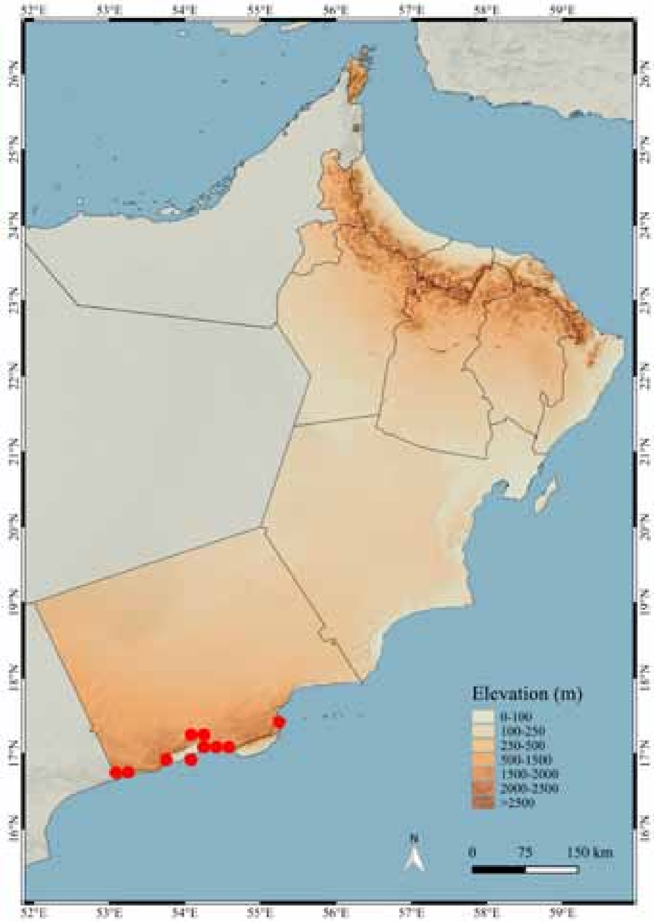

C

Useful information	
IUCN Category	LC
Endemic	NO
Venomous	YES
Insular	NO
Present in a protected area	YES

D

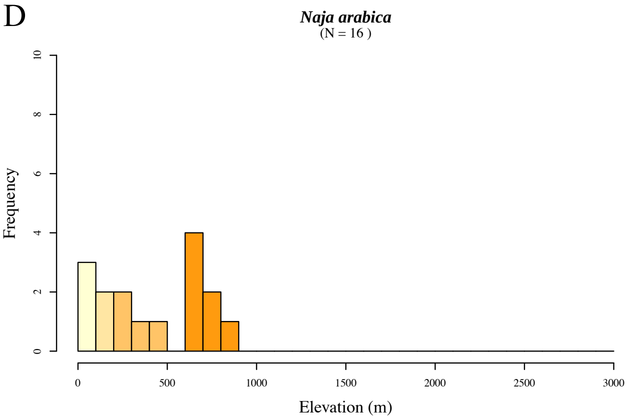

E

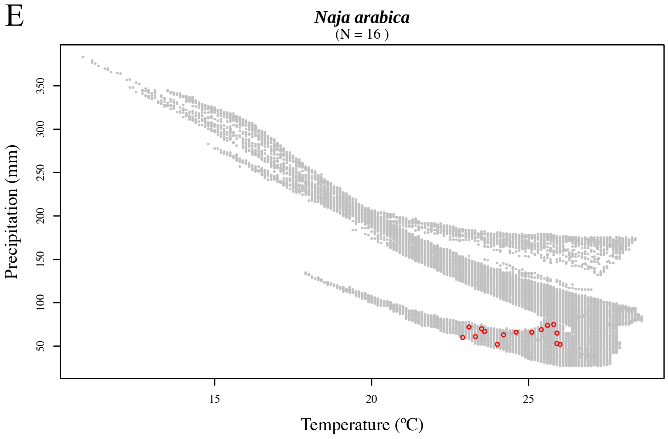

F

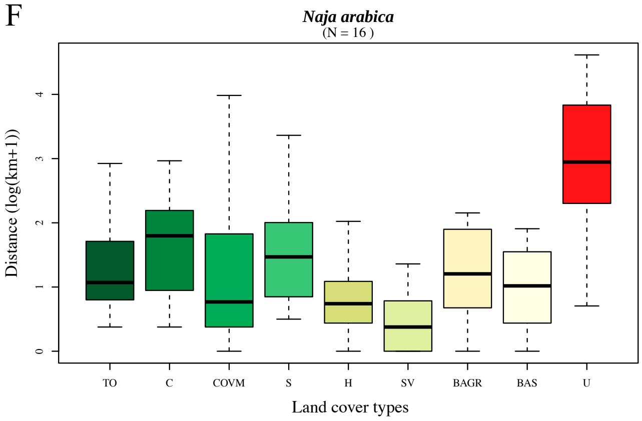

S89: Species information

Snakes

Lamprophiidae

Atractaspis andersonii
Boulenger, 1905

A

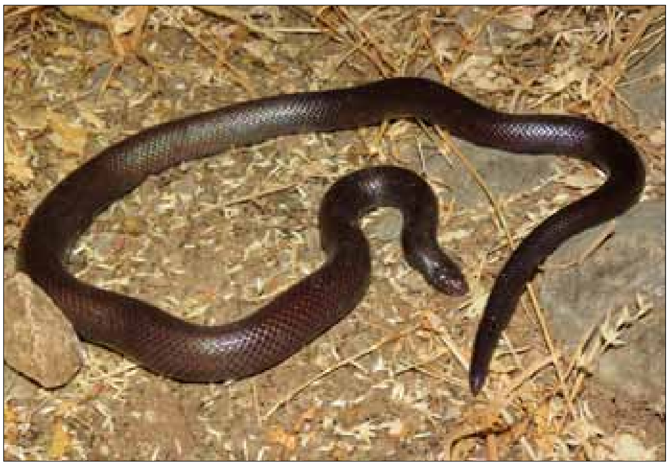

B

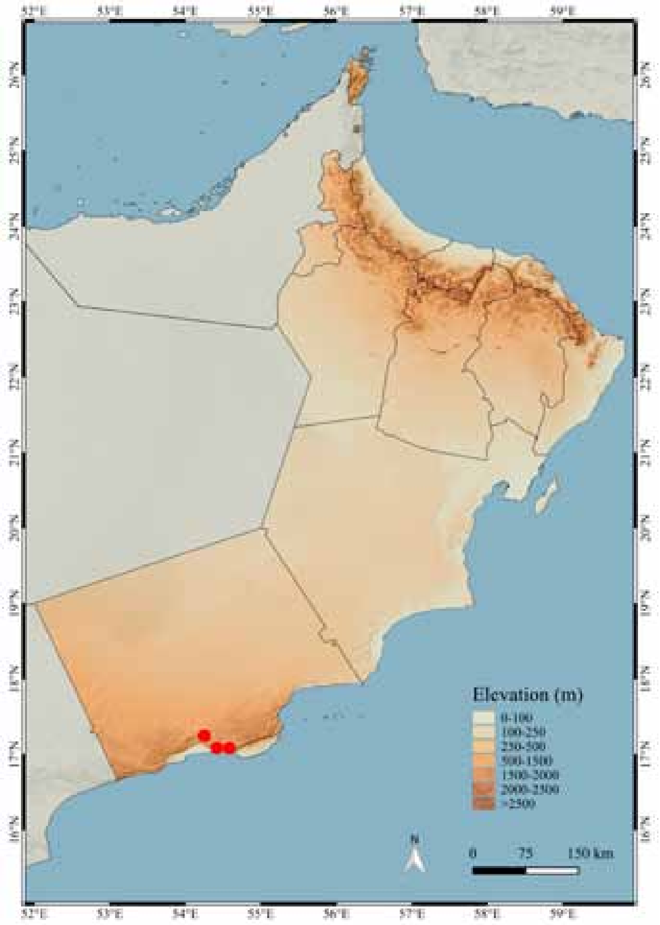

C

Useful information	
IUCN Category	LC
Endemic	NO
Venomous	YES
Insular	NO
Present in a protected area	NO

D

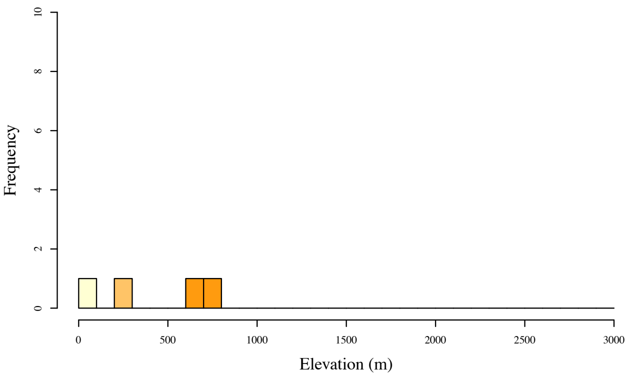

E

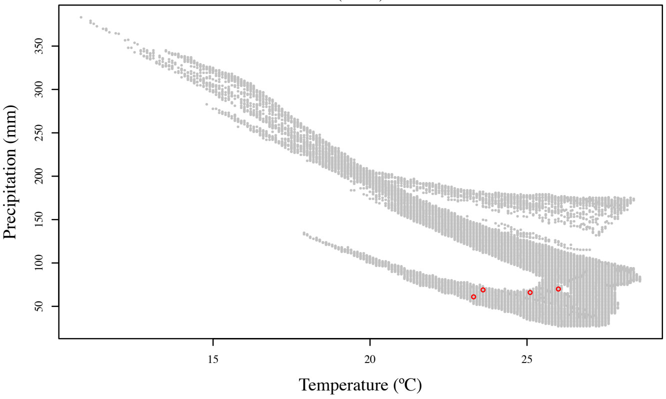

F

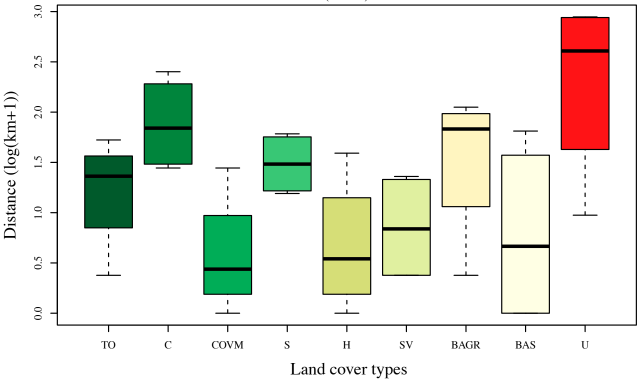

Psammophis schokari
(Forskal, 1775)

S90: Species information
Snakes
Lamprophiidae

A

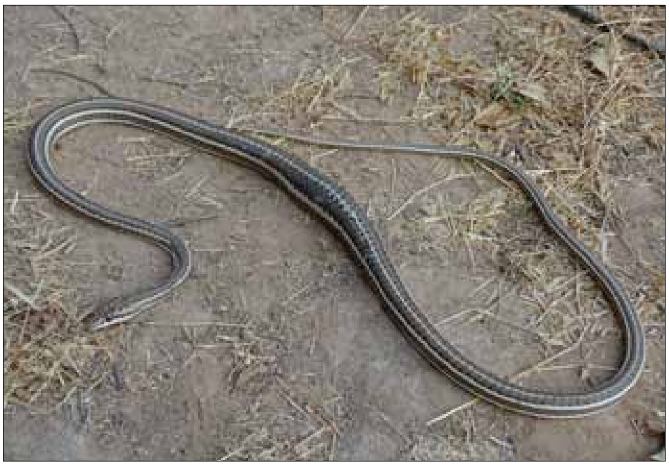

B

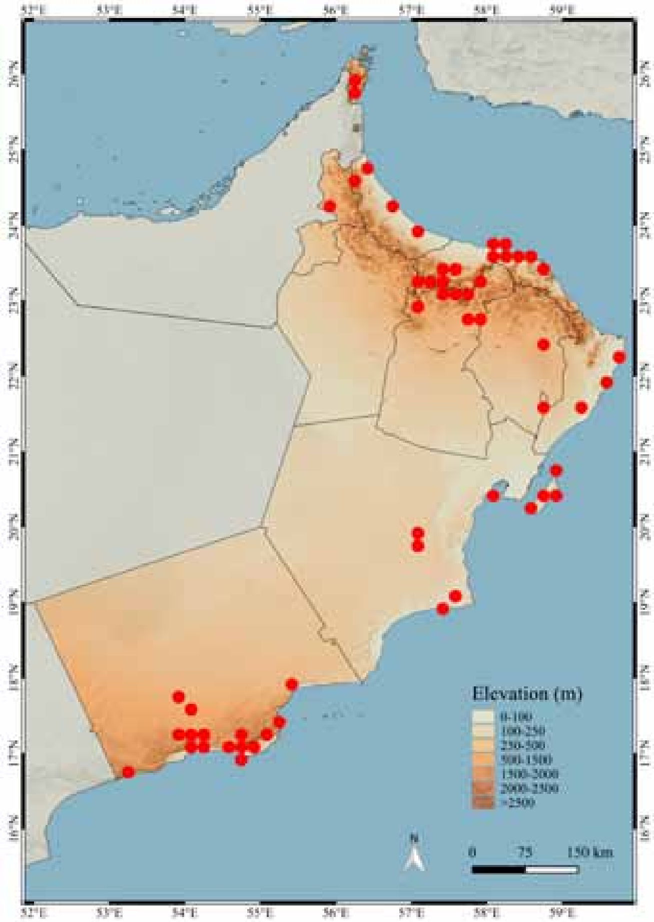

C

Useful information	
IUCN Category	LC*
Endemic	NO
Venomous	NO
Insular	YES
Present in a protected area	YES

*Not available on the web

D

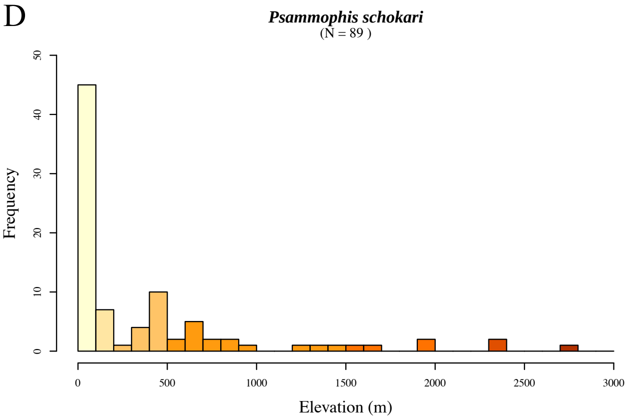

E

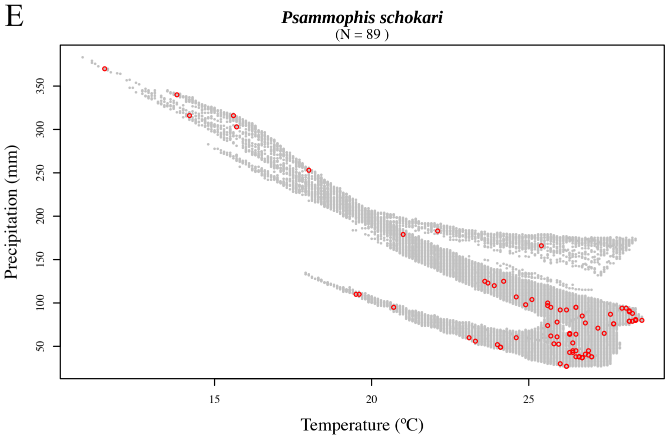

F

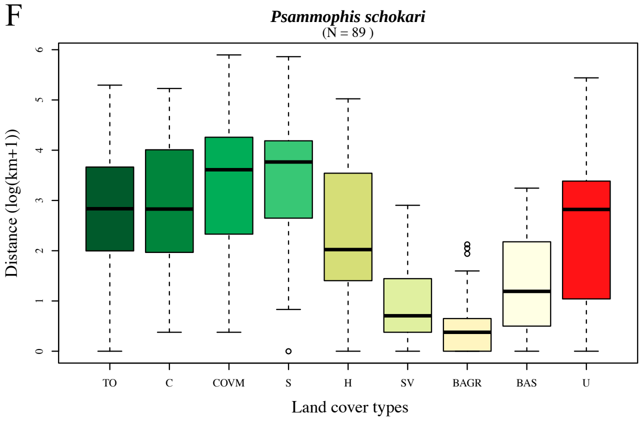

S91: Species information

Snakes

Lamprophiidae

Rhagerhis moilensis
(Reuss, 1834)

A

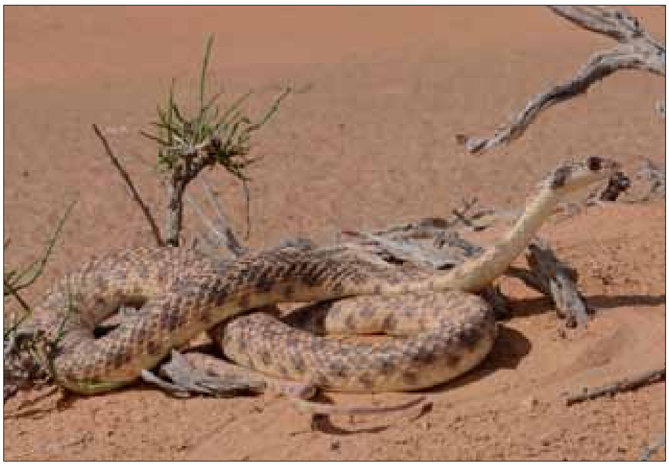

B

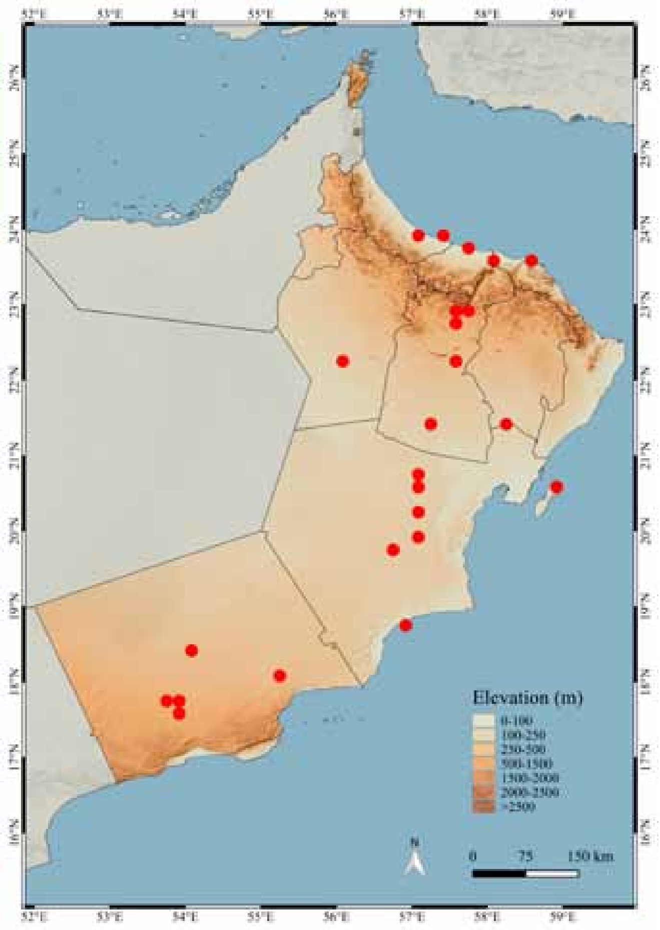

C

Useful information

Useful information	
IUCN Category	LC*
Endemic	NO
Venomous	NO
Insular	YES
Present in a protected area	YES

*Not available on the web

D

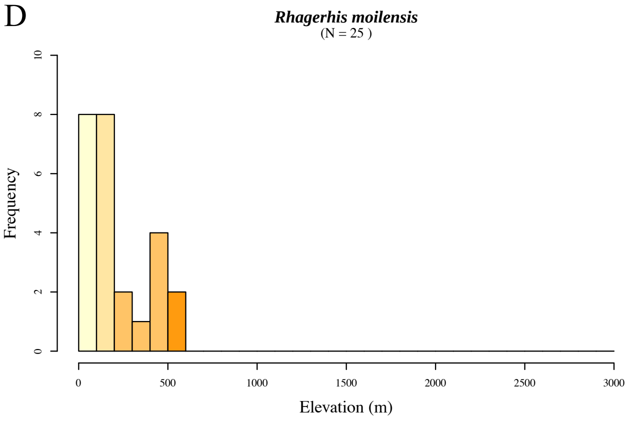

E

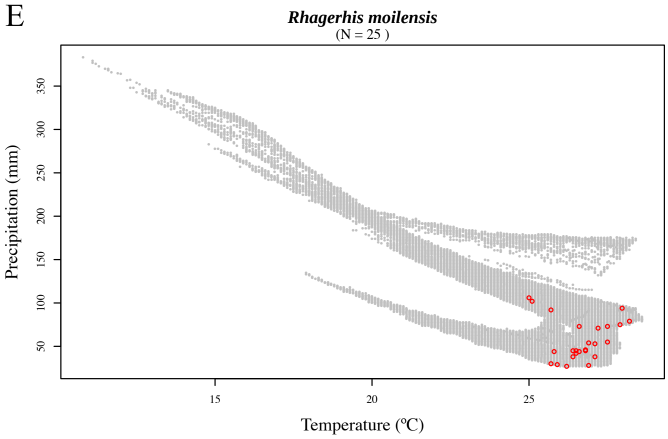

F

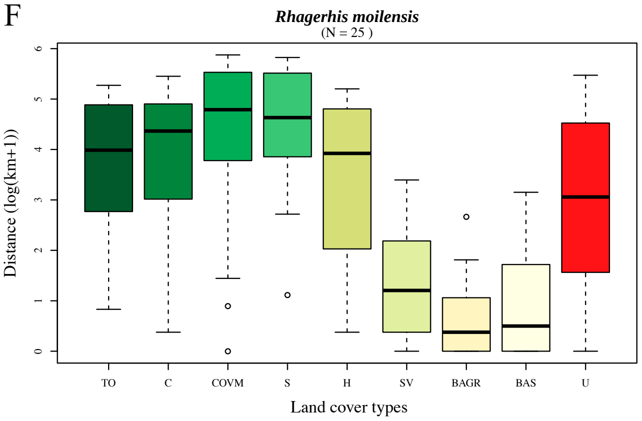

Myriopholis macrorhyncha
(Jan, 1860)

S92: Species information
Snakes
Leptotyphlopidae

A

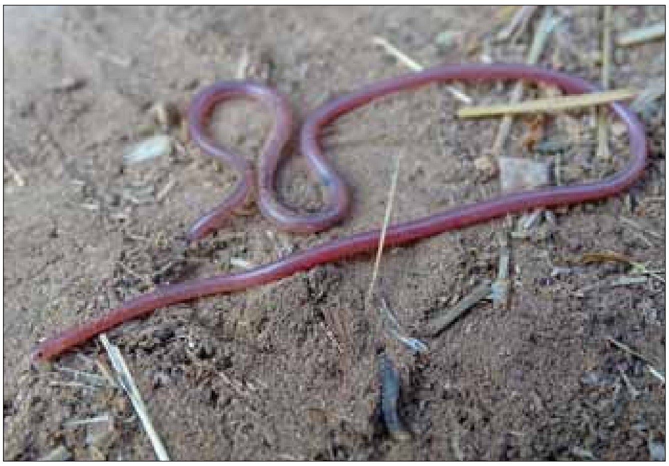

B

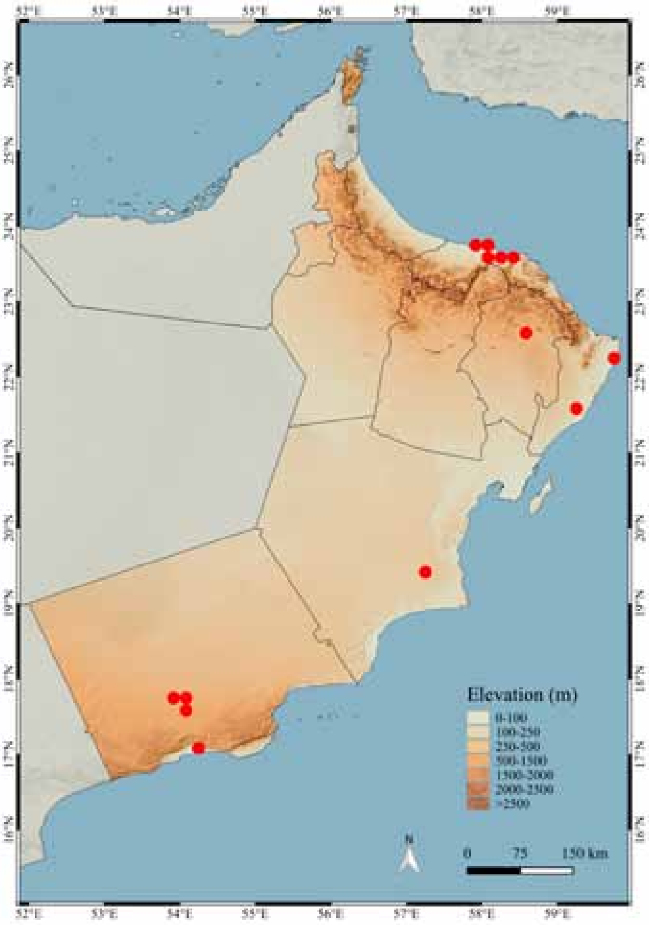

C

Useful information	
IUCN Category	LC*
Endemic	NO
Venomous	NO
Insular	NO
Present in a protected area	YES

*Not available on the web

D

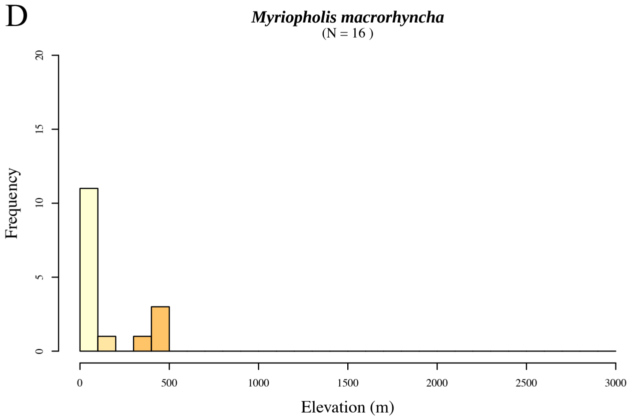

E

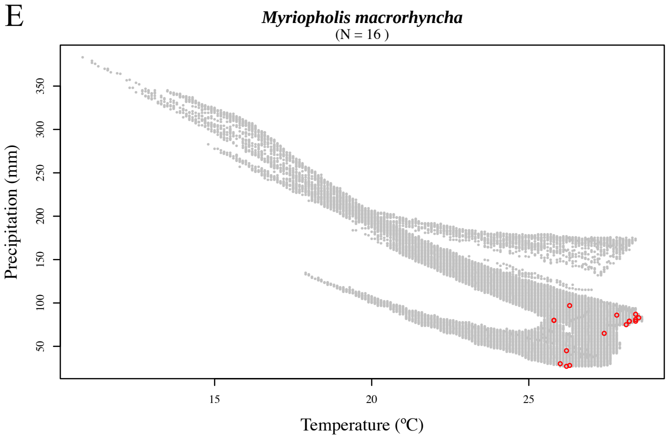

F

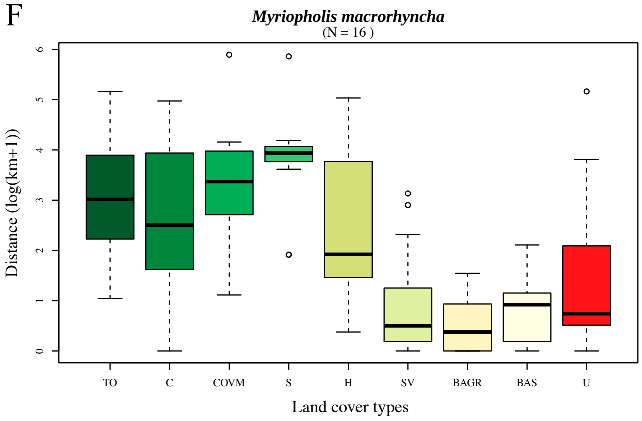

S93: Species information

Snakes

Leptotyphlopidae

Myriopholis nursii (Anderson, 1896)

A

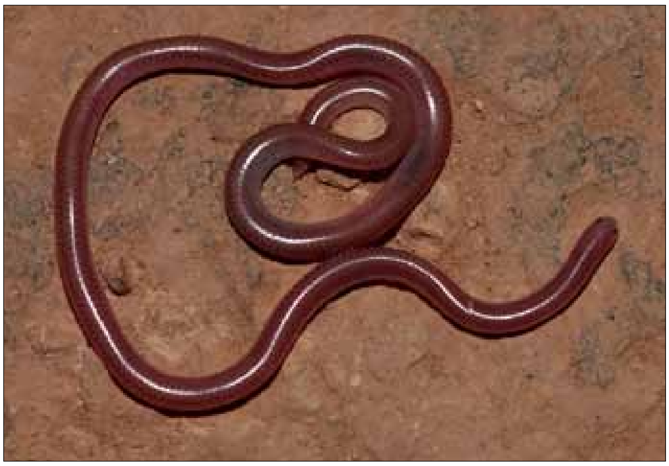

B

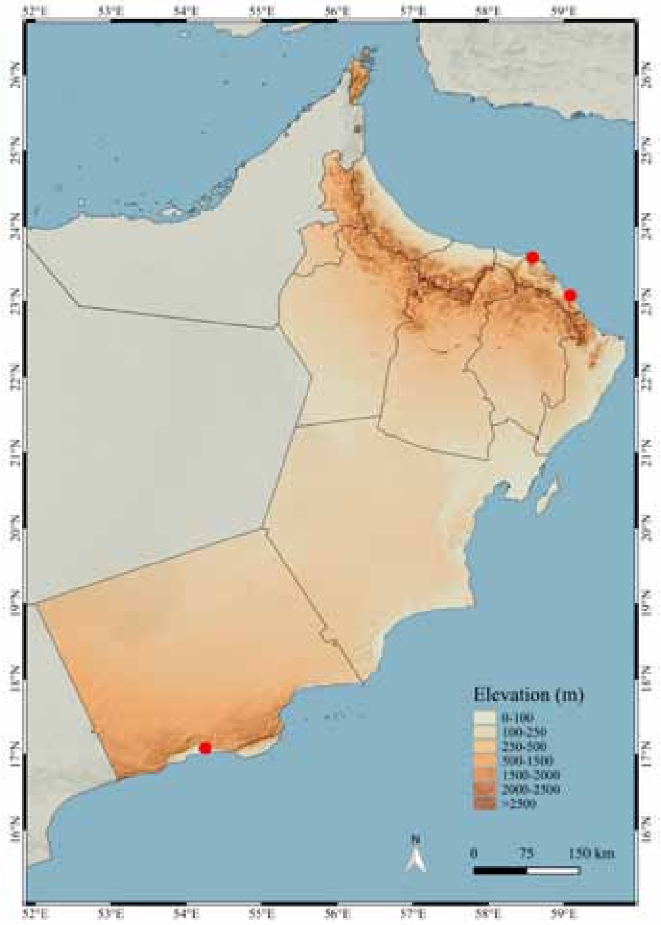

C

Useful information

IUCN Category	LC*
Endemic	NO
Venomous	NO
Insular	NO
Present in a protected area	YES

*Not available on the web

D

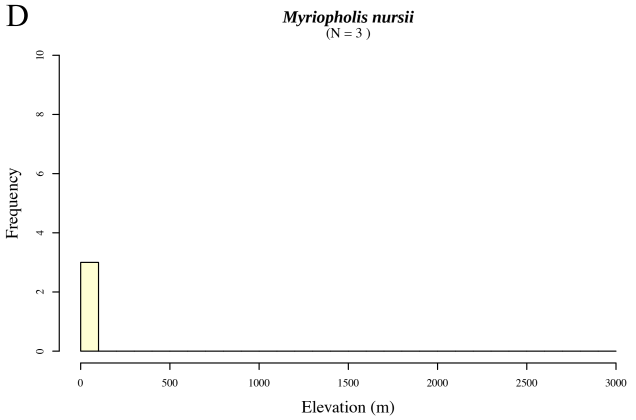

E

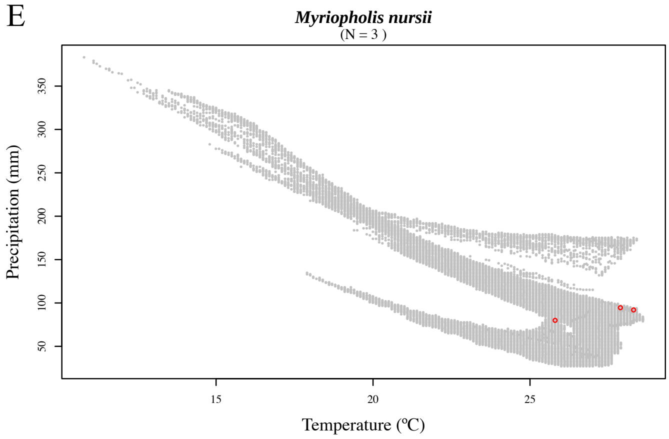

F

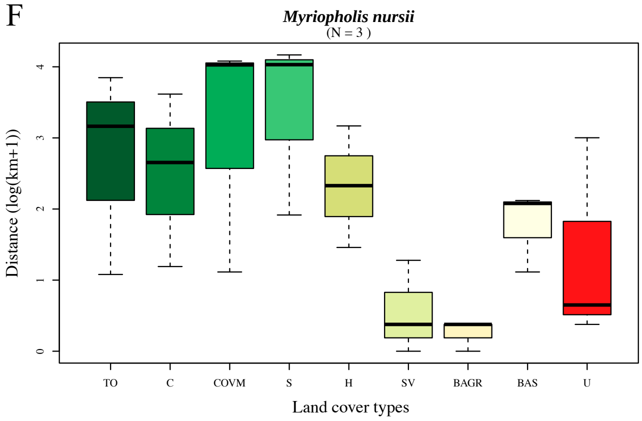

Indotyphlops braminus
(Daudin, 1803)

S94: Species information
Snakes
Typhlopidae

A

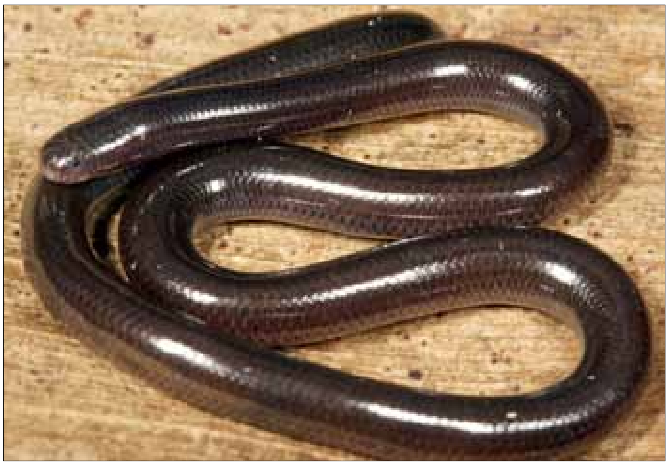

B

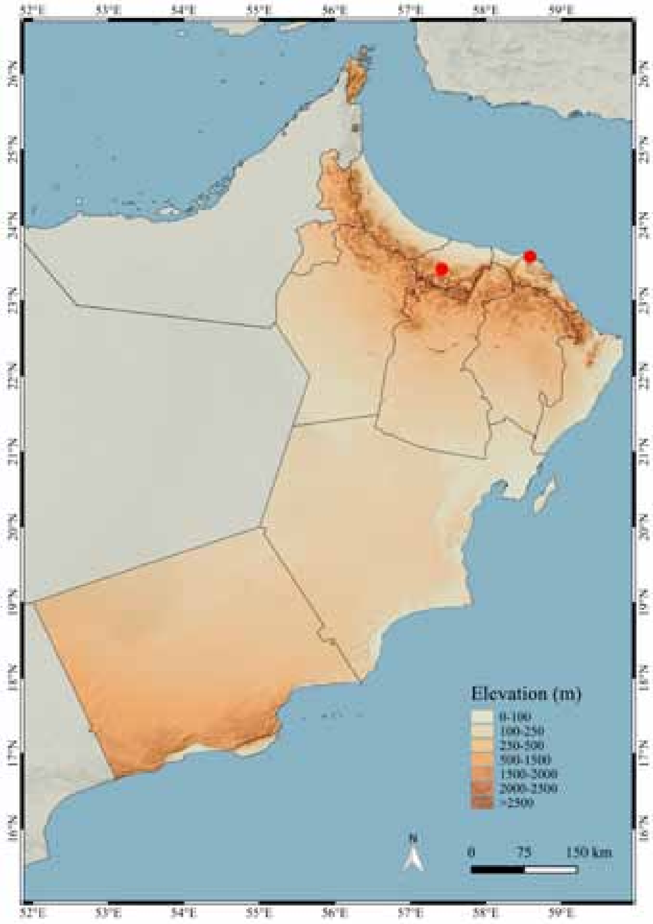

C

Useful information	
IUCN Category	LC*
Endemic	NO
Venomous	NO
Insular	NO
Present in a protected area	NO

*Not available on the web

D

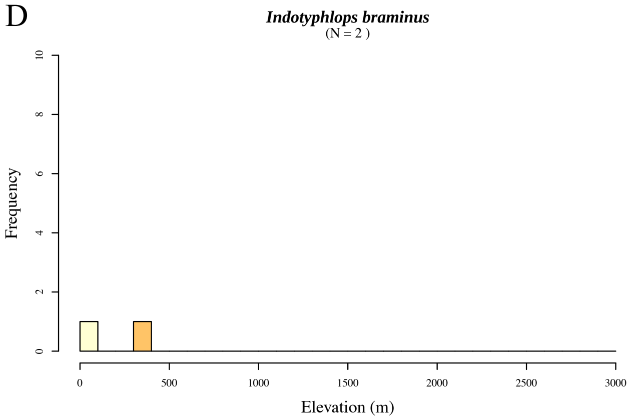

E

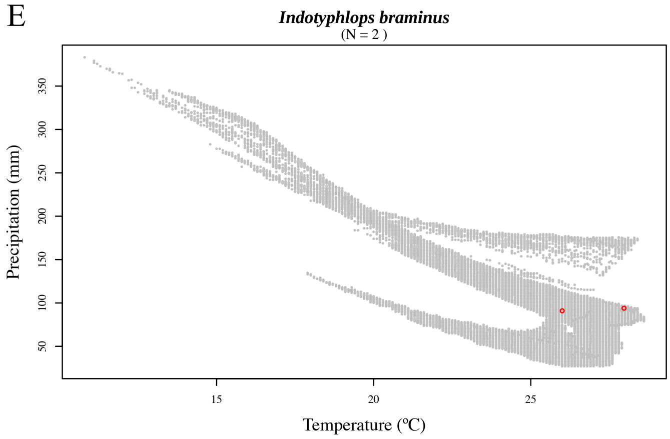

F

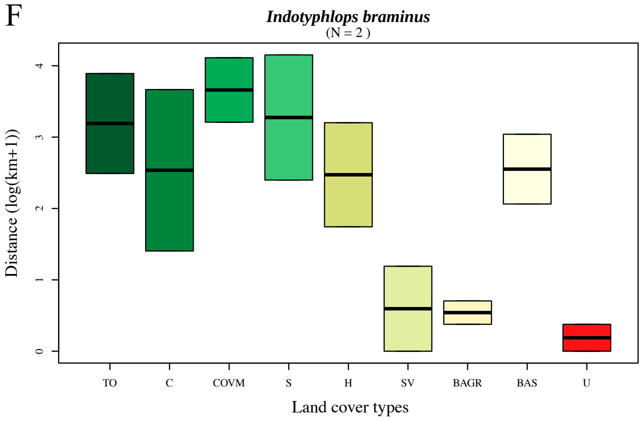

S95: Species information

Snakes

Viperidae

Bitis arietans

Merrem, 1820

A

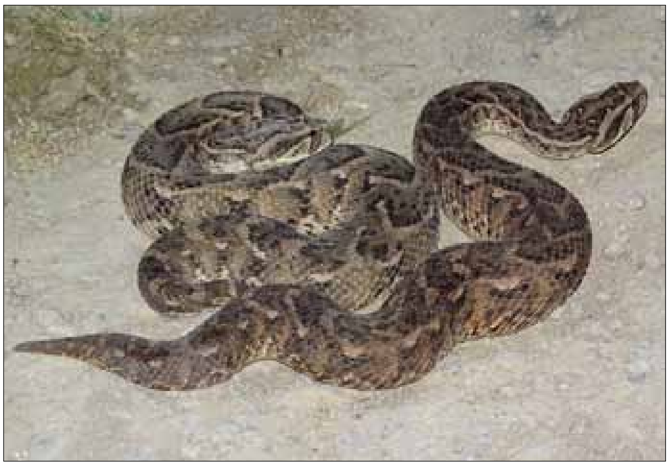

B

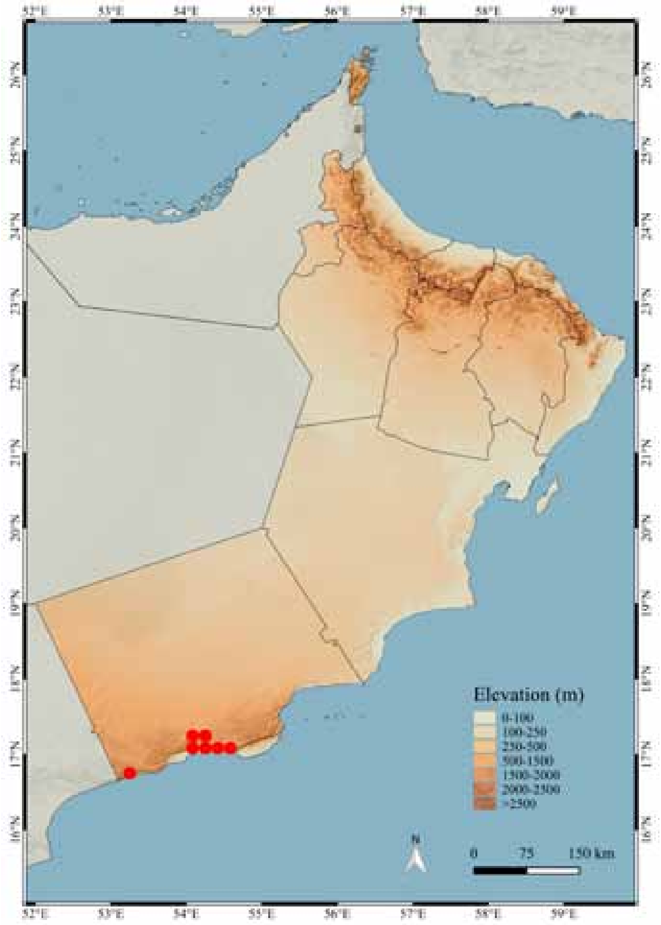

C

Useful information	
IUCN Category	LC*
Endemic	NO
Venomous	YES
Insular	NO
Present in a protected area	NO

*Not available on the web

D

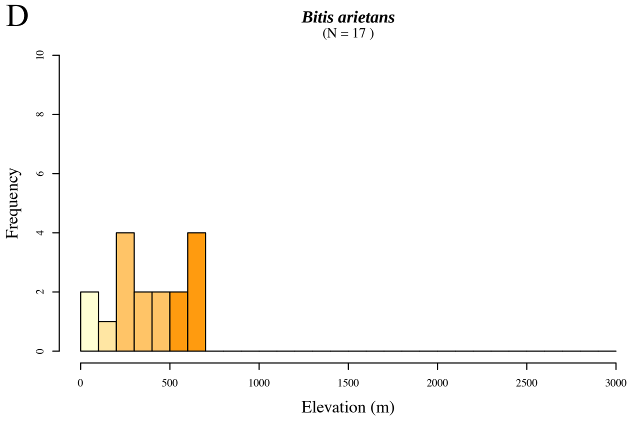

E

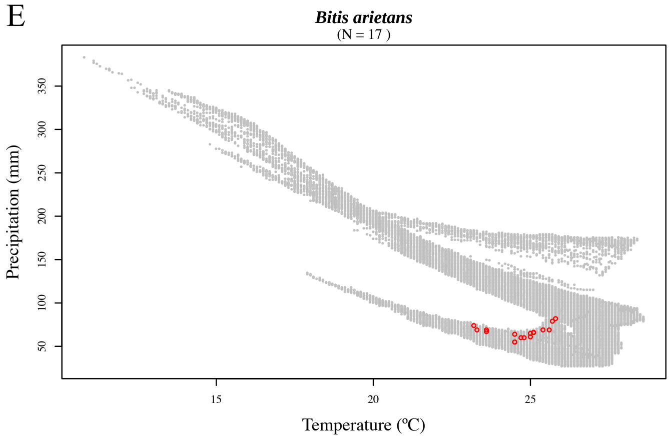

F

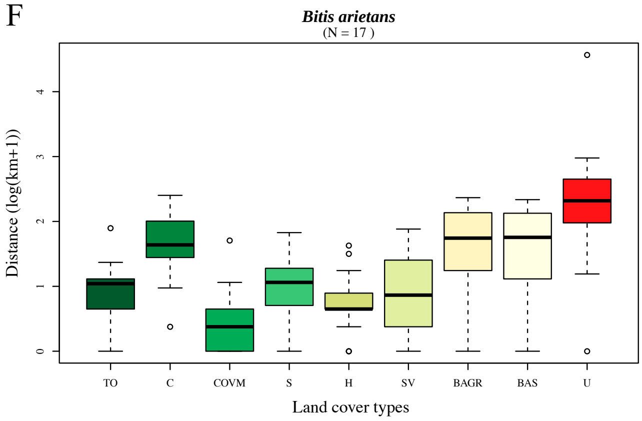

Cerastes gasperettii gasperettii
Leviton & Anderson, 1967

S96: Species information
Snakes
Viperidae

A

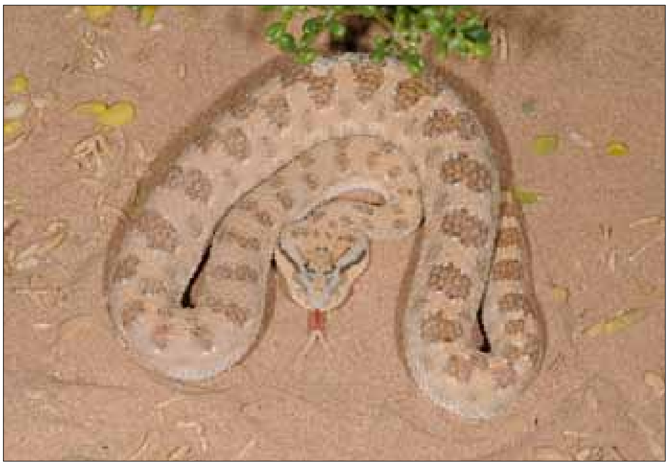

B

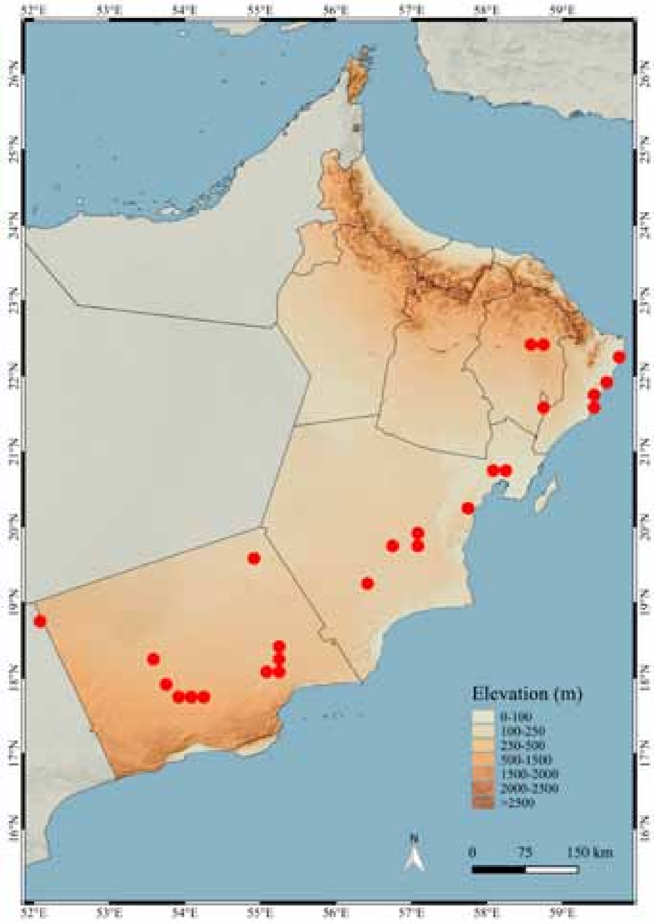

C

Useful information	
IUCN Category	LC
Endemic	NO
Venomous	YES
Insular	NO
Present in a protected area	YES

D

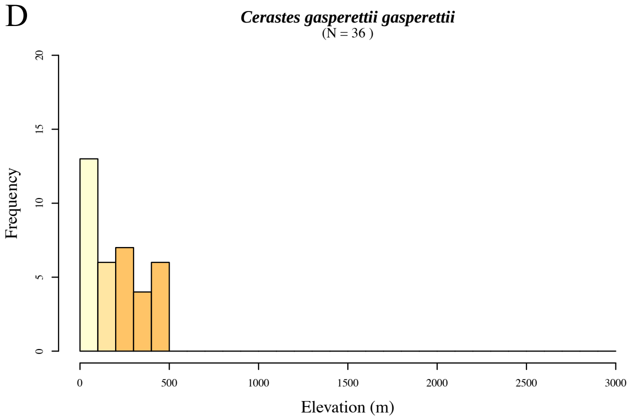

E

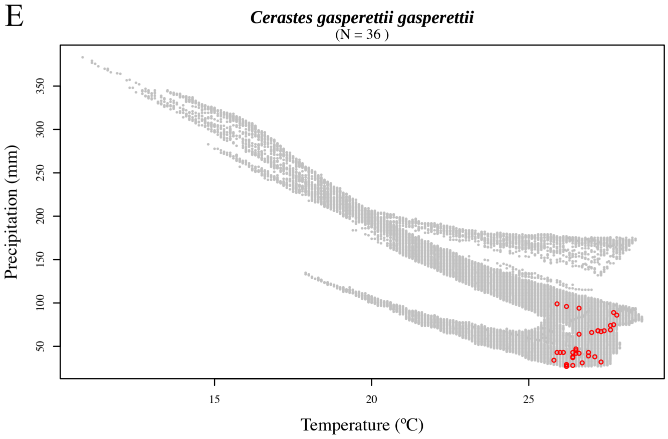

F

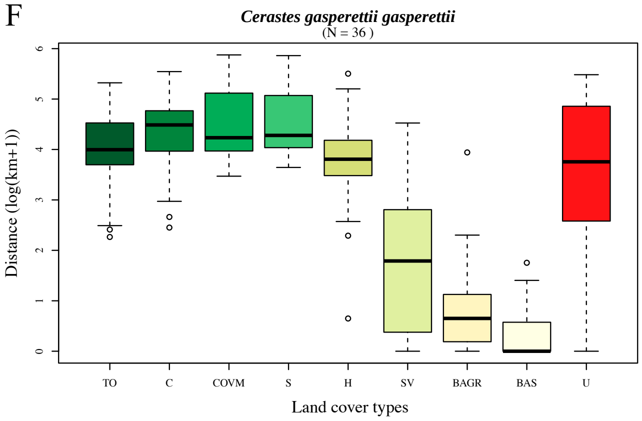

S97: Species information

Snakes

Viperidae

Echis carinatus sochureki

Stemmler, 1969

A

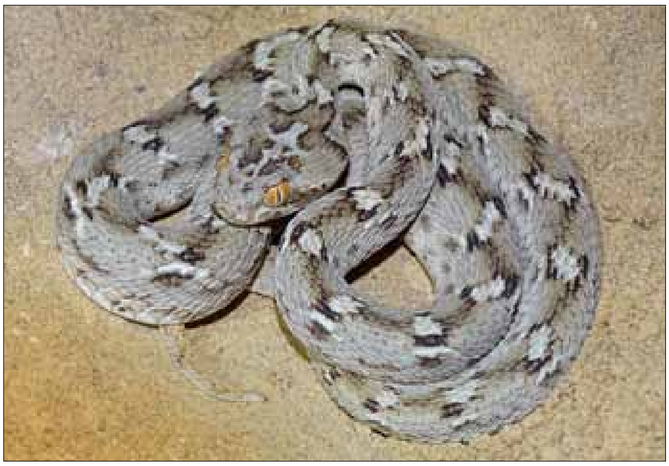

B

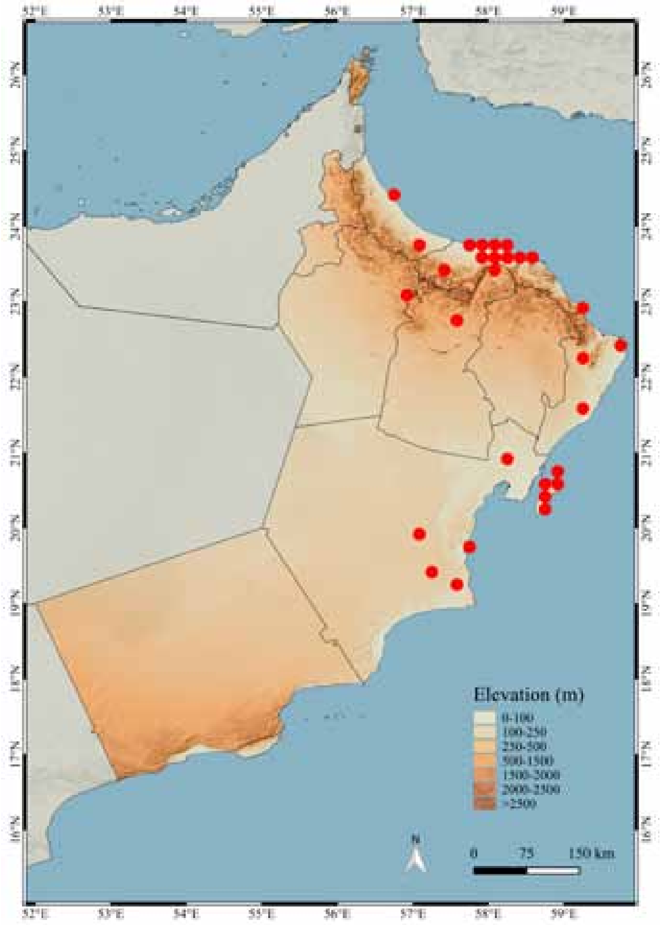

C

Useful information	
IUCN Category	LC*
Endemic	NO
Venomous	YES
Insular	YES
Present in a protected area	YES

*Not available on the web

D

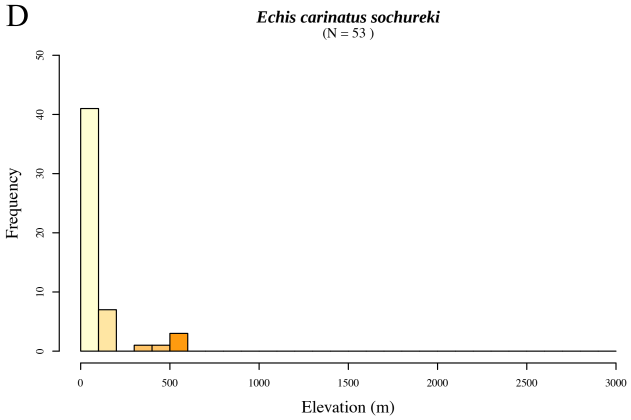

E

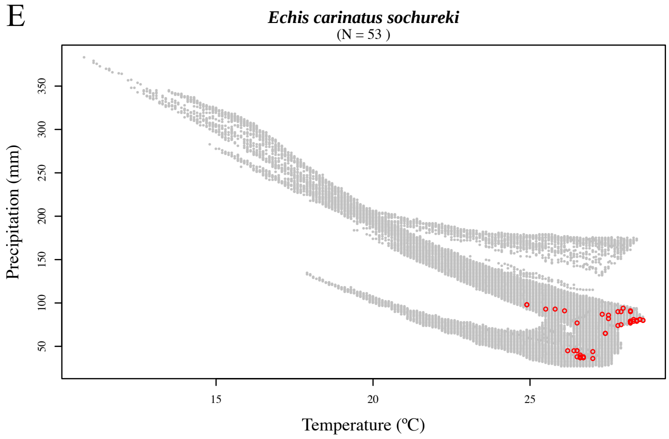

F

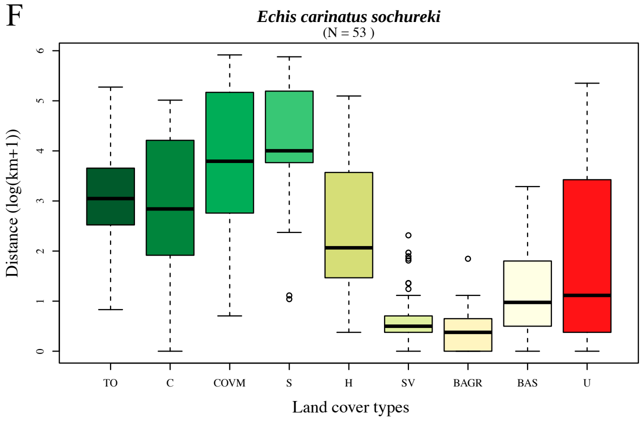

Echis coloratus
Günther, 1878

S98: Species information
Snakes
Viperidae

A

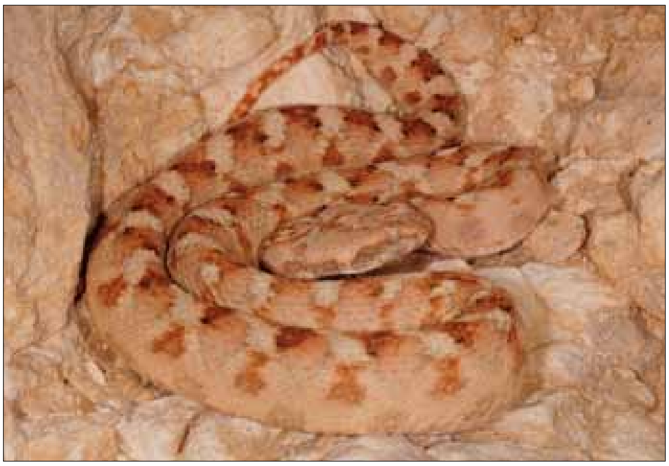

B

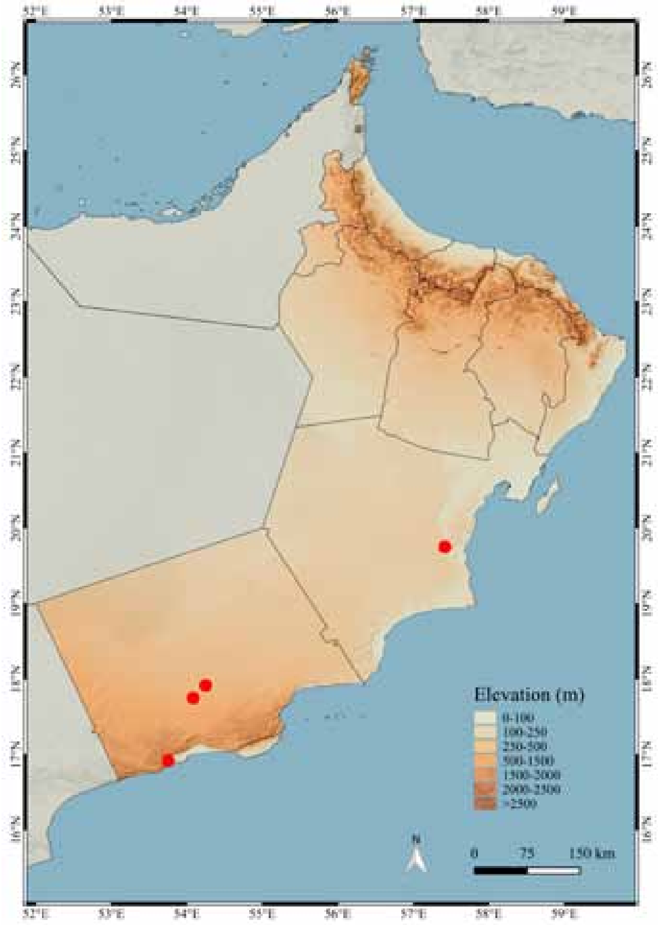

C

Useful information	
IUCN Category	LC*
Endemic	NO
Venomous	YES
Insular	NO
Present in a protected area	YES

*Not available on the web

D

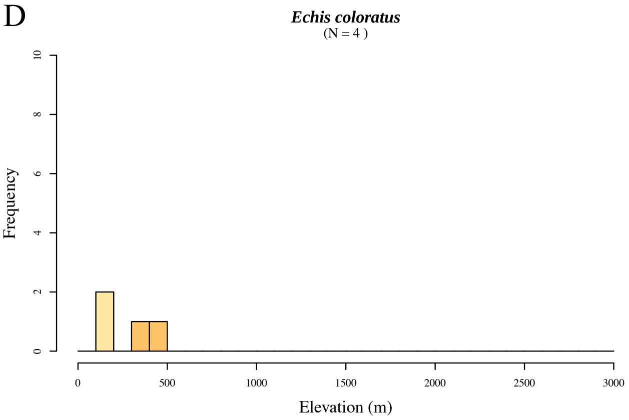

E

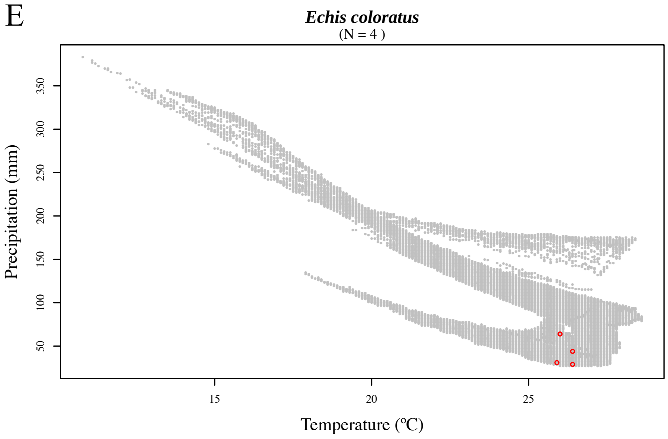

F

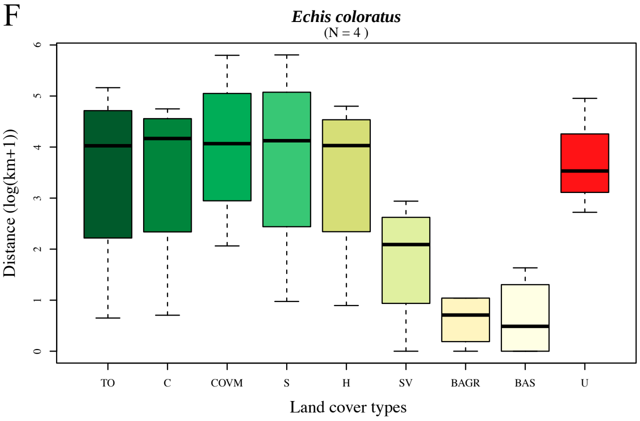

S99: Species information

Snakes

Viperidae

Echis khosatzkii
Cherlin, 1990

B

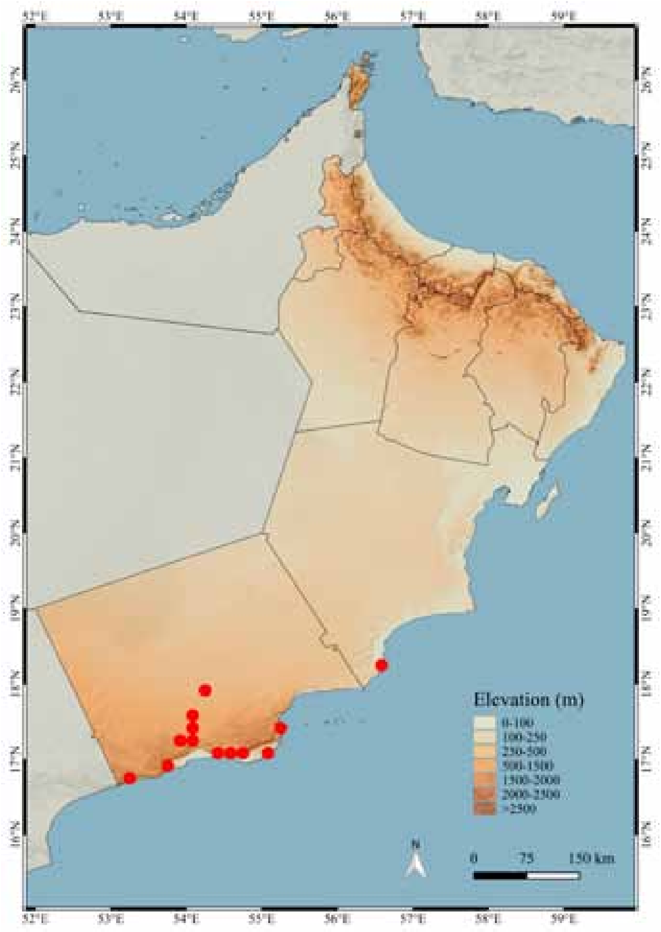

A

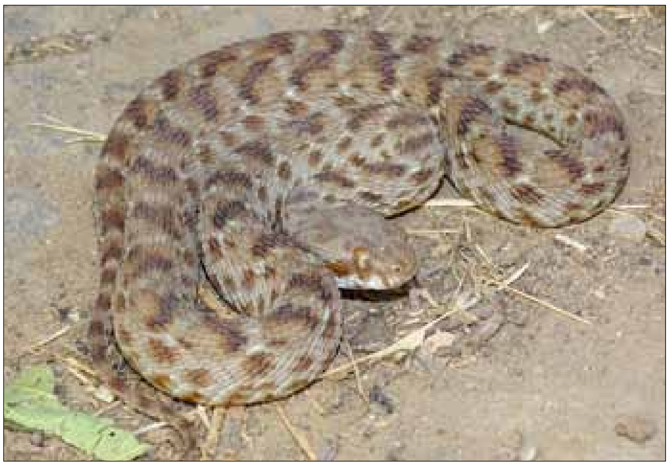

C

Useful information	
IUCN Category	LC
Endemic	NO
Venomous	YES
Insular	NO
Present in a protected area	YES

D

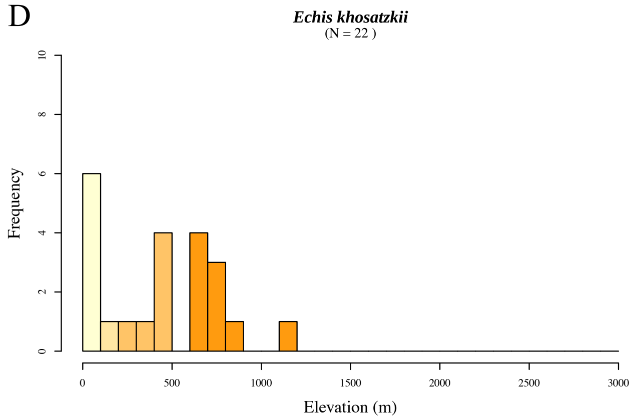

E

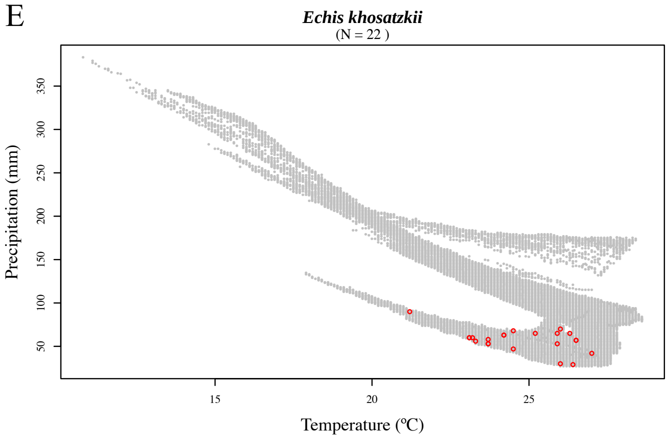

F

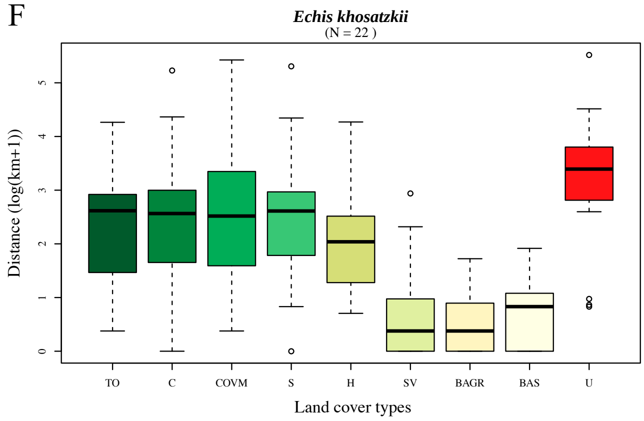

Echis omanensis
Babocsay, 2004

S100: Species information
Snakes
Viperidae

A

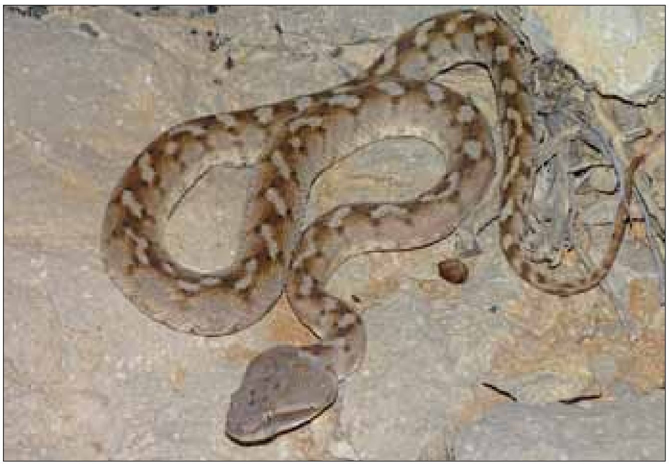

B

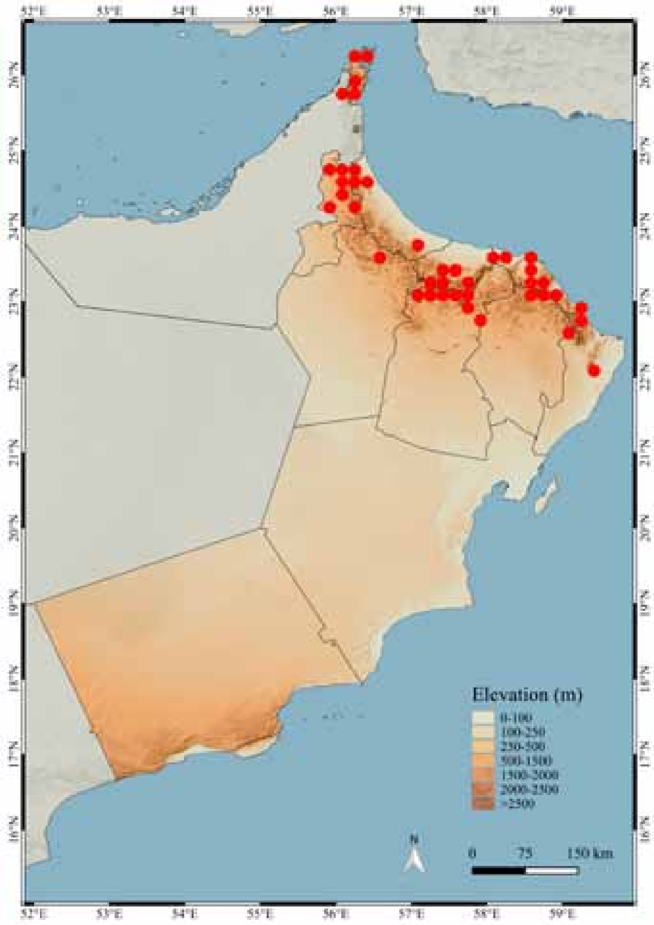

C

Useful information	
IUCN Category	LC
Endemic	NO
Venomous	YES
Insular	NO
Present in a protected area	YES

D

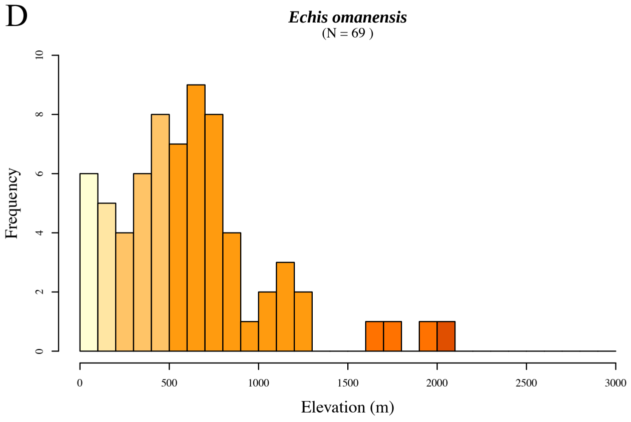

E

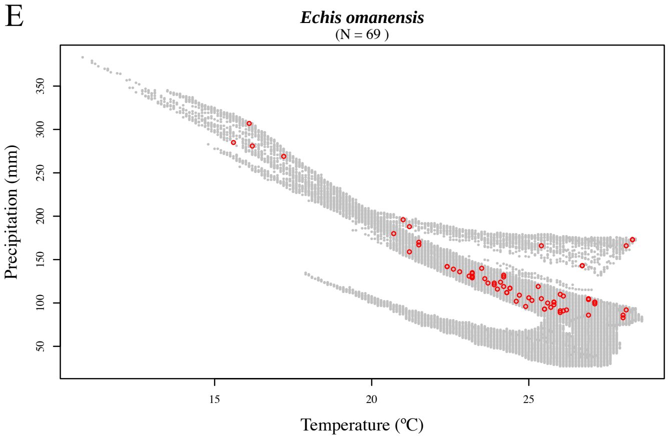

F

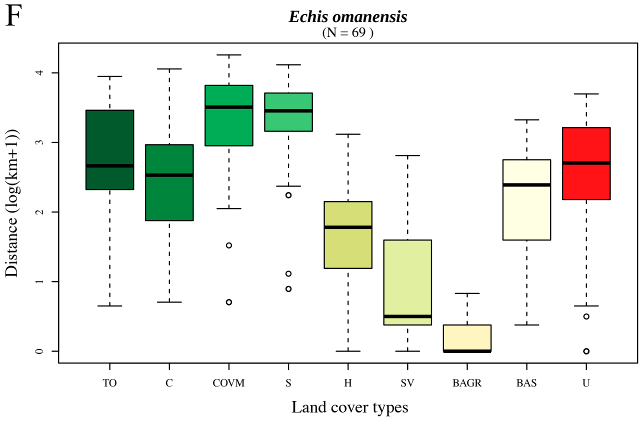

S101: Species information

Snakes

Viperidae

Pseudocerastes persicus

(Duméril, Bibron & Duméril, 1854)

A

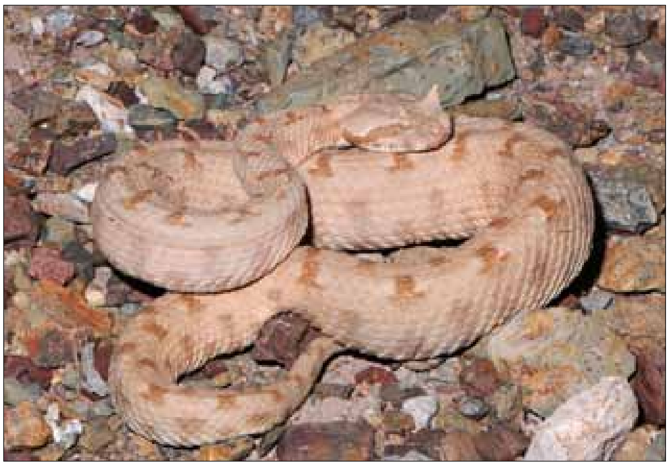

B

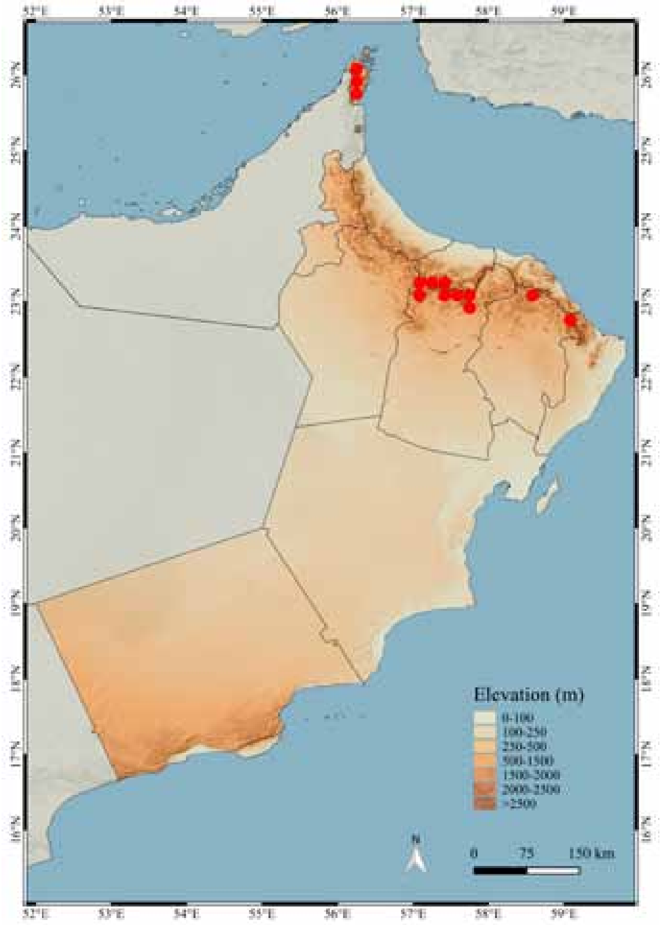

C

Useful information	
IUCN Category	LC
Endemic	NO
Venomous	YES
Insular	NO
Present in a protected area	YES

D

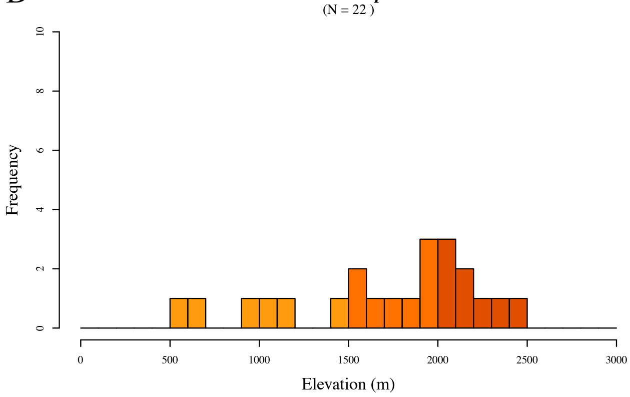

E

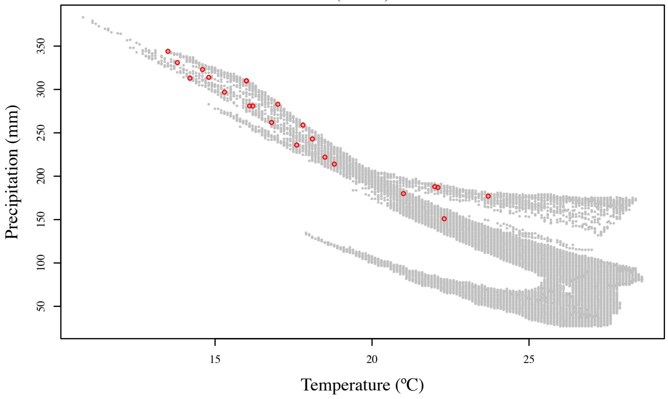

F

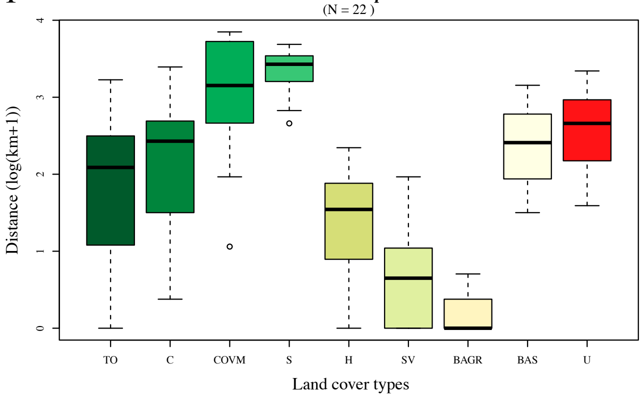

Snakebite Protocol

As shown in the present Atlas, the nine venomous terrestrial snakes belong to three different families: **Family Elapidae**: *Naja Arabica*; **Family Lamprophiidae**: *Atractaspis andersonii*; **Family Viperidae**: *Bitis arietans*, *Cerastes gasperettii gasperettii*, *Echis carinarus sochureki*, *Echis coloratus*, *Echis khosatzkii*, *Echis omanensis* and *Pseudocerastes persicus.*

The venoms of these nine species have different effects and it is very important that the bitten person is transported to a medical facility as quickly as possible. The following guidelines are only essential first-aid procedures.

1.- Move the victim to safety form the area where people might be bitten again. It is important to bear in mind that all nine venomous snakes of Oman are capable of biting repeatedly. If possible, take a picture of the snake for identification or try to remember some characteristics such as size, head shape and coloration.

2.- It is VERY IMPORTANT to calm down and reassure the victim, who may be terrified as a result of the snakebite. This step is crucial and is justified as in many occasions venomous snakes give “dry bites”, which are bites in which venoms is not injected and therefore they do not produce any medical complication. But even if the victim is envenomed, there should be enough time to arrive to the nearest medical facility in Oman.

3.- Immediately remove constricting clothing, rings, bracelets, bands, sock, shoes, etc. from the bitten limb, as in most occasions swelling occurs immediately after the bite.

4.- Immobilize the whole patient, especially the bitten limb, using a sling and try to keep the bitten limb below the level of the patient’s heart. Muscular contractions anywhere in the body but especially in the bitten limb help to spread the venom from the site of the bite, so it is very important that the patient does not move.

5.- Transport the patient to a medical facility without any further delay.

IT IS VERY IMPORTANT THAT AFTER A BITE:

1.- **DO NOT** give the victim any food water or medication until reaching a medical facility. If there is a considerable delay before reaching medical aid (several hours to days) then give clear fluids by mouth to prevent dehydration.

2.- **DO NOT** use Tourniquets, cut, suck, scarify, press or touch the wound or apply ice, hot water, chemicals or electric shocks. These measures are useless and potentially dangerous.

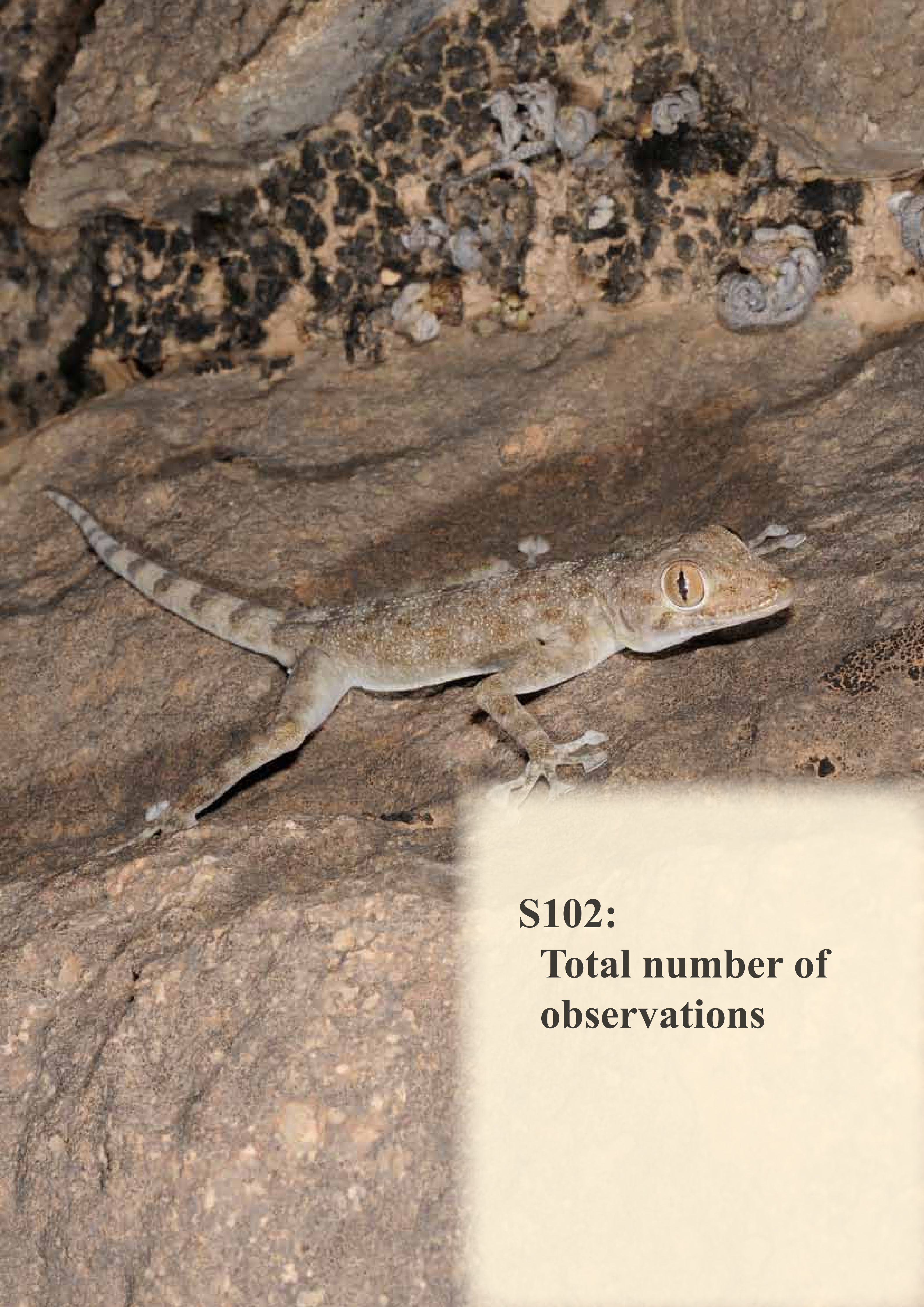

S102: Total number of observations

S102: Total number of observations

Species list of the terrestrials reptiles of Oman with the total number of observations and observations of each specie.

A

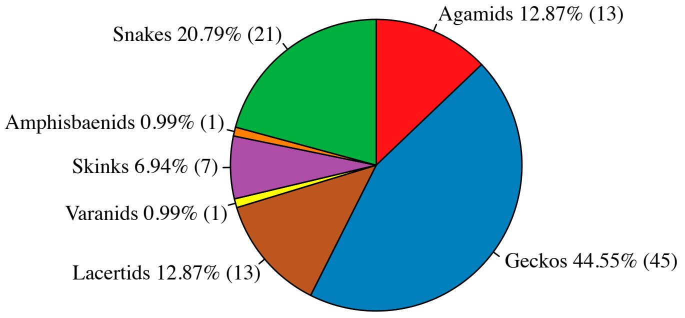

B

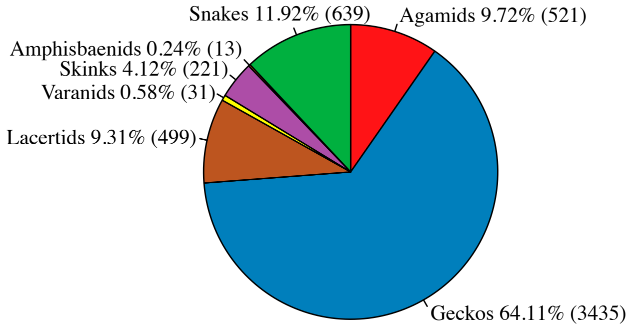

Main taxonomic groups observed. Percentage of the total number of A) species and B) observations in the seven main taxonomic groups of Oman reptiles in the database (between parenthesis, the respective number of observation).

S102: Total number of observations

Observations		
**Taxa**	**Family/Specie**	**Observations**
**Lizards**		**4720**
**Agamids**		**521**
	Agamidae	
	Acanthocercus adramitanus	26
	Calotes versicolor	43
	Phrynocephalus arabicus	18
	Phrynocephalus maculatus	15
	Phrynocephalus sakoi	44
	Pseudotrapelus dhofarensis	84
	Pseudotrapelus jensvindumi	100
	Trapelus flavimaculatus	61
	Uromastyx aegyptia leptieni	11
	Uromastyx aegyptia microlepis	37
	Uromastyx benti	15
	Uromastyx thomasi	20
	Chamaeleonidae	
	Chamaeleo arabicus	47
**Geckos**		**3435**
	Gekkonidae	
	Bunopus tuberculatus	102
	Cyrtopodion scabrum	26
	Hemidactylus alkiyumii	137
	Hemidactylus endophis	1
	Hemidactylus festivus	62
	Hemidactylus flaviviridis	55
	Hemidactylus hajarensis	65
	Hemidactylus inexpectatus	33
	Hemidactylus lemurinus	33
	Hemidactylus leschenaultii	3
	Hemidactylus luqueorum	55
	Hemidactylus masirahensis	10
	Hemidactylus minutus	70
	Hemidactylus paucituberculatus	44
	Hemidactylus persicus	1
	Hemidactylus robustus	135
	Hemidactylus sp.	1
	Pseudoceramodactylus khobarensis	15
	Stenodactylus arabicus	32
	Stenodactylus doriae	75
	Stenodactylus leptocosymbotes	151
	Stenodactylus sharqiyahensis	54
	Trachydactylus hajarensis	98
	Trachydactylus spatalurus	6
	Tropiocolotes scortecci	19
	Tropiocolotes sp.	9
	Phyllodactylidae	
	Asaccus gallagheri	41
	Asaccus gardneri	49
	Asaccus margaritae	9
	Asaccus montanus	36
	Asaccus platyrhynchus	92
	Asaccus arnoldi	18
	Ptyodactylus dhofarensis	72
	Ptyodactylus orlovi	206
	Ptyodactylus ruusaljibalicus	24
	Sphaerodactylidae	
	Pristurus carteri	305
	Pristurus celerrimus	152
	Pristurus gallagheri	59
	Pristurus minimus	155
	Pristurus rupestris rupestris	297
	Pristurus sp. 1	322
	Pristurus sp. 2	84
	Pristurus sp. 3	117
	Pristurus sp. 4	3
	Pristurus sp. 5	102

Observations		
**Taxa**	**Family/Specie**	**Observations**
**Lizards**		**4720**
**Lacertids**		**499**
	Lacertidae	
	Acanthodactylus blanfordii	27
	Acanthodactylus boskianus	25
	Acanthodactylus felicis	30
	Acanthodactylus haasi	6
	Acanthodactylus masirae	30
	Acanthodactylus opheodurus	27
	Acanthodactylus schmidti	62
	Mesalina adramitana	108
	Mesalina ayunensis	5
	Mesalina sp. 1	10
	Mesalina sp. 2	1
	Omanosaura cyanura	40
	Omanosaura jayakari	128
**Skinks**		**221**
	Scincidae	
	Ablepharus pannonicus	25
	Chalcides ocellatus ocellatus	28
	Heremites septemtaeniatus	2
	Scincus mitranus	70
	Scincus scincus conirostris	1
	Trachylepis brevicollis	33
	Trachylepis tessellata	62
**Amphisbaenids**		**13**
	Trogonophidae	
	Diplometopon zarudnyi	13
**Varanids**		**31**
	Varanidae	
	Varanus griseus	31
**Snakes**		**639**
	Boidae	
	Eryx jayakari	22
	Colubridae	
	Lytorhynchus diadema diadema	22
	Platyceps rhodorachis rhodorachis	149
	Platyceps thomasi	13
	Rhynchocalamus arabicus	2
	Spalerosophis diadema cliffordii	17
	Telescopus dhara dhara	36
	Elapidae	
	Naja arabica	16
	Lamprophiidae	
	Atractaspis andersonii	4
	Psammophis schokari	89
	Rhagerhis moilensis	25
	Leptotyphlopidae	
	Myriopholis macrorhyncha	16
	Myriopholis nursii	3
	Typhlopidae	
	Indotyphlops braminus	2
	Viperidae	
	Bitis arietans	17
	Cerastes gasperettii gasperettii	36
	Echis carinatus sochureki	53
	Echis coloratus	4
	Echis khosatzkii	22
	Echis omanensis	69
	Pseudocerastes persicus	22

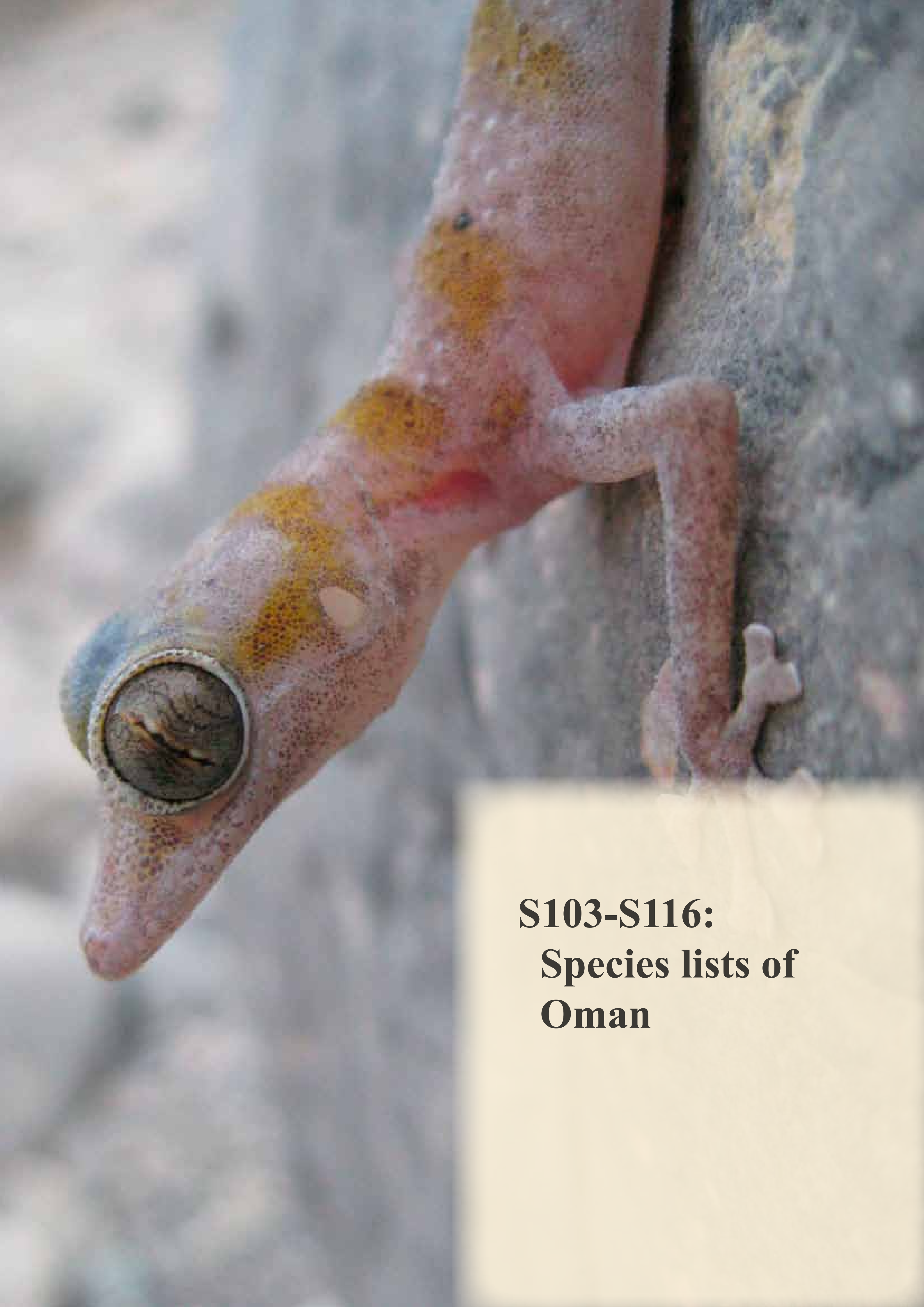

S103-S116: Species lists of Oman

S103-S116: Species lists of Oman

(S103-S104: Species lists of Oman) Map of Oman with the checklist of the terrestrial reptiles of Oman with their IUCN censervation categories with a list of the endemic, venomous and insular species.

(S105-S116: Species list by governorate) Map of the 11 governorates of Oman with the checklist of all the terrestrial reptile species present in the governorate with their IUCN conservation categories and with a list of the endemic, venomous and insular species.

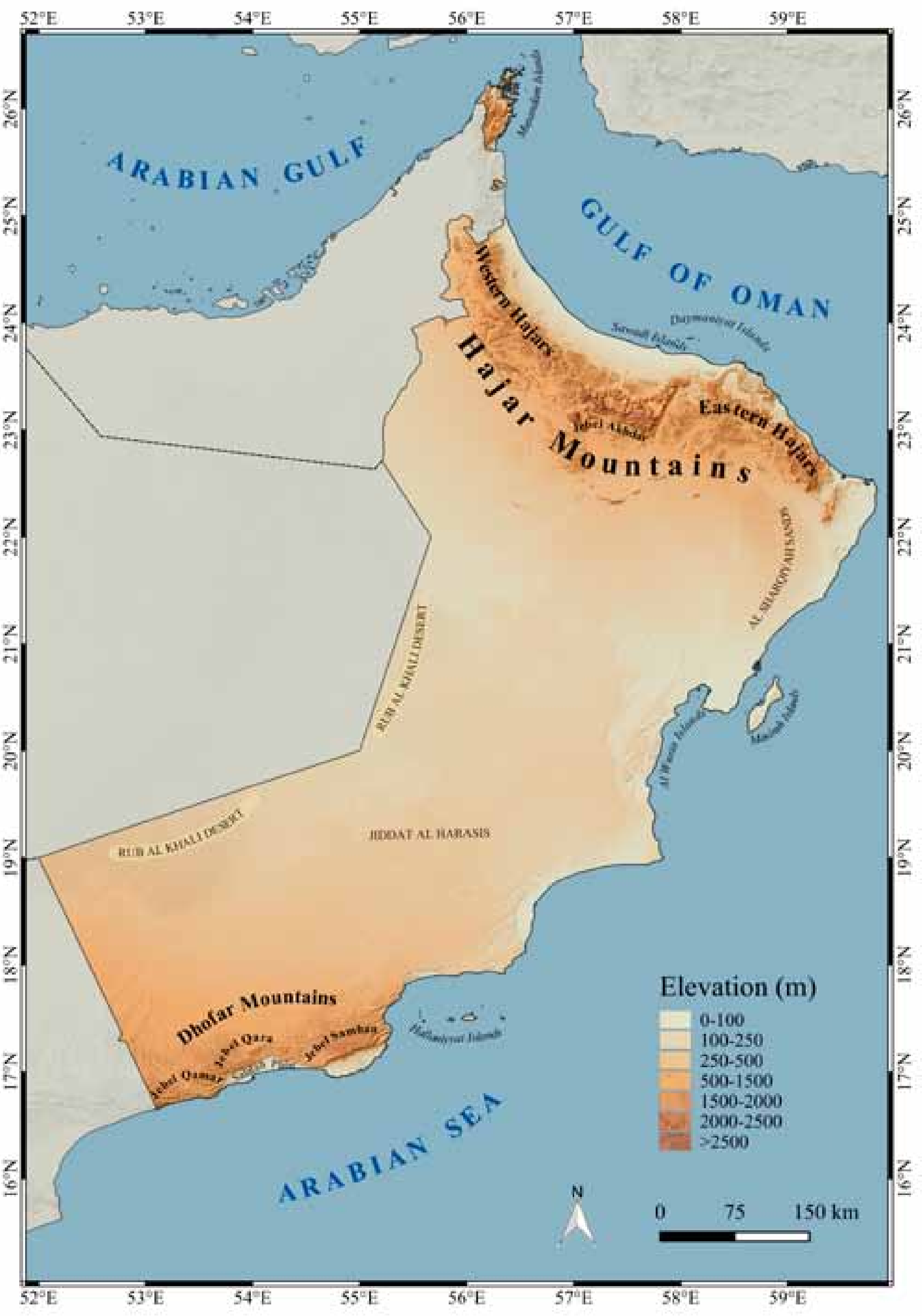

Physical map of Oman with altitude and names of the most relevant toponyms for this study.

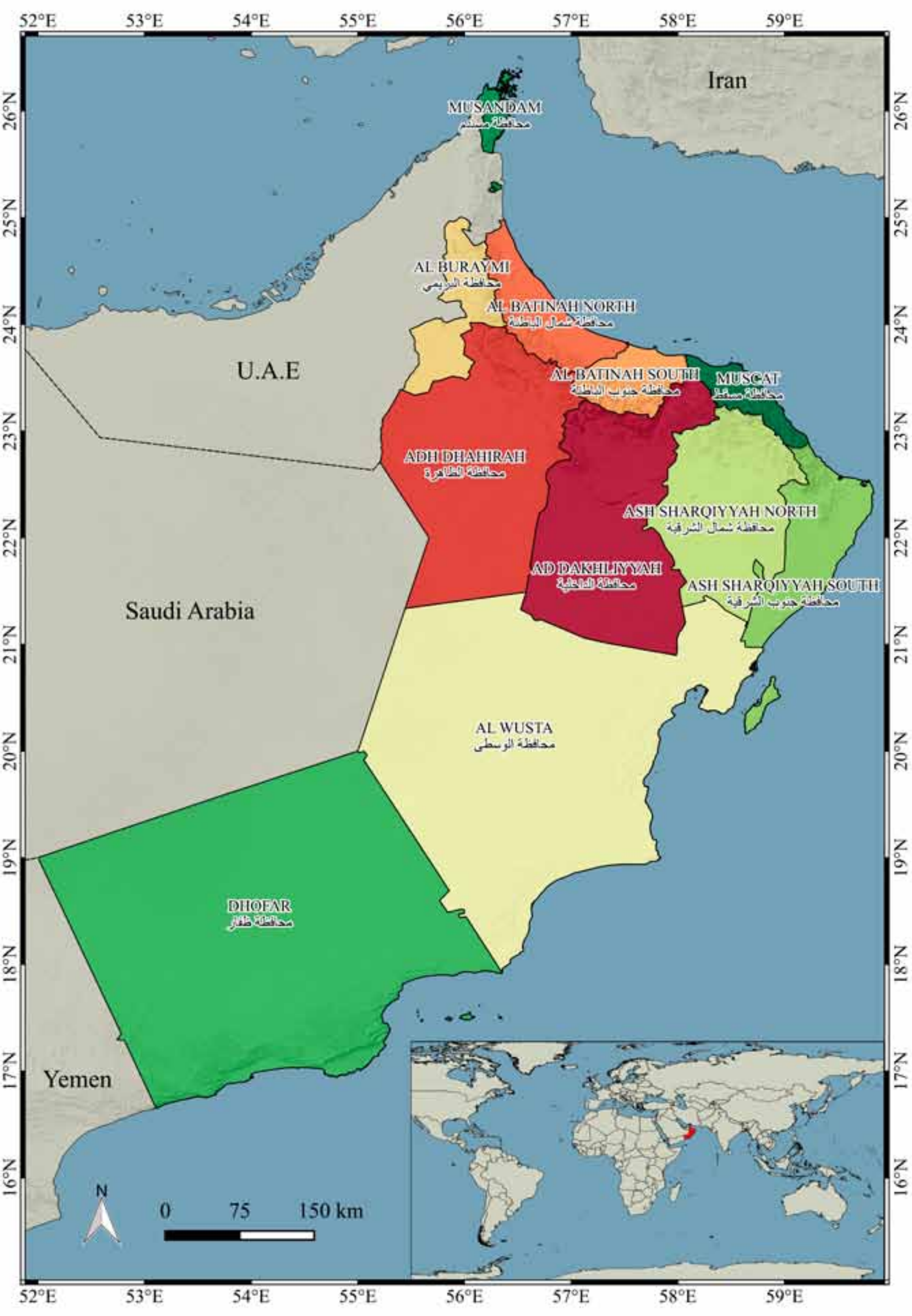

Political map of Oman showing the limits of the 11 governorates. Inlet map: Location of Oman (red) in the world map.

Oman

S103: Species list of Oman

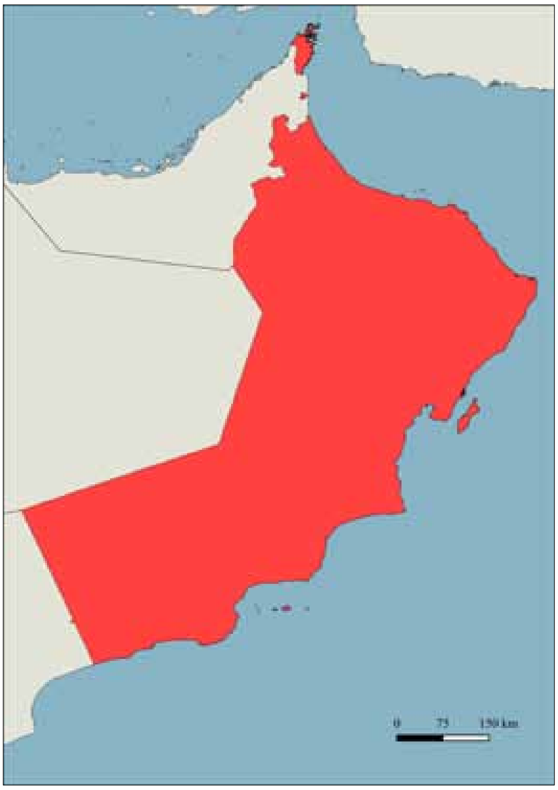

IUCN categories		
Taxa	Family/Specie	Total
**Lizards**		**80**
**Agamids**		**13**
	Agamidae	
	Acanthocercus adramitanus	LC
	Calotes versicolor	LC*
	Phrynocephalus arabicus	LC
	Phrynocephalus maculatus	LC*
	Phrynocephalus sakoi	NE
	Pseudotrapelus dhofarensis	NE
	Pseudotrapelus jensvindumi	NE
	Trapelus flavimaculatus	LC
	Uromastyx aegyptia leptieni	VU
	Uromastyx aegyptia microlepis	VU
	Uromastyx benti	LC
	Uromastyx thomasi	VU
	Chamaeleonidae	
	Chamaeleo arabicus	LC
**Geckos**		**45**
	Gekkonidae	
	Bunopus tuberculatus	LC
	Cyrtopodion scabrum	LC
	Hemidactylus alkiyumii	NE
	Hemidactylus endophis	NE
	Hemidactylus festivus	NE
	Hemidactylus flaviviridis	LC*
	Hemidactylus hajarensis	NE
	Hemidactylus inexpectatus	NE
	Hemidactylus lemurinus	DD
	Hemidactylus leschenaultii	LC*
	Hemidactylus luqueorum	NE
	Hemidactylus masirahensis	NE
	Hemidactylus minutus	NE
	Hemidactylus paucituberculatus	NE
	Hemidactylus persicus	LC*
	Hemidactylus robustus	LC*
	Hemidactylus sp.	NE
	Pseudoceramodactylus khobarensis	LC
	Stenodactylus arabicus	LC
	Stenodactylus doriae	LC
	Stenodactylus leptocosymbotes	LC*
	Stenodactylus sharqiyahensis	NE
	Trachydactylus hajarensis	NE
	Trachydactylus spatalurus	NE
	Tropiocolotes scortecci	LC
	Tropiocolotes sp.	NE
	Phyllodactylidae	
	Asaccus gallagheri	NE
	Asaccus gardneri	NE
	Asaccus margaritae	NE
	Asaccus montanus	VU
	Asaccus platyrhynchus	LC
	Asaccus arnoldi	NE
	Ptyodactylus dhofarensis	NE
	Ptyodactylus orlovi	NE
	Ptyodactylus ruusaljibalicus	NE
	Sphaerodactylidae	
	Pristurus carteri	LC
	Pristurus celerrimus	LC
	Pristurus gallagheri	NT
	Pristurus minimus	LC
	Pristurus rupestris rupestris	NE
	Pristurus sp. 1	NE
	Pristurus sp. 2	NE
	Pristurus sp. 3	NE
	Pristurus sp. 4	NE
	Pristurus sp. 5	NE
**Lacertids**		**13**
	Lacertidae	
	Acanthodactylus blanfordii	LC*
	Acanthodactylus boskianus	LC*
	Acanthodactylus felicis	VU
	Acanthodactylus haasi	LC
	Acanthodactylus masirae	DD
	Acanthodactylus opheodurus	LC
	Acanthodactylus schmidti	LC
	Mesalina adramitana	LC
	Mesalina ayunensis	DD
	Mesalina sp. 1	NE
	Mesalina sp. 2	NE
	Omanosaura cyanura	LC
	Omanosaura jayakari	LC
**Skinks**		**7**
	Scincidae	
	Ablepharus pannonicus	LC*
	Chalcides ocellatus ocellatus	LC*
	Heremites septemtaeniatus	LC*
	Scincus mitranus	LC
	Scincus scincus conirostris	LC*
	Trachylepis brevicollis	LC*
	Trachylepis tessellata	LC
**Amphisbaenids**		**1**
	Trogonophidae	
	Diplometopon zarudnyi	LC
**Varanids**		**1**
	Varanidae	
	Varanus griseus	LC*
**Snakes**		**21**
	Boidae	
	Eryx jayakari	LC
	Colubridae	
	Lytorhynchus diadema diadema	LC
	Platyceps rhodorachis rhodorachis	LC*
	Platyceps thomasi	DD
	Rhynchocalamus arabicus	DD
	Spalerosophis diadema cliffordii	LC*
	Telescopus dhara dhara	LC*
	Elapidae	
	Naja arabica	LC
	Lamprophiidae	
	Atractaspis andersonii	LC
	Psammophis schokari	LC*
	Rhagerhis moilensis	LC*
	Leptotyphlopidae	
	Myriopholis macrorhyncha	LC*
	Myriopholis nursii	LC*
	Typhlopidae	
	Indotyphlops braminus	LC*
	Viperidae	
	Bitis arietans	LC*
	Cerastes gasperettii gasperettii	LC
	Echis carinatus sochureki	LC*
	Echis coloratus	LC*
	Echis khosatzkii	LC
	Echis omanensis	LC
	Pseudocerastes persicus	LC

* Not available on the web

S104: Species list of Oman

Oman

Venomous species		
**Taxa**	**Family/Specie**	**Total**
**Lizards**		**0**
**Snakes**		**9**
	Elapidae	
	Naja arabica	
	Lamprophiidae	
	Atractaspis andersonii	
	Viperidae	
	Bitis arietans	
	Cerastes gasperettii gasperettii	
	Echis carinatus sochureki	
	Echis coloratus	
	Echis khosatzkii	
	Echis omanensis	
	Pseudocerastes persicus	

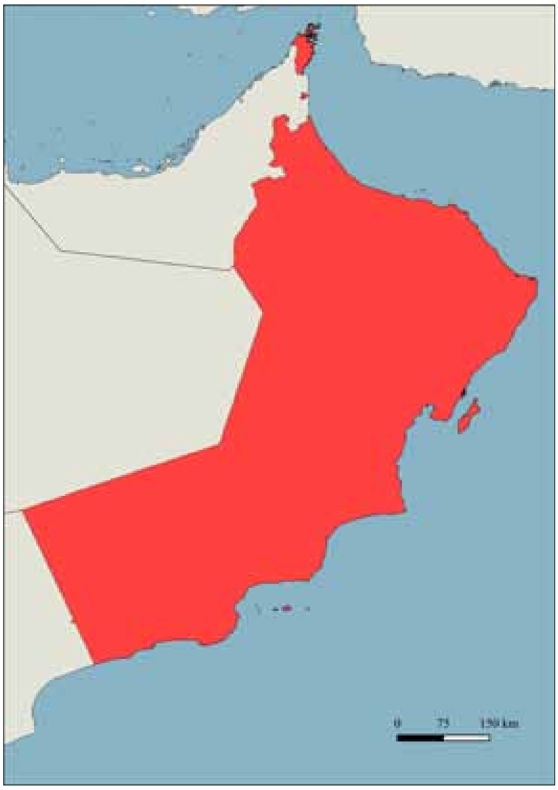

Insular species		
**Taxa**	**Family/Specie**	**Total**
**Lizards**		**23**
**Agamids**		**3**
	Agamidae	
	Pseudotrapelus dhofarensis	
	Uromastyx thomasi	
	Chamaeleonidae	
	Chamaeleo arabicus	
**Geckos**		**14**
	Gekkonidae	
	Hemidactylus alkiyumii	
	Hemidactylus inexpectatus	
	Hemidactylus masirahensis	
	Hemidactylus paucituberculatus	
	Hemidactylus robustus	
	Stenodactylus leptocosymbotes	
	Trachydactylus hajarensis	
	Tropiocolotes scortecci	
	Phyllodactylidae	
	Ptyodactylus ruusaljibalicus	
	Sphaerodactylidae	
	Pristurus carteri	
	Pristurus celerrimus	
	Pristurus minimus	
	Pristurus rupestris rupestris	
	Pristurus sp. 1	
**Lacertids**		**2**
	Lacertidae	
	Acanthodactylus masirae	
	Mesalina adramitana	
**Skinks**		**4**
	Scincidae	
	Chalcides ocellatus ocellatus	
	Heremites septemtaeniatus	
	Scincus mitranus	
	Trachylepis tessellata	
**Snakes**		**6**
	Colubridae	
	Platyceps rhodorachis rhodorachis	
	Spalerosophis diadema cliffordii	
	Telescopus dhara dhara	
	Lamprophiidae	
	Psammophis schokari	
	Rhagerhis moilensis	
	Viperidae	
	Echis carinatus sochureki	

Endemic species		
**Taxa**	**Family/Specie**	**Total**
**Lizards**		**20**
**Agamids**		**2**
	Agamidae	
	Phrynocephalus sakoi	
	Uromastyx thomasi	
**Geckos**		**16**
	Gekkonidae	
	Hemidactylus endophis	
	Hemidactylus hajarensis	
	Hemidactylus inexpectatus	
	Hemidactylus luqueorum	
	Hemidactylus masirahensis	
	Hemidactylus paucituberculatus	
	Hemidactylus sp.	
	Stenodactylus sharqiyahensis	
	Tropiocolotes sp.	
	Phyllodactylidae	
	Asaccus montanus	
	Asaccus platyrhynchus	
	Asaccus arnoldi	
	Sphaerodactylidae	
	Pristurus gallagheri	
	Pristurus sp. 2	
	Pristurus sp. 4	
	Pristurus sp. 5	
**Lacertids**		**2**
	Lacertidae	
	Acanthodactylus masirae	
	Mesalina sp. 2	
**Snakes**		**0**

Musandam Governorate

S105: Species list by governorate

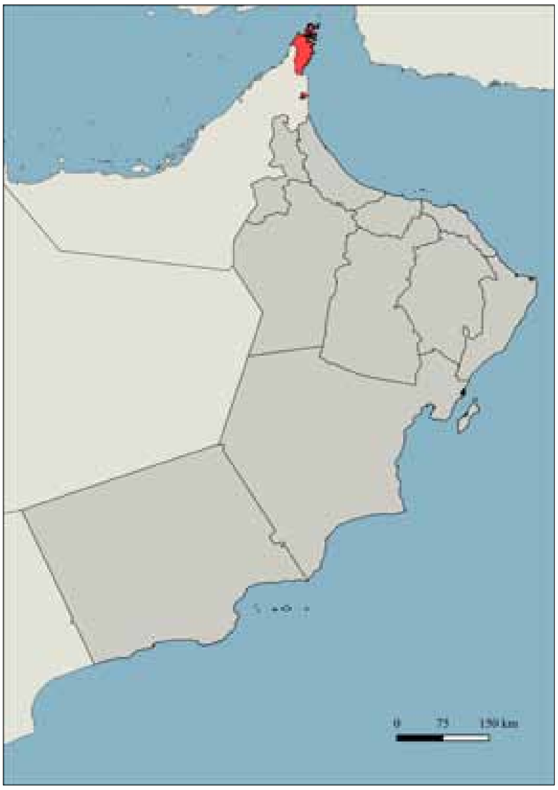

IUCN categories		
**Taxa**	**Family/Specie**	**Total**
**Lizards**		**15**
**Agamids**		**1**
	Agamidae	
	Pseudotrapelus jensvindumi	NE
**Geckos**		**10**
	Gekkonidae	
	Cyrtopodion scabrum	LC
	Hemidactylus flaviviridis	LC*
	Hemidactylus robustus	LC*
	Trachydactylus hajarensis	NE
	Phyllodactylidae	
	Asaccus gallagheri	NE
	Asaccus gardneri	NE
	Asaccus margaritae	NE
	Ptyodactylus ruusaljibalicus	NE
	Sphaerodactylidae	
	Pristurus celerrimus	LC
	Pristurus sp. 3	NE
**Lacertids**		**2**
	Lacertidae	
	Omanosaura cyanura	LC
	Omanosaura jayakari	LC
**Skinks**		**2**
	Scincidae	
	Chalcides ocellatus ocellatus	LC*
	Trachylepis tessellata	LC
**Snakes**		**5**
	Colubridae	
	Platyceps rhodorachis rhodorachis	LC*
	Telescopus dhara dhara	LC*
	Lamprophiidae	
	Psammophis schokari	LC*
	Viperidae	
	Echis omanensis	LC
	Pseudocerastes persicus	LC

* Not available on the web

Venomous species		
**Taxa**	**Family/Specie**	**Total**
**Lizards**		**0**
**Snakes**		**2**
	Viperidae	
	Echis omanensis	
	Pseudocerastes persicus	

Species of Musandam Islands		
**Taxa**	**Family/Specie**	**Total**
**Lizards**		**3**
**Geckos**		**3**
	Gekkonidae	
	Hemidactylus robustus	
	Phyllodactylidae	
	Ptyodactylus ruusaljibalicus	
	Sphaerodactylidae	
	Pristurus celerrimus	
**Snakes**		**0**

S106: Species list by governorate

Al Buraymi Governorate

IUCN categories		
**Taxa**	**Family/Specie**	**Total**
**Lizards**		**24**
**Agamids**		**4**
	Agamidae	
	Phrynocephalus arabicus	LC
	Pseudotrapelus jensvindumi	NE
	Trapelus flavimaculatus	LC
	Uromastyx aegyptia leptieni	VU
**Geckos**		**11**
	Gekkonidae	
	Bunopus tuberculatus	LC
	Hemidactylus persicus	LC*
	Hemidactylus robustus	LC*
	Stenodactylus arabicus	LC
	Stenodactylus doriae	LC
	Stenodactylus leptocosymbotes	LC*
	Trachydactylus hajarensis	NE
	Phyllodactylidae	
	Ptyodactylus orlovi	NE
	Sphaerodactylidae	
	Pristurus carteri	LC
	Pristurus celerrimus	LC
	Pristurus sp. 3	NE
**Lacertids**		**5**
	Lacertidae	
	Acanthodactylus boskianus	LC*
	Acanthodactylus schmidti	LC
	Mesalina adramitana	LC
	Omanosaura cyanura	LC
	Omanosaura jayakari	LC
**Skinks**		**3**
	Scincidae	
	Ablepharus pannonicus	LC*
	Scincus mitranus	LC
	Trachylepis tessellata	LC
**Amphisbaenids**		**1**
	Trogonophidae	
	Diplometopon zarudnyi	LC
**Snakes**		**4**
	Boidae	
	Eryx jayakari	LC
	Colubridae	
	Platyceps rhodorachis rhodorachis	LC*
	Lamprophiidae	
	Psammophis schokari	LC*
	Viperidae	
	Echis omanensis	LC

* Not available on the web

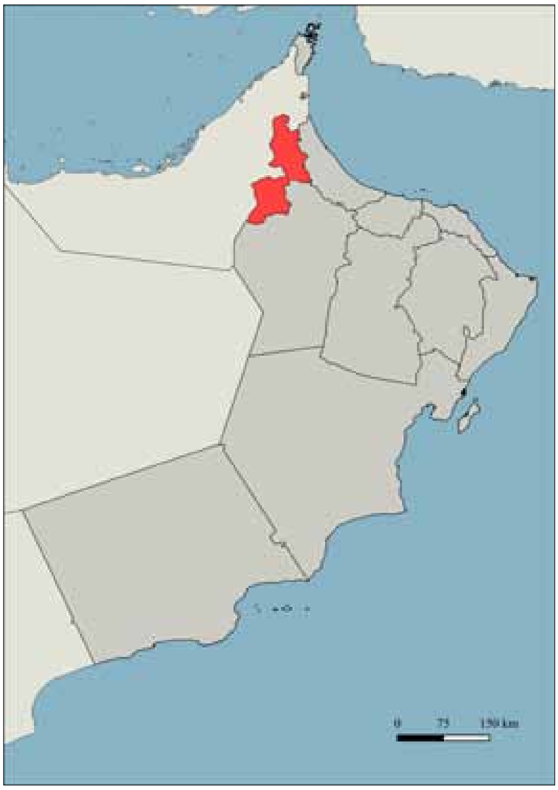

Venomous species		
**Taxa**	**Family/Specie**	**Total**
**Lizards**		**0**
**Snakes**		**1**
	Echis omanensis	

Al Batinah North Governorate

S107: Species list by governorate

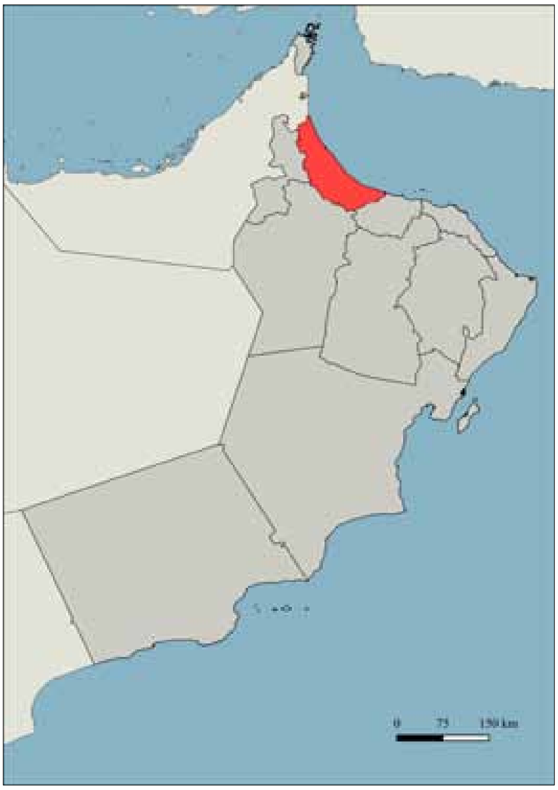

IUCN categories		
**Taxa**	**Family/Specie**	**Total**
**Lizards**		**20**
**Agamids**		**2**
	Agamidae	
	Pseudotrapelus jensvindumi	NE
	Trapelus flavimaculatus	LC
**Geckos**		**10**
	Gekkonidae	
	Bunopus tuberculatus	LC
	Hemidactylus flaviviridis	LC*
	Hemidactylus robustus	LC*
	Stenodactylus leptocosymbotes	LC*
	Trachydactylus hajarensis	NE
	Phyllodactylidae	
	Asaccus gallagheri	NE
	Ptyodactylus orlovi	NE
	Sphaerodactylidae	
	Pristurus celerrimus	LC
	Pristurus rupestris rupestris	NE
	Pristurus sp. 3	NE
**Lacertids**		**5**
	Lacertidae	
	Acanthodactylus blanfordii	LC*
	Acanthodactylus opheodurus	LC
	Mesalina adramitana	LC
	Omanosaura cyanura	LC
	Omanosaura jayakari	LC
**Skinks**		**2**
	Scincidae	
	Chalcides ocellatus ocellatus	LC*
	Trachylepis tessellata	LC
**Varanids**		**1**
	Varanidae	
	Varanus griseus	LC*
**Snakes**		**8**
	Boidae	
	Eryx jayakari	LC
	Colubridae	
	Lytorhynchus diadema diadema	LC
	Platyceps rhodorachis rhodorachis	LC*
	Spalerosophis diadema cliffordii	LC*
	Lamprophiidae	
	Psammophis schokari	LC*
	Rhagerhis moilensis	LC*
	Viperidae	
	Echis carinatus sochureki	LC*
	Echis omanensis	LC

* Not available on the web

Venomous species		
**Taxa**	**Family/Specie**	**Total**
**Lizards**		**0**
**Snakes**		**2**
	Viperidae	
	Echis carinatus sochureki	
	Echis omanensis	

S108: Species list by governorate

Al Batinah South Governorate

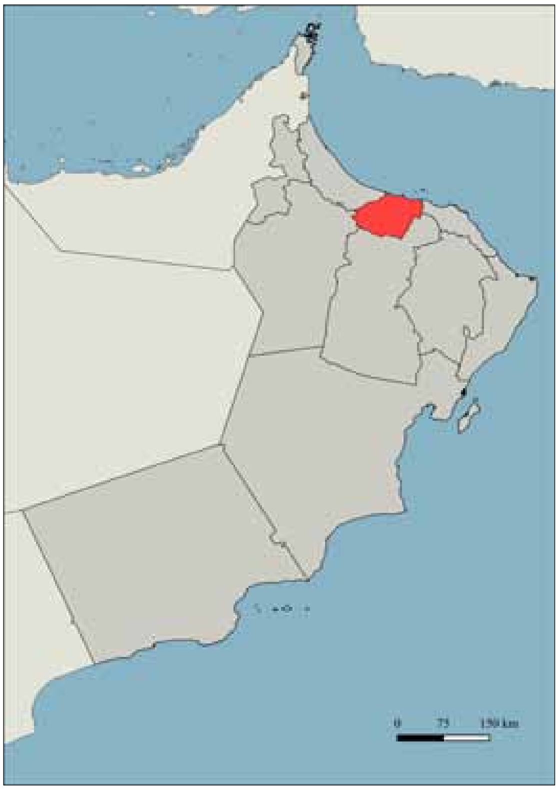

IUCN categories		
**Taxa**	**Family/Specie**	**Total**
**Lizards**		**29**
**Agamids**		**3**
	Agamidae	
	Pseudotrapelus jensvindumi	NE
	Trapelus flavimaculatus	LC
	Uromastyx aegyptia leptieni	VU
**Geckos**		**16**
	Gekkonidae	
	Bunopus tuberculatus	LC
	Hemidactylus flaviviridis	LC*
	Hemidactylus hajarensis	NE
	Hemidactylus leschenaultii	LC*
	Hemidactylus luqueorum	NE
	Hemidactylus robustus	LC*
	Stenodactylus leptocosymbotes	LC*
	Trachydactylus hajarensis	NE
	Phyllodactylidae	
	Asaccus gallagheri	NE
	Asaccus platyrhynchus	LC
	Ptyodactylus orlovi	NE
	Sphaerodactylidae	
	Pristurus carteri	LC
	Pristurus celerrimus	LC
	Pristurus gallagheri	NT
	Pristurus rupestris rupestris	NE
	Pristurus sp. 3	NE
**Lacertids**		**4**
	Lacertidae	
	Acanthodactylus blanfordii	LC*
	Mesalina adramitana	LC
	Omanosaura cyanura	LC
	Omanosaura jayakari	LC
**Skinks**		**5**
	Scincidae	
	Ablepharus pannonicus	LC*
	Chalcides ocellatus ocellatus	LC*
	Heremites septemtaeniatus	LC*
	Scincus mitranus	LC
	Trachylepis tessellata	LC
**Varanids**		**1**
	Varanidae	
	Varanus griseus	LC*
**Snakes**		**11**
	Boidae	
	Eryx jayakari	LC
	Colubridae	
	Platyceps rhodorachis rhodorachis	LC*
	Spalerosophis diadema cliffordii	LC*
	Telescopus dhara dhara	LC*
	Lamprophiidae	
	Psammophis schokari	LC*
	Rhagerhis moilensis	LC*
	Leptotyphlopidae	
	Myriopholis macrorhyncha	LC*
	Typhlopidae	
	Indotyphlops braminus	LC*
	Viperidae	
	Echis carinatus sochureki	LC*
	Echis omanensis	LC
	Pseudocerastes persicus	LC

* Not available on the web

Venomous species		
**Taxa**	**Family/Specie**	**Total**
**Lizards**		**0**
**Snakes**		**3**
	Viperidae	
	Echis carinatus sochureki	
	Echis omanensis	
	Pseudocerastes persicus	

Species of Daymaniyat Islands		
**Taxa**	**Family/Specie**	**Total**
**Lizards**		**3**
**Geckos**		**2**
	Gekkonidae	
	Hemidactylus robustus	
	Sphaerodactylidae	
	Pristurus rupestris rupestris	
**Skinks**		**1**
	Scincidae	
	Heremites septemtaeniatus	
**Snakes**		**1**
	Colubridae	
	Platyceps rhodorachis rhodorachis	

Endemic species		
**Taxa**	**Family/Specie**	**Total**
**Lizards**		**4**
**Geckos**		**4**
	Gekkonidae	
	Hemidactylus hajarensis	
	Hemidactylus luqueorum	
	Phyllodactylidae	
	Asaccus platyrhynchus	
	Sphaerodactylidae	
	Pristurus gallagheri	
**Snakes**		**0**

Species of Sawadi Islands		
**Taxa**	**Family/Specie**	**Total**
**Lizards**		**3**
**Geckos**		**1**
	Sphaerodactylidae	
	Pristurus rupestris rupestris	
**Lacertids**		**1**
	Lacertidae	
	Mesalina adramitana	
**Skinks**		**1**
	Scincidae	
	Chalcides ocellatus ocellatus	
**Snakes**		**1**
	Viperidae	
	Echis carinatus sochureki	

Adh Dhahirah Governorate

S109: Species list by governorate

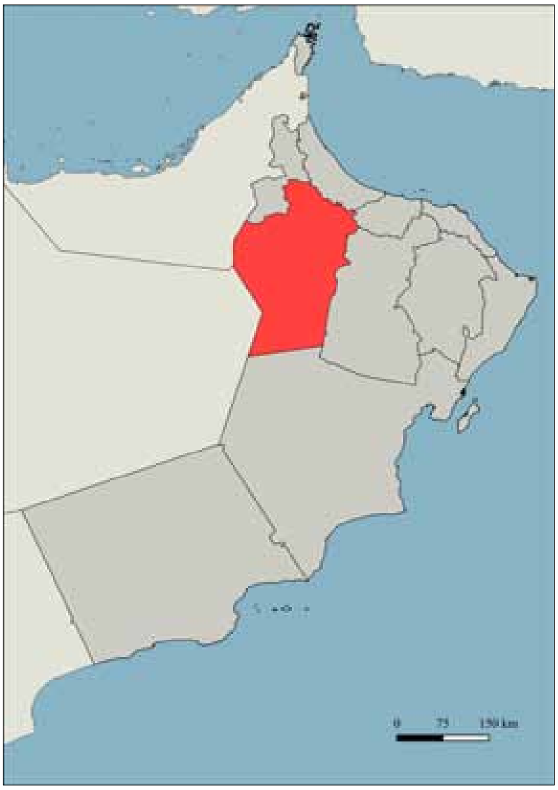

Venomous species		
**Taxa**	**Family/Specie**	**Total**
**Lizards**		**0**
**Snakes**		**2**
	Viperidae	
	Echis carinatus sochureki	
	Echis omanensis	

Endemic species		
**Taxa**	**Family/Specie**	**Total**
**Lizards**		**3**
**Geckos**		**3**
	Gekkonidae	
	Hemidactylus luqueorum	
	Phyllodactylidae	
	Asaccus montanus	
	Sphaerodactylidae	
	Pristurus sp. 5	
**Snakes**		**0**

IUCN categories		
**Taxa**	**Family/Specie**	**Total**
**Lizards**		**27**
**Agamids**		**1**
	Agamidae	
	Pseudotrapelus jensvindumi	NE
**Geckos**		**16**
	Gekkonidae	
	Bunopus tuberculatus	LC
	Hemidactylus flaviviridis	LC*
	Hemidactylus luqueorum	NE
	Stenodactylus arabicus	LC
	Stenodactylus doriae	LC
	Stenodactylus leptocosymbotes	LC*
	Trachydactylus hajarensis	NE
	Phyllodactylidae	
	Asaccus gallagheri	NE
	Asaccus montanus	VU
	Ptyodactylus orlovi	NE
	Sphaerodactylidae	
	Pristurus carteri	LC
	Pristurus celerrimus	LC
	Pristurus minimus	LC
	Pristurus rupestris rupestris	NE
	Pristurus sp. 3	NE
	Pristurus sp. 5	NE
**Lacertids**		**5**
	Lacertidae	
	Acanthodactylus boskianus	LC*
	Acanthodactylus opheodurus	LC
	Acanthodactylus schmidti	LC
	Mesalina adramitana	LC
	Omanosaura jayakari	LC
**Skinks**		**4**
	Scincidae	
	Ablepharus pannonicus	LC*
	Scincus mitranus	LC
	Scincus scincus conirostris	LC*
	Trachylepis tessellata	LC
**Varanids**		**1**
	Varanidae	
	Varanus griseus	LC*
**Snakes**		**6**
	Colubridae	
	Platyceps rhodorachis rhodorachis	LC*
	Spalerosophis diadema cliffordii	LC*
	Lamprophiidae	
	Psammophis schokari	LC*
	Rhagerhis moilensis	LC*
	Viperidae	
	Echis carinatus sochureki	LC*
	Echis omanensis	LC

* Not available on the web

S110: Species list by governorate

Ad Dakhliyyah Governorate

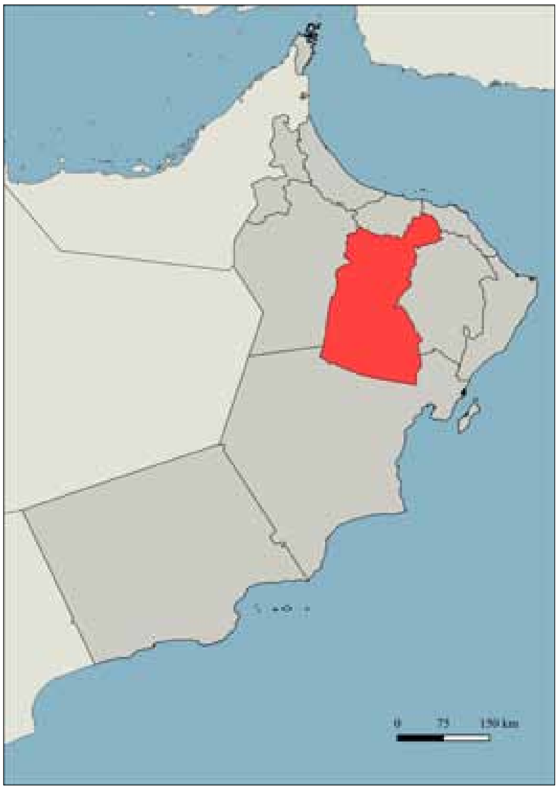

IUCN categories		
**Taxa**	**Family/Specie**	**Total**
**Lizards**		**35**
**Agamids**		**5**
	Agamidae	
	Pseudotrapelus dhofarensis	NE
	Pseudotrapelus jensvindumi	NE
	Trapelus flavimaculatus	LC
	Uromastyx aegyptia leptieni	VU
	Uromastyx aegyptia microlepis	VU
**Geckos**		**19**
	Gekkonidae	
	Bunopus tuberculatus	LC
	Cyrtopodion scabrum	LC
	Hemidactylus flaviviridis	LC*
	Hemidactylus hajarensis	NE
	Hemidactylus luqueorum	NE
	Hemidactylus robustus	LC*
	Stenodactylus leptocosymbotes	LC*
	Trachydactylus hajarensis	NE
	Phyllodactylidae	
	Asaccus gallagheri	NE
	Asaccus montanus	VU
	Asaccus platyrhynchus	LC
	Ptyodactylus orlovi	NE
	Sphaerodactylidae	
	Pristurus carteri	LC
	Pristurus celerrimus	LC
	Pristurus gallagheri	NT
	Pristurus minimus	LC
	Pristurus rupestris rupestris	NE
	Pristurus sp. 3	NE
	Pristurus sp. 5	NE
**Lacertids**		**6**
	Lacertidae	
	Acanthodactylus boskianus	LC*
	Acanthodactylus opheodurus	LC
	Acanthodactylus schmidti	LC
	Mesalina adramitana	LC
	Omanosaura cyanura	LC
	Omanosaura jayakari	LC
**Skinks**		**4**
	Scincidae	
	Ablepharus pannonicus	LC*
	Chalcides ocellatus ocellatus	LC*
	Scincus mitranus	LC
	Trachylepis tessellata	LC
**Varanids**		**1**
	Varanidae	
	Varanus griseus	LC*
**Snakes**		**9**
	Boidae	
	Eryx jayakari	LC
	Colubridae	
	Lytorhynchus diadema diadema	LC
	Platyceps rhodorachis rhodorachis	LC*
	Telescopus dhara dhara	LC*
	Lamprophiidae	
	Psammophis schokari	LC*
	Rhagerhis moilensis	LC*
	Viperidae	
	Echis carinatus sochureki	LC*
	Echis omanensis	LC
	Pseudocerastes persicus	LC

* Not available on the web

Venomous species		
**Taxa**	**Family/Specie**	**Total**
**Lizards**		**0**
**Snakes**		**3**
	Viperidae	
	Echis carinatus sochureki	
	Echis omanensis	
	Pseudocerastes persicus	

Endemic species		
**Taxa**	**Family/Specie**	**Total**
**Lizards**		**6**
**Geckos**		**6**
	Gekkonidae	
	Hemidactylus hajarensis	
	Hemidactylus luqueorum	
	Phyllodactylidae	
	Asaccus montanus	
	Asaccus platyrhynchus	
	Sphaerodactylidae	
	Pristurus gallagheri	
	Pristurus sp. 5	
**Snakes**		**0**

Muscat Governorate

S111: Species list by governorate

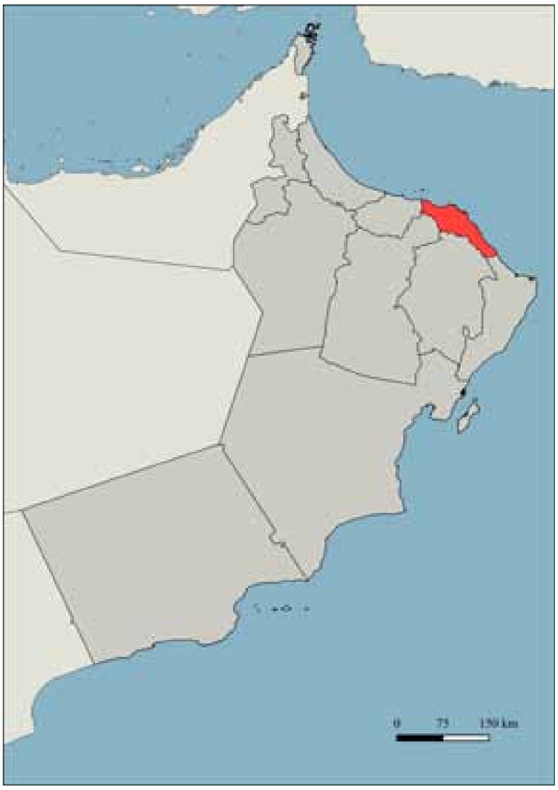

IUCN categories		
**Taxa**	**Family/Specie**	**Total**
**Lizards**		**27**
**Agamids**		**3**
	Agamidae	
	Pseudotrapelus jensvindumi	NE
	Trapelus flavimaculatus	LC
	Uromastyx aegyptia leptieni	VU
**Geckos**		**15**
	Gekkonidae	
	Bunopus tuberculatus	LC
	Cyrtopodion scabrum	LC
	Hemidactylus endophis	NE
	Hemidactylus flaviviridis	LC*
	Hemidactylus hajarensis	NE
	Hemidactylus leschenaultii	LC*
	Hemidactylus robustus	LC*
	Stenodactylus leptocosymbotes	LC*
	Trachydactylus hajarensis	NE
	Phyllodactylidae	
	Asaccus arnoldi	NE
	Ptyodactylus orlovi	NE
	Sphaerodactylidae	
	Pristurus carteri	LC
	Pristurus rupestris rupestris	NE
	Pristurus sp. 2	NE
	Pristurus sp. 5	NE
**Lacertids**		**4**
	Lacertidae	
	Acanthodactylus blanfordii	LC*
	Mesalina adramitana	LC
	Omanosaura cyanura	LC
	Omanosaura jayakari	LC
**Skinks**		**4**
	Scincidae	
	Chalcides ocellatus ocellatus	LC*
	Heremites septemtaeniatus	LC*
	Scincus mitranus	LC
	Trachylepis tessellata	LC
**Varanids**		**1**
	Varanidae	
	Varanus griseus	LC*
**Snakes**		**12**
	Boidae	
	Eryx jayakari	LC
	Colubridae	
	Lytorhynchus diadema diadema	LC
	Platyceps rhodorachis rhodorachis	LC*
	Spalerosophis diadema cliffordii	LC*
	Telescopus dhara dhara	LC*
	Lamprophiidae	
	Psammophis schokari	LC*

Venomous species		
**Taxa**	**Family/Specie**	**Total**
**Lizards**		**0**
**Snakes**		**2**
	Viperidae	
	Echis carinatus sochureki	
	Echis omanensis	

Endemic species		
**Taxa**	**Family/Specie**	**Total**
**Lizards**		**5**
**Geckos**		**5**
	Gekkonidae	
	Hemidactylus endophis	
	Hemidactylus hajarensis	
	Phyllodactylidae	
	Asaccus arnoldi	
	Sphaerodactylidae	
	Pristurus sp. 2	
	Pristurus sp. 5	
**Snakes**		**0**

S112: Species list by governorate

Ash Sharqiyyah North Governorate

IUCN categories		
**Taxa**	**Family/Specie**	**Total**
**Lizards**		**33**
**Agamids**		**4**
	Agamidae	
	Phrynocephalus sakoi	NE
	Pseudotrapelus jensvindumi	NE
	Trapelus flavimaculatus	LC
	Uromastyx aegyptia microlepis	VU
**Geckos**		**18**
	Gekkonidae	
	Bunopus tuberculatus	LC
	Hemidactylus flaviviridis	LC*
	Hemidactylus hajarensis	NE
	Hemidactylus robustus	LC*
	Hemidactylus sp.	NE
	Stenodactylus doriae	LC
	Stenodactylus leptocosymbotes	LC*
	Stenodactylus sharqiyahensis	NE
	Trachydactylus hajarensis	NE
	Phyllodactylidae	
	Asaccus arnoldi	NE
	Ptyodactylus orlovi	NE
	Sphaerodactylidae	
	Pristurus carteri	LC
	Pristurus minimus	LC
	Pristurus rupestris rupestris	NE
	Pristurus sp. 2	NE
	Pristurus sp. 3	NE
	Pristurus sp. 4	NE
	Pristurus sp. 5	NE
**Lacertids**		**7**
	Lacertidae	
	Acanthodactylus boskianus	LC*
	Acanthodactylus haasi	LC
	Acanthodactylus opheodurus	LC
	Acanthodactylus schmidti	LC
	Mesalina adramitana	LC
	Omanosaura cyanura	LC
	Omanosaura jayakari	LC
**Skinks**		**2**
	Scincidae	
	Scincus mitranus	LC
	Trachylepis tessellata	LC
**Amphisbaenids**		**1**
	Trogonophidae	
	Diplometopon zarudnyi	LC
**Varanids**		**1**
	Varanidae	
	Varanus griseus	LC*
**Snakes**		**9**
	Boidae	
	Eryx jayakari	LC
	Colubridae	
	Platyceps rhodorachis rhodorachis	LC*
	Telescopus dhara dhara	LC*
	Lamprophiidae	
	Psammophis schokari	LC*
	Rhagerhis moilensis	LC*
	Leptotyphlopidae	
	Myriopholis macrorhyncha	LC*
	Viperidae	
	Cerastes gasperettii gasperettii	LC
	Echis omanensis	LC
	Pseudocerastes persicus	LC

* Not available on the web

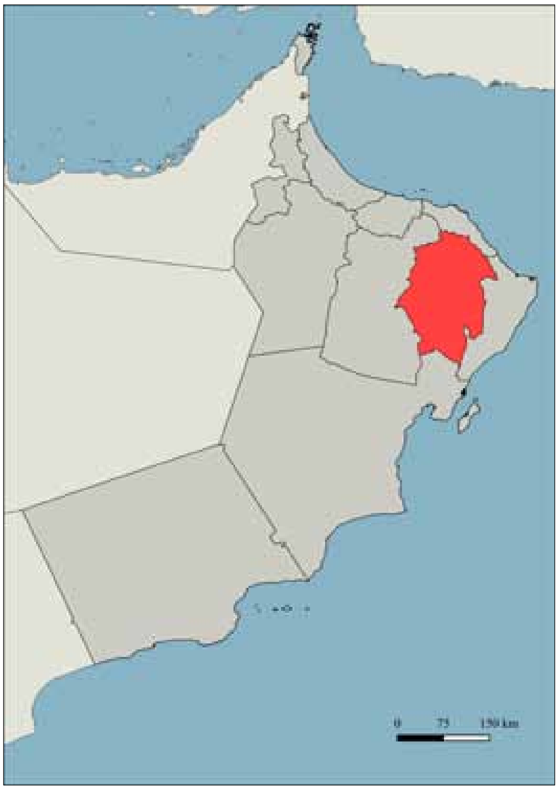

Venomous species		
**Taxa**	**Family/Specie**	**Total**
**Lizards**		**0**
**Snakes**		**3**
	Viperidae	
	Cerastes gasperettii gasperettii	
	Echis omanensis	
	Pseudocerastes persicus	

Endemic species		
**Taxa**	**Family/Specie**	**Total**
**Lizards**		**8**
**Agamids**		**1**
	Agamidae	
	Phrynocephalus sakoi	
**Geckos**		**7**
	Gekkonidae	
	Hemidactylus hajarensis	
	Hemidactylus sp.	
	Stenodactylus sharqiyahensis	
	Phyllodactylidae	
	Asaccus arnoldi	
	Sphaerodactylidae	
	Pristurus sp. 2	
	Pristurus sp. 4	
	Pristurus sp. 5	
**Snakes**		**0**

Ash Sharqiyyah South Governorate

S113: Species list by governorate

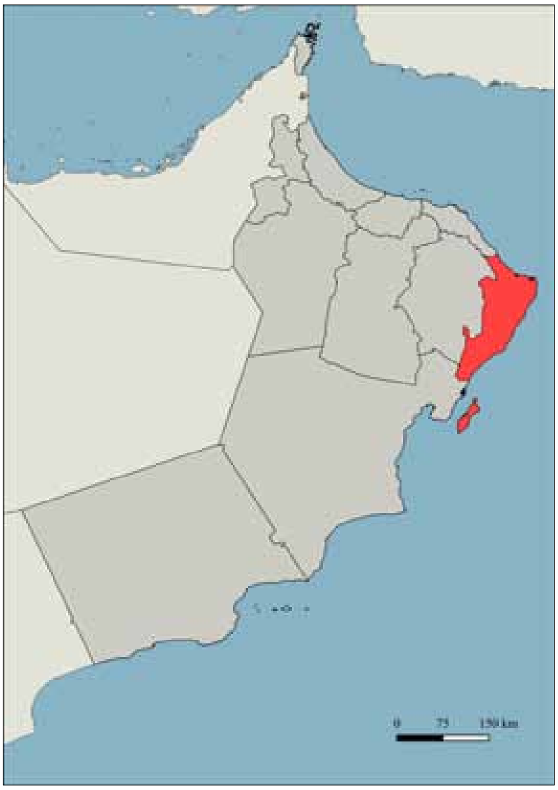

IUCN categories		
**Taxa**	**Family/Specie**	**Total**
**Lizards**		**37**
**Agamids**		**5**
	Agamidae	
	Phrynocephalus sakoi	NE
	Pseudotrapelus dhofarensis	NE
	Pseudotrapelus jensvindumi	NE
	Uromastyx thomasi	VU
	Chamaeleonidae	
	Chamaeleo arabicus	LC
**Geckos**		**19**
	Gekkonidae	
	Bunopus tuberculatus	LC
	Hemidactylus flaviviridis	LC*
	Hemidactylus hajarensis	NE
	Hemidactylus masirahensis	NE
	Hemidactylus minutus	NE
	Hemidactylus robustus	LC*
	Pseudoceramodactylus khobarensis	LC
	Stenodactylus doriae	LC
	Stenodactylus leptocosymbotes	LC*
	Stenodactylus sharqiyahensis	NE
	Trachydactylus hajarensis	NE
	Phyllodactylidae	
	Asaccus arnoldi	NE
	Ptyodactylus orlovi	NE
	Sphaerodactylidae	
	Pristurus carteri	LC
	Pristurus minimus	LC
	Pristurus rupestris rupestris	NE
	Pristurus sp. 2	NE
	Pristurus sp. 3	NE
	Pristurus sp. 5	NE
**Lacertids**		**8**
	Lacertidae	
	Acanthodactylus boskianus	LC*
	Acanthodactylus haasi	LC
	Acanthodactylus masirae	DD
	Acanthodactylus opheodurus	LC
	Acanthodactylus schmidti	LC
	Mesalina adramitana	LC
	Omanosaura cyanura	LC
	Omanosaura jayakari	LC
**Skinks**		**3**
	Scincidae	
	Chalcides ocellatus ocellatus	LC*
	Scincus mitranus	LC
	Trachylepis tessellata	LC
**Amphisbaenids**		**1**
	Trogonophidae	
	Diplometopon zarudnyi	LC
**Varanids**		**1**
	Varanidae	
	Varanus griseus	LC*
**Snakes**		**11**
	Boidae	
	Eryx jayakari	LC
	Colubridae	
	Lytorhynchus diadema diadema	LC
	Platyceps rhodorachis rhodorachis	LC*
	Spalerosophis diadema cliffordii	LC*
	Telescopus dhara dhara	LC*
	Lamprophiidae	
	Psammophis schokari	LC*
	Rhagerhis moilensis	LC*
	Leptotyphlopidae	
	Myriopholis macrorhyncha	LC*
	Viperidae	
	Cerastes gasperettii gasperettii	LC
	Echis carinatus sochureki	LC*
	Echis omanensis	LC

* Not available on the web

Venomous species		
**Taxa**	**Family/Specie**	**Total**
**Lizards**		**0**
**Snakes**		**3**
	Viperidae	
	Cerastes gasperettii gasperettii	
	Echis carinatus sochureki	
	Echis omanensis	

S114: Species list by governorate

Ash Sharqiyyah South Governorate

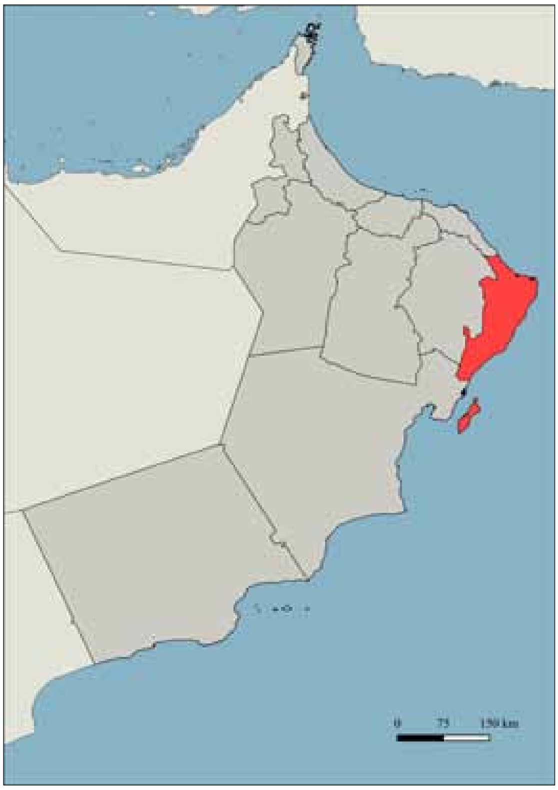

Endemic species		
**Taxa**	**Family/Specie**	**Total**
**Lizards**		**9**
**Agamids**		**2**
	Agamidae	
	Phrynocephalus sakoi	
	Uromastyx thomasi	
**Geckos**		**6**
	Gekkonidae	
	Hemidactylus hajarensis	
	Hemidactylus masirahensis	
	Stenodactylus sharqiyahensis	
	Phyllodactylidae	
	Asaccus arnoldi	
	Sphaerodactylidae	
	Pristurus sp. 2	
	Pristurus sp. 5	
**Lacertids**		**1**
	Lacertidae	
	Acanthodactylus masirae	
**Snakes**		**0**

Species of Masirah Islands		
**Taxa**	**Family/Specie**	**Total**
**Lizards**		**13**
**Agamids**		**3**
	Agamidae	
	Pseudotrapelus dhofarensis	
	Uromastyx thomasi	
	Chamaeleonidae	
	Chamaeleo arabicus	
**Geckos**		**6**
	Gekkonidae	
	Hemidactylus masirahensis	
	Hemidactylus robustus	
	Stenodactylus leptocosymbotes	
	Trachydactylus hajarensis	
	Sphaerodactylidae	
	Pristurus carteri	
	Pristurus minimus	
**Lacertids**		**2**
	Lacertidae	
	Acanthodactylus masirae	
	Mesalina adramitana	
**Skinks**		**2**
	Scincidae	
	Scincus mitranus	
	Trachylepis tessellata	
**Snakes**		**6**
	Colubridae	
	Platyceps rhodorachis rhodorachis	
	Spalerosophis diadema cliffordii	
	Telescopus dhara dhara	
	Lamprophiidae	
	Psammophis schokari	
	Rhagerhis moilensis	
	Viperidae	
	Echis carinatus sochureki	

Al Wusta Governorate

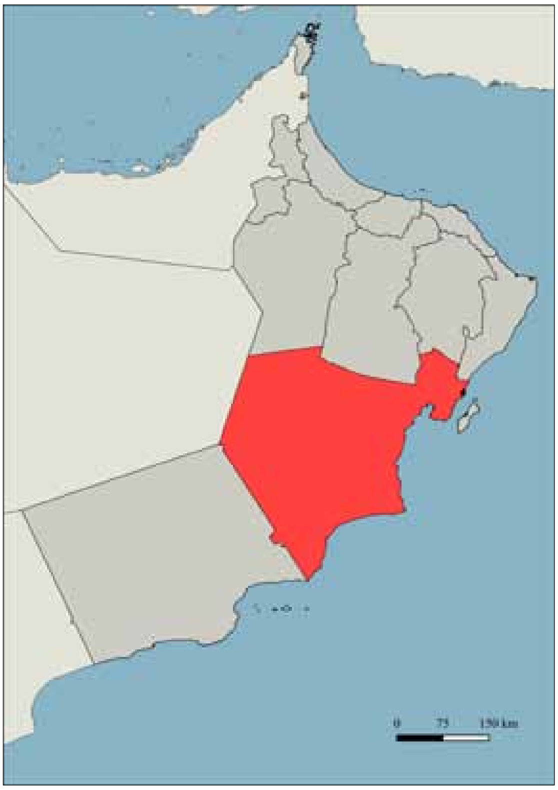

Venomous species		
**Taxa**	**Family/Specie**	**Total**
**Lizards**		**0**
**Snakes**		**4**
	Viperidae	
	Cerastes gasperettii gasperettii	
	Echis carinatus sochureki	
	Echis coloratus	
	Echis khosatzkii	

Endemic species		
**Taxa**	**Family/Specie**	**Total**
**Lizards**		**5**
**Agamids**		**2**
	Agamidae	
	Phrynocephalus sakoi	
	Uromastyx thomasi	
**Geckos**		**2**
	Gekkonidae	
	Hemidactylus inexpectatus	
	Stenodactylus sharqiyahensis	
**Lacertids**		**1**
	Lacertidae	
	Acanthodactylus masirae	
**Snakes**		**0**

Species of Al Wusta Islands		
**Taxa**	**Family/Specie**	**Total**
**Lizards**		**4**
**Geckos**		**3**
	Gekkonidae	
	Hemidactylus inexpectatus	
	Sphaerodactylidae	
	Pristurus carteri	
	Pristurus sp. 1	
**Lacertids**		**1**
	Lacertidae	
	Acanthodactylus masirae	
**Snakes**		**0**

S115: Species list by governorate

IUCN categories		
**Taxa**	**Family/Specie**	**Total**
**Lizards**		**31**
**Agamids**		**7**
	Agamidae	
	Phrynocephalus arabicus	LC
	Phrynocephalus maculatus	LC*
	Phrynocephalus sakoi	NE
	Pseudotrapelus dhofarensis	NE
	Trapelus flavimaculatus	LC
	Uromastyx aegyptia microlepis	VU
	Uromastyx thomasi	VU
**Geckos**		**15**
	Gekkonidae	
	Bunopus tuberculatus	LC
	Hemidactylus festivus	NE
	Hemidactylus inexpectatus	NE
	Hemidactylus minutus	NE
	Hemidactylus robustus	LC*
	Pseudoceramodactylus khobarensis	LC
	Stenodactylus arabicus	LC
	Stenodactylus doriae	LC
	Stenodactylus leptocosymbotes	LC*
	Stenodactylus sharqiyahensis	NE
	Trachydactylus hajarensis	NE
	Tropiocolotes scortecci	LC
	Sphaerodactylidae	
	Pristurus carteri	LC
	Pristurus minimus	LC
	Pristurus sp. 1	NE
**Lacertids**		**6**
	Lacertidae	
	Acanthodactylus boskianus	LC*
	Acanthodactylus haasi	LC
	Acanthodactylus masirae	DD
	Acanthodactylus opheodurus	LC
	Acanthodactylus schmidti	LC
	Mesalina adramitana	LC
**Skinks**		**1**
	Scincidae	
	Scincus mitranus	LC
**Amphisbaenids**		**1**
	Trogonophidae	
	Diplometopon zarudnyi	LC
**Varanids**		**1**
	Varanidae	
	Varanus griseus	LC*
**Snakes**		**12**
	Boidae	
	Eryx jayakari	LC
	Colubridae	
	Lytorhynchus diadema diadema	LC
	Platyceps rhodorachis rhodorachis	LC*
	Spalerosophis diadema cliffordii	LC*
	Telescopus dhara dhara	LC*
	Lamprophiidae	
	Psammophis schokari	LC*
	Rhagerhis moilensis	LC*
	Leptotyphlopidae	
	Myriopholis macrorhyncha	LC*
	Viperidae	
	Cerastes gasperettii gasperettii	LC
	Echis carinatus sochureki	LC*
	Echis coloratus	LC*
	Echis khosatzkii	LC

* Not available on the web

S116: Species list by governorate

Dhofar Governorate

IUCN categories		
**Taxa**	**Family/Specie**	**Total**
**Lizards**		**43**
**Agamids**		**10**
	Agamidae	
	Acanthocercus adramitanus	LC
	Calotes versicolor	LC*
	Phrynocephalus arabicus	LC
	Phrynocephalus maculatus	LC*
	Pseudotrapelus dhofarensis	NE
	Trapelus flavimaculatus	LC
	Uromastyx aegyptia microlepis	VU
	Uromastyx benti	LC
	Uromastyx thomasi	VU
	Chamaeleonidae	
	Chamaeleo arabicus	LC
**Geckos**		**19**
	Gekkonidae	
	Bunopus tuberculatus	LC
	Cyrtopodion scabrum	LC
	Hemidactylus alkiyumii	NE
	Hemidactylus festivus	NE
	Hemidactylus flaviviridis	LC*
	Hemidactylus lemurinus	DD
	Hemidactylus minutus	NE
	Hemidactylus paucituberculatus	NE
	Hemidactylus robustus	LC*
	Stenodactylus arabicus	LC
	Stenodactylus doriae	LC
	Stenodactylus leptocosymbotes	LC*
	Trachydactylus spatalurus	NE
	Tropiocolotes scortecci	LC
	Tropiocolotes sp.	NE
	Phyllodactylidae	
	Ptyodactylus dhofarensis	NE
	Sphaerodactylidae	
	Pristurus carteri	LC
	Pristurus minimus	LC
	Pristurus sp. 1	NE
**Lacertids**		**9**
	Lacertidae	
	Acanthodactylus boskianus	LC*
	Acanthodactylus felicis	VU
	Acanthodactylus masirae	DD
	Acanthodactylus opheodurus	LC
	Acanthodactylus schmidti	LC
	Mesalina adramitana	LC
	Mesalina ayunensis	DD
	Mesalina sp. 1	NE
	Mesalina sp. 2	NE
**Skinks**		**4**
	Scincidae	
	Chalcides ocellatus ocellatus	LC*
	Scincus mitranus	LC
	Trachylepis brevicollis	LC*
	Trachylepis tessellata	LC
**Varanids**		**1**
	Varanidae	
	Varanus griseus	LC*
**Snakes**		**17**
	Boidae	
	Eryx jayakari	LC
	Colubridae	
	Lytorhynchus diadema diadema	LC
	Platyceps rhodorachis rhodorachis	LC*
	Platyceps thomasi	DD
	Rhynchocalamus arabicus	DD
	Spalerosophis diadema cliffordii	LC*
	Telescopus dhara dhara	LC*
	Elapidae	
	Naja arabica	LC
	Lamprophiidae	
	Atractaspis andersonii	LC
	Psammophis schokari	LC*
	Rhagerhis moilensis	LC*
	Leptotyphlopidae	
	Myriopholis macrorhyncha	LC*
	Myriopholis nursii	LC*
	Viperidae	
	Bitis arietans	LC*
	Cerastes gasperettii gasperettii	LC
	Echis coloratus	LC*
	Echis khosatzkii	LC

* Not available on the web

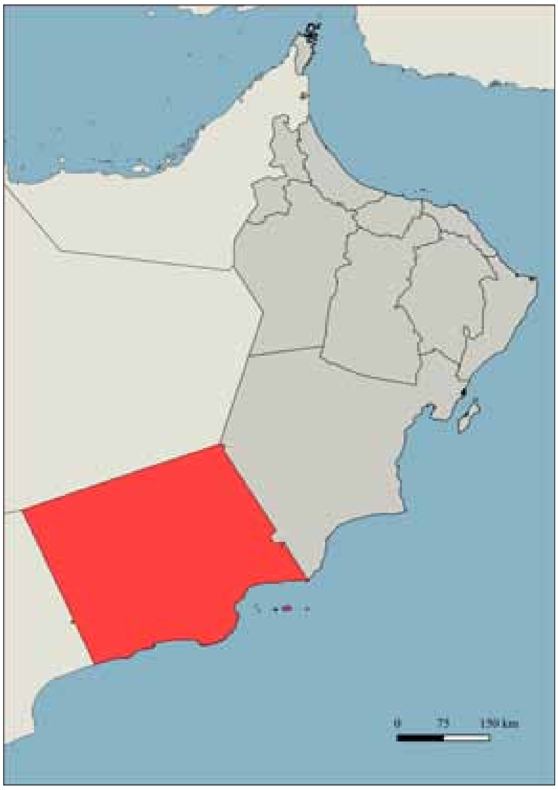

Venomous species		
**Taxa**	**Family/Specie**	**Total**
**Lizards**		**0**
**Snakes**		**6**
	Elapidae	
	Naja arabica	
	Lamprophiidae	
	Atractaspis andersonii	
	Viperidae	
	Bitis arietans	
	Cerastes gasperettii gasperettii	
	Echis coloratus	
	Echis khosatzkii	

Endemic species		
**Taxa**	**Family/Specie**	**Total**
**Lizards**		**5**
**Agamids**		**1**
	Agamidae	
	Uromastyx thomasi	
**Geckos**		**2**
	Gekkonidae	
	Hemidactylus paucituberculatus	
	Tropiocolotes sp.	
**Lacertids**		**2**
	Lacertidae	
	Acanthodactylus masirae	
	Mesalina sp. 2	
**Snakes**		**0**

Species of Hallaniyyat Islands		
**Taxa**	**Family/Specie**	**Total**
**Lizards**		**6**
**Geckos**		**6**
	Gekkonidae	
	Hemidactylus alkiyumii	
	Hemidactylus paucituberculatus	
	Hemidactylus robustus	
	Tropiocolotes scortecci	
	Sphaerodactylidae	
	Pristurus carteri	
	Pristurus sp. 1	
**Snakes**		**0**

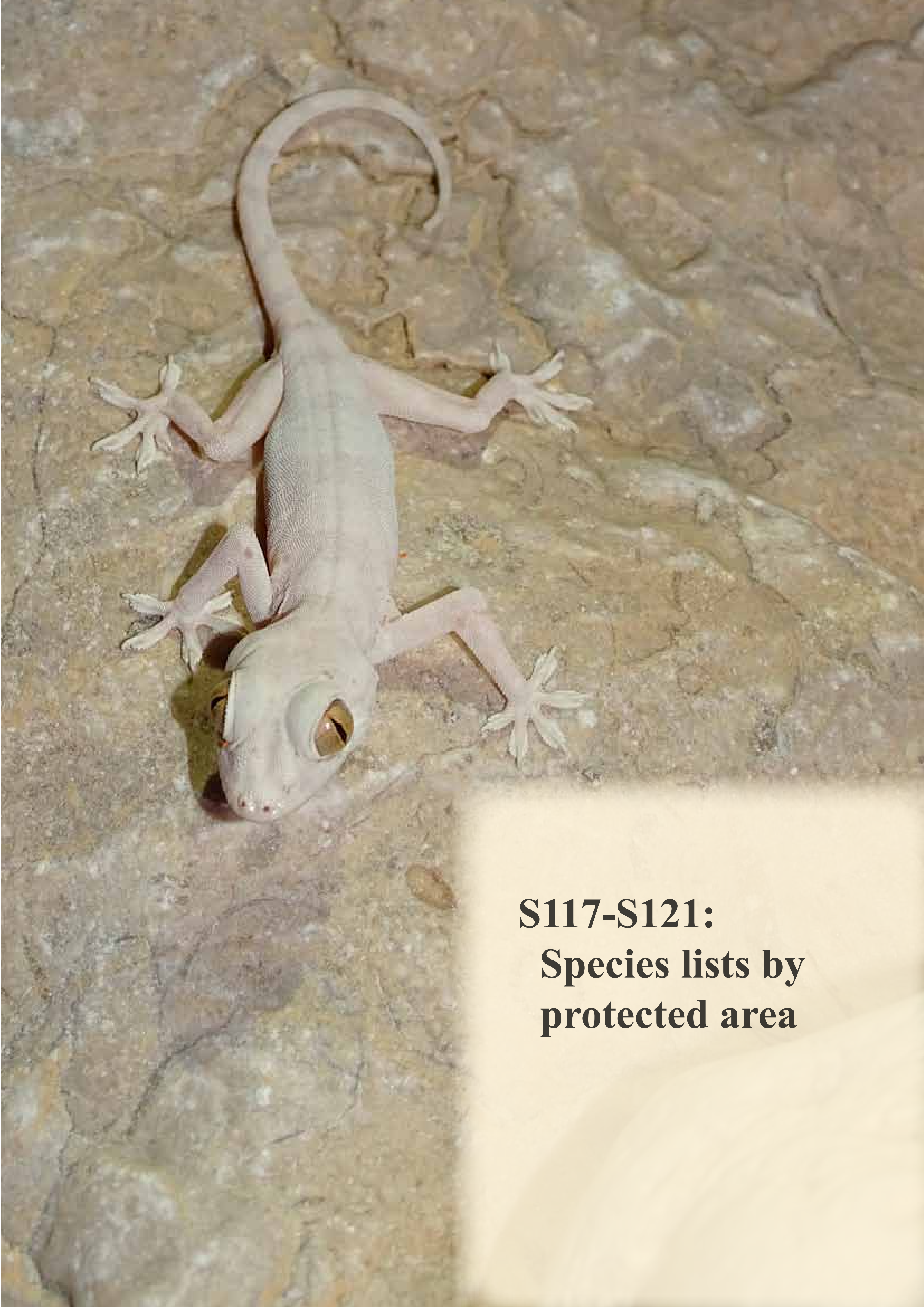

S117-S121: Species lists by protected area

S117-S121: Species lists by protected area

(S117) Maps of Oman showing the protected areas and a complete list of all the species of terrestrial reptiles within protected areas indicating if the species are endemic, venomous and with their IUCN conservation category. **(S118-S121)** Map of Oman showing the protected areas and with independent species lists for each one of the 22 protected areas indicating if the species are endemic, venomous and with their IUCN conservation category.

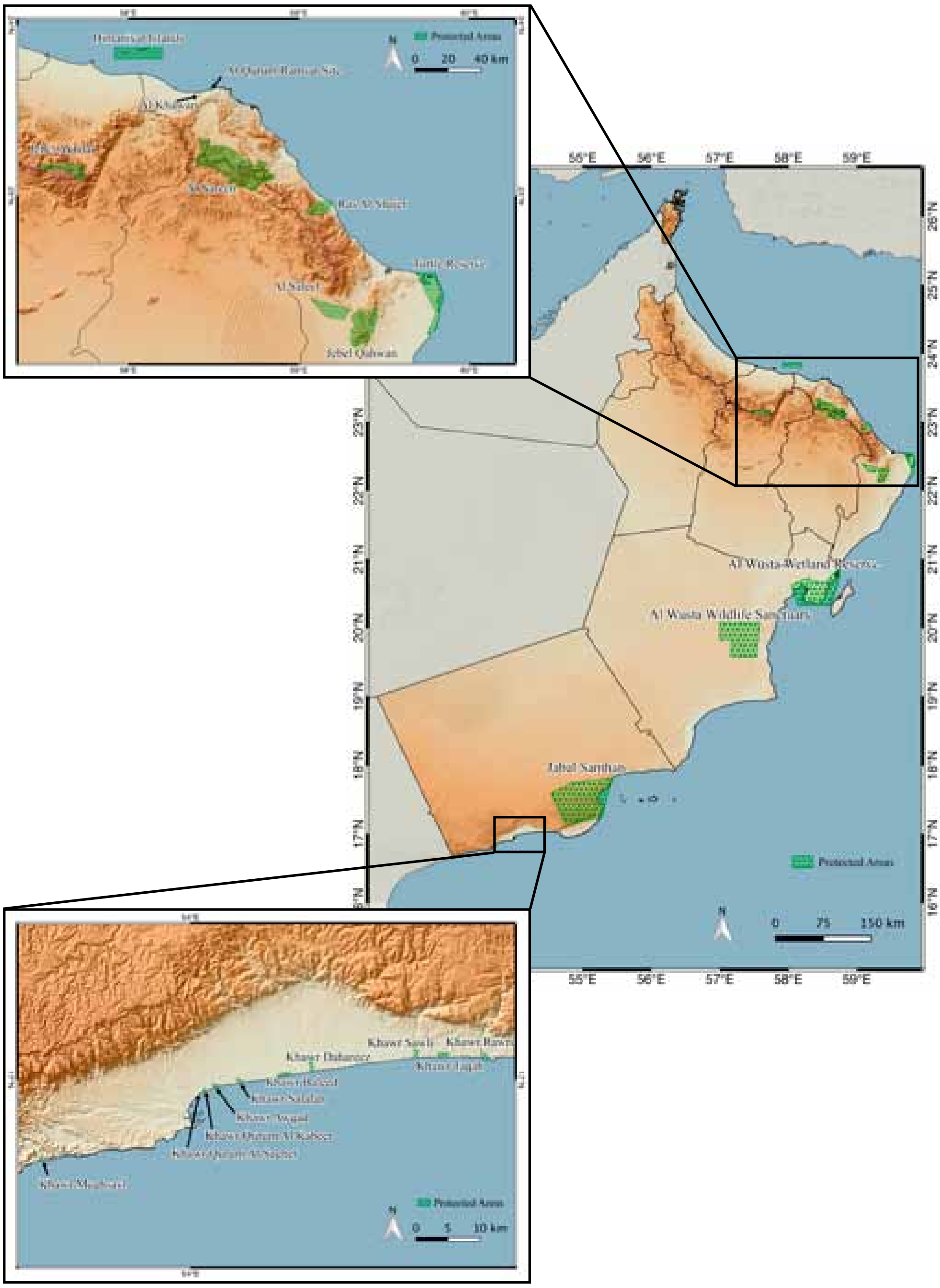

Topographical map of Oman showing the distribution, limits and names of the 22 protected areas.

S117: Species list by protected areas

Protected areas of Oman

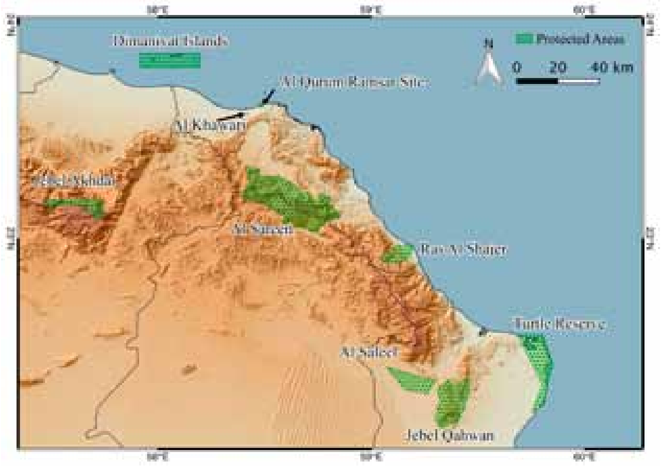

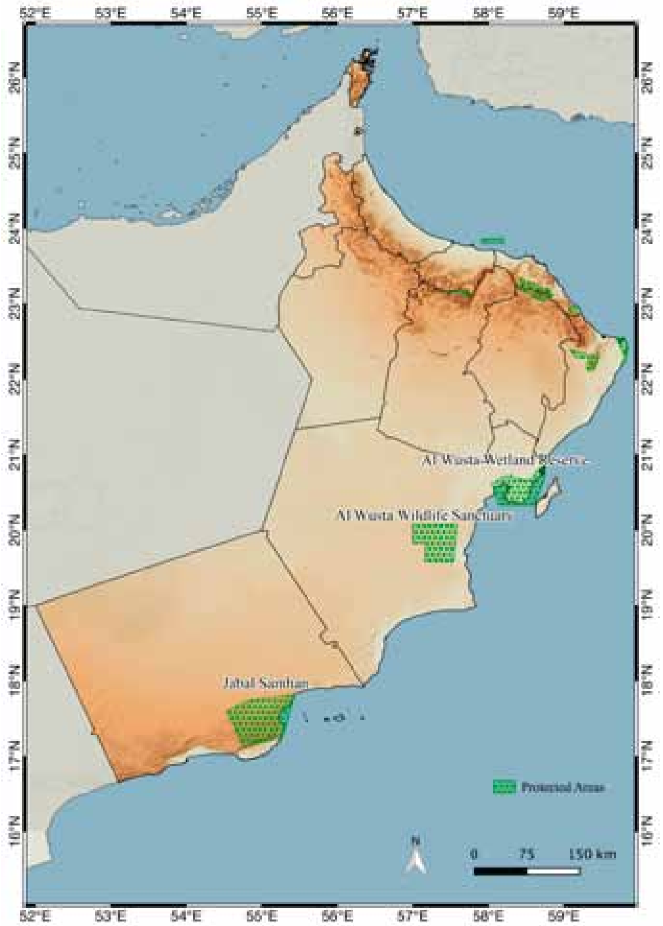

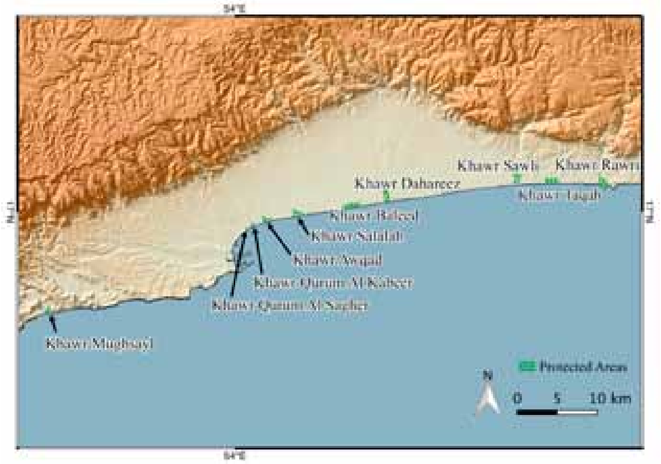

Species in protected areas				
**Taxa**	**Family/Specie**	**Endemics**	**Venomous**	**Total**
**Lizards**		**10**	**0**	**47**
**Agamids**		**2**	**0**	**8**
	Agamidae			
	Phrynocephalus maculatus	NO	NO	LC*
	Phrynocephalus sakoi	YES	NO	NE
	Pseudotrapelus dhofarensis	NO	NO	NE
	Pseudotrapelus jensvindumi	NO	NO	NE
	Trapelus flavimaculatus	NO	NO	LC
	Uromastyx aegyptia microlepis	NO	NO	VU
	Uromastyx thomasi	YES	NO	VU
	Chamaeleonidae			
	Chamaeleo arabicus	NO	NO	LC
**Geckos**		**7**	**0**	**25**
	Gekkonidae			
	Bunopus tuberculatus	NO	NO	LC
	Hemidactylus alkiyumii	NO	NO	NE
	Hemidactylus flaviviridis	NO	NO	LC*
	Hemidactylus hajarensis	YES	NO	NE
	Hemidactylus minutus	NO	NO	NE
	Hemidactylus paucituberculatus	YES	NO	NE
	Hemidactylus robustus	NO	NO	LC*
	Pseudoceramodactylus khobarensis	NO	NO	LC
	Stenodactylus doriae	NO	NO	LC
	Stenodactylus leptocosymbotes	NO	NO	LC*
	Stenodactylus sharqiyahensis	YES	NO	NE
	Trachydactylus hajarensis	NO	NO	NE
	Trachydactylus spatalurus	NO	NO	NE
	Tropiocolotes scortecci	NO	NO	LC
	Phyllodactylidae			
	Asaccus arnoldi	YES	NO	NE
	Ptyodactylus dhofarensis	NO	NO	NE
	Ptyodactylus orlovi	NO	NO	NE
	Sphaerodactylidae			
	Pristurus carteri	NO	NO	LC
	Pristurus celerrimus	NO	NO	LC
	Pristurus gallagheri	YES	NO	NT
	Pristurus minimus	NO	NO	LC
	Pristurus rupestris rupestris	NO	NO	NE
	Pristurus sp. 1	NO	NO	NE
	Pristurus sp. 4	YES	NO	NE
	Pristurus sp. 5	YES	NO	NE
**Lacertids**		**1**	**0**	**8**
	Lacertidae			
	Acanthodactylus blanfordii	NO	NO	LC*
	Acanthodactylus felicis	NO	NO	VU
	Acanthodactylus masirae	YES	NO	DD
	Acanthodactylus schmidti	NO	NO	LC
	Mesalina adramitana	NO	NO	LC
	Mesalina sp. 1	NO	NO	NE
	Omanosaura cyanura	NO	NO	LC
	Omanosaura jayakari	NO	NO	LC
**Skinks**		**0**	**0**	**4**
	Scincidae			
	Chalcides ocellatus ocellatus	NO	NO	LC*
	Heremites septemtaeniatus	NO	NO	LC*
	Scincus mitranus	NO	NO	LC
	Trachylepis tessellata	NO	NO	LC
**Amphisbaenids**		**0**	**0**	**1**
	Trogonophidae			
	Diplometopon zarudnyi	NO	NO	LC
**Varanids**		**0**	**0**	**1**
	Varanidae			
	Varanus griseus	NO	NO	LC*
**Snakes**		**0**	**7**	**17**
	Boidae			
	Eryx jayakari	NO	NO	LC
	Colubridae			
	Lytorhynchus diadema diadema	NO	NO	LC
	Platyceps rhodorachis rhodorachis	NO	NO	LC*
	Platyceps thomasi	NO	NO	DD
	Spalerosophis diadema cliffordii	NO	NO	LC*
	Telescopus dhara dhara	NO	NO	LC*
	Elapidae			
	Naja arabica	NO	YES	LC
	Lamprophiidae			
	Psammophis schokari	NO	NO	LC*
	Rhagerhis moilensis	NO	NO	LC*
	Leptotyphlopidae			
	Myriopholis macrorhyncha	NO	NO	LC*
	Myriopholis nursii	NO	NO	LC*
	Viperidae			
	Cerastes gasperettii gasperettii	NO	YES	LC
	Echis carinatus sochureki	NO	YES	LC*
	Echis coloratus	NO	YES	LC*
	Echis khosatzkii	NO	YES	LC
	Echis omanensis	NO	YES	LC
	Pseudocerastes persicus	NO	YES	LC

* Not available on the web

Dimaniyat Islands, Al Qurum Ramsar Site, Al Khawari, Jebel Akhdar, Al Sareen & Ras Al Shajer

S118: Species list by protected areas

Dimaniyat Islands				
**Taxa**	**Family/Specie**	**Endemics**	**Venomous**	**Total**
**Lizards**		**0**	**0**	**3**
**Geckos**		**0**	**0**	**2**
	Gekkonidae			
	Hemidactylus robustus	NO	NO	LC*
	Sphaerodactylidae			
	Pristurus rupestris rupestris	NO	NO	NE
**Skinks**		**0**	**0**	**1**
	Scincidae			
	Heremites septemtaeniatus	NO	NO	LC*
**Snakes**		**0**	**0**	**1**
	Colubridae			
	Platyceps rhodorachis rhodorachis	NO	NO	LC*

* Not available on the web

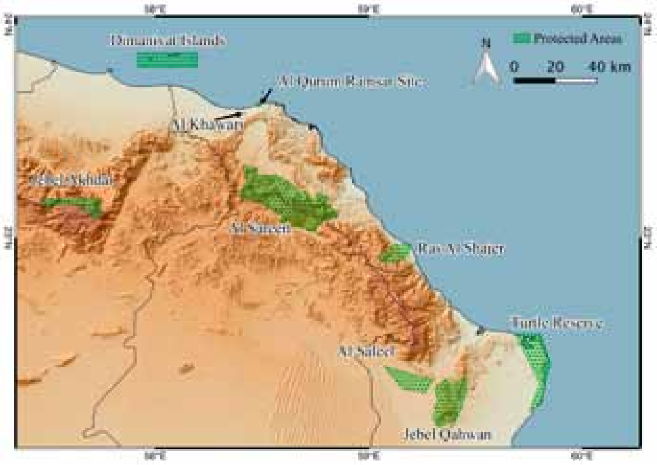

Al Qurum Ramsar Site				
**Taxa**	**Family/Specie**	**Endemics**	**Venomous**	**Total**
**Lizards**		**0**	**0**	**3**
**Geckos**		**0**	**0**	**1**
	Gekkonidae			
	Bunopus tuberculatus	NO	NO	LC
**Lacertids**		**0**	**0**	**1**
	Lacertidae			
	Acanthodactylus blanfordii	NO	NO	LC*
**Skinks**		**0**	**0**	**1**
	Scincidae			
	Scincus mitranus	NO	NO	LC
**Snakes**		**0**	**0**	**2**
	Colubridae			
	Platyceps rhodorachis rhodorachis	NO	NO	LC*
	Spalerosophis diadema cliffordii	NO	NO	LC*

* Not available on the web

Al Khawari				
**Taxa**	**Family/Specie**	**Endemics**	**Venomous**	**Total**
**Lizards**		**0**	**0**	**1**
**Lacertids**		**0**	**0**	**1**
	Lacertidae			
	Acanthodactylus blanfordii	NO	NO	LC*
**Snakes**		**0**	**0**	**0**

* Not available on the web

Jebel Akhdar				
**Taxa**	**Family/Specie**	**Endemics**	**Venomous**	**Total**
**Lizards**		**1**	**0**	**5**
**Geckos**		**1**	**0**	**4**
	Phyllodactylidae			
	Ptyodactylus orlovi	NO	NO	NE
	Sphaerodactylidae			
	Pristurus celerrimus	NO	NO	LC
	Pristurus gallagheri	YES	NO	NT
	Pristurus rupestris rupestris	NO	NO	NE
**Lacertids**		**0**	**0**	**1**
	Lacertidae			
	Omanosaura jayakari	NO	NO	LC
**Snakes**		**0**	**1**	**2**
	Colubridae			
	Platyceps rhodorachis rhodorachis	NO	NO	LC*
	Viperidae			
	Pseudocerastes persicus	NO	YES	LC

* Not available on the web

Al Sareen				
**Taxa**	**Family/Specie**	**Endemics**	**Venomous**	**Total**
**Lizards**		**3**	**0**	**9**
**Agamids**		**0**	**0**	**1**
	Agamidae			
	Pseudotrapelus jensvindumi	NO	NO	NE
**Geckos**		**3**	**0**	**6**
	Gekkonidae			
	Hemidactylus hajarensis	YES	NO	NE
	Trachydactylus hajarensis	NO	NO	NE
	Phyllodactylidae			
	Asaccus arnoldi	YES	NO	NE
	Ptyodactylus orlovi	NO	NO	NE
	Sphaerodactylidae			
	Pristurus rupestris rupestris	NO	NO	NE
	Pristurus sp. 4	YES	NO	NE
**Lacertids**		**0**	**0**	**2**
	Lacertidae			
	Omanosaura cyanura	NO	NO	LC
	Omanosaura jayakari	NO	NO	LC
**Snakes**		**0**	**2**	**3**
	Colubridae			
	Platyceps rhodorachis rhodorachis	NO	NO	LC*
	Viperidae			
	Echis omanensis	NO	YES	LC
	Pseudocerastes persicus	NO	YES	LC

* Not available on the web

Ras Al Shajer				
**Taxa**	**Family/Specie**	**Endemics**	**Venomous**	**Total**
**Lizards**		**0**	**0**	**3**
**Geckos**		**0**	**0**	**2**
	Phyllodactylidae			
	Ptyodactylus orlovi	NO	NO	NE
	Sphaerodactylidae			
	Pristurus rupestris rupestris	NO	NO	NE
**Lacertids**		**0**	**0**	**1**
	Lacertidae			
	Omanosaura jayakari	NO	NO	LC
**Snakes**		**0**	**0**	**0**

* Not available on the web

S119: Species list by protected areas

Al Saleel, Turtle Reserve & Jebel Qahwan

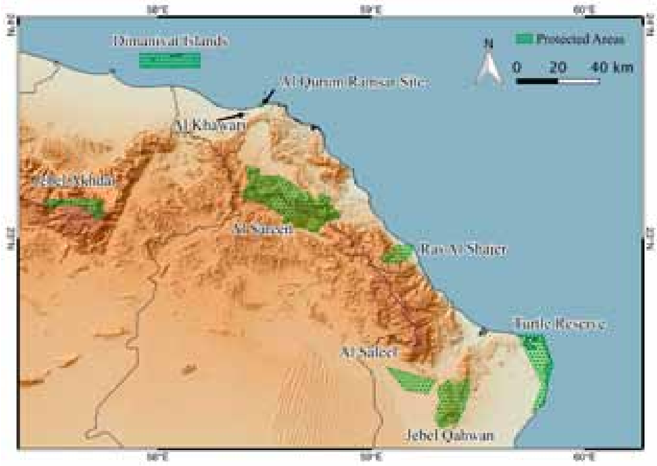

Turtle Reserve				
**Taxa**	**Family/Specie**	**Endemics**	**Venomous**	**Total**
**Lizards**		**2**	**0**	**12**
**Geckos**		**2**	**0**	**8**
	Gekkonidae			
	Hemidactylus flaviviridis	NO	NO	LC*
	Hemidactylus hajarensis	YES	NO	NE
	Hemidactylus minutus	NO	NO	NE
	Stenodactylus leptocosymbotes	NO	NO	LC*
	Trachydactylus hajarensis	NO	NO	NE
	Sphaerodactylidae			
	Pristurus carteri	NO	NO	LC
	Pristurus minimus	NO	NO	LC
	Pristurus sp. 5	YES	NO	NE
**Lacertids**		**0**	**0**	**2**
	Lacertidae			
	Acanthodactylus schmidti	NO	NO	LC
	Mesalina adramitana	NO	NO	LC
**Skinks**		**0**	**0**	**1**
	Scincidae			
	Chalcides ocellatus ocellatus	NO	NO	LC*
**Amphisbaenids**		**0**	**0**	**1**
	Trogonophidae			
	Diplometopon zarudnyi	NO	NO	LC
**Snakes**		**0**	**2**	**5**
	Colubridae			
	Telescopus dhara dhara	NO	NO	LC*
	Lamprophiidae			
	Psammophis schokari	NO	NO	LC*
	Leptotyphlopidae			
	Myriopholis macrorhyncha	NO	NO	LC*
	Viperidae			
	Cerastes gasperettii gasperettii	NO	YES	LC
	Echis carinatus sochureki	NO	YES	LC*

* Not available on the web

Al Saleel				
**Taxa**	**Family/Specie**	**Endemics**	**Venomous**	**Total**
**Lizards**		**0**	**0**	**6**
**Geckos**		**0**	**0**	**4**
	Gekkonidae			
	Bunopus tuberculatus	NO	NO	LC
	Hemidactylus robustus	NO	NO	LC*
	Stenodactylus leptocosymbotes	NO	NO	LC*
	Sphaerodactylidae			
	Pristurus minimus	NO	NO	LC
**Lacertids**		**0**	**0**	**1**
	Lacertidae			
	Mesalina adramitana	NO	NO	LC
**Skinks**		**0**	**0**	**1**
	Scincidae			
	Scincus mitranus	NO	NO	LC
**Snakes**		**0**	**0**	**2**
	Colubridae			
	Lytorhynchus diadema diadema	NO	NO	LC
	Telescopus dhara dhara	NO	NO	LC*

* Not available on the web

Jebel Qahwan				
**Taxa**	**Family/Specie**	**Endemics**	**Venomous**	**Total**
**Lizards**		**2**	**0**	**4**
**Agamids**		**0**	**0**	**1**
	Agamidae			
	Pseudotrapelus jensvindumi	NO	NO	NE
**Geckos**		**2**	**0**	**3**
	Gekkonidae			
	Hemidactylus hajarensis	YES	NO	NE
	Phyllodactylidae			
	Asaccus sp.	YES	NO	NE
	Ptyodactylus orlovi	NO	NO	NE
**Snakes**		**0**	**0**	**0**

* Not available on the web

Al Wusta Wetland Reserve, Al Wusta Wildlife Sanctuary & Jabal Samhan

S120: Species list by protected areas

Al Wusta Wetland Reserve				
**Taxa**	**Family/Specie**	**Endemics**	**Venomous**	**Total**
**Lizards**		**3**	**0**	**13**
**Agamids**		**1**	**0**	**2**
	Agamidae			
	Phrynocephalus maculatus	NO	NO	LC*
	Phrynocephalus sakoi	YES	NO	NE
**Geckos**		**1**	**0**	**8**
	Gekkonidae			
	Hemidactylus robustus	NO	NO	LC*
	Pseudoceramodactylus khobarensis	NO	NO	LC
	Stenodactylus doriae	NO	NO	LC
	Stenodactylus leptocosymbotes	NO	NO	LC*
	Stenodactylus sharqiyahensis	YES	NO	NE
	Sphaerodactylidae			
	Pristurus carteri	NO	NO	LC
	Pristurus minimus	NO	NO	LC
	Pristurus sp. 1	NO	NO	NE
**Lacertids**		**1**	**0**	**3**
	Lacertidae			
	Acanthodactylus masirae	YES	NO	DD
	Acanthodactylus schmidti	NO	NO	LC
	Mesalina adramitana	NO	NO	LC
**Snakes**		**0**	**0**	**0**

* Not available on the web

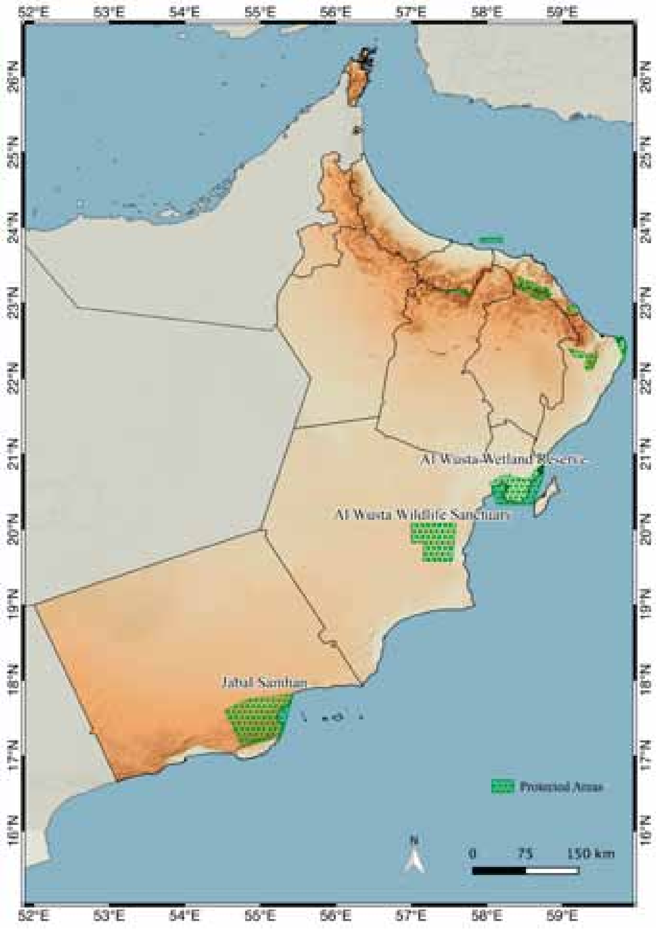

Al Wusta Wildlife Sanctuary				
**Taxa**	**Family/Specie**	**Endemics**	**Venomous**	**Total**
**Lizards**		**1**	**0**	**14**
**Agamids**		**1**	**0**	**3**
	Agamidae			
	Trapelus flavimaculatus	NO	NO	LC
	Uromastyx aegyptia microlepis	NO	NO	VU
	Uromastyx thomasi	YES	NO	VU
**Geckos**		**0**	**0**	**7**
	Gekkonidae			
	Bunopus tuberculatus	NO	NO	LC
	Hemidactylus robustus	NO	NO	LC*
	Stenodactylus leptocosymbotes	NO	NO	LC*
	Trachydactylus hajarensis	NO	NO	NE
	Sphaerodactylidae			
	Pristurus carteri	NO	NO	LC
	Pristurus minimus	NO	NO	LC
	Pristurus sp. 1	NO	NO	NE
**Lacertids**		**0**	**0**	**1**
	Lacertidae			
	Mesalina adramitana	NO	NO	LC
**Skinks**		**0**	**0**	**1**
	Scincidae			
	Scincus mitranus	NO	NO	LC
**Amphisbaenids**		**0**	**0**	**1**
	Trogonophidae			
	Diplometopon zarudnyi	NO	NO	LC
**Varanids**		**0**	**0**	**1**
	Varanidae			
	Varanus griseus	NO	NO	LC*
**Snakes**		**0**	**3**	**7**
	Boidae			
	Eryx jayakari	NO	NO	LC
	Colubridae			
	Telescopus dhara dhara	NO	NO	LC*
	Lamprophiidae			
	Psammophis schokari	NO	NO	LC*
	Rhagerhis moilensis	NO	NO	LC*
	Viperidae			
	Cerastes gasperettii gasperettii	NO	YES	LC
	Echis carinatus sochureki	NO	YES	LC*
	Echis coloratus	NO	YES	LC*

* Not available on the web

Jabal Samhan				
**Taxa**	**Family/Specie**	**Endemics**	**Venomous**	**Total**
**Lizards**		**1**	**0**	**11**
**Agamids**		**0**	**0**	**2**
	Agamidae			
	Pseudotrapelus dhofarensis	NO	NO	NE
	Chamaeleonidae			
	Chamaeleo arabicus	NO	NO	LC
**Geckos**		**1**	**0**	**6**
	Gekkonidae			
	Hemidactylus alkiyumii	NO	NO	NE
	Hemidactylus paucituberculatus	YES	NO	NE
	Tropiocolotes scortecci	NO	NO	LC
	Phyllodactylidae			
	Ptyodactylus dhofarensis	NO	NO	NE
	Sphaerodactylidae			
	Pristurus carteri	NO	NO	LC
	Pristurus sp. 1	NO	NO	NE
**Lacertids**		**0**	**0**	**2**
	Lacertidae			
	Acanthodactylus felicis	NO	NO	VU
	Mesalina sp. 1	NO	NO	NE
**Skinks**		**0**	**0**	**1**
	Scincidae			
	Trachylepis tessellata	NO	NO	LC
**Snakes**		**0**	**2**	**6**
	Colubridae			
	Platyceps rhodorachis rhodorachis	NO	NO	LC*
	Platyceps thomasi	NO	NO	DD
	Spalerosophis diadema cliffordii	NO	NO	LC*
	Elapidae			
	Naja arabica	NO	YES	LC
	Lamprophiidae			
	Psammophis schokari	NO	NO	LC*
	Viperidae			
	Echis khosatzkii	NO	YES	LC

* Not available on the web

S121: Species list by protected areas

Khawr Rawri, Khawr Taqah, Khawr Sawli, Khawr Dahareez, Khawr Baleed, Khawr Salalah, Khawr Awqad, Khawr Qurum Al Kabeer, Khawr Qurum Al Sagher & Khawr Mughsayi

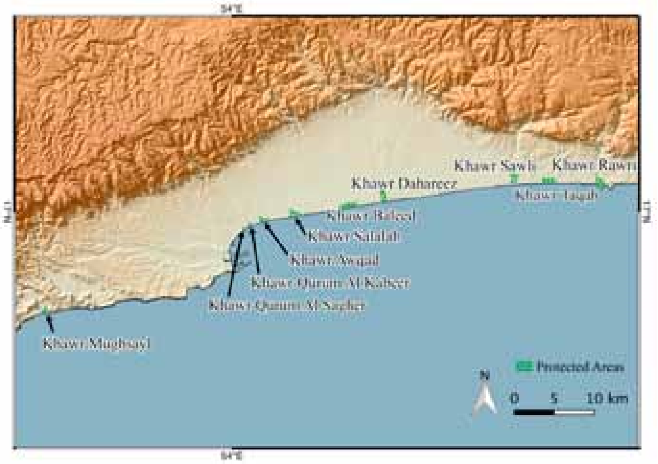

Khawr Rawri				
**Taxa**	**Family/Specie**	**Endemics**	**Venomous**	**Total**
**Lizards**		**1**	**0**	**5**
**Agamids**		**0**	**0**	**1**
	Agamidae			
	Pseudotrapelus dhofarensis	NO	NO	NE
**Geckos**		**1**	**0**	**4**
	Gekkonidae			
	Hemidactylus alkiyumii	NO	NO	NE
	Hemidactylus paucituberculatus	YES	NO	NE
	Sphaerodactylidae			
	Pristurus carteri	NO	NO	LC
	Pristurus sp. 1	NO	NO	NE
**Snakes**		**0**	**0**	**0**

* Not available on the web

Khawr Taqah				
**Taxa**	**Family/Specie**	**Endemics**	**Venomous**	**Total**
**Lizards**		**0**	**0**	**0**
**Snakes**		**0**	**0**	**0**

* Not available on the web

Khawr Dahareez				
**Taxa**	**Family/Specie**	**Endemics**	**Venomous**	**Total**
**Lizards**		**0**	**0**	**4**
**Agamids**		**0**	**0**	**1**
	Agamidae			
	Pseudotrapelus dhofarensis	NO	NO	NE
**Geckos**		**0**	**0**	**3**
	Gekkonidae			
	Hemidactylus robustus	NO	NO	LC*
	Trachydactylus spatalurus	NO	NO	NE
	Sphaerodactylidae			
	Pristurus carteri	NO	NO	LC
**Snakes**		**0**	**0**	**2**
	Leptotyphlopidae			
	Myriopholis macrorhyncha	NO	NO	LC*
	Myriopholis nursii	NO	NO	LC*

* Not available on the web

Khawr Sawli				
**Taxa**	**Family/Specie**	**Endemics**	**Venomous**	**Total**
**Lizards**		**0**	**0**	**0**
**Snakes**		**0**	**0**	**0**

* Not available on the web

Khawr Baleed				
**Taxa**	**Family/Specie**	**Endemics**	**Venomous**	**Total**
**Lizards**		**0**	**0**	**0**
**Snakes**		**0**	**0**	**0**

* Not available on the web

Khawr Salalah				
**Taxa**	**Family/Specie**	**Endemics**	**Venomous**	**Total**
**Lizards**		**0**	**0**	**0**
**Snakes**		**0**	**0**	**0**

* Not available on the web

Khawr Awqad				
**Taxa**	**Family/Specie**	**Endemics**	**Venomous**	**Total**
**Lizards**		**0**	**0**	**0**
**Snakes**		**0**	**0**	**0**

* Not available on the web

Khawr Qurum Al Kabeer				
**Taxa**	**Family/Specie**	**Endemics**	**Venomous**	**Total**
**Lizards**		**0**	**0**	**0**
**Snakes**		**0**	**0**	**0**

* Not available on the web

Khawr Qurum Al Sagher				
**Taxa**	**Family/Specie**	**Endemics**	**Venomous**	**Total**
**Lizards**		**0**	**0**	**0**
**Snakes**		**0**	**0**	**0**

* Not available on the web

Khawr Mughsayl				
**Taxa**	**Family/Specie**	**Endemics**	**Venomous**	**Total**
**Lizards**		**0**	**0**	**0**
**Snakes**		**0**	**0**	**0**

* Not available on the web

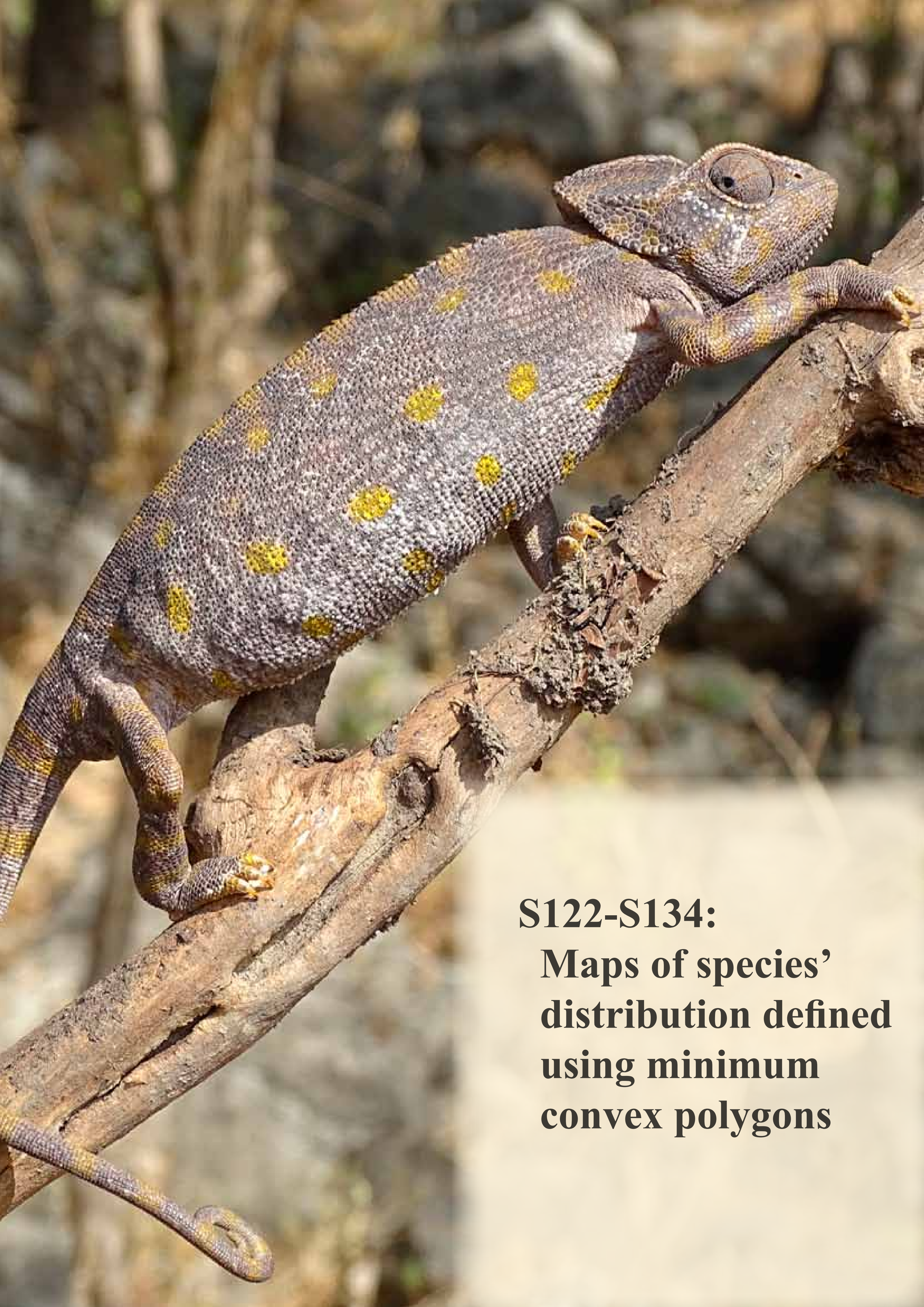

S122-S134:
Maps of species' distribution defined using minimum convex polygons

S122-S134: Maps of species' distribution defined using minimum convex polygons

Plates including independent maps for each species showing the 22 pretected areas of Oman and the species' distribution area defined using the extent of occurrence with a minimum convex polygon (MCP) of the observations filtred by species' average altitude.

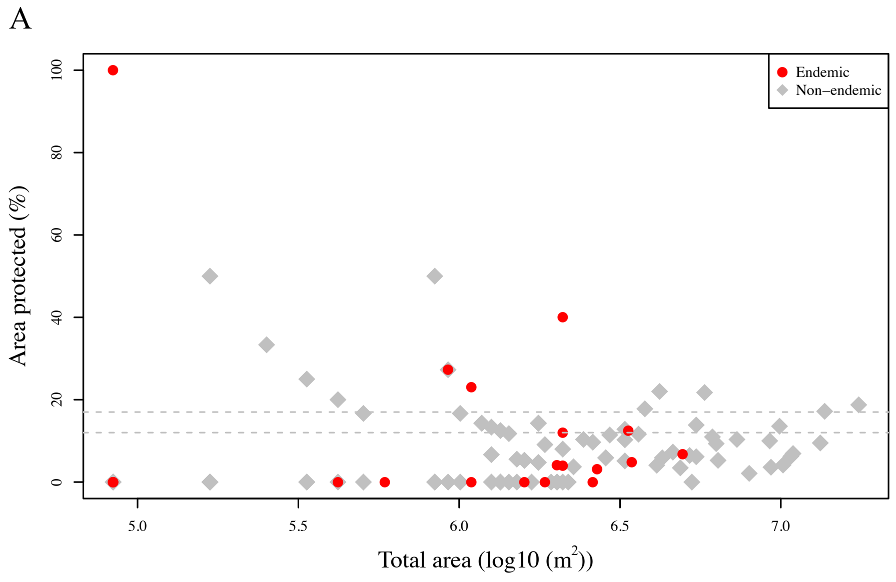

Percentage of the species' distribution area included within a protected area (see material and methods). Dashed lines indicate the conservation target of 17% and 12% of the total species' distribution area within a protected area. A) The extent of species occurrence was defined using the presence-absence in every pixel of 1 km x 1 km. B) The extent of species occurrence was defined using a minimum convex polygon (MCP) of the observations filtred by species' average altitude.

S122: Maps of species' distribution defined using minimum convex polygons

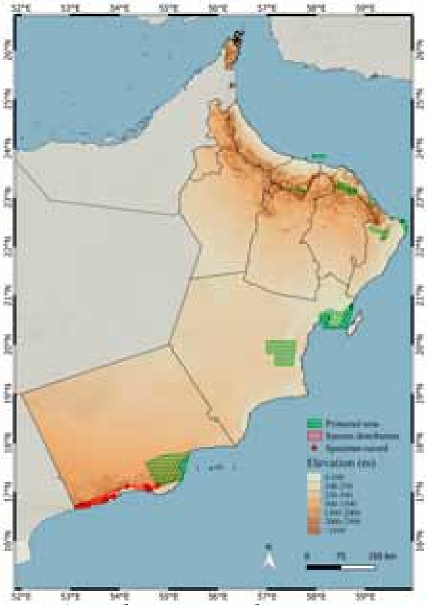

Acanthocercus adramitanus

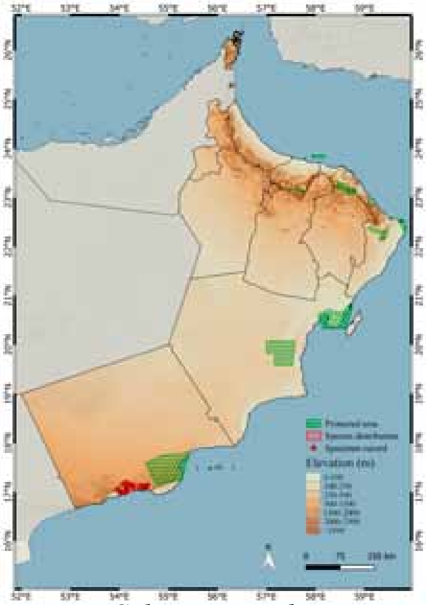

Calotes versicolor

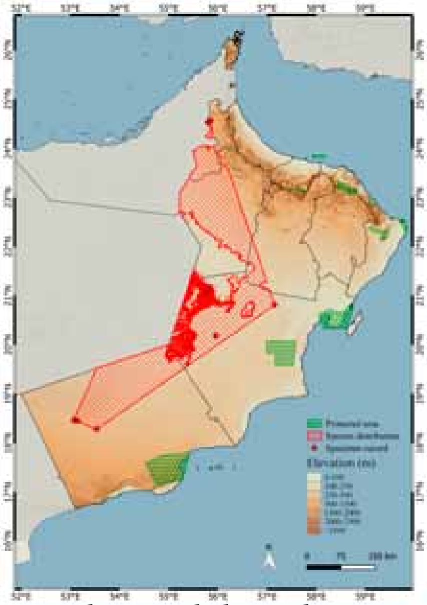

Phrynocephalus arabicus

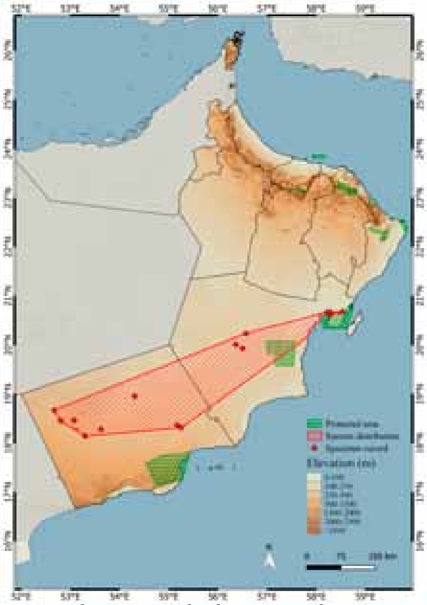

Phrynocephalus maculatus

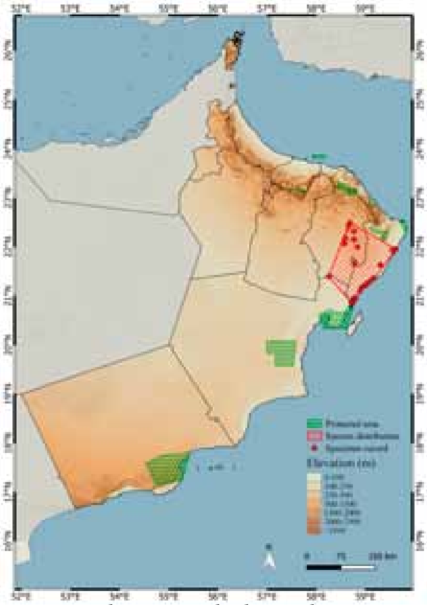

Phrynocephalus sakoi

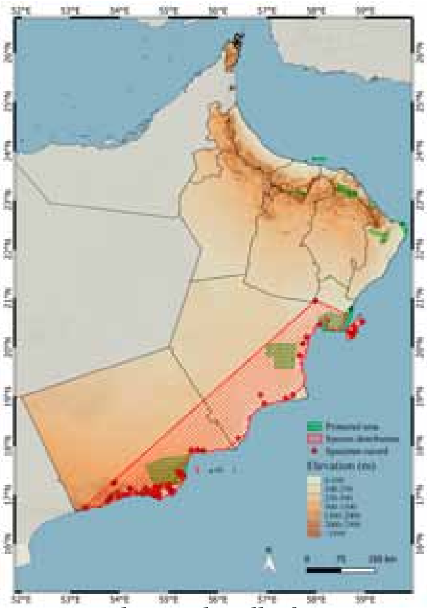

Pseudotrapelus dhofarensis

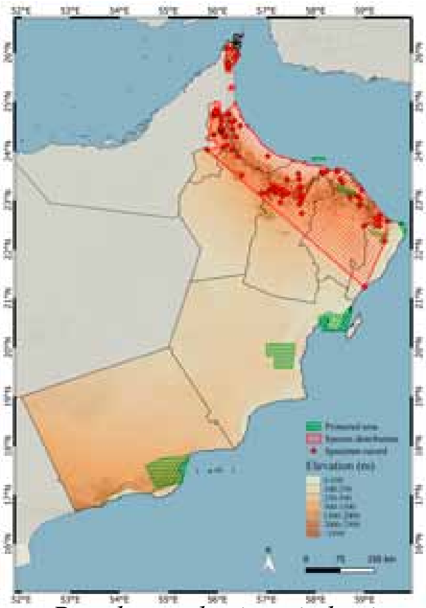

Pseudotrapelus jensvindumi

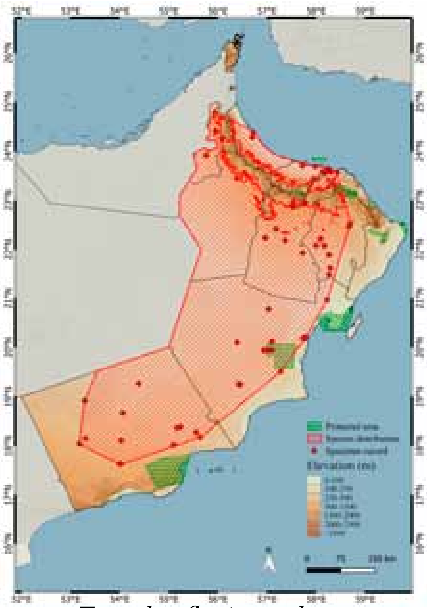

Trapelus flavimaculatus

S123: Maps of species' distribution defined using minimum convex polygons

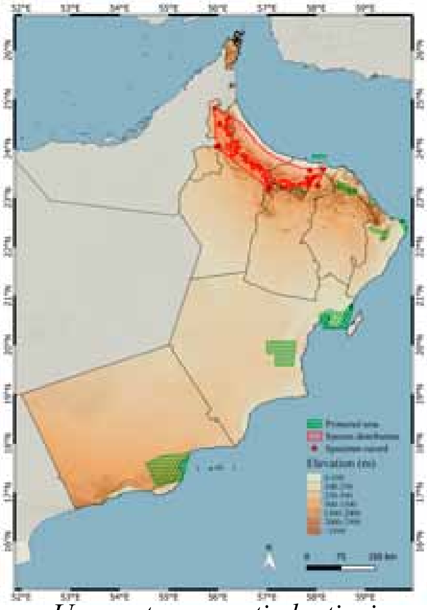

Uromastyx aegyptia leptieni

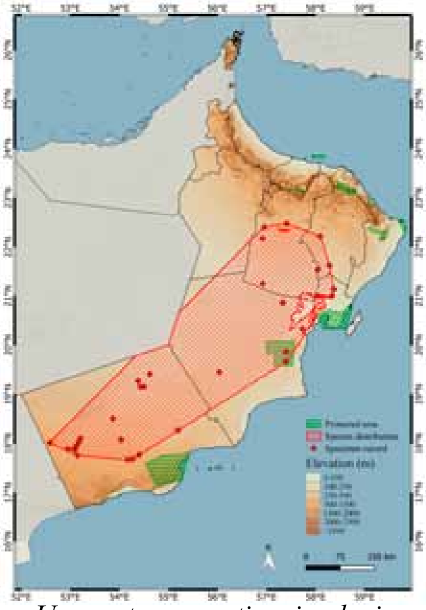

Uromastyx aegyptia microlepis

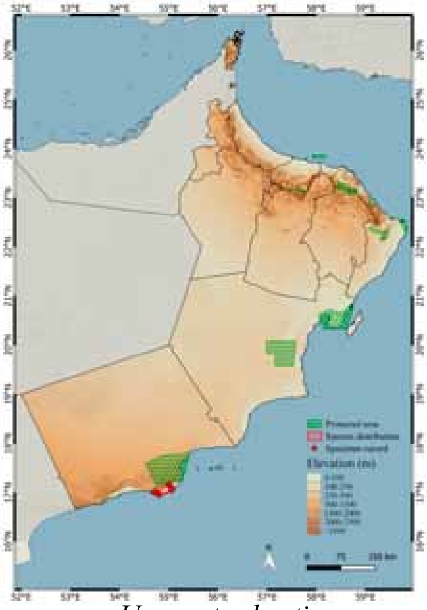

Uromastyx benti

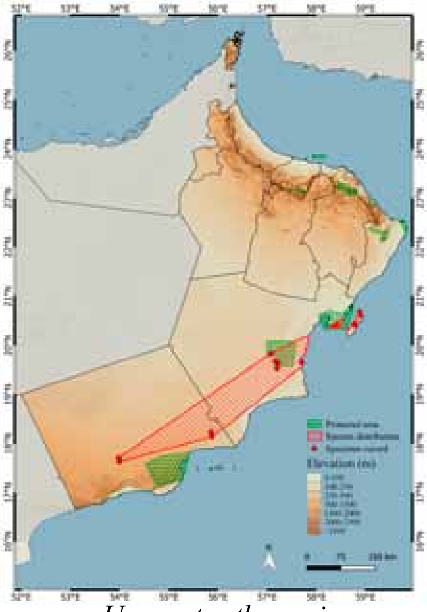

Uromastyx thomasi

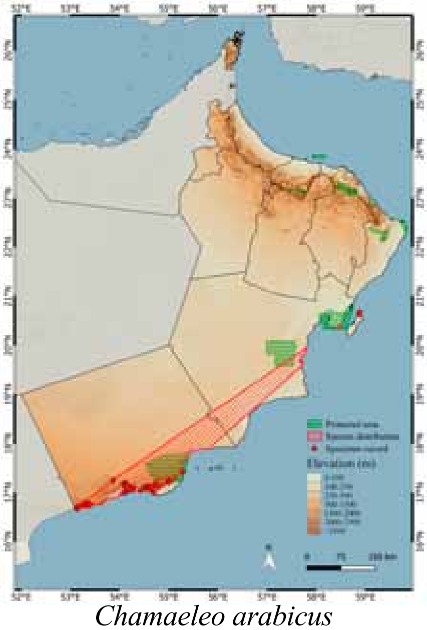

Chamaeleo arabicus

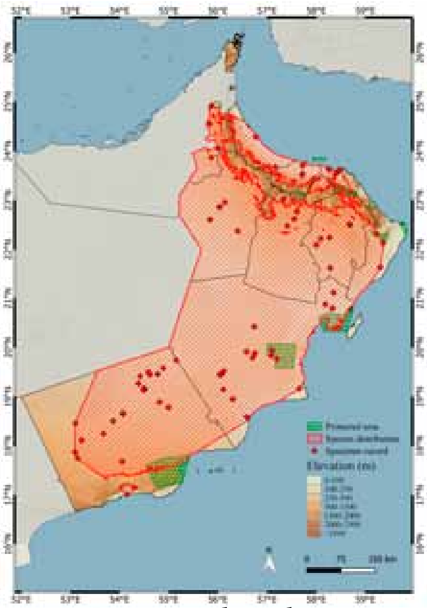

Bunopus tuberculatus

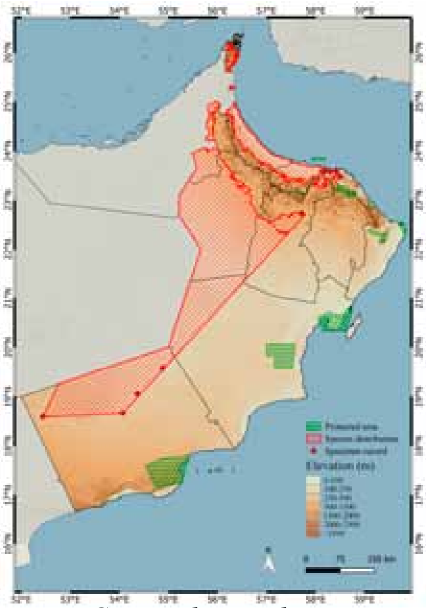

Cyrtopodion scabrum

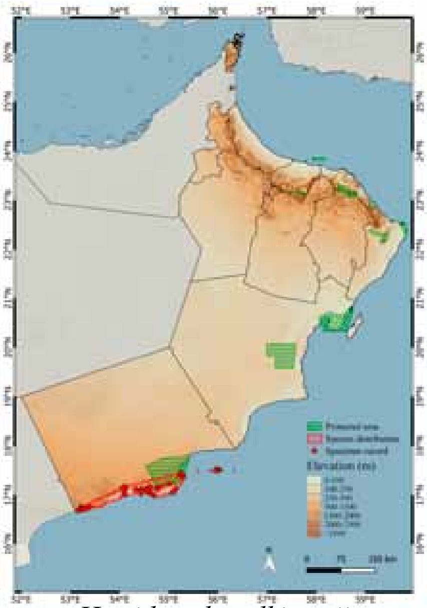

Hemidactylus alkiyumii

S124: Maps of species' distribution defined using minimum convex polygons

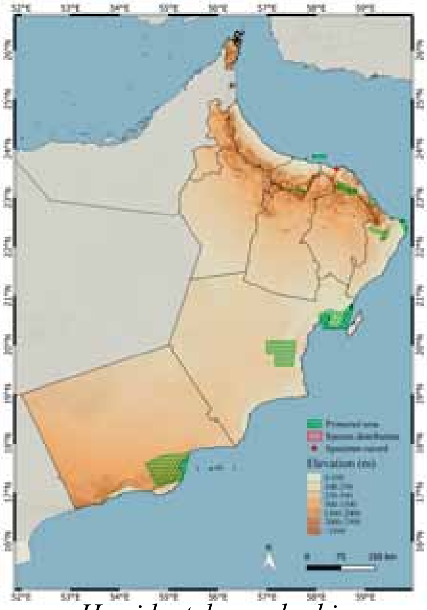

Hemidactylus endophis

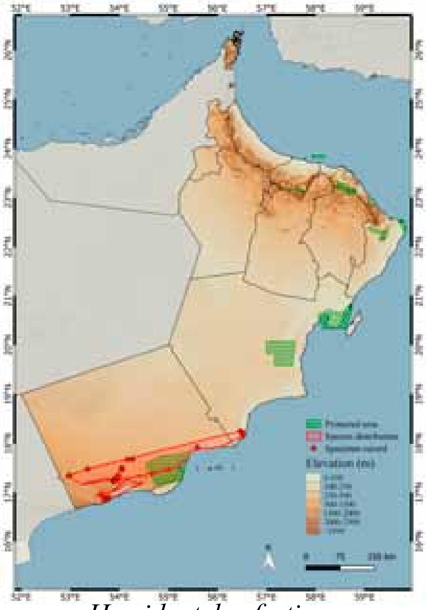

Hemidactylus festivus

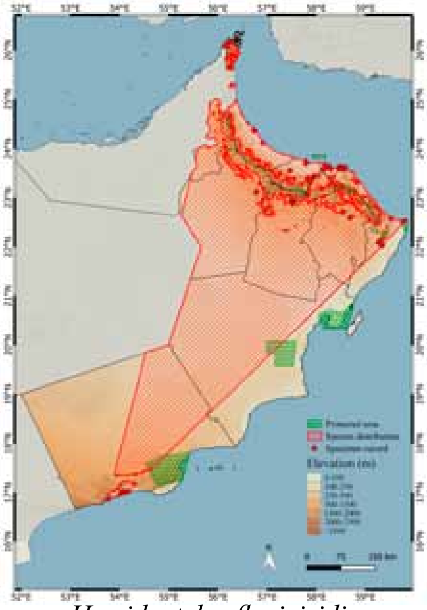

Hemidactylus flaviviridis

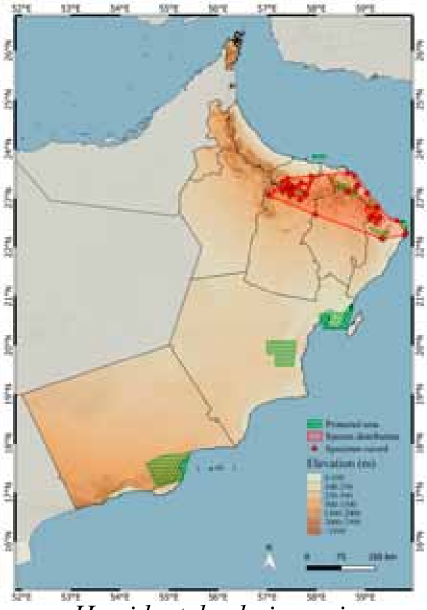

Hemidactylus hajarensis

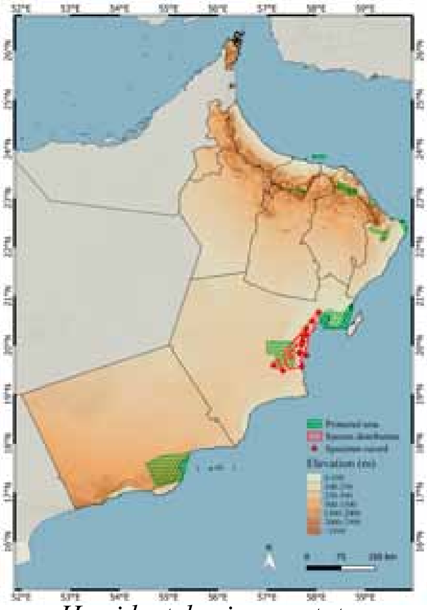

Hemidactylus inexpectatus

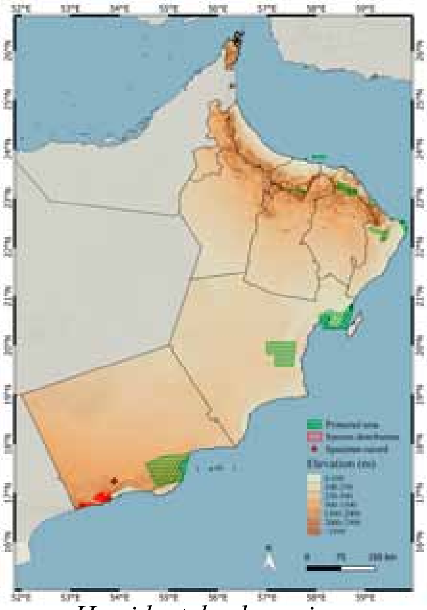

Hemidactylus lemurinus

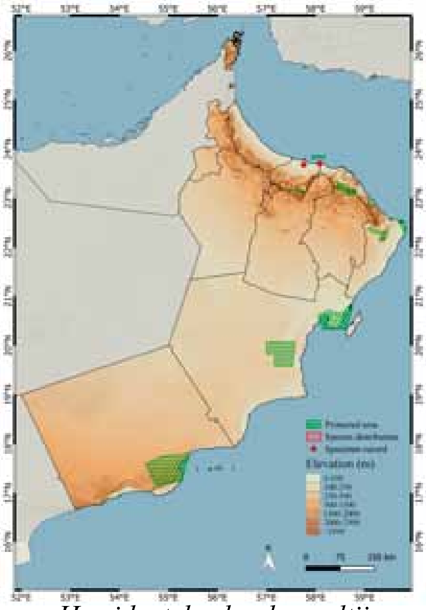

Hemidactylus leschenaultii

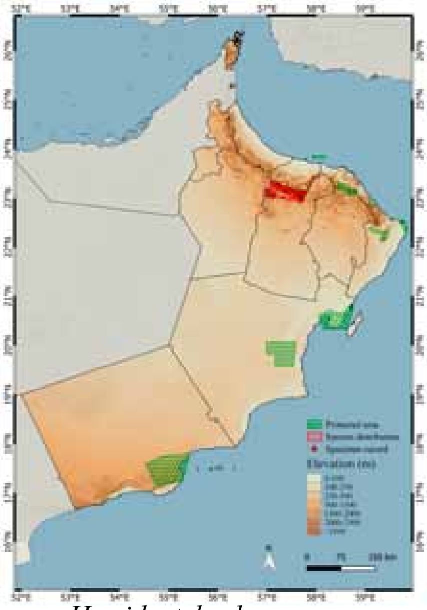

Hemidactylus luqueorum

S125: Maps of species' distribution defined using minimum convex polygons

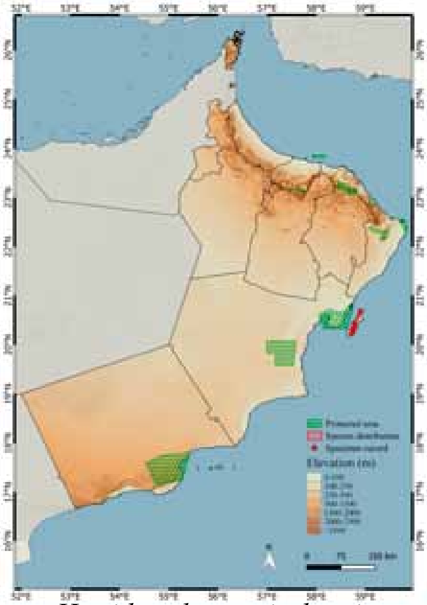

Hemidactylus masirahensis

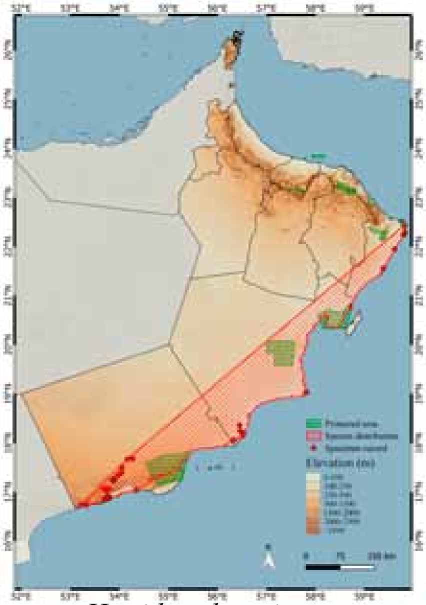

Hemidactylus minutus

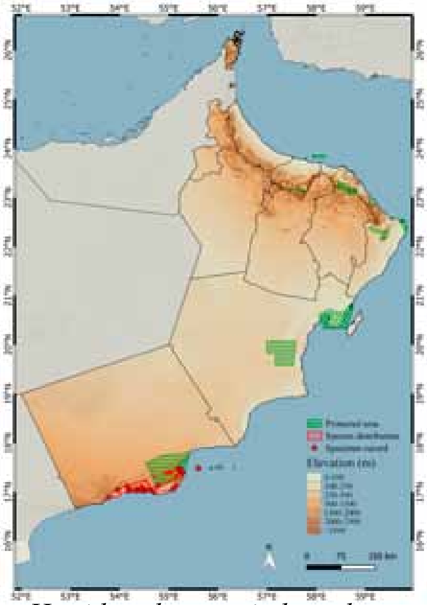

Hemidactylus paucituberculatus

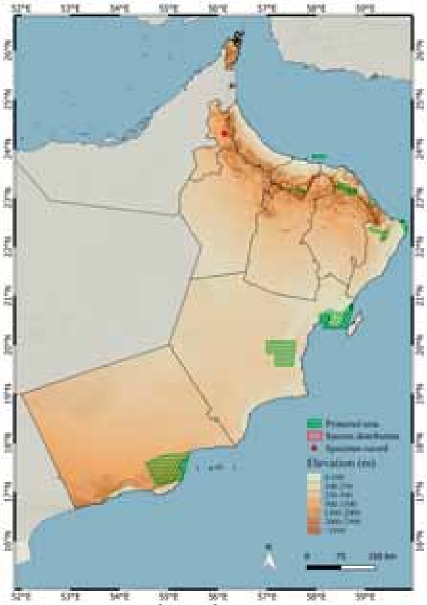

Hemidactylus persicus

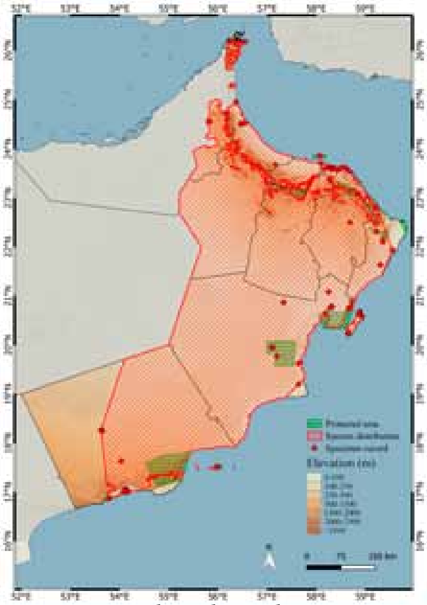

Hemidactylus robustus

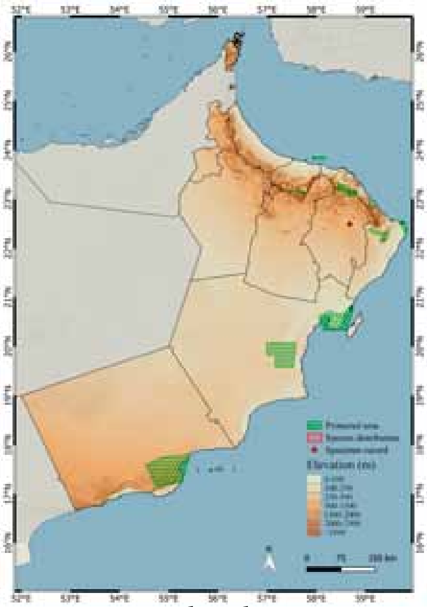

Hemidactylus sp.

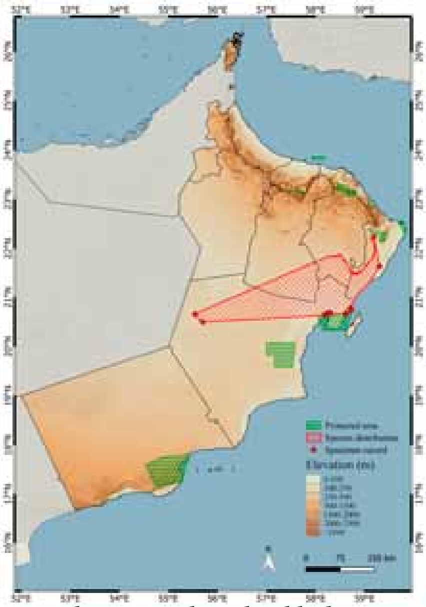

Pseudoceramodactylus khobarensis

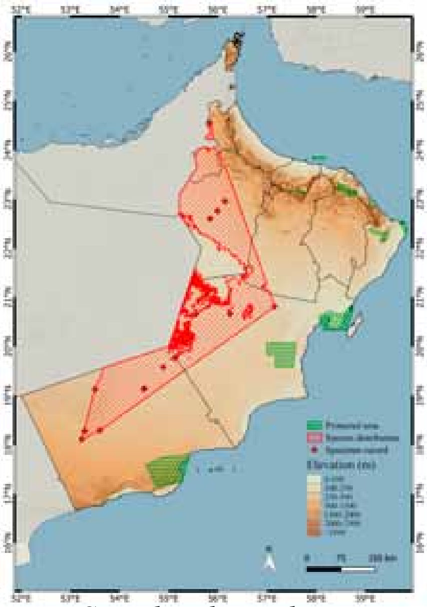

Stenodactylus arabicus

S126: Maps of species' distribution defined using minimum convex polygons

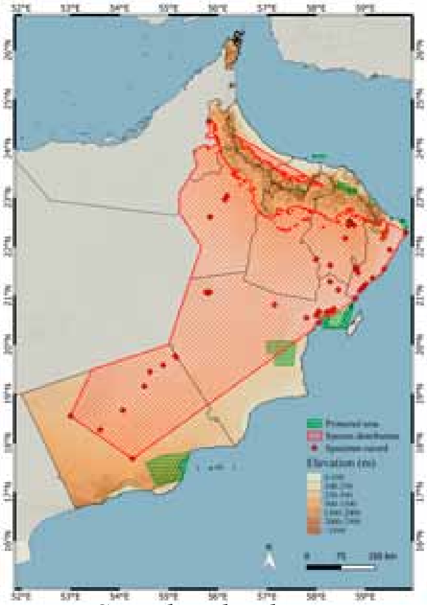

Stenodactylus doriae

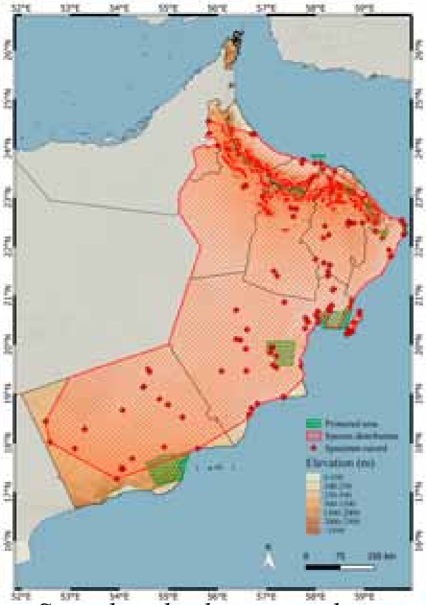

Stenodactylus leptocosymbotes

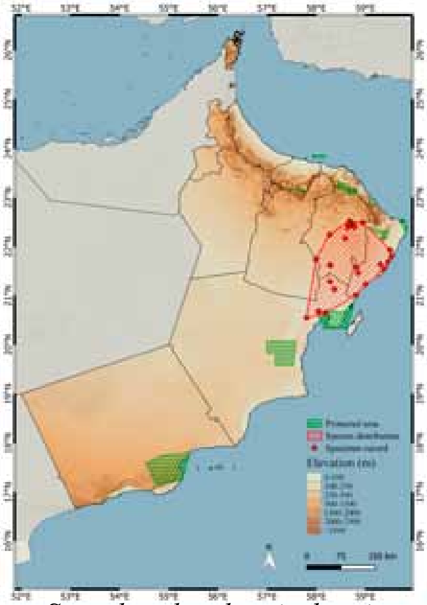

Stenodactylus sharqiyahensis

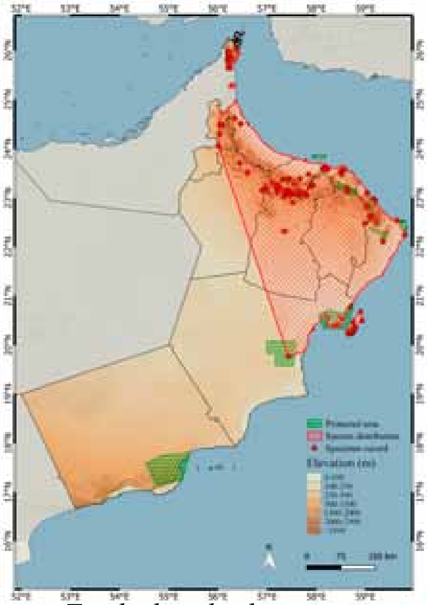

Trachydactylus hajarensis

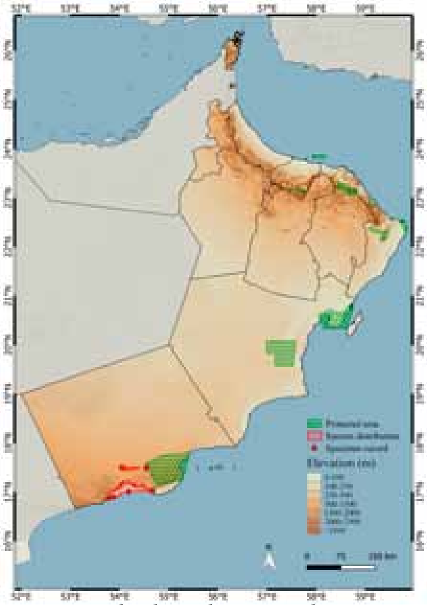

Trachydactylus spatalurus

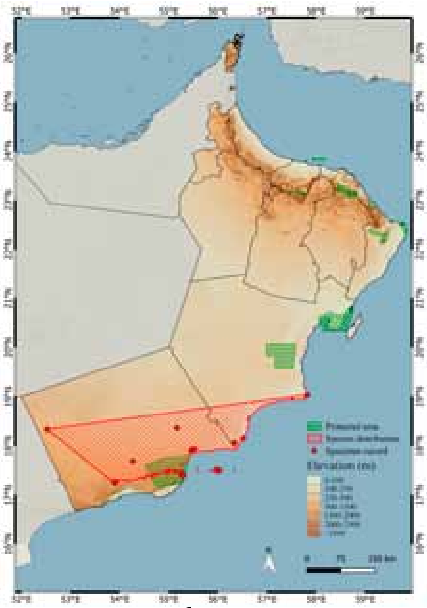

Tropiocolotes scortecci

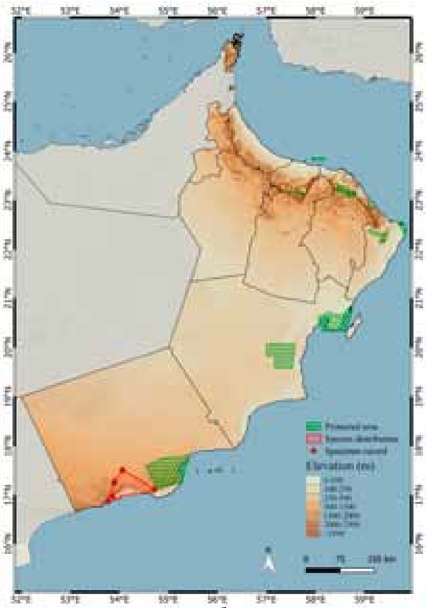

Tropiocolotes sp.

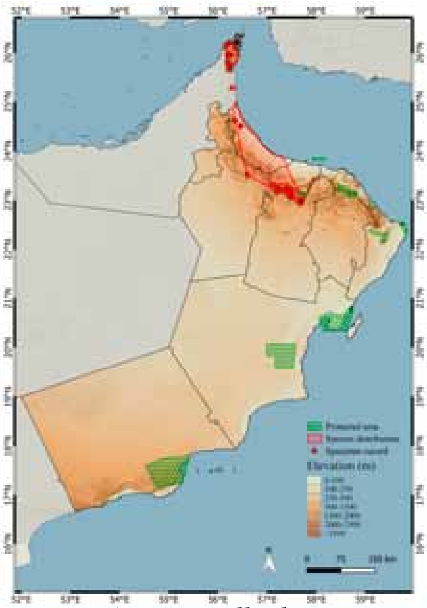

Asaccus gallagheri

S127: Maps of species' distribution defined using minimum convex polygons

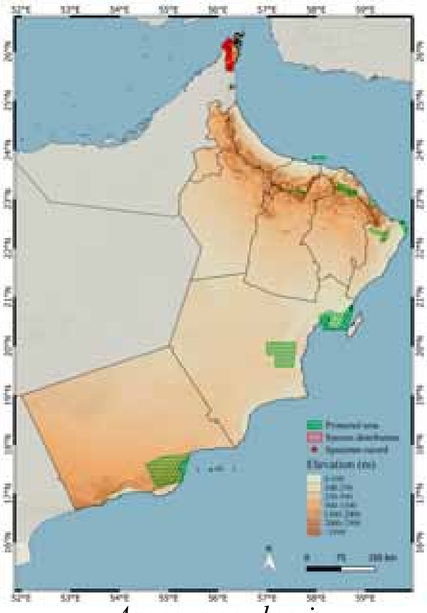

Asaccus gardneri

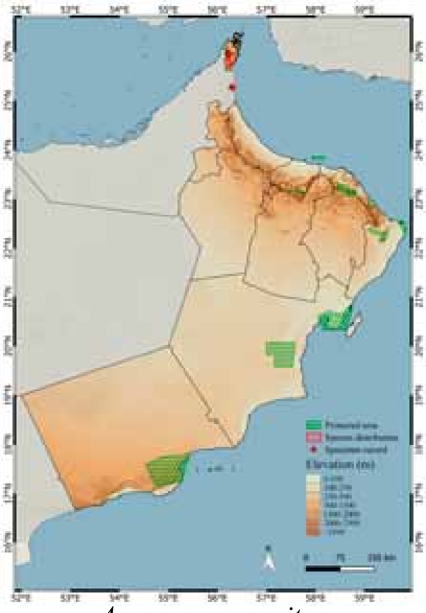

Asaccus margaritae

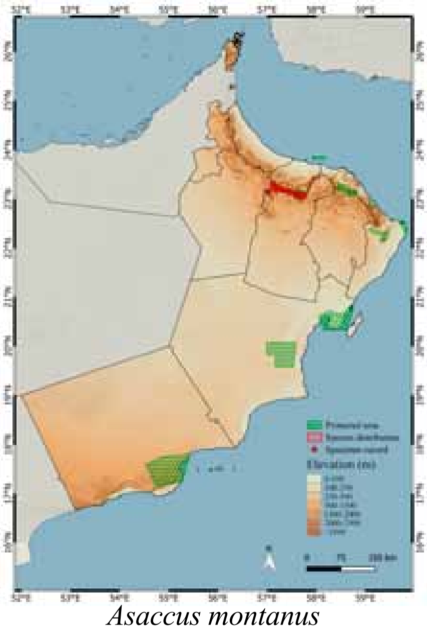

Asaccus montanus

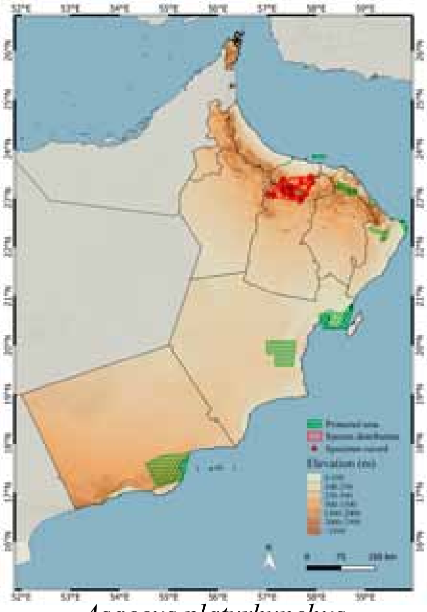

Asaccus platyrhynchus

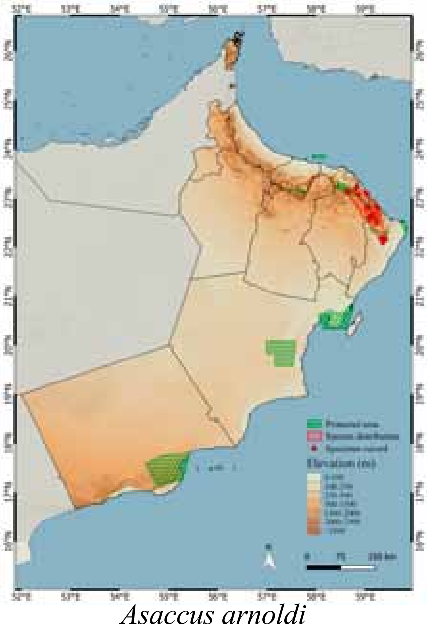

Asaccus arnoldi

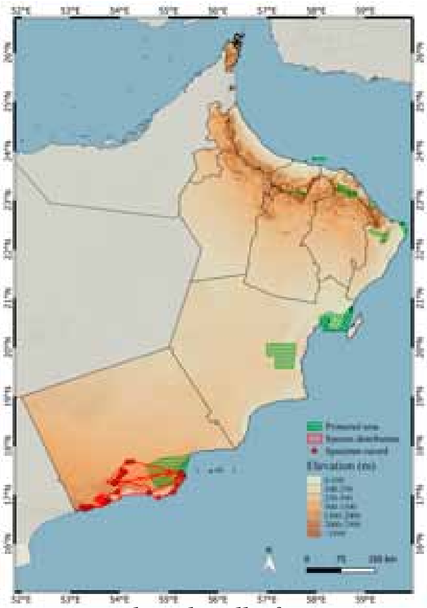

Ptyodactylus dhofarensis

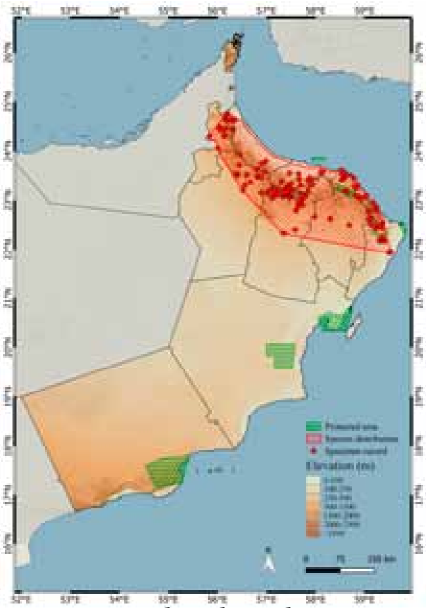

Ptyodactylus orlovi

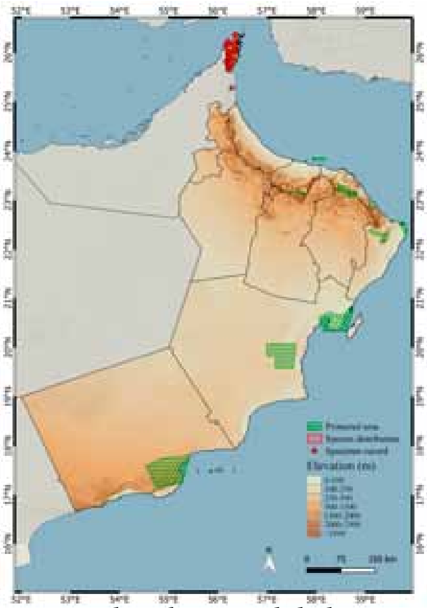

Ptyodactylus ruusaljibalicus

S128: Maps of species' distribution defined using minimum convex polygons

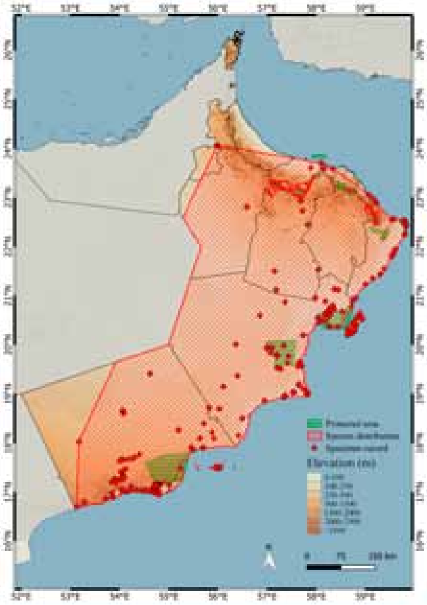

Pristurus carteri

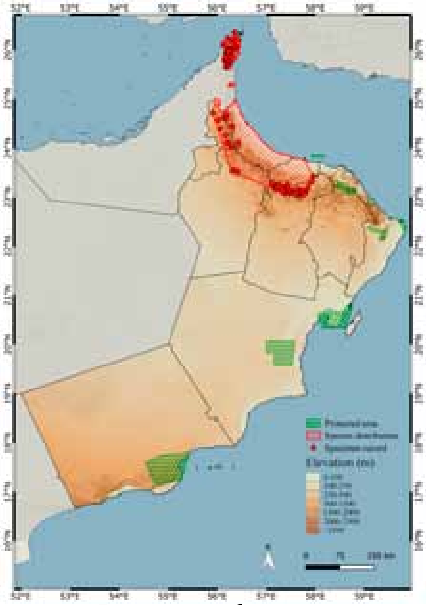

Pristurus celerrimus

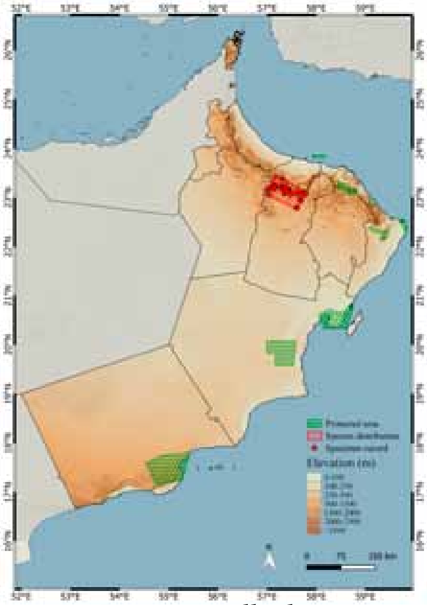

Pristurus gallagheri

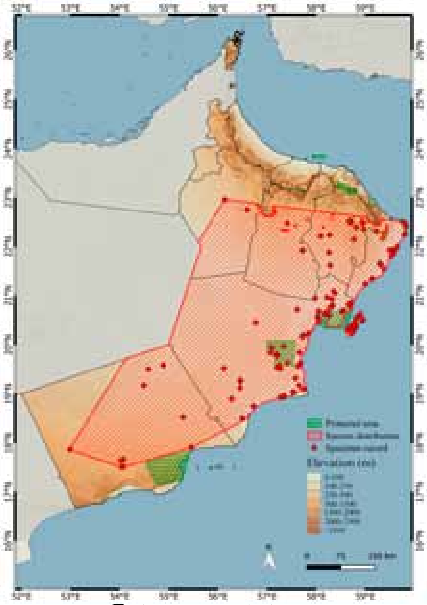

Pristurus minimus

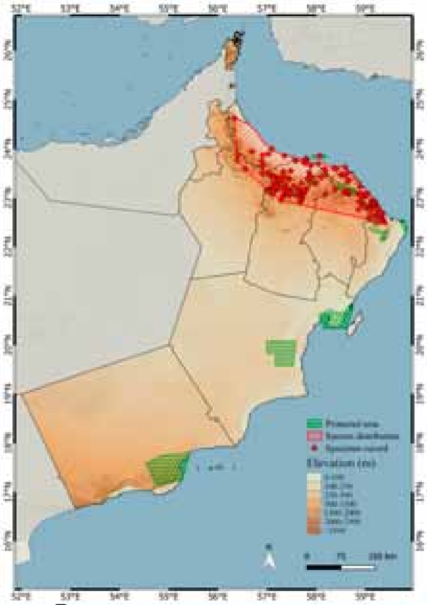

Pristurus rupestris rupestris

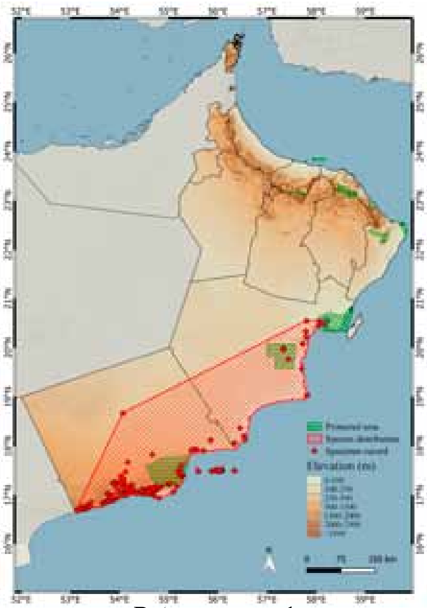

Pristurus sp. 1

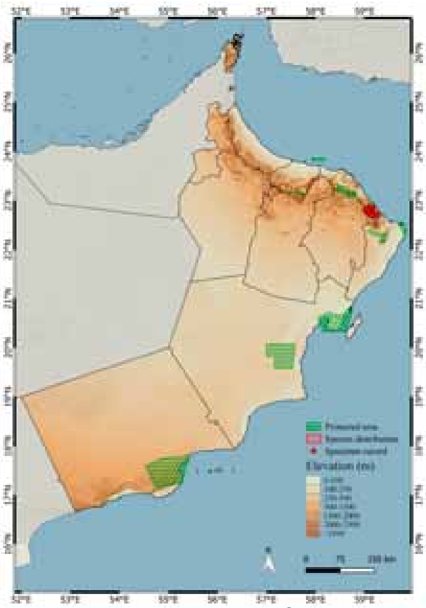

Pristurus sp. 2

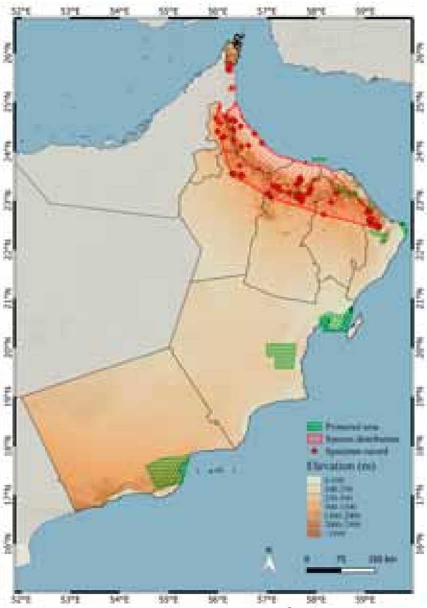

Pristurus sp. 3

S129: Maps of species' distribution defined using minimum convex polygons

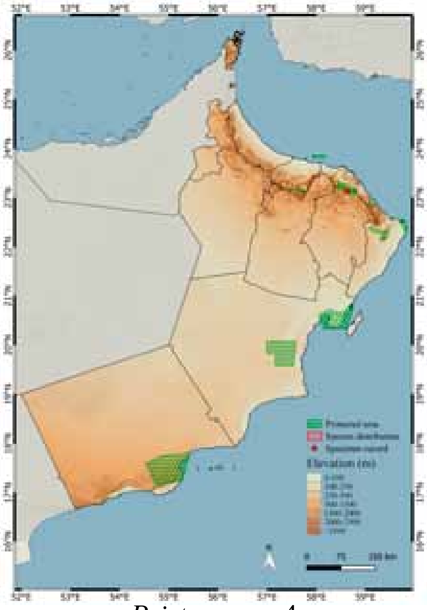

Pristurus sp. 4

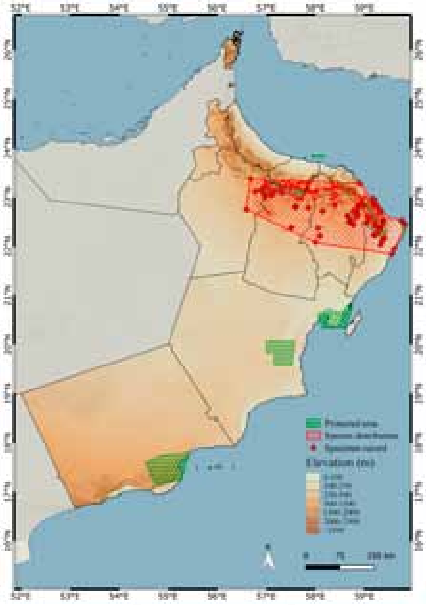

Pristurus sp. 5

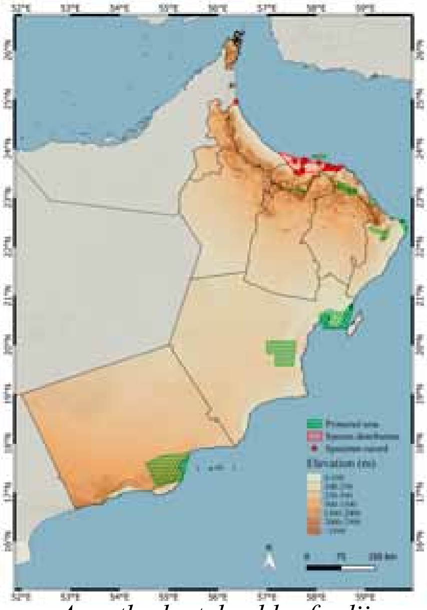

Acanthodactylus blanfordii

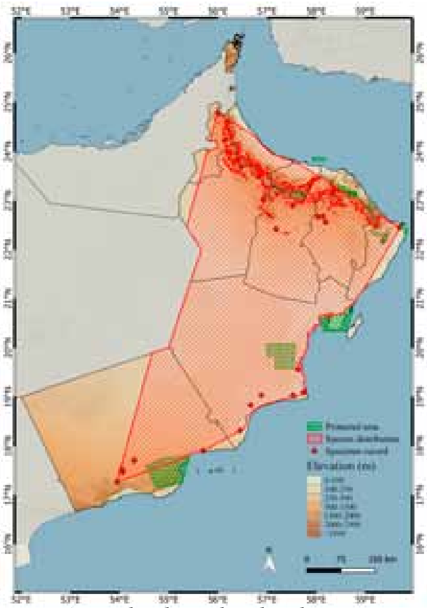

Acanthodactylus boskianus

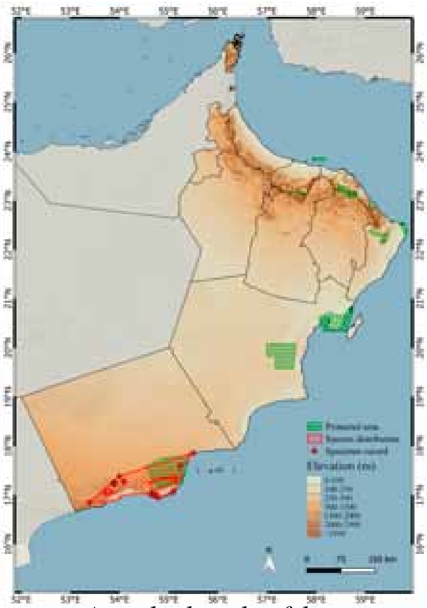

Acanthodactylus felicis

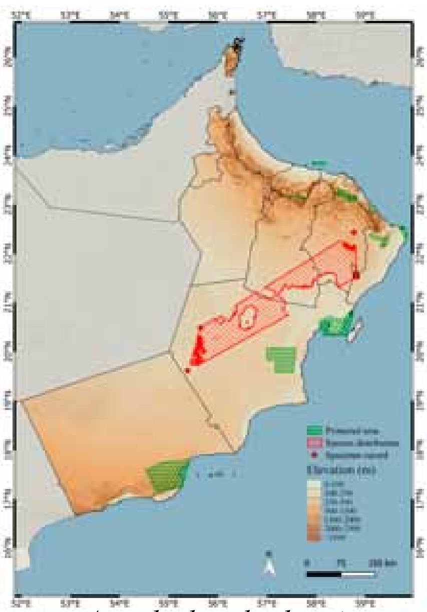

Acanthodactylus haasi

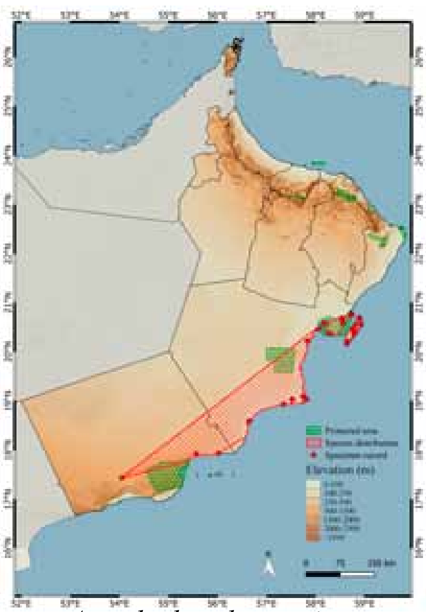

Acanthodactylus masirae

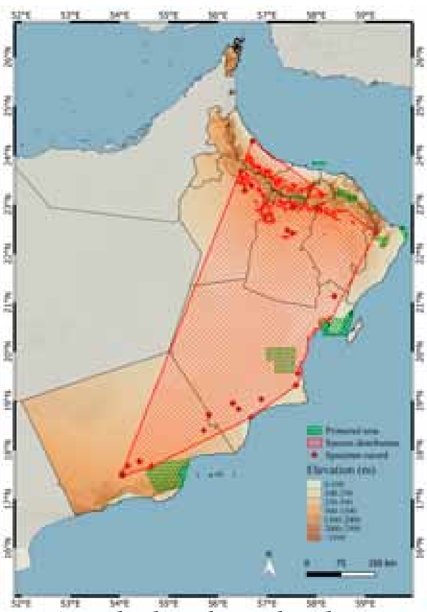

Acanthodactylus opheodurus

S130: Maps of species' distribution defined using minimum convex polygons

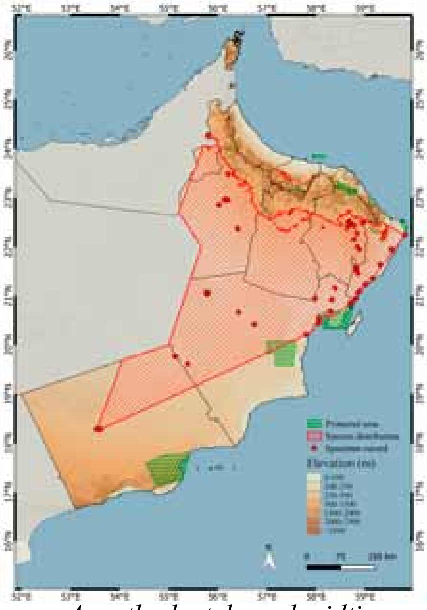

Acanthodactylus schmidti

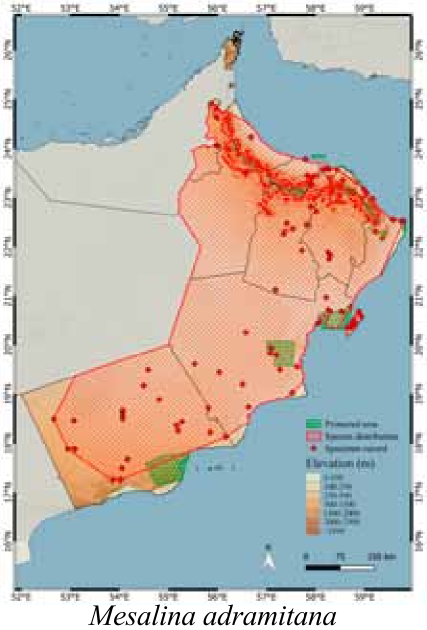

Mesalina adramitana

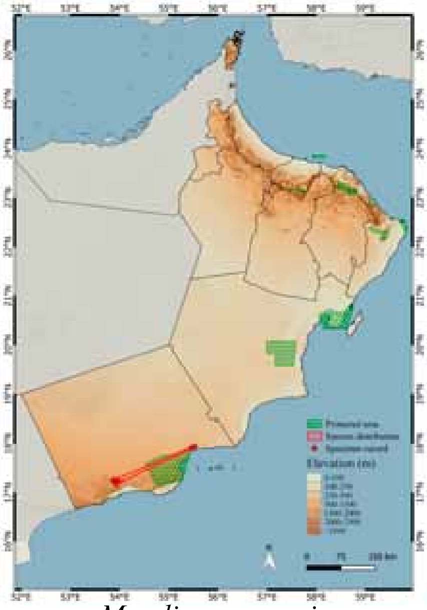

Mesalina ayunensis

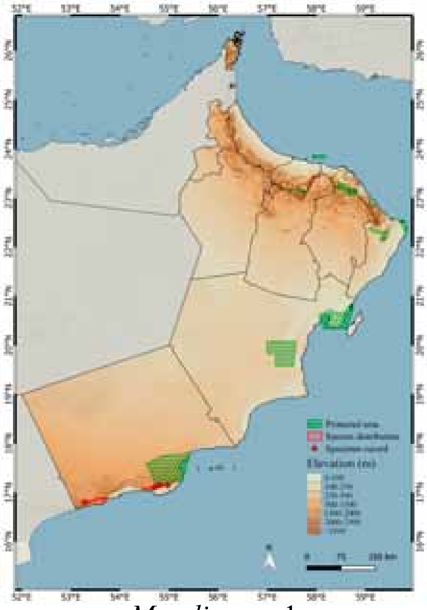

Mesalina sp. 1

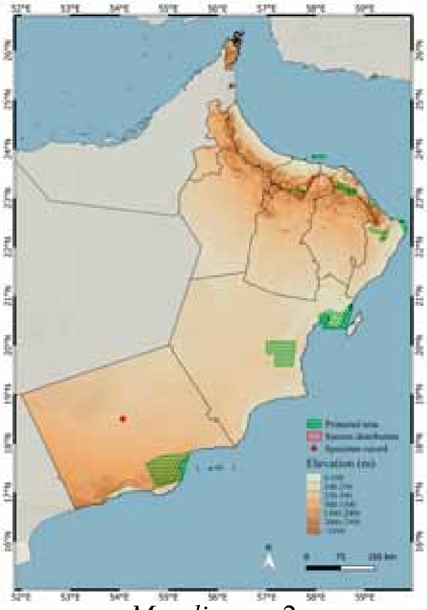

Mesalina sp. 2

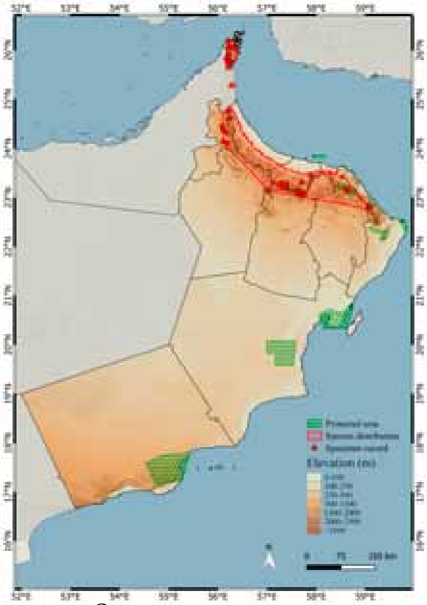

Omanosaura cyanura

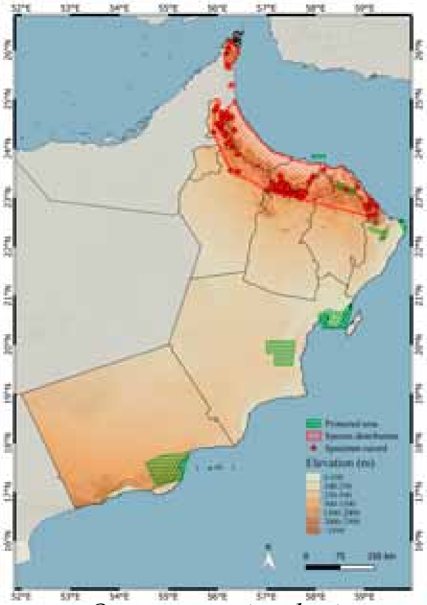

Omanosaura jayakari

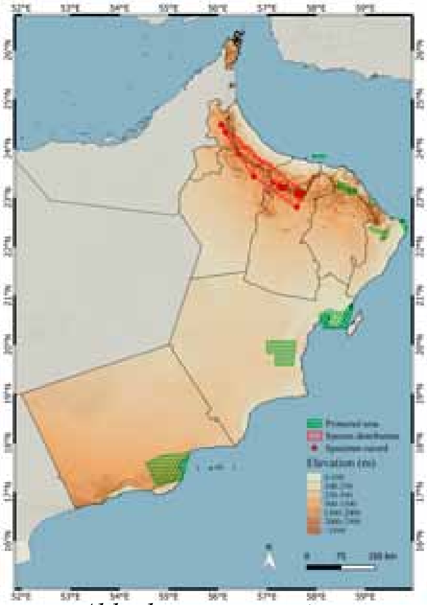

Ablepharus pannonicus

S131: Maps of species' distribution defined using minimum convex polygons

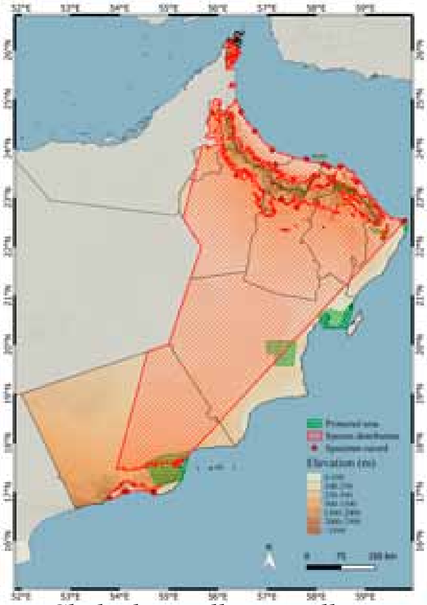
Chalcides ocellatus ocellatus

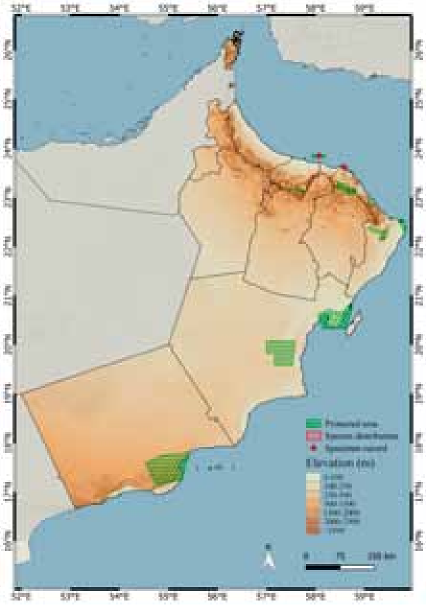
Heremites septemtaeniatus

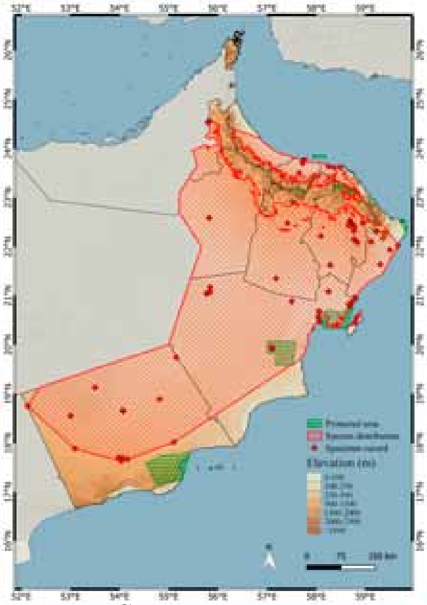
Scincus mitranus

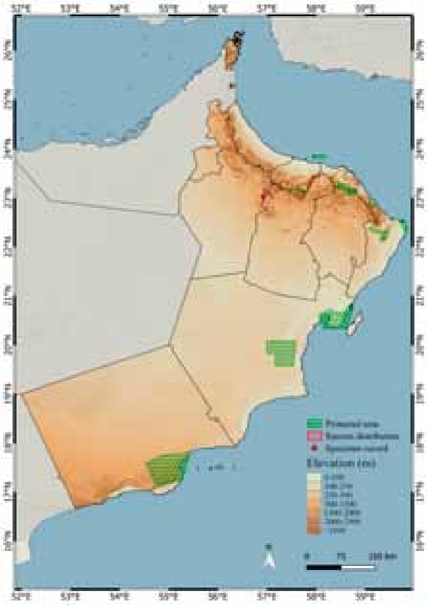
Scincus scincus conirostris

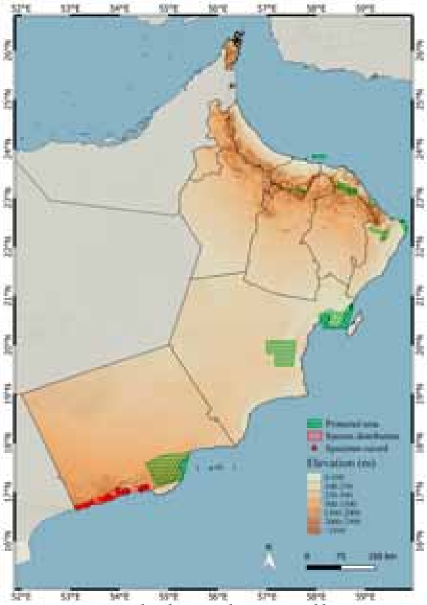
Trachylepis brevicollis

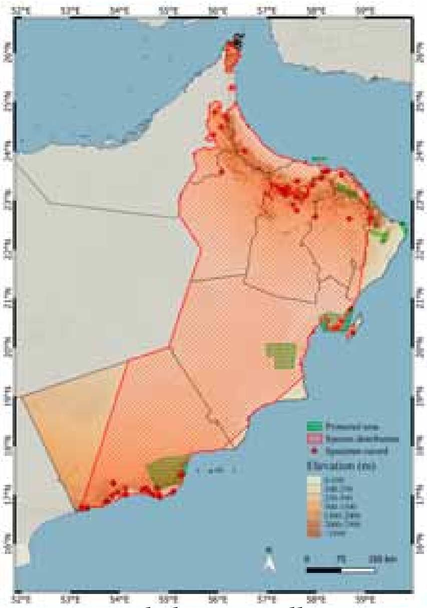
Trachylepis tessellata

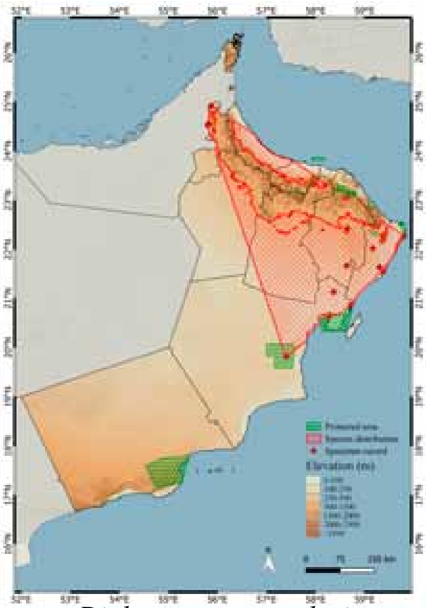
Diplometopon zarudnyi

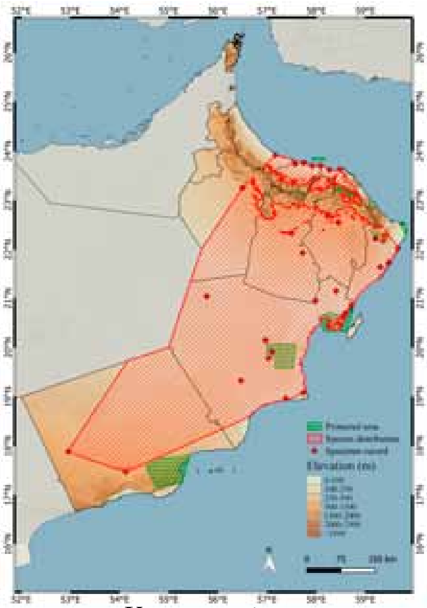
Varanus griseus

S132: Maps of species' distribution defined using minimum convex polygons

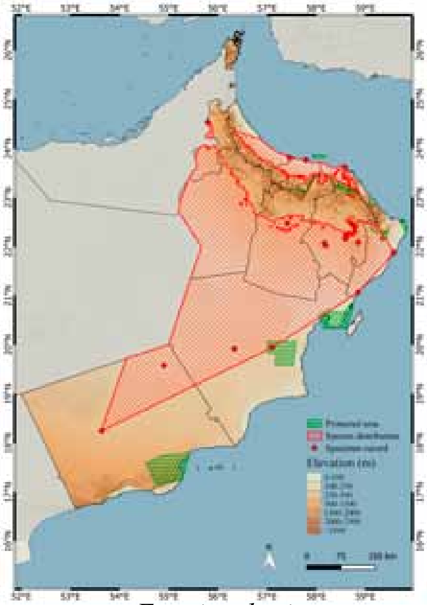

Eryx jayakari

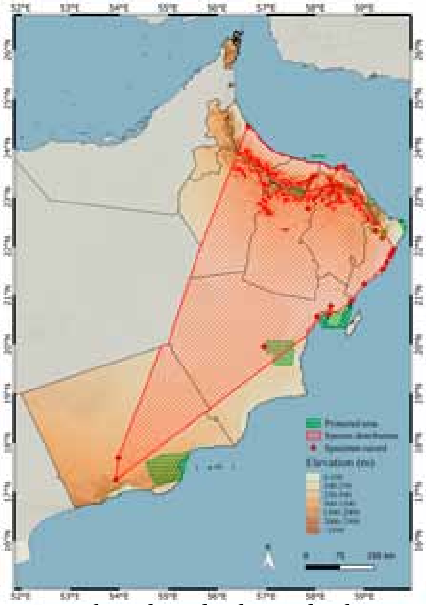

Lytorhynchus diadema diadema

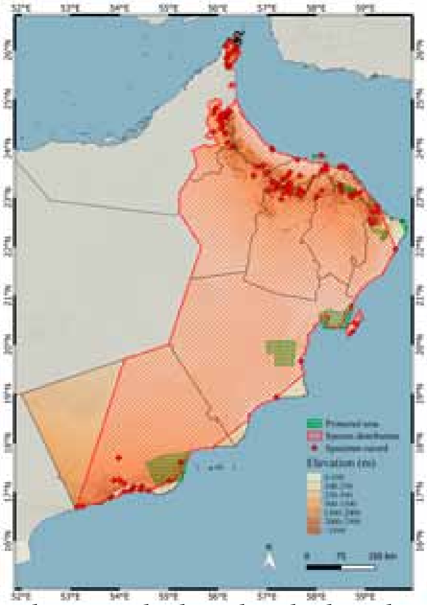

Platyceps rhodorachis rhodorachis

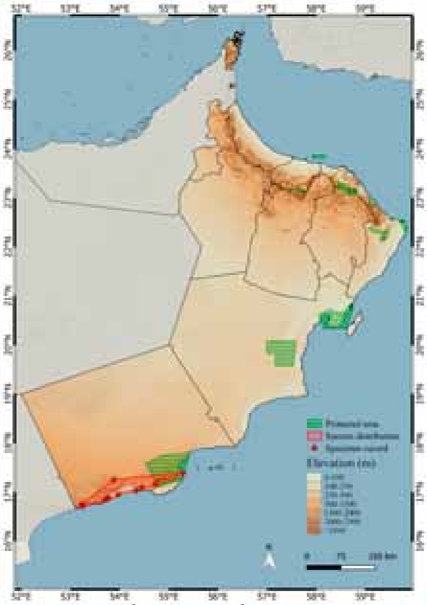

Platyceps thomasi

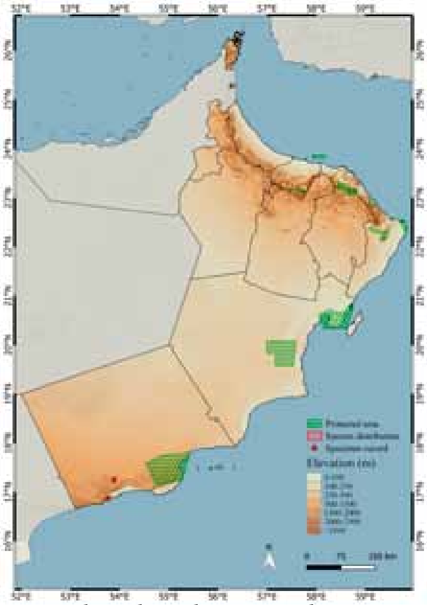

Rhynchocalamus arabicus

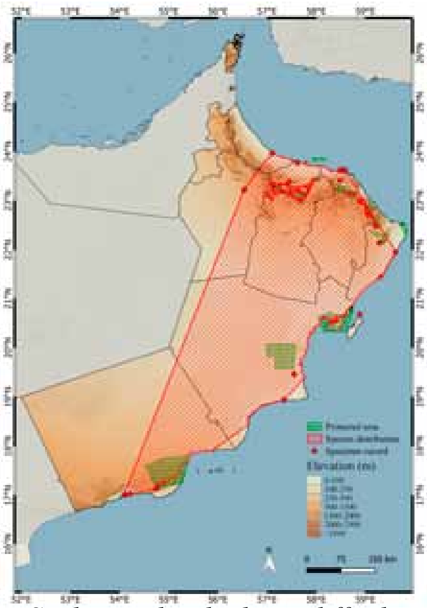

Spalerosophis diadema cliffordii

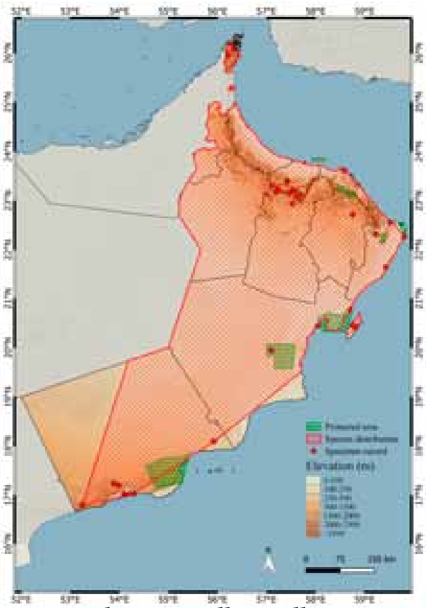

Telescopus dhara dhara

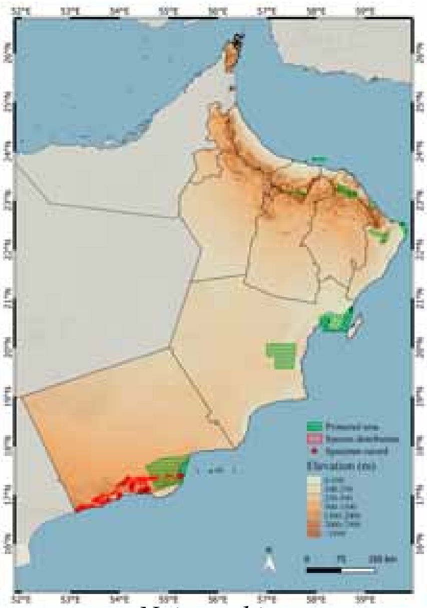

Naja arabica

S133: Maps of species' distribution defined using minimum convex polygons

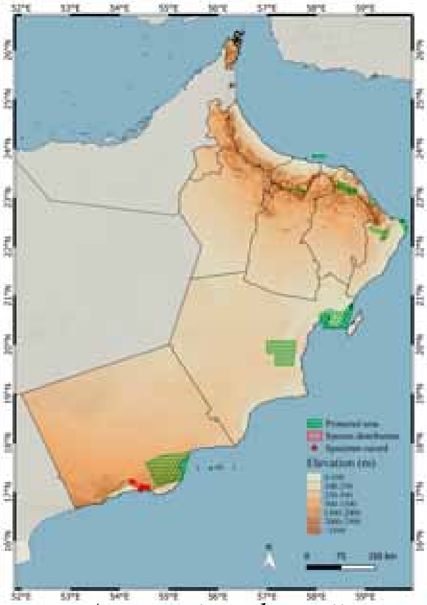

Atractaspis andersonii

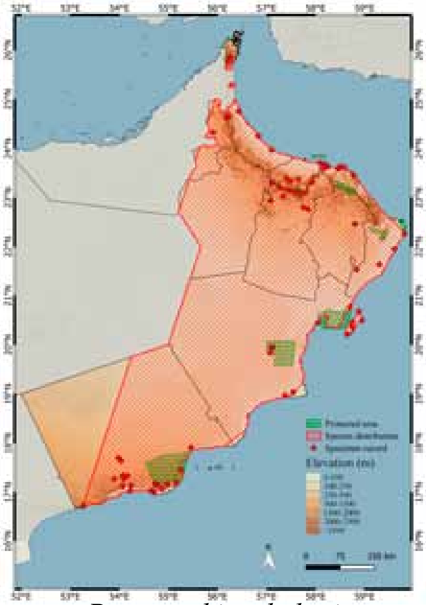

Psammophis schokari

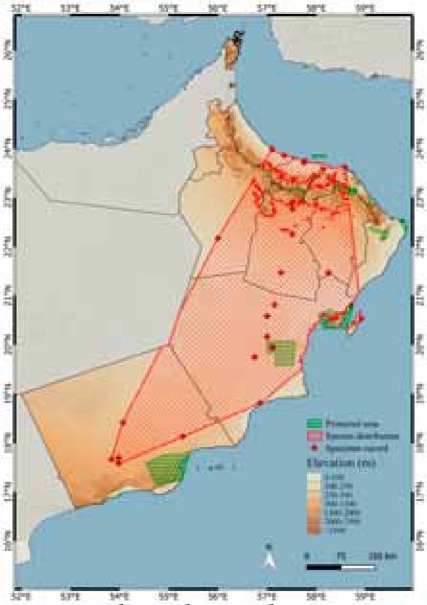

Rhagerhis moilensis

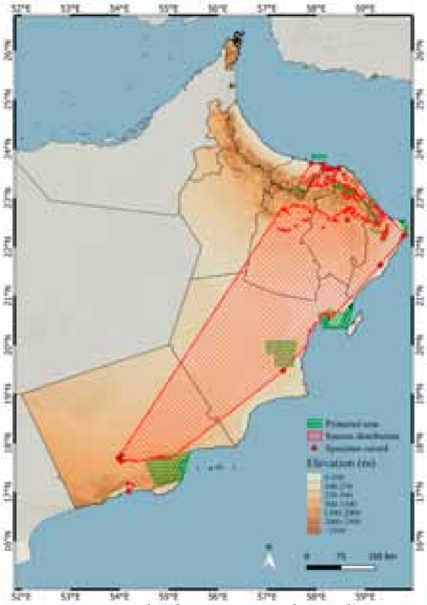

Myriopholis macrorhyncha

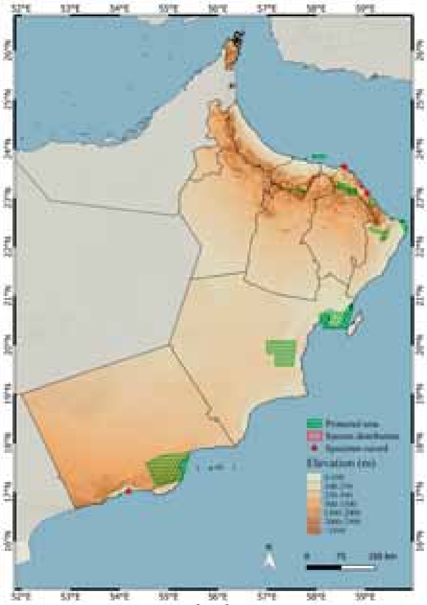

Myriopholis nursii

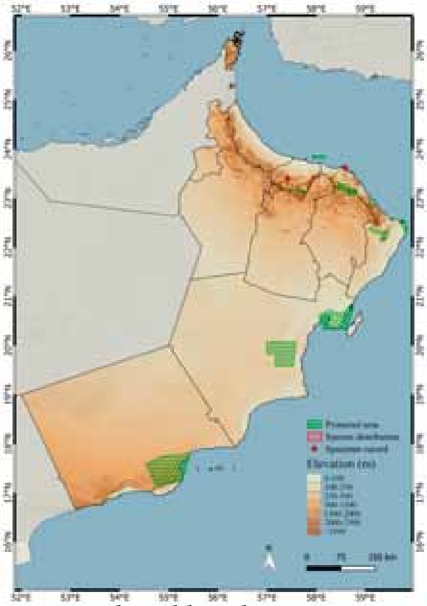

Indotyphlops braminus

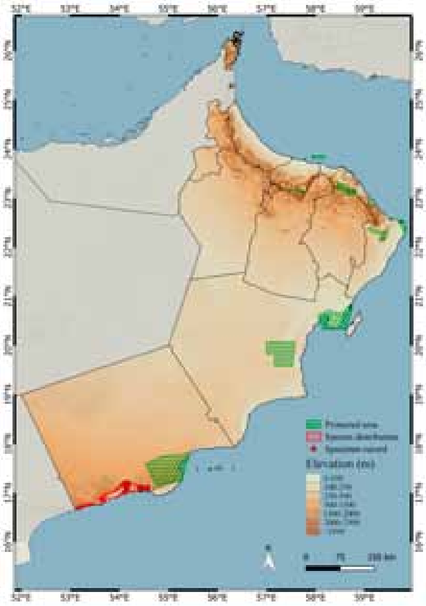

Bitis arietans

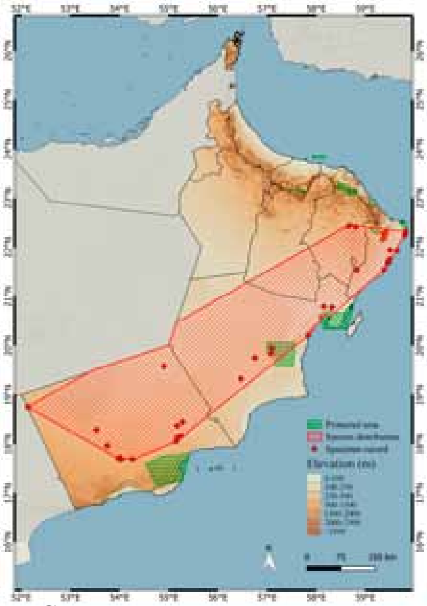

Cerastes gasperettii gasperettii

S134: Maps of species' distribution defined using minimum convex polygons

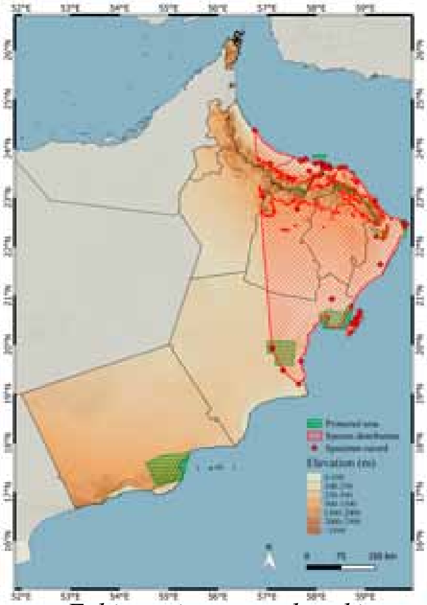

Echis carinatus sochureki

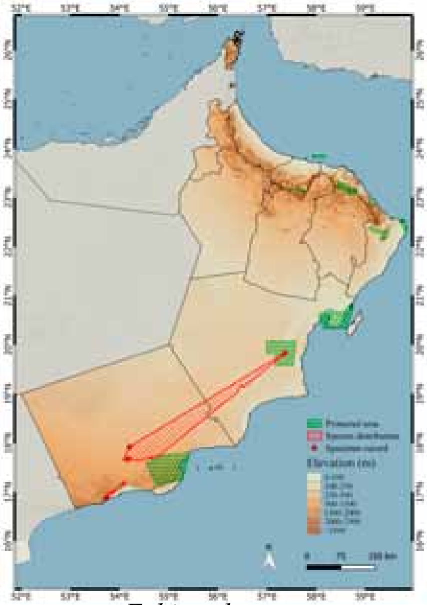

Echis coloratus

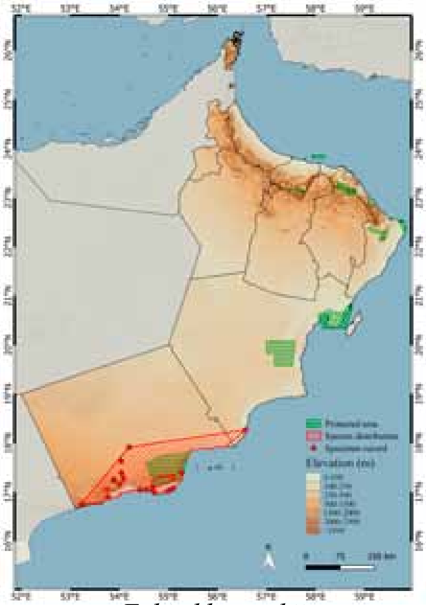

Echis khosatzkii

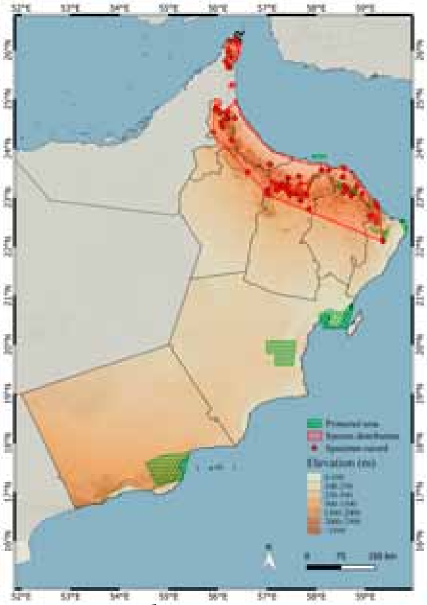

Echis omanensis

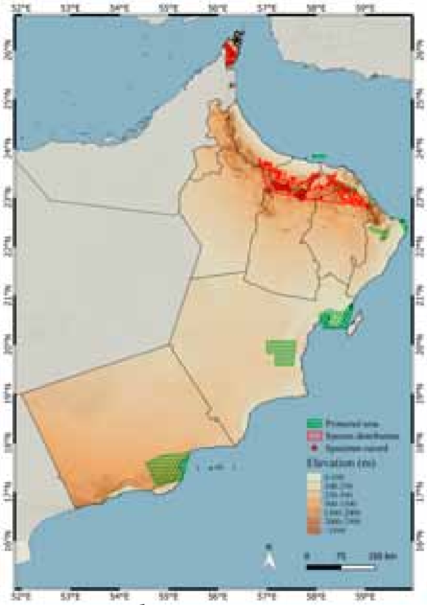

Pseudocerastes persicus

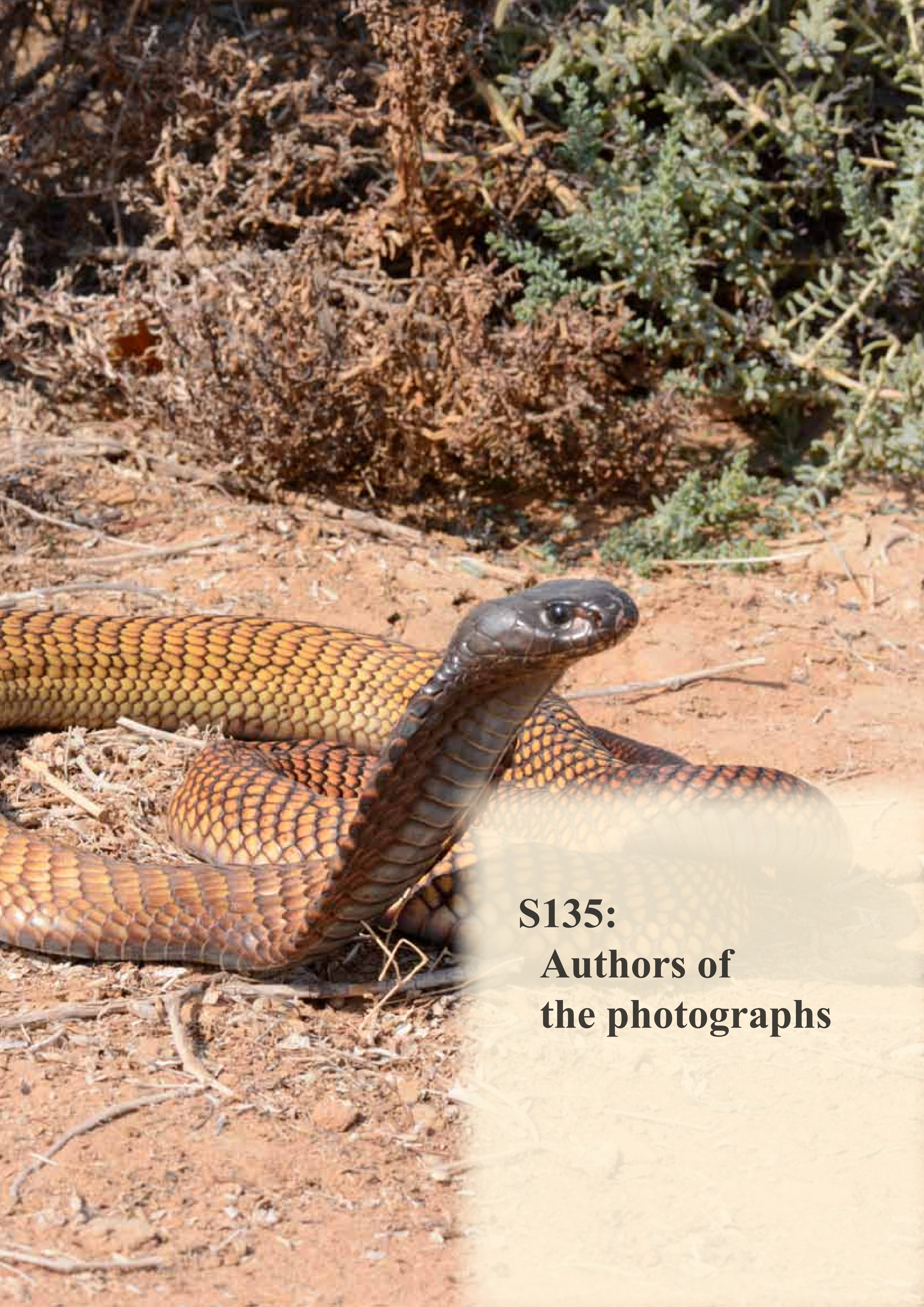

S135: Authors of the photographs

S135: Authors of the photographs

Authors list of photograph with the page, and species' photos between contents.

Amat, Fèlix (9): pg 14, pg 15, pg 17, pg 32, pg 48, pg 49, pg 55, pg 56 , pg 59.

Carranza, Salvador (47): pg 10, pg 21, pg 22, pg 24, pg 25, pg 26, pg 28, pg 29, pg 30, pg 34, pg 35, pg 36, pg 37, pg 41, pg 42, pg 43, pg 44, pg 50, pg 51, pg 52, pg 53, pg 54, pg 57, pg 58, pg 60, pg 61, pg 62, pg 63, pg 64, pg 65, pg 66, pg 69, pg 71, pg 73, pg 87, pg 88, pg 89, pg 90, pg 92, pg 98, pg 100, pg 103, pg 104, pg 105, pg 107, pg 108, pg 109.

Donaire, David (1): pg 80.

Els, Johannes (4): pg 11, pg 94, pg 96, pg 99.

Frenández, Daniel (8): pg 27, pg 45, pg 67, pg 72, pg 74, pg 79, pg 91, pg 97.

Gardner, Andrew (2): pg 23, pg70.

Gebhart, Jürgen (5): pg 19, pg 68, pg 81, pg 86, pg 101.

Gower, David (1): pg 77.

Martínez, Gabriel (2): pg 38, pg 93.

Mazuch, Tomas (2): pg 20, pg 106.

Mozaffari, Omid (1): pg 82.

Pierson, Todd (1): pg 102.

Pyron, Alex (1): pg 31.

Sampiano, Filipa (1): pg 77.

Sindaco, Roberto (12): pg 9, pg 12, pg 33, pg 39, pg 40, pg 46, pg 47, pg 76, pg 78, pg 83, pg 85, pg 95.

Šmíd, Jiri (5): pg 13, pg 16, pg 18, pg 75, pg 84.

Cover page: *Asaccus arnoldi* (Carranza, Salvador)

Pg 7: *Trapelus flavimaculatus* (Šmíd, Jiri)

Pg 111: *Ptyodactylus dhofarensis* (Carranza, Salvador)

Pg 115: *Asaccus platyrhynchus* (Carranza, Salvador)

Pg 133: *Hemidactylus lemurinus* (Carranza, Salvador)

Pg 141: *Chamaeleo arabicus* (Carranza, Salvador)

Pg 157: *Naja arabica* (Gebhart, Jürgen)
