# Supplementary figures and images for: Diversity, distribution and conservation of the terrestrial reptiles of Oman (Sauropsida, Squamata)
Source: PLoS One. 2018 Feb 7;13(2):e0190389. doi: 10.1371/journal.pone.0190389 (PMC5802441; doi:10.1371/journal.pone.0190389)

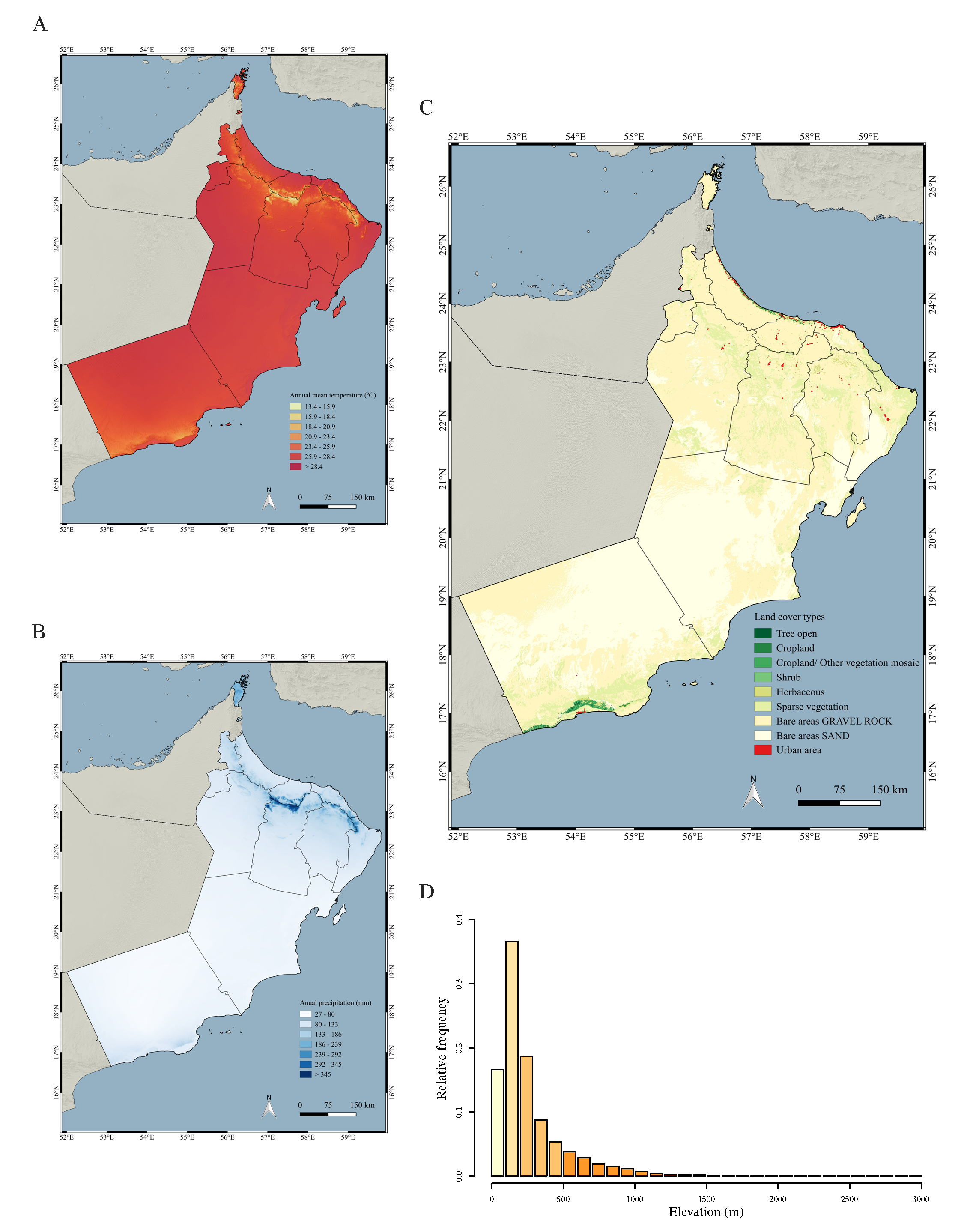

Supplement: S1 Fig — (A) Map of the annual mean temperatures in °C (BIO1); (B) Map of the annual precipitation in mm (BIO12); (C) Map of land cover types of the year 2008; (D) Graph of the elevation frequency dividing Oman into intervals of 100 m. Details in the Material and Methods section. (TIFF) [file pone.0190389.s004.tiff]

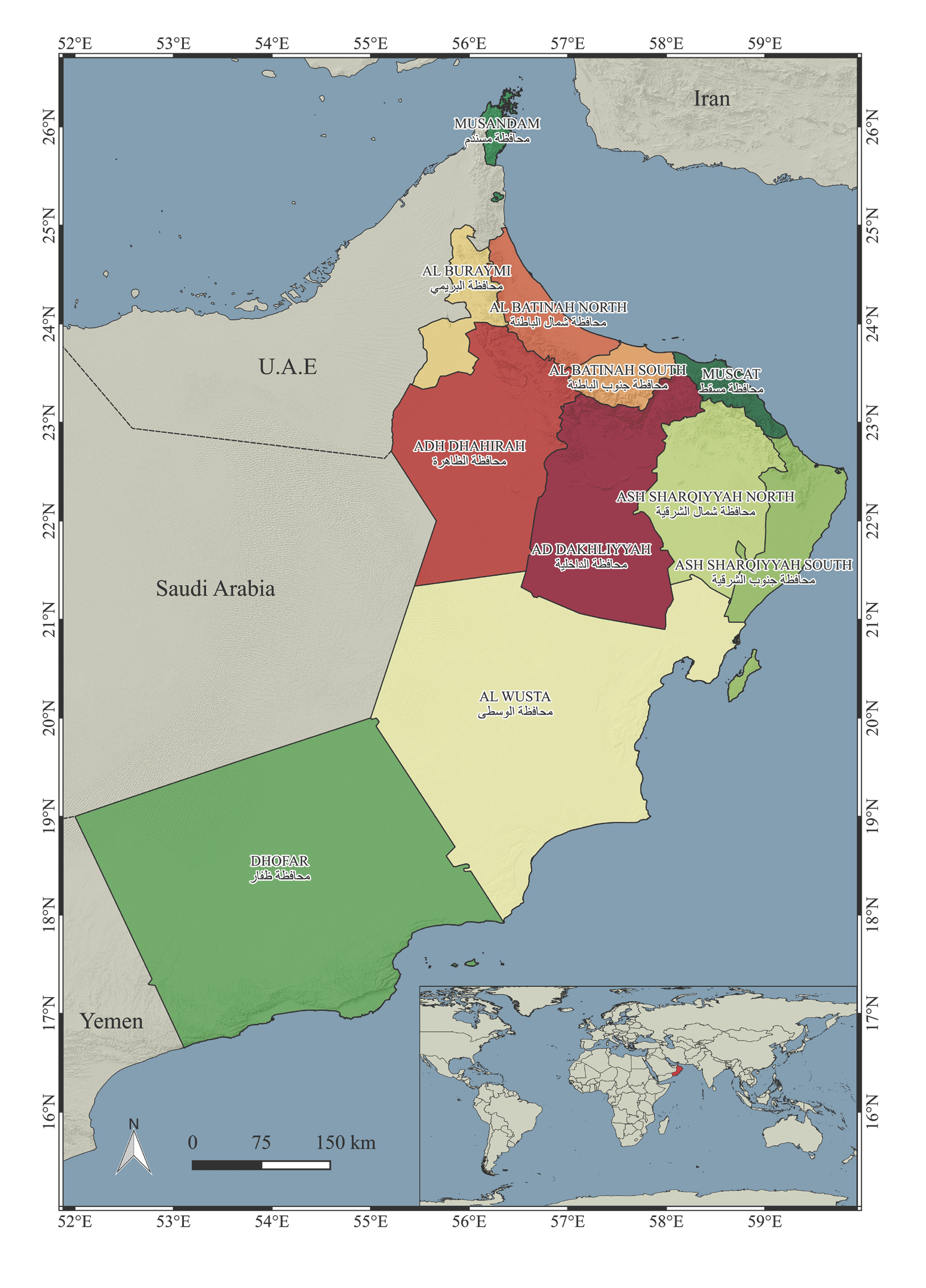

Supplement: S2 Fig — The map shows the limits of the 11 governorates. Inlet: Location of Oman (red) in the world map. (TIFF) [file pone.0190389.s005.tiff]

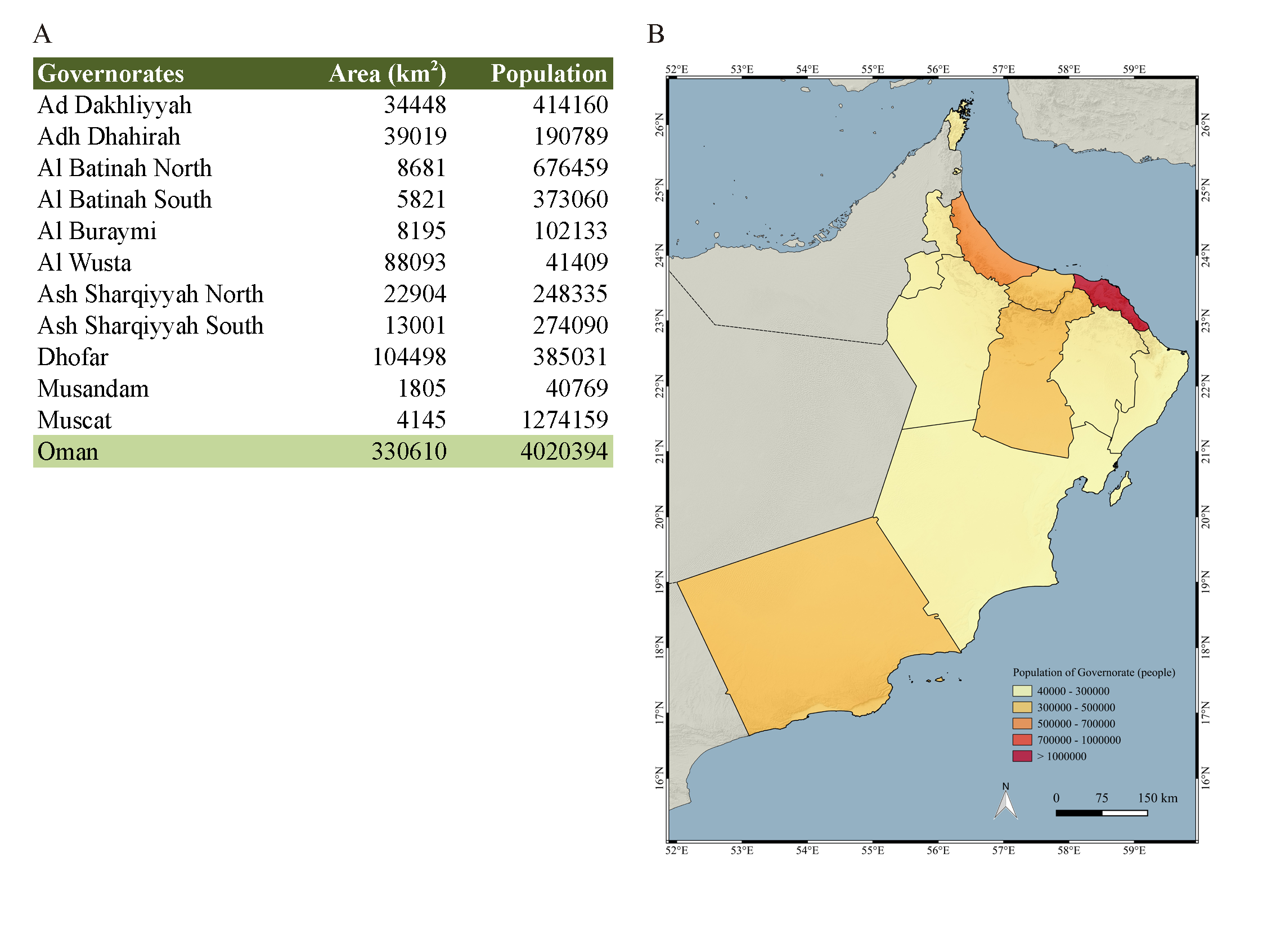

Supplement: S3 Fig — (A) Table indicating the area and population of each governorate; (B) The same information presented visually on a map. (TIF) [file pone.0190389.s006.tif]

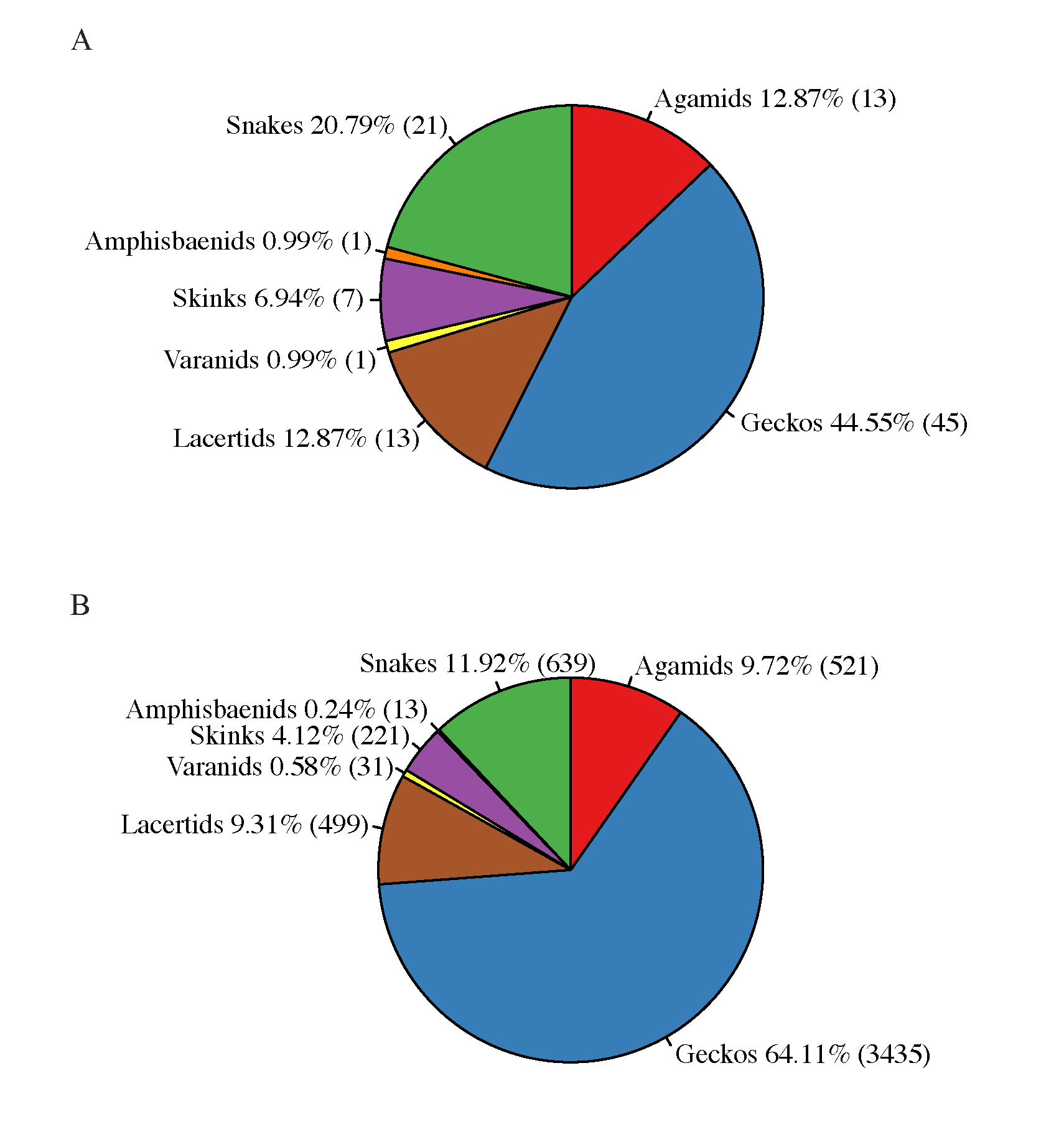

Supplement: S4 Fig — Percentage of the total number of (A) species and (B) observations in the seven main taxonomic groups of Oman reptiles in the database (between parenthesis, the respective number of observation). (TIFF) [file pone.0190389.s007.tiff]

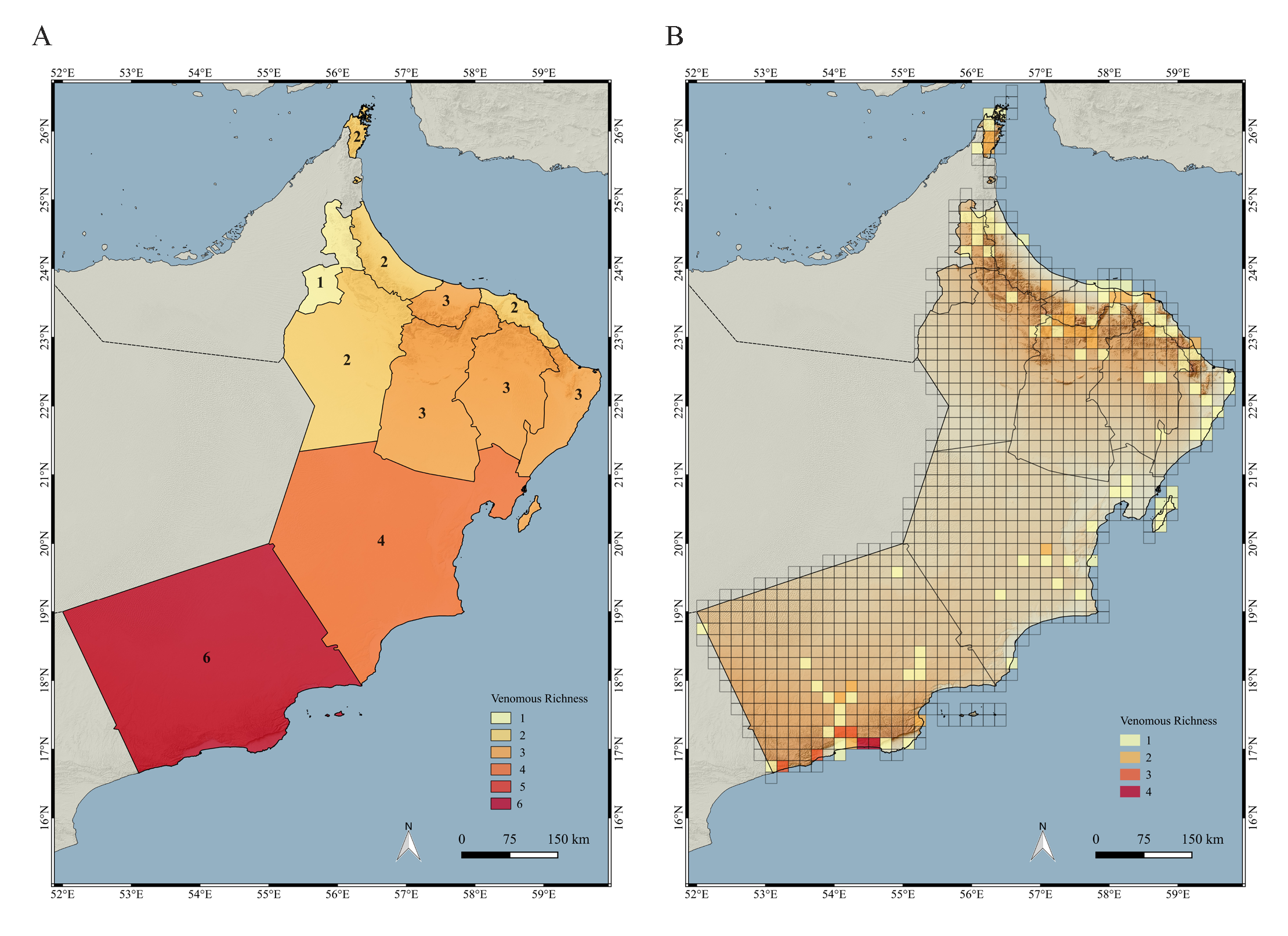

Supplement: S5 Fig — (A) Venomous species richness by governorate; (B) venomous species richness by grids of 10 arc-minutes of latitude and longitude. (TIFF) [file pone.0190389.s008.tiff]

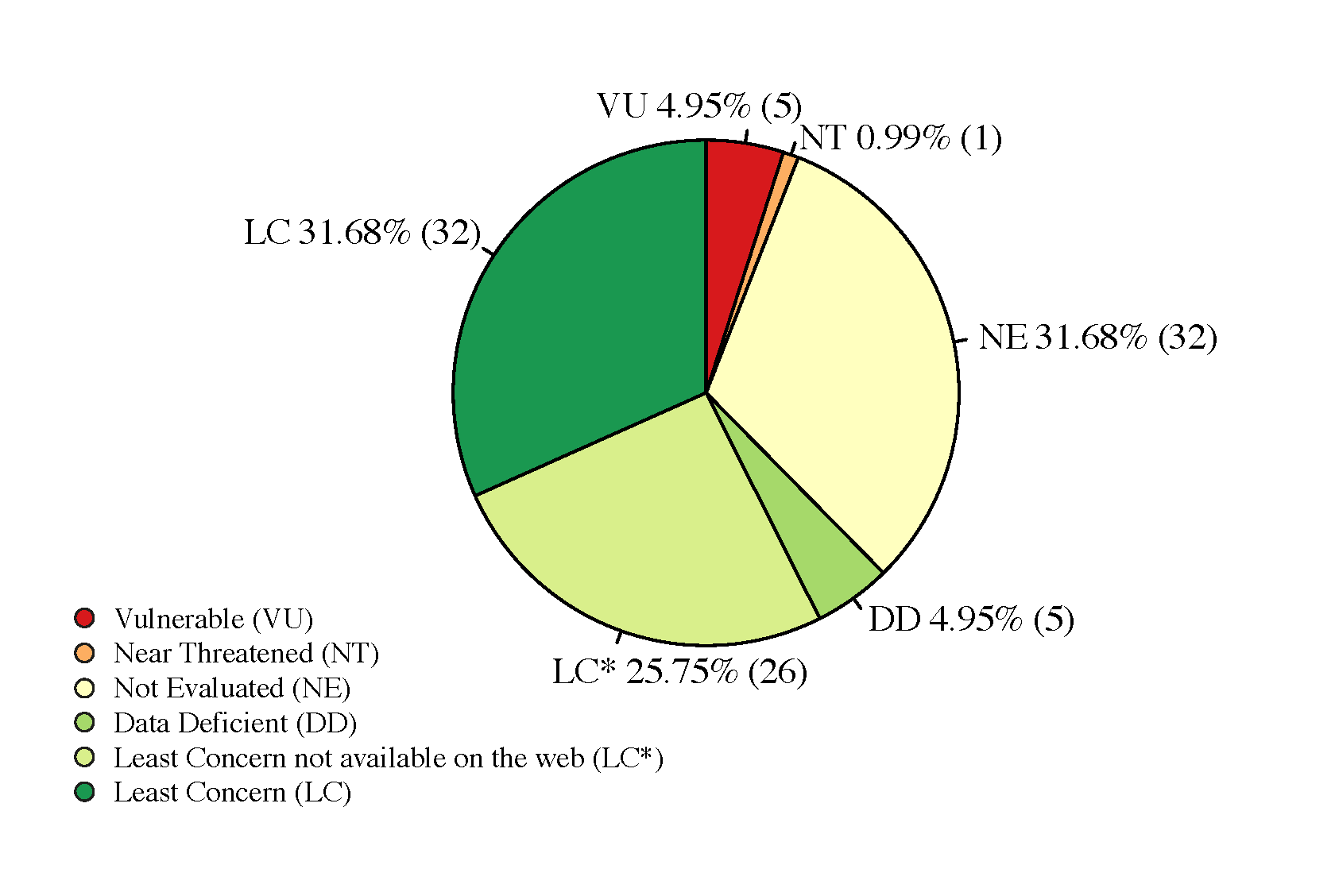

Supplement: S6 Fig — LC* indicates species that have been evaluated by Cox et al. (2012) [19] but are still pending of final approval from IUCN and publication on the web of the IUCN Red List of Threatened species (http://www.iucnredlist.org/)). (TIFF) [file pone.0190389.s009.tiff]

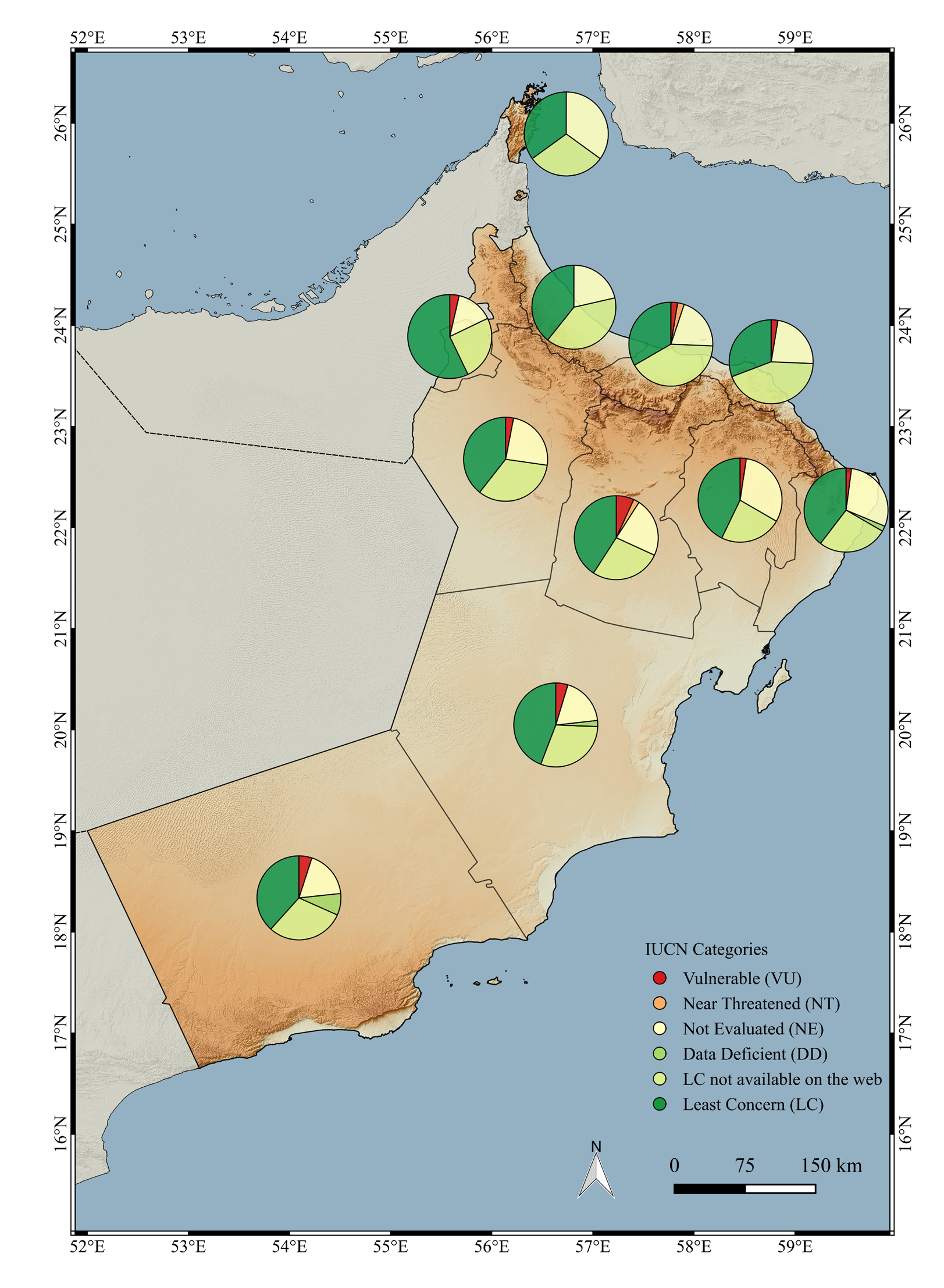

Supplement: S7 Fig — “LC not available on the web” indicates species that have been evaluated by Cox et al. (2012) [19] but are still pending of final approval from IUCN and publication on the web of the IUCN Red List of Threatened species (http://www.iucnredlist.org/)). (TIFF) [file pone.0190389.s010.tiff]
