# Supplementary material for: Diversity, distribution and conservation of the terrestrial reptiles of Oman (Sauropsida, Squamata)
Source: PLoS One. 2018 Feb 7;13(2):e0190389. doi: 10.1371/journal.pone.0190389 (PMC5802441; doi:10.1371/journal.pone.0190389)
Supplement: S1 Table — Number and percentage of the total number of species, endemic species, venomous species, threatened species, species not evaluated (NE) by the IUCN Red List of Threatened species organized by governorates, protected areas and islands or island groups. The last two columns show the area of each governorate, protected area and island or island group and the percentage of this area with respect to the total area of Oman. (DOCX) [file pone.0190389.s011.docx]

**S1 Table.** **Information on the 101 species of Oman terrestrial reptiles.** Number and percentage of the total number of species, endemic species, venomous species, threatened species, species not evaluated (NE) by the IUCN Red List of Threatened species organized by governorates, protected areas and islands or island groups. The last two columns show the area of each governorate, protected area and island or island group and the percentage of this area with respect to the total area of Oman.

|  | | **Species** | | **Endemic species** | | | **Venomous** | | **Threatened species** | | **Not Evaluated (NE)** | | | **Area (Km^2^)** | |
| --- | --- | --- | --- | --- | --- | --- | --- | --- | --- | --- | --- | --- | --- | --- | --- |
| **Governorates / Oman** | **101** | | **100%** | **20** | **100%** | **9** | | **100%** | **5** | **100%** | | **32** | **100%** | **330610,00** | **100%** |
| Ad Dakhliyyah | 44 | | 43,56% | 6 | 30,00% | 3 | | 33,33% | 3 | 60,00% | | 10 | 31,25% | 34448,00 | 10,42% |
| Adh Dhahirah | 33 | | 32,67% | 3 | 15,00% | 2 | | 22,22% | 1 | 20,00% | | 8 | 25,00% | 39019,00 | 11,80% |
| Al Batinah North | 28 | | 27,72% | 0 | 0,00% | 2 | | 22,22% | 0 | 0,00% | | 6 | 18,75% | 8681,00 | 2,63% |
| Al Batinah South | 40 | | 39,60% | 4 | 20,00% | 3 | | 33,33% | 1 | 20,00% | | 8 | 25,00% | 5821,00 | 1,76% |
| Al Buraymi | 28 | | 27,72% | 0 | 0,00% | 1 | | 11,11% | 1 | 20,00% | | 4 | 12,50% | 8195,00 | 2,48% |
| Al Wusta | 43 | | 42,57% | 5 | 25,00% | 4 | | 44,44% | 2 | 40,00% | | 8 | 25,00% | 88093,00 | 26,65% |
| Ash Sharqiyyah North | 42 | | 41,58% | 8 | 40,00% | 3 | | 33,33% | 1 | 20,00% | | 13 | 40,63% | 22904,00 | 6,93% |
| Ash Sharqiyyah South | 48 | | 47,52% | 9 | 45,00% | 3 | | 33,33% | 1 | 20,00% | | 14 | 43,75% | 13001,00 | 3,93% |
| Dhofar | 60 | | 59,41% | 5 | 25,00% | 6 | | 66,67% | 3 | 60,00% | | 11 | 34,38% | 104498,00 | 31,61% |
| Musandam | 20 | | 19,80% | 0 | 0,00% | 2 | | 22,22% | 0 | 0,00% | | 7 | 21,88% | 1805,00 | 0,55% |
| Muscat | 39 | | 38,61% | 5 | 25,00% | 2 | | 22,22% | 1 | 20,00% | | 9 | 28,13% | 4145,00 | 1,25% |
| **Protected Areas** | **64** | | **63,37%** | **10** | **50,00%** | **7** | | **77,78%** | **3** | **60,00%** | | **18** | **56,25%** | **12916,52** | **3,91%** |
| Al Sareen | 12 | | 11,88% | 3 | 15,00% | 2 | | 22,22% | 0 | 0,00% | | 7 | 21,88% | 785,10 | 0,2375% |
| Ras Al Shajer | 3 | | 2,97% | 0 | 0,00% | 0 | | 0,00% | 0 | 0,00% | | 2 | 6,25% | 102,07 | 0,0309% |
| Khawr Salalah | 0 | | 0,00% | 0 | 0,00% | 0 | | 0,00% | 0 | 0,00% | | 0 | 0,00% | 0,67 | 0,0002% |
| Al Wusta Wildlife Sanctuary | 21 | | 20,79% | 1 | 5,00% | 3 | | 33,33% | 2 | 40,00% | | 2 | 6,25% | 3013,24 | 0,9114% |
| Dimaniyat Islands | 4 | | 3,96% | 0 | 0,00% | 0 | | 0,00% | 0 | 0,00% | | 1 | 3,13% | 233,64 | 0,0707% |
| Turtle Reserve | 17 | | 16,83% | 2 | 10,00% | 2 | | 22,22% | 0 | 0,00% | | 4 | 12,50% | 302,25 | 0,0914% |
| Jabal Samhan | 17 | | 16,83% | 1 | 5,00% | 2 | | 22,22% | 1 | 20,00% | | 6 | 18,75% | 5057,49 | 1,5297% |
| Khawr Mughsayl | 0 | | 0,00% | 0 | 0,00% | 0 | | 0,00% | 0 | 0,00% | | 0 | 0,00% | 0,16 | - |
| Khawr Baleed | 0 | | 0,00% | 0 | 0,00% | 0 | | 0,00% | 0 | 0,00% | | 0 | 0,00% | 0,77 | 0,0002% |
| Khawr Sawli | 0 | | 0,00% | 0 | 0,00% | 0 | | 0,00% | 0 | 0,00% | | 0 | 0,00% | 0,83 | 0,0003% |
| Khawr Dahareez | 6 | | 5,94% | 0 | 0,00% | 0 | | 0,00% | 0 | 0,00% | | 2 | 6,25% | 0,81 | 0,0002% |
| Khawr Taqah | 0 | | 0,00% | 0 | 0,00% | 0 | | 0,00% | 0 | 0,00% | | 0 | 0,00% | 0,97 | 0,0003% |
| Khawr Rawri | 5 | | 4,95% | 1 | 5,00% | 0 | | 0,00% | 0 | 0,00% | | 4 | 12,50% | 0,87 | 0,0003% |
| Khawr Awqad | 0 | | 0,00% | 0 | 0,00% | 0 | | 0,00% | 0 | 0,00% | | 0 | 0,00% | 0,27 | 0,0001% |
| Khawr Qurum Al Sagher | 0 | | 0,00% | 0 | 0,00% | 0 | | 0,00% | 0 | 0,00% | | 0 | 0,00% | 0,04 | - |
| Khawr Qurum Al Kabeer | 0 | | 0,00% | 0 | 0,00% | 0 | | 0,00% | 0 | 0,00% | | 0 | 0,00% | 0,11 | - |
| Al Saleel | 8 | | 7,92% | 0 | 0,00% | 0 | | 0,00% | 0 | 0,00% | | 0 | 0,00% | 159,43 | 0,0482% |
| Al Khawair | 1 | | 0,99% | 0 | 0,00% | 0 | | 0,00% | 0 | 0,00% | | 0 | 0,00% | 0,32 | 0,0001% |
| Jebel Akhdar | 7 | | 6,93% | 1 | 5,00% | 1 | | 11,11% | 0 | 0,00% | | 2 | 6,25% | 133,06 | 0,0402% |
| Al Qurum Ramsar Site | 5 | | 4,95% | 0 | 0,00% | 0 | | 0,00% | 0 | 0,00% | | 0 | 0,00% | 1,91 | 0,0006% |
| Al Wusta Wetland Reserve | 13 | | 12,87% | 3 | 15,00% | 0 | | 0,00% | 0 | 0,00% | | 3 | 9,38% | 2809,10 | 0,8497% |
| Jebel Qahwan | 4 | | 3,96% | 2 | 10,00% | 0 | | 0,00% | 0 | 0,00% | | 4 | 12,50% | 313,41 | 0,0948% |
| **Islands** | **29** | | **28,71%** | **5** | **25,00%** | **1** | | **11,11%** | **1** | **20,00%** | | **9** | **28,13%** | **809,34** | **0,24%** |
| Daymaniyat Islands | 4 | | 3,96% | 0 | 0,00% | 0 | | 0,00% | 0 | 0,00% | | 1 | 3,13% | 1,24 | 0,0004% |
| Sawadi Islands | 4 | | 3,96% | 0 | 0,00% | 1 | | 11,11% | 0 | 0,00% | | 1 | 3,13% | 0,31 | 0,0001% |
| Al Wusta Islands | 4 | | 3,96% | 2 | 10,00% | 0 | | 0,00% | 0 | 0,00% | | 2 | 6,25% | 12,91 | 0,0039% |
| Masirah Islands | 19 | | 18,81% | 3 | 15,00% | 1 | | 11,11% | 1 | 20,00% | | 3 | 9,38% | 700,16 | 0,2118% |
| Hallaniyyat Islands | 6 | | 5,94% | 1 | 5,00% | 0 | | 0,00% | 0 | 0,00% | | 3 | 9,38% | 82,30 | 0,0249% |
| Musandam Islands | 3 | | 2,97% | 0 | 0,00% | 0 | | 0,00% | 0 | 0,00% | | 1 | 3,13% | 12,42 | 0,0038% |
