# Supplementary material for: Diversity, distribution and conservation of the terrestrial reptiles of Oman (Sauropsida, Squamata)
Source: PLoS One. 2018 Feb 7;13(2):e0190389. doi: 10.1371/journal.pone.0190389 (PMC5802441; doi:10.1371/journal.pone.0190389)
Supplement: S2 Table — Number of occupied cells of 4 km2 and Area of Occupancy (AOO) calculated for each species according to the IUCN Red List Guidelines 2016. (DOCX) [file pone.0190389.s012.docx]

**S2_Table. Area of Occupancy at 4 km^2^ of all 101 terrestrial reptiles of Oman.** Number of occupied cells of 4 km^2^ and Area of Occupancy (AOO) calculated for each species according to the IUCN Red List Guidelines 2016.

| **Species** | **No. occupied cells** | **AOO (Km^2^)** |
| --- | --- | --- |
| *Ablepharus pannonicus* | 15 | 60 |
| *Acanthocercus adramitanus* | 14 | 56 |
| *Acanthodactylus blanfordii* | 21 | 84 |
| *Acanthodactylus boskianus* | 23 | 92 |
| *Acanthodactylus felicis* | 17 | 68 |
| *Acanthodactylus haasi* | 6 | 24 |
| *Acanthodactylus masirae* | 25 | 100 |
| *Acanthodactylus opheodurus* | 23 | 92 |
| *Acanthodactylus schmidti* | 49 | 196 |
| *Asaccus gallagheri* | 24 | 96 |
| *Asaccus gardneri* | 16 | 64 |
| *Asaccus margaritae* | 4 | 16 |
| *Asaccus montanus* | 17 | 68 |
| *Asaccus platyrhynchus* | 25 | 100 |
| *Asaccus arnoldi* | 10 | 40 |
| *Atractaspis andersonii* | 4 | 16 |
| *Bitis arietans* | 14 | 56 |
| *Bunopus tuberculatus* | 73 | 292 |
| *Calotes versicolor* | 23 | 92 |
| *Cerastes gasperettii gasperettii* | 34 | 136 |
| *Chalcides ocellatus ocellatus* | 20 | 80 |
| *Chamaeleo arabicus* | 35 | 140 |
| *Cyrtopodion scabrum* | 11 | 44 |
| *Diplometopon zarudnyi* | 12 | 48 |
| *Echis carinatus sochureki* | 42 | 168 |
| *Echis coloratus* | 4 | 16 |
| *Echis khosatzkii* | 18 | 72 |
| *Echis omanensis* | 58 | 232 |
| *Eryx jayakari* | 18 | 72 |
| *Hemidactylus alkiyumii* | 42 | 168 |
| *Hemidactylus endophis* | 1 | 4 |
| *Hemidactylus festivus* | 19 | 76 |
| *Hemidactylus flaviviridis* | 39 | 156 |
| *Hemidactylus hajarensis* | 37 | 148 |
| *Hemidactylus inexpectatus* | 13 | 52 |
| *Hemidactylus lemurinus* | 8 | 32 |
| *Hemidactylus leschenaultii* | 2 | 8 |
| *Hemidactylus luqueorum* | 21 | 84 |
| *Hemidactylus masirahensis* | 5 | 20 |
| *Hemidactylus minutus* | 33 | 132 |
| *Hemidactylus paucituberculatus* | 23 | 92 |
| *Hemidactylus persicus* | 1 | 4 |
| *Hemidactylus robustus* | 62 | 248 |
| *Hemidactylus* sp. | 1 | 4 |
| *Heremites septemtaeniatus* | 2 | 8 |
| *Indotyphlops braminus* | 2 | 8 |
| *Lytorhynchus diadema diadema* | 19 | 76 |
| *Mesalina adramitana* | 84 | 336 |
| *Mesalina ayunensis* | 5 | 20 |
| *Mesalina* sp. 1 | 4 | 16 |
| *Mesalina* sp. 2 | 1 | 4 |

| **Species** | **No. occupied cells** | **AOO (Km2)** |
| --- | --- | --- |
| *Myriopholis macrorhyncha* | 14 | 56 |
| *Myriopholis nursii* | 3 | 12 |
| *Naja arabica* | 16 | 64 |
| *Omanosaura cyanura* | 33 | 132 |
| *Omanosaura jayakari* | 101 | 404 |
| *Phrynocephalus arabicus* | 15 | 60 |
| *Phrynocephalus maculatus* | 14 | 56 |
| *Phrynocephalus sakoi* | 24 | 96 |
| *Platyceps rhodorachis rhodorachis* | 121 | 484 |
| *Platyceps thomasi* | 11 | 44 |
| *Pristurus carteri* | 193 | 772 |
| *Pristurus celerrimus* | 111 | 444 |
| *Pristurus gallagheri* | 38 | 152 |
| *Pristurus minimus* | 115 | 460 |
| *Pristurus rupestris rupestris* | 142 | 568 |
| *Pristurus* sp. 1 | 142 | 568 |
| *Pristurus* sp. 2 | 20 | 80 |
| *Pristurus* sp. 3 | 60 | 240 |
| *Pristurus* sp. 4 | 1 | 4 |
| *Pristurus* sp. 5 | 58 | 232 |
| *Psammophis schokari* | 71 | 284 |
| *Pseudoceramodactylus khobarensis* | 9 | 36 |
| *Pseudocerastes persicus* | 21 | 84 |
| *Pseudotrapelus dhofarensis* | 66 | 264 |
| *Pseudotrapelus jensvindumi* | 91 | 364 |
| *Ptyodactylus dhofarensis* | 38 | 152 |
| *Ptyodactylus orlovi* | 120 | 480 |
| *Ptyodactylus ruusaljibalicus* | 20 | 80 |
| *Rhagerhis moilensis* | 25 | 100 |
| *Rhynchocalamus arabicus* | 2 | 8 |
| *Scincus mitranus* | 61 | 244 |
| *Scincus scincus conirostris* | 1 | 4 |
| *Spalerosophis diadema cliffordii* | 17 | 68 |
| *Stenodactylus arabicus* | 16 | 64 |
| *Stenodactylus doriae* | 50 | 200 |
| *Stenodactylus leptocosymbotes* | 107 | 428 |
| *Stenodactylus sharqiyahensis* | 31 | 124 |
| *Telescopus dhara dhara* | 28 | 112 |
| *Trachydactylus hajarensis* | 75 | 300 |
| *Trachydactylus spatalurus* | 6 | 24 |
| *Trachylepis brevicollis* | 23 | 92 |
| *Trachylepis tessellata* | 54 | 216 |
| *Trapelus flavimaculatus* | 58 | 232 |
| *Tropiocolotes scortecci* | 14 | 56 |
| *Tropiocolotes* sp. | 7 | 28 |
| *Uromastyx aegyptia leptieni* | 9 | 36 |
| *Uromastyx aegyptia microlepis* | 34 | 136 |
| *Uromastyx benti* | 9 | 36 |
| *Uromastyx thomasi* | 12 | 48 |
| *Varanus griseus* | 31 | 124 |
