# Supplementary material for: Diversity, distribution and conservation of the terrestrial reptiles of Oman (Sauropsida, Squamata)
Source: PLoS One. 2018 Feb 7;13(2):e0190389. doi: 10.1371/journal.pone.0190389 (PMC5802441; doi:10.1371/journal.pone.0190389)
Supplement: S3 Table — The two variables with the highest loadings values in both PC1 and PC2 are highlighted in bold. (DOCX) [file pone.0190389.s013.docx]

**S3 Table. Loadings, eigenvalues, and variance explained by the two first components retained from the Principal Component Analysis (PCA)** **performed on the 12 bioclimatic variables used in this study.** The two variables with the highest loadings values in both PC1 and PC2 are highlighted in bold.

BIO1 = Annual Mean Temperature, BIO4 = Temperature Seasonality, BIO5 = Max Temperature of Warmest Month, BIO6 = Min Temperature of Coldest Month, BIO7 = Temperature Annual Range, BIO10 = Mean Temperature of Warmest Quarter, BIO11 = Mean Temperature of Coldest Quarter, BIO12 = Annual Precipitation, BIO13 = Precipitation of Wettest Month, BIO16 = Precipitation of Wettest Quarter, BIO18 = Precipitation of Warmest Quarter, BIO19 = Precipitation of Coldest Quarter

| **Climatic variables** | **PC1** | **PC2** |
| --- | --- | --- |
| BIO 1 | 0.33681386 | -0.21530059 |
| BIO 4 | -0.19218433 | **-0.47188112** |
| BIO 5 | 0.23244799 | **-0.43976101** |
| BIO 6 | 0.28214375 | 0.09814554 |
| BIO 7 | -0.06479644 | -0.42614351 |
| BIO 10 | 0.22753366 | -0.40755087 |
| BIO 11 | **0.36376038** | 0.03073937 |
| BIO 12 | **-0.36520793** | -0.02214994 |
| BIO 13 | -0.32512623 | -0.19658534 |
| BIO 16 | -0.33935509 | -0.15171354 |
| BIO 18 | -0.28819923 | 0.26795269 |
| BIO 19 | -0.30064616 | -0.21297557 |
| Eigenvalues | 2.5811 | 1.7960 |
| Variance explained (%) | 55.52 | 26.88 |
