# Supplementary material for: Diversity, distribution and conservation of the terrestrial reptiles of Oman (Sauropsida, Squamata)
Source: PLoS One. 2018 Feb 7;13(2):e0190389. doi: 10.1371/journal.pone.0190389 (PMC5802441; doi:10.1371/journal.pone.0190389)
Supplement: S4 Table — List of all 101 reptile species of Oman, showing if they are endemic or not, total area of extent of occurrence (defined by the presence-absence in every pixel of 1 km x 1 km), area of extent of occurrence inside protected areas and percentage of the area of extent of occurrence inside protected areas. (DOCX) [file pone.0190389.s014.docx]

| **Specie** | **Endemic** | **Total area (m^2^)** | **Area protected (m^2^)** | **Area protected (%)** |
| --- | --- | --- | --- | --- |
| *Ablepharus pannonicus* | NO | 1259408.55 | 83960.57 | 6.67 |
| *Acanthocercus adramitanus* | NO | 1427329.69 | 0.00 | 0.00 |
| *Acanthodactylus blanfordii* | NO | 1847132.54 | 167921.14 | 9.09 |
| *Acanthodactylus boskianus* | NO | 1931093.11 | 0.00 | 0.00 |
| *Acanthodactylus felicis* | NO | 1763171.97 | 83960.57 | 4.76 |
| *Acanthodactylus haasi* | NO | 503763.42 | 0.00 | 0.00 |
| *Acanthodactylus masirae* | YES | 2099014.25 | 251881.71 | 12.00 |
| *Acanthodactylus opheodurus* | NO | 2015053.68 | 0.00 | 0.00 |
| *Acanthodactylus schmidti* | NO | 4114067.93 | 167921.14 | 4.08 |
| *Asaccus gallagheri* | NO | 2099014.25 | 0.00 | 0.00 |
| *Asaccus gardneri* | NO | 1343369.12 | 0.00 | 0.00 |
| *Asaccus margaritae* | NO | 335842.28 | 0.00 | 0.00 |
| *Asaccus montanus* | YES | 1595250.83 | 0.00 | 0.00 |
| *Asaccus platyrhynchus* | YES | 2602777.67 | 0.00 | 0.00 |
| *Asaccus arnoldi* | YES | 923566.27 | 251881.71 | 27.27 |
| *Atractaspis andersonii* | NO | 335842.28 | 0.00 | 0.00 |
| *Bitis arietans* | NO | 1343369.12 | 0.00 | 0.00 |
| *Bunopus tuberculatus* | NO | 6297042.75 | 587723.99 | 9.33 |
| *Calotes versicolor* | NO | 2266935.39 | 83960.57 | 3.70 |
| *Cerastes gasperettii gasperettii* | NO | 2938619.95 | 335842.28 | 11.43 |
| *Chalcides ocellatus ocellatus* | NO | 1763171.97 | 251881.71 | 14.29 |
| *Chamaeleo arabicus* | NO | 3274462.23 | 167921.14 | 5.13 |
| *Cyrtopodion scabrum* | NO | 1007526.84 | 0.00 | 0.00 |
| *Diplometopon zarudnyi* | NO | 1007526.84 | 167921.14 | 16.67 |
| *Echis carinatus sochureki* | NO | 3610304.51 | 419802.85 | 11.63 |
| *Echis coloratus* | NO | 335842.28 | 83960.57 | 25.00 |
| *Echis khosatzkii* | NO | 1511290.26 | 83960.57 | 5.56 |
| *Echis omanensis* | NO | 5457437.05 | 335842.28 | 6.15 |
| *Eryx jayakari* | NO | 1595250.83 | 83960.57 | 5.26 |
| *Hemidactylus alkiyumii* | NO | 4198028.50 | 923566.27 | 22.00 |
| *Hemidactylus endophis* | YES | 83960.57 | 0.00 | 0.00 |
| *Hemidactylus festivus* | NO | 2015053.68 | 0.00 | 0.00 |
| *Hemidactylus flaviviridis* | NO | 3274462.23 | 335842.28 | 10.26 |
| *Hemidactylus hajarensis* | YES | 3358422.80 | 419802.85 | 12.50 |
| *Hemidactylus inexpectatus* | YES | 1091487.41 | 0.00 | 0.00 |
| *Hemidactylus lemurinus* | NO | 923566.27 | 0.00 | 0.00 |
| *Hemidactylus leschenaultii* | NO | 167921.14 | 0.00 | 0.00 |
| *Hemidactylus luqueorum* | YES | 2015053.68 | 83960.57 | 4.17 |
| *Hemidactylus masirahensis* | YES | 419802.85 | 0.00 | 0.00 |
| *Hemidactylus minutus* | NO | 3274462.23 | 419802.85 | 12.82 |
| *Hemidactylus paucituberculatus* | YES | 2099014.25 | 839605.70 | 40.00 |
| *Hemidactylus persicus* | NO | 83960.57 | 0.00 | 0.00 |
| *Hemidactylus robustus* | NO | 5457437.05 | 755645.13 | 13.85 |
| *Hemidactylus* sp. | YES | 83960.57 | 0.00 | 0.00 |
| *Heremites septemtaeniatus* | NO | 503763.42 | 83960.57 | 16.67 |
| *Indotyphlops braminus* | NO | 167921.14 | 0.00 | 0.00 |
| *Lytorhynchus diadema diadema* | NO | 1595250.83 | 83960.57 | 5.26 |
| *Mesalina adramitana* | NO | 7304569.59 | 755645.13 | 10.34 |
| *Mesalina ayunensis* | NO | 419802.85 | 0.00 | 0.00 |
| *Mesalina* sp. 1 | NO | 419802.85 | 83960.57 | 20.00 |
| *Mesalina* sp. 2 | YES | 83960.57 | 0.00 | 0.00 |

| **Specie** | **Endemic** | **Total area (m^2^)** | **Area**  **protected (m^2^)** | **Area protected (%)** |
| --- | --- | --- | --- | --- |
| *Myriopholis macrorhyncha* | NO | 1175447.98 | 167921.14 | 14.29 |
| *Myriopholis nursii* | NO | 251881.71 | 83960.57 | 33.33 |
| *Naja arabica* | NO | 1343369.12 | 167921.14 | 12.50 |
| *Omanosaura cyanura* | NO | 2854659.38 | 167921.14 | 5.88 |
| *Omanosaura jayakari* | NO | 9319623.27 | 335842.28 | 3.60 |
| *Phrynocephalus arabicus* | NO | 1259408.55 | 0.00 | 0.00 |
| *Phrynocephalus maculatus* | NO | 1175447.98 | 167921.14 | 14.29 |
| *Phrynocephalus sakoi* | YES | 2099014.25 | 83960.57 | 4.00 |
| *Platyceps rhodorachis rhodorachis* | NO | 10746952.96 | 671684.56 | 6.25 |
| *Platyceps thomasi* | NO | 923566.27 | 251881.71 | 27.27 |
| *Pristurus carteri* | NO | 17463798.56 | 3274462.23 | 18.75 |
| *Pristurus celerrimus* | NO | 10159228.97 | 419802.85 | 4.13 |
| *Pristurus gallagheri* | YES | 3442383.37 | 167921.14 | 4.88 |
| *Pristurus minimus* | NO | 9907347.26 | 1343369.12 | 13.56 |
| *Pristurus rupestris rupestris* | NO | 13265770.06 | 1259408.55 | 9.49 |
| *Pristurus* sp. 1 | NO | 13685572.91 | 2350895.96 | 17.18 |
| *Pristurus* sp. 2 | YES | 1847132.54 | 0.00 | 0.00 |
| *Pristurus* sp. 3 | NO | 5289515.91 | 0.00 | 0.00 |
| *Pristurus* sp. 4 | YES | 83960.57 | 83960.57 | 100.00 |
| *Pristurus* sp. 5 | YES | 4953673.63 | 335842.28 | 6.78 |
| *Psammophis schokari* | NO | 6129121.61 | 671684.56 | 10.96 |
| *Pseudoceramodactylus khobarensis* | NO | 5793279.33 | 1259408.55 | 21.74 |
| *Pseudocerastes persicus* | NO | 7976254.15 | 167921.14 | 2.11 |
| *Pseudotrapelus dhofarensis* | NO | 839605.70 | 419802.85 | 50.00 |
| *Pseudotrapelus jensvindumi* | NO | 1763171.97 | 251881.71 | 14.29 |
| *Ptyodactylus dhofarensis* | NO | 3778225.65 | 671684.56 | 17.78 |
| *Ptyodactylus orlovi* | NO | 10914874.10 | 755645.13 | 6.92 |
| *Ptyodactylus ruusaljibalicus* | NO | 1679211.40 | 0.00 | 0.00 |
| *Rhagerhis moilensis* | NO | 2099014.25 | 167921.14 | 8.00 |
| *Rhynchocalamus arabicus* | NO | 167921.14 | 0.00 | 0.00 |
| *Scincus mitranus* | NO | 83960.57 | 0.00 | 0.00 |
| *Scincus scincus conirostris* | NO | 5205555.34 | 335842.28 | 6.45 |
| *Spalerosophis diadema cliffordii* | NO | 1427329.69 | 167921.14 | 11.76 |
| *Stenodactylus arabicus* | NO | 1511290.26 | 0.00 | 0.00 |
| *Stenodactylus doriae* | NO | 4281989.07 | 251881.71 | 5.88 |
| *Stenodactylus leptocosymbotes* | NO | 9235662.70 | 923566.27 | 10.00 |
| *Stenodactylus sharqiyahensis* | YES | 2686738.24 | 83960.57 | 3.13 |
| *Telescopus dhara dhara* | NO | 2434856.53 | 251881.71 | 10.34 |
| *Trachydactylus hajarensis* | NO | 2182974.82 | 0.00 | 0.00 |
| *Trachydactylus spatalurus* | NO | 6381003.32 | 335842.28 | 5.26 |
| *Trachylepis brevicollis* | NO | 167921.14 | 83960.57 | 50.00 |
| *Trachylepis tessellata* | NO | 4617831.35 | 335842.28 | 7.27 |
| *Trapelus flavimaculatus* | NO | 4869713.06 | 167921.14 | 3.45 |
| *Tropiocolotes scortecci* | NO | 1259408.55 | 167921.14 | 13.33 |
| *Tropiocolotes* sp. | YES | 587723.99 | 0.00 | 0.00 |
| *Uromastyx aegyptia leptieni* | NO | 839605.70 | 0.00 | 0.00 |
| *Uromastyx aegyptia microlepis* | NO | 2854659.38 | 167921.14 | 5.88 |
| *Uromastyx benti* | NO | 839605.70 | 0.00 | 0.00 |
| *Uromastyx thomasi* | YES | 1091487.41 | 251881.71 | 23.08 |
| *Varanus griseus* | NO | 2602777.67 | 251881.71 | 9.68 |

**S4 Table.** **Information for the gap analysis with distribution records at a resolution of 1 km^2^**. List of all 101 reptile species of Oman, showing if they are endemic or no, total area of extent of occurrence (defined by the presence-absence in every pixel of 1 km x 1 km), area of extent of occurrence inside protected areas and percentage of the area of extent of occurrence inside protected areas.
