# Supplementary material for: Diversity, distribution and conservation of the terrestrial reptiles of Oman (Sauropsida, Squamata)
Source: PLoS One. 2018 Feb 7;13(2):e0190389. doi: 10.1371/journal.pone.0190389 (PMC5802441; doi:10.1371/journal.pone.0190389)
Supplement: S5 Table — List of all 101 reptile species of Oman, showing if they are endemic or not, total area of extent occurrence (defined by the MCP), area of extent of occurrence inside protected areas and the percentage of the area of extent of occurrence inside protected areas. (DOCX) [file pone.0190389.s015.docx]

| **Specie** | **Endemic** | **Total area (m^2^)** | **Area protected (m^2^)** | **Area protected (%)** |
| --- | --- | --- | --- | --- |
| *Ablepharus pannonicus* | NO | 723908034.50 | 9907347.26 | 1.37 |
| *Acanthocercus adramitanus* | NO | 107553490.20 | 0.00 | 0.00 |
| *Acanthodactylus blanfordii* | NO | 246592194.10 | 1511290.26 | 0.61 |
| *Acanthodactylus boskianus* | NO | 22928372258.00 | 505610552.50 | 2.21 |
| *Acanthodactylus felicis* | NO | 947662953.60 | 327194341.30 | 34.53 |
| *Acanthodactylus haasi* | NO | 2325204026.00 | 0.00 | 0.00 |
| *Acanthodactylus masirae* | YES | 3564210157.00 | 409559660.50 | 11.49 |
| *Acanthodactylus opheodurus* | NO | 17654808857.00 | 326018893.30 | 1.85 |
| *Acanthodactylus schmidti* | NO | 16221517966.00 | 174637985.60 | 1.08 |
| *Asaccus gallagheri* | NO | 1169906582.00 | 2602777.67 | 0.22 |
| *Asaccus gardneri* | NO | 54154567.65 | 0.00 | 0.00 |
| *Asaccus margaritae* | NO | 13937454.62 | 0.00 | 0.00 |
| *Asaccus montanus* | YES | 38957704.48 | 1679211.40 | 4.31 |
| *Asaccus platyrhynchus* | YES | 287480991.70 | 5877239.90 | 2.04 |
| *Asaccus arnoldi* | YES | 358763515.60 | 68259943.41 | 19.03 |
| *Atractaspis andersonii* | NO | 24516486.44 | 0.00 | 0.00 |
| *Bitis arietans* | NO | 148274366.60 | 419802.85 | 0.28 |
| *Bunopus tuberculatus* | NO | 25552224032.00 | 652037786.60 | 2.55 |
| *Calotes versicolor* | NO | 86395426.53 | 923566.27 | 1.07 |
| *Cerastes gasperettii gasperettii* | NO | 14935493916.00 | 267750257.70 | 1.79 |
| *Chalcides ocellatus ocellatus* | NO | 20921042951.00 | 406537079.90 | 1.94 |
| *Chamaeleo arabicus* | NO | 2788582411.00 | 437350609.10 | 15.68 |
| *Cyrtopodion scabrum* | NO | 8913505993.00 | 167921.14 | 0.00 |
| *Diplometopon zarudnyi* | NO | 7718663121.00 | 120819260.20 | 1.57 |
| *Echis carinatus sochureki* | NO | 8511922587.00 | 521898903.10 | 6.13 |
| *Echis coloratus* | NO | 1203490810.00 | 9655465.55 | 0.80 |
| *Echis khosatzkii* | NO | 2120256274.00 | 410819069.00 | 19.38 |
| *Echis omanensis* | NO | 4025069726.00 | 136771768.50 | 3.40 |
| *Eryx jayakari* | NO | 16105232577.00 | 94623562.39 | 0.59 |
| *Hemidactylus alkiyumii* | NO | 548178561.50 | 143068811.30 | 26.10 |
| *Hemidactylus endophis* | YES | 83960.57 | 0.00 | 0.00 |
| *Hemidactylus festivus* | NO | 1405499942.00 | 270017193.10 | 19.21 |
| *Hemidactylus flaviviridis* | NO | 20366399425.00 | 221655904.80 | 1.09 |
| *Hemidactylus hajarensis* | YES | 2172815591.00 | 146343273.50 | 6.74 |
| *Hemidactylus inexpectatus* | YES | 357588067.60 | 108309135.30 | 30.29 |
| *Hemidactylus lemurinus* | NO | 37782256.50 | 83960.57 | 0.22 |
| *Hemidactylus leschenaultii* | NO | 167921.14 | 0.00 | 0.00 |
| *Hemidactylus luqueorum* | YES | 168760745.70 | 7892293.58 | 4.68 |
| *Hemidactylus masirahensis* | YES | 10662992.39 | 0.00 | 0.00 |
| *Hemidactylus minutus* | NO | 6974352668.00 | 826088048.20 | 11.84 |
| *Hemidactylus paucituberculatus* | YES | 265987085.80 | 54238528.22 | 20.39 |
| *Hemidactylus persicus* | NO | 83960.57 | 0.00 | 0.00 |
| *Hemidactylus robustus* | NO | 27084924237.00 | 952868508.90 | 3.52 |
| *Hemidactylus* sp. | YES | 83960.57 | 0.00 | 0.00 |
| *Heremites septemtaeniatus* | NO | 167921.14 | 83960.57 |  |
| *Indotyphlops braminus* | NO | 167921.14 | 0.00 | 0.00 |
| *Lytorhynchus diadema diadema* | NO | 18787772788.00 | 285298016.90 | 1.52 |
| *Mesalina adramitana* | NO | 27834524206.00 | 700315114.40 | 2.52 |
| *Mesalina ayunensis* | NO | 129383238.40 | 43155732.98 | 33.35 |
| *Mesalina* sp. 1 | NO | 34927597.12 | 3106541.09 | 8.89 |
| *Mesalina* sp. 2 | YES | 83960.57 | 0.00 | 0.00 |

| **Specie** | **Endemic** | **Total area (m^2^)** | **Area**  **protected (m^2^)** | **Area protected (%)** |
| --- | --- | --- | --- | --- |
| *Myriopholis macrorhyncha* | NO | 13687839845.00 | 416192545.50 | 3.04 |
| *Myriopholis nursii* | NO | 4869713.06 | 83960.57 | 1.72 |
| *Naja arabica* | NO | 269681350.80 | 8983780.99 | 3.33 |
| *Omanosaura cyanura* | NO | 2070047853.00 | 97898024.62 | 4.73 |
| *Omanosaura jayakari* | NO | 3445489911.00 | 113262808.90 | 3.29 |
| *Phrynocephalus arabicus* | NO | 6647242287.00 | 0.00 | 0.00 |
| *Phrynocephalus maculatus* | NO | 5945583804.00 | 200078038.30 | 3.37 |
| *Phrynocephalus sakoi* | YES | 1399706662.00 | 1259408.55 | 0.09 |
| *Platyceps rhodorachis rhodorachis* | NO | 28198157435.00 | 1086281855.00 | 3.85 |
| *Platyceps thomasi* | NO | 483696843.80 | 61878940.09 | 12.79 |
| *Pristurus carteri* | NO | 28009917837.00 | 1129269667.00 | 4.03 |
| *Pristurus celerrimus* | NO | 2296741392.00 | 17295877.42 | 0.75 |
| *Pristurus gallagheri* | YES | 329041473.80 | 11754479.80 | 3.57 |
| *Pristurus minimus* | NO | 21101222334.00 | 543644690.80 | 2.58 |
| *Pristurus rupestris rupestris* | NO | 3057508117.00 | 116705192.30 | 3.82 |
| *Pristurus* sp. 1 | NO | 8704192292.00 | 779070129.00 | 8.95 |
| *Pristurus* sp. 2 | YES | 32072937.74 | 0.00 | 0.00 |
| *Pristurus* sp. 3 | NO | 3434742958.00 | 87906716.79 | 2.56 |
| *Pristurus* sp. 4 | YES | 83960.57 | 83960.57 | 100.00 |
| *Pristurus* sp. 5 | YES | 3464968763.00 | 132069976.60 | 3.81 |
| *Psammophis schokari* | NO | 28067346866.00 | 1121545294.00 | 4.00 |
| *Pseudoceramodactylus khobarensis* | NO | 3172030335.00 | 40217113.03 | 1.27 |
| *Pseudocerastes persicus* | NO | 842460359.40 | 73297577.61 | 8.70 |
| *Pseudotrapelus dhofarensis* | NO | 5980763283.00 | 921971019.20 | 15.42 |
| *Pseudotrapelus jensvindumi* | NO | 6143226986.00 | 159189240.70 | 2.59 |
| *Ptyodactylus dhofarensis* | NO | 750103732.40 | 121658865.90 | 16.22 |
| *Ptyodactylus orlovi* | NO | 5407732393.00 | 167417376.60 | 3.10 |
| *Ptyodactylus ruusaljibalicus* | NO | 105118633.60 | 0.00 | 0.00 |
| *Rhagerhis moilensis* | NO | 17310234677.00 | 379417815.80 | 2.19 |
| *Rhynchocalamus arabicus* | NO | 167921.14 | 0.00 | 0.00 |
| *Scincus mitranus* | NO | 24060076781.00 | 513418885.60 | 2.13 |
| *Scincus scincus conirostris* | NO | 83960.57 | 0.00 | 0.00 |
| *Spalerosophis diadema cliffordii* | NO | 19438383245.00 | 876464390.20 | 4.51 |
| *Stenodactylus arabicus* | NO | 6991564585.00 | 0.00 | 0.00 |
| *Stenodactylus doriae* | NO | 20088070136.00 | 234082069.20 | 1.17 |
| *Stenodactylus leptocosymbotes* | NO | 27748968385.00 | 702833931.50 | 2.53 |
| *Stenodactylus sharqiyahensis* | YES | 2385319794.00 | 11334676.95 | 0.48 |
| *Telescopus dhara dhara* | NO | 27406409259.00 | 912987238.20 | 3.33 |
| *Trachydactylus hajarensis* | NO | 11543402927.00 | 390332689.90 | 3.38 |
| *Trachydactylus spatalurus* | NO | 151045065.40 | 3022580.52 | 2.00 |
| *Trachylepis brevicollis* | NO | 124681446.50 | 2770698.81 | 2.22 |
| *Trachylepis tessellata* | NO | 27104487050.00 | 1023395388.00 | 3.78 |
| *Trapelus flavimaculatus* | NO | 21022971083.00 | 266742730.90 | 1.27 |
| *Tropiocolotes scortecci* | NO | 4700868354.00 | 275978393.60 | 5.87 |
| *Tropiocolotes* sp. | YES | 341383677.60 | 2770698.81 | 0.81 |
| *Uromastyx aegyptia leptieni* | NO | 1436229510.00 | 0.00 | 0.00 |
| *Uromastyx aegyptia microlepis* | NO | 13716554360.00 | 264559756.10 | 1.93 |
| *Uromastyx benti* | NO | 44331180.96 | 0.00 | 0.00 |
| *Uromastyx thomasi* | YES | 2890846386.00 | 225182248.70 | 7.79 |
| *Varanus griseus* | NO | 21565608247.00 | 478239406.70 | 2.22 |

**S5 Table. Information for the gap analysis with distribution records using the minimum convex polygon (MCP) filtered by the species’ average altitude**. List of all 101 reptile species of Oman, showing if they are endemic or no, total area of extent occurrence (defined by the MCP), area of extent of occurrence inside protected areas and the percentage of the area of extent of occurrence inside protected areas
